# Integrative Analysis of Bulk RNA-Seq and Single-Cell RNA-Seq Unveils the Characteristics of the Immune Microenvironment and Prognosis Signature in Prostate Cancer

**DOI:** 10.1155/2022/6768139

**Published:** 2022-07-19

**Authors:** Ruisong Wang, Yaqian Xiao, Meisen Pan, Zhongyuan Chen, Pinhong Yang

**Affiliations:** ^1^College of Life and Environmental Sciences, Hunan University of Arts and Science, Changde 415000, Hunan, China; ^2^Changde Research Centre for Artificial Intelligence and Biomedicine, Changde 415000, Hunan, China; ^3^Furong College, Hunan University of Arts and Science, Changde 415000, Hunan, China; ^4^Medical College, Hunan University of Arts and Science, Changde 415000, Hunan, China; ^5^Affiliated Hospital of Hunan University of Arts and Science (The Maternal and Child Health Hospital), Changde 415000, Hunan, China

## Abstract

The immune microenvironment is a culmination of the collaborative effort of immune cells and is important in cancer development. The underlying mechanisms of the tumor immune microenvironment in regulating prostate cancer (PRAD) are unclear. In the current study, 144 natural killer cell-related genes were identified using differential expression, single-sample gene set enrichment analysis, and weighted gene coexpression network analysis. Furthermore, VCL, ACTA2, MYL9, MYLK, MYH11, TPM1, ACTG2, TAGLN, and FLNC were selected as hub genes via the protein-protein interaction network. Based on the expression patterns of the hub genes, endothelial, epithelial, and tissue stem cells were identified as key cell subpopulations, which could regulate PRAD via immune response, extracellular signaling, and protein formation. Moreover, 27 genes were identified as prognostic signatures and used to construct the risk score model. Receiver operating characteristic curves revealed the good performance of the risk score model in both the training and testing datasets. Different chemotherapeutic responses were observed between the low- and high-risk groups. Additionally, a nomogram based on the risk score and other clinical features was established to predict the 1-, 3-, and 5-year progression-free interval of patients with PRAD. This study provides novel insights into the molecular mechanisms of the immune microenvironment and its role in the pathogenesis of PARD. The identification of key cell subpopulations has a potential therapeutic and prognostic use in PRAD.

## 1. Introduction

PRAD is a common malignancy in males [1, 2], ranking first in incidence and second in mortality [3]. However, the aetiology of a disease as common as PRAD remains unclear [4]. Hormones regulate the growth and maintenance of the prostate gland, with androgens playing a key role in PRAD progression [1, 5]. Additionally, several dietary factors, such as soy protein, animal fat, and fibers, have been considered etiologic factors that are vital for the progression of PRAD [6].

Current PRAD examinations, including digital rectal diagnosis, serum prostate-specific antigen (PSA), ultrasound detection, computed tomography (CT), and magnetic resonance imaging (MRI), present certain limitations, especially in patients suffering from biochemical recurrence and re-evaluation after treatment [7]. However, the current diagnoses cannot reveal PRAD's heterogeneity [8], which is vital in predicting drug treatment sensitivity or primary resistance [9]. Therefore, exploring the pathogenesis of PRAD is highly significant as it can aid in identifying new targets for its diagnosis, thereby developing new drugs and improving patient prognosis.

It has been widely reported that tumorigenesis, tumor growth, and tumor metastasis are affected by the tumor immune microenvironment (TME), which includes immune cells, stromal cells, blood vessels, and the extracellular matrix [10]. Genetic alterations have been reported to alter the microenvironment in epithelial cells [11]. Furthermore, numerous studies report the complexity of TME, with the tumor-infiltrating immune cells (TIICs) playing a crucial role in TME [12]. TIICs in the TME are correlated with the prognosis and treatment response [10]. However, many modulatory interactions between the immune cells remain unknown because of TME complexity. Because of the heterogeneous and dynamic nature of cancer's microenvironment, the immune contexture of TME has been shown to affect the disease's outcome [13]. Hence, it is necessary to conduct an in-depth analysis of the PRAD TME at the single-cell level.

Single-cell sequencing is a technology that performs sequencing and analysis at the level of the genome, transcriptome, and epigenome in a single cell. Single-cell resolution is beneficial for analyzing intratumor heterogeneity [14]. Unlike bulk RNA-Seq data analysis, single-cell RNA-Seq (scRNA-seq) analysis allows for an in-depth profile of cell populations at the cellular level and aids in the discovery of rare subpopulation functions, thus eliminating the problem of deconvolution caused by bulk measurements. However, most of the present study uses bulk RNA-Seq data. RNA-Seq data from bulk samples assembles the variant allele frequency profiles of thousands to millions of cells, which cannot be obtained from scRNA-Seq data. The mechanism underlying the effects of immune cell infiltration on the prostate at the single-cell level remains unclear. Therefore, considering the heterogeneity of PRAD cells, this study uses bioinformatic analyzes, including extensive genomic and transcriptomic characterization with both bulk RNA-Seq and scRNA-Seq to elucidate the mechanisms. Firstly, bulk RNA-Seq data are used to find key genes associated with PRAD TME. Secondly, the single-cell sequencing data of PRAD is used to analyze the expression patterns of key genes in different cell subsets. Thus, the role and potential mechanisms of TME in PRAD could be explained at the single-cell level.

## 2. Materials and Methods

### 2.1. Data Source

For bulk RNA-seq analysis, the R package “TCGAbiolinks” was used to obtain level 3 mRNA expression data of PRAD from the cancer genome atlas (TCGA) database. Then, the data was further filtered by the TCGAanalyze_Preprocessing function using coefficient >0.6 among samples as the criterion, and the genes with an average expression level ranking in the top 75% were retained by the TCGAanalyze_Filtering function. After data processing, 498 PRAD and 52 adjacent control samples (normal tissue), namely the TCGA-PRAD cohort consisting of 13,125 genes, were used for downstream analysis. The normalized PRAD single-cell sequencing data, including 36,424 cells and 24,391 genes, which have been preprocessed in a previous study [15], were downloaded from the gene expression omnibus (GEO) GSE141445 dataset for downstream analysis. Additionally, the bulk RNA-seq data and survival information of patients with PRAD in GSE54460 (*N* = 106), GSE46602 (*N* = 36), GSE70768 (*N* = 111), and GSE70769 (*N* = 92) were used as testing sets. The pipeline of the entire process is shown in Figure S1.

### 2.2. Identification and Functional Analysis of Differentially Expressed Genes (DEGs) in TCGA-PRAD

DEGs in PRAD and adjacent control samples were identified using the “DESeq2” R package, with a threshold of |log2FC| > 1 and adjusted *p*-value < 0.05. R package “ClusterProfiler” was employed to perform the Kyoto Encyclopedia of Genes and Genomes (KEGG) and Gene Ontology (GO) enrichment analyzes of DEGs. A *q*-value <0.05 indicated significant enrichment.

### 2.3. Landscape of Immune Infiltration in TCGA-PRAD

Single-sample gene set enrichment analysis (ssGSEA) algorithm in the R package “GSVA” was utilized to calculate the infiltration levels of 27 immune cell types in PRAD and adjacent control samples [16]. The difference in immune cell infiltration between PRAD and adjacent control samples was determined using the Wilcoxon test [17]. Immune cells with a *p*-value < 0.05 were considered differentially infiltrated immune cells (DIICs).

### 2.4. Identification of DIIC-Related DEGs in TCGA-PRAD

Weighted correlation network analysis (WGCNA) was performed based on the gene expression profiles and DIICs. A sample clustering treemap was constructed to detect and eliminate outliers. Subsequently, WGCNA was performed based on the gene expression profiles and sample traits (differentially enriched immune cell types). The “pick Soft Threshold” function of WGCNA was used to calculate *β* and select the best soft threshold. Based on the selected soft threshold, the adjacency matrix was converted to a topological overlap matrix for constructing the network. Moreover, the gene dendrogram and module color were established using a dissimilarity degree. The initial module was further divided using dynamic tree cutting, and similar modules were merged. Pearson's correlation coefficient between the module eigengenes and DIICs was calculated to identify the most relevant module associated with DIICs. Using module membership (MM) > 0.8 and gene significance (GS) > 0.5 as criteria [18], DEGs in the most relevant module associated with DIICs were screened for further analysis.

### 2.5. Identification of Hub Differentially Infiltrated Immune Cell-Related Genes (DIICs-DEGs) in PRAD

DIICs-DEGs were input into the STRING database (https://www.string-db.org/) to construct the protein-protein interaction network (PPI). The top 10 genes with the highest interaction degrees were extracted and defined as candidate hub DIICs-DEGs. Pearson's correlations among the candidate hub DIICs-DEGs were calculated and visualized using the R package “ggcorrplot.” The function of candidate hub DIICs-DEGs was analyzed using the Metascape online tool (https://metascape.org/). The expression patterns of the candidate hub DIICs-DEGs in PRAD and adjacent control samples in TCGA-PRAD were tested in an external dataset, GSE54450. The candidate hub DIICs-DEGs with consistent expression patterns in the TCGA-PRAD and GSE54460 datasets were identified as differentially infiltrated immune cell-related genes (DIICRGs).

### 2.6. Single-Cell Analysis

The Seurat pipeline was used to analyze single-cell sequencing data and classify the cell groups. The cell types were identified using SingleR [19], and the cell differentiation trajectory was analyzed using Monocle [20].

### 2.7. Construction and Validation of the Risk Score Model

After converting the gene symbol of the selected DEGs in TCGA to ENTREZ ID, the intersection of DEGs and marker genes of cell clusters was analyzed using univariate Cox regression to screen genes related to the progression-free interval (PFI) (HR ≠ 1, *p*-value < 0.0001 for stricter screening) [21]. The LASSO algorithm was employed to further screen for prognostic signatures under the optimal lambda with the smallest classification error using the R package “glmnet.” After the proportional hazards (PH) assumption, prognostic signatures with a *p*-value >0.05 were used to construct the risk score (multivariate Cox regression) model in the training set. Then, the risk score was determined using the following formula: ∑_1_^*n*^ExpGene*i*^*∗*^Coefi (ExpGene*i*, expression of the gene; Coefi, coefficient of the gene). To evaluate the performance of the risk score model, the receiver operating characteristics (ROC) curve was plotted using the R package “survivalROC.” Moreover, the risk score model was tested in external datasets, including GSE54460, GSE46602, GSE70768, and GSE70769. According to the expressions and coefficients of model genes, the risk score of each patient was calculated, and the patients with PRAD were divided into low- and high-risk score groups based on the median value of the risk score. The PFIs in the low- and high-risk groups were analyzed and compared using the Kaplan–Meier curves and the log-rank test. The “pRRophetic” R package and Wilcoxon test were used to analyze and compare the chemotherapeutic response of patients in the low- and high-risk groups to 138 drugs [22].

### 2.8. Establishment of the Predictive Nomogram for PFI

The risk score and clinical features of patients with PRAD (age and TNM stage) were analyzed using univariate Cox regression to detect independent prognostic factors (*p*-value <0.05). The screened independent prognostic factors were then analyzed using multivariate Cox regression to construct the nomogram for predicting the 1-, 3-, and 5-year PFI of patients with PRAD. The nomogram's efficiency was evaluated using ROC curves.

## 3. Results

### 3.1. DEGs Identified in TCGA-PRAD Have Multiple Functions

**Figure 1:**
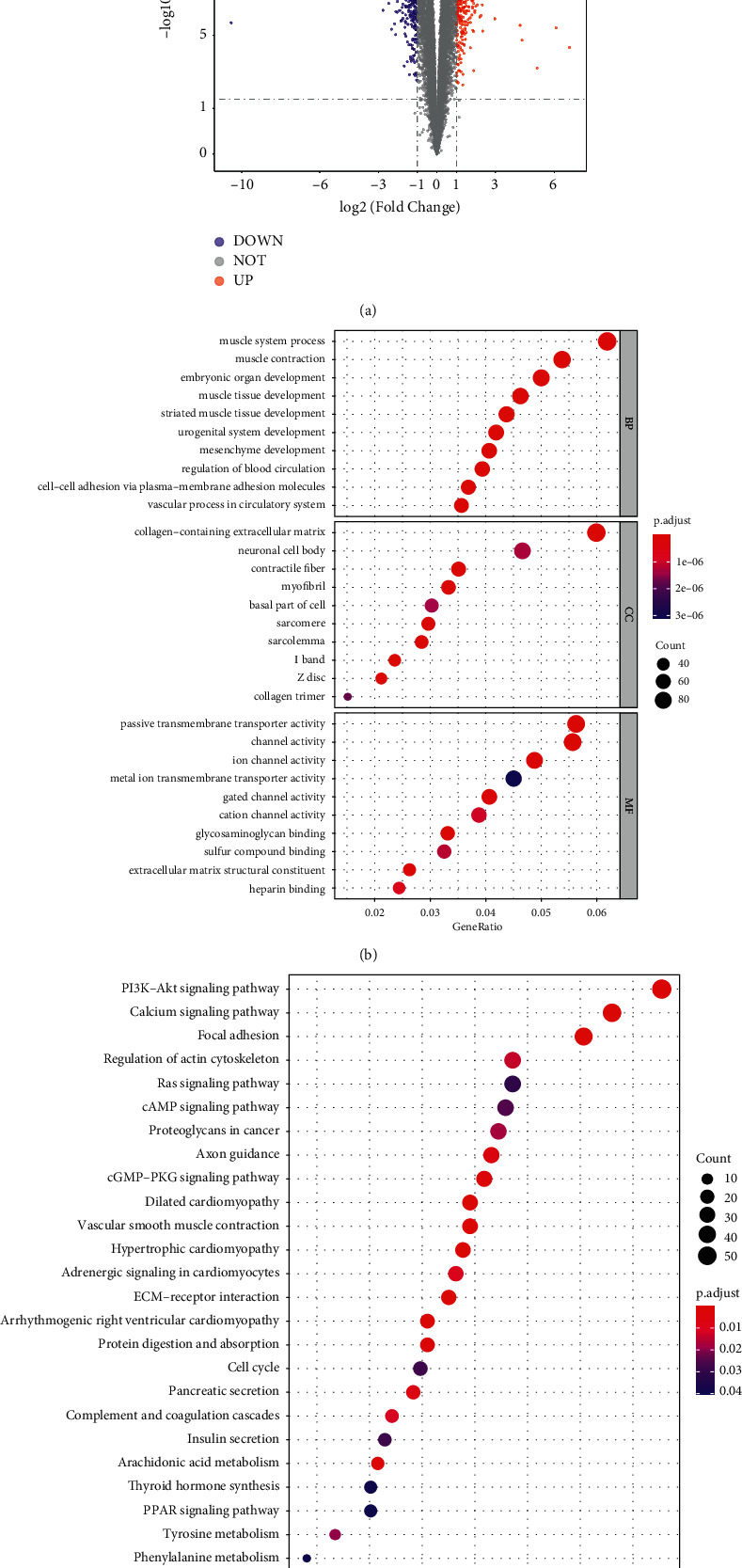
Multiple functions of DEGs have been identified in the cancer genome atlas prostate cancer (TCGA-PRAD) cohort. (a) DEGs between PRAD and control samples were identified using fold-change values and adjusted *p*-values. DEGs, differentially expressed genes; PRAD, prostate cancer; Control, normal tissue. Red and blue dots indicate upregulated and down-regulated mRNAs, respectively. Statistical significance was set at *p*-value <0.05. (b). DEGs enriched in the gene ontology (GO) pathways. BP, biological process; CC, cellular component; MF, molecular function. Adjusted *p* value <0.05 was considered statistically significant. (c) DEGs enriched in the Kyoto Encyclopedia of Genes and Genomes (KEGG) pathways. Adjusted *p* value <0.05 was considered statistically significant.

A total of 1,750 DEGs, including 714 upregulated (PRAD versus control) and 1,036 downregulated genes (PRAD versus control), were identified in the TCGA-PRAD cohort (Figure 1(a) and Table S1). The DEGs were significantly enriched in 473 biological processes, 65 cellular components, and 55 molecular functions (Table S2). Moreover, the top biological processes were mainly associated with the muscle system, urogenital system, circulatory system, and cell adhesion, such as muscle contraction, blood circulation regulation, and cell-cell adhesion via plasma-membrane adhesion molecules (Figure 1(b)). Additionally, 29 significantly enriched KEGG pathways were identified (Table S3). These pathways were further found to be relevant to cell proliferation, cell adhesion, cardiomyopathy, and immunity, such as focal adhesion, extracellular matrix (ECM)-receptor interaction, cell cycle, complement and coagulation cascades, hypertrophic cardiomyopathy, pI3K-Akt signaling pathway, and calcium signaling pathway (Figure 1(c)). Therefore, the complexity of PRAD aetiology could be attributed to multiple biological processes and signaling pathways that contribute to the development of PRAD.

### 3.2. TME Is Altered in PRAD

**Figure 2:**
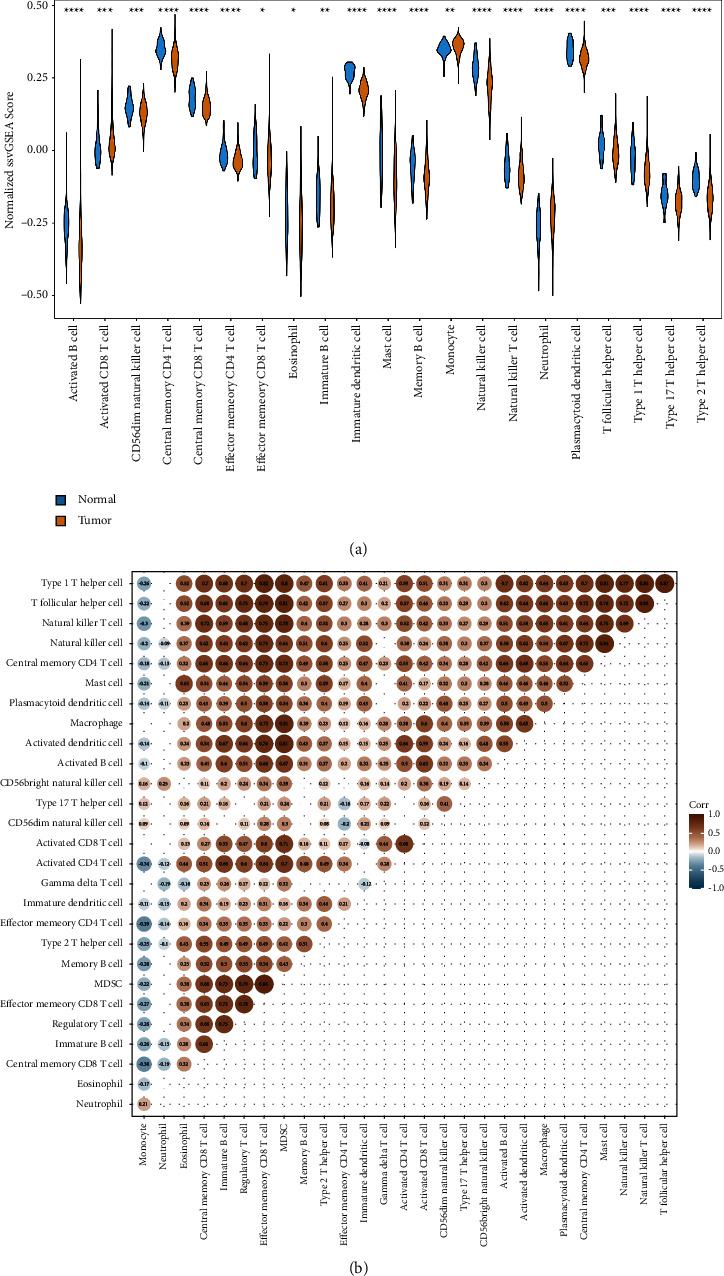
The altered immune microenvironment in prostate cancer (PRAD). (a) A total of 21 types of tumour-infiltrating immune cells were observed in PRAD compared to normal tissues in The cancer genome atlas cohort. Blue and yellow indicate normal and tumour tissues, respectively. (b) Matrix demonstrating the correlation between 28 immune cells ns, *p* > 0.05; ^*∗*^*p* < 0.05; ^*∗∗*^*p* ≤ 0.01; ^*∗∗∗*^*p* ≤ 0.001; ^*∗∗∗∗*^*p* ≤ 0.0001.

A growing body of evidence reveals the important role of the TME in cancer [10, 11, 23]. Therefore, the infiltration levels in PRAD and control samples were analyzed using ssGSEA. A total of 21 DIICs (immature B cells, memory B cells, activated B cells, central memory CD8^+^ T cells, central memory CD4^+^ T cells, activated CD8^+^ T cells, NK cells, effector memory CD8^+^ T cells, type 2T helper cells, effector memory CD4^+^ T cells, CD56dim, NK cells, type 17T helper cells, monocytes, NK T cells, immature dendritic cells, plasmacytoid dendritic cells, mast cells, T follicular helper cells, eosinophils, neutrophils, and type 1T helper cells), most of which showed higher infiltration levels in control samples than PRAD samples, were identified (Tables S4-S5 and Figure 2(a)). Further analysis showed that these DIICs had moderate to strong correlations with each other (Figure 2(b)).

### 3.3. Nine Hub DIICRGs Identified in PRAD

**Figure 3:**
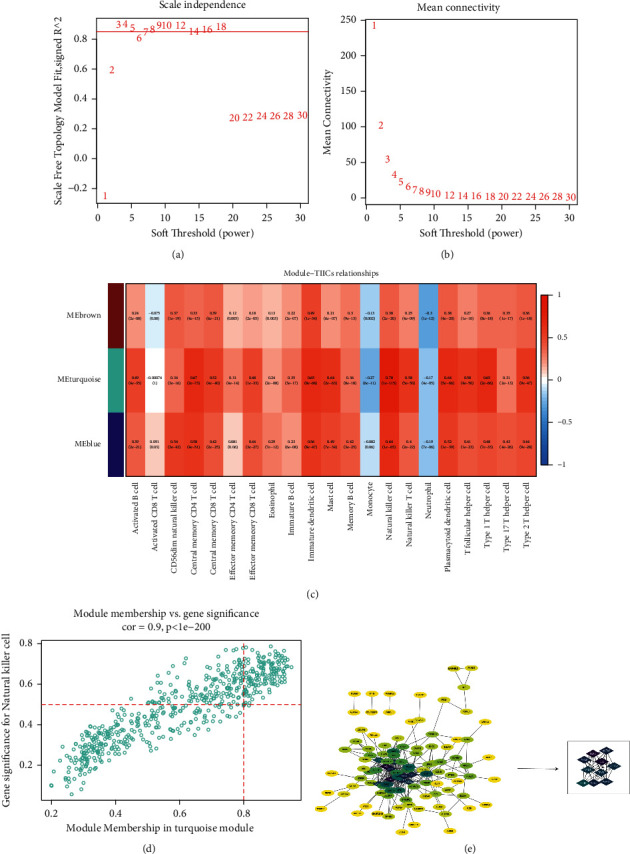
The selection of hub genes. (a) The nature of the network topology is constructed using the unique soft threshold (Power). (b) The relationship between Soft Threshold (Power) values and mean connectivity. (c) The correlation between the different modules of ME brown, ME turquoise, ME blue, and tumour-infiltrating immune cells. (d) Scatter plot showing the correlation between genes (MM) and natural killer cells (GS) in the turquoise module. The genes meeting the criteria of GS > 0.5 and MM > 0.8 were selected for further analysis. (e) Protein-protein interaction (PPI) networks in 144 genes and the selection of 10 hub genes. The circle indicates genes in the turquoise module while the edges indicate the interactions between genes. The PPI network was constructed using STRING and visualised using the R package “ggcorrplot.”

To explore genes associated with DIICs, WGCNA analysis was performed. After sample clustering, outlier samples were excluded (data not shown). Using the “pick Soft Threshold” function of WGCNA, the optimal soft threshold power was found to be 3, wherein *R*^2^ was approximately 0.9 (Figure 3(a)). Three modules were identified from the coexpression network. According to the module-trait relationships shown in Figure 3(b), the turquoise module was selected as the most relevant module with NK cells (cor = 0.78, *p*-value <0.05). Moreover, using GS > 0.5 and MM > 0.8, 144 genes were obtained from the turquoise module for further analysis (Figure 3(c)). The PPI network of the genes was constructed and visualized using Cytoscape (Figure 3(d)). Furthermore, the top ten genes with the highest interaction degrees, including, FLNA, VCL, ACTA2, MYL9, MYLK, MYH11, TPM1, ACTG2, TAGLN, and FLNC, were selected using the “cytohubba” plug-in (Figure 3(d)) and identified as candidate hub genes in PRAD.

**Figure 4:**
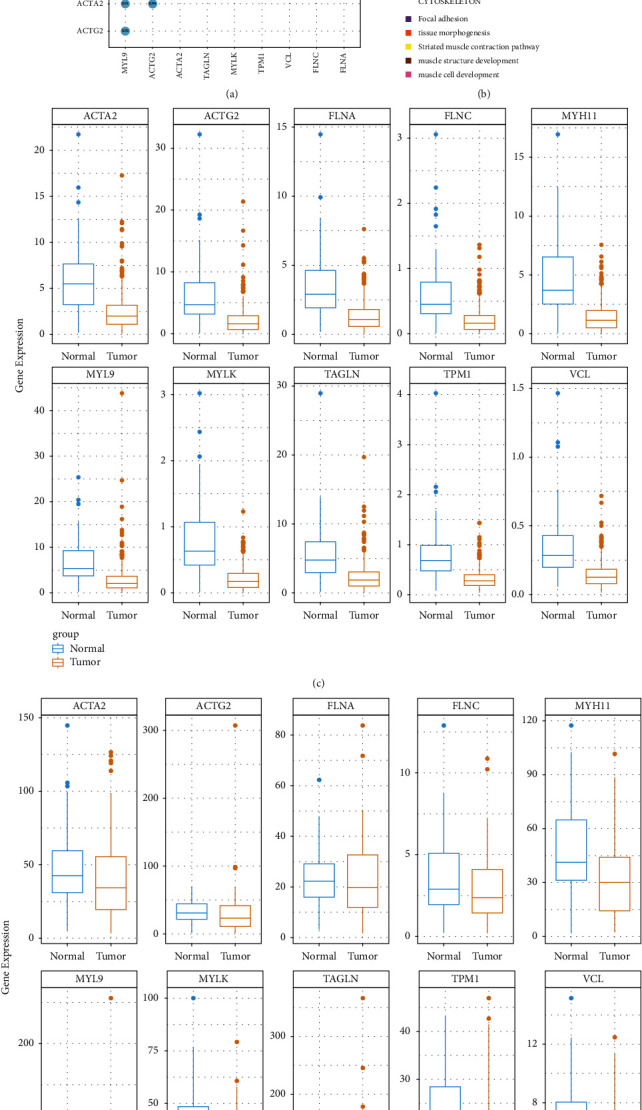
Validation of the hub gene expressions in the GSE54460 dataset. (a) Correlation analysis of candidate hub genes. (b) Gene ontology (GO) and Kyoto encyclopedia of genes and genomes (KEGG) enrichment of candidate hub genes using Matascape. (c) Expression of candidate hub gene in the cancer genome atlas cohort C and GSE54460 dataset (d) Blue, Normal tissue; Yellow, Tumour tissue.

Correlation analysis revealed that candidate hub genes had a moderate to a strong relationship with each other (Figure 4(a)). Metascape revealed that they were associated with the contraction of the smooth muscle, RHO GTpase activation of PAKs, regulation of the actin cytoskeleton, focal adhesion, tissue morphogenesis, striated muscle contraction pathway, and development of muscle structure and muscle cell (Figure 4(b)). Thereafter, the expression patterns of the genes in the TCGA-PRAD and GSE54460 datasets were examined, revealing that the expressions of VCL, ACTA2, MYL9, MYLK, MYH11, TPM1, ACTG2, TAGLN, and FLNC were significantly different between PRAD and control samples in both cohorts (Figures 4(c)–4(d)). Thus, these nine genes were identified as hub DIICRGs in PRAD.

### 3.4. Hub DIICRGs Are Mainly Expressed in Three Cell Subpopulations

**Figure 5:**
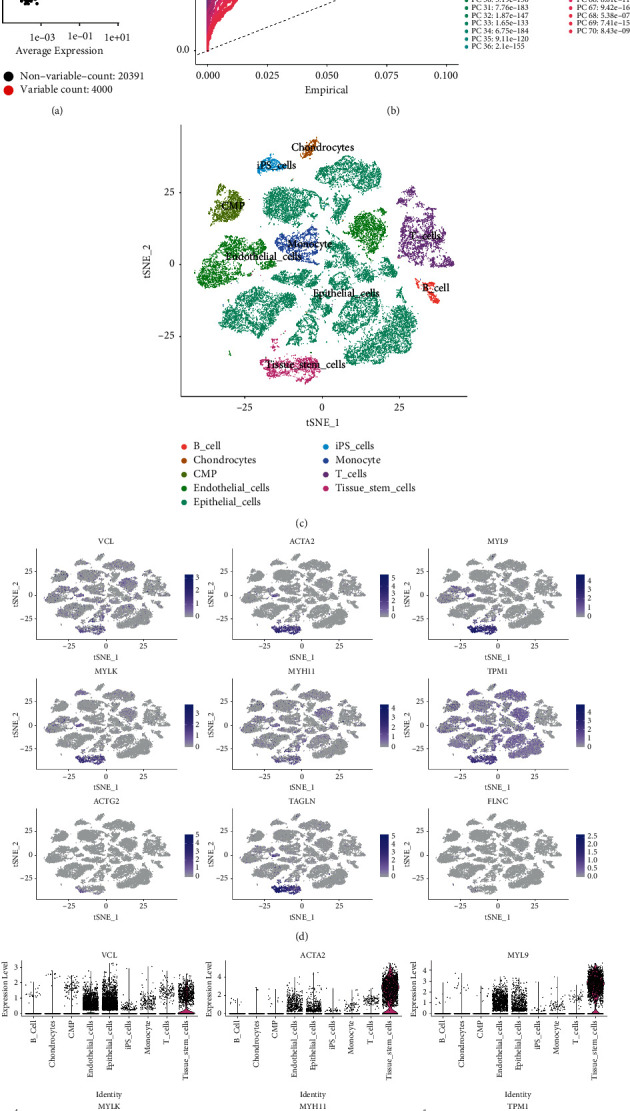
Expression profiles of hub differentially infiltrated immune cell-related genes (DIICRGs) in cell subpopulations. (a) The characteristic variance diagram displays the genes with significant differences across cells. (b) Line chart displaying the significantly available dimensions of data sets with *p* value <0.05, which were identified using principal component analysis. PC, principal component. (c) The annotated cell subpopulations using SingleR. (d) Mapping of hub gene expressions in the epithelial, tissue stem and endothelial cells via the tSNE method. (e) Hub gene expressions in the epithelial, tissue stem and endothelial cells.

The top 4000 highly variable genes (Figure 5(a)) were selected for downstream analysis. After performing the principal component analysis (PCA) on highly variable genes, 70 significant principal components (PCs) were identified (Figure 5(b)). Using the tSNE method, PRAD cells were clustered into nine distinct cell subpopulations, including B cells, chondrocytes, common myeloid progenitor, endothelial and epithelial cells, induced pluripotent stem cells, monocytes, T cells, and tissue stem cells (Figure 5(c)). The examination of the expression patterns of hub genes in those cell subpopulations revealed that they were highly expressed in endothelial, epithelial, and tissue stem cells (Figures 5(d)–5(e)), suggesting that these cell subpopulations could be crucial to PRAD aetiology.

### 3.5. The Pseudotime Trajectory Reveals the Important Role of Epithelial Cells in PRAD

**Figure 6:**
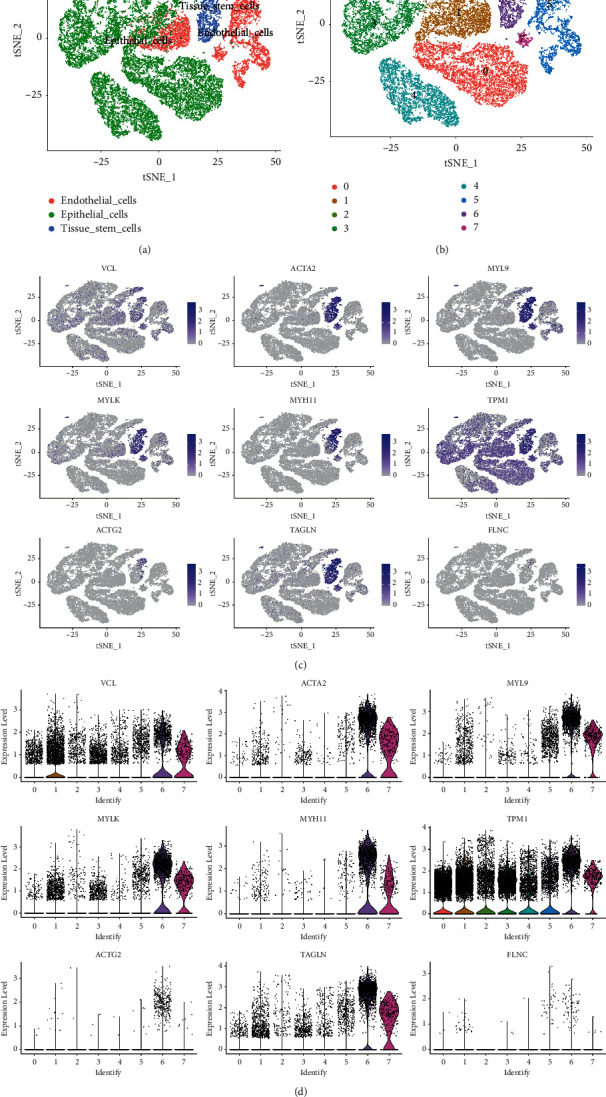
Subclustering of the cell subpopulations for endothelial, epithelial and tissue stem cells. (a) Distribution of the cell subpopulations of endothelial, epithelial and tissue stem cells. (b) Eight clusters in the cell subpopulations of endothelial, epithelial and tissue stem cells. (c) Expression of hub genes in the subclusters (tSNE mapping). (d) Violin plots revealing the expression of hub genes in the eight subclusters.

To further explore the heterogeneity of endothelial, epithelial, and tissue stem cells, the cells were subclustered into eight clusters, with cluster 5, cluster 7, and partial cluster 1 belonging to endothelial cells. Cluster 6 belongs to the tissue stem cells, and clusters 0, 2, 3, 4, and partial cluster 1 belong to the epithelial cells (Figures 6(a) and 6(b)). Moreover, VCL and TPM1 were abundant in all clusters, while the other hub genes were mainly expressed in clusters 6 and 7 (Figures 6(c)–6(d)).

**Figure 7:**
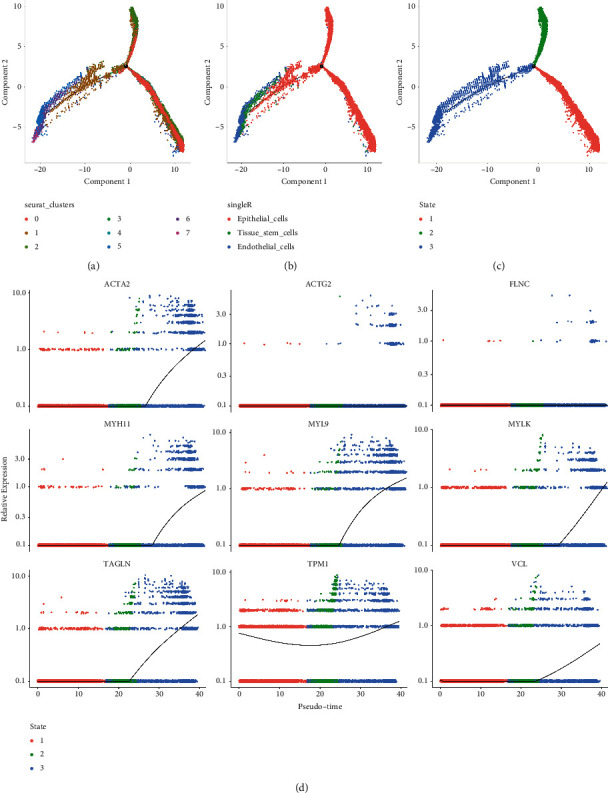
Trajectory analysis deciphering the origin of prostate cancer (PRAD). (a) The distribution of the eight sub-clusters in the trajectory. (b) Cell annotations in trajectory analysis. (c) The state of the trajectory analysis. (d) Nine hub gene expressions across trajectory time.

Trajectory analysis showed that endothelial cells (cluster 5, cluster 7, partial cells in cluster 1) and tissue stem cells (cluster 6) mainly originated from the epithelial cells (cluster 0 and cluster 2) (Figures 7(a)–7(c)), indicating the critical role of epithelial cells in PRAD progression. The dynamic changes of hub gene expression were further analyzed along the trajectory, which revealed that the expressions of ACTA2, MYH11, MYL9, MYLK, TAGLN, and VCL significantly increased in the late stage of cell differentiation (Figure 7(d)). This increased expression could be closely associated with the occurrence of PRAD.

**Figure 8:**
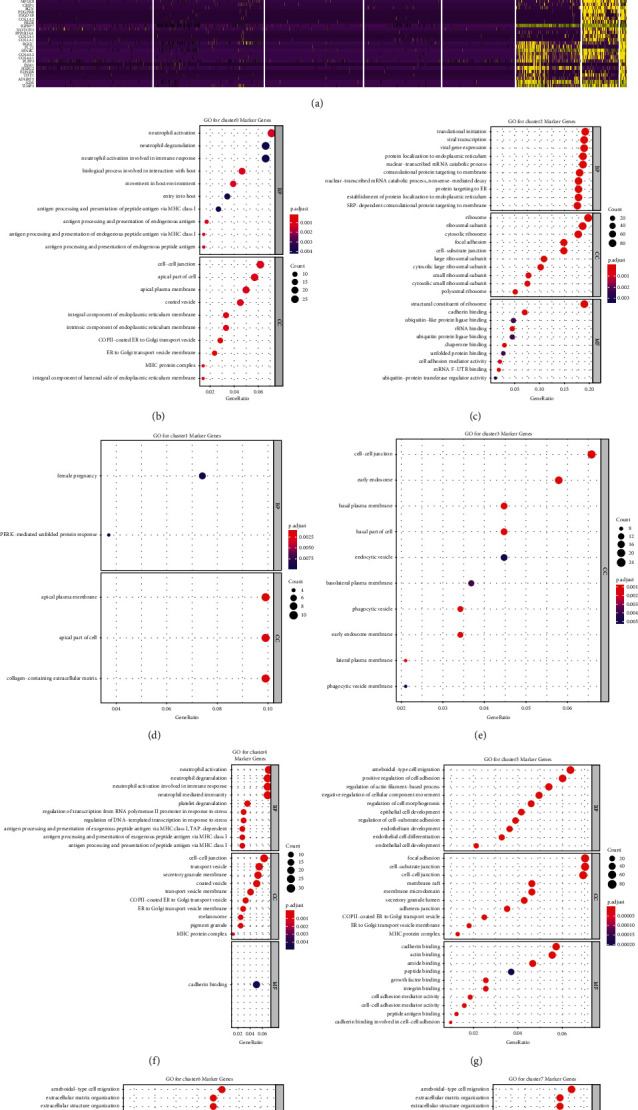
Function analysis of each cluster in prostate cancer (PRAD). (a) Heat map demonstrating the top 20% significant marker genes with the criteria of |log_2_FoldChange|> 0.5 and *p* value <0.05. Gene ontology enrichment analysis with adjusted *p*-values for (b) cluster 0, (c) cluster 1, (d) cluster 2, (e) cluster 3, (f) cluster 4, (g) cluster 5, (h) cluster 6, and (i) cluster 7 marker genes.

To illustrate each cluster's role, marker genes in each cluster were identified (Figure 8(a)), and the functional analysis of the markers in each cluster was performed (all enrichment results can be found in Tables S6-S13 for cluster 0–7). The common and distinct functions among the clusters could contribute to the complexity of PRAD. The markers of clusters 0 and 4 were mainly enriched in immune-related biological processes, such as neutrophil activation (Figures 8(b) and 8(f)). Markers in clusters 6 and 7 were mainly associated with ECM processes, such as ECM organization and cell-substrate adhesion (Figures 8(h)-8(i). Furthermore, the functions of the markers in cluster 5 were similar to those in clusters 6 and 7, which were also relevant to endothelial cell differentiation (Figure 8(g)). Notably, markers in clusters 1, 2, and 3 had important roles in the formation of proteins, such as PERK-mediated unfolded protein response, translation initiation, and plasma-membrane protein development (Figures 8(c)–8(e)).

### 3.6. Construction of a Risk Score Model Based on 25 Gene Signatures in PRAD

**Figure 9:**
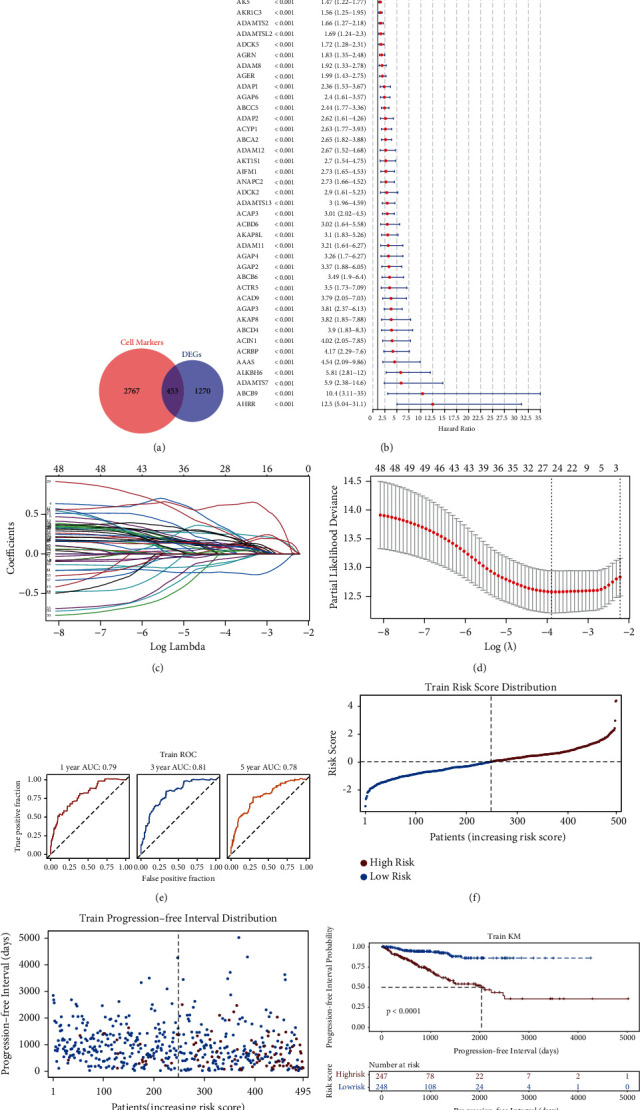
Construction of a prognosis signature of 25 genes in prostate cancer (PRAD). (a) A total of 453 genes overlapped the marker genes, which were obtained from Figure 8(a), and the differentially expressed genes (DEGs) using the venn diagram. (b) Univariate Cox regression analysis revealed 53 genes significantly linked to the progression-free interval (PFI) at a *p* -value <0.05. (c) The coefficients of the 53 genes are shown using the lambda parameter. (d) The optimal genes are determined using LASSO regression analysis to construct the risk model. (e) Receiver operating characteristic (ROC) curve analysis for 1-, 3- and 5-year PFI using the TCGA training set. (f) Patients with PRAD in the TCGA cohort are listed in ascending order of risk score. (g) PFI distribution versus the risk score of each patient in the TCGA-PRAD cohort. (h) Kaplan–Meier curves of patients having different risk levels in the internal TCGA training set.

To explore the role of marker genes in the prognosis of PRAD, the gene symbols of DEGs were converted into ENTREZ ID, with 1,723 DEGs (Table S14) overlapping with 3,220 marker genes and revealing 453 differentially expressed marker genes in PRAD (Figure 9(a) and Table S15). The univariate Cox regression analysis revealed 53 differentially expressed marker genes that were linked with the PFI in patients with PRAD significantly (Figure 9(b)). To obtain a more robust prognostic signature, the LASSO regression analysis was performed, and 27 genes, including ABCA2, ABCB6, ABCB9, ABCC5, ACAP3, ACIN1, ACOX2, ACRBP, ACYP1, ADAM11, ADAMTS2, ADAMTS7, ADAP1, ADAP2, ADCK2, ADH5, ADRA1D, AGAP2, AGAP3, AGRN, AHRR, AIFM1, AK5, AKAP7, AKR1C3, ALB, and ALDH1A2, were identified at a lambda min of 0.02042135 at 10-fold cross-validation (Figures 9(c) and 9(d)). After PH assumption, 25 prognostic signatures (ABCA2, ABCB6, ABCB9, ABCC5, ACAP3, ACIN1, ACOX2, ACRBP, ACYP1, ADAM11, ADAMTS2, ADAP1, ADAP2, ADCK2, ADH5, ADRA1D, AGAP2, AGAP3, AGRN, AHRR, AIFM1, AK5, AKAP7, AKR1C3, and ALDH1A2) were selected for the construction of the risk score mode. The areas under the ROC curves of 1-, 3-, and 5-year PFI were 0.79, 0.81, and 0.78, respectively, indicating the good performance of the risk score model (Figure 9(e)). Patients with PRAD in the TCGA training set were divided into low- and high-risk groups by the median risk score (Figure 9(f)). PFI was observed to reduce along with an increasing risk score (Figure 9(g)). Moreover, patients in the low-risk group had significantly longer PFI than those in the high-risk group (Figure 9(h)).

Moreover, similar results were observed in four external datasets (Figures S1–S4), demonstrating the reliability of the risk score model. Additionally, the chemotherapeutic responses of patients to 85 drugs were significantly different between the low- and high-risk groups (Figures S5 and S6), indicating that the prognostic biomarkers may affect the chemotherapy response of patients with PRAD. Among them, the half-maximal inhibitory concentration (IC_50_) of Bicalutamide, which is used in the treatment of advanced PRAD, in the high-risk group was significantly lower than that in the low-risk group. Cisplatin, another chemotherapy drug, presented significantly lower IC_50_ values in the low-risk group, suggesting its efficacy in treating localized PRAD or early-stage PRAD.

### 3.7. A Predictive Nomogram Is Established in PRAD

**Figure 10:**
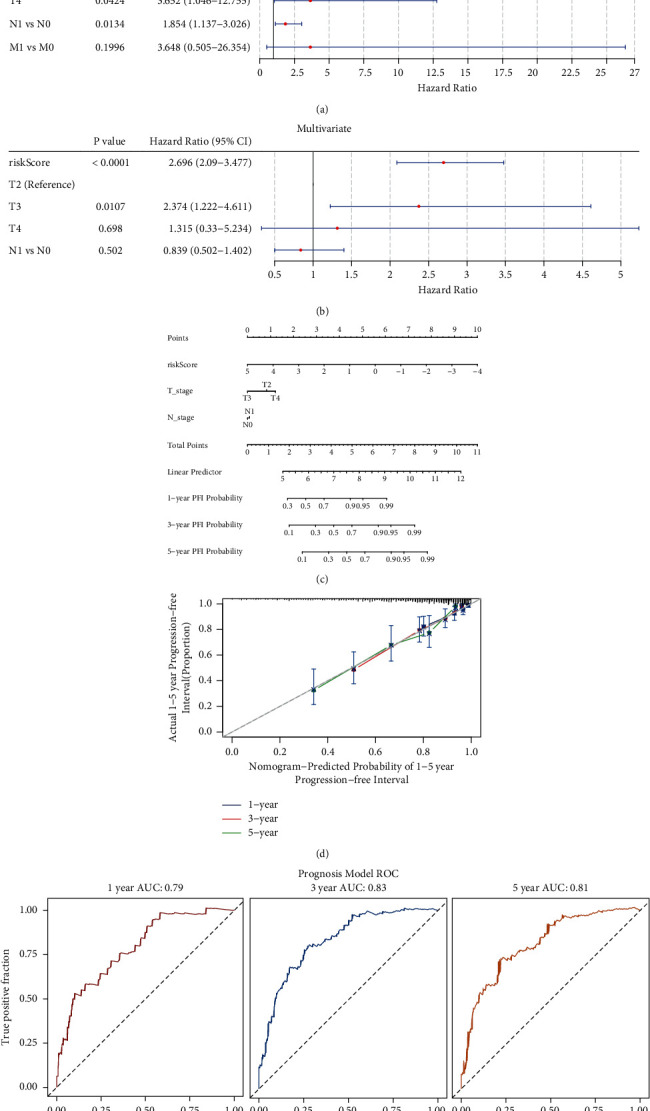
Establishment of the predictive nomogram. Independent prognostic factor analyzes using (a) univariate and (b) multivariate cox regression. (c) Nomogram based on risk score and T&N stage for the prediction of the 1-, 3- and 5-year progression-free interval (PFI). (d) The calibration curves of the nomograms for the 1-, 3- and 5-year PFI. (e) Receiver operating characteristics curve analysis for the 1-, 3- and 5-year PFI.

Finally, the risk score as an independent prognostic factor in PRAD was investigated. Using the univariate Cox regression analysis, the risk score, T stage, and N stage were found to be significantly related to the survival of patients with PRAD (Figure 10(a)). After the multivariate Cox regression analysis, the risk score remained significantly associated with the survival of patients with PRAD (Figure 10(b)), indicating that the risk score was an independent prognostic factor in PRAD. Thus, a nomogram based on the risk score and other clinical features was established to predict the 1-, 3-, and 5-year PFI of patients with PRAD (Figure 10(c)). The calibration curves showed that the predicted PFI was very close to the actual PFI (Figure 10(d)), suggesting the clinical application of the nomogram. Furthermore, the areas under the ROC curves were 0.79, 0.83, and 0.81 for the 1-, 3-, and 5-year PFI, respectively (Figure 10(e)), further indicating the good performance of the nomogram.

## 4. Discussion

A total of 1,750 DEGs were identified in the TCGA-PRAD cohort. ssGSEA showed that the proportions of 21 immune cells were significantly different between PRAD and control samples. WGCNA identified 144NK cell-related genes and nine hub genes, including VCL, ACTA2, MYL9, MYLK, MYH11, TPM1, ACTG2, TAGLN, and FLNC, which were selected by the PPI network. Using Single R, nine cell subpopulations were detected in PRAD. Based on the expression patterns of hub genes, endothelial, epithelial, and tissue stem cells were identified as key cell subpopulations, which regulate PRAD via immune response, extracellular signaling, and protein formation.

The functional analysis of DEGs in cancer and peritumoral samples was performed, revealing that the muscle tissue development and the blood circulatory system were mainly related to the DEGs. It has been reported that angiogenesis is closely related to the progression of cancer cells [24–26]. The increased local angiogenesis could be a marker for detecting PRAD [27]. Some drugs include Zoledronic acid [28], curcumin [29], and the EZH2 inhibitor GSK126 or EPZ6438 [26], which inhibit neoangiogenesis in patients with PRAD. To date, to the best of our knowledge, there is no direct evidence that muscle tissue development is associated with cancer progression. Hypoxia occurring within the muscle during exercise could stimulate angiogenesis [30–32]. Moreover, hypoxia is common in many cancers [33]. Therefore, it is inferred that a high oxygen demand during cancer cell growth could lead to hypoxia, wherein hypoxia simulates the muscle near the prostate to generate a stimulus for angiogenesis, thereby leading to cancer progression.

Subsequently, the immune microenvironment between the tumor and peritumoral samples was compared, revealing that the TME of PRAD was significantly altered, which was reflected by the significant change in infiltration levels in all 21 types of immune cells. Similarly, consistent with the results of Gao et al. [34], the current study showed a similar trend in terms of the infiltration level of T follicular helper cells. An increase in neutrophils was also reported [10, 35]. These increased immune cells are speculated to be involved in the progression of PRAD [35, 36]. Among these immune cells, NK cells, which were reduced in the tumor tissues, were found to be heavily involved in the anticancer activity as a type of lymphocyte population [37, 38]. Several studies indicate that a lower degree of NK cells is associated with an increased risk of PRAD [38–40]. The reduction of NK cell levels (Figure 2(a)) in PRAD, as reported by Li et al. [41], could be a favorable environment for neutrophils to promote metastasis. Conversely, a high level of NK cells indicates a good prognosis or lower risk of PRAD [42, 43]. Thus, NK cells could be used as a potential therapeutic target. Although immunotherapy has been used to treat various solid tumors, Sipuleucel-T is the only approved PRAD immunotherapy for castration-resistant PRAD, which is the advanced stage of PRAD [44, 45]. It is suggested that effective NK activation and tumor targeting/binding are essential mechanisms in NK cell-mediated cancer treatment. Highly effective NK cells are critical limiting factors in cancer immunotherapy efficacy [46, 47]. Hence, based on the current study, the increasing activity of the NK cells via checkpoint inhibitors, NK cell engagers, and cytokines could improve PRAD immunotherapy efficacy at different stages.

Next, nine hub genes, namely VCL, ACTA2, MYL9, MYLK, MYH11, TPM1, ACTG2, TAGLN, and FLNC, were obtained using WGCNA, PPI, and external gene set validation. VCL is a focal adhesion-related cytoskeletal protein that plays an essential role in cell adhesion and signal transduction. These nine hub genes mainly are involved in the processes, including metastasis, progression, and survival. MYL9 encodes an actin-binding protein involved in cell motility, division, and adhesion. Previous studies have confirmed that the expression level of MYL9 is downregulated in the stroma of PRAD, indicating the poor prognosis of patients with PRAD [48, 49]. MYL9 has the potential to become a molecular marker for diagnosing PRAD and predicting cancer progression and prognosis. TPM1 belongs to the tropomyosin family and plays a vital role in cytoskeletal functions, such as cell proliferation, migration, and apoptosis, thereby playing a key role in tumor growth and metastasis. MYH11 is a member of the Myosin family, which regulates functions, such as signal transduction, muscle contraction, and cell movement in the body, and the mutation of MYH11 has also been observed in prostate cancer, however, further exploration is needed [50, 51]. Myosin light-chain kinase (MYLK) is a member of the immunoglobulin superfamily, an enzyme independent of calcium-/calmodulin that facilitates myosin interaction with actin filaments and produces contractile activity. ACTA2 are found in muscle tissues and are the significant constituents of the contractile apparatus. ACTG2 gene encodes actin, a gamma-enteric smooth muscle protein found in human enteric tissues. The PPI network (Figure 3(d)) indicates the interactions among the hub genes (MYH11, MYLK, ACTA2, and ACTG2). Therefore, PRAD development was speculated to be correlated with muscle development (Figure 1(b)). Moreover, TAGLN and FLNC genes have functions in muscle tissues, indicating their involvement in PRAD progression.

Subsequently, using PRAD single-cell sequencing data, three cell subsets that could play an essential role in the occurrence and development of PRAD were screened. The tumorigenicity of epithelial cells was reported to be the major reason for PRAD development [52]. Epithelial-mesenchymal transition (EMT) is a normal cellular physiological process that involves transforming epithelial cells into cells with a mesenchymal phenotype. EMT is a necessary process that drives the metastasis of PRAD [53, 54]. Various proteins regulate e-cadherin expression, including snail [55], epidermal growth factor, epidermal growth factor receptor [56], twist [57], and miRNAs [58, 59]. The decreased expression, including the silencing of e-cadherin in the epithelial cells, leads to the sustained loss of normal polarity and adhesion [60] and subsequently to PRAD cell invasion and metastasis. In other words, the abnormal expression of genes in epithelial cells could promote PRAD development. However, further in-depth research is required to clarify the mechanisms and provide new targets and directions for the diagnosis, prognosis, and treatment of PRAD. Endothelial cells are strongly linked to metastasis. Endothelial cells surrounding tumors are the basis of angiogenesis [61]. Additionally, they boost autophagy and accelerate focal adhesion protein disassembly [62]. This type of cell is also associated with drug resistance in PRAD. Recent studies have shown that vascular endothelial cells can modulate the response of tumor cells to chemotherapy [63]. Akiyama et al. [64] found that endothelial cells can acquire drug resistance from TME.

Tissue stem cells are the dominant cell types in PRAD, suggesting that PRAD could be a stem cell disease. In addition to the stem cell characteristics of proliferation, self-renewal, and differentiation potential [65], cancer stem cells (CSCs) have the ability to generate the heterogeneous lineages of cancer cells that develop into tumors [66, 67]. Only a tiny portion of PRAD exhibit the phenotypical and functional characteristics of normal prostate stem cells and participate in tumorigenesis, metastasis, and drug resistance [68–70]. However, PRAD CSC markers remain undefined in clinical practice. There is increasing evidence that PRAD–CSC–specific markers (CD44, CD133, CD166, FAM65 B, MFI2, and LEF1) can predict the patients' overall survival, which suggests the clinical potential of PRAD CSC as biomarkers and therapeutic targets [71, 72]. The combined use of CSC vaccines with immunomodulators, such as anti-PD-L1 antibodies, can significantly improve the anti-tumor efficiency of CSC-based vaccines and block the immunosuppressive effect of the TME [73, 74]. Thus, immunotherapy targeting CSC could have clinical implications in treating PRAD.

To further reveal the function of the three cell subsets in PRAD, they were subdivided into eight cell clusters, for which cell trajectory and functional analyses were performed. The pseudotemporal cell trajectory analysis indicated that the epithelial cells were at the beginning of the entire cell tree. It has been suggested that endothelial cells are a type of epithelial cells, indicating that epithelial cells could transform into endothelial cells [75]. Chen et al. [15] claimed that this differentiation occurs in carcinoma cells that have undergone EMT, which contributes to tumor growth. Additionally, following the treatment of proangiogenic signals, which is a common phenomenon during tumors, epithelial cells can transition to endothelial cells *in vivo* [76]. There is little robust evidence that epithelial cells could be differentiated into stem cells in PRAD. However, it is assumed that epithelial cells reacquiring self-renewal capabilities could lead to carcinogenesis [77, 78]. Following EMT, tumor cells could gain stem cell-associated properties [79–82]. Therefore, similar signals *in vivo* could be applied to epithelial cells to validate the transition to stem cells through EMT.

These enrichment results match the cell subpopulations' functions (Figures 6(a) and 6(b)), indicating that cell clusters from the same source have similar functions but cell clusters from different sources have dissimilar functions. This ability to differentiate into different types of cells contributes to the heterogeneity of PRAD. The pseudotime trajectory and pathway enrichment (functional analysis) analysis might provide speculations regarding the occurrence of PRAD. In the beginning, epithelial cells (clusters 0 and 4) were the main subpopulations of the cells, and functional analysis deciphers the activation of neutrophils as the primary function. Following activation, neutrophils, inflammation, and cancer can elevate DNA replication errors and release reactive oxygen species in epithelial cells [23]. Thus, activated neutrophils trigger the oncogenic transformation, and epithelial cells are subsequently driven to carcinogenesis and the abnormal expression of specific genes. Following this, clusters 1, 2, and 3, which were considered the main types, including epithelial and endothelial cells, were found to be involved in the pathways of PERK-mediated unfolded protein response (UPR) and translation initiation, which correlates with previous studies. PERK-mediated pathways introduce endoplasmic reticulum stress into cells and reduce the expression of e-cadherin [83, 84]. Thus, epithelial cells could be differentiated into endothelial cells. UPR, to some extent, aids in the survival of tumor cells [83, 85]. Furthermore, PERK-mediated processes assist the production of proangiogenic factors and the growth of blood vessels, offering tumor cells a route to metastasis [83, 86]. Proteins that are relevant to angiogenesis are then produced by endothelial cells [87, 88]. During angiogenesis, endothelial cells grow and promote the formation of new blood vessels through the vascular endothelial growth factor (VEGF). UPR also reduces cell-cell junction markers, promoting metastasis [89, 90]. However, epithelial cells could also transition to CSCs or stem cells. Stem cells, including CSCs, induce the expression of endothelial markers [91, 92], and VEGF also promotes endothelial cell differentiation [93]. Throughout angiogenesis and EMT, ECM organization is dysregulated and cell-substrate adhesion is reduced [94], resulting in cancer cells acquiring an invasive and migratory phenotype.

To further explore the prognostic value of cell subset marker genes, 25 prognostic genes were identified through univariate, LASSO, and multivariate analyses. Their roles in PRAD are summarized in Table 1. Based on these 25 genes, a risk model was constructed, which divided patients into high- and low-risk groups. Interestingly, a significant difference was observed in chemosensitivity between the high- and low-risk groups. Bicalutamide was found to be more effective for patients with PRAD in the high-risk group than in the low-risk group. Clinically, Bicalutamide is used to treat advanced PRAD [121, 122], corroborating our results. Cisplatin is used to treat early-stage PRAD [123], which corroborates with the result that the patients in the low-risk group are more sensitive to Cisplatin than the high-risk group. Moreover, a significant difference was observed in the chemotherapy response to the other 83 drugs, although they are not yet approved for the treatment of PRAD. Despite the advances in PRAD treatment, therapeutic options for PRAD remain limited [124, 125]. These 83 drugs can be considered a pool when looking for novel therapies. For instance, GDC.0449 is a drug targeting the Hedgehog pathway, whose activation could indicate the potential effectiveness of GDC.0449 in PRAD [8, 126]. Moreover, the SRC family kinase activity is observed in hormone-refractory PRAD [127], suggesting that medications targeting the SRC family, such as A.770041, AZD.0530, and WH.4.023, could be effective in PRAD treatment.

The small sample size (13 tumors) limits the applicability of the study [15]. This study combined the analysis of high-throughput gene chip data and single-cell sequencing data, which avoided the insufficiency of a single research method and improved the credibility of the results. The occurrence and development of PRAD, a highly heterogeneous disease, involves complex mechanisms, and the DEGs obtained form only a small part of the mechanism. Furthermore, novel insights into the molecular mechanisms of TME in the pathogenesis of PARD are reported. Hence, precision medicine is crucial in PRAD treatment because of the disease's heterogenicity. Further immunological functional studies to clarify the biological functions of these genes and cell subsets in PRAD are essential, which could provide a solid foundation for improved clinical diagnosis and treatment.

## Figures and Tables

**Table 1:** Roles of the 25 genes used in the prognosis signature.

Gene	Role	References
ABCA2	Drug resistance and malignant progression	Zhu et al. [95]; pasello et al. [96]
ABCB6	Progression	Karatas et al. [97]
ABCB9	Drug resistance	Guazzelli et al. [98]
ACAP3	Prosurvival role	Sullivan et al. [99]
ACIN1	Belongs to the canonical apoptosis signaling pathway	Ali et al. [100]
ACOX2	Tumour progression	Bjørklund et al. [101]
ACRBP	Paclitaxel resistance	Whitehurst et al. [102]
ACYP1	Tumorigenesis and progression; poor prognosis	Zhou et al. [103]
ADAM11	Tumour suppressor	Ribeiro et al. [104]
ADAMTS2	The inhibition of proliferation and angiogenesis of endothelial cells	Kirana et al. [105]
ADAP1	Metastasis and invasion	Oga et al. [106]
ADAP2	a key accessory protein for relaying signals via natural killer cell receptors	Campbell and colonna [107]
ABCC5	Accelerating tumour growth and migration	Ji et al. [108]
ADCK2	The proliferation and survival of cancer cells; regulation of superoxide activity	Vierthaler et al. [109]; schoolmeesters et al. [110]
ADH5	Associated with disease-free survival and metabolic functions	
ADRA1D	Related to benign prostatic hyperplasia	Kim et al. [111]
AGAP2	Facilitating the growth of the prostate	Zhao et al. [112]
AGAP3	Putative driver gene	Shimizu et al. [113]
AGRN	Critical to the tumour-promoting function of long noncoding RNA NEAT1	Li et al. [114]
AHRR	Tumour suppressor	Burris et al. [115]
AIFM1	The induction of apoptosis in a caspase-independent manner	Liu et al. [116]
AK5	Apoptosis inhibition and autophagy promotion	Zhang et al. [117]
AKAP7	Progression by regulating miR-526b-5p and SERP1	Mekhail et al. [118]
AKR1C3	Promoting epithelial-mesenchymal transition and metastasis by activating ERK signaling	Wang et al. [119]
ALDH1A2	Reduction in ALDH1A2 protein is an early event in human prostate cancer	Kim et al. [120]

## Data Availability

The datasets analyzed in this study could be found in [GSE141445] at the following: [https://www.ncbi.nlm.nih.gov/geo/query/acc.cgi?acc=GSE141445]; in [GSE54460] at [https://www.ncbi.nlm.nih.gov/geo/query/acc.cgi?acc=GSE54460]; in [GSE46602] at [https://www.ncbi.nlm.nih.gov/geo/query/acc.cgi?acc=GSE46602]; in [GSE70768] at [https://www.ncbi.nlm.nih.gov/geo/query/acc.cgi?acc=GSE70768]; and in [GSE70769] at [https://www.ncbi.nlm.nih.gov/geo/query/acc.cgi?acc=GSE70769].

## Supplementary Materials

Supplementary Materials Figure S1. Workflow of the analysis. Figure S2. Validation of the risk score model using the GSE54460 dataset. A. Patients with prostate cancer (PRAD) in the GSE54460 cohort are listed in ascending order of risk score. B. Progression-free interval (PFI) distribution versus the risk score of each patient in the GSE54460 cohort. C. Kaplan–Meier (KM) curves of patients with different risk levels in the GSE54460 validation set. D. Receiver Operating Characteristic (ROC) curve analysis for 1-, 3- and 5-year PFI using the clinical information of patients of the GSE54460 validation dataset. Figure S3. Validation of the risk score model using the GSE46602 dataset. A. Patients with prostate cancer (PRAD) in the GSE46602 cohort are listed in ascending order of risk score. B. Progression-free interval (PFI) distribution versus the risk score of each patient in the GSE46602 cohort. C. Kaplan–Meier (KM) curves of patients with different risk levels in the GSE46602 validation dataset. D. Receiver Operating Characteristic (ROC) curve analysis for 1-, 3- and 5-year PFI using the clinical information of patients of the GSE46602 validation dataset. Figure S4. Validation of the risk score model using the GSE70768 dataset. A. Patients with prostate cancer (PRAD) in the GSE70768 cohort are listed in ascending order of risk score. B. Progression-free interval (PFI) distribution versus the risk score of each patient in the GSE70768 cohort. C. Kaplan–Meier (KM) curves of patients with different risk levels in the GSE70768 validation dataset. D. Receiver Operating Characteristic (ROC) curve analysis for 1-, 3- and 5-year PFI using the clinical information of patients of the GSE70768 validation dataset. Figure S5. Validation of the risk score model using the GSE70769 dataset. A. Patients with prostate cancer (PRAD) in the GSE70769 validation dataset are listed in ascending order of risk score. B. Progression-free interval (PFI) distribution versus the risk score of each patient in the GSE70769 cohort. C. Kaplan–Meier (KM) curves of patients with different risk levels in the GSE70769 validation set. D. Receiver Operating Characteristic (ROC) curve analysis for 1-, 3- and 5-year PFI using the clinical information of patients of the GSE70768 validation dataset. Figure S6. Drug response is estimated by the half-maximal inhibitory concentration (IC_50_) value wherein the sensitivity of the high-risk patients is higher than that of the low-risk patients in the TCGA training cohort. Figure S7. Drug response is estimated by the half-maximal inhibitory concentration (IC_50_) value wherein the sensitivity of the high-risk patients is lower than that of the low-risk patients in the TCGA training cohort. Table S1. DEGs identified in the TCGA-PRAD cohort. Table S2. DEGs enrichment by GO. Table S3. DEGs enriched in KEGG pathway. Table S4. Immune score in each sample of TCGA-PRAD cohort. Table S5. The altered immune microenvironment in prostate cancer (PRAD). Table S6. GO enrichment for cluster 0. Table S7. GO enrichment for cluster 1. Table S8. GO enrichment for cluster 2. Table S9. GO enrichment for cluster 3. Table S10. GO enrichment for cluster 4. Table S11. GO enrichment for cluster 5. Table S12. GO enrichment for cluster 6. Table S13. GO enrichment for cluster 7. Table S14. Conversion of the gene symbols of DEGs into ENTREZ ID. Table S15. 453 differentially expressed marker genes in PRAD.
