# Supplementary material for: Integrative Analysis of Bulk RNA-Seq and Single-Cell RNA-Seq Unveils the Characteristics of the Immune Microenvironment and Prognosis Signature in Prostate Cancer
Source: J Oncol. 2022 Jul 19;2022:6768139. doi: 10.1155/2022/6768139 (PMC9325591; doi:10.1155/2022/6768139)
Supplement: Supplementary Materials — Figure S1. Workflow of the analysis. Figure S2. Validation of the risk score model using the GSE54460 dataset. A. Patients with prostate cancer (PRAD) in the GSE54460 cohort are listed in ascending order of risk score. B. Progression-free interval (PFI) distribution versus the risk score of each patient in the GSE54460 cohort. C. Kaplan–Meier (KM) curves of patients with different risk levels in the GSE54460 validation set. D. Receiver Operating Characteristic (ROC) curve analysis for 1-, 3- and 5-year PFI using the clinical information of patients of the GSE54460 validation dataset. Figure S3. Validation of the risk score model using the GSE46602 dataset. A. Patients with prostate cancer (PRAD) in the GSE46602 cohort are listed in ascending order of risk score. B. Progression-free interval (PFI) distribution versus the risk score of each patient in the GSE46602 cohort. C. Kaplan–Meier (KM) curves of patients with different risk levels in the GSE46602 validation dataset. D. Receiver Operating Characteristic (ROC) curve analysis for 1-, 3- and 5-year PFI using the clinical information of patients of the GSE46602 validation dataset. Figure S4. Validation of the risk score model using the GSE70768 dataset. A. Patients with prostate cancer (PRAD) in the GSE70768 cohort are listed in ascending order of risk score. B. Progression-free interval (PFI) distribution versus the risk score of each patient in the GSE70768 cohort. C. Kaplan–Meier (KM) curves of patients with different risk levels in the GSE70768 validation dataset. D. Receiver Operating Characteristic (ROC) curve analysis for 1-, 3- and 5-year PFI using the clinical information of patients of the GSE70768 validation dataset. Figure S5. Validation of the risk score model using the GSE70769 dataset. A. Patients with prostate cancer (PRAD) in the GSE70769 validation dataset are listed in ascending order of risk score. B. Progression-free interval (PFI) distribution versus the risk score of each patient in the GSE707 [file 6768139.f1.zip › 6768139.f1/Table S1.pdf]

| GeneSymbol | baseMean | log2FoldChange | lfcSE | stat | pvalue | padj | change |
|---|---|---|---|---|---|---|---|
| MFSD2A | 547.397751 | -6.089436103 | 0.212091271 | -28.7114 | 2.75E-181 | 3.61E-177 | DOWN |
| SERPINA5 | 1103.158444 | -6.843844106 | 0.25132288 | -27.2313 | 2.77E-163 | 1.82E-159 | DOWN |
| ACSL6 | 283.8521077 | -5.032831012 | 0.192634787 | -26.1263 | 1.83E-150 | 8.02E-147 | DOWN |
| AKR1B1 | 2617.914535 | -3.785834964 | 0.155176929 | -24.3969 | 1.85E-131 | 6.05E-128 | DOWN |
| MCF2 | 99.27570647 | -5.329594949 | 0.221729016 | -24.0365 | 1.15E-127 | 3.03E-124 | DOWN |
| KLHL14 | 165.0137371 | -4.355249739 | 0.181930114 | -23.9391 | 1.20E-126 | 2.62E-123 | DOWN |
| EMX2 | 250.961879 | -6.921566364 | 0.310494517 | -22.2921 | 4.41E-110 | 8.27E-107 | DOWN |
| NDRG4 | 377.8077206 | -3.302691938 | 0.148706329 | -22.2095 | 2.78E-109 | 4.56E-106 | DOWN |
| SLC2A9 | 261.2616179 | -2.696747276 | 0.122045142 | -22.0963 | 3.43E-108 | 5.00E-105 | DOWN |
| HOXB8 | 94.75335795 | -6.382338105 | 0.288914797 | -22.0907 | 3.88E-108 | 5.09E-105 | DOWN |
| SPINK2 | 353.8330227 | -7.606998884 | 0.349898926 | -21.7406 | 8.49E-105 | 1.01E-101 | DOWN |
| EMX2OS | 328.211919 | -6.06143359 | 0.281070726 | -21.5655 | 3.79E-103 | 4.14E-100 | DOWN |
| RASL10B | 254.3845766 | -3.501810543 | 0.163688842 | -21.3931 | 1.55E-101 | 1.56E-98 | DOWN |
| CRTAC1 | 318.7621565 | -4.359758361 | 0.205207635 | -21.2456 | 3.62E-100 | 3.39E-97 | DOWN |
| CYSLTR2 | 103.9511846 | -3.808674169 | 0.18090584 | -21.0534 | 2.13E-98 | 1.86E-95 | DOWN |
| CLDN2 | 478.5786464 | -7.938826864 | 0.385226245 | -20.6082 | 2.32E-94 | 1.90E-91 | DOWN |
| KCP | 81.31455289 | -3.572395585 | 0.180543815 | -19.7869 | 3.86E-87 | 2.98E-84 | DOWN |
| ANO1 | 2358.913355 | -2.925680737 | 0.149004888 | -19.6348 | 7.80E-86 | 5.69E-83 | DOWN |
| PAQR8 | 743.3167739 | -2.582337768 | 0.133194934 | -19.3877 | 9.81E-84 | 6.78E-81 | DOWN |
| KCNJ5 | 398.2766638 | -3.066849026 | 0.158532045 | -19.3453 | 2.23E-83 | 1.47E-80 | DOWN |
| ABCG2 | 845.2340297 | -2.824981606 | 0.146050521 | -19.3425 | 2.36E-83 | 1.47E-80 | DOWN |
| PATE2 | 63.53203613 | -5.470756301 | 0.288560654 | -18.9588 | 3.74E-80 | 2.23E-77 | DOWN |
| ST6GALNAC4 | 510.927775 | -1.983203264 | 0.105909661 | -18.7254 | 3.07E-78 | 1.75E-75 | DOWN |
| CA2 | 370.5306383 | -4.476875109 | 0.240272716 | -18.6325 | 1.75E-77 | 9.58E-75 | DOWN |
| PTGES | 1242.752029 | -3.142981571 | 0.16972735 | -18.5178 | 1.48E-76 | 7.79E-74 | DOWN |
| PNMT | 121.9918697 | -4.962071488 | 0.268681516 | -18.4682 | 3.72E-76 | 1.88E-73 | DOWN |
| STAC2 | 561.1757468 | -4.618947031 | 0.250614278 | -18.4305 | 7.48E-76 | 3.64E-73 | DOWN |
| GCNT4 | 137.9462036 | -2.377977678 | 0.129636138 | -18.3435 | 3.72E-75 | 1.74E-72 | DOWN |
| HOXB6 | 123.9311695 | -4.581101332 | 0.2519947 | -18.1794 | 7.52E-74 | 3.40E-71 | DOWN |
| PLA2G4A | 667.2056812 | -2.269077721 | 0.125637119 | -18.0606 | 6.51E-73 | 2.85E-70 | DOWN |
| WWC3 | 2747.599849 | -1.441694754 | 0.080113235 | -17.9957 | 2.10E-72 | 8.91E-70 | DOWN |
| FRMD3 | 267.993794 | -2.21376406 | 0.123474561 | -17.9289 | 7.01E-72 | 2.88E-69 | DOWN |
| RAB11FIP2 | 1532.105034 | -1.248648109 | 0.069769524 | -17.8968 | 1.25E-71 | 4.97E-69 | DOWN |
| MGAM | 64.92440565 | -4.777070681 | 0.270295549 | -17.6735 | 6.71E-70 | 2.59E-67 | DOWN |
| ANXA13 | 106.3568731 | -5.620687918 | 0.323121446 | -17.395 | 9.01E-68 | 3.38E-65 | DOWN |
| EPHA10 | 556.1193919 | 2.172472997 | 0.124976763 | 17.38302 | 1.11E-67 | 4.05E-65 | UP |
| PRDM16 | 85.30955998 | -2.574329505 | 0.149170848 | -17.2576 | 9.81E-67 | 3.48E-64 | DOWN |
| SLC16A12 | 59.04349166 | -3.745262011 | 0.217717463 | -17.2024 | 2.55E-66 | 8.80E-64 | DOWN |
| QPRT | 343.0099682 | -2.52554271 | 0.146840313 | -17.1992 | 2.69E-66 | 9.05E-64 | DOWN |
| PIP | 3502.392479 | -6.719511862 | 0.391451373 | -17.1656 | 4.80E-66 | 1.58E-63 | DOWN |
| PLLP | 463.0219647 | -1.747013564 | 0.103784808 | -16.833 | 1.40E-63 | 4.47E-61 | DOWN |
| SLC45A2 | 405.3392183 | 5.184764132 | 0.309851715 | 16.73305 | 7.53E-63 | 2.35E-60 | UP |
| HPN | 14938.40718 | 2.184903315 | 0.1306064 | 16.72891 | 8.07E-63 | 2.46E-60 | UP |
| RHOBTB2 | 1110.968017 | -1.321964534 | 0.080475985 | -16.4268 | 1.23E-60 | 3.67E-58 | DOWN |
| NKX2-3 | 120.9245652 | 4.261949395 | 0.260517595 | 16.35955 | 3.72E-60 | 1.08E-57 | UP |
| SNAP25 | 218.2417456 | -3.03612954 | 0.185720745 | -16.3478 | 4.51E-60 | 1.29E-57 | DOWN |
| FUT3 | 56.66707812 | -4.198380278 | 0.257211287 | -16.3227 | 6.81E-60 | 1.90E-57 | DOWN |
| GNAO1 | 606.7697625 | -2.515948249 | 0.154561816 | -16.2779 | 1.42E-59 | 3.87E-57 | DOWN |
| GSTM3 | 2984.331994 | -2.329755384 | 0.143637692 | -16.2197 | 3.66E-59 | 9.81E-57 | DOWN |
| ABHD6 | 464.6144783 | -1.302903314 | 0.080646737 | -16.1557 | 1.04E-58 | 2.72E-56 | DOWN |
| G0S2 | 311.6926043 | -2.278677508 | 0.142129221 | -16.0324 | 7.59E-58 | 1.95E-55 | DOWN |
| LPCAT2 | 1022.035971 | -1.786661993 | 0.111512556 | -16.0221 | 8.96E-58 | 2.26E-55 | DOWN |
| EFNB1 | 1170.933881 | -1.409537254 | 0.088386486 | -15.9474 | 2.97E-57 | 7.35E-55 | DOWN |
| KCNJ15 | 213.59077 | -3.309784501 | 0.210125583 | -15.7515 | 6.71E-56 | 1.60E-53 | DOWN |
| GLIS3 | 492.0722572 | -2.133930749 | 0.136226905 | -15.6645 | 2.64E-55 | 6.20E-53 | DOWN |
| KCNQ1 | 2105.054787 | -2.089813708 | 0.133634159 | -15.6383 | 3.99E-55 | 9.19E-53 | DOWN |
| LAPTM4A | 16669.9414 | -1.015007479 | 0.065457567 | -15.5063 | 3.14E-54 | 7.11E-52 | DOWN |
| ARHGEF38 | 2402.401798 | 1.670988 | 0.107994312 | 15.47293 | 5.28E-54 | 1.18E-51 | UP |
| UNC5B | 1746.625336 | -2.268225411 | 0.147808942 | -15.3457 | 3.79E-53 | 8.28E-51 | DOWN |
| PAX2 | 83.29266495 | -4.163506705 | 0.271700664 | -15.3239 | 5.30E-53 | 1.14E-50 | DOWN |
| LPL | 863.3127909 | -3.781254817 | 0.246875623 | -15.3164 | 5.94E-53 | 1.26E-50 | DOWN |
| HOXC6 | 714.0659889 | 2.972363804 | 0.194529876 | 15.27973 | 1.04E-52 | 2.17E-50 | UP |

| | | | | | | | |
|---|---|---|---|---|---|---|---|
| GPRC5B | 1015.949196 | -1.852299523 | 0.121633728 | -15.2285 | 2.29E-52 | 4.69E-50 | DOWN |
| SIM2 | 4225.499514 | 2.224215122 | 0.146496449 | 15.18272 | 4.60E-52 | 9.29E-50 | UP |
| GSTP1 | 7542.42729 | -2.350251418 | 0.154897655 | -15.1729 | 5.34E-52 | 1.06E-49 | DOWN |
| AMACR | 7838.633147 | 3.27238178 | 0.216689383 | 15.10172 | 1.58E-51 | 3.09E-49 | UP |
| SP6 | 278.1967859 | -2.069279872 | 0.137134897 | -15.0894 | 1.90E-51 | 3.67E-49 | DOWN |
| PIK3C2G | 76.2724522 | -4.495328762 | 0.300800942 | -14.9445 | 1.69E-50 | 3.22E-48 | DOWN |
| ONECUT2 | 494.0425162 | 3.438815348 | 0.230125892 | 14.94319 | 1.73E-50 | 3.23E-48 | UP |
| HOXB9 | 115.3069616 | -3.721000305 | 0.249779105 | -14.8972 | 3.44E-50 | 6.36E-48 | DOWN |
| SLC31A2 | 65.24399455 | -2.052424887 | 0.138649693 | -14.803 | 1.40E-49 | 2.56E-47 | DOWN |
| PALM3 | 160.3022992 | -2.967691233 | 0.200582955 | -14.7953 | 1.57E-49 | 2.82E-47 | DOWN |
| ZIC2 | 214.229832 | 4.472915432 | 0.302401548 | 14.79131 | 1.67E-49 | 2.96E-47 | UP |
| NAGS | 144.2870737 | -1.609297136 | 0.108913859 | -14.7759 | 2.10E-49 | 3.67E-47 | DOWN |
| CLU | 25266.47137 | -2.289242309 | 0.155169119 | -14.7532 | 2.93E-49 | 5.07E-47 | DOWN |
| MICALL1 | 2469.938894 | -1.179125496 | 0.080031534 | -14.7333 | 3.94E-49 | 6.72E-47 | DOWN |
| TMEM114 | 71.05660661 | -9.369384084 | 0.638332489 | -14.6779 | 8.93E-49 | 1.50E-46 | DOWN |
| GNAZ | 563.9791969 | -1.848569298 | 0.126187972 | -14.6493 | 1.36E-48 | 2.26E-46 | DOWN |
| PRPS2 | 3218.922814 | -1.346857289 | 0.092354735 | -14.5835 | 3.58E-48 | 5.87E-46 | DOWN |
| GRAMD1B | 137.5296207 | -1.880850857 | 0.129233904 | -14.5538 | 5.52E-48 | 8.95E-46 | DOWN |
| CDK18 | 617.5865474 | -1.772893286 | 0.121915846 | -14.5419 | 6.57E-48 | 1.05E-45 | DOWN |
| PJA1 | 1626.129534 | -1.005227868 | 0.069220924 | -14.522 | 8.79E-48 | 1.39E-45 | DOWN |
| FAM167A | 231.4710212 | -2.912985373 | 0.201921865 | -14.4263 | 3.54E-47 | 5.52E-45 | DOWN |
| ZNF185 | 2534.152979 | -2.067386852 | 0.143528226 | -14.404 | 4.88E-47 | 7.54E-45 | DOWN |
| ZNF219 | 1618.658034 | -1.235669892 | 0.085947469 | -14.377 | 7.21E-47 | 1.10E-44 | DOWN |
| CAMK2G | 2680.478298 | -1.025672857 | 0.071650758 | -14.3149 | 1.77E-46 | 2.66E-44 | DOWN |
| TRIM36 | 2712.174281 | 1.558995208 | 0.109706697 | 14.21057 | 7.88E-46 | 1.16E-43 | UP |
| ETNK2 | 553.8189009 | -1.34590019 | 0.094745391 | -14.2054 | 8.48E-46 | 1.24E-43 | DOWN |
| CGREF1 | 1553.436519 | 1.9025755 | 0.133966368 | 14.20189 | 8.92E-46 | 1.29E-43 | UP |
| C11orf45 | 91.70931951 | -1.879644233 | 0.132432575 | -14.1932 | 1.01E-45 | 1.44E-43 | DOWN |
| APOBEC3G | 452.7101341 | -1.932074315 | 0.1363636 | -14.1685 | 1.43E-45 | 2.02E-43 | DOWN |
| EZH2 | 411.0261805 | 1.634237608 | 0.115443637 | 14.15615 | 1.71E-45 | 2.39E-43 | UP |
| ZIC5 | 103.4527833 | 5.237158911 | 0.37000318 | 14.15436 | 1.76E-45 | 2.42E-43 | UP |
| AOX1 | 1645.545758 | -2.428811295 | 0.171950899 | -14.125 | 2.66E-45 | 3.64E-43 | DOWN |
| UCN | 102.9496754 | 2.070089581 | 0.146643833 | 14.11644 | 3.01E-45 | 4.07E-43 | UP |
| PATE4 | 550.4712761 | -9.648400826 | 0.684849031 | -14.0884 | 4.48E-45 | 6.00E-43 | DOWN |
| PLBD1 | 438.8669183 | -1.501276943 | 0.107030824 | -14.0266 | 1.07E-44 | 1.42E-42 | DOWN |
| SLCO2A1 | 3691.664767 | -1.820546172 | 0.129930329 | -14.0117 | 1.32E-44 | 1.73E-42 | DOWN |
| APOBEC3C | 1645.46963 | -2.072610045 | 0.148094165 | -13.9952 | 1.67E-44 | 2.17E-42 | DOWN |
| RASAL1 | 57.40105828 | -3.129652175 | 0.223658838 | -13.993 | 1.72E-44 | 2.21E-42 | DOWN |
| PAQR7 | 798.7831557 | -1.432836783 | 0.102644137 | -13.9593 | 2.76E-44 | 3.49E-42 | DOWN |
| PDE7B | 320.6907647 | -1.604245396 | 0.11501234 | -13.9485 | 3.21E-44 | 4.02E-42 | DOWN |
| SNPH | 663.5541927 | -1.843854785 | 0.132328635 | -13.9339 | 3.94E-44 | 4.88E-42 | DOWN |
| PYCR1 | 8858.526218 | 1.226742607 | 0.088319937 | 13.88976 | 7.31E-44 | 8.96E-42 | UP |
| YPEL1 | 318.0079276 | 1.454066174 | 0.10511714 | 13.83282 | 1.62E-43 | 1.96E-41 | UP |
| LRFN1 | 497.0593598 | 1.448639202 | 0.104825166 | 13.81957 | 1.94E-43 | 2.34E-41 | UP |
| SLC19A1 | 2978.395887 | 1.191124806 | 0.086465546 | 13.77572 | 3.57E-43 | 4.26E-41 | UP |
| ISYNA1 | 2128.428143 | -1.504065417 | 0.109617458 | -13.721 | 7.60E-43 | 8.98E-41 | DOWN |
| CA14 | 152.7052244 | -2.61873247 | 0.191011808 | -13.7098 | 8.87E-43 | 1.04E-40 | DOWN |
| TMLHE | 141.4515102 | -1.552073106 | 0.113527485 | -13.6713 | 1.51E-42 | 1.75E-40 | DOWN |
| LIPG | 312.7837759 | -2.847837452 | 0.208894657 | -13.6329 | 2.55E-42 | 2.94E-40 | DOWN |
| NETO2 | 556.3032834 | 2.207368653 | 0.162111912 | 13.61633 | 3.20E-42 | 3.66E-40 | UP |
| LMX1B | 119.5617709 | 3.077865925 | 0.226307398 | 13.60038 | 3.98E-42 | 4.51E-40 | UP |
| SCARA3 | 1894.286323 | -1.809924664 | 0.13401883 | -13.505 | 1.46E-41 | 1.64E-39 | DOWN |
| MARCKSL1 | 22564.34202 | 1.250927402 | 0.092785336 | 13.48195 | 2.00E-41 | 2.22E-39 | UP |
| SLC13A2 | 92.48844568 | -6.242360378 | 0.463366764 | -13.4717 | 2.29E-41 | 2.53E-39 | DOWN |
| B3GNT8 | 147.7395844 | -1.689885471 | 0.125666847 | -13.4473 | 3.19E-41 | 3.43E-39 | DOWN |
| PTGS1 | 1212.974317 | -2.587238265 | 0.192526316 | -13.4384 | 3.60E-41 | 3.84E-39 | DOWN |
| AFAP1L2 | 1176.4484 | -1.637254267 | 0.12198058 | -13.4223 | 4.48E-41 | 4.70E-39 | DOWN |
| HOXB7 | 145.4400793 | -2.532106638 | 0.189345685 | -13.3729 | 8.70E-41 | 9.07E-39 | DOWN |
| SLMAP | 5807.516967 | -1.113663831 | 0.083335442 | -13.3636 | 9.86E-41 | 1.02E-38 | DOWN |
| DNAH5 | 2345.338143 | 2.370091485 | 0.177649078 | 13.34142 | 1.33E-40 | 1.36E-38 | UP |
| NYNRIN | 946.4484288 | -1.962914113 | 0.1472054 | -13.3345 | 1.46E-40 | 1.48E-38 | DOWN |
| SLC26A6 | 1347.387079 | 1.300655231 | 0.097694116 | 13.31355 | 1.93E-40 | 1.95E-38 | UP |
| TGFBR3 | 2132.048807 | -1.627316774 | 0.122484366 | -13.2859 | 2.79E-40 | 2.80E-38 | DOWN |

| | | | | | | | |
|---|---|---|---|---|---|---|---|
| AIF1L | 1770.544699 | -1.662864502 | 0.125343128 | -13.2665 | 3.62E-40 | 3.60E-38 | DOWN |
| RRAS | 3812.395754 | -1.318248682 | 0.099394367 | -13.2628 | 3.80E-40 | 3.75E-38 | DOWN |
| PDK4 | 6649.394204 | -2.764287495 | 0.209080836 | -13.2211 | 6.63E-40 | 6.49E-38 | DOWN |
| DOK4 | 852.8508522 | -1.166281806 | 0.088455681 | -13.1849 | 1.07E-39 | 1.03E-37 | DOWN |
| PTGS2 | 4030.034872 | -2.994413766 | 0.227246578 | -13.1769 | 1.19E-39 | 1.14E-37 | DOWN |
| RAB17 | 1895.70189 | 1.348008224 | 0.102318375 | 13.17464 | 1.23E-39 | 1.17E-37 | UP |
| SNHG3 | 1922.361686 | 1.652007737 | 0.12585805 | 13.12596 | 2.34E-39 | 2.18E-37 | UP |
| RBM38 | 1605.2467 | -1.23909024 | 0.094604355 | -13.0976 | 3.40E-39 | 3.14E-37 | DOWN |
| TSPAN18 | 1156.667453 | -1.768781109 | 0.135184802 | -13.0842 | 4.06E-39 | 3.72E-37 | DOWN |
| SLIT1 | 918.0594327 | 2.919095218 | 0.224189153 | 13.02068 | 9.33E-39 | 8.45E-37 | UP |
| VWA5B2 | 127.714642 | -2.482980284 | 0.19102221 | -12.9984 | 1.25E-38 | 1.12E-36 | DOWN |
| ALDH1L2 | 244.6668654 | -1.879732811 | 0.144628634 | -12.997 | 1.27E-38 | 1.13E-36 | DOWN |
| C20orf194 | 1245.114469 | -1.175080771 | 0.090436267 | -12.9935 | 1.33E-38 | 1.17E-36 | DOWN |
| SMPDL3B | 1407.278805 | 1.723578046 | 0.133167065 | 12.94298 | 2.57E-38 | 2.24E-36 | UP |
| CPLX1 | 417.5024937 | 1.424991433 | 0.110101637 | 12.94251 | 2.59E-38 | 2.24E-36 | UP |
| ME1 | 584.3974042 | -1.751804949 | 0.135464841 | -12.9318 | 2.98E-38 | 2.55E-36 | DOWN |
| BIRC5 | 423.5400805 | 2.411359872 | 0.186667032 | 12.91797 | 3.56E-38 | 3.00E-36 | UP |
| C2orf88 | 291.2008323 | -2.264581552 | 0.175378918 | -12.9125 | 3.83E-38 | 3.20E-36 | DOWN |
| SOX15 | 214.5317363 | -1.574626005 | 0.122405372 | -12.864 | 7.17E-38 | 5.92E-36 | DOWN |
| UGT2B4 | 386.6668263 | 4.641073735 | 0.360994696 | 12.85635 | 7.92E-38 | 6.50E-36 | UP |
| NDRG2 | 5693.935922 | -1.429056185 | 0.111235045 | -12.8472 | 8.92E-38 | 7.27E-36 | DOWN |
| RAB9B | 302.2726335 | -1.762487517 | 0.137526233 | -12.8156 | 1.34E-37 | 1.09E-35 | DOWN |
| E2F5 | 665.2050318 | 1.127923265 | 0.088206398 | 12.78732 | 1.93E-37 | 1.55E-35 | UP |
| SLC26A3 | 1088.072491 | -4.860299157 | 0.380373862 | -12.7777 | 2.18E-37 | 1.75E-35 | DOWN |
| DUSP2 | 1210.897259 | -2.490348278 | 0.194920021 | -12.7763 | 2.23E-37 | 1.77E-35 | DOWN |
| DLGAP5 | 149.7751396 | 2.330468733 | 0.182887947 | 12.7426 | 3.43E-37 | 2.68E-35 | UP |
| SNHG4 | 206.6906855 | 2.18500379 | 0.171647518 | 12.7296 | 4.05E-37 | 3.13E-35 | UP |
| PHGR1 | 191.8200076 | 3.635220657 | 0.285628186 | 12.72711 | 4.18E-37 | 3.21E-35 | UP |
| ANXA9 | 377.5714536 | -1.521882558 | 0.119795293 | -12.704 | 5.62E-37 | 4.29E-35 | DOWN |
| GATM | 556.5687776 | -1.428443768 | 0.112461954 | -12.7016 | 5.80E-37 | 4.40E-35 | DOWN |
| GAS2L1 | 2300.333372 | -1.040841468 | 0.08212901 | -12.6732 | 8.32E-37 | 6.28E-35 | DOWN |
| ANGPT1 | 890.0138732 | -1.997018597 | 0.157882518 | -12.6488 | 1.14E-36 | 8.48E-35 | DOWN |
| ARC | 148.3859405 | -2.837274031 | 0.224433763 | -12.6419 | 1.24E-36 | 9.19E-35 | DOWN |
| PAX8 | 240.346924 | -3.111319507 | 0.247065116 | -12.5931 | 2.30E-36 | 1.70E-34 | DOWN |
| PLEKHA2 | 1244.042703 | -1.258604971 | 0.100138583 | -12.5686 | 3.14E-36 | 2.29E-34 | DOWN |
| POU2F3 | 119.5680461 | -1.449538211 | 0.115354245 | -12.566 | 3.25E-36 | 2.36E-34 | DOWN |
| STIL | 431.0513433 | 1.321864868 | 0.105777738 | 12.49663 | 7.79E-36 | 5.59E-34 | UP |
| ILK | 970.5650102 | -1.158044628 | 0.092974409 | -12.4555 | 1.30E-35 | 9.26E-34 | DOWN |
| KCNJ16 | 134.5571447 | -5.391410828 | 0.432935795 | -12.4531 | 1.34E-35 | 9.49E-34 | DOWN |
| NEK5 | 516.4659042 | 1.975921172 | 0.158973423 | 12.42925 | 1.81E-35 | 1.27E-33 | UP |
| MMP26 | 151.7792914 | 3.266116075 | 0.262943356 | 12.42137 | 2.00E-35 | 1.40E-33 | UP |
| NEURL1B | 1696.327951 | -1.331465348 | 0.107222526 | -12.4178 | 2.09E-35 | 1.45E-33 | DOWN |
| C1QTNF1 | 1868.476208 | -1.745315181 | 0.140577716 | -12.4153 | 2.16E-35 | 1.49E-33 | DOWN |
| NLRP12 | 109.8834388 | 2.651397326 | 0.21356132 | 12.41516 | 2.16E-35 | 1.49E-33 | UP |
| AQP2 | 1075.926535 | -9.246404362 | 0.745192225 | -12.4081 | 2.36E-35 | 1.62E-33 | DOWN |
| AMOT | 2003.795827 | -1.743697187 | 0.140654254 | -12.397 | 2.71E-35 | 1.83E-33 | DOWN |
| ARL4D | 396.6720832 | -1.834842295 | 0.148219144 | -12.3793 | 3.38E-35 | 2.27E-33 | DOWN |
| SEC23A | 2321.288279 | -1.073787502 | 0.086826476 | -12.3671 | 3.94E-35 | 2.63E-33 | DOWN |
| TRPM4 | 30801.99362 | 1.347347368 | 0.109043958 | 12.356 | 4.52E-35 | 3.00E-33 | UP |
| PCSK6 | 924.3779512 | 1.710281194 | 0.138580375 | 12.34144 | 5.42E-35 | 3.57E-33 | UP |
| KIF4A | 256.4763694 | 2.31435944 | 0.187592841 | 12.33714 | 5.71E-35 | 3.75E-33 | UP |
| ATP8A2 | 818.7390227 | 3.644419225 | 0.295424527 | 12.33621 | 5.78E-35 | 3.78E-33 | UP |
| CST2 | 364.7632246 | 3.794650367 | 0.308308504 | 12.30797 | 8.21E-35 | 5.33E-33 | UP |
| KCNIP3 | 260.6531558 | -1.325926768 | 0.107751656 | -12.3054 | 8.47E-35 | 5.48E-33 | DOWN |
| PLEKHG3 | 1485.301918 | -1.088084576 | 0.088516001 | -12.2925 | 9.94E-35 | 6.36E-33 | DOWN |
| VWCE | 70.07827233 | -1.545545347 | 0.125871378 | -12.2788 | 1.18E-34 | 7.47E-33 | DOWN |
| CAMKK2 | 19289.7595 | 1.566284654 | 0.1275839 | 12.27651 | 1.21E-34 | 7.64E-33 | UP |
| PCA3 | 19831.68815 | 3.461501247 | 0.282493505 | 12.25338 | 1.61E-34 | 1.01E-32 | UP |
| TBX2 | 1187.809766 | -1.562102279 | 0.12751433 | -12.2504 | 1.67E-34 | 1.04E-32 | DOWN |
| LYVE1 | 147.9359435 | -2.206921683 | 0.180313174 | -12.2394 | 1.91E-34 | 1.18E-32 | DOWN |
| RRM2 | 474.6379126 | 2.179562896 | 0.178163966 | 12.23347 | 2.06E-34 | 1.26E-32 | UP |
| PRR7 | 209.3769861 | 1.735281681 | 0.141905379 | 12.22844 | 2.19E-34 | 1.34E-32 | UP |
| FBLIM1 | 4419.756609 | -1.006095453 | 0.082349444 | -12.2174 | 2.51E-34 | 1.53E-32 | DOWN |

| | | | | | | | |
|---|---|---|---|---|---|---|---|
| LRRC56 | 671.2655688 | 1.092930192 | 0.089538911 | 12.2062 | 2.88E-34 | 1.74E-32 | UP |
| RANBP17 | 354.2169815 | 1.032804747 | 0.084613752 | 12.20611 | 2.88E-34 | 1.74E-32 | UP |
| DUOX1 | 839.8336291 | -2.155039757 | 0.176737075 | -12.1935 | 3.37E-34 | 2.02E-32 | DOWN |
| SLC25A45 | 825.7337879 | 1.195305714 | 0.098101495 | 12.18438 | 3.77E-34 | 2.24E-32 | UP |
| CAV1 | 4647.626817 | -1.767490113 | 0.14531727 | -12.163 | 4.89E-34 | 2.88E-32 | DOWN |
| SLPI | 3026.227696 | -3.354325693 | 0.275981193 | -12.1542 | 5.45E-34 | 3.19E-32 | DOWN |
| IL18R1 | 159.4465977 | -1.605818974 | 0.132135722 | -12.1528 | 5.54E-34 | 3.22E-32 | DOWN |
| MPP6 | 1070.560495 | 1.353830303 | 0.111415134 | 12.15122 | 5.65E-34 | 3.27E-32 | UP |
| RHBDL3 | 80.45959105 | -1.696895078 | 0.139839013 | -12.1346 | 6.92E-34 | 3.98E-32 | DOWN |
| CCNA2 | 232.3320654 | 1.792327648 | 0.147836151 | 12.12374 | 7.91E-34 | 4.51E-32 | UP |
| TCF7L1 | 811.0679602 | -1.287574369 | 0.106258598 | -12.1174 | 8.55E-34 | 4.83E-32 | DOWN |
| PCDH7 | 1516.154117 | -1.911491403 | 0.157778541 | -12.115 | 8.79E-34 | 4.94E-32 | DOWN |
| ANO6 | 3003.117478 | -1.377638235 | 0.11371376 | -12.115 | 8.80E-34 | 4.94E-32 | DOWN |
| TP53INP1 | 9246.71329 | 1.174433647 | 0.09702723 | 12.10417 | 1.00E-33 | 5.61E-32 | UP |
| C2CD4C | 139.7955263 | 2.274981655 | 0.188231008 | 12.08612 | 1.25E-33 | 6.96E-32 | UP |
| PIP5K1B | 209.0619975 | -1.706512512 | 0.141209738 | -12.0849 | 1.27E-33 | 7.02E-32 | DOWN |
| C2orf72 | 3274.686308 | 2.278041498 | 0.188537658 | 12.08269 | 1.30E-33 | 7.19E-32 | UP |
| TMEM37 | 162.2611816 | -1.642272251 | 0.136040308 | -12.072 | 1.49E-33 | 8.12E-32 | DOWN |
| MELK | 157.1880917 | 2.184610924 | 0.181006591 | 12.06923 | 1.54E-33 | 8.36E-32 | UP |
| C16orf45 | 1175.058896 | -1.280855318 | 0.106282976 | -12.0514 | 1.91E-33 | 1.03E-31 | DOWN |
| HMMR | 229.6331345 | 2.053413888 | 0.170714715 | 12.02834 | 2.52E-33 | 1.35E-31 | UP |
| MYBL2 | 369.2319118 | 2.416865864 | 0.200945834 | 12.02745 | 2.55E-33 | 1.35E-31 | UP |
| ALB | 241.2523065 | 3.95257207 | 0.329194544 | 12.0068 | 3.27E-33 | 1.73E-31 | UP |
| TLE2 | 2573.380135 | -1.041190809 | 0.08672312 | -12.0059 | 3.31E-33 | 1.74E-31 | DOWN |
| PDIA2 | 162.2666381 | 3.651841357 | 0.304228534 | 12.00361 | 3.40E-33 | 1.79E-31 | UP |
| COL2A1 | 4368.105867 | 5.013727557 | 0.417702058 | 12.00312 | 3.42E-33 | 1.79E-31 | UP |
| C15orf41 | 356.9488218 | -1.19814106 | 0.099830072 | -12.0018 | 3.48E-33 | 1.81E-31 | DOWN |
| MATK | 572.8535439 | 2.356316611 | 0.196384865 | 11.99846 | 3.62E-33 | 1.88E-31 | UP |
| LMO3 | 536.4818113 | -2.097873333 | 0.174897133 | -11.9949 | 3.78E-33 | 1.95E-31 | DOWN |
| SAMD12 | 459.0515052 | -1.759825383 | 0.146756941 | -11.9914 | 3.94E-33 | 2.03E-31 | DOWN |
| PAQR6 | 690.8883673 | 2.128840701 | 0.177601024 | 11.98665 | 4.17E-33 | 2.13E-31 | UP |
| APOC1 | 1018.176098 | 2.281431142 | 0.190490393 | 11.97662 | 4.71E-33 | 2.40E-31 | UP |
| PAK3 | 224.0681044 | -1.804007805 | 0.150707792 | -11.9702 | 5.09E-33 | 2.58E-31 | DOWN |
| CRISP1 | 195.093321 | -9.19126038 | 0.768394621 | -11.9616 | 5.64E-33 | 2.85E-31 | DOWN |
| GOLM1 | 79115.45041 | 1.714801534 | 0.143384302 | 11.95948 | 5.79E-33 | 2.91E-31 | UP |
| ESRP2 | 3939.958828 | 1.036670196 | 0.086709013 | 11.95574 | 6.06E-33 | 3.04E-31 | UP |
| C12orf75 | 1612.290692 | -1.552423933 | 0.129894554 | -11.9514 | 6.38E-33 | 3.19E-31 | DOWN |
| NOX4 | 125.6142109 | 2.308115551 | 0.193246762 | 11.94388 | 6.99E-33 | 3.47E-31 | UP |
| CHEK1 | 592.4943353 | -1.637307929 | 0.137233868 | -11.9308 | 8.18E-33 | 4.05E-31 | DOWN |
| KCNG3 | 211.7710016 | 3.260163015 | 0.273729207 | 11.91018 | 1.05E-32 | 5.15E-31 | UP |
| HRASLS5 | 56.50905371 | -2.177552799 | 0.182914038 | -11.9048 | 1.12E-32 | 5.47E-31 | DOWN |
| MKI67 | 1012.882874 | 1.912501322 | 0.160714171 | 11.90002 | 1.18E-32 | 5.77E-31 | UP |
| IRF5 | 430.3674778 | -1.373996327 | 0.115490486 | -11.8971 | 1.23E-32 | 5.94E-31 | DOWN |
| TES | 4340.383378 | -1.12068277 | 0.094207405 | -11.8959 | 1.24E-32 | 6.00E-31 | DOWN |
| MAMLD1 | 248.7272174 | -1.519779669 | 0.128077417 | -11.8661 | 1.78E-32 | 8.47E-31 | DOWN |
| SCPEP1 | 4482.171445 | -1.014600661 | 0.085511806 | -11.865 | 1.80E-32 | 8.55E-31 | DOWN |
| RASL11B | 206.1025884 | -1.87915051 | 0.158551045 | -11.852 | 2.10E-32 | 9.95E-31 | DOWN |
| HOXB2 | 247.2276261 | -1.693490022 | 0.142975408 | -11.8446 | 2.29E-32 | 1.08E-30 | DOWN |
| CTU1 | 263.1868217 | 1.078340428 | 0.091050247 | 11.84336 | 2.33E-32 | 1.09E-30 | UP |
| ZNF485 | 235.2291575 | 1.007712757 | 0.0851257 | 11.83794 | 2.48E-32 | 1.16E-30 | UP |
| ZNF154 | 181.3169174 | -1.440125485 | 0.121689098 | -11.8345 | 2.59E-32 | 1.21E-30 | DOWN |
| SNORD104 | 106.2179442 | 2.448123264 | 0.207319389 | 11.80846 | 3.53E-32 | 1.63E-30 | UP |
| GTSE1 | 139.8095456 | 2.136737973 | 0.181003126 | 11.80498 | 3.68E-32 | 1.69E-30 | UP |
| SLCO3A1 | 539.4164158 | -1.302539481 | 0.110365414 | -11.8021 | 3.81E-32 | 1.74E-30 | DOWN |
| CHTF18 | 827.8922292 | 1.105740135 | 0.093731332 | 11.79691 | 4.05E-32 | 1.85E-30 | UP |
| HFE | 500.2034358 | -1.299751144 | 0.110257473 | -11.7883 | 4.48E-32 | 2.04E-30 | DOWN |
| SBK1 | 1526.512883 | 1.238813635 | 0.105106395 | 11.78628 | 4.59E-32 | 2.08E-30 | UP |
| CBX8 | 582.5690108 | 1.09867667 | 0.093237329 | 11.78366 | 4.74E-32 | 2.14E-30 | UP |
| STX19 | 715.3481634 | 1.701506841 | 0.144489802 | 11.77596 | 5.19E-32 | 2.33E-30 | UP |
| FRMD6 | 2183.541351 | -1.47454833 | 0.125493813 | -11.75 | 7.06E-32 | 3.15E-30 | DOWN |
| CTTNBP2 | 264.9649795 | -1.416466587 | 0.120623641 | -11.7429 | 7.68E-32 | 3.42E-30 | DOWN |
| TMEM132C | 139.9331158 | -2.47822586 | 0.211113553 | -11.7388 | 8.06E-32 | 3.57E-30 | DOWN |
| B4GALNT4 | 1371.749729 | 1.738426625 | 0.148157528 | 11.73364 | 8.57E-32 | 3.76E-30 | UP |

| | | | | | | | |
|---|---|---|---|---|---|---|---|
| RIMS3 | 298.4610928 | -1.219353748 | 0.103986708 | -11.7261 | 9.37E-32 | 4.10E-30 | DOWN |
| HOXC4 | 339.7979032 | 2.367982189 | 0.202038933 | 11.72043 | 1.00E-31 | 4.37E-30 | UP |
| PAEP | 226.5581282 | -9.546592026 | 0.814545191 | -11.7202 | 1.00E-31 | 4.37E-30 | DOWN |
| GATA3 | 705.7553246 | -2.214120236 | 0.188949199 | -11.7181 | 1.03E-31 | 4.46E-30 | DOWN |
| CCDC78 | 270.2876996 | 2.441567001 | 0.208822819 | 11.69205 | 1.40E-31 | 6.00E-30 | UP |
| C16orf74 | 101.6823564 | -1.606561908 | 0.137438555 | -11.6893 | 1.45E-31 | 6.18E-30 | DOWN |
| ASPN | 813.3549774 | 2.073070326 | 0.177365507 | 11.68813 | 1.47E-31 | 6.25E-30 | UP |
| NCAPG | 171.9357773 | 2.047654323 | 0.175200107 | 11.68752 | 1.48E-31 | 6.27E-30 | UP |
| KCTD14 | 236.4405699 | -1.968596925 | 0.168472776 | -11.685 | 1.52E-31 | 6.44E-30 | DOWN |
| C22orf23 | 112.4553091 | -1.137494833 | 0.097487622 | -11.6681 | 1.86E-31 | 7.83E-30 | DOWN |
| NCS1 | 2817.475437 | -1.277515517 | 0.109563271 | -11.6601 | 2.04E-31 | 8.58E-30 | DOWN |
| PDLIM5 | 83111.3357 | 1.74854106 | 0.150182059 | 11.64281 | 2.50E-31 | 1.04E-29 | UP |
| HSPA6 | 446.0844202 | -3.510324949 | 0.301582923 | -11.6397 | 2.59E-31 | 1.08E-29 | DOWN |
| PAQR5 | 72.44820067 | -1.573858113 | 0.135250643 | -11.6366 | 2.68E-31 | 1.12E-29 | DOWN |
| PLP2 | 2212.734355 | -1.210503361 | 0.10425892 | -11.6105 | 3.64E-31 | 1.50E-29 | DOWN |
| DEPDC1B | 100.7875669 | 2.217983786 | 0.191050669 | 11.6094 | 3.69E-31 | 1.51E-29 | UP |
| POU5F1B | 186.1614548 | 1.711484184 | 0.147584726 | 11.59662 | 4.29E-31 | 1.75E-29 | UP |
| FOXQ1 | 329.1802982 | -2.34112119 | 0.202155791 | -11.5808 | 5.16E-31 | 2.10E-29 | DOWN |
| APOF | 488.6982058 | 2.550245758 | 0.220373916 | 11.57236 | 5.69E-31 | 2.30E-29 | UP |
| DBNDD2 | 120.2726592 | -1.184327992 | 0.102629228 | -11.5399 | 8.31E-31 | 3.34E-29 | DOWN |
| SVIL | 8641.357139 | -1.461729861 | 0.126680033 | -11.5388 | 8.41E-31 | 3.38E-29 | DOWN |
| MECOM | 784.7296673 | -1.445484443 | 0.125438512 | -11.5235 | 1.01E-30 | 4.02E-29 | DOWN |
| DCHS2 | 84.86453942 | -2.210793953 | 0.191861431 | -11.5229 | 1.01E-30 | 4.04E-29 | DOWN |
| RHPN1 | 1863.164507 | 1.352605932 | 0.117439104 | 11.51751 | 1.08E-30 | 4.28E-29 | UP |
| B3GNT9 | 791.2589993 | -1.40525059 | 0.122037232 | -11.5149 | 1.11E-30 | 4.40E-29 | DOWN |
| DMPK | 3461.83076 | -1.300766639 | 0.112979289 | -11.5133 | 1.13E-30 | 4.47E-29 | DOWN |
| PGF | 384.9948641 | -1.744171121 | 0.151607473 | -11.5045 | 1.25E-30 | 4.91E-29 | DOWN |
| GSTM4 | 806.5290547 | -1.212669883 | 0.105408518 | -11.5045 | 1.25E-30 | 4.91E-29 | DOWN |
| DLX2 | 173.99514 | 3.257091711 | 0.283187284 | 11.50155 | 1.30E-30 | 5.06E-29 | UP |
| FAM110C | 486.0406926 | -2.218209942 | 0.192876364 | -11.5007 | 1.31E-30 | 5.10E-29 | DOWN |
| SNCG | 709.3814351 | -1.897146157 | 0.164990653 | -11.4985 | 1.34E-30 | 5.21E-29 | DOWN |
| SYT8 | 53.8906653 | -2.928802967 | 0.254760979 | -11.4963 | 1.38E-30 | 5.33E-29 | DOWN |
| DNAJB5 | 1270.293794 | -1.516807663 | 0.132039789 | -11.4875 | 1.52E-30 | 5.88E-29 | DOWN |
| MPP2 | 409.1947555 | -1.517045099 | 0.132062275 | -11.4873 | 1.53E-30 | 5.88E-29 | DOWN |
| GNAL | 796.4619688 | -1.431400542 | 0.124713733 | -11.4775 | 1.71E-30 | 6.57E-29 | DOWN |
| DNAH14 | 391.5950081 | 1.107299655 | 0.096487971 | 11.47604 | 1.74E-30 | 6.66E-29 | UP |
| CTF1 | 473.8838345 | -1.497983223 | 0.130741097 | -11.4576 | 2.15E-30 | 8.17E-29 | DOWN |
| DAAM2 | 1089.490053 | -1.715756018 | 0.149758723 | -11.4568 | 2.17E-30 | 8.22E-29 | DOWN |
| CCDC69 | 1887.056407 | -1.422483886 | 0.124243057 | -11.4492 | 2.37E-30 | 8.92E-29 | DOWN |
| TPM1 | 31134.42185 | -1.394098406 | 0.121827485 | -11.4432 | 2.54E-30 | 9.54E-29 | DOWN |
| ACSM1 | 4973.252744 | 2.803664531 | 0.245122081 | 11.43783 | 2.71E-30 | 1.01E-28 | UP |
| NACC2 | 2492.977492 | -1.052068867 | 0.091988703 | -11.4369 | 2.73E-30 | 1.02E-28 | DOWN |
| FXYD6 | 1373.459691 | -1.80933726 | 0.158258676 | -11.4328 | 2.87E-30 | 1.06E-28 | DOWN |
| TLCD1 | 918.9559407 | 1.289341114 | 0.11286233 | 11.42402 | 3.17E-30 | 1.17E-28 | UP |
| GDPD1 | 667.8596769 | 1.525774286 | 0.133698056 | 11.41209 | 3.64E-30 | 1.33E-28 | UP |
| F2RL2 | 86.08137951 | 2.326132011 | 0.203874713 | 11.40962 | 3.74E-30 | 1.37E-28 | UP |
| EYA4 | 313.6092933 | -1.677875114 | 0.147214104 | -11.3975 | 4.30E-30 | 1.57E-28 | DOWN |
| NUP210 | 3923.120393 | 1.162924761 | 0.102047793 | 11.39588 | 4.38E-30 | 1.59E-28 | UP |
| AVPI1 | 652.8739565 | -1.248638635 | 0.109672448 | -11.3852 | 4.96E-30 | 1.79E-28 | DOWN |
| SEMA6D | 266.8507593 | -1.659309664 | 0.145795321 | -11.3811 | 5.19E-30 | 1.87E-28 | DOWN |
| PALLD | 13998.14115 | -1.536280405 | 0.135082865 | -11.3729 | 5.71E-30 | 2.04E-28 | DOWN |
| PCP4L1 | 193.5506932 | -2.22108349 | 0.195332347 | -11.3708 | 5.85E-30 | 2.08E-28 | DOWN |
| BICD1 | 2294.845349 | 1.595489569 | 0.140354385 | 11.36758 | 6.06E-30 | 2.15E-28 | UP |
| AMH | 119.9040159 | 3.188243823 | 0.280469795 | 11.36751 | 6.07E-30 | 2.15E-28 | UP |
| SLC7A11 | 724.9433956 | 1.810641813 | 0.159348563 | 11.36277 | 6.41E-30 | 2.26E-28 | UP |
| WFDC2 | 2528.420171 | -2.424728887 | 0.213425154 | -11.361 | 6.54E-30 | 2.30E-28 | DOWN |
| SKA3 | 128.176584 | 1.96076263 | 0.172756587 | 11.34986 | 7.43E-30 | 2.61E-28 | UP |
| HJURP | 253.9519829 | 2.205006077 | 0.194416412 | 11.34167 | 8.16E-30 | 2.86E-28 | UP |
| TSPAN19 | 101.2821408 | 3.478281809 | 0.306787078 | 11.33777 | 8.53E-30 | 2.98E-28 | UP |
| JPH4 | 772.1706043 | -2.162938403 | 0.19089701 | -11.3304 | 9.28E-30 | 3.23E-28 | DOWN |
| CERK | 948.2923554 | -1.213114874 | 0.107117599 | -11.3251 | 9.86E-30 | 3.42E-28 | DOWN |
| GPR160 | 5052.279343 | 1.094497324 | 0.096709094 | 11.31742 | 1.08E-29 | 3.73E-28 | UP |
| EPHA2 | 958.5105308 | -2.032765715 | 0.179689867 | -11.3126 | 1.14E-29 | 3.92E-28 | DOWN |

| | | | | | | | |
|---|---|---|---|---|---|---|---|
| L3MBTL4 | 124.9938893 | -1.446040857 | 0.127835463 | -11.3117 | 1.15E-29 | 3.95E-28 | DOWN |
| PAX1 | 79.98178669 | 5.018771054 | 0.444223684 | 11.29785 | 1.34E-29 | 4.61E-28 | UP |
| RASL12 | 1923.744593 | -1.715944971 | 0.152031918 | -11.2867 | 1.53E-29 | 5.20E-28 | DOWN |
| ARHGAP20 | 268.6671707 | -1.653566722 | 0.146547771 | -11.2835 | 1.58E-29 | 5.37E-28 | DOWN |
| HIST3H2A | 2091.814612 | 1.473799802 | 0.130618561 | 11.28323 | 1.59E-29 | 5.37E-28 | UP |
| PRICKLE2 | 823.720984 | -1.371262876 | 0.12157174 | -11.2795 | 1.66E-29 | 5.59E-28 | DOWN |
| DAB2IP | 3207.194784 | -1.027052066 | 0.091073163 | -11.2772 | 1.70E-29 | 5.72E-28 | DOWN |
| MOV10L1 | 163.7796238 | 2.480958622 | 0.220206742 | 11.2665 | 1.92E-29 | 6.43E-28 | UP |
| C19orf48 | 16481.65031 | 1.202752267 | 0.107039493 | 11.23653 | 2.70E-29 | 8.99E-28 | UP |
| RAPGEFL1 | 560.5790281 | -1.438034504 | 0.128016973 | -11.2332 | 2.80E-29 | 9.31E-28 | DOWN |
| ACE | 1346.145644 | -1.386386501 | 0.123452612 | -11.2301 | 2.90E-29 | 9.62E-28 | DOWN |
| KCTD17 | 449.8027383 | -1.107306095 | 0.09862999 | -11.2269 | 3.01E-29 | 9.95E-28 | DOWN |
| FEZ1 | 485.4716236 | -1.239687294 | 0.110497924 | -11.2191 | 3.29E-29 | 1.08E-27 | DOWN |
| KIF9 | 540.4629955 | 1.04977659 | 0.093574369 | 11.21863 | 3.30E-29 | 1.09E-27 | UP |
| CDC20 | 284.8763165 | 1.969318382 | 0.175604984 | 11.21448 | 3.46E-29 | 1.13E-27 | UP |
| ASPHD1 | 432.3796847 | 1.619102754 | 0.144505199 | 11.20446 | 3.88E-29 | 1.26E-27 | UP |
| TMEM139 | 54.10156149 | -1.547182859 | 0.138215448 | -11.194 | 4.36E-29 | 1.41E-27 | DOWN |
| CASKIN1 | 237.6024692 | 1.712084599 | 0.153102274 | 11.18262 | 4.96E-29 | 1.60E-27 | UP |
| CDHR1 | 95.6547408 | -1.914665041 | 0.171271254 | -11.1791 | 5.16E-29 | 1.65E-27 | DOWN |
| CAV2 | 1299.059387 | -1.36836125 | 0.122474924 | -11.1726 | 5.55E-29 | 1.77E-27 | DOWN |
| STAC | 451.6435539 | -2.433582463 | 0.217834956 | -11.1717 | 5.61E-29 | 1.79E-27 | DOWN |
| BUB1B | 206.8472519 | 1.676526117 | 0.15011711 | 11.16812 | 5.84E-29 | 1.86E-27 | UP |
| GPX2 | 275.3009714 | -2.568956536 | 0.230063333 | -11.1663 | 5.96E-29 | 1.89E-27 | DOWN |
| KITLG | 1461.244239 | -1.232267688 | 0.110357735 | -11.1661 | 5.97E-29 | 1.89E-27 | DOWN |
| ZNF30 | 617.7180486 | 1.48209882 | 0.132760084 | 11.16374 | 6.14E-29 | 1.94E-27 | UP |
| ST5 | 2521.076583 | -1.248587013 | 0.111856788 | -11.1624 | 6.23E-29 | 1.96E-27 | DOWN |
| EBF2 | 201.9230611 | 2.41798348 | 0.216636272 | 11.16149 | 6.29E-29 | 1.98E-27 | UP |
| CFL2 | 3583.413053 | -1.361360616 | 0.1219882 | -11.1598 | 6.42E-29 | 2.01E-27 | DOWN |
| EPHB1 | 130.4187195 | -2.115480167 | 0.189613021 | -11.1568 | 6.63E-29 | 2.07E-27 | DOWN |
| ABCC4 | 34887.45126 | 1.653579647 | 0.148279061 | 11.15181 | 7.02E-29 | 2.19E-27 | UP |
| TGFB1I1 | 3163.947514 | -1.588910548 | 0.142553975 | -11.146 | 7.49E-29 | 2.33E-27 | DOWN |
| BHMT2 | 315.5503983 | -1.476692598 | 0.132553495 | -11.1404 | 7.98E-29 | 2.47E-27 | DOWN |
| MCC | 1591.107636 | -1.393902939 | 0.125205893 | -11.1329 | 8.68E-29 | 2.68E-27 | DOWN |
| ADAM2 | 224.0980559 | 3.888350899 | 0.349351551 | 11.1302 | 8.94E-29 | 2.76E-27 | UP |
| ALDH1A2 | 1336.497199 | -1.691619104 | 0.152112763 | -11.1208 | 9.94E-29 | 3.05E-27 | DOWN |
| KIT | 506.4135962 | -1.671674438 | 0.150378886 | -11.1164 | 1.04E-28 | 3.19E-27 | DOWN |
| TMEM132A | 3243.787985 | 1.445420377 | 0.130176876 | 11.10351 | 1.21E-28 | 3.68E-27 | UP |
| SERPINF2 | 1037.99547 | -1.703582169 | 0.153475659 | -11.1 | 1.25E-28 | 3.82E-27 | DOWN |
| CCDC40 | 554.2943419 | 1.049345575 | 0.094610942 | 11.09117 | 1.38E-28 | 4.21E-27 | UP |
| PLCD3 | 1260.21961 | -1.200410227 | 0.108347588 | -11.0793 | 1.58E-28 | 4.77E-27 | DOWN |
| VCL | 11365.81124 | -1.432624486 | 0.129327038 | -11.0775 | 1.61E-28 | 4.85E-27 | DOWN |
| TCN2 | 882.230337 | -1.038411767 | 0.093752445 | -11.0761 | 1.64E-28 | 4.91E-27 | DOWN |
| FERMT2 | 3388.92518 | -1.281478971 | 0.11569799 | -11.0761 | 1.64E-28 | 4.91E-27 | DOWN |
| HSPA4L | 448.14311 | -1.359138693 | 0.122773372 | -11.0703 | 1.75E-28 | 5.21E-27 | DOWN |
| HPX | 165.2656591 | 1.671339266 | 0.151028072 | 11.06641 | 1.83E-28 | 5.43E-27 | UP |
| RBMS3 | 878.4098227 | -1.273040022 | 0.115061012 | -11.064 | 1.87E-28 | 5.55E-27 | DOWN |
| CORO1C | 3836.436095 | -1.016781422 | 0.09198395 | -11.0539 | 2.10E-28 | 6.20E-27 | DOWN |
| ANP32E | 1349.230492 | -1.060978243 | 0.095996122 | -11.0523 | 2.14E-28 | 6.30E-27 | DOWN |
| TMTC4 | 3090.344845 | 1.04339313 | 0.094447891 | 11.04729 | 2.26E-28 | 6.65E-27 | UP |
| MR1 | 1185.797637 | -1.152009901 | 0.104340281 | -11.0409 | 2.43E-28 | 7.12E-27 | DOWN |
| PDZRN4 | 601.0217607 | -1.687768427 | 0.152875769 | -11.0401 | 2.45E-28 | 7.17E-27 | DOWN |
| GSC | 105.0881123 | 1.698347721 | 0.153944659 | 11.0322 | 2.67E-28 | 7.78E-27 | UP |
| TARBP1 | 2597.227963 | 1.038397502 | 0.094178024 | 11.0259 | 2.87E-28 | 8.29E-27 | UP |
| ACSF2 | 830.0904659 | -1.486349611 | 0.135025499 | -11.0079 | 3.50E-28 | 1.01E-26 | DOWN |
| DEFB132 | 219.1944782 | -2.230050028 | 0.202599827 | -11.0072 | 3.53E-28 | 1.01E-26 | DOWN |
| EPCAM | 15438.58873 | 1.053890687 | 0.095870032 | 10.99291 | 4.13E-28 | 1.18E-26 | UP |
| ADAT2 | 441.8802907 | 1.212138448 | 0.110446094 | 10.97493 | 5.04E-28 | 1.43E-26 | UP |
| PIK3R1 | 4169.392453 | -1.009768505 | 0.092032058 | -10.9719 | 5.22E-28 | 1.48E-26 | DOWN |
| RNF157 | 1696.575824 | 2.462726985 | 0.224532005 | 10.96827 | 5.43E-28 | 1.54E-26 | UP |
| CCDC8 | 545.4769449 | -1.464183167 | 0.133543344 | -10.9641 | 5.69E-28 | 1.60E-26 | DOWN |
| QRICH2 | 350.4386434 | 1.137503966 | 0.103754093 | 10.96346 | 5.73E-28 | 1.61E-26 | UP |
| GAL | 171.7212594 | 2.355108972 | 0.214991703 | 10.95442 | 6.33E-28 | 1.77E-26 | UP |
| CYP4F8 | 2894.361754 | -4.04208879 | 0.369194257 | -10.9484 | 6.76E-28 | 1.89E-26 | DOWN |

| | | | | | | | |
|---|---|---|---|---|---|---|---|
| UNC5A | 841.5784782 | 3.520699285 | 0.32163683 | 10.94619 | 6.93E-28 | 1.93E-26 | UP |
| PRKCB | 719.8519778 | -1.784903412 | 0.163072086 | -10.9455 | 6.98E-28 | 1.94E-26 | DOWN |
| MAP1A | 1045.666634 | -1.568589229 | 0.143450794 | -10.9347 | 7.87E-28 | 2.18E-26 | DOWN |
| KANK2 | 7512.52928 | -1.410583661 | 0.129008225 | -10.9341 | 7.92E-28 | 2.19E-26 | DOWN |
| GFRA2 | 70.95500069 | -1.602166617 | 0.14654019 | -10.9333 | 7.99E-28 | 2.20E-26 | DOWN |
| COL4A6 | 1039.880431 | -1.882251049 | 0.172368512 | -10.9199 | 9.26E-28 | 2.53E-26 | DOWN |
| CORO2B | 111.7724172 | -1.254120965 | 0.114918949 | -10.9131 | 9.98E-28 | 2.72E-26 | DOWN |
| FAM107B | 4878.708184 | -1.216690523 | 0.111496426 | -10.9124 | 1.01E-27 | 2.74E-26 | DOWN |
| A4GALT | 1717.839121 | -1.04867748 | 0.096111506 | -10.9111 | 1.02E-27 | 2.77E-26 | DOWN |
| GAS6 | 7029.88821 | -1.249327525 | 0.114677139 | -10.8943 | 1.23E-27 | 3.33E-26 | DOWN |
| PODXL2 | 7599.22759 | 1.167930807 | 0.107253646 | 10.88943 | 1.29E-27 | 3.50E-26 | UP |
| CACNA1D | 3057.156964 | 2.199536138 | 0.202029513 | 10.8872 | 1.33E-27 | 3.58E-26 | UP |
| TWIST1 | 564.0831138 | 2.202080945 | 0.202586911 | 10.86981 | 1.61E-27 | 4.29E-26 | UP |
| TINAGL1 | 2055.102167 | -1.347641006 | 0.123999244 | -10.8681 | 1.64E-27 | 4.36E-26 | DOWN |
| MAMDC2 | 332.4741816 | -1.489211259 | 0.137074987 | -10.8642 | 1.71E-27 | 4.54E-26 | DOWN |
| SEMA3B | 1383.867655 | -1.090979805 | 0.100479682 | -10.8577 | 1.83E-27 | 4.86E-26 | DOWN |
| CEND1 | 109.9092056 | -1.835610696 | 0.169100388 | -10.8552 | 1.88E-27 | 4.99E-26 | DOWN |
| ARHGAP23 | 1804.76141 | -1.548923441 | 0.142919612 | -10.8377 | 2.28E-27 | 6.00E-26 | DOWN |
| FABP5 | 2311.204804 | 3.104943166 | 0.286503664 | 10.83736 | 2.29E-27 | 6.01E-26 | UP |
| SPINK1 | 680.8422008 | 4.374773526 | 0.404219609 | 10.82276 | 2.69E-27 | 7.04E-26 | UP |
| TMEM184A | 3495.16836 | 1.014766091 | 0.093861651 | 10.8113 | 3.04E-27 | 7.94E-26 | UP |
| ATG9B | 168.0872311 | 1.678061565 | 0.155269259 | 10.80743 | 3.17E-27 | 8.27E-26 | UP |
| CHRDL1 | 4798.505325 | -1.955392594 | 0.180947874 | -10.8064 | 3.21E-27 | 8.34E-26 | DOWN |
| RALGAPA2 | 9102.369047 | 1.215138527 | 0.112528285 | 10.79852 | 3.50E-27 | 9.07E-26 | UP |
| DBNDD1 | 2036.097528 | 1.178753703 | 0.109174379 | 10.79698 | 3.56E-27 | 9.21E-26 | UP |
| SGCB | 2201.172152 | -1.013790373 | 0.094060756 | -10.778 | 4.37E-27 | 1.12E-25 | DOWN |
| ADAMTS5 | 381.6987558 | -1.758835753 | 0.163214952 | -10.7762 | 4.46E-27 | 1.14E-25 | DOWN |
| KIAA0087 | 60.95804218 | 4.022285338 | 0.373404711 | 10.77192 | 4.67E-27 | 1.20E-25 | UP |
| NPR2 | 577.6058762 | -1.050312008 | 0.097690731 | -10.7514 | 5.84E-27 | 1.48E-25 | DOWN |
| CLIP4 | 1071.019588 | -1.088346593 | 0.101236574 | -10.7505 | 5.89E-27 | 1.50E-25 | DOWN |
| CRABP2 | 1104.755838 | -2.021251344 | 0.18804648 | -10.7487 | 6.01E-27 | 1.52E-25 | DOWN |
| HSPB8 | 5338.489127 | -1.919411989 | 0.178660055 | -10.7434 | 6.37E-27 | 1.61E-25 | DOWN |
| MUC15 | 125.3899096 | -2.632088689 | 0.245025819 | -10.7421 | 6.46E-27 | 1.62E-25 | DOWN |
| RAB38 | 87.60680269 | -1.378297033 | 0.128334533 | -10.7399 | 6.61E-27 | 1.66E-25 | DOWN |
| CNTFR | 194.9742063 | -2.041056479 | 0.190056284 | -10.7392 | 6.66E-27 | 1.67E-25 | DOWN |
| DIXDC1 | 2371.433906 | -1.086998403 | 0.101263372 | -10.7344 | 7.02E-27 | 1.75E-25 | DOWN |
| ANXA6 | 7115.632194 | -1.256290102 | 0.117042869 | -10.7336 | 7.08E-27 | 1.77E-25 | DOWN |
| ELL3 | 119.2083471 | 1.509731508 | 0.14069553 | 10.73049 | 7.32E-27 | 1.82E-25 | UP |
| ASB2 | 866.9476825 | -1.622919395 | 0.151325764 | -10.7247 | 7.80E-27 | 1.93E-25 | DOWN |
| EFEMP2 | 2619.362756 | -1.290854268 | 0.120379393 | -10.7232 | 7.92E-27 | 1.96E-25 | DOWN |
| NECAB1 | 287.1970313 | -1.608001607 | 0.150073003 | -10.7148 | 8.67E-27 | 2.14E-25 | DOWN |
| UBXN10 | 266.3234758 | -1.778551671 | 0.166059719 | -10.7103 | 9.11E-27 | 2.25E-25 | DOWN |
| ZNF853 | 879.2426064 | -1.058597233 | 0.098897542 | -10.704 | 9.75E-27 | 2.40E-25 | DOWN |
| FAM107A | 2486.11254 | -1.656512426 | 0.154845569 | -10.6978 | 1.04E-26 | 2.55E-25 | DOWN |
| HOXB4 | 66.60701198 | -1.999084069 | 0.186885969 | -10.6968 | 1.05E-26 | 2.57E-25 | DOWN |
| SMTN | 12234.45954 | -1.514474266 | 0.141644633 | -10.6921 | 1.11E-26 | 2.70E-25 | DOWN |
| MSLN | 285.4399165 | -2.861498521 | 0.267686953 | -10.6897 | 1.14E-26 | 2.76E-25 | DOWN |
| GPR161 | 1158.78715 | -1.295026983 | 0.121165179 | -10.6881 | 1.16E-26 | 2.81E-25 | DOWN |
| CDC25C | 73.57272806 | 2.061930838 | 0.193076796 | 10.67933 | 1.27E-26 | 3.08E-25 | UP |
| ACOX2 | 407.3005634 | -1.567107046 | 0.146871212 | -10.6699 | 1.41E-26 | 3.39E-25 | DOWN |
| NHS | 330.9813098 | -1.242174813 | 0.116470117 | -10.6652 | 1.48E-26 | 3.55E-25 | DOWN |
| MCOLN2 | 253.456376 | 1.883906819 | 0.176794057 | 10.65594 | 1.64E-26 | 3.89E-25 | UP |
| MYLK | 39255.2491 | -1.857110018 | 0.174296999 | -10.6549 | 1.65E-26 | 3.93E-25 | DOWN |
| RBPMS2 | 744.3795178 | -1.29582832 | 0.121629643 | -10.6539 | 1.67E-26 | 3.96E-25 | DOWN |
| C9orf163 | 50.07423155 | 1.891845621 | 0.177650438 | 10.64926 | 1.76E-26 | 4.14E-25 | UP |
| CAPG | 2439.6551 | -1.375224972 | 0.129244686 | -10.6405 | 1.93E-26 | 4.53E-25 | DOWN |
| MTG1 | 405.1874358 | 1.06895949 | 0.100505453 | 10.63584 | 2.03E-26 | 4.76E-25 | UP |
| DLX1 | 1963.738816 | 2.913562869 | 0.274126806 | 10.62852 | 2.20E-26 | 5.14E-25 | UP |
| RIMKLA | 629.650421 | 1.117678815 | 0.105286536 | 10.61559 | 2.52E-26 | 5.89E-25 | UP |
| TDRD1 | 1028.915043 | 3.048062714 | 0.287239583 | 10.61157 | 2.63E-26 | 6.14E-25 | UP |
| ANXA2P2 | 108.5522589 | -1.304786013 | 0.123028415 | -10.6056 | 2.81E-26 | 6.52E-25 | DOWN |
| CPT1B | 106.3444313 | 1.614820683 | 0.15231364 | 10.60194 | 2.92E-26 | 6.77E-25 | UP |
| AHNAK2 | 1571.104362 | -1.788107916 | 0.168772334 | -10.5948 | 3.15E-26 | 7.28E-25 | DOWN |

| | | | | | | | |
|---|---|---|---|---|---|---|---|
| MSRB3 | 4023.891182 | -1.524031787 | 0.143940373 | -10.5879 | 3.39E-26 | 7.81E-25 | DOWN |
| PNCK | 638.2177931 | -1.541516076 | 0.145637759 | -10.5846 | 3.51E-26 | 8.08E-25 | DOWN |
| LUZP2 | 2116.58672 | 2.073539523 | 0.195977183 | 10.58052 | 3.67E-26 | 8.42E-25 | UP |
| CCDC110 | 292.0725553 | 1.289251128 | 0.121864953 | 10.57934 | 3.72E-26 | 8.51E-25 | UP |
| SLC16A5 | 520.7282753 | -1.748592522 | 0.165411299 | -10.5712 | 4.05E-26 | 9.25E-25 | DOWN |
| CHST11 | 628.2183146 | -1.208750957 | 0.114458567 | -10.5606 | 4.54E-26 | 1.03E-24 | DOWN |
| PDLIM7 | 6021.933992 | -1.390562792 | 0.131755142 | -10.5541 | 4.86E-26 | 1.10E-24 | DOWN |
| PRKG1 | 518.9008926 | -1.475210465 | 0.139775791 | -10.5541 | 4.86E-26 | 1.10E-24 | DOWN |
| CDC42EP3 | 5840.674021 | -1.201667486 | 0.113906882 | -10.5496 | 5.10E-26 | 1.15E-24 | DOWN |
| PEG3 | 421.5157847 | -1.097571737 | 0.104045404 | -10.549 | 5.14E-26 | 1.16E-24 | DOWN |
| CENPM | 233.0732364 | 1.773962326 | 0.168173034 | 10.54844 | 5.17E-26 | 1.16E-24 | UP |
| POPDC2 | 755.9050867 | -1.473893515 | 0.139743445 | -10.5471 | 5.24E-26 | 1.18E-24 | DOWN |
| PVT1 | 670.4592364 | 1.329385623 | 0.126098074 | 10.54247 | 5.50E-26 | 1.23E-24 | UP |
| PPP2R2B | 73.88853274 | -1.471337088 | 0.139671409 | -10.5343 | 6.00E-26 | 1.34E-24 | DOWN |
| RPL22L1 | 2802.304812 | 1.477522446 | 0.140540661 | 10.51313 | 7.52E-26 | 1.67E-24 | UP |
| SLCO1A2 | 421.7115119 | 3.417720418 | 0.325138625 | 10.51158 | 7.64E-26 | 1.69E-24 | UP |
| MXD3 | 275.7694585 | 1.402522118 | 0.133529068 | 10.5035 | 8.32E-26 | 1.83E-24 | UP |
| ASPA | 161.0392871 | -1.925947733 | 0.183368084 | -10.5032 | 8.35E-26 | 1.84E-24 | DOWN |
| CACHD1 | 733.5737723 | -1.275274844 | 0.121441973 | -10.5011 | 8.54E-26 | 1.87E-24 | DOWN |
| DUOXA1 | 274.3221327 | -2.203641906 | 0.210043616 | -10.4914 | 9.47E-26 | 2.07E-24 | DOWN |
| RGS10 | 4508.943593 | 1.115333365 | 0.10633391 | 10.48897 | 9.71E-26 | 2.12E-24 | UP |
| TLR2 | 333.1326523 | -1.330349702 | 0.12695642 | -10.4788 | 1.08E-25 | 2.36E-24 | DOWN |
| CDCA8 | 222.6244845 | 1.394842473 | 0.133133536 | 10.47702 | 1.10E-25 | 2.40E-24 | UP |
| HES4 | 933.8419558 | 1.419444039 | 0.135545275 | 10.4721 | 1.16E-25 | 2.52E-24 | UP |
| ANKRD34B | 440.1414197 | 4.379387851 | 0.418312054 | 10.46919 | 1.20E-25 | 2.59E-24 | UP |
| PPP1R3C | 663.5747347 | -1.58088308 | 0.151294286 | -10.4491 | 1.48E-25 | 3.17E-24 | DOWN |
| ANGPTL3 | 87.63793482 | 3.516029947 | 0.336840308 | 10.43827 | 1.66E-25 | 3.54E-24 | UP |
| NT5E | 748.2114554 | -1.260977476 | 0.120865591 | -10.4329 | 1.75E-25 | 3.74E-24 | DOWN |
| DPYSL3 | 12039.59391 | -1.29033223 | 0.123733534 | -10.4283 | 1.84E-25 | 3.92E-24 | DOWN |
| TRIP6 | 2567.495566 | -1.183410047 | 0.113521634 | -10.4245 | 1.92E-25 | 4.06E-24 | DOWN |
| ARHGDIG | 208.1902075 | 2.566078977 | 0.246161966 | 10.42435 | 1.92E-25 | 4.06E-24 | UP |
| RGS11 | 976.7996926 | 1.662862716 | 0.159536481 | 10.42309 | 1.95E-25 | 4.11E-24 | UP |
| FGFRL1 | 5235.553737 | 1.360657541 | 0.130601458 | 10.41839 | 2.04E-25 | 4.31E-24 | UP |
| RND2 | 198.4299912 | -1.442426949 | 0.138479429 | -10.4162 | 2.09E-25 | 4.41E-24 | DOWN |
| NAV2 | 859.4626424 | -1.414116308 | 0.135789952 | -10.414 | 2.14E-25 | 4.50E-24 | DOWN |
| LRGUK | 57.39838134 | 1.698115657 | 0.163173094 | 10.40684 | 2.31E-25 | 4.83E-24 | UP |
| CDH23 | 157.7014241 | -1.447510376 | 0.139209032 | -10.3981 | 2.53E-25 | 5.28E-24 | DOWN |
| SLC10A5 | 105.8511178 | 1.591768562 | 0.153152124 | 10.39338 | 2.66E-25 | 5.53E-24 | UP |
| FGF10 | 116.1941543 | -1.93017904 | 0.185796047 | -10.3887 | 2.79E-25 | 5.80E-24 | DOWN |
| LIMS2 | 4057.735229 | -1.49592837 | 0.144024998 | -10.3866 | 2.85E-25 | 5.92E-24 | DOWN |
| PDE1C | 96.47466242 | -2.23585715 | 0.215759957 | -10.3627 | 3.66E-25 | 7.55E-24 | DOWN |
| ZNF90 | 92.3828532 | -1.611439173 | 0.1555042 | -10.3627 | 3.67E-25 | 7.55E-24 | DOWN |
| FAM47E | 308.6592009 | 1.011492666 | 0.097640177 | 10.35939 | 3.79E-25 | 7.79E-24 | UP |
| IQGAP3 | 233.9382295 | 1.955047055 | 0.188769619 | 10.35679 | 3.90E-25 | 7.99E-24 | UP |
| ITGA2 | 1350.382177 | -1.300457853 | 0.125610647 | -10.3531 | 4.05E-25 | 8.30E-24 | DOWN |
| CLIC4 | 9464.451582 | -1.023136375 | 0.098840806 | -10.3514 | 4.13E-25 | 8.44E-24 | DOWN |
| CDCA5 | 297.0702074 | 1.61224335 | 0.155765882 | 10.35043 | 4.17E-25 | 8.50E-24 | UP |
| POU3F3 | 171.2643087 | -7.846354518 | 0.758124336 | -10.3497 | 4.20E-25 | 8.56E-24 | DOWN |
| SH3PXD2B | 1412.176313 | -1.167366765 | 0.11281455 | -10.3477 | 4.29E-25 | 8.73E-24 | DOWN |
| JAZF1 | 1219.011927 | -1.262080964 | 0.122065352 | -10.3394 | 4.68E-25 | 9.48E-24 | DOWN |
| GPC2 | 69.66154052 | 1.738645167 | 0.168228264 | 10.33504 | 4.89E-25 | 9.91E-24 | UP |
| PLAG1 | 127.1171948 | -1.423342492 | 0.137726476 | -10.3346 | 4.92E-25 | 9.94E-24 | DOWN |
| OTX1 | 412.1553896 | 2.156189548 | 0.20873717 | 10.32969 | 5.17E-25 | 1.04E-23 | UP |
| MSI1 | 880.8848055 | 1.07103971 | 0.103721109 | 10.32615 | 5.37E-25 | 1.08E-23 | UP |
| PPARGC1B | 330.1861001 | -1.183304157 | 0.114594945 | -10.326 | 5.38E-25 | 1.08E-23 | DOWN |
| SPON1 | 1476.826125 | -1.658966217 | 0.160682828 | -10.3245 | 5.46E-25 | 1.10E-23 | DOWN |
| NRG2 | 107.9133788 | -1.613804996 | 0.156347488 | -10.3219 | 5.61E-25 | 1.12E-23 | DOWN |
| TEAD1 | 2923.946217 | -1.017852631 | 0.098670615 | -10.3157 | 5.99E-25 | 1.19E-23 | DOWN |
| ZNF516 | 1081.636416 | -1.292987337 | 0.125431027 | -10.3084 | 6.46E-25 | 1.28E-23 | DOWN |
| NSUN5P1 | 755.2865642 | 1.305333332 | 0.126687461 | 10.30357 | 6.79E-25 | 1.35E-23 | UP |
| LMOD1 | 10989.99663 | -1.711990428 | 0.166190499 | -10.3014 | 6.95E-25 | 1.38E-23 | DOWN |
| CDK5R1 | 210.2354098 | 1.346139716 | 0.130781369 | 10.29305 | 7.57E-25 | 1.50E-23 | UP |
| ATP2B4 | 6147.649774 | -1.48492033 | 0.144410917 | -10.2826 | 8.44E-25 | 1.66E-23 | DOWN |

| | | | | | | | | |
|---|---|---|---|---|---|---|---|---|
| C14orf132 | 1010.215111 | -1.312893472 | 0.127849204 | -10.2691 | 9.71E-25 | 1.90E-23 | DOWN |
| TRPC6 | 141.0925353 | -1.379173929 | 0.134304658 | -10.269 | 9.72E-25 | 1.90E-23 | DOWN |
| GCNT1 | 5012.273518 | 1.70753504 | 0.166378812 | 10.26294 | 1.04E-24 | 2.02E-23 | UP |
| GNG13 | 63.75187146 | 2.895140997 | 0.282207399 | 10.25891 | 1.08E-24 | 2.10E-23 | UP |
| SLC18A2 | 154.8972173 | -2.488346215 | 0.242654934 | -10.2547 | 1.13E-24 | 2.19E-23 | DOWN |
| SEMG2 | 9187.547392 | -6.308034736 | 0.615286264 | -10.2522 | 1.16E-24 | 2.24E-23 | DOWN |
| CLIP2 | 1381.250499 | -1.026374861 | 0.100144991 | -10.2489 | 1.20E-24 | 2.31E-23 | DOWN |
| SYNM | 15090.08126 | -1.822523356 | 0.177946331 | -10.242 | 1.29E-24 | 2.48E-23 | DOWN |
| BIK | 1343.148744 | 1.063253654 | 0.103945334 | 10.22897 | 1.47E-24 | 2.82E-23 | UP |
| PKIB | 1143.829317 | 1.753532874 | 0.171446415 | 10.22788 | 1.49E-24 | 2.85E-23 | UP |
| MAK | 128.4463424 | 1.768727841 | 0.172991161 | 10.22438 | 1.54E-24 | 2.95E-23 | UP |
| PLIN4 | 1668.267222 | -1.498367175 | 0.14664348 | -10.2178 | 1.65E-24 | 3.15E-23 | DOWN |
| GPR89A | 291.4451835 | 1.04455348 | 0.10236291 | 10.20441 | 1.89E-24 | 3.60E-23 | UP |
| CLIP3 | 1675.207539 | -1.529661295 | 0.150041027 | -10.195 | 2.09E-24 | 3.95E-23 | DOWN |
| FBXO17 | 473.976817 | -1.375172252 | 0.134977841 | -10.1881 | 2.24E-24 | 4.22E-23 | DOWN |
| HOXD13 | 403.2870591 | -1.823594467 | 0.179032385 | -10.1858 | 2.29E-24 | 4.31E-23 | DOWN |
| RPGRIP1 | 68.2995985 | 1.395811212 | 0.137049097 | 10.18475 | 2.32E-24 | 4.36E-23 | UP |
| F12 | 210.3613248 | 1.760913239 | 0.172904132 | 10.18433 | 2.33E-24 | 4.37E-23 | UP |
| NKX6-1 | 57.19697546 | 4.193296456 | 0.4119004 | 10.18037 | 2.43E-24 | 4.54E-23 | UP |
| MAMSTR | 106.4278756 | -1.033266243 | 0.101517488 | -10.1782 | 2.48E-24 | 4.64E-23 | DOWN |
| STOX2 | 232.7638054 | -1.426162638 | 0.140178663 | -10.1739 | 2.59E-24 | 4.83E-23 | DOWN |
| TROAP | 149.1228164 | 1.874997749 | 0.184316774 | 10.17269 | 2.63E-24 | 4.89E-23 | UP |
| WNT10A | 115.4538763 | -1.533680723 | 0.150819512 | -10.169 | 2.73E-24 | 5.07E-23 | DOWN |
| FOXB2 | 47.75128959 | 4.898150755 | 0.482185109 | 10.15824 | 3.05E-24 | 5.65E-23 | UP |
| CALD1 | 36477.89224 | -1.227512618 | 0.120900163 | -10.1531 | 3.21E-24 | 5.93E-23 | DOWN |
| OR51E2 | 32457.52727 | 2.398426532 | 0.236240331 | 10.15249 | 3.23E-24 | 5.95E-23 | UP |
| STARD8 | 494.6343731 | -1.310247522 | 0.129079774 | -10.1507 | 3.29E-24 | 6.06E-23 | DOWN |
| ERBB4 | 169.375629 | -1.822872083 | 0.179678249 | -10.1452 | 3.48E-24 | 6.39E-23 | DOWN |
| DMD | 1950.357773 | -1.530331848 | 0.150876674 | -10.1429 | 3.56E-24 | 6.52E-23 | DOWN |
| CELSR2 | 1647.892279 | -1.102107325 | 0.108712992 | -10.1378 | 3.76E-24 | 6.87E-23 | DOWN |
| TMEM200B | 577.5093174 | -1.44693145 | 0.142749414 | -10.1362 | 3.82E-24 | 6.97E-23 | DOWN |
| MXRA7 | 4690.042319 | -1.267012732 | 0.1250286 | -10.1338 | 3.91E-24 | 7.13E-23 | DOWN |
| YJEFN3 | 304.5799123 | 1.773700594 | 0.175047673 | 10.13267 | 3.96E-24 | 7.20E-23 | UP |
| CYP4B1 | 739.8543856 | -2.201126283 | 0.217410849 | -10.1243 | 4.31E-24 | 7.84E-23 | DOWN |
| MBNL2 | 2641.649551 | -1.023685716 | 0.101145966 | -10.1209 | 4.46E-24 | 8.10E-23 | DOWN |
| ADCY5 | 1623.958648 | -1.454409467 | 0.143707174 | -10.1206 | 4.47E-24 | 8.11E-23 | DOWN |
| NACAD | 280.5267421 | -1.201983575 | 0.118796913 | -10.118 | 4.60E-24 | 8.32E-23 | DOWN |
| C3orf70 | 734.1371149 | -1.340226542 | 0.13249445 | -10.1153 | 4.72E-24 | 8.54E-23 | DOWN |
| HIST1H1E | 118.5120829 | 2.431152334 | 0.24039652 | 10.11309 | 4.83E-24 | 8.73E-23 | UP |
| RAB6C | 181.1852078 | 1.291374763 | 0.127775819 | 10.10657 | 5.17E-24 | 9.29E-23 | UP |
| GMDS | 3337.718964 | 1.173580368 | 0.116155134 | 10.10356 | 5.33E-24 | 9.56E-23 | UP |
| RERG | 716.0299262 | -1.097913352 | 0.108764751 | -10.0944 | 5.85E-24 | 1.05E-22 | DOWN |
| ZP3 | 464.1847793 | 1.470352624 | 0.145721451 | 10.09016 | 6.11E-24 | 1.09E-22 | UP |
| CABP4 | 309.0350323 | 1.328822637 | 0.131698216 | 10.08991 | 6.12E-24 | 1.09E-22 | UP |
| ELAVL2 | 215.9247289 | 2.464035516 | 0.244415943 | 10.08132 | 6.68E-24 | 1.19E-22 | UP |
| DIAPH3 | 87.75595882 | 1.700038542 | 0.168761434 | 10.07362 | 7.23E-24 | 1.28E-22 | UP |
| COL21A1 | 220.341716 | -1.22962191 | 0.122105749 | -10.0701 | 7.49E-24 | 1.32E-22 | DOWN |
| SLC22A10 | 58.09935735 | 3.225100435 | 0.320482264 | 10.06327 | 8.03E-24 | 1.42E-22 | UP |
| CSRNP3 | 232.2008381 | -1.372360008 | 0.136550216 | -10.0502 | 9.17E-24 | 1.61E-22 | DOWN |
| CENPF | 577.0957308 | 1.723685223 | 0.171542736 | 10.04814 | 9.36E-24 | 1.64E-22 | UP |
| FOXM1 | 367.5618394 | 1.613937369 | 0.160695815 | 10.04343 | 9.82E-24 | 1.72E-22 | UP |
| ST6GALNAC2 | 426.7991626 | -1.369318288 | 0.136356733 | -10.0422 | 9.95E-24 | 1.74E-22 | DOWN |
| C10orf95 | 104.3619457 | 1.318569117 | 0.131404106 | 10.03446 | 1.08E-23 | 1.87E-22 | UP |
| KLHL35 | 149.6656916 | 1.617011608 | 0.161167026 | 10.03314 | 1.09E-23 | 1.89E-22 | UP |
| EFEMP1 | 3062.48361 | -1.402411503 | 0.139779515 | -10.033 | 1.09E-23 | 1.89E-22 | DOWN |
| MYOF | 5305.404875 | -1.381363519 | 0.137729892 | -10.0295 | 1.13E-23 | 1.96E-22 | DOWN |
| PGM5 | 8103.372751 | -1.844970421 | 0.183986761 | -10.0277 | 1.15E-23 | 1.99E-22 | DOWN |
| MYOCD | 1117.52672 | -1.744190161 | 0.173948149 | -10.0271 | 1.16E-23 | 2.00E-22 | DOWN |
| PPP1R14A | 1027.831486 | -1.523884072 | 0.152063933 | -10.0213 | 1.23E-23 | 2.11E-22 | DOWN |
| ACACB | 1319.074093 | -1.057155013 | 0.105516578 | -10.0189 | 1.26E-23 | 2.16E-22 | DOWN |
| TACC1 | 7936.121959 | -1.076971099 | 0.107511108 | -10.0173 | 1.28E-23 | 2.19E-22 | DOWN |
| TRIM6 | 126.3856185 | -1.110237454 | 0.110877825 | -10.0132 | 1.33E-23 | 2.28E-22 | DOWN |
| SHCBP1 | 100.9281239 | 1.700595798 | 0.169850996 | 10.01228 | 1.35E-23 | 2.29E-22 | UP |

| | | | | | | | |
|---|---|---|---|---|---|---|---|
| C14orf39 | 72.81243489 | 1.691581501 | 0.169034175 | 10.00733 | 1.42E-23 | 2.40E-22 | UP |
| CSRP1 | 46656.42531 | -1.374034726 | 0.137405986 | -9.99982 | 1.53E-23 | 2.59E-22 | DOWN |
| POLN | 263.1351866 | 1.184130314 | 0.118468434 | 9.995323 | 1.60E-23 | 2.70E-22 | UP |
| SLC34A2 | 588.3149077 | -3.211520411 | 0.321549934 | -9.98763 | 1.73E-23 | 2.91E-22 | DOWN |
| TGM3 | 774.2776884 | 2.308677818 | 0.23118506 | 9.986276 | 1.75E-23 | 2.95E-22 | UP |
| LPAR1 | 942.4964096 | -1.141065807 | 0.114291345 | -9.98383 | 1.79E-23 | 3.01E-22 | DOWN |
| DUOX2 | 307.8278038 | -2.657855385 | 0.266253419 | -9.98243 | 1.82E-23 | 3.05E-22 | DOWN |
| ZNF577 | 1458.666842 | 1.006143822 | 0.100819785 | 9.979627 | 1.87E-23 | 3.14E-22 | UP |
| ACSL4 | 1380.506984 | -1.021273907 | 0.102369764 | -9.97632 | 1.94E-23 | 3.24E-22 | DOWN |
| RNASE1 | 1750.054303 | -1.188733028 | 0.119178525 | -9.97439 | 1.97E-23 | 3.30E-22 | DOWN |
| S100A6 | 8472.921546 | -1.408418027 | 0.141219852 | -9.97323 | 2.00E-23 | 3.33E-22 | DOWN |
| PGR | 576.0863164 | -1.462283771 | 0.146642549 | -9.97176 | 2.03E-23 | 3.38E-22 | DOWN |
| QSOX1 | 4873.735819 | -1.184149904 | 0.118758478 | -9.97108 | 2.04E-23 | 3.40E-22 | DOWN |
| RARG | 1001.737665 | -1.059272335 | 0.106263173 | -9.96839 | 2.10E-23 | 3.49E-22 | DOWN |
| CENPA | 78.72865064 | 2.120185264 | 0.21269781 | 9.968063 | 2.10E-23 | 3.49E-22 | UP |
| SPC25 | 80.58876884 | 1.77827203 | 0.178667645 | 9.952961 | 2.45E-23 | 4.05E-22 | UP |
| GRIK5 | 440.5767247 | -1.0832921 | 0.108994923 | -9.93892 | 2.82E-23 | 4.65E-22 | DOWN |
| KCNMB1 | 2272.750536 | -1.585068295 | 0.159543544 | -9.93502 | 2.93E-23 | 4.82E-22 | DOWN |
| BEND4 | 2799.590641 | 1.72440479 | 0.173760041 | 9.924058 | 3.27E-23 | 5.37E-22 | UP |
| LYNX1 | 1379.095993 | -1.06966869 | 0.107793609 | -9.9233 | 3.30E-23 | 5.40E-22 | DOWN |
| DNAJB4 | 875.1055429 | -1.195857511 | 0.120591002 | -9.91664 | 3.52E-23 | 5.75E-22 | DOWN |
| VWA5A | 1063.806735 | -1.209098104 | 0.121970003 | -9.91308 | 3.65E-23 | 5.95E-22 | DOWN |
| KSR1 | 430.0944873 | -1.132045851 | 0.114203662 | -9.91252 | 3.67E-23 | 5.98E-22 | DOWN |
| KRT16 | 202.8564438 | -2.590082203 | 0.261378873 | -9.9093 | 3.79E-23 | 6.17E-22 | DOWN |
| RBP1 | 636.5784846 | -1.513459119 | 0.152741689 | -9.90862 | 3.82E-23 | 6.20E-22 | DOWN |
| TRIP13 | 237.0996688 | 1.402397848 | 0.141540848 | 9.908079 | 3.84E-23 | 6.23E-22 | UP |
| COL23A1 | 262.6382871 | -1.529187507 | 0.154426638 | -9.90236 | 4.07E-23 | 6.58E-22 | DOWN |
| NIPAL4 | 50.21632664 | -1.822625623 | 0.184075424 | -9.90152 | 4.10E-23 | 6.63E-22 | DOWN |
| KIF18B | 92.62375094 | 2.105670997 | 0.212908706 | 9.890018 | 4.60E-23 | 7.43E-22 | UP |
| SEMG1 | 18283.24877 | -6.659217637 | 0.673377814 | -9.88927 | 4.63E-23 | 7.47E-22 | DOWN |
| CCNB2 | 392.9984284 | 1.416373704 | 0.143257939 | 9.886878 | 4.75E-23 | 7.64E-22 | UP |
| TSLP | 159.7885271 | -1.680521325 | 0.170073987 | -9.88112 | 5.03E-23 | 8.09E-22 | DOWN |
| FBXL22 | 305.482318 | -1.014866161 | 0.102723988 | -9.87954 | 5.11E-23 | 8.20E-22 | DOWN |
| EHD2 | 4163.055212 | -1.290160801 | 0.130615732 | -9.87753 | 5.21E-23 | 8.36E-22 | DOWN |
| GPR89B | 218.0219535 | 1.045380878 | 0.105903062 | 9.871111 | 5.55E-23 | 8.90E-22 | UP |
| SLC47A1 | 174.1788418 | -1.277297529 | 0.129635855 | -9.85296 | 6.66E-23 | 1.06E-21 | DOWN |
| FHOD3 | 1264.441963 | -1.351441118 | 0.137217928 | -9.84887 | 6.93E-23 | 1.10E-21 | DOWN |
| RAP1GAP | 7696.799566 | 1.212750276 | 0.123256471 | 9.839242 | 7.63E-23 | 1.21E-21 | UP |
| HIST3H2BB | 198.291083 | 1.808586566 | 0.183856606 | 9.836941 | 7.80E-23 | 1.24E-21 | UP |
| GLDN | 53.99700793 | -1.85576035 | 0.188905576 | -9.82375 | 8.90E-23 | 1.41E-21 | DOWN |
| EFHD1 | 569.1460797 | -1.303177216 | 0.132702384 | -9.8203 | 9.21E-23 | 1.45E-21 | DOWN |
| PARM1 | 10259.56178 | -1.202691522 | 0.12250813 | -9.81724 | 9.49E-23 | 1.49E-21 | DOWN |
| LGR6 | 264.5580925 | -2.213619676 | 0.225529104 | -9.81523 | 9.68E-23 | 1.52E-21 | DOWN |
| HAGHL | 387.1498444 | 1.708671282 | 0.174188154 | 9.809343 | 1.03E-22 | 1.61E-21 | UP |
| ENO2 | 892.8387729 | -1.028586045 | 0.104863941 | -9.80877 | 1.03E-22 | 1.61E-21 | DOWN |
| SLC43A1 | 4423.609564 | 1.402754371 | 0.143093224 | 9.80308 | 1.09E-22 | 1.70E-21 | UP |
| METTL7A | 5400.766336 | -1.084861735 | 0.110681963 | -9.80161 | 1.11E-22 | 1.72E-21 | DOWN |
| LHX6 | 203.1958522 | 1.087822717 | 0.110999088 | 9.800285 | 1.12E-22 | 1.74E-21 | UP |
| PIPOX | 139.5692462 | -1.104213186 | 0.112730433 | -9.79516 | 1.18E-22 | 1.83E-21 | DOWN |
| PPARGC1A | 299.3670955 | -1.631205936 | 0.166583393 | -9.79213 | 1.22E-22 | 1.88E-21 | DOWN |
| ITGA3 | 3703.545934 | -1.012616272 | 0.103449113 | -9.78854 | 1.26E-22 | 1.95E-21 | DOWN |
| SLC39A2 | 212.0262032 | -3.90351415 | 0.398818002 | -9.78771 | 1.27E-22 | 1.96E-21 | DOWN |
| EMILIN3 | 373.5323276 | -1.362481895 | 0.139312987 | -9.78001 | 1.37E-22 | 2.11E-21 | DOWN |
| FFAR2 | 615.069402 | 1.844477952 | 0.188670555 | 9.776183 | 1.42E-22 | 2.19E-21 | UP |
| JPH2 | 1782.701322 | -1.596244699 | 0.163410633 | -9.7683 | 1.54E-22 | 2.35E-21 | DOWN |
| FGL1 | 152.919231 | 3.217486424 | 0.329450926 | 9.766208 | 1.57E-22 | 2.39E-21 | UP |
| S100A16 | 1305.224663 | -1.267481804 | 0.129808018 | -9.76428 | 1.60E-22 | 2.44E-21 | DOWN |
| KCNMA1 | 4316.716276 | -1.395640843 | 0.143004415 | -9.75942 | 1.68E-22 | 2.55E-21 | DOWN |
| ADRA1A | 224.0949528 | -2.041580512 | 0.209198134 | -9.75908 | 1.69E-22 | 2.56E-21 | DOWN |
| VGF | 131.3958506 | 2.298921113 | 0.235864638 | 9.746782 | 1.90E-22 | 2.88E-21 | UP |
| KLB | 90.21375785 | 1.930562566 | 0.198154342 | 9.742721 | 1.98E-22 | 3.00E-21 | UP |
| PNMA1 | 3036.994165 | -1.077836056 | 0.110706608 | -9.73597 | 2.12E-22 | 3.20E-21 | DOWN |
| SLC24A3 | 925.8059797 | -1.200669655 | 0.123378458 | -9.7316 | 2.21E-22 | 3.33E-21 | DOWN |

| | | | | | | | |
|---|---|---|---|---|---|---|---|
| SLC41A2 | 445.7459179 | -1.194250622 | 0.122785021 | -9.72635 | 2.33E-22 | 3.50E-21 | DOWN |
| LIME1 | 146.1755873 | 1.233330732 | 0.12695556 | 9.714665 | 2.61E-22 | 3.92E-21 | UP |
| CYP26B1 | 169.3932498 | -1.85455385 | 0.19090663 | -9.71445 | 2.62E-22 | 3.92E-21 | DOWN |
| SLC7A5 | 1046.657353 | -1.737456196 | 0.178867864 | -9.71363 | 2.64E-22 | 3.94E-21 | DOWN |
| GLIS1 | 292.3234164 | -1.669904882 | 0.171956816 | -9.71119 | 2.70E-22 | 4.03E-21 | DOWN |
| PLA2G7 | 3560.492589 | 1.763512311 | 0.181820596 | 9.699189 | 3.04E-22 | 4.53E-21 | UP |
| CTHRC1 | 304.5271296 | 1.854007049 | 0.191151903 | 9.699129 | 3.04E-22 | 4.53E-21 | UP |
| SCN2B | 91.8476523 | -1.591086135 | 0.164331517 | -9.68217 | 3.59E-22 | 5.33E-21 | DOWN |
| CYP2E1 | 160.9487918 | -1.768284664 | 0.18269897 | -9.67868 | 3.71E-22 | 5.51E-21 | DOWN |
| SH3RF1 | 7719.515793 | 1.16821199 | 0.120725454 | 9.6766 | 3.79E-22 | 5.61E-21 | UP |
| HCG11 | 348.779537 | -1.022470379 | 0.105712105 | -9.67222 | 3.96E-22 | 5.84E-21 | DOWN |
| NETO1 | 46.67538015 | 4.671064427 | 0.482953609 | 9.67187 | 3.97E-22 | 5.86E-21 | UP |
| SYDE1 | 879.7049272 | -1.078249984 | 0.111511235 | -9.66943 | 4.07E-22 | 5.98E-21 | DOWN |
| FZD8 | 925.9154517 | 1.516184515 | 0.156907679 | 9.662908 | 4.33E-22 | 6.37E-21 | UP |
| PHYHIP | 322.5034523 | -1.407789049 | 0.145941002 | -9.64629 | 5.10E-22 | 7.47E-21 | DOWN |
| SLC25A27 | 971.4820668 | 1.473781499 | 0.152865825 | 9.641014 | 5.37E-22 | 7.85E-21 | UP |
| RASSF5 | 902.6720024 | -1.121239735 | 0.116415538 | -9.63136 | 5.89E-22 | 8.60E-21 | DOWN |
| CAPN6 | 474.7775382 | -2.238112994 | 0.232378222 | -9.63134 | 5.90E-22 | 8.60E-21 | DOWN |
| TMEM108 | 56.99426572 | -1.680798732 | 0.174536729 | -9.63006 | 5.97E-22 | 8.70E-21 | DOWN |
| PDGFD | 838.2926281 | -1.141324776 | 0.118599192 | -9.62338 | 6.37E-22 | 9.24E-21 | DOWN |
| CHST3 | 1179.586987 | -1.188257333 | 0.123485628 | -9.62264 | 6.42E-22 | 9.30E-21 | DOWN |
| P2RY2 | 56.97650098 | -1.623589601 | 0.168778472 | -9.61965 | 6.61E-22 | 9.56E-21 | DOWN |
| PLK1 | 224.444629 | 1.526839058 | 0.158740855 | 9.618438 | 6.68E-22 | 9.66E-21 | UP |
| REC8 | 403.7199725 | -1.079814305 | 0.11227947 | -9.6172 | 6.76E-22 | 9.77E-21 | DOWN |
| FOLR1 | 62.30602129 | -2.304173539 | 0.23970253 | -9.61264 | 7.07E-22 | 1.02E-20 | DOWN |
| SOX7 | 295.8961293 | -1.218308032 | 0.126825778 | -9.60615 | 7.53E-22 | 1.08E-20 | DOWN |
| HSPB1 | 24995.98163 | -1.23233118 | 0.128321475 | -9.60347 | 7.73E-22 | 1.11E-20 | DOWN |
| ERCC6L | 79.42011517 | 1.443046663 | 0.150309749 | 9.600486 | 7.96E-22 | 1.14E-20 | UP |
| KCNH2 | 1241.658268 | -1.219578965 | 0.127207337 | -9.58733 | 9.04E-22 | 1.29E-20 | DOWN |
| CDO1 | 895.4984681 | -2.320211975 | 0.242075114 | -9.58468 | 9.27E-22 | 1.32E-20 | DOWN |
| RTN4RL2 | 220.632226 | 1.25553408 | 0.130997375 | 9.584422 | 9.30E-22 | 1.32E-20 | UP |
| IER3 | 1347.654363 | -1.738700774 | 0.181419422 | -9.58387 | 9.35E-22 | 1.33E-20 | DOWN |
| KIFC1 | 309.1241606 | 1.47616257 | 0.154037863 | 9.583115 | 9.42E-22 | 1.34E-20 | UP |
| PRC1 | 638.4715324 | 1.154833939 | 0.120568738 | 9.57822 | 9.87E-22 | 1.40E-20 | UP |
| SYNPO2 | 24471.9758 | -1.721341144 | 0.179938625 | -9.56627 | 1.11E-21 | 1.56E-20 | DOWN |
| TUBA4A | 1712.505143 | -1.146798265 | 0.119884806 | -9.56583 | 1.11E-21 | 1.57E-20 | DOWN |
| CPEB1 | 92.77058133 | -1.240042119 | 0.129677037 | -9.56254 | 1.15E-21 | 1.61E-20 | DOWN |
| PHYHIPL | 143.0536096 | -1.679912007 | 0.175733297 | -9.55944 | 1.18E-21 | 1.66E-20 | DOWN |
| KCNQ4 | 244.3226771 | -1.056247165 | 0.110574577 | -9.55235 | 1.27E-21 | 1.78E-20 | DOWN |
| SPEG | 1963.851866 | -1.425484663 | 0.149267014 | -9.5499 | 1.30E-21 | 1.82E-20 | DOWN |
| TPX2 | 547.1018787 | 1.628619227 | 0.170685348 | 9.541646 | 1.41E-21 | 1.97E-20 | UP |
| P2RX2 | 111.6343249 | -1.873764307 | 0.196452751 | -9.53799 | 1.46E-21 | 2.03E-20 | DOWN |
| ENTPD3 | 263.4740902 | -1.312014265 | 0.137686862 | -9.52897 | 1.59E-21 | 2.21E-20 | DOWN |
| PRKCA | 2091.046631 | -1.230400776 | 0.129183967 | -9.52441 | 1.66E-21 | 2.30E-20 | DOWN |
| SORBS1 | 9191.224647 | -1.58354616 | 0.166305483 | -9.52191 | 1.70E-21 | 2.35E-20 | DOWN |
| MANEAL | 1042.040164 | 1.054040964 | 0.110711414 | 9.520617 | 1.72E-21 | 2.38E-20 | UP |
| P2RX5 | 88.8339278 | 1.715586006 | 0.180215409 | 9.519641 | 1.74E-21 | 2.40E-20 | UP |
| TGM1 | 126.3867521 | -1.374189367 | 0.144403961 | -9.51629 | 1.79E-21 | 2.47E-20 | DOWN |
| HR | 551.4023473 | -1.265625129 | 0.133049367 | -9.51245 | 1.86E-21 | 2.56E-20 | DOWN |
| AK5 | 876.5843604 | 2.147153559 | 0.225747876 | 9.51129 | 1.88E-21 | 2.59E-20 | UP |
| C19orf18 | 107.327288 | 1.675667239 | 0.176186104 | 9.51078 | 1.89E-21 | 2.60E-20 | UP |
| SNHG8 | 6092.986626 | 1.017378043 | 0.107098067 | 9.4995 | 2.11E-21 | 2.88E-20 | UP |
| KCNJ8 | 560.5423199 | -1.188355671 | 0.125117704 | -9.4979 | 2.14E-21 | 2.92E-20 | DOWN |
| KCNQ5 | 116.1868545 | -1.442454955 | 0.151890687 | -9.49666 | 2.17E-21 | 2.95E-20 | DOWN |
| SPDYA | 57.53954957 | 1.509867044 | 0.159001417 | 9.495935 | 2.18E-21 | 2.97E-20 | UP |
| MEIS2 | 2114.436364 | -1.384236498 | 0.145899116 | -9.48763 | 2.36E-21 | 3.19E-20 | DOWN |
| TMEM100 | 283.4066804 | -1.80520061 | 0.190291493 | -9.4865 | 2.39E-21 | 3.23E-20 | DOWN |
| SLC8A1 | 1627.723225 | -1.431836699 | 0.15093659 | -9.48635 | 2.39E-21 | 3.23E-20 | DOWN |
| ID4 | 1438.462913 | -1.55397186 | 0.163823742 | -9.48563 | 2.41E-21 | 3.24E-20 | DOWN |
| FAM111B | 338.1243778 | 1.763401911 | 0.185976249 | 9.481866 | 2.50E-21 | 3.36E-20 | UP |
| SLC22A17 | 3813.974265 | -1.056155657 | 0.111450632 | -9.47644 | 2.63E-21 | 3.53E-20 | DOWN |
| ANK2 | 618.1292914 | -1.371853075 | 0.144900601 | -9.46755 | 2.86E-21 | 3.83E-20 | DOWN |
| C21orf62 | 50.69838248 | -2.417041892 | 0.255307716 | -9.46717 | 2.88E-21 | 3.84E-20 | DOWN |

| | | | | | | | |
|---|---|---|---|---|---|---|---|
| PRODH | 48.81115907 | -1.549671104 | 0.163735956 | -9.46445 | 2.95E-21 | 3.92E-20 | DOWN |
| LY6D | 97.40275233 | -3.536073936 | 0.374025421 | -9.4541 | 3.26E-21 | 4.32E-20 | DOWN |
| ANGPTL1 | 269.9169976 | -1.76376075 | 0.186610017 | -9.45159 | 3.34E-21 | 4.42E-20 | DOWN |
| REEP2 | 394.6009873 | -1.09015983 | 0.115464325 | -9.44153 | 3.67E-21 | 4.84E-20 | DOWN |
| ARHGEF19 | 765.6246527 | -1.157061752 | 0.122564905 | -9.4404 | 3.71E-21 | 4.88E-20 | DOWN |
| CA12 | 645.7698802 | -1.519102582 | 0.160999085 | -9.43547 | 3.89E-21 | 5.09E-20 | DOWN |
| PRR16 | 1550.300175 | 1.934929413 | 0.205124513 | 9.432951 | 3.99E-21 | 5.21E-20 | UP |
| SPOCK3 | 2366.053931 | -1.564177821 | 0.165926653 | -9.42692 | 4.22E-21 | 5.50E-20 | DOWN |
| EFS | 1876.50255 | -1.475808041 | 0.15679201 | -9.41252 | 4.84E-21 | 6.28E-20 | DOWN |
| GPM6B | 1078.19112 | -1.10113495 | 0.117003021 | -9.41117 | 4.91E-21 | 6.36E-20 | DOWN |
| ZMYND10 | 177.7134747 | 2.100427879 | 0.223318271 | 9.405535 | 5.18E-21 | 6.69E-20 | UP |
| IGSF1 | 129.8877193 | -2.215825866 | 0.235749479 | -9.39907 | 5.50E-21 | 7.09E-20 | DOWN |
| SAMD14 | 169.4064706 | -1.082895951 | 0.115257752 | -9.39543 | 5.70E-21 | 7.33E-20 | DOWN |
| DZIP1 | 546.1517019 | -1.023369809 | 0.108942055 | -9.39371 | 5.79E-21 | 7.44E-20 | DOWN |
| RHOB | 21461.56643 | -1.241448475 | 0.132159825 | -9.39354 | 5.80E-21 | 7.44E-20 | DOWN |
| DNAJB1 | 9463.800458 | -1.177224956 | 0.125338397 | -9.39237 | 5.87E-21 | 7.52E-20 | DOWN |
| CPAMD8 | 2857.181258 | -1.380097579 | 0.147002798 | -9.38824 | 6.10E-21 | 7.80E-20 | DOWN |
| DDR2 | 2411.734003 | -1.280173875 | 0.136365112 | -9.38784 | 6.12E-21 | 7.83E-20 | DOWN |
| GALNT6 | 174.4624853 | -1.074757136 | 0.114558752 | -9.38171 | 6.49E-21 | 8.28E-20 | DOWN |
| SPAG5 | 386.5817602 | 1.28213709 | 0.136697064 | 9.379405 | 6.63E-21 | 8.45E-20 | UP |
| EMP3 | 722.5572342 | -1.059057931 | 0.113030707 | -9.36965 | 7.28E-21 | 9.24E-20 | DOWN |
| B3GNT6 | 754.7321119 | 4.144872559 | 0.442621766 | 9.364367 | 7.65E-21 | 9.70E-20 | UP |
| SLCO1B3 | 90.88314732 | 4.118649077 | 0.440108039 | 9.358268 | 8.11E-21 | 1.02E-19 | UP |
| PPP1R12B | 14904.62461 | -1.318127438 | 0.141121458 | -9.34038 | 9.60E-21 | 1.20E-19 | DOWN |
| CNN1 | 23160.74603 | -1.608103055 | 0.172186937 | -9.33929 | 9.70E-21 | 1.22E-19 | DOWN |
| NEFM | 76.81811669 | -2.944547165 | 0.315508907 | -9.33269 | 1.03E-20 | 1.29E-19 | DOWN |
| RGN | 320.2372741 | -1.219204106 | 0.130730027 | -9.32612 | 1.10E-20 | 1.37E-19 | DOWN |
| CDC25A | 259.7155977 | 1.117256854 | 0.119841066 | 9.322821 | 1.13E-20 | 1.41E-19 | UP |
| TUBB6 | 2206.928668 | -1.047031964 | 0.11231104 | -9.32261 | 1.14E-20 | 1.41E-19 | DOWN |
| HIST1H2BN | 214.315158 | 1.198658093 | 0.128662384 | 9.316306 | 1.20E-20 | 1.49E-19 | UP |
| EDNRA | 2068.660326 | -1.273345223 | 0.136719891 | -9.31353 | 1.24E-20 | 1.53E-19 | DOWN |
| ZP1 | 77.16427228 | 2.26883305 | 0.243635798 | 9.312396 | 1.25E-20 | 1.55E-19 | UP |
| CAMK2N2 | 76.12224671 | 1.687619355 | 0.181323626 | 9.307223 | 1.31E-20 | 1.62E-19 | UP |
| HOXB3 | 355.0157539 | -1.589958788 | 0.170849018 | -9.30622 | 1.32E-20 | 1.63E-19 | DOWN |
| IRAK3 | 441.6947874 | -1.024106011 | 0.110075314 | -9.30368 | 1.36E-20 | 1.67E-19 | DOWN |
| S100A14 | 340.802515 | -2.127243395 | 0.228730018 | -9.30024 | 1.40E-20 | 1.72E-19 | DOWN |
| ROR2 | 1152.698395 | -1.33935681 | 0.144021439 | -9.2997 | 1.41E-20 | 1.73E-19 | DOWN |
| BUB1 | 215.9705691 | 1.556723271 | 0.167409268 | 9.298907 | 1.42E-20 | 1.74E-19 | UP |
| CECR2 | 475.0163087 | 1.310742913 | 0.140957461 | 9.298854 | 1.42E-20 | 1.74E-19 | UP |
| SCUBE1 | 134.2967164 | -1.53695265 | 0.165480462 | -9.28782 | 1.57E-20 | 1.92E-19 | DOWN |
| SLC6A11 | 500.6277703 | 1.754857658 | 0.189078133 | 9.281124 | 1.68E-20 | 2.04E-19 | UP |
| FOLH1 | 26884.20335 | 1.744074574 | 0.187924403 | 9.280724 | 1.68E-20 | 2.05E-19 | UP |
| PRRG4 | 1401.966824 | -1.089876244 | 0.117436027 | -9.2806 | 1.69E-20 | 2.05E-19 | DOWN |
| TMEM158 | 846.8239217 | -1.407071854 | 0.151637997 | -9.27915 | 1.71E-20 | 2.07E-19 | DOWN |
| ZBTB7C | 393.5283839 | -1.354063151 | 0.145990435 | -9.27501 | 1.78E-20 | 2.14E-19 | DOWN |
| LONRF3 | 74.25062507 | -1.288113464 | 0.138914548 | -9.2727 | 1.81E-20 | 2.19E-19 | DOWN |
| SSPN | 1525.067732 | -1.028351783 | 0.110927977 | -9.27045 | 1.85E-20 | 2.23E-19 | DOWN |
| IL1RL1 | 75.30613206 | -2.133363613 | 0.230224882 | -9.26643 | 1.92E-20 | 2.31E-19 | DOWN |
| RNF112 | 181.9823701 | -1.709530456 | 0.184542885 | -9.26359 | 1.98E-20 | 2.36E-19 | DOWN |
| CCDC7 | 122.4029325 | 1.041824133 | 0.11249221 | 9.2613 | 2.02E-20 | 2.41E-19 | UP |
| NCAPH | 157.8347239 | 1.616434928 | 0.174565944 | 9.259738 | 2.05E-20 | 2.45E-19 | UP |
| FILIP1 | 275.6095192 | -1.573172233 | 0.169922023 | -9.2582 | 2.08E-20 | 2.48E-19 | DOWN |
| MARVELD1 | 2304.805454 | -1.029130744 | 0.11116806 | -9.25743 | 2.09E-20 | 2.49E-19 | DOWN |
| GLYATL1 | 3591.797907 | 1.849679403 | 0.199898247 | 9.253105 | 2.18E-20 | 2.59E-19 | UP |
| NOTCH1 | 3164.591862 | -1.127103623 | 0.121934336 | -9.24353 | 2.38E-20 | 2.83E-19 | DOWN |
| FAM162B | 71.81643318 | -1.118059365 | 0.120963303 | -9.24296 | 2.40E-20 | 2.84E-19 | DOWN |
| RAB40A | 56.57506456 | -1.370657923 | 0.14830875 | -9.24192 | 2.42E-20 | 2.86E-19 | DOWN |
| KRT20 | 24.62388392 | 3.662937891 | 0.396578257 | 9.236356 | 2.55E-20 | 3.01E-19 | UP |
| HPD | 49.46025257 | -1.472097545 | 0.159499924 | -9.22946 | 2.72E-20 | 3.21E-19 | DOWN |
| DDIT4 | 3157.351608 | -1.295783805 | 0.14048937 | -9.22336 | 2.88E-20 | 3.39E-19 | DOWN |
| TNS1 | 23459.43364 | -1.461771876 | 0.158502074 | -9.22241 | 2.90E-20 | 3.42E-19 | DOWN |
| ZDHHC11 | 267.9157127 | 1.608618684 | 0.174436009 | 9.221827 | 2.92E-20 | 3.44E-19 | UP |
| PLCL1 | 636.0814153 | -1.52889685 | 0.165819211 | -9.22026 | 2.96E-20 | 3.48E-19 | DOWN |

| | | | | | | | |
|---|---|---|---|---|---|---|---|
| GJA1 | 4796.729327 | -1.287668144 | 0.139664807 | -9.2197 | 2.98E-20 | 3.50E-19 | DOWN |
| SPC24 | 171.8501198 | 1.509899823 | 0.163817403 | 9.216968 | 3.06E-20 | 3.58E-19 | UP |
| IQCA1 | 102.9668346 | -1.247901249 | 0.135394051 | -9.21681 | 3.06E-20 | 3.59E-19 | DOWN |
| KIF20A | 236.1203861 | 1.68984059 | 0.183433394 | 9.212284 | 3.19E-20 | 3.72E-19 | UP |
| HLF | 1171.402885 | -1.167736437 | 0.126774964 | -9.2111 | 3.23E-20 | 3.76E-19 | DOWN |
| FUT2 | 160.2172501 | -1.649244505 | 0.179112636 | -9.20786 | 3.33E-20 | 3.87E-19 | DOWN |
| CDCA3 | 185.1098578 | 1.379453055 | 0.149827918 | 9.206916 | 3.36E-20 | 3.91E-19 | UP |
| ITGA9 | 1533.694468 | -1.251472819 | 0.135945683 | -9.20568 | 3.40E-20 | 3.94E-19 | DOWN |
| ACSS3 | 584.2381601 | -1.175902261 | 0.127738547 | -9.20554 | 3.40E-20 | 3.94E-19 | DOWN |
| TRO | 270.7547909 | -1.077135172 | 0.117117162 | -9.19707 | 3.68E-20 | 4.24E-19 | DOWN |
| ASS1 | 4437.402801 | -1.358446794 | 0.147718217 | -9.1962 | 3.71E-20 | 4.27E-19 | DOWN |
| PHF21B | 417.1103258 | 1.461306118 | 0.158905989 | 9.196042 | 3.71E-20 | 4.27E-19 | UP |
| ZNF804A | 57.82670182 | -1.46621323 | 0.15949359 | -9.19293 | 3.82E-20 | 4.39E-19 | DOWN |
| NFE2L3 | 687.3994755 | 1.184066309 | 0.12881171 | 9.192226 | 3.85E-20 | 4.41E-19 | UP |
| DUSP15 | 123.7372484 | -1.382605729 | 0.150578026 | -9.18199 | 4.23E-20 | 4.84E-19 | DOWN |
| ATP2A3 | 3779.237474 | -1.37418691 | 0.149677166 | -9.18101 | 4.27E-20 | 4.88E-19 | DOWN |
| CCNJL | 86.63867941 | -1.240125936 | 0.135224786 | -9.17085 | 4.69E-20 | 5.34E-19 | DOWN |
| CCBE1 | 151.0840108 | -1.892167892 | 0.206349282 | -9.16973 | 4.74E-20 | 5.39E-19 | DOWN |
| SLITRK6 | 444.7660671 | -1.619928843 | 0.176722304 | -9.16652 | 4.89E-20 | 5.55E-19 | DOWN |
| LRRIQ1 | 295.1628941 | 1.485574682 | 0.162149874 | 9.161738 | 5.11E-20 | 5.79E-19 | UP |
| REPS2 | 7624.452994 | 1.098552203 | 0.119991372 | 9.15526 | 5.42E-20 | 6.14E-19 | UP |
| KRT13 | 2421.793291 | -3.489757644 | 0.381455046 | -9.14854 | 5.77E-20 | 6.52E-19 | DOWN |
| HIST1H4E | 108.0806244 | 2.327656824 | 0.254583781 | 9.142989 | 6.07E-20 | 6.84E-19 | UP |
| SDS | 192.2919345 | 1.574023823 | 0.172191121 | 9.141144 | 6.18E-20 | 6.94E-19 | UP |
| GJB1 | 4437.209421 | 1.319693902 | 0.144428547 | 9.137348 | 6.40E-20 | 7.19E-19 | UP |
| KCNAB1 | 841.5510683 | -1.503284168 | 0.164613673 | -9.1322 | 6.71E-20 | 7.53E-19 | DOWN |
| NOL4 | 229.1935176 | 2.3963002 | 0.262485742 | 9.129259 | 6.90E-20 | 7.73E-19 | UP |
| GPLD1 | 110.5515813 | -1.014289473 | 0.111131878 | -9.1269 | 7.05E-20 | 7.89E-19 | DOWN |
| COLEC12 | 5548.716127 | 1.143262443 | 0.125276968 | 9.125879 | 7.12E-20 | 7.96E-19 | UP |
| KLK15 | 386.8794941 | 1.974328608 | 0.216349585 | 9.125641 | 7.13E-20 | 7.97E-19 | UP |
| ITGB3 | 135.1435665 | -1.388816577 | 0.152260035 | -9.12135 | 7.42E-20 | 8.27E-19 | DOWN |
| CCL18 | 199.455128 | 2.280178702 | 0.249995206 | 9.12089 | 7.45E-20 | 8.30E-19 | UP |
| CHST2 | 716.1826776 | -1.312977007 | 0.14403063 | -9.11596 | 7.80E-20 | 8.66E-19 | DOWN |
| GPRASP1 | 1249.897156 | -1.078506618 | 0.118379215 | -9.11061 | 8.19E-20 | 9.08E-19 | DOWN |
| GPR37 | 300.0987151 | 1.437974352 | 0.157937917 | 9.104681 | 8.65E-20 | 9.57E-19 | UP |
| APOBEC3D | 115.3237434 | -1.163986421 | 0.127908801 | -9.10013 | 9.02E-20 | 9.95E-19 | DOWN |
| PFKFB3 | 1856.949773 | -1.092487136 | 0.120194429 | -9.08933 | 9.96E-20 | 1.10E-18 | DOWN |
| BAIAP2L2 | 348.5689566 | 2.118782682 | 0.23321329 | 9.085171 | 1.04E-19 | 1.14E-18 | UP |
| RASD2 | 1105.563726 | -1.014088231 | 0.111711062 | -9.07778 | 1.11E-19 | 1.22E-18 | DOWN |
| DOCK3 | 202.6952681 | -1.304635249 | 0.143867525 | -9.06831 | 1.21E-19 | 1.32E-18 | DOWN |
| SEMA3A | 181.2949362 | -1.527073066 | 0.168420836 | -9.06701 | 1.22E-19 | 1.34E-18 | DOWN |
| KCNS1 | 83.57664554 | -2.652333694 | 0.292548946 | -9.06629 | 1.23E-19 | 1.34E-18 | DOWN |
| PITPNM3 | 697.4490721 | -1.076441581 | 0.118796639 | -9.06121 | 1.29E-19 | 1.40E-18 | DOWN |
| FAT2 | 551.7408029 | -1.875762012 | 0.207081761 | -9.05807 | 1.33E-19 | 1.44E-18 | DOWN |
| GSTM1 | 780.3673625 | -3.415174888 | 0.377101422 | -9.05638 | 1.35E-19 | 1.46E-18 | DOWN |
| DUSP4 | 1841.952607 | -1.796485454 | 0.198733508 | -9.03967 | 1.57E-19 | 1.69E-18 | DOWN |
| LRRK2 | 373.8019425 | -1.076031436 | 0.119094165 | -9.03513 | 1.64E-19 | 1.76E-18 | DOWN |
| NLGN3 | 440.4650385 | -1.227359271 | 0.13585192 | -9.03454 | 1.65E-19 | 1.77E-18 | DOWN |
| AURKB | 129.5956738 | 1.674922704 | 0.18542475 | 9.032897 | 1.67E-19 | 1.79E-18 | UP |
| DIO1 | 71.18836043 | 2.730305601 | 0.30256745 | 9.023792 | 1.82E-19 | 1.95E-18 | UP |
| C5orf49 | 411.4153377 | 1.203565631 | 0.133401972 | 9.022098 | 1.85E-19 | 1.97E-18 | UP |
| GLI3 | 778.1559168 | -1.020077319 | 0.113092774 | -9.01983 | 1.88E-19 | 2.01E-18 | DOWN |
| NEK2 | 132.9246302 | 1.835218759 | 0.203519385 | 9.017415 | 1.93E-19 | 2.05E-18 | UP |
| HS3ST3A1 | 79.621397 | -1.308133905 | 0.145074522 | -9.01698 | 1.93E-19 | 2.06E-18 | DOWN |
| DYNC1I1 | 352.6054589 | -1.169887173 | 0.129826636 | -9.01115 | 2.04E-19 | 2.17E-18 | DOWN |
| ITIH5 | 2506.051092 | -1.242358177 | 0.137915147 | -9.00813 | 2.10E-19 | 2.23E-18 | DOWN |
| ZGLP1 | 91.91746088 | 1.39149191 | 0.154477978 | 9.007704 | 2.10E-19 | 2.23E-18 | UP |
| SERPINA1 | 1709.340412 | -1.688452197 | 0.187446461 | -9.00765 | 2.11E-19 | 2.23E-18 | DOWN |
| HAAO | 372.0154056 | -1.175710793 | 0.130551423 | -9.00573 | 2.14E-19 | 2.27E-18 | DOWN |
| ROBO1 | 2476.374117 | -1.175197787 | 0.13052422 | -9.00368 | 2.18E-19 | 2.31E-18 | DOWN |
| IRX2 | 903.8199077 | 1.017427334 | 0.113006027 | 9.003302 | 2.19E-19 | 2.31E-18 | UP |
| CATSPERB | 152.4603484 | 1.221693993 | 0.135697498 | 9.003069 | 2.19E-19 | 2.32E-18 | UP |
| CDC45 | 128.2337794 | 1.57399825 | 0.175014085 | 8.993552 | 2.39E-19 | 2.52E-18 | UP |

| | | | | | | | |
|---|---|---|---|---|---|---|---|
| CTNND2 | 896.7159621 | 1.220920127 | 0.135810205 | 8.9899 | 2.47E-19 | 2.60E-18 | UP |
| B3GAT1 | 1885.546059 | 1.619978701 | 0.180251655 | 8.987317 | 2.53E-19 | 2.66E-18 | UP |
| FZD7 | 1884.081244 | -1.025085985 | 0.11406794 | -8.98663 | 2.55E-19 | 2.67E-18 | DOWN |
| ROPN1B | 128.6485047 | 1.752246503 | 0.195049586 | 8.983595 | 2.62E-19 | 2.75E-18 | UP |
| MRVI1 | 3480.595744 | -1.36283217 | 0.151806485 | -8.97743 | 2.77E-19 | 2.90E-18 | DOWN |
| LRP4 | 446.4901012 | -1.076092003 | 0.119952849 | -8.97096 | 2.94E-19 | 3.08E-18 | DOWN |
| SCGB3A1 | 339.3484361 | -2.675392919 | 0.298265415 | -8.96984 | 2.97E-19 | 3.10E-18 | DOWN |
| NLRP8 | 64.42647065 | 2.205630995 | 0.246030917 | 8.964853 | 3.11E-19 | 3.24E-18 | UP |
| COL17A1 | 1617.270988 | -1.92845863 | 0.215172178 | -8.9624 | 3.18E-19 | 3.31E-18 | DOWN |
| SDK1 | 3577.779975 | 1.679971177 | 0.187523623 | 8.958718 | 3.28E-19 | 3.42E-18 | UP |
| TTLL6 | 48.76988709 | 1.295234645 | 0.144647866 | 8.954399 | 3.42E-19 | 3.55E-18 | UP |
| ITPRIPL1 | 108.2594339 | -1.315047909 | 0.14698525 | -8.9468 | 3.66E-19 | 3.79E-18 | DOWN |
| PITX1 | 609.6572601 | -1.436836079 | 0.160609912 | -8.94612 | 3.68E-19 | 3.81E-18 | DOWN |
| EME2 | 1277.076382 | 1.132658097 | 0.126637504 | 8.944097 | 3.75E-19 | 3.88E-18 | UP |
| CCNB1 | 632.2028596 | 1.07986279 | 0.120757459 | 8.942411 | 3.81E-19 | 3.94E-18 | UP |
| RPSAP9 | 67.19666276 | 1.215097999 | 0.136048848 | 8.931336 | 4.21E-19 | 4.33E-18 | UP |
| RAC3 | 1707.822103 | 1.033727844 | 0.115792531 | 8.927414 | 4.36E-19 | 4.48E-18 | UP |
| SLC26A1 | 322.9161779 | 1.055096229 | 0.118220582 | 8.92481 | 4.46E-19 | 4.57E-18 | UP |
| SAMD5 | 1342.571661 | 1.511939064 | 0.169418736 | 8.924273 | 4.49E-19 | 4.59E-18 | UP |
| PTGIS | 2071.389499 | -1.389196336 | 0.15571986 | -8.92113 | 4.62E-19 | 4.72E-18 | DOWN |
| GPRIN1 | 141.0034751 | 1.287540584 | 0.144336213 | 8.920427 | 4.64E-19 | 4.74E-18 | UP |
| RAB19 | 78.55000076 | 1.36367936 | 0.152871936 | 8.920404 | 4.65E-19 | 4.74E-18 | UP |
| CAND2 | 292.4233929 | -1.133979473 | 0.127174582 | -8.91671 | 4.80E-19 | 4.88E-18 | DOWN |
| TMSB15A | 2000.556736 | 1.574616892 | 0.176741438 | 8.909155 | 5.14E-19 | 5.22E-18 | UP |
| CD40 | 693.1302778 | -1.042165813 | 0.117053159 | -8.90335 | 5.42E-19 | 5.50E-18 | DOWN |
| CYBA | 1594.689813 | -1.089497737 | 0.122376504 | -8.90283 | 5.44E-19 | 5.52E-18 | DOWN |
| KIF26B | 286.0881997 | -1.541104033 | 0.173208922 | -8.89737 | 5.72E-19 | 5.79E-18 | DOWN |
| CCNE2 | 107.7428575 | 1.130774265 | 0.127157922 | 8.892677 | 5.97E-19 | 6.04E-18 | UP |
| PATE1 | 1126.556242 | -8.583364177 | 0.966148692 | -8.8841 | 6.44E-19 | 6.49E-18 | DOWN |
| ITGB8 | 1487.599784 | -1.218077726 | 0.137167691 | -8.88021 | 6.67E-19 | 6.70E-18 | DOWN |
| AJAP1 | 118.039183 | -1.500944085 | 0.169034155 | -8.87953 | 6.71E-19 | 6.73E-18 | DOWN |
| GHRHR | 46.38375582 | 2.374635574 | 0.267542594 | 8.875729 | 6.95E-19 | 6.96E-18 | UP |
| APBA2 | 655.0728048 | 1.154799734 | 0.130208133 | 8.868876 | 7.39E-19 | 7.38E-18 | UP |
| ASPM | 178.3905821 | 1.610390288 | 0.181594413 | 8.868061 | 7.44E-19 | 7.42E-18 | UP |
| HIF3A | 271.9999211 | -1.57755535 | 0.177946011 | -8.86536 | 7.63E-19 | 7.59E-18 | DOWN |
| TRHDE | 124.6692909 | -1.546557295 | 0.174499764 | -8.8628 | 7.80E-19 | 7.76E-18 | DOWN |
| CYP2J2 | 1018.89632 | 1.560527429 | 0.176172201 | 8.857966 | 8.15E-19 | 8.10E-18 | UP |
| MAML2 | 1322.071009 | -1.025710352 | 0.115851153 | -8.85369 | 8.47E-19 | 8.40E-18 | DOWN |
| EVX2 | 69.43300852 | -2.340810225 | 0.264531166 | -8.8489 | 8.84E-19 | 8.76E-18 | DOWN |
| CSPG4 | 1787.086808 | -1.305147889 | 0.147563045 | -8.84468 | 9.18E-19 | 9.07E-18 | DOWN |
| TFF3 | 13754.38499 | 2.614138455 | 0.29558035 | 8.844087 | 9.23E-19 | 9.11E-18 | UP |
| SCNN1D | 329.862033 | 1.16327442 | 0.131541911 | 8.843375 | 9.29E-19 | 9.16E-18 | UP |
| CLCA2 | 173.1266486 | -2.946867231 | 0.333361405 | -8.83986 | 9.58E-19 | 9.44E-18 | DOWN |
| SNHG10 | 293.2512666 | 1.044138093 | 0.118118247 | 8.83977 | 9.59E-19 | 9.44E-18 | UP |
| TUBB2A | 1815.02316 | 1.298020045 | 0.146866728 | 8.838081 | 9.74E-19 | 9.57E-18 | UP |
| SKA1 | 86.83188765 | 1.533471187 | 0.173590769 | 8.833829 | 1.01E-18 | 9.92E-18 | UP |
| DTNA | 1212.404568 | -1.260595423 | 0.142735763 | -8.83167 | 1.03E-18 | 1.01E-17 | DOWN |
| ANXA2 | 14311.41577 | -1.108448759 | 0.125542189 | -8.82929 | 1.05E-18 | 1.03E-17 | DOWN |
| SOSTDC1 | 83.31442073 | -1.782724525 | 0.202001039 | -8.82532 | 1.09E-18 | 1.06E-17 | DOWN |
| CDKL1 | 111.5089039 | -1.007202691 | 0.114165464 | -8.82231 | 1.12E-18 | 1.09E-17 | DOWN |
| BLM | 118.8419027 | 1.108277698 | 0.125627088 | 8.821964 | 1.12E-18 | 1.09E-17 | UP |
| BNIPL | 267.8929831 | -1.652351023 | 0.187329386 | -8.82056 | 1.14E-18 | 1.11E-17 | DOWN |
| PTGDS | 11923.3833 | -1.637521387 | 0.185794388 | -8.81362 | 1.21E-18 | 1.18E-17 | DOWN |
| KCNG1 | 488.4405476 | -1.148180192 | 0.13040783 | -8.80453 | 1.31E-18 | 1.27E-17 | DOWN |
| ID3 | 3175.807301 | -1.298462948 | 0.147534233 | -8.8011 | 1.35E-18 | 1.31E-17 | DOWN |
| PRR22 | 209.9260556 | 1.040059339 | 0.118209424 | 8.798447 | 1.39E-18 | 1.34E-17 | UP |
| ACTC1 | 2144.921892 | -2.659148348 | 0.302362671 | -8.79457 | 1.44E-18 | 1.38E-17 | DOWN |
| HEPH | 1250.31654 | -1.209187978 | 0.137790205 | -8.77557 | 1.70E-18 | 1.63E-17 | DOWN |
| CYP4F22 | 118.9090863 | -2.497820253 | 0.284795258 | -8.77058 | 1.78E-18 | 1.70E-17 | DOWN |
| GJC1 | 700.5655258 | -1.127033899 | 0.128612701 | -8.76301 | 1.90E-18 | 1.82E-17 | DOWN |
| VAV3 | 396.2905368 | -1.184431669 | 0.135178591 | -8.76198 | 1.92E-18 | 1.83E-17 | DOWN |
| PLS3 | 1561.429435 | -1.013326621 | 0.11576381 | -8.7534 | 2.07E-18 | 1.97E-17 | DOWN |
| GSTM5 | 610.4000458 | -1.426363659 | 0.163007733 | -8.75028 | 2.13E-18 | 2.02E-17 | DOWN |

| | | | | | | | |
|---|---|---|---|---|---|---|---|
| MRGPRF | 1970.572856 | -1.261818732 | 0.144222463 | -8.74911 | 2.15E-18 | 2.04E-17 | DOWN |
| RGS7BP | 190.2101152 | -1.453591995 | 0.166406847 | -8.73517 | 2.43E-18 | 2.30E-17 | DOWN |
| PCDHGA1 | 130.5000558 | 1.588321303 | 0.18197834 | 8.728079 | 2.59E-18 | 2.43E-17 | UP |
| FAT3 | 364.0126312 | -1.767381863 | 0.202590325 | -8.72392 | 2.69E-18 | 2.52E-17 | DOWN |
| BRSK2 | 381.062815 | 2.198804994 | 0.252247587 | 8.716852 | 2.86E-18 | 2.68E-17 | UP |
| ERG | 7078.111621 | 2.567248523 | 0.294613108 | 8.713966 | 2.93E-18 | 2.74E-17 | UP |
| IQSEC3 | 140.4003699 | -1.56325501 | 0.179407896 | -8.71341 | 2.95E-18 | 2.75E-17 | DOWN |
| PDE2A | 591.7682266 | -1.039972366 | 0.11936458 | -8.71257 | 2.97E-18 | 2.77E-17 | DOWN |
| MAL | 96.85173136 | -1.801649249 | 0.206869944 | -8.70909 | 3.06E-18 | 2.85E-17 | DOWN |
| CENPN | 2646.004589 | 1.349732431 | 0.155000535 | 8.707921 | 3.09E-18 | 2.88E-17 | UP |
| RGS22 | 83.98126293 | -1.082829507 | 0.124359369 | -8.70726 | 3.11E-18 | 2.89E-17 | DOWN |
| GDF15 | 25638.69484 | 1.64344463 | 0.188758976 | 8.706577 | 3.13E-18 | 2.91E-17 | UP |
| RAB34 | 2085.114344 | -1.007418973 | 0.11574495 | -8.70378 | 3.21E-18 | 2.98E-17 | DOWN |
| HBB | 451.6193936 | -1.73658697 | 0.199693019 | -8.69628 | 3.43E-18 | 3.17E-17 | DOWN |
| TIMP3 | 366.633192 | -1.422317121 | 0.163610929 | -8.69329 | 3.52E-18 | 3.25E-17 | DOWN |
| NUF2 | 95.74729759 | 1.504492871 | 0.173365228 | 8.67817 | 4.02E-18 | 3.70E-17 | UP |
| RFX3 | 1406.994565 | 1.041263562 | 0.119988278 | 8.678044 | 4.03E-18 | 3.71E-17 | UP |
| MEIS1 | 1099.017103 | -1.142406446 | 0.131721296 | -8.67291 | 4.21E-18 | 3.87E-17 | DOWN |
| CHKB-CPT1B | 70.42605216 | 1.334665349 | 0.154014882 | 8.665821 | 4.48E-18 | 4.10E-17 | UP |
| ATOH1 | 90.19962612 | 4.294166408 | 0.495529207 | 8.665819 | 4.48E-18 | 4.10E-17 | UP |
| SNAI2 | 785.0793409 | -1.464183693 | 0.168969874 | -8.66535 | 4.50E-18 | 4.11E-17 | DOWN |
| ITGBL1 | 346.9553976 | 1.863006548 | 0.215067127 | 8.662442 | 4.62E-18 | 4.22E-17 | UP |
| EDNRB | 1160.938744 | -1.521320807 | 0.17568546 | -8.65934 | 4.74E-18 | 4.33E-17 | DOWN |
| SNHG12 | 510.5114376 | 1.162848773 | 0.134294591 | 8.65894 | 4.76E-18 | 4.34E-17 | UP |
| CDKN3 | 119.8295679 | 1.494311808 | 0.172581469 | 8.658588 | 4.78E-18 | 4.35E-17 | UP |
| GLOD5 | 58.09910147 | 1.417875662 | 0.16385083 | 8.653454 | 5.00E-18 | 4.54E-17 | UP |
| KLF5 | 2800.814193 | -1.208954469 | 0.13974974 | -8.65085 | 5.11E-18 | 4.64E-17 | DOWN |
| SRD5A2 | 1063.708577 | -1.64594857 | 0.190273554 | -8.65043 | 5.13E-18 | 4.65E-17 | DOWN |
| NMUR1 | 75.95420506 | -1.298691319 | 0.150158431 | -8.64881 | 5.20E-18 | 4.71E-17 | DOWN |
| BEND3 | 599.6906978 | 1.100744821 | 0.12732808 | 8.644949 | 5.38E-18 | 4.87E-17 | UP |
| TTK | 244.3407572 | 1.427058485 | 0.1651322 | 8.641915 | 5.53E-18 | 4.99E-17 | UP |
| KIF11 | 417.113125 | 1.103443468 | 0.127794769 | 8.634496 | 5.90E-18 | 5.31E-17 | UP |
| MYRIP | 1025.824986 | 1.06965564 | 0.123921486 | 8.631721 | 6.04E-18 | 5.44E-17 | UP |
| GNG11 | 1098.969112 | -1.029876986 | 0.119351583 | -8.62893 | 6.19E-18 | 5.57E-17 | DOWN |
| PRDM8 | 1265.824174 | -1.755188952 | 0.203454557 | -8.62693 | 6.30E-18 | 5.67E-17 | DOWN |
| NCAM1 | 787.294856 | -1.763733988 | 0.204458029 | -8.62639 | 6.33E-18 | 5.68E-17 | DOWN |
| SLIT3 | 1192.360842 | -1.326135336 | 0.153774916 | -8.62387 | 6.47E-18 | 5.79E-17 | DOWN |
| CD200 | 496.6871585 | -1.1436817 | 0.132633362 | -8.62288 | 6.53E-18 | 5.84E-17 | DOWN |
| SFRP5 | 95.07400199 | -2.977740603 | 0.34559126 | -8.61637 | 6.91E-18 | 6.16E-17 | DOWN |
| SEC31B | 418.1456167 | 1.230264129 | 0.142895686 | 8.609526 | 7.34E-18 | 6.52E-17 | UP |
| GJB5 | 104.9519021 | -1.962755723 | 0.227994197 | -8.6088 | 7.38E-18 | 6.55E-17 | DOWN |
| NOX1 | 134.0018613 | -1.528279997 | 0.17754653 | -8.60777 | 7.45E-18 | 6.61E-17 | DOWN |
| PBK | 119.8466279 | 1.734136783 | 0.201477208 | 8.607111 | 7.49E-18 | 6.64E-17 | UP |
| CENPE | 223.7862468 | 1.172033193 | 0.136256266 | 8.601683 | 7.86E-18 | 6.94E-17 | UP |
| PTTG1 | 299.6105853 | 1.458250236 | 0.16966248 | 8.595007 | 8.33E-18 | 7.35E-17 | UP |
| C1QL1 | 56.47446709 | -2.235382652 | 0.260123261 | -8.59355 | 8.43E-18 | 7.43E-17 | DOWN |
| COL10A1 | 463.9703534 | 2.36462386 | 0.275191616 | 8.592645 | 8.50E-18 | 7.48E-17 | UP |
| SLITRK3 | 79.65400831 | -2.335613833 | 0.271840273 | -8.59186 | 8.56E-18 | 7.52E-17 | DOWN |
| UGT1A3 | 70.47235991 | 3.621661927 | 0.421569224 | 8.590907 | 8.63E-18 | 7.58E-17 | UP |
| HSF4 | 1735.742058 | 1.296980888 | 0.151076344 | 8.584937 | 9.09E-18 | 7.97E-17 | UP |
| MEX3A | 935.5102758 | 1.681532761 | 0.195876414 | 8.584662 | 9.11E-18 | 7.98E-17 | UP |
| SOX8 | 211.894923 | 1.222168099 | 0.142499402 | 8.576654 | 9.77E-18 | 8.53E-17 | UP |
| CPZ | 52.82687042 | -1.761189115 | 0.205554738 | -8.56798 | 1.05E-17 | 9.17E-17 | DOWN |
| ANO5 | 438.6912123 | -1.377352873 | 0.160901341 | -8.56023 | 1.13E-17 | 9.78E-17 | DOWN |
| ZNF204P | 381.5027616 | -1.249798515 | 0.146022654 | -8.55894 | 1.14E-17 | 9.89E-17 | DOWN |
| ATRNL1 | 282.8931461 | -1.54000332 | 0.179979897 | -8.55653 | 1.16E-17 | 1.01E-16 | DOWN |
| MEIS3P1 | 142.1607441 | -1.161425186 | 0.135753524 | -8.5554 | 1.17E-17 | 1.02E-16 | DOWN |
| NRXN3 | 539.9698192 | -1.384244169 | 0.161873208 | -8.55141 | 1.22E-17 | 1.05E-16 | DOWN |
| SCARA5 | 102.202558 | -2.246937026 | 0.262773459 | -8.55085 | 1.22E-17 | 1.06E-16 | DOWN |
| CFD | 1619.62511 | -1.716380778 | 0.200791717 | -8.54807 | 1.25E-17 | 1.08E-16 | DOWN |
| MYL9 | 53217.4858 | -1.398450111 | 0.163609641 | -8.54748 | 1.26E-17 | 1.08E-16 | DOWN |
| FAM124A | 368.2286182 | -1.09358308 | 0.12798198 | -8.54482 | 1.29E-17 | 1.11E-16 | DOWN |
| GABRB3 | 2357.368343 | 1.444098472 | 0.169021152 | 8.543892 | 1.30E-17 | 1.12E-16 | UP |

| | | | | | | | |
|---|---|---|---|---|---|---|---|
| KIAA1614 | 218.4261022 | -1.156856231 | 0.135573143 | -8.53308 | 1.43E-17 | 1.22E-16 | DOWN |
| EN2 | 55.22731233 | 2.843635796 | 0.333269989 | 8.532529 | 1.43E-17 | 1.23E-16 | UP |
| P2RX1 | 373.4169777 | -1.588109669 | 0.186294232 | -8.52474 | 1.53E-17 | 1.31E-16 | DOWN |
| FHIT | 616.3384869 | 1.049917084 | 0.123178153 | 8.523566 | 1.55E-17 | 1.32E-16 | UP |
| AGMAT | 218.1589084 | 1.394463475 | 0.163666568 | 8.520149 | 1.59E-17 | 1.36E-16 | UP |
| CAP2 | 1086.179436 | -1.071485465 | 0.125770829 | -8.51935 | 1.60E-17 | 1.36E-16 | DOWN |
| SYNC | 188.7064728 | -1.213566437 | 0.142506173 | -8.51589 | 1.65E-17 | 1.40E-16 | DOWN |
| CLVS2 | 222.8195636 | -1.729969119 | 0.203353955 | -8.50718 | 1.78E-17 | 1.51E-16 | DOWN |
| LRCH2 | 601.0633184 | -1.204796447 | 0.141631201 | -8.50658 | 1.79E-17 | 1.52E-16 | DOWN |
| UBE2C | 238.5573165 | 1.700128151 | 0.19993884 | 8.503241 | 1.84E-17 | 1.56E-16 | UP |
| TK1 | 814.0356487 | 1.21174807 | 0.142515448 | 8.502574 | 1.85E-17 | 1.57E-16 | UP |
| GRIN3A | 1351.798188 | 2.635679052 | 0.310000598 | 8.502174 | 1.86E-17 | 1.57E-16 | UP |
| PENK | 247.3821222 | -2.205210707 | 0.259481288 | -8.49853 | 1.92E-17 | 1.62E-16 | DOWN |
| FGFR2 | 2096.67372 | -1.487461916 | 0.175126561 | -8.49364 | 2.00E-17 | 1.68E-16 | DOWN |
| SSTR1 | 1056.297919 | 2.142874693 | 0.252417859 | 8.489394 | 2.08E-17 | 1.74E-16 | UP |
| AOC3 | 3870.931685 | -1.210434947 | 0.142606507 | -8.48794 | 2.10E-17 | 1.76E-16 | DOWN |
| CEP55 | 140.8571182 | 1.528145015 | 0.180173558 | 8.481517 | 2.22E-17 | 1.86E-16 | UP |
| NTM | 302.2628963 | 1.974560227 | 0.232844665 | 8.480161 | 2.25E-17 | 1.88E-16 | UP |
| BNC2 | 453.5010777 | -1.129767513 | 0.133225596 | -8.48011 | 2.25E-17 | 1.88E-16 | DOWN |
| GABRD | 60.65997526 | 1.791248586 | 0.211279688 | 8.478092 | 2.29E-17 | 1.91E-16 | UP |
| SMOC1 | 1610.937664 | -1.710267523 | 0.201774072 | -8.47615 | 2.33E-17 | 1.94E-16 | DOWN |
| ZFP36L1 | 16011.44191 | -1.047894733 | 0.123629341 | -8.4761 | 2.33E-17 | 1.94E-16 | DOWN |
| FAM83B | 79.73197349 | -1.935283957 | 0.228568554 | -8.46697 | 2.52E-17 | 2.09E-16 | DOWN |
| ATP1A2 | 1968.194562 | -1.713017082 | 0.202377225 | -8.46448 | 2.57E-17 | 2.13E-16 | DOWN |
| KIF14 | 69.67943209 | 1.640278356 | 0.193783997 | 8.464468 | 2.57E-17 | 2.13E-16 | UP |
| SLC27A2 | 1026.960127 | 1.042538182 | 0.123254676 | 8.458407 | 2.71E-17 | 2.24E-16 | UP |
| DNAH8 | 1591.926607 | 2.322164632 | 0.2749251 | 8.446536 | 3.00E-17 | 2.48E-16 | UP |
| ENPP3 | 515.0762442 | -2.020450058 | 0.239307053 | -8.44292 | 3.10E-17 | 2.55E-16 | DOWN |
| CYP3A5 | 514.2775122 | -1.962152604 | 0.232449941 | -8.44118 | 3.14E-17 | 2.59E-16 | DOWN |
| FLNC | 19271.16482 | -1.832384159 | 0.217180013 | -8.43717 | 3.25E-17 | 2.67E-16 | DOWN |
| GATA5 | 165.4670763 | -1.829685697 | 0.216861702 | -8.43711 | 3.25E-17 | 2.67E-16 | DOWN |
| ITGB1BP2 | 119.39562 | -1.217299813 | 0.144331652 | -8.43405 | 3.34E-17 | 2.74E-16 | DOWN |
| CD248 | 1058.055217 | -1.089706083 | 0.129209711 | -8.43362 | 3.35E-17 | 2.74E-16 | DOWN |
| TRIB1 | 13136.14538 | 1.318369049 | 0.156332637 | 8.433102 | 3.37E-17 | 2.75E-16 | UP |
| TSPAN2 | 900.6788546 | -1.052271447 | 0.124782381 | -8.43285 | 3.37E-17 | 2.76E-16 | DOWN |
| TPO | 169.0308904 | 1.888014719 | 0.224019052 | 8.42792 | 3.52E-17 | 2.87E-16 | UP |
| TBX10 | 74.12269585 | 2.721739295 | 0.323236608 | 8.420269 | 3.76E-17 | 3.06E-16 | UP |
| NRP2 | 1466.530472 | -1.192247359 | 0.141637048 | -8.41762 | 3.84E-17 | 3.12E-16 | DOWN |
| DNASE1L2 | 90.48435315 | 1.364142154 | 0.162083944 | 8.416269 | 3.89E-17 | 3.16E-16 | UP |
| RSPH1 | 550.748457 | 1.222314438 | 0.14524249 | 8.415681 | 3.91E-17 | 3.17E-16 | UP |
| GJB3 | 151.434796 | -1.682126449 | 0.19989493 | -8.41505 | 3.93E-17 | 3.19E-16 | DOWN |
| ALDH3A1 | 67.14261033 | -1.633452501 | 0.194156156 | -8.41309 | 3.99E-17 | 3.24E-16 | DOWN |
| ATAD3C | 173.8998366 | -1.172798812 | 0.139417616 | -8.41213 | 4.03E-17 | 3.26E-16 | DOWN |
| PPFIA2 | 593.8371886 | 2.615488626 | 0.311051259 | 8.408545 | 4.15E-17 | 3.36E-16 | UP |
| HOXD8 | 323.9350067 | -1.249483816 | 0.148616474 | -8.40744 | 4.19E-17 | 3.38E-16 | DOWN |
| CELSR3 | 391.108564 | 1.78876899 | 0.212826641 | 8.404817 | 4.29E-17 | 3.46E-16 | UP |
| SLC23A3 | 47.07384121 | 1.432959617 | 0.170505127 | 8.404202 | 4.31E-17 | 3.47E-16 | UP |
| TSPAN1 | 54058.73311 | 1.055410956 | 0.125634338 | 8.400657 | 4.44E-17 | 3.58E-16 | UP |
| BCL2 | 1517.48417 | -1.08722216 | 0.129459247 | -8.39818 | 4.53E-17 | 3.65E-16 | DOWN |
| PLXNA4 | 175.9304054 | -1.290499814 | 0.153664354 | -8.39817 | 4.53E-17 | 3.65E-16 | DOWN |
| NCCRP1 | 64.57938648 | -1.76851285 | 0.210697022 | -8.39363 | 4.71E-17 | 3.78E-16 | DOWN |
| NUDT10 | 721.0832504 | -1.324663797 | 0.157994927 | -8.38422 | 5.11E-17 | 4.09E-16 | DOWN |
| EXO1 | 85.74733713 | 1.570843007 | 0.187594505 | 8.373609 | 5.59E-17 | 4.46E-16 | UP |
| PDLIM1 | 3819.999912 | -1.012166042 | 0.120940745 | -8.36911 | 5.81E-17 | 4.63E-16 | DOWN |
| ZNF560 | 98.04441066 | 3.773924589 | 0.451139967 | 8.365308 | 6.00E-17 | 4.78E-16 | UP |
| FLNA | 127003.0199 | -1.434004966 | 0.17151486 | -8.36082 | 6.23E-17 | 4.95E-16 | DOWN |
| COL9A2 | 16129.37229 | 2.112832533 | 0.252707032 | 8.360798 | 6.23E-17 | 4.95E-16 | UP |
| CLIC6 | 564.0380793 | -1.218839172 | 0.145826387 | -8.35815 | 6.37E-17 | 5.06E-16 | DOWN |
| SGCA | 615.3752129 | -1.30200478 | 0.155825749 | -8.35552 | 6.51E-17 | 5.17E-16 | DOWN |
| HAPLN2 | 103.5265131 | -1.289084164 | 0.154404647 | -8.34874 | 6.90E-17 | 5.46E-16 | DOWN |
| ETV5 | 650.4733436 | -1.091186499 | 0.130743068 | -8.34604 | 7.06E-17 | 5.57E-16 | DOWN |
| MAP1B | 6232.325212 | -1.2710081 | 0.152300714 | -8.34539 | 7.10E-17 | 5.60E-16 | DOWN |
| DGCR5 | 85.87811664 | 1.694165939 | 0.20302894 | 8.344455 | 7.15E-17 | 5.64E-16 | UP |

| | | | | | | | |
|---|---|---|---|---|---|---|---|
| NSUN5P2 | 116.7683275 | 1.084789414 | 0.130023935 | 8.342998 | 7.24E-17 | 5.70E-16 | UP |
| ITGB4 | 5058.646688 | -1.307173279 | 0.156763461 | -8.33851 | 7.52E-17 | 5.91E-16 | DOWN |
| PRLR | 711.5021748 | -1.762968017 | 0.211495038 | -8.33574 | 7.70E-17 | 6.04E-16 | DOWN |
| TOP2A | 1009.701363 | 1.481374203 | 0.177715992 | 8.335627 | 7.71E-17 | 6.04E-16 | UP |
| ANKRD30A | 33.25961567 | 6.161757247 | 0.739291304 | 8.334681 | 7.77E-17 | 6.09E-16 | UP |
| MNX1 | 190.2739892 | 2.207645447 | 0.264953159 | 8.33221 | 7.93E-17 | 6.21E-16 | UP |
| C9orf43 | 68.51427119 | 1.015626857 | 0.121915349 | 8.330591 | 8.04E-17 | 6.28E-16 | UP |
| TCEAL2 | 625.7247137 | -1.419976915 | 0.170457579 | -8.33038 | 8.06E-17 | 6.28E-16 | DOWN |
| CES1 | 1593.248603 | -1.379653909 | 0.165683829 | -8.32703 | 8.29E-17 | 6.45E-16 | DOWN |
| ITPR1 | 4631.307357 | -1.296263695 | 0.15573037 | -8.32377 | 8.52E-17 | 6.61E-16 | DOWN |
| CBX2 | 284.7442788 | 1.516149546 | 0.182196252 | 8.321519 | 8.68E-17 | 6.74E-16 | UP |
| KIF2C | 202.6094879 | 1.23585826 | 0.148647267 | 8.314033 | 9.25E-17 | 7.17E-16 | UP |
| WSCD2 | 325.8179609 | -1.462454623 | 0.175931441 | -8.31264 | 9.36E-17 | 7.24E-16 | DOWN |
| RBPMS | 4450.07756 | -1.11088872 | 0.133698771 | -8.30889 | 9.66E-17 | 7.46E-16 | DOWN |
| SLITRK2 | 54.52177006 | -1.95416551 | 0.235210003 | -8.30817 | 9.72E-17 | 7.50E-16 | DOWN |
| PYGL | 1492.237368 | -1.206447455 | 0.145243236 | -8.30639 | 9.87E-17 | 7.60E-16 | DOWN |
| LAMB3 | 3006.907989 | -1.738871123 | 0.209382663 | -8.30475 | 1.00E-16 | 7.70E-16 | DOWN |
| TPM2 | 33384.31049 | -1.396609038 | 0.168180924 | -8.30421 | 1.00E-16 | 7.74E-16 | DOWN |
| KRT7 | 4120.255292 | -1.962836473 | 0.2365775 | -8.2968 | 1.07E-16 | 8.21E-16 | DOWN |
| ISX | 178.5177274 | 1.815165572 | 0.21884914 | 8.294141 | 1.09E-16 | 8.39E-16 | UP |
| PTP4A3 | 5813.473087 | 1.259960385 | 0.15194026 | 8.292472 | 1.11E-16 | 8.50E-16 | UP |
| NKAIN1 | 1369.154187 | 2.235632837 | 0.269732914 | 8.28832 | 1.15E-16 | 8.79E-16 | UP |
| FEV | 1104.400912 | 1.714766996 | 0.207073251 | 8.280968 | 1.22E-16 | 9.32E-16 | UP |
| PCYT1B | 76.59587283 | -1.418255613 | 0.171317675 | -8.27851 | 1.25E-16 | 9.50E-16 | DOWN |
| NELL2 | 879.9654868 | -1.754462574 | 0.212020943 | -8.27495 | 1.29E-16 | 9.77E-16 | DOWN |
| NID1 | 2569.857848 | -1.115672383 | 0.134986082 | -8.26509 | 1.40E-16 | 1.06E-15 | DOWN |
| TAGLN | 59984.88198 | -1.277971132 | 0.15465616 | -8.26331 | 1.42E-16 | 1.07E-15 | DOWN |
| ZNF750 | 465.555455 | -1.618228402 | 0.195943017 | -8.25867 | 1.47E-16 | 1.11E-15 | DOWN |
| FOXL2 | 125.2394743 | 2.33439696 | 0.282762226 | 8.255689 | 1.51E-16 | 1.14E-15 | UP |
| DYSF | 885.6846523 | -1.00183804 | 0.121464057 | -8.24802 | 1.61E-16 | 1.22E-15 | DOWN |
| LGALS4 | 71.45522486 | -1.709522562 | 0.207780436 | -8.22754 | 1.91E-16 | 1.44E-15 | DOWN |
| SLC38A5 | 156.1725914 | -1.10319347 | 0.134141057 | -8.22413 | 1.97E-16 | 1.48E-15 | DOWN |
| INMT | 1491.220553 | -1.183686877 | 0.14396294 | -8.22216 | 2.00E-16 | 1.50E-15 | DOWN |
| ITGA5 | 4660.60765 | -1.237617458 | 0.150558707 | -8.22017 | 2.03E-16 | 1.52E-15 | DOWN |
| ANKRD35 | 308.9542753 | -1.017216975 | 0.123749285 | -8.21998 | 2.04E-16 | 1.52E-15 | DOWN |
| ARMC3 | 63.87291538 | 2.435169089 | 0.296391348 | 8.21606 | 2.10E-16 | 1.57E-15 | UP |
| ATP1B1 | 3846.913422 | -1.083149098 | 0.131947022 | -8.20897 | 2.23E-16 | 1.67E-15 | DOWN |
| ID1 | 2752.193853 | -1.558524434 | 0.189877226 | -8.20806 | 2.25E-16 | 1.68E-15 | DOWN |
| PCDH10 | 1303.765816 | -1.296609334 | 0.158031809 | -8.20474 | 2.31E-16 | 1.72E-15 | DOWN |
| MGAT5B | 105.983877 | 1.796369492 | 0.219108815 | 8.198527 | 2.43E-16 | 1.81E-15 | UP |
| SNHG9 | 440.3944815 | 1.174567109 | 0.143266967 | 8.19845 | 2.44E-16 | 1.81E-15 | UP |
| VSNL1 | 142.9571096 | -1.789469298 | 0.218302437 | -8.1972 | 2.46E-16 | 1.83E-15 | DOWN |
| MIPEP | 4588.331202 | 1.706267283 | 0.208318743 | 8.190657 | 2.60E-16 | 1.93E-15 | UP |
| LDB3 | 1538.276795 | -1.741326457 | 0.212755187 | -8.18465 | 2.73E-16 | 2.03E-15 | DOWN |
| PRIMA1 | 314.1913812 | -1.353948713 | 0.165439104 | -8.18397 | 2.75E-16 | 2.04E-15 | DOWN |
| HSD17B13 | 394.8516695 | -2.511581258 | 0.306922379 | -8.18312 | 2.77E-16 | 2.05E-15 | DOWN |
| C5orf30 | 1334.321096 | 1.125231394 | 0.137593528 | 8.177938 | 2.89E-16 | 2.14E-15 | UP |
| INPP5D | 831.6486899 | -1.050086204 | 0.128449094 | -8.17512 | 2.96E-16 | 2.18E-15 | DOWN |
| C4orf48 | 107.1022966 | 1.405586779 | 0.172046632 | 8.169801 | 3.09E-16 | 2.28E-15 | UP |
| MATN2 | 6620.656644 | -1.069663607 | 0.130943352 | -8.1689 | 3.11E-16 | 2.29E-15 | DOWN |
| DKK3 | 4216.997378 | -1.199830999 | 0.146896771 | -8.16785 | 3.14E-16 | 2.31E-15 | DOWN |
| CATSPER2 | 240.0919778 | 1.052194949 | 0.128855405 | 8.165703 | 3.20E-16 | 2.35E-15 | UP |
| PPEF1 | 47.46610156 | 1.505478032 | 0.184499869 | 8.159778 | 3.36E-16 | 2.46E-15 | UP |
| DCST2 | 121.78715 | 1.161575236 | 0.142407121 | 8.156722 | 3.44E-16 | 2.53E-15 | UP |
| LGALS1 | 6445.334024 | -1.067338463 | 0.130876236 | -8.15533 | 3.48E-16 | 2.55E-15 | DOWN |
| IER5 | 937.9270684 | -1.045459372 | 0.128327839 | -8.14679 | 3.74E-16 | 2.73E-15 | DOWN |
| AFF2 | 54.18726782 | -1.484334393 | 0.182355515 | -8.13978 | 3.96E-16 | 2.88E-15 | DOWN |
| TMOD1 | 447.0806983 | -1.011010994 | 0.124217386 | -8.13905 | 3.98E-16 | 2.89E-15 | DOWN |
| IL33 | 1283.162459 | -1.013194019 | 0.124487436 | -8.13893 | 3.99E-16 | 2.90E-15 | DOWN |
| SPARCL1 | 24483.84288 | -1.166488317 | 0.143378982 | -8.1357 | 4.10E-16 | 2.97E-15 | DOWN |
| CPNE6 | 153.9975169 | -2.256378 | 0.277433285 | -8.13305 | 4.19E-16 | 3.03E-15 | DOWN |
| GPX3 | 4030.271832 | -1.238557125 | 0.152292164 | -8.13277 | 4.20E-16 | 3.04E-15 | DOWN |
| SERPINB5 | 526.7886637 | -1.985881278 | 0.244271295 | -8.12982 | 4.30E-16 | 3.11E-15 | DOWN |

| | | | | | | | |
|---|---|---|---|---|---|---|---|
| APLN | 530.008914 | 1.515627558 | 0.186442326 | 8.129203 | 4.32E-16 | 3.12E-15 | UP |
| AURKA | 222.8403641 | 1.116510918 | 0.137382736 | 8.12701 | 4.40E-16 | 3.17E-15 | UP |
| PCDHGB1 | 184.1891516 | 1.722233952 | 0.211959789 | 8.125286 | 4.46E-16 | 3.22E-15 | UP |
| TRPC4 | 266.1959918 | -1.068510564 | 0.1316381 | -8.11703 | 4.78E-16 | 3.44E-15 | DOWN |
| GAS1 | 716.9776353 | -1.290705927 | 0.159036469 | -8.11579 | 4.83E-16 | 3.47E-15 | DOWN |
| MAPK8IP2 | 1272.497591 | 1.122728691 | 0.138362414 | 8.114405 | 4.88E-16 | 3.51E-15 | UP |
| SERPINB1 | 1283.851976 | -1.127254199 | 0.138982283 | -8.11078 | 5.03E-16 | 3.61E-15 | DOWN |
| FHL1 | 11193.32665 | -1.524864971 | 0.188015018 | -8.11034 | 5.05E-16 | 3.62E-15 | DOWN |
| HEY2 | 255.587628 | 1.323267618 | 0.163273465 | 8.104609 | 5.29E-16 | 3.79E-15 | UP |
| TRIM31 | 46.98699077 | -1.711306264 | 0.211178618 | -8.1036 | 5.34E-16 | 3.81E-15 | DOWN |
| KANK4 | 105.1978642 | -1.531031412 | 0.189157735 | -8.09394 | 5.78E-16 | 4.11E-15 | DOWN |
| WIF1 | 318.0986413 | -2.233650261 | 0.276011685 | -8.09259 | 5.84E-16 | 4.16E-15 | DOWN |
| TBX1 | 1116.226499 | 1.474259033 | 0.182251121 | 8.089163 | 6.01E-16 | 4.27E-15 | UP |
| SLC24A2 | 56.38624408 | 2.645687712 | 0.327240659 | 8.084838 | 6.22E-16 | 4.41E-15 | UP |
| ITGB6 | 699.3126918 | -1.484832689 | 0.18373661 | -8.08131 | 6.41E-16 | 4.53E-15 | DOWN |
| GAD1 | 109.1780298 | 1.370024906 | 0.169549425 | 8.080387 | 6.46E-16 | 4.56E-15 | UP |
| HCN2 | 104.8105235 | 1.422045089 | 0.176020161 | 8.078876 | 6.54E-16 | 4.61E-15 | UP |
| ADM2 | 2293.377159 | 1.006895898 | 0.12464493 | 8.078114 | 6.58E-16 | 4.64E-15 | UP |
| NEXN | 2556.418162 | -1.283102469 | 0.158846568 | -8.07762 | 6.60E-16 | 4.65E-15 | DOWN |
| ARMCX1 | 1226.343271 | -1.221323322 | 0.151205384 | -8.07725 | 6.62E-16 | 4.66E-15 | DOWN |
| OLFML2A | 962.3966984 | -1.033581998 | 0.127981277 | -8.07604 | 6.69E-16 | 4.71E-15 | DOWN |
| AQP3 | 7746.991764 | -1.149381783 | 0.142371509 | -8.07312 | 6.85E-16 | 4.81E-15 | DOWN |
| KCNB1 | 364.7249605 | -1.231065987 | 0.152493733 | -8.0729 | 6.87E-16 | 4.82E-15 | DOWN |
| POLQ | 74.68015416 | 1.442803812 | 0.179078043 | 8.056844 | 7.83E-16 | 5.49E-15 | UP |
| MYO6 | 12923.03175 | 1.094595643 | 0.135878182 | 8.055713 | 7.90E-16 | 5.53E-15 | UP |
| HOXA7 | 51.16317751 | -1.1416481 | 0.141815739 | -8.05022 | 8.26E-16 | 5.78E-15 | DOWN |
| KRT19 | 14595.86968 | -1.369504709 | 0.170144142 | -8.04909 | 8.34E-16 | 5.83E-15 | DOWN |
| SSPO | 439.4677244 | 1.363023137 | 0.169410288 | 8.045693 | 8.58E-16 | 5.98E-15 | UP |
| HIST1H2BG | 705.5566114 | 1.434068592 | 0.178453545 | 8.036089 | 9.28E-16 | 6.45E-15 | UP |
| CRYAB | 3775.946109 | -1.513313932 | 0.188357943 | -8.03425 | 9.42E-16 | 6.55E-15 | DOWN |
| SLC16A8 | 165.1382857 | 1.405945565 | 0.175016373 | 8.033223 | 9.49E-16 | 6.60E-15 | UP |
| CIT | 269.6482786 | 1.032016623 | 0.128503963 | 8.03101 | 9.67E-16 | 6.71E-15 | UP |
| SS18L2 | 1825.130909 | 1.018816097 | 0.126903287 | 8.028288 | 9.88E-16 | 6.86E-15 | UP |
| SSTR2 | 116.1143228 | -1.112118986 | 0.138582608 | -8.02495 | 1.02E-15 | 7.04E-15 | DOWN |
| RNFT2 | 101.1417934 | 1.250812646 | 0.155875036 | 8.024458 | 1.02E-15 | 7.06E-15 | UP |
| ESPL1 | 130.9879059 | 1.44821059 | 0.180484616 | 8.024011 | 1.02E-15 | 7.08E-15 | UP |
| CAMK1G | 85.34720127 | -1.228422281 | 0.153128168 | -8.02218 | 1.04E-15 | 7.19E-15 | DOWN |
| CLEC3B | 418.1816345 | -1.21111169 | 0.150978789 | -8.02173 | 1.04E-15 | 7.21E-15 | DOWN |
| CKAP2L | 88.90232473 | 1.492623778 | 0.186237947 | 8.014606 | 1.10E-15 | 7.62E-15 | UP |
| APOBEC3F | 345.5666321 | -1.04282494 | 0.130187196 | -8.0102 | 1.15E-15 | 7.88E-15 | DOWN |
| PHLDA1 | 1623.24897 | -1.151158283 | 0.143754544 | -8.0078 | 1.17E-15 | 8.02E-15 | DOWN |
| RAD51 | 137.9043488 | 1.237716181 | 0.15458933 | 8.006479 | 1.18E-15 | 8.09E-15 | UP |
| DNAJC12 | 575.316845 | 1.368310142 | 0.171075332 | 7.99829 | 1.26E-15 | 8.63E-15 | UP |
| ACTA2 | 54009.82091 | -1.250117822 | 0.156353555 | -7.99546 | 1.29E-15 | 8.82E-15 | DOWN |
| RCAN2 | 1379.562567 | -1.090441123 | 0.136526278 | -7.98704 | 1.38E-15 | 9.42E-15 | DOWN |
| CSDC2 | 529.4201726 | -1.148141657 | 0.143862079 | -7.98085 | 1.45E-15 | 9.89E-15 | DOWN |
| FOXS1 | 119.2576882 | 1.217907484 | 0.152608659 | 7.980592 | 1.46E-15 | 9.91E-15 | UP |
| IL1RN | 164.5539072 | -1.422985977 | 0.178314453 | -7.98021 | 1.46E-15 | 9.93E-15 | DOWN |
| PLAC9 | 282.6995171 | -1.556325652 | 0.195179551 | -7.97382 | 1.54E-15 | 1.04E-14 | DOWN |
| GIPC2 | 120.633452 | -1.021204378 | 0.128152528 | -7.96866 | 1.60E-15 | 1.09E-14 | DOWN |
| AMPH | 77.9201366 | -1.006472719 | 0.126325211 | -7.96731 | 1.62E-15 | 1.10E-14 | DOWN |
| LGI3 | 97.05048671 | -1.475595815 | 0.185510942 | -7.95423 | 1.80E-15 | 1.22E-14 | DOWN |
| HES6 | 479.3757171 | 1.418697432 | 0.178381798 | 7.953151 | 1.82E-15 | 1.23E-14 | UP |
| SLC4A11 | 248.9269276 | -1.029064065 | 0.129399544 | -7.95261 | 1.83E-15 | 1.23E-14 | DOWN |
| HSD11B1 | 224.1047246 | -1.25464473 | 0.157931795 | -7.94422 | 1.95E-15 | 1.32E-14 | DOWN |
| MDH1B | 149.2241278 | 1.405118723 | 0.177038943 | 7.936778 | 2.08E-15 | 1.40E-14 | UP |
| KCNH8 | 296.7638271 | 1.953188986 | 0.246118852 | 7.935958 | 2.09E-15 | 1.41E-14 | UP |
| FAM71F2 | 92.8444809 | 1.175275868 | 0.148098078 | 7.935794 | 2.09E-15 | 1.41E-14 | UP |
| SLC14A1 | 3905.321448 | -1.865700043 | 0.235139521 | -7.93444 | 2.11E-15 | 1.42E-14 | DOWN |
| AOC2 | 68.17624646 | 1.142890663 | 0.144172348 | 7.927253 | 2.24E-15 | 1.50E-14 | UP |
| SYCE1L | 363.945665 | 1.49091913 | 0.188194284 | 7.922234 | 2.33E-15 | 1.56E-14 | UP |
| STK33 | 174.1279345 | -1.079525046 | 0.136277649 | -7.92151 | 2.35E-15 | 1.57E-14 | DOWN |
| HRH2 | 54.79341031 | -1.296868697 | 0.164117956 | -7.90205 | 2.74E-15 | 1.82E-14 | DOWN |

| | | | | | | | |
|---|---|---|---|---|---|---|---|
| SERPINA11 | 296.2952687 | 2.450074812 | 0.310194633 | 7.898508 | 2.82E-15 | 1.87E-14 | UP |
| KCNAB3 | 94.39997986 | 1.151489203 | 0.145854355 | 7.894788 | 2.91E-15 | 1.93E-14 | UP |
| TP63 | 1707.155373 | -1.78699374 | 0.226444384 | -7.89153 | 2.98E-15 | 1.98E-14 | DOWN |
| GJA3 | 96.73821587 | 2.207268862 | 0.279833805 | 7.887785 | 3.08E-15 | 2.04E-14 | UP |
| PTCHD1 | 246.8712868 | -1.340249894 | 0.169918554 | -7.8876 | 3.08E-15 | 2.04E-14 | DOWN |
| ASF1B | 247.8880871 | 1.27884695 | 0.162306478 | 7.879211 | 3.29E-15 | 2.17E-14 | UP |
| NAALADL2 | 3747.445059 | 1.038311872 | 0.131780253 | 7.879116 | 3.30E-15 | 2.17E-14 | UP |
| GRPR | 148.4327385 | 2.232622219 | 0.283809985 | 7.866609 | 3.64E-15 | 2.39E-14 | UP |
| WFDC1 | 2076.651659 | -1.256775925 | 0.159809223 | -7.86423 | 3.71E-15 | 2.44E-14 | DOWN |
| RPL36A | 5550.929539 | 1.123323905 | 0.142877323 | 7.862157 | 3.78E-15 | 2.47E-14 | UP |
| DLK2 | 546.0941387 | -1.350135531 | 0.171727173 | -7.8621 | 3.78E-15 | 2.47E-14 | DOWN |
| C3orf35 | 47.02679589 | 1.204785349 | 0.153273671 | 7.860354 | 3.83E-15 | 2.50E-14 | UP |
| TMC5 | 6055.341501 | 1.154455847 | 0.147132962 | 7.846344 | 4.28E-15 | 2.79E-14 | UP |
| PACSIN1 | 233.5711022 | 1.032695597 | 0.131633168 | 7.845254 | 4.32E-15 | 2.82E-14 | UP |
| PCDHA1 | 67.5596812 | 2.919010323 | 0.372102135 | 7.844648 | 4.34E-15 | 2.83E-14 | UP |
| C1QTNF9B | 69.96125725 | 2.265538364 | 0.288810689 | 7.844372 | 4.35E-15 | 2.83E-14 | UP |
| SPATA18 | 414.8414887 | -1.111551284 | 0.141741272 | -7.84211 | 4.43E-15 | 2.88E-14 | DOWN |
| NUDT8 | 2186.316795 | 1.534536023 | 0.195814092 | 7.836699 | 4.63E-15 | 3.01E-14 | UP |
| BCHE | 304.8611319 | -1.671539358 | 0.213320278 | -7.83582 | 4.66E-15 | 3.03E-14 | DOWN |
| CACNB1 | 323.5792592 | -1.02427386 | 0.130751608 | -7.83374 | 4.74E-15 | 3.07E-14 | DOWN |
| REEP1 | 536.9877286 | -1.102818752 | 0.140805967 | -7.83219 | 4.79E-15 | 3.11E-14 | DOWN |
| MUC6 | 5217.745362 | -3.971842618 | 0.507166614 | -7.83144 | 4.82E-15 | 3.13E-14 | DOWN |
| SGPP2 | 327.8875075 | -1.400089966 | 0.178801429 | -7.83042 | 4.86E-15 | 3.15E-14 | DOWN |
| LRMP | 159.6674778 | -1.099476716 | 0.140630419 | -7.8182 | 5.36E-15 | 3.46E-14 | DOWN |
| DSC3 | 1218.925326 | -1.554592516 | 0.199052073 | -7.80998 | 5.72E-15 | 3.68E-14 | DOWN |
| ENTPD5 | 21893.96488 | 1.143287387 | 0.146430492 | 7.807714 | 5.82E-15 | 3.74E-14 | UP |
| CPNE7 | 488.2435865 | 1.682222483 | 0.215669401 | 7.800005 | 6.19E-15 | 3.97E-14 | UP |
| AADAT | 1470.755619 | 1.005342726 | 0.128892236 | 7.79987 | 6.20E-15 | 3.97E-14 | UP |
| EGLN3 | 586.6668747 | -1.095371622 | 0.140515472 | -7.79538 | 6.42E-15 | 4.11E-14 | DOWN |
| MADCAM1 | 48.65919997 | 1.354634164 | 0.174319524 | 7.770984 | 7.79E-15 | 4.95E-14 | UP |
| TMEM191A | 173.4518752 | 1.005260564 | 0.129601045 | 7.756578 | 8.73E-15 | 5.53E-14 | UP |
| CCDC80 | 4276.982234 | -1.306778093 | 0.168489296 | -7.75585 | 8.78E-15 | 5.55E-14 | DOWN |
| HSPB6 | 4818.575107 | -1.582621025 | 0.204274652 | -7.74752 | 9.37E-15 | 5.92E-14 | DOWN |
| MCCC2 | 24180.63623 | 1.231429555 | 0.159034012 | 7.743184 | 9.70E-15 | 6.12E-14 | UP |
| COL6A2 | 25901.33281 | -1.003271683 | 0.12963639 | -7.73912 | 1.00E-14 | 6.31E-14 | DOWN |
| TP53AIP1 | 50.01842349 | -1.318568543 | 0.170379467 | -7.73901 | 1.00E-14 | 6.31E-14 | DOWN |
| SP5 | 569.7151364 | 1.559779114 | 0.201655286 | 7.734878 | 1.04E-14 | 6.52E-14 | UP |
| THBS4 | 3843.986242 | 1.618095171 | 0.20924669 | 7.732955 | 1.05E-14 | 6.61E-14 | UP |
| PKMYT1 | 244.4860371 | 1.229786431 | 0.159100032 | 7.729643 | 1.08E-14 | 6.77E-14 | UP |
| CLIC3 | 107.3270338 | -1.034074141 | 0.133903877 | -7.72251 | 1.14E-14 | 7.14E-14 | DOWN |
| ELF5 | 652.7005656 | -1.843910694 | 0.238811049 | -7.72121 | 1.15E-14 | 7.21E-14 | DOWN |
| CHIT1 | 621.2698143 | 2.197568706 | 0.284839436 | 7.715114 | 1.21E-14 | 7.55E-14 | UP |
| CCND2 | 3236.07779 | -1.193729011 | 0.154733467 | -7.71474 | 1.21E-14 | 7.57E-14 | DOWN |
| KRT5 | 9706.229432 | -1.812272692 | 0.235005451 | -7.71162 | 1.24E-14 | 7.75E-14 | DOWN |
| CA4 | 109.4504361 | -1.727141777 | 0.224037562 | -7.70916 | 1.27E-14 | 7.90E-14 | DOWN |
| ABCC3 | 761.5335712 | -1.279218677 | 0.165982161 | -7.70696 | 1.29E-14 | 8.03E-14 | DOWN |
| RELN | 1132.499735 | 1.754488863 | 0.227771486 | 7.702847 | 1.33E-14 | 8.29E-14 | UP |
| IL20RB | 56.28290324 | -1.284106254 | 0.166708451 | -7.70271 | 1.33E-14 | 8.29E-14 | DOWN |
| CRHBP | 55.05380032 | -1.354426783 | 0.17613694 | -7.68962 | 1.48E-14 | 9.17E-14 | DOWN |
| SEMA5A | 844.9299856 | -1.120821276 | 0.145880199 | -7.68316 | 1.55E-14 | 9.62E-14 | DOWN |
| EDIL3 | 741.4414908 | -1.112742577 | 0.144882726 | -7.6803 | 1.59E-14 | 9.83E-14 | DOWN |
| RAB3B | 13147.54531 | 1.008574673 | 0.131321455 | 7.680197 | 1.59E-14 | 9.83E-14 | UP |
| PABPC1L | 721.9632428 | 1.25572035 | 0.163522881 | 7.679172 | 1.60E-14 | 9.90E-14 | UP |
| CAMK1D | 961.4609464 | -1.043804965 | 0.135934308 | -7.67875 | 1.61E-14 | 9.93E-14 | DOWN |
| FCGBP | 2405.846406 | -1.920581748 | 0.250321453 | -7.67246 | 1.69E-14 | 1.04E-13 | DOWN |
| SLC2A5 | 868.8672953 | -1.83151361 | 0.239048823 | -7.66167 | 1.84E-14 | 1.13E-13 | DOWN |
| LGALS3 | 6798.705481 | -1.045974911 | 0.136569281 | -7.65893 | 1.87E-14 | 1.15E-13 | DOWN |
| DPP6 | 55.01047988 | -2.222320658 | 0.290481346 | -7.65048 | 2.00E-14 | 1.22E-13 | DOWN |
| DGKG | 112.1074122 | -1.327450504 | 0.173545648 | -7.649 | 2.03E-14 | 1.24E-13 | DOWN |
| DNASE2B | 669.3032618 | 1.643689467 | 0.2149805 | 7.645761 | 2.08E-14 | 1.27E-13 | UP |
| LRP1B | 64.79064653 | -1.605650113 | 0.210247894 | -7.63694 | 2.22E-14 | 1.35E-13 | DOWN |
| KIAA1210 | 736.0809871 | -1.832158933 | 0.240069181 | -7.6318 | 2.32E-14 | 1.40E-13 | DOWN |
| ADRA1D | 167.4220074 | -1.775171877 | 0.232639456 | -7.63057 | 2.34E-14 | 1.42E-13 | DOWN |

| | | | | | | | |
|---|---|---|---|---|---|---|---|
| FOXP2 | 229.3123883 | -1.13696806 | 0.149015353 | -7.62987 | 2.35E-14 | 1.42E-13 | DOWN |
| CXCL11 | 501.5415486 | 1.5638467 | 0.204983902 | 7.62912 | 2.36E-14 | 1.43E-13 | UP |
| CST6 | 56.94470369 | -2.231836506 | 0.292681839 | -7.62547 | 2.43E-14 | 1.47E-13 | DOWN |
| ITGAX | 487.2432039 | 1.024507714 | 0.134428226 | 7.621225 | 2.51E-14 | 1.52E-13 | UP |
| F10 | 400.7156225 | -1.08246797 | 0.142090576 | -7.61815 | 2.57E-14 | 1.55E-13 | DOWN |
| ITGA7 | 2228.123334 | -1.079561965 | 0.141710187 | -7.6181 | 2.57E-14 | 1.55E-13 | DOWN |
| FBXO2 | 587.4365572 | -1.033783645 | 0.135722165 | -7.61691 | 2.60E-14 | 1.56E-13 | DOWN |
| ACTG2 | 60879.13058 | -1.436430377 | 0.188685074 | -7.61285 | 2.68E-14 | 1.61E-13 | DOWN |
| SV2C | 301.2401806 | 1.142172128 | 0.150062081 | 7.611331 | 2.71E-14 | 1.63E-13 | UP |
| KLK14 | 287.1705356 | 2.206013131 | 0.289871467 | 7.610315 | 2.73E-14 | 1.64E-13 | UP |
| CPA6 | 203.9286334 | -1.827825169 | 0.24034606 | -7.60497 | 2.85E-14 | 1.71E-13 | DOWN |
| NDP | 173.5884011 | -1.50213758 | 0.197610993 | -7.60149 | 2.93E-14 | 1.75E-13 | DOWN |
| CHRNA5 | 234.8159188 | 1.314817312 | 0.173015258 | 7.59943 | 2.97E-14 | 1.78E-13 | UP |
| TMEM40 | 88.42112882 | -1.7191066 | 0.22627933 | -7.59728 | 3.02E-14 | 1.81E-13 | DOWN |
| GSTM2 | 835.6116166 | -1.233881685 | 0.162417076 | -7.59699 | 3.03E-14 | 1.81E-13 | DOWN |
| HIST2H2BF | 316.84665 | 1.355050789 | 0.178442604 | 7.593763 | 3.11E-14 | 1.86E-13 | UP |
| ZEB2 | 1518.212627 | -1.009423749 | 0.132976674 | -7.59098 | 3.17E-14 | 1.89E-13 | DOWN |
| HUNK | 197.3311366 | -1.236167632 | 0.162935674 | -7.58684 | 3.28E-14 | 1.95E-13 | DOWN |
| LRFN5 | 137.3888415 | -1.151911522 | 0.151936117 | -7.58155 | 3.41E-14 | 2.03E-13 | DOWN |
| HLA-DMB | 3714.690523 | 1.709494166 | 0.225578519 | 7.578267 | 3.50E-14 | 2.08E-13 | UP |
| CELF5 | 112.4283034 | 1.65121806 | 0.217922654 | 7.577083 | 3.53E-14 | 2.09E-13 | UP |
| TMEM145 | 74.87417791 | 1.575333667 | 0.20809402 | 7.570298 | 3.72E-14 | 2.20E-13 | UP |
| SFRP4 | 4336.480242 | 1.566728983 | 0.206971362 | 7.569786 | 3.74E-14 | 2.21E-13 | UP |
| OLR1 | 199.9501361 | 1.26965231 | 0.167870863 | 7.563268 | 3.93E-14 | 2.31E-13 | UP |
| SRMS | 375.8025891 | 1.549956393 | 0.204933896 | 7.563202 | 3.93E-14 | 2.31E-13 | UP |
| B3GALT2 | 190.0844346 | -1.336055355 | 0.17670768 | -7.56082 | 4.01E-14 | 2.36E-13 | DOWN |
| HIST1H2AE | 792.9194752 | 1.328813471 | 0.1757555 | 7.56058 | 4.01E-14 | 2.36E-13 | UP |
| SFTPA2 | 5189.70467 | 2.050436525 | 0.271307727 | 7.557605 | 4.11E-14 | 2.41E-13 | UP |
| CNTN1 | 1822.756282 | -1.209252635 | 0.160039252 | -7.55598 | 4.16E-14 | 2.44E-13 | DOWN |
| ZWINT | 601.1295382 | 1.005673497 | 0.13311063 | 7.55517 | 4.18E-14 | 2.45E-13 | UP |
| TTR | 144.3447592 | 2.221390943 | 0.29410112 | 7.553154 | 4.25E-14 | 2.49E-13 | UP |
| BVES | 560.0056816 | -1.021340535 | 0.135282246 | -7.5497 | 4.36E-14 | 2.55E-13 | DOWN |
| SYT9 | 146.1386436 | -1.658904453 | 0.220133736 | -7.53589 | 4.85E-14 | 2.82E-13 | DOWN |
| RND3 | 1822.014695 | -1.126663356 | 0.149822318 | -7.52 | 5.48E-14 | 3.18E-13 | DOWN |
| SDC1 | 6218.890423 | -1.135918508 | 0.151067828 | -7.51926 | 5.51E-14 | 3.20E-13 | DOWN |
| RGS9 | 161.9196377 | -1.238431301 | 0.16471729 | -7.51853 | 5.54E-14 | 3.21E-13 | DOWN |
| SERPINF1 | 5000.80027 | -1.08081522 | 0.143820751 | -7.51502 | 5.69E-14 | 3.30E-13 | DOWN |
| FOXF1 | 1153.553161 | -1.115204745 | 0.148409442 | -7.51438 | 5.72E-14 | 3.31E-13 | DOWN |
| IP6K3 | 136.1646126 | -1.740443913 | 0.231713468 | -7.51119 | 5.86E-14 | 3.39E-13 | DOWN |
| PPM1E | 944.8657513 | 1.354362669 | 0.180416725 | 7.506858 | 6.06E-14 | 3.50E-13 | UP |
| KLK12 | 548.309 | 2.305222514 | 0.307215207 | 7.503608 | 6.21E-14 | 3.58E-13 | UP |
| RNF165 | 221.9228789 | -1.201509741 | 0.160181999 | -7.5009 | 6.34E-14 | 3.65E-13 | DOWN |
| PILRB | 283.670978 | 1.321273535 | 0.176198734 | 7.498769 | 6.44E-14 | 3.70E-13 | UP |
| COMP | 3464.3706 | 1.933021918 | 0.257938181 | 7.494129 | 6.67E-14 | 3.83E-13 | UP |
| PCDH9 | 233.3955407 | -1.170820815 | 0.156486146 | -7.48195 | 7.32E-14 | 4.18E-13 | DOWN |
| MASP1 | 2635.29323 | -1.45624261 | 0.194646439 | -7.48148 | 7.35E-14 | 4.20E-13 | DOWN |
| ASTN1 | 60.03323478 | -1.996207395 | 0.266862074 | -7.4803 | 7.42E-14 | 4.23E-13 | DOWN |
| PCDHGA5 | 405.1684436 | 1.72104666 | 0.230088409 | 7.479936 | 7.44E-14 | 4.24E-13 | UP |
| NTRK1 | 51.94334834 | -1.237773879 | 0.165482902 | -7.47977 | 7.45E-14 | 4.25E-13 | DOWN |
| RAB26 | 685.125397 | 1.034209335 | 0.138434968 | 7.470723 | 7.98E-14 | 4.54E-13 | UP |
| FGF2 | 651.9408693 | -1.045038384 | 0.139912481 | -7.46923 | 8.07E-14 | 4.59E-13 | DOWN |
| LILRB4 | 303.9737627 | 1.012724228 | 0.135644506 | 7.466017 | 8.27E-14 | 4.70E-13 | UP |
| RARRES2 | 2648.177288 | -1.088984436 | 0.146300756 | -7.44346 | 9.81E-14 | 5.55E-13 | DOWN |
| SLC17A4 | 31.23985798 | 4.076259183 | 0.547648573 | 7.443202 | 9.83E-14 | 5.56E-13 | UP |
| ADRA2A | 809.7080512 | 1.407669343 | 0.189128503 | 7.442925 | 9.85E-14 | 5.57E-13 | UP |
| TMEM52 | 188.5900941 | 1.101642295 | 0.148109843 | 7.438009 | 1.02E-13 | 5.77E-13 | UP |
| EMILIN1 | 6866.635666 | -1.043216638 | 0.140256695 | -7.43791 | 1.02E-13 | 5.77E-13 | DOWN |
| CDKN2A | 218.5904263 | 1.151958888 | 0.154914309 | 7.436104 | 1.04E-13 | 5.84E-13 | UP |
| RSPH4A | 69.56485467 | 1.405857305 | 0.189114373 | 7.4339 | 1.05E-13 | 5.93E-13 | UP |
| NRK | 256.4869358 | -1.502679598 | 0.202159236 | -7.43315 | 1.06E-13 | 5.96E-13 | DOWN |
| BANK1 | 2104.48993 | 1.742172602 | 0.234386261 | 7.432913 | 1.06E-13 | 5.97E-13 | UP |
| HMGCLL1 | 62.01411548 | -1.65135301 | 0.222611795 | -7.41808 | 1.19E-13 | 6.65E-13 | DOWN |
| SULT4A1 | 46.61510283 | 2.553250068 | 0.344292273 | 7.415938 | 1.21E-13 | 6.75E-13 | UP |

| | | | | | | | |
|---|---|---|---|---|---|---|---|
| VSTM2L | 7682.556477 | 1.496825413 | 0.202181868 | 7.403361 | 1.33E-13 | 7.41E-13 | UP |
| TBX5 | 167.3047121 | -1.702586335 | 0.23000331 | -7.40244 | 1.34E-13 | 7.46E-13 | DOWN |
| DAB1 | 265.7839041 | -1.521184257 | 0.205719365 | -7.39446 | 1.42E-13 | 7.90E-13 | DOWN |
| DNAJC15 | 925.7103737 | -1.187332926 | 0.160581823 | -7.39394 | 1.43E-13 | 7.93E-13 | DOWN |
| FAT4 | 708.3933113 | -1.179821858 | 0.159625501 | -7.39119 | 1.46E-13 | 8.09E-13 | DOWN |
| PCDHB16 | 465.1872522 | 1.449934778 | 0.196283861 | 7.386928 | 1.50E-13 | 8.34E-13 | UP |
| SIX2 | 171.3840167 | -1.38064166 | 0.186916199 | -7.38642 | 1.51E-13 | 8.37E-13 | DOWN |
| SMS | 26222.90365 | 1.309756208 | 0.177330645 | 7.385955 | 1.51E-13 | 8.39E-13 | UP |
| GPM6A | 321.1354768 | -1.53459396 | 0.20777686 | -7.38578 | 1.52E-13 | 8.40E-13 | DOWN |
| TRIM29 | 3903.83075 | -1.695038734 | 0.229963484 | -7.3709 | 1.69E-13 | 9.35E-13 | DOWN |
| SYCP2L | 69.55435428 | 1.935118729 | 0.262596453 | 7.369173 | 1.72E-13 | 9.46E-13 | UP |
| C1orf53 | 124.0409049 | 1.004178908 | 0.136280799 | 7.368455 | 1.73E-13 | 9.50E-13 | UP |
| PLN | 1237.948118 | -1.179694929 | 0.160151307 | -7.36613 | 1.76E-13 | 9.66E-13 | DOWN |
| CCDC3 | 1261.101919 | -1.135351567 | 0.154247363 | -7.36059 | 1.83E-13 | 1.00E-12 | DOWN |
| COL6A1 | 13311.06789 | -1.015686532 | 0.138002294 | -7.35992 | 1.84E-13 | 1.01E-12 | DOWN |
| NTN4 | 3924.753796 | -1.039248815 | 0.14135274 | -7.35217 | 1.95E-13 | 1.07E-12 | DOWN |
| SLC8A2 | 98.48933837 | -1.339603471 | 0.182410434 | -7.3439 | 2.07E-13 | 1.13E-12 | DOWN |
| FGF7 | 629.7193356 | -1.322536402 | 0.1801635 | -7.34076 | 2.12E-13 | 1.16E-12 | DOWN |
| FAM181B | 61.59627066 | -1.050407117 | 0.143137816 | -7.33843 | 2.16E-13 | 1.18E-12 | DOWN |
| CHP2 | 101.8570592 | -2.135783462 | 0.291195867 | -7.33453 | 2.23E-13 | 1.21E-12 | DOWN |
| CTLA4 | 59.4073848 | 1.435988631 | 0.195788031 | 7.334405 | 2.23E-13 | 1.21E-12 | UP |
| TDRD6 | 910.7038484 | 1.303576914 | 0.177885624 | 7.328175 | 2.33E-13 | 1.27E-12 | UP |
| DPT | 865.2221381 | -1.710794429 | 0.233621921 | -7.32292 | 2.43E-13 | 1.32E-12 | DOWN |
| HCG27 | 80.59721331 | 1.122401981 | 0.153289954 | 7.322084 | 2.44E-13 | 1.32E-12 | UP |
| MIAT | 110.4833933 | 1.42777064 | 0.195021081 | 7.321109 | 2.46E-13 | 1.33E-12 | UP |
| SCNN1A | 4135.878126 | -1.223700348 | 0.16724154 | -7.31696 | 2.54E-13 | 1.37E-12 | DOWN |
| CSPG5 | 173.1794364 | 1.200721042 | 0.164281093 | 7.308942 | 2.69E-13 | 1.45E-12 | UP |
| TCEAL7 | 181.8810932 | -1.013860411 | 0.138741709 | -7.30754 | 2.72E-13 | 1.46E-12 | DOWN |
| ANXA1 | 8620.338856 | -1.00195882 | 0.137148977 | -7.30562 | 2.76E-13 | 1.48E-12 | DOWN |
| MYO1G | 485.8021125 | 1.034449832 | 0.141639933 | 7.303377 | 2.81E-13 | 1.51E-12 | UP |
| FASN | 98520.81753 | 1.218700436 | 0.167009565 | 7.297189 | 2.94E-13 | 1.57E-12 | UP |
| HOXD11 | 126.1001604 | -1.391874429 | 0.190947394 | -7.28931 | 3.12E-13 | 1.66E-12 | DOWN |
| RFX6 | 114.1919707 | 2.187601149 | 0.300266109 | 7.285541 | 3.20E-13 | 1.71E-12 | UP |
| SCGB1A1 | 803.0330635 | -2.509303526 | 0.344788639 | -7.2778 | 3.39E-13 | 1.81E-12 | DOWN |
| RSPO3 | 330.2364992 | -1.319477008 | 0.181329277 | -7.27669 | 3.42E-13 | 1.82E-12 | DOWN |
| TBX4 | 249.1621803 | -2.239465372 | 0.307768094 | -7.27647 | 3.43E-13 | 1.82E-12 | DOWN |
| S1PR5 | 92.13122779 | -1.236072509 | 0.169872981 | -7.27645 | 3.43E-13 | 1.82E-12 | DOWN |
| MYB | 540.5402988 | 1.028329758 | 0.141373106 | 7.273871 | 3.49E-13 | 1.85E-12 | UP |
| PNMA2 | 67.41332496 | -1.308719097 | 0.179938275 | -7.27316 | 3.51E-13 | 1.86E-12 | DOWN |
| PCSK2 | 57.06906377 | -1.721146158 | 0.236756492 | -7.26969 | 3.60E-13 | 1.91E-12 | DOWN |
| BDNF | 111.7332969 | -1.187099164 | 0.163385426 | -7.26564 | 3.71E-13 | 1.96E-12 | DOWN |
| CRYBA2 | 86.27043548 | 1.419570174 | 0.195399416 | 7.264966 | 3.73E-13 | 1.97E-12 | UP |
| ACOT11 | 159.0364992 | -1.065080483 | 0.146956283 | -7.2476 | 4.24E-13 | 2.24E-12 | DOWN |
| DPY19L2P4 | 108.4469678 | 1.196590736 | 0.165323879 | 7.237858 | 4.56E-13 | 2.40E-12 | UP |
| OGN | 1984.294578 | -1.249215009 | 0.172639305 | -7.23598 | 4.62E-13 | 2.43E-12 | DOWN |
| SLC26A5 | 96.30469796 | 1.267100274 | 0.17524551 | 7.230429 | 4.81E-13 | 2.53E-12 | UP |
| KCNJ3 | 290.43828 | -1.896073342 | 0.262344757 | -7.22741 | 4.92E-13 | 2.58E-12 | DOWN |
| HIST1H4H | 469.4746237 | 1.264928988 | 0.175074804 | 7.225077 | 5.01E-13 | 2.62E-12 | UP |
| WNT2B | 568.5586723 | -1.166095283 | 0.161430556 | -7.22351 | 5.07E-13 | 2.65E-12 | DOWN |
| GGT6 | 466.9485108 | -1.409091014 | 0.195153919 | -7.22041 | 5.18E-13 | 2.71E-12 | DOWN |
| PTPRR | 59.29344733 | 2.018868586 | 0.279607393 | 7.220369 | 5.18E-13 | 2.71E-12 | UP |
| TGFB3 | 3433.883115 | -1.259854339 | 0.174571392 | -7.21684 | 5.32E-13 | 2.77E-12 | DOWN |
| APOE | 6193.277171 | 1.221864783 | 0.169352979 | 7.2149 | 5.40E-13 | 2.81E-12 | UP |
| PRDM6 | 209.5276066 | -1.108334964 | 0.153631191 | -7.21426 | 5.42E-13 | 2.83E-12 | DOWN |
| UPK1A | 280.7872286 | -1.858018557 | 0.257614215 | -7.21241 | 5.50E-13 | 2.86E-12 | DOWN |
| SLC6A17 | 85.11800248 | 1.753385165 | 0.243186151 | 7.210054 | 5.59E-13 | 2.91E-12 | UP |
| MYH11 | 192671.1798 | -1.579674983 | 0.219265228 | -7.2044 | 5.83E-13 | 3.03E-12 | DOWN |
| LSAMP | 2817.780794 | -1.348825643 | 0.187338263 | -7.19995 | 6.02E-13 | 3.12E-12 | DOWN |
| THSD4 | 6157.220105 | -1.050873309 | 0.145980312 | -7.19873 | 6.08E-13 | 3.15E-12 | DOWN |
| ADPRHL1 | 428.010299 | 1.142687573 | 0.158774805 | 7.196907 | 6.16E-13 | 3.19E-12 | UP |
| FADS2 | 10088.95049 | -1.588791749 | 0.221039445 | -7.18782 | 6.58E-13 | 3.40E-12 | DOWN |
| ISG15 | 3046.628549 | 1.573461092 | 0.219027093 | 7.183865 | 6.78E-13 | 3.49E-12 | UP |
| SORCS2 | 312.4184564 | -1.119878448 | 0.155894323 | -7.18357 | 6.79E-13 | 3.50E-12 | DOWN |

| | | | | | | | |
|---|---|---|---|---|---|---|---|
| PYGM | 856.3643835 | -1.438941932 | 0.200420564 | -7.17961 | 6.99E-13 | 3.60E-12 | DOWN |
| ZNF556 | 55.49733357 | 2.590794346 | 0.360864791 | 7.179405 | 7.00E-13 | 3.60E-12 | UP |
| ADH1B | 245.1701239 | -1.491927581 | 0.208043824 | -7.17122 | 7.43E-13 | 3.82E-12 | DOWN |
| HIST1H3H | 512.4071326 | 1.441473484 | 0.201236421 | 7.163084 | 7.89E-13 | 4.04E-12 | UP |
| MON1B | 11218.44123 | 1.426890347 | 0.19938702 | 7.156385 | 8.28E-13 | 4.23E-12 | UP |
| HSPB7 | 1257.310605 | -1.350088774 | 0.188833591 | -7.14962 | 8.70E-13 | 4.44E-12 | DOWN |
| SPON2 | 84131.1065 | 1.772893137 | 0.248163287 | 7.144059 | 9.06E-13 | 4.62E-12 | UP |
| SPZ1 | 2.670610508 | 3.609295749 | 0.505402874 | 7.141423 | 9.24E-13 | 4.70E-12 | UP |
| ADCYAP1R1 | 67.23647635 | -1.478398434 | 0.207147856 | -7.13692 | 9.54E-13 | 4.84E-12 | DOWN |
| PI16 | 753.6500146 | -2.062985991 | 0.289197484 | -7.13349 | 9.79E-13 | 4.96E-12 | DOWN |
| NGFR | 1253.886783 | -1.092435783 | 0.15316695 | -7.13232 | 9.87E-13 | 5.00E-12 | DOWN |
| SLC2A4 | 545.7289704 | -1.254083289 | 0.176584092 | -7.1019 | 1.23E-12 | 6.18E-12 | DOWN |
| SV2B | 349.4205093 | -1.596101373 | 0.224817663 | -7.09954 | 1.25E-12 | 6.28E-12 | DOWN |
| MLC1 | 103.4458943 | -1.857538277 | 0.261830683 | -7.09443 | 1.30E-12 | 6.50E-12 | DOWN |
| MET | 1146.190343 | -1.00516299 | 0.141686804 | -7.09426 | 1.30E-12 | 6.51E-12 | DOWN |
| CAMK4 | 602.5888829 | -1.010042565 | 0.142382978 | -7.09384 | 1.30E-12 | 6.52E-12 | DOWN |
| LYPD6B | 404.973837 | -1.156163109 | 0.163013039 | -7.09246 | 1.32E-12 | 6.59E-12 | DOWN |
| SCUBE3 | 290.8798578 | -1.061109062 | 0.149725165 | -7.08705 | 1.37E-12 | 6.84E-12 | DOWN |
| TMPRSS4 | 262.7149285 | -1.893878548 | 0.267309549 | -7.08496 | 1.39E-12 | 6.93E-12 | DOWN |
| PDE5A | 4656.981163 | -1.114549802 | 0.157379491 | -7.08193 | 1.42E-12 | 7.08E-12 | DOWN |
| C15orf48 | 3410.679366 | 1.973754475 | 0.27874074 | 7.080969 | 1.43E-12 | 7.12E-12 | UP |
| NEAT1 | 58434.99751 | 1.241253692 | 0.175299218 | 7.080771 | 1.43E-12 | 7.13E-12 | UP |
| TUBB3 | 49.08761195 | 1.532017052 | 0.216762453 | 7.067723 | 1.57E-12 | 7.80E-12 | UP |
| DMKN | 620.8378158 | -1.171878726 | 0.165826741 | -7.06689 | 1.58E-12 | 7.84E-12 | DOWN |
| GPR87 | 222.8616913 | -1.65392405 | 0.234085359 | -7.06547 | 1.60E-12 | 7.91E-12 | DOWN |
| ASXL3 | 113.5213015 | -1.06525739 | 0.150934217 | -7.05776 | 1.69E-12 | 8.34E-12 | DOWN |
| ATCAY | 214.6456995 | -1.281934162 | 0.181702669 | -7.05512 | 1.72E-12 | 8.49E-12 | DOWN |
| PPP1R1A | 173.0504101 | -1.413401839 | 0.200529461 | -7.04835 | 1.81E-12 | 8.89E-12 | DOWN |
| VIT | 123.5374283 | -1.446164022 | 0.205402938 | -7.04062 | 1.91E-12 | 9.37E-12 | DOWN |
| CALML3 | 149.5062898 | -1.820530245 | 0.258677929 | -7.03783 | 1.95E-12 | 9.55E-12 | DOWN |
| PCDHB11 | 382.6923351 | 1.425993762 | 0.202667621 | 7.03612 | 1.98E-12 | 9.66E-12 | UP |
| ETS2 | 5772.367134 | -1.002044033 | 0.142425253 | -7.03558 | 1.98E-12 | 9.69E-12 | DOWN |
| MPZ | 127.9471651 | -1.495905824 | 0.212783112 | -7.03019 | 2.06E-12 | 1.01E-11 | DOWN |
| SEC14L5 | 195.0820644 | 1.230309737 | 0.175358668 | 7.015962 | 2.28E-12 | 1.11E-11 | UP |
| IL2RA | 69.04385854 | 1.192336036 | 0.169951884 | 7.015727 | 2.29E-12 | 1.11E-11 | UP |
| SMTNL2 | 68.09601184 | -1.743840636 | 0.248969433 | -7.00424 | 2.48E-12 | 1.20E-11 | DOWN |
| CBR3 | 647.9560375 | 1.086234463 | 0.155122896 | 7.002412 | 2.52E-12 | 1.21E-11 | UP |
| KRT4 | 357.1071442 | -2.197025927 | 0.31412535 | -6.99411 | 2.67E-12 | 1.29E-11 | DOWN |
| CHI3L1 | 366.6874668 | 1.465066153 | 0.209571308 | 6.990776 | 2.73E-12 | 1.32E-11 | UP |
| INHA | 48.7675804 | 1.099113921 | 0.157619338 | 6.973217 | 3.10E-12 | 1.49E-11 | UP |
| C4A | 978.1348662 | 1.41195979 | 0.202799782 | 6.962334 | 3.35E-12 | 1.60E-11 | UP |
| NEGR1 | 548.7781841 | -1.062312447 | 0.152587545 | -6.96199 | 3.36E-12 | 1.60E-11 | DOWN |
| KRT23 | 812.9338221 | -1.703412792 | 0.244760769 | -6.9595 | 3.41E-12 | 1.63E-11 | DOWN |
| CRYGS | 79.32372058 | 1.07653365 | 0.154866905 | 6.951347 | 3.62E-12 | 1.72E-11 | UP |
| ZNF536 | 78.5491606 | -1.460872696 | 0.210215276 | -6.94941 | 3.67E-12 | 1.74E-11 | DOWN |
| HIST4H4 | 349.9984746 | 1.30436364 | 0.188023633 | 6.937232 | 4.00E-12 | 1.90E-11 | UP |
| VAX2 | 84.94606401 | 1.443188559 | 0.208114714 | 6.934582 | 4.07E-12 | 1.93E-11 | UP |
| NOS1 | 208.7812645 | -1.473485282 | 0.212764602 | -6.92542 | 4.35E-12 | 2.06E-11 | DOWN |
| FXYD1 | 119.7003137 | -1.171663587 | 0.169206018 | -6.92448 | 4.38E-12 | 2.07E-11 | DOWN |
| CLNK | 82.25231898 | 1.843535138 | 0.266264966 | 6.923686 | 4.40E-12 | 2.08E-11 | UP |
| TOX3 | 646.7928615 | 1.145980259 | 0.165563249 | 6.921707 | 4.46E-12 | 2.11E-11 | UP |
| C2orf40 | 703.8474202 | -1.218963635 | 0.176138908 | -6.92047 | 4.50E-12 | 2.12E-11 | DOWN |
| SKAP1 | 152.7903436 | -1.044590263 | 0.15102468 | -6.91669 | 4.62E-12 | 2.18E-11 | DOWN |
| NTRK2 | 576.4662392 | -1.087330951 | 0.157406379 | -6.90779 | 4.92E-12 | 2.31E-11 | DOWN |
| C12orf60 | 248.9257995 | 1.172669843 | 0.169826986 | 6.905085 | 5.02E-12 | 2.35E-11 | UP |
| MCM10 | 67.47943964 | 1.325014568 | 0.19194588 | 6.903063 | 5.09E-12 | 2.38E-11 | UP |
| LEAP2 | 96.92266996 | 1.065213498 | 0.154357195 | 6.900964 | 5.17E-12 | 2.42E-11 | UP |
| RPRM | 180.1113274 | -1.069181322 | 0.154969359 | -6.89931 | 5.23E-12 | 2.44E-11 | DOWN |
| PCDHB8 | 142.9210252 | 2.018538961 | 0.292739091 | 6.895352 | 5.37E-12 | 2.51E-11 | UP |
| NPY6R | 92.58417475 | -2.087255438 | 0.302814585 | -6.89285 | 5.47E-12 | 2.55E-11 | DOWN |
| PCP4 | 3214.893667 | -1.363570294 | 0.197890789 | -6.89052 | 5.56E-12 | 2.59E-11 | DOWN |
| KLKP1 | 436.8564712 | 1.143970016 | 0.166039303 | 6.889754 | 5.59E-12 | 2.60E-11 | UP |
| ZFHX4 | 452.7849652 | -1.189157558 | 0.172769477 | -6.88291 | 5.86E-12 | 2.72E-11 | DOWN |

| | | | | | | | |
|---|---|---|---|---|---|---|---|
| MACC1 | 326.9269054 | -1.192582836 | 0.173659226 | -6.86737 | 6.54E-12 | 3.02E-11 | DOWN |
| SNORD17 | 19.76934531 | 2.093935357 | 0.305726647 | 6.849044 | 7.43E-12 | 3.42E-11 | UP |
| HIST1H2BF | 75.0570733 | 1.670279352 | 0.244069244 | 6.843465 | 7.73E-12 | 3.55E-11 | UP |
| RAD54L | 78.35495651 | 1.181180634 | 0.17272444 | 6.838526 | 8.00E-12 | 3.66E-11 | UP |
| LTBP4 | 17054.78124 | -1.013018177 | 0.148153674 | -6.83762 | 8.05E-12 | 3.68E-11 | DOWN |
| IGDCC4 | 48.07892602 | -1.454635871 | 0.212788316 | -6.83607 | 8.14E-12 | 3.72E-11 | DOWN |
| PRAME | 77.19087382 | 3.092519315 | 0.452719693 | 6.83098 | 8.43E-12 | 3.85E-11 | UP |
| NTN1 | 730.4829594 | -1.085412093 | 0.158949367 | -6.82867 | 8.57E-12 | 3.91E-11 | DOWN |
| HIST2H2AC | 54.01715613 | 1.050041283 | 0.153851597 | 6.825027 | 8.79E-12 | 4.01E-11 | UP |
| BMP6 | 514.1620111 | 1.37614143 | 0.201729474 | 6.821717 | 9.00E-12 | 4.09E-11 | UP |
| NEURL3 | 116.9673368 | -1.163098701 | 0.170551367 | -6.81964 | 9.13E-12 | 4.15E-11 | DOWN |
| ECT2L | 45.90903171 | 1.406006839 | 0.206220535 | 6.817977 | 9.23E-12 | 4.19E-11 | UP |
| ACE2 | 41.10876998 | -1.310566175 | 0.192342407 | -6.81371 | 9.51E-12 | 4.31E-11 | DOWN |
| SLC35F3 | 167.4075309 | 1.111397502 | 0.163286449 | 6.806428 | 1.00E-11 | 4.52E-11 | UP |
| CHD5 | 126.5059026 | -1.169177229 | 0.171919504 | -6.80072 | 1.04E-11 | 4.69E-11 | DOWN |
| CDC20B | 49.83039573 | 3.160061488 | 0.465035404 | 6.795314 | 1.08E-11 | 4.86E-11 | UP |
| OIP5 | 47.79882923 | 1.091525903 | 0.160758646 | 6.789843 | 1.12E-11 | 5.03E-11 | UP |
| EME1 | 58.78329491 | 1.040058318 | 0.153208222 | 6.788528 | 1.13E-11 | 5.07E-11 | UP |
| TMEM45B | 2329.975569 | 1.374556957 | 0.202630093 | 6.783578 | 1.17E-11 | 5.24E-11 | UP |
| HIST1H2BC | 859.6564846 | 1.247037108 | 0.183852467 | 6.782814 | 1.18E-11 | 5.27E-11 | UP |
| NRG1 | 138.3458352 | -1.222699221 | 0.180296939 | -6.78159 | 1.19E-11 | 5.31E-11 | DOWN |
| TNS4 | 1693.814398 | -1.63079789 | 0.240682435 | -6.77572 | 1.24E-11 | 5.51E-11 | DOWN |
| HNF1A | 64.76083267 | 2.9489663 | 0.435289162 | 6.774729 | 1.25E-11 | 5.55E-11 | UP |
| WDR93 | 209.4296546 | 1.160736655 | 0.171570142 | 6.765377 | 1.33E-11 | 5.91E-11 | UP |
| DLGAP2 | 102.8802201 | 1.371815848 | 0.202821653 | 6.763656 | 1.35E-11 | 5.97E-11 | UP |
| NCKAP5 | 272.4906194 | 1.034848555 | 0.15330923 | 6.750073 | 1.48E-11 | 6.54E-11 | UP |
| CENPI | 56.49269437 | 1.105957384 | 0.164196204 | 6.735584 | 1.63E-11 | 7.19E-11 | UP |
| CYP2W1 | 69.83811908 | -1.470116863 | 0.218534073 | -6.72717 | 1.73E-11 | 7.60E-11 | DOWN |
| MYT1 | 73.58613977 | 2.162391357 | 0.321541333 | 6.725081 | 1.75E-11 | 7.70E-11 | UP |
| SPINK8 | 62.12834638 | 1.814095158 | 0.269855239 | 6.722475 | 1.79E-11 | 7.83E-11 | UP |
| NRXN1 | 182.9902574 | -1.405555759 | 0.209254542 | -6.71697 | 1.86E-11 | 8.10E-11 | DOWN |
| CCL22 | 139.1727519 | 1.125300459 | 0.167662708 | 6.711692 | 1.92E-11 | 8.39E-11 | UP |
| MND1 | 54.99642074 | 1.201625918 | 0.1794759 | 6.695194 | 2.15E-11 | 9.36E-11 | UP |
| FAM189A1 | 274.7263034 | 1.256362593 | 0.187664235 | 6.694736 | 2.16E-11 | 9.38E-11 | UP |
| ZYG11A | 263.3954765 | 1.152534309 | 0.172386149 | 6.685771 | 2.30E-11 | 9.94E-11 | UP |
| CPXM1 | 499.6041564 | -1.130830258 | 0.169187257 | -6.6839 | 2.33E-11 | 1.01E-10 | DOWN |
| ALX4 | 81.49660207 | 2.05624143 | 0.307782671 | 6.680823 | 2.38E-11 | 1.03E-10 | UP |
| EYA1 | 552.3449079 | -1.155949675 | 0.173543931 | -6.66085 | 2.72E-11 | 1.17E-10 | DOWN |
| E2F2 | 79.00321131 | 1.219781145 | 0.18336309 | 6.652272 | 2.89E-11 | 1.24E-10 | UP |
| VGLL1 | 64.81170857 | -1.509151952 | 0.22755547 | -6.63202 | 3.31E-11 | 1.41E-10 | DOWN |
| TMEM63C | 99.30830595 | 1.517979461 | 0.22915146 | 6.62435 | 3.49E-11 | 1.48E-10 | UP |
| ZFP92 | 65.34614901 | -1.045047052 | 0.157776262 | -6.6236 | 3.51E-11 | 1.49E-10 | DOWN |
| L1CAM | 216.9719939 | -1.307608782 | 0.197638737 | -6.61616 | 3.69E-11 | 1.56E-10 | DOWN |
| LRRC26 | 2936.941102 | 1.183040981 | 0.178844986 | 6.614896 | 3.72E-11 | 1.57E-10 | UP |
| TACR2 | 150.8808835 | -1.066222847 | 0.161235895 | -6.61281 | 3.77E-11 | 1.59E-10 | DOWN |
| PRSS12 | 159.9205472 | -1.043632511 | 0.15800821 | -6.60493 | 3.98E-11 | 1.68E-10 | DOWN |
| HBA2 | 169.6209946 | -1.408016478 | 0.213220076 | -6.60358 | 4.01E-11 | 1.69E-10 | DOWN |
| DDC | 277.4975805 | 2.005084172 | 0.304185947 | 6.59164 | 4.35E-11 | 1.83E-10 | UP |
| NCALD | 2230.025249 | 1.032967508 | 0.157135817 | 6.573724 | 4.91E-11 | 2.05E-10 | UP |
| ANXA8 | 95.01208103 | -1.589620209 | 0.24184748 | -6.57282 | 4.94E-11 | 2.06E-10 | DOWN |
| PCDHB2 | 559.9097137 | 1.35094267 | 0.205869264 | 6.562139 | 5.30E-11 | 2.21E-10 | UP |
| S100B | 135.6510534 | -1.291033566 | 0.196812495 | -6.55971 | 5.39E-11 | 2.24E-10 | DOWN |
| BDKRB2 | 272.5879906 | -1.019359051 | 0.155521365 | -6.55446 | 5.58E-11 | 2.32E-10 | DOWN |
| KRT14 | 2022.963199 | -1.591186594 | 0.242891341 | -6.55102 | 5.71E-11 | 2.37E-10 | DOWN |
| AREG | 187.8128159 | -1.633692767 | 0.249559305 | -6.54631 | 5.90E-11 | 2.44E-10 | DOWN |
| C7 | 5036.903738 | -1.271636199 | 0.194267849 | -6.54579 | 5.92E-11 | 2.45E-10 | DOWN |
| CLCA4 | 182.8475036 | -2.455792192 | 0.375459932 | -6.54076 | 6.12E-11 | 2.53E-10 | DOWN |
| PAX5 | 105.7669182 | 1.63137373 | 0.249434418 | 6.540291 | 6.14E-11 | 2.54E-10 | UP |
| LRRN1 | 2404.152545 | 1.444197697 | 0.220947451 | 6.536385 | 6.30E-11 | 2.60E-10 | UP |
| RPLP0P2 | 449.3806812 | 1.187254589 | 0.181926444 | 6.526014 | 6.75E-11 | 2.78E-10 | UP |
| FBLN1 | 15159.34094 | -1.04235762 | 0.159727503 | -6.52585 | 6.76E-11 | 2.78E-10 | DOWN |
| FOXF2 | 330.9353097 | -1.308705594 | 0.200561345 | -6.52521 | 6.79E-11 | 2.79E-10 | DOWN |
| NTRK3 | 256.9458727 | -1.080410498 | 0.165735452 | -6.51889 | 7.08E-11 | 2.91E-10 | DOWN |

| | | | | | | | |
|---|---|---|---|---|---|---|---|
| IL5RA | 465.2564729 | 1.988211575 | 0.3052503 | 6.513381 | 7.35E-11 | 3.02E-10 | UP |
| ST18 | 85.92191336 | 1.650324836 | 0.253535028 | 6.509258 | 7.55E-11 | 3.10E-10 | UP |
| CLDN8 | 3474.357319 | 1.030366831 | 0.158364556 | 6.506297 | 7.70E-11 | 3.16E-10 | UP |
| FOSL1 | 256.7681631 | -1.768129898 | 0.271794498 | -6.50539 | 7.75E-11 | 3.17E-10 | DOWN |
| LRRC7 | 246.5541013 | 1.413827408 | 0.217366289 | 6.504355 | 7.80E-11 | 3.19E-10 | UP |
| SCN7A | 742.5963247 | -1.075402451 | 0.165494257 | -6.49813 | 8.13E-11 | 3.32E-10 | DOWN |
| KIF15 | 144.9310991 | 1.03229313 | 0.158966133 | 6.493793 | 8.37E-11 | 3.41E-10 | UP |
| STXBP5L | 267.8629916 | -1.201844125 | 0.185741069 | -6.47054 | 9.77E-11 | 3.96E-10 | DOWN |
| C16orf89 | 171.6638016 | -1.163515672 | 0.179820379 | -6.47043 | 9.77E-11 | 3.96E-10 | DOWN |
| WFIKKN1 | 123.9821588 | 1.076076353 | 0.166460287 | 6.464463 | 1.02E-10 | 4.11E-10 | UP |
| SLC9A3 | 150.6676646 | 1.590894935 | 0.246517539 | 6.453476 | 1.09E-10 | 4.41E-10 | UP |
| TRPM8 | 31107.44376 | 1.193214479 | 0.185002919 | 6.449706 | 1.12E-10 | 4.52E-10 | UP |
| CDK1 | 497.4157217 | 1.10580838 | 0.171530829 | 6.446703 | 1.14E-10 | 4.61E-10 | UP |
| SCGB2A1 | 130.9165912 | -1.314817496 | 0.20426716 | -6.43675 | 1.22E-10 | 4.91E-10 | DOWN |
| LHX4 | 56.30192227 | 1.280973197 | 0.199019524 | 6.43642 | 1.22E-10 | 4.92E-10 | UP |
| HSPB2 | 54.65856983 | -1.063297033 | 0.165313084 | -6.43202 | 1.26E-10 | 5.05E-10 | DOWN |
| NTF4 | 142.8209598 | -1.327117555 | 0.206453243 | -6.42817 | 1.29E-10 | 5.17E-10 | DOWN |
| RASGRF1 | 53.17983066 | -1.346282059 | 0.209532397 | -6.42517 | 1.32E-10 | 5.27E-10 | DOWN |
| VTCN1 | 250.9391544 | -1.580987057 | 0.246247152 | -6.42033 | 1.36E-10 | 5.43E-10 | DOWN |
| WDR62 | 153.3821406 | 1.030134545 | 0.160632199 | 6.413002 | 1.43E-10 | 5.69E-10 | UP |
| AGR3 | 529.664595 | 1.84003436 | 0.287159593 | 6.407706 | 1.48E-10 | 5.88E-10 | UP |
| IL1RAPL1 | 76.16514001 | 1.785622381 | 0.278948359 | 6.401265 | 1.54E-10 | 6.11E-10 | UP |
| WNT3A | 100.7818676 | -1.318493707 | 0.206009915 | -6.40015 | 1.55E-10 | 6.15E-10 | DOWN |
| SERHL | 276.1711123 | 1.147913502 | 0.179415074 | 6.398088 | 1.57E-10 | 6.23E-10 | UP |
| SCRG1 | 243.9468423 | -1.035012442 | 0.161795835 | -6.39703 | 1.58E-10 | 6.27E-10 | DOWN |
| COX7A1 | 672.1384314 | -1.061647663 | 0.166059717 | -6.39317 | 1.62E-10 | 6.42E-10 | DOWN |
| CDCA2 | 63.20282557 | 1.161133666 | 0.181701015 | 6.390353 | 1.66E-10 | 6.53E-10 | UP |
| CYP27A1 | 2966.03852 | -1.204693749 | 0.188739131 | -6.38285 | 1.74E-10 | 6.85E-10 | DOWN |
| NHSL2 | 89.62394396 | -1.169185324 | 0.183267673 | -6.37966 | 1.77E-10 | 6.99E-10 | DOWN |
| HS6ST3 | 280.8205696 | 1.479275898 | 0.231887108 | 6.379293 | 1.78E-10 | 7.00E-10 | UP |
| SUCNR1 | 50.45619145 | 1.795640725 | 0.282102647 | 6.365203 | 1.95E-10 | 7.64E-10 | UP |
| TNFRSF18 | 137.0920134 | 1.012042305 | 0.159066348 | 6.362391 | 1.99E-10 | 7.78E-10 | UP |
| TRPM2 | 760.603301 | 1.20262729 | 0.189446246 | 6.348119 | 2.18E-10 | 8.50E-10 | UP |
| RPE65 | 53.70192395 | -2.014903986 | 0.318093735 | -6.33431 | 2.38E-10 | 9.25E-10 | DOWN |
| PCBP3 | 129.0714662 | 1.190897008 | 0.188107431 | 6.330941 | 2.44E-10 | 9.44E-10 | UP |
| YBX2 | 61.77542458 | -1.09190326 | 0.172592883 | -6.32647 | 2.51E-10 | 9.70E-10 | DOWN |
| DES | 97888.07236 | -1.442263471 | 0.227979507 | -6.32629 | 2.51E-10 | 9.71E-10 | DOWN |
| NUSAP1 | 435.9300031 | 1.027308182 | 0.163002376 | 6.302412 | 2.93E-10 | 1.13E-09 | UP |
| ASB9 | 138.4499698 | 1.389895752 | 0.220574544 | 6.301252 | 2.95E-10 | 1.13E-09 | UP |
| ANLN | 264.6890446 | 1.066584297 | 0.169515774 | 6.291947 | 3.14E-10 | 1.20E-09 | UP |
| CBS | 194.00981 | 1.375497498 | 0.21871186 | 6.289085 | 3.19E-10 | 1.22E-09 | UP |
| FLRT3 | 1227.99589 | -1.204565977 | 0.192048912 | -6.27218 | 3.56E-10 | 1.36E-09 | DOWN |
| HS3ST4 | 297.8826179 | 1.301978641 | 0.207835023 | 6.264481 | 3.74E-10 | 1.42E-09 | UP |
| KCNK3 | 1719.116426 | -1.277769569 | 0.204061581 | -6.26169 | 3.81E-10 | 1.45E-09 | DOWN |
| HSPA1A | 9090.337576 | -1.047237711 | 0.167324898 | -6.25871 | 3.88E-10 | 1.47E-09 | DOWN |
| DCN | 12612.78993 | -1.049327994 | 0.168028246 | -6.24495 | 4.24E-10 | 1.60E-09 | DOWN |
| RET | 1329.654373 | 1.463208077 | 0.23455009 | 6.238361 | 4.42E-10 | 1.67E-09 | UP |
| PLA2G2A | 80264.66592 | 1.881836278 | 0.302038401 | 6.230454 | 4.65E-10 | 1.75E-09 | UP |
| FN1 | 28108.8038 | -1.187936855 | 0.190752865 | -6.22762 | 4.74E-10 | 1.78E-09 | DOWN |
| CST4 | 42.82376898 | -2.466971766 | 0.396322136 | -6.22466 | 4.83E-10 | 1.82E-09 | DOWN |
| COL14A1 | 3525.553765 | -1.029976381 | 0.165826885 | -6.21115 | 5.26E-10 | 1.97E-09 | DOWN |
| COL28A1 | 558.9560221 | 1.17060269 | 0.189079018 | 6.191077 | 5.98E-10 | 2.23E-09 | UP |
| MUC4 | 875.3726257 | -1.756436198 | 0.283981367 | -6.18504 | 6.21E-10 | 2.31E-09 | DOWN |
| ESPNL | 51.7403453 | -1.282709442 | 0.207487709 | -6.1821 | 6.33E-10 | 2.35E-09 | DOWN |
| FAP | 264.9516347 | 1.034049292 | 0.167616483 | 6.169138 | 6.87E-10 | 2.54E-09 | UP |
| PEX10 | 4841.6149 | 1.118753292 | 0.181389939 | 6.16767 | 6.93E-10 | 2.57E-09 | UP |
| CXCL10 | 729.206161 | 1.22299634 | 0.198493939 | 6.161379 | 7.21E-10 | 2.66E-09 | UP |
| GCNT3 | 67.32855883 | -1.339449222 | 0.217414723 | -6.1608 | 7.24E-10 | 2.67E-09 | DOWN |
| SAMD13 | 415.8870043 | 1.03004556 | 0.167444388 | 6.151568 | 7.67E-10 | 2.83E-09 | UP |
| NPY | 53044.51584 | 2.072016051 | 0.336956018 | 6.149218 | 7.79E-10 | 2.87E-09 | UP |
| CST1 | 721.9548094 | 2.319583291 | 0.377462634 | 6.1452 | 7.99E-10 | 2.94E-09 | UP |
| LRRC2 | 96.90555504 | -1.408215038 | 0.229468923 | -6.13684 | 8.42E-10 | 3.09E-09 | DOWN |
| EDN3 | 134.6996843 | -1.622297766 | 0.266069451 | -6.09727 | 1.08E-09 | 3.91E-09 | DOWN |

| | | | | | | | |
|---|---|---|---|---|---|---|---|
| KLHL30 | 53.90359585 | -1.202556991 | 0.197283062 | -6.09559 | 1.09E-09 | 3.95E-09 | DOWN |
| F5 | 3441.102193 | 1.506467523 | 0.247665153 | 6.082679 | 1.18E-09 | 4.26E-09 | UP |
| PLP1 | 380.740396 | -1.461946092 | 0.240466969 | -6.07961 | 1.20E-09 | 4.34E-09 | DOWN |
| FOLH1B | 516.0514109 | 1.988570973 | 0.327739191 | 6.067541 | 1.30E-09 | 4.66E-09 | UP |
| HAS2 | 92.56459429 | -1.122239595 | 0.185163368 | -6.06081 | 1.35E-09 | 4.85E-09 | DOWN |
| PROC | 64.14575795 | 1.613672375 | 0.266850977 | 6.047092 | 1.47E-09 | 5.27E-09 | UP |
| TYRP1 | 55.52935674 | 1.815329154 | 0.300225994 | 6.046542 | 1.48E-09 | 5.29E-09 | UP |
| CD38 | 3682.157107 | -1.562066896 | 0.258362967 | -6.04602 | 1.48E-09 | 5.31E-09 | DOWN |
| ENPP6 | 164.020308 | -1.134371789 | 0.187726407 | -6.04269 | 1.52E-09 | 5.41E-09 | DOWN |
| PRR4 | 247.6320115 | 1.421462734 | 0.235250881 | 6.042327 | 1.52E-09 | 5.42E-09 | UP |
| ART4 | 133.3395519 | 1.640527854 | 0.272034054 | 6.030597 | 1.63E-09 | 5.81E-09 | UP |
| KRT15 | 7589.870839 | -1.612530186 | 0.267657759 | -6.0246 | 1.70E-09 | 6.02E-09 | DOWN |
| SRL | 205.4476873 | -1.345309659 | 0.223369727 | -6.02279 | 1.71E-09 | 6.08E-09 | DOWN |
| UHRF1 | 158.80987 | 1.072931474 | 0.17826727 | 6.018668 | 1.76E-09 | 6.23E-09 | UP |
| PABPC1L2B | 285.2223057 | 1.336295409 | 0.222207159 | 6.013737 | 1.81E-09 | 6.42E-09 | UP |
| IFI6 | 8122.834044 | 1.392276028 | 0.231591577 | 6.011773 | 1.84E-09 | 6.49E-09 | UP |
| DSG3 | 68.22938263 | -1.787776099 | 0.29773464 | -6.0046 | 1.92E-09 | 6.77E-09 | DOWN |
| DPY19L2 | 709.8720296 | 1.102730699 | 0.183739407 | 6.001601 | 1.95E-09 | 6.88E-09 | UP |
| CEACAM6 | 285.6926432 | -1.459850428 | 0.243423738 | -5.99716 | 2.01E-09 | 7.07E-09 | DOWN |
| ACAN | 107.4184935 | 1.372375042 | 0.228938579 | 5.994512 | 2.04E-09 | 7.18E-09 | UP |
| EGF | 597.8794792 | 1.13371256 | 0.18993342 | 5.969 | 2.39E-09 | 8.33E-09 | UP |
| BCL11A | 363.8893821 | -1.030499275 | 0.172643583 | -5.96894 | 2.39E-09 | 8.33E-09 | DOWN |
| SLC44A5 | 497.1513265 | 1.827154902 | 0.30649086 | 5.961531 | 2.50E-09 | 8.70E-09 | UP |
| PCDHGB2 | 282.1566158 | 1.259840549 | 0.211391446 | 5.959752 | 2.53E-09 | 8.79E-09 | UP |
| HS3ST2 | 155.7490609 | 1.056383928 | 0.177345847 | 5.956632 | 2.57E-09 | 8.95E-09 | UP |
| RDH12 | 155.575606 | 1.158996364 | 0.195407961 | 5.931162 | 3.01E-09 | 1.04E-08 | UP |
| MMP9 | 758.9540934 | 1.3278553 | 0.225468327 | 5.889321 | 3.88E-09 | 1.33E-08 | UP |
| CRIP3 | 71.91171659 | -1.101549194 | 0.187456157 | -5.8763 | 4.20E-09 | 1.43E-08 | DOWN |
| EDAR | 66.18997228 | -1.438103632 | 0.245702023 | -5.85304 | 4.83E-09 | 1.63E-08 | DOWN |
| MYH6 | 82.89951309 | -2.760510118 | 0.472015776 | -5.84834 | 4.96E-09 | 1.68E-08 | DOWN |
| CCK | 479.5927967 | -1.907819211 | 0.326652549 | -5.84052 | 5.20E-09 | 1.75E-08 | DOWN |
| GPRC5D | 67.40422704 | 1.213148837 | 0.207767761 | 5.838966 | 5.25E-09 | 1.77E-08 | UP |
| KCNC2 | 1460.373288 | 1.940036661 | 0.332515497 | 5.834425 | 5.40E-09 | 1.81E-08 | UP |
| CD163L1 | 176.2764941 | 1.217068549 | 0.208805111 | 5.82873 | 5.59E-09 | 1.87E-08 | UP |
| SLC4A4 | 20544.44981 | 1.068450028 | 0.183376044 | 5.826552 | 5.66E-09 | 1.89E-08 | UP |
| AGTR1 | 1148.956151 | 1.486777255 | 0.255488934 | 5.819341 | 5.91E-09 | 1.97E-08 | UP |
| INSM1 | 343.0855509 | 1.537564542 | 0.264304737 | 5.817393 | 5.98E-09 | 1.99E-08 | UP |
| PCDHB10 | 288.5363753 | 1.043790786 | 0.179656517 | 5.809924 | 6.25E-09 | 2.08E-08 | UP |
| PCOTH | 451.9970492 | 1.630365449 | 0.280648991 | 5.809269 | 6.27E-09 | 2.08E-08 | UP |
| GPRC5A | 2634.915944 | -1.178913849 | 0.202984686 | -5.8079 | 6.33E-09 | 2.10E-08 | DOWN |
| KCNN2 | 2449.600097 | 1.058659629 | 0.182527459 | 5.800002 | 6.63E-09 | 2.20E-08 | UP |
| MMP10 | 390.8104212 | 1.360812108 | 0.234937097 | 5.79224 | 6.95E-09 | 2.30E-08 | UP |
| LRRN3 | 120.5238935 | -1.321796767 | 0.228324227 | -5.78912 | 7.08E-09 | 2.34E-08 | DOWN |
| MIR17HG | 52.92828816 | 1.195926808 | 0.206710831 | 5.785506 | 7.23E-09 | 2.38E-08 | UP |
| IL1RL2 | 87.2812232 | -1.173324446 | 0.20301728 | -5.77943 | 7.50E-09 | 2.47E-08 | DOWN |
| RHCG | 67.04929309 | -2.099665265 | 0.363529246 | -5.77578 | 7.66E-09 | 2.52E-08 | DOWN |
| PRSS16 | 432.1239835 | -1.051358551 | 0.182073072 | -5.77438 | 7.72E-09 | 2.54E-08 | DOWN |
| DKK1 | 349.7186868 | -1.447621915 | 0.250751554 | -5.77313 | 7.78E-09 | 2.56E-08 | DOWN |
| OLFM4 | 9574.442349 | -2.026610051 | 0.352853646 | -5.74349 | 9.27E-09 | 3.02E-08 | DOWN |
| SOX14 | 254.8051614 | 1.936373614 | 0.337417629 | 5.738804 | 9.53E-09 | 3.10E-08 | UP |
| GOLGA8B | 1033.342117 | 1.03878453 | 0.181204279 | 5.732671 | 9.89E-09 | 3.20E-08 | UP |
| HIST1H2AD | 95.43616295 | 1.024826458 | 0.179101493 | 5.722043 | 1.05E-08 | 3.40E-08 | UP |
| C4orf19 | 136.3630763 | -1.241912338 | 0.217228966 | -5.71707 | 1.08E-08 | 3.50E-08 | DOWN |
| ERN2 | 137.0822297 | -1.765402047 | 0.308936792 | -5.71444 | 1.10E-08 | 3.55E-08 | DOWN |
| IL31RA | 83.26149979 | 1.406798551 | 0.24661797 | 5.704364 | 1.17E-08 | 3.75E-08 | UP |
| RSPO2 | 256.3820767 | -1.213241401 | 0.213227769 | -5.68988 | 1.27E-08 | 4.07E-08 | DOWN |
| IGFN1 | 85.91762824 | 1.478989927 | 0.260670375 | 5.673794 | 1.40E-08 | 4.45E-08 | UP |
| KCNK15 | 96.09787548 | -1.117076992 | 0.197101195 | -5.66753 | 1.45E-08 | 4.61E-08 | DOWN |
| ZFPM2 | 65.99076381 | -1.029442379 | 0.181749979 | -5.66406 | 1.48E-08 | 4.69E-08 | DOWN |
| CEL | 105.8771841 | -1.325896692 | 0.234557833 | -5.65275 | 1.58E-08 | 4.99E-08 | DOWN |
| ANO4 | 361.6601907 | -1.005496866 | 0.178126474 | -5.64485 | 1.65E-08 | 5.22E-08 | DOWN |
| SGK1 | 6427.987098 | -1.206389451 | 0.213787289 | -5.64294 | 1.67E-08 | 5.27E-08 | DOWN |
| GDF7 | 692.2944994 | -1.054481977 | 0.186874022 | -5.64274 | 1.67E-08 | 5.28E-08 | DOWN |

| | | | | | | | |
|---|---|---|---|---|---|---|---|
| DUSP5 | 1545.976026 | -1.099433676 | 0.195044409 | -5.63684 | 1.73E-08 | 5.45E-08 | DOWN |
| CNKSR2 | 329.8576339 | 1.021235801 | 0.181198812 | 5.635996 | 1.74E-08 | 5.47E-08 | UP |
| CLSPN | 51.33948273 | 1.0653735 | 0.189076033 | 5.63463 | 1.75E-08 | 5.51E-08 | UP |
| SYP | 354.0550708 | 1.002722795 | 0.178495704 | 5.61763 | 1.94E-08 | 6.05E-08 | UP |
| CXCL17 | 401.8812577 | -1.609404031 | 0.287321945 | -5.6014 | 2.13E-08 | 6.63E-08 | DOWN |
| PROM1 | 418.3589509 | -1.382860698 | 0.246930841 | -5.60019 | 2.14E-08 | 6.67E-08 | DOWN |
| ALOX15 | 336.3273956 | 1.789520245 | 0.319618524 | 5.598925 | 2.16E-08 | 6.72E-08 | UP |
| PMP2 | 57.63899893 | -1.365825273 | 0.24423025 | -5.59237 | 2.24E-08 | 6.96E-08 | DOWN |
| TDO2 | 239.3354078 | 2.258764478 | 0.405082048 | 5.576067 | 2.46E-08 | 7.61E-08 | UP |
| GJB2 | 379.9632476 | 1.114301789 | 0.20004647 | 5.570215 | 2.54E-08 | 7.86E-08 | UP |
| EPHA6 | 229.6876723 | 1.217501296 | 0.218841009 | 5.563406 | 2.65E-08 | 8.15E-08 | UP |
| GOLGA8A | 1020.441455 | 1.07934977 | 0.194148035 | 5.559416 | 2.71E-08 | 8.33E-08 | UP |
| ABO | 431.7510314 | -1.295322634 | 0.233514448 | -5.54708 | 2.90E-08 | 8.91E-08 | DOWN |
| MME | 10503.76033 | -1.191684194 | 0.215314587 | -5.53462 | 3.12E-08 | 9.54E-08 | DOWN |
| TXLNB | 172.6450193 | 1.088181748 | 0.196709389 | 5.531926 | 3.17E-08 | 9.68E-08 | UP |
| SCIN | 1298.473739 | 1.133889726 | 0.205484838 | 5.518119 | 3.43E-08 | 1.04E-07 | UP |
| B3GNT3 | 204.8725877 | -1.376633881 | 0.249739877 | -5.51227 | 3.54E-08 | 1.08E-07 | DOWN |
| CGA | 79.60211211 | 2.193498555 | 0.398356551 | 5.50637 | 3.66E-08 | 1.11E-07 | UP |
| CDH22 | 137.146562 | -1.179087126 | 0.214558455 | -5.49541 | 3.90E-08 | 1.18E-07 | DOWN |
| POPDC3 | 118.2290582 | 1.351493307 | 0.246000324 | 5.493868 | 3.93E-08 | 1.19E-07 | UP |
| SULT1C2 | 60.38443134 | 1.253185585 | 0.228312288 | 5.48891 | 4.04E-08 | 1.22E-07 | UP |
| ITGA8 | 2992.926024 | -1.088685174 | 0.198622647 | -5.48117 | 4.23E-08 | 1.27E-07 | DOWN |
| PPP1R14C | 59.88572643 | -1.071726614 | 0.195933252 | -5.46986 | 4.50E-08 | 1.35E-07 | DOWN |
| BMPER | 247.1560129 | -1.256459416 | 0.229711152 | -5.46974 | 4.51E-08 | 1.35E-07 | DOWN |
| KCNMB2 | 120.2852089 | 1.697125842 | 0.310793428 | 5.460623 | 4.74E-08 | 1.42E-07 | UP |
| TMEM217 | 83.72381297 | 1.095622904 | 0.201129575 | 5.447349 | 5.11E-08 | 1.53E-07 | UP |
| VTN | 85.61853217 | -1.526769982 | 0.281308101 | -5.42739 | 5.72E-08 | 1.70E-07 | DOWN |
| CD177 | 4053.279616 | -1.748010895 | 0.322306303 | -5.42345 | 5.85E-08 | 1.74E-07 | DOWN |
| TMEM26 | 626.3202853 | 1.148878427 | 0.212022536 | 5.418662 | 6.00E-08 | 1.78E-07 | UP |
| SLC38A11 | 2116.072943 | 1.206079928 | 0.2226157 | 5.417767 | 6.03E-08 | 1.79E-07 | UP |
| SNORA74B | 3.869780108 | 2.966621273 | 0.548216449 | 5.411405 | 6.25E-08 | 1.85E-07 | UP |
| ANGPTL4 | 963.7248806 | -1.044533018 | 0.193759336 | -5.39088 | 7.01E-08 | 2.06E-07 | DOWN |
| CHRDL2 | 417.7161533 | -1.107713286 | 0.205582515 | -5.38817 | 7.12E-08 | 2.09E-07 | DOWN |
| CPB1 | 439.8427688 | 1.593666414 | 0.296163551 | 5.381035 | 7.41E-08 | 2.17E-07 | UP |
| SALL1 | 54.28769831 | -1.232881427 | 0.229267928 | -5.37747 | 7.55E-08 | 2.21E-07 | DOWN |
| CDH7 | 392.8035027 | 1.255047783 | 0.233669745 | 5.371032 | 7.83E-08 | 2.28E-07 | UP |
| NRCAM | 750.1348896 | -1.231363067 | 0.229616704 | -5.36269 | 8.20E-08 | 2.38E-07 | DOWN |
| ELFN2 | 1322.377052 | 1.083369627 | 0.202374218 | 5.353299 | 8.64E-08 | 2.51E-07 | UP |
| CAPNS2 | 59.78042483 | -1.807726066 | 0.338262131 | -5.34416 | 9.08E-08 | 2.63E-07 | DOWN |
| MFAP5 | 202.7443928 | -1.304010157 | 0.244531816 | -5.33268 | 9.68E-08 | 2.79E-07 | DOWN |
| CXCL9 | 957.9791978 | 1.167121507 | 0.218952889 | 5.330469 | 9.80E-08 | 2.82E-07 | UP |
| CXCL14 | 2365.886405 | 1.244660609 | 0.233965809 | 5.31984 | 1.04E-07 | 2.99E-07 | UP |
| CASQ2 | 345.755373 | -1.245139568 | 0.234436946 | -5.31119 | 1.09E-07 | 3.13E-07 | DOWN |
| CARTPT | 153.8039497 | -1.841238553 | 0.346892898 | -5.3078 | 1.11E-07 | 3.18E-07 | DOWN |
| BMP7 | 726.477038 | -1.015912981 | 0.192344806 | -5.28173 | 1.28E-07 | 3.64E-07 | DOWN |
| KRT6A | 144.7006478 | -1.623934799 | 0.307611757 | -5.27917 | 1.30E-07 | 3.69E-07 | DOWN |
| C1QTNF3 | 633.1918641 | 1.30813577 | 0.247933038 | 5.276166 | 1.32E-07 | 3.74E-07 | UP |
| C2CD4A | 119.8004785 | 1.420008751 | 0.269255271 | 5.273838 | 1.34E-07 | 3.78E-07 | UP |
| PABPC1L2A | 205.9486545 | 1.186976933 | 0.225343224 | 5.267418 | 1.38E-07 | 3.91E-07 | UP |
| PCDHA10 | 202.3453654 | 1.483108767 | 0.281990156 | 5.259435 | 1.44E-07 | 4.08E-07 | UP |
| ELOVL2 | 1959.9942 | 1.025972505 | 0.195928227 | 5.236471 | 1.64E-07 | 4.59E-07 | UP |
| ACER1 | 49.30632649 | -1.178349271 | 0.225107254 | -5.23461 | 1.65E-07 | 4.64E-07 | DOWN |
| HP | 39.34451739 | 1.717382758 | 0.328444142 | 5.228843 | 1.71E-07 | 4.77E-07 | UP |
| EDDM3A | 100.7689594 | -10.52593986 | 2.01696448 | -5.2187 | 1.80E-07 | 5.03E-07 | DOWN |
| SCARNA7 | 8.19571834 | 1.611469494 | 0.308807493 | 5.218363 | 1.81E-07 | 5.04E-07 | UP |
| SLC5A8 | 112.1699656 | -1.605094987 | 0.307669436 | -5.21695 | 1.82E-07 | 5.07E-07 | DOWN |
| SH2D1A | 122.0872618 | 1.17399878 | 0.22615093 | 5.191218 | 2.09E-07 | 5.78E-07 | UP |
| FOXI1 | 101.5588918 | -2.165438811 | 0.419036338 | -5.16766 | 2.37E-07 | 6.53E-07 | DOWN |
| SCARNA21 | 15.2693669 | 1.325552437 | 0.257430081 | 5.149175 | 2.62E-07 | 7.17E-07 | UP |
| UNC80 | 750.480613 | 1.001201502 | 0.194534835 | 5.146644 | 2.65E-07 | 7.26E-07 | UP |
| NR4A3 | 1319.839912 | -1.310007831 | 0.255086475 | -5.13554 | 2.81E-07 | 7.68E-07 | DOWN |
| LIX1 | 104.733523 | -1.483361081 | 0.288925729 | -5.13406 | 2.84E-07 | 7.74E-07 | DOWN |
| RIMS4 | 124.697968 | -1.104191841 | 0.215717645 | -5.11869 | 3.08E-07 | 8.36E-07 | DOWN |

| | | | | | | | |
|---|---|---|---|---|---|---|---|
| MYH15 | 129.4557662 | 1.0088161 | 0.197417401 | 5.110067 | 3.22E-07 | 8.74E-07 | UP |
| EEF1A2 | 5674.366036 | 1.267049447 | 0.248137163 | 5.106246 | 3.29E-07 | 8.90E-07 | UP |
| SERPINA3 | 246.9074707 | 1.440403585 | 0.282399967 | 5.10058 | 3.39E-07 | 9.16E-07 | UP |
| MUC13 | 542.0687494 | 1.549878 | 0.304219537 | 5.094604 | 3.49E-07 | 9.42E-07 | UP |
| GC | 31.63263894 | 4.250857697 | 0.835495443 | 5.087829 | 3.62E-07 | 9.75E-07 | UP |
| RBP4 | 89.53816391 | -1.132476351 | 0.224015468 | -5.05535 | 4.30E-07 | 1.15E-06 | DOWN |
| FAM3D | 1806.622948 | -1.163089291 | 0.23036558 | -5.04888 | 4.44E-07 | 1.18E-06 | DOWN |
| SGCG | 65.58926317 | -1.273591704 | 0.253038175 | -5.0332 | 4.82E-07 | 1.28E-06 | DOWN |
| CHST9 | 419.3100774 | -1.161137914 | 0.230719584 | -5.03268 | 4.84E-07 | 1.28E-06 | DOWN |
| S100A9 | 399.6886036 | -1.022181219 | 0.204306483 | -5.00318 | 5.64E-07 | 1.48E-06 | DOWN |
| PGC | 6182.654338 | -1.643726026 | 0.329256314 | -4.99224 | 5.97E-07 | 1.57E-06 | DOWN |
| PCDHB3 | 286.6141644 | 1.198357208 | 0.24068438 | 4.978957 | 6.39E-07 | 1.67E-06 | UP |
| LIPF | 19.84064118 | 6.105736633 | 1.230376057 | 4.962496 | 6.96E-07 | 1.81E-06 | UP |
| S100A2 | 470.8814692 | -1.178034187 | 0.237869093 | -4.95245 | 7.33E-07 | 1.91E-06 | DOWN |
| TMED6 | 144.2745274 | 1.436148884 | 0.290225632 | 4.948387 | 7.48E-07 | 1.94E-06 | UP |
| TGM4 | 31817.38622 | -2.317997431 | 0.473168994 | -4.89888 | 9.64E-07 | 2.47E-06 | DOWN |
| VEPH1 | 81.72723261 | -1.060117623 | 0.217344436 | -4.87759 | 1.07E-06 | 2.74E-06 | DOWN |
| PLA2G2D | 80.59846218 | 1.298196142 | 0.2666772 | 4.868043 | 1.13E-06 | 2.87E-06 | UP |
| SOCS3 | 5238.92386 | -1.088656635 | 0.223635064 | -4.86801 | 1.13E-06 | 2.87E-06 | DOWN |
| DLX6 | 47.71092786 | 1.448771488 | 0.297812345 | 4.864713 | 1.15E-06 | 2.91E-06 | UP |
| CEACAM22P | 112.5864394 | 1.835760802 | 0.378891086 | 4.845088 | 1.27E-06 | 3.20E-06 | UP |
| UNC13A | 35.98700735 | 1.030633757 | 0.213130594 | 4.835691 | 1.33E-06 | 3.35E-06 | UP |
| KRTAP13-2 | 47.71469168 | 2.340041874 | 0.486100186 | 4.813909 | 1.48E-06 | 3.71E-06 | UP |
| GLP1R | 24.70717524 | 1.863402338 | 0.388530556 | 4.796025 | 1.62E-06 | 4.04E-06 | UP |
| REG4 | 440.9631386 | 1.296342399 | 0.271310397 | 4.778079 | 1.77E-06 | 4.40E-06 | UP |
| FOXD3 | 91.62567399 | 1.225575883 | 0.257065964 | 4.767554 | 1.86E-06 | 4.62E-06 | UP |
| ETV1 | 1710.567029 | 1.350062204 | 0.284005145 | 4.753654 | 2.00E-06 | 4.93E-06 | UP |
| HPGD | 3716.218446 | 1.345785721 | 0.283344141 | 4.749651 | 2.04E-06 | 5.03E-06 | UP |
| ABCC11 | 617.1314046 | 1.492277219 | 0.314959868 | 4.737992 | 2.16E-06 | 5.31E-06 | UP |
| OR51E1 | 6227.442691 | 1.298357879 | 0.276746228 | 4.691511 | 2.71E-06 | 6.59E-06 | UP |
| FCRL3 | 47.60629094 | 1.27493454 | 0.27183193 | 4.690157 | 2.73E-06 | 6.64E-06 | UP |
| FGFBP1 | 79.07726842 | -1.645282956 | 0.351278157 | -4.6837 | 2.82E-06 | 6.84E-06 | DOWN |
| EOMES | 61.16156282 | 1.057961552 | 0.226377914 | 4.673431 | 2.96E-06 | 7.17E-06 | UP |
| CCDC141 | 198.849781 | 1.420164315 | 0.304318333 | 4.666706 | 3.06E-06 | 7.39E-06 | UP |
| TIMP4 | 167.0954465 | -1.043313871 | 0.223970951 | -4.65826 | 3.19E-06 | 7.67E-06 | DOWN |
| BMP5 | 354.8782257 | -2.111898647 | 0.454693568 | -4.64466 | 3.41E-06 | 8.19E-06 | DOWN |
| SNX31 | 94.80569257 | -1.105611403 | 0.239861584 | -4.60937 | 4.04E-06 | 9.63E-06 | DOWN |
| ZNF385B | 1678.403877 | 1.021420016 | 0.222435935 | 4.591974 | 4.39E-06 | 1.04E-05 | UP |
| DAPL1 | 110.5472718 | -1.59393047 | 0.348400522 | -4.57499 | 4.76E-06 | 1.13E-05 | DOWN |
| CES3 | 103.1635833 | 1.281014431 | 0.28079029 | 4.562175 | 5.06E-06 | 1.19E-05 | UP |
| PCDHA4 | 123.4611172 | 1.227876999 | 0.270228794 | 4.543842 | 5.52E-06 | 1.30E-05 | UP |
| COL9A1 | 315.7748278 | -1.242234554 | 0.279125093 | -4.45046 | 8.57E-06 | 1.97E-05 | DOWN |
| SELE | 1337.834918 | -1.158190149 | 0.260905375 | -4.43912 | 9.03E-06 | 2.07E-05 | DOWN |
| GABRP | 945.6583231 | -1.093170829 | 0.246439647 | -4.43586 | 9.17E-06 | 2.11E-05 | DOWN |
| DIRAS2 | 106.4186542 | 1.453598907 | 0.328198287 | 4.429026 | 9.47E-06 | 2.17E-05 | UP |
| SEMA3D | 934.9613047 | -1.034954094 | 0.23418632 | -4.41936 | 9.90E-06 | 2.26E-05 | DOWN |
| LRRC31 | 271.7278721 | 1.298939143 | 0.294593733 | 4.409256 | 1.04E-05 | 2.36E-05 | UP |
| FGB | 20.2543314 | 4.349954064 | 0.990128429 | 4.393323 | 1.12E-05 | 2.53E-05 | UP |
| CLSTN2 | 1090.039788 | -1.240757504 | 0.28280396 | -4.38734 | 1.15E-05 | 2.60E-05 | DOWN |
| ECEL1 | 38.79777955 | 1.013325596 | 0.230973657 | 4.387191 | 1.15E-05 | 2.60E-05 | UP |
| NEFL | 279.7575363 | -1.517215797 | 0.351467347 | -4.3168 | 1.58E-05 | 3.54E-05 | DOWN |
| DEFB1 | 223.0961452 | -1.124509041 | 0.261141401 | -4.30613 | 1.66E-05 | 3.71E-05 | DOWN |
| MUCL1 | 49.90951818 | 1.145998051 | 0.266404332 | 4.301725 | 1.69E-05 | 3.77E-05 | UP |
| KRT17 | 8592.107967 | -1.01317228 | 0.23688342 | -4.27709 | 1.89E-05 | 4.19E-05 | DOWN |
| RLN2 | 277.3083321 | 1.240893761 | 0.291598611 | 4.255486 | 2.09E-05 | 4.59E-05 | UP |
| FNDC1 | 90.76595755 | 1.021509933 | 0.240589151 | 4.245869 | 2.18E-05 | 4.78E-05 | UP |
| OXGR1 | 77.13855221 | 1.312219578 | 0.309510153 | 4.239666 | 2.24E-05 | 4.91E-05 | UP |
| CEACAM5 | 148.4781507 | 1.377297564 | 0.327393885 | 4.206852 | 2.59E-05 | 5.63E-05 | UP |
| CXCL13 | 396.0840888 | -1.437502329 | 0.342877088 | -4.19247 | 2.76E-05 | 5.98E-05 | DOWN |
| DPYS | 234.4022163 | -1.344739633 | 0.320935814 | -4.19006 | 2.79E-05 | 6.04E-05 | DOWN |
| PCDHA11 | 98.74814264 | 1.39497549 | 0.334463046 | 4.170791 | 3.04E-05 | 6.55E-05 | UP |
| CTNNA2 | 64.63932345 | -1.278868095 | 0.306938519 | -4.16653 | 3.09E-05 | 6.67E-05 | DOWN |
| EML6 | 309.7504256 | 1.000296217 | 0.240376494 | 4.161373 | 3.16E-05 | 6.81E-05 | UP |

| | | | | | | | |
|---|---|---|---|---|---|---|---|
| NKX3-2 | 40.61079022 | 1.060466926 | 0.255052002 | 4.157846 | 3.21E-05 | 6.90E-05 | UP |
| ARX | 322.2196433 | 1.060472467 | 0.255891773 | 4.144223 | 3.41E-05 | 7.30E-05 | UP |
| PTPRT | 1231.623078 | 1.116069365 | 0.269726543 | 4.137781 | 3.51E-05 | 7.50E-05 | UP |
| DEFA5 | 45.59884016 | 6.776937667 | 1.663354521 | 4.074259 | 4.62E-05 | 9.74E-05 | UP |
| POTEG | 82.36307182 | 1.072705241 | 0.263357656 | 4.073188 | 4.64E-05 | 9.78E-05 | UP |
| MUC2 | 242.0794623 | 1.206078323 | 0.296845114 | 4.062989 | 4.84E-05 | 0.000102 | UP |
| XIRP1 | 71.23113623 | 1.711813646 | 0.421615432 | 4.06013 | 4.90E-05 | 0.0001031 | UP |
| TCAP | 380.4611458 | -1.265014433 | 0.312972411 | -4.04194 | 5.30E-05 | 0.0001111 | DOWN |
| NEFH | 30366.52571 | -1.214775966 | 0.302876845 | -4.01079 | 6.05E-05 | 0.0001258 | DOWN |
| TNNC1 | 288.3494335 | -1.175075336 | 0.29431179 | -3.99262 | 6.53E-05 | 0.0001352 | DOWN |
| COL11A1 | 68.77345053 | 1.220455669 | 0.305791115 | 3.991142 | 6.58E-05 | 0.000136 | UP |
| C12orf56 | 105.6911603 | 1.304930979 | 0.331668847 | 3.934439 | 8.34E-05 | 0.0001702 | UP |
| SLC3A1 | 114.7560511 | 1.085443895 | 0.277743479 | 3.908081 | 9.30E-05 | 0.0001888 | UP |
| MYH3 | 968.1886808 | -1.075391417 | 0.278252519 | -3.8648 | 0.0001112 | 0.0002234 | DOWN |
| PCDHA6 | 59.88490441 | 1.380224338 | 0.361045825 | 3.822851 | 0.0001319 | 0.0002627 | UP |
| SI | 533.8946709 | 1.162867021 | 0.309745052 | 3.754271 | 0.0001738 | 0.0003415 | UP |
| CYP24A1 | 138.8327531 | -1.155583597 | 0.308655753 | -3.74392 | 0.0001812 | 0.0003553 | DOWN |
| H19 | 286.4870813 | -1.202233703 | 0.323226267 | -3.71948 | 0.0001996 | 0.0003897 | DOWN |
| PLA2G4D | 386.757826 | -1.361330221 | 0.370061951 | -3.67865 | 0.0002345 | 0.0004535 | DOWN |
| CPS1 | 257.7221128 | 1.134522909 | 0.310458507 | 3.654346 | 0.0002578 | 0.0004956 | UP |
| HAPLN1 | 50.78231476 | -1.238608209 | 0.343774933 | -3.60296 | 0.0003146 | 0.0005974 | DOWN |
| CKM | 665.5300006 | -1.346823109 | 0.374503826 | -3.59629 | 0.0003228 | 0.0006117 | DOWN |
| UGT2B15 | 66.23790096 | 1.218127534 | 0.339503121 | 3.587972 | 0.0003333 | 0.0006304 | UP |
| GLDC | 288.4526879 | 1.109385698 | 0.310787114 | 3.5696 | 0.0003575 | 0.000674 | UP |
| SIK1 | 186.6071114 | -1.206171998 | 0.340827708 | -3.53895 | 0.0004017 | 0.0007523 | DOWN |
| CASQ1 | 181.8491049 | -1.035686666 | 0.294572159 | -3.5159 | 0.0004383 | 0.0008171 | DOWN |
| APOBEC2 | 21.22765057 | -1.320196956 | 0.376906797 | -3.50271 | 0.0004605 | 0.0008556 | DOWN |
| CLEC3A | 147.4207687 | -1.523671611 | 0.437286706 | -3.48438 | 0.0004933 | 0.0009127 | DOWN |
| SERPINB3 | 74.85897742 | -1.146496864 | 0.332385417 | -3.4493 | 0.000562 | 0.0010323 | DOWN |
| NAP1L6 | 104.672176 | 1.215644011 | 0.353482153 | 3.439053 | 0.0005838 | 0.0010689 | UP |
| GRM7 | 128.7964852 | 1.061091303 | 0.313572365 | 3.38388 | 0.0007147 | 0.0012942 | UP |
| VIP | 22.01897152 | 1.074581647 | 0.319900104 | 3.359116 | 0.0007819 | 0.0014068 | UP |
| SERPINA4 | 93.92001148 | -1.678882025 | 0.506712242 | -3.31328 | 0.0009221 | 0.0016439 | DOWN |
| DEFA6 | 7.338732712 | 5.126386418 | 1.587549132 | 3.22912 | 0.0012417 | 0.0021756 | UP |
| GCG | 55.54659413 | 1.147516787 | 0.366026927 | 3.135061 | 0.0017182 | 0.002954 | UP |
| PNMA5 | 3.705556656 | 1.880218144 | 0.600016557 | 3.13361 | 0.0017267 | 0.0029675 | UP |
| SAA2 | 144.568943 | 1.096534173 | 0.350022553 | 3.132753 | 0.0017318 | 0.0029746 | UP |
| CR2 | 41.52799023 | 1.224595808 | 0.395969407 | 3.092653 | 0.0019838 | 0.0033766 | UP |
| GFAP | 38.876525 | 1.007788674 | 0.331178184 | 3.043041 | 0.002342 | 0.0039404 | UP |
| NPPC | 134.4233536 | -1.087207532 | 0.358708267 | -3.0309 | 0.0024383 | 0.0040934 | DOWN |
| PON1 | 122.3401689 | 1.322514907 | 0.442183174 | 2.990876 | 0.0027818 | 0.0046236 | UP |
| SERPINB11 | 1404.650742 | -1.173393666 | 0.392332557 | -2.99081 | 0.0027823 | 0.0046236 | DOWN |
| HSPB3 | 53.55305567 | -1.40978911 | 0.4726915 | -2.98247 | 0.0028593 | 0.0047438 | DOWN |
| MYL3 | 53.15097833 | -1.041690222 | 0.350433005 | -2.97258 | 0.0029531 | 0.0048895 | DOWN |
| WFDC12 | 76.17553374 | -1.116262194 | 0.378803299 | -2.94681 | 0.0032107 | 0.005292 | DOWN |
| SCARNA10 | 4.519121242 | 1.017862368 | 0.34747271 | 2.92933 | 0.0033969 | 0.0055787 | UP |
| TNNT3 | 94.09949524 | -1.041521669 | 0.375171856 | -2.77612 | 0.0055012 | 0.0087498 | DOWN |
| PAH | 124.537324 | 1.018643681 | 0.375037968 | 2.716108 | 0.0066054 | 0.0103654 | UP |
| CRISP3 | 10011.3825 | 1.073638012 | 0.400472066 | 2.680931 | 0.0073418 | 0.0114239 | UP |
| NKX2-1 | 54.29400371 | 1.318307607 | 0.50676095 | 2.601439 | 0.0092834 | 0.0142125 | UP |
