# Supplementary material for: Integrative Analysis of Bulk RNA-Seq and Single-Cell RNA-Seq Unveils the Characteristics of the Immune Microenvironment and Prognosis Signature in Prostate Cancer
Source: J Oncol. 2022 Jul 19;2022:6768139. doi: 10.1155/2022/6768139 (PMC9325591; doi:10.1155/2022/6768139)
Supplement: Supplementary Materials — Figure S1. Workflow of the analysis. Figure S2. Validation of the risk score model using the GSE54460 dataset. A. Patients with prostate cancer (PRAD) in the GSE54460 cohort are listed in ascending order of risk score. B. Progression-free interval (PFI) distribution versus the risk score of each patient in the GSE54460 cohort. C. Kaplan–Meier (KM) curves of patients with different risk levels in the GSE54460 validation set. D. Receiver Operating Characteristic (ROC) curve analysis for 1-, 3- and 5-year PFI using the clinical information of patients of the GSE54460 validation dataset. Figure S3. Validation of the risk score model using the GSE46602 dataset. A. Patients with prostate cancer (PRAD) in the GSE46602 cohort are listed in ascending order of risk score. B. Progression-free interval (PFI) distribution versus the risk score of each patient in the GSE46602 cohort. C. Kaplan–Meier (KM) curves of patients with different risk levels in the GSE46602 validation dataset. D. Receiver Operating Characteristic (ROC) curve analysis for 1-, 3- and 5-year PFI using the clinical information of patients of the GSE46602 validation dataset. Figure S4. Validation of the risk score model using the GSE70768 dataset. A. Patients with prostate cancer (PRAD) in the GSE70768 cohort are listed in ascending order of risk score. B. Progression-free interval (PFI) distribution versus the risk score of each patient in the GSE70768 cohort. C. Kaplan–Meier (KM) curves of patients with different risk levels in the GSE70768 validation dataset. D. Receiver Operating Characteristic (ROC) curve analysis for 1-, 3- and 5-year PFI using the clinical information of patients of the GSE70768 validation dataset. Figure S5. Validation of the risk score model using the GSE70769 dataset. A. Patients with prostate cancer (PRAD) in the GSE70769 validation dataset are listed in ascending order of risk score. B. Progression-free interval (PFI) distribution versus the risk score of each patient in the GSE707 [file 6768139.f1.zip › 6768139.f1/Table S10.pdf]

ONTOLOGY	ID	Description	GeneRatio	p.adjust	Count
BP	GO:00025	platelet degranulation	17/478	0.000102	17
BP	GO:00024	antigen processing and presentation of exogenous peptide antigen via MHC class I, TAP-dependent	13/478	0.000102	13
BP	GO:00425	antigen processing and presentation of exogenous peptide antigen via MHC class I	13/478	0.00015	13
BP	GO:00421	neutrophil activation	34/478	0.000221	34
BP	GO:00433	neutrophil degranulation	33/478	0.000253	33
BP	GO:00022	neutrophil activation involved in immune response	33/478	0.000253	33
BP	GO:00024	neutrophil mediated immunity	33/478	0.000359	33
BP	GO:00436	regulation of transcription from RNA polymerase II promoter in response to stress	14/478	0.000368	14
BP	GO:00436	regulation of DNA-templated transcription in response to stress	14/478	0.000572	14
BP	GO:00024	antigen processing and presentation of peptide antigen via MHC class I	13/478	0.000572	13
BP	GO:00360	positive regulation of transcription from RNA polymerase II promoter in response to stress	7/478	0.000619	7
BP	GO:00091	purine ribonucleotide metabolic process	28/478	0.000748	28
BP	GO:19904	positive regulation of transcription from RNA polymerase II promoter in response to endoplasmic reticulum stress	5/478	0.001404	5
BP	GO:00092	ribonucleotide metabolic process	28/478	0.001404	28
BP	GO:00196	ribose phosphate metabolic process	28/478	0.001947	28
BP	GO:00725	purine-containing compound metabolic process	29/478	0.001947	29
BP	GO:00620	regulation of small molecule metabolic process	28/478	0.001953	28
BP	GO:00061	purine nucleotide metabolic process	28/478	0.002185	28
BP	GO:00024	antigen processing and presentation of exogenous peptide antigen	16/478	0.002758	16
BP	GO:00364	PERK-mediated unfolded protein response	6/478	0.003993	6
BP	GO:00198	antigen processing and presentation of exogenous antigen	16/478	0.004182	16
BP	GO:00069	vesicle budding from membrane	12/478	0.004182	12
BP	GO:00523	modulation by symbiont of entry into host	8/478	0.004793	8
BP	GO:00603	type I interferon signaling pathway	11/478	0.004793	11
BP	GO:00060	pyruvate metabolic process	14/478	0.004793	14
BP	GO:00300	actin filament-based movement	14/478	0.004793	14
BP	GO:00973	response to alcohol	18/478	0.004793	18
BP	GO:00518	positive regulation of focal adhesion assembly	6/478	0.004793	6
BP	GO:00713	cellular response to type I interferon	11/478	0.004798	11
BP	GO:00067	ATP generation from ADP	12/478	0.005058	12
BP	GO:00329	regulation of actin cytoskeleton organization	23/478	0.005058	23
BP	GO:00480	antigen processing and presentation of peptide antigen	16/478	0.005058	16
BP	GO:00198	antigen processing and presentation of endogenous antigen	6/478	0.005058	6
BP	GO:00091	purine nucleoside diphosphate metabolic process	13/478	0.005058	13
BP	GO:00091	purine ribonucleoside diphosphate metabolic process	13/478	0.005058	13
BP	GO:01404	integrated stress response signaling	6/478	0.005666	6
BP	GO:00198	antigen processing and presentation of endogenous peptide antigen via MHC class I	5/478	0.005666	5
BP	GO:00091	ribonucleoside diphosphate metabolic process	13/478	0.005666	13
BP	GO:00343	response to type I interferon	11/478	0.005666	11
BP	GO:19026	secondary alcohol metabolic process	14/478	0.005666	14
BP	GO:00069	ER-nucleus signaling pathway	8/478	0.005666	8
BP	GO:00329	regulation of actin filament-based process	24/478	0.006428	24
BP	GO:00460	ADP metabolic process	12/478	0.006589	12
BP	GO:01100	regulation of actin filament organization	19/478	0.007457	19
BP	GO:19029	regulation of supramolecular fiber organization	23/478	0.007599	23
BP	GO:00066	cholesterol biosynthetic process	9/478	0.007815	9
BP	GO:19026	secondary alcohol biosynthetic process	9/478	0.007815	9
BP	GO:00060	alcohol metabolic process	23/478	0.008044	23
BP	GO:00024	antigen processing and presentation of endogenous peptide antigen	5/478	0.008051	5
BP	GO:01501	positive regulation of cell-substrate junction organization	6/478	0.008083	6
BP	GO:00309	endoplasmic reticulum unfolded protein response	12/478	0.00821	12
BP	GO:00064	protein folding	17/478	0.00821	17
BP	GO:00463	carboxylic acid biosynthetic process	21/478	0.00821	21
BP	GO:00066	fatty acid biosynthetic process	14/478	0.00821	14
BP	GO:00198	antigen processing and presentation	17/478	0.008406	17
BP	GO:00310	actomyosin structure organization	15/478	0.00883	15
BP	GO:00016	temperature homeostasis	14/478	0.009396	14
CC	GO:00125	ER to Golgi transport vesicle membrane	14/491	1.67E-07	14
CC	GO:00301	COPII-coated ER to Golgi transport vesicle	16/491	3.90E-07	16
CC	GO:00306	secretory granule membrane	26/491	1.13E-05	26
CC	GO:00301	coated vesicle	25/491	1.70E-05	25
CC	GO:00306	transport vesicle membrane	20/491	2.86E-05	20
CC	GO:00059	cell-cell junction	31/491	0.000189	31
CC	GO:00301	transport vesicle	27/491	0.000296	27
CC	GO:00424	melanosome	12/491	0.000824	12
CC	GO:00487	pigment granule	12/491	0.000824	12
CC	GO:00426	MHC protein complex	6/491	0.001439	6
CC	GO:00059	adherens junction	15/491	0.001439	15
CC	GO:00057	peroxisome	13/491	0.001462	13
CC	GO:00425	microbody	13/491	0.001462	13
CC	GO:00453	phagocytic vesicle	13/491	0.001462	13
CC	GO:00306	coated vesicle membrane	15/491	0.00203	15
CC	GO:00347	secretory granule lumen	21/491	0.002313	21
CC	GO:00602	cytoplasmic vesicle lumen	21/491	0.002593	21
CC	GO:00319	vesicle lumen	21/491	0.00267	21
CC	GO:00057	endoplasmic reticulum-Golgi intermediate compartment	12/491	0.002864	12
CC	GO:00058	proteasome core complex	5/491	0.002864	5
CC	GO:00163	apicolateral plasma membrane	5/491	0.003401	5
CC	GO:00308	cortical actin cytoskeleton	9/491	0.003401	9
CC	GO:00059	focal adhesion	24/491	0.003401	24
CC	GO:00300	myofibril	16/491	0.00394	16
CC	GO:00300	cell-substrate junction	24/491	0.004016	24
CC	GO:00432	contractile fiber	16/491	0.005157	16
CC	GO:00057	endoplasmic reticulum lumen	19/491	0.005588	19
CC	GO:00550	recycling endosome membrane	9/491	0.006177	9
CC	GO:00332	eukaryotic 48S preinitiation complex	4/491	0.007643	4
CC	GO:00057	vacuolar lumen	13/491	0.007662	13
CC	GO:00310	platelet alpha granule	9/491	0.007805	9
CC	GO:00057	Golgi-associated vesicle	9/491	0.007938	9
CC	GO:00057	primary lysosome	12/491	0.007938	12
CC	GO:00425	azurophil granule	12/491	0.007938	12
CC	GO:00451	membrane raft	19/491	0.007938	19
CC	GO:00988	membrane microdomain	19/491	0.007938	19
CC	GO:00313	intrinsic component of organelle membrane	22/491	0.007938	22
CC	GO:00301	integral component of endoplasmic reticulum membrane	12/491	0.007938	12
CC	GO:00057	lysosomal membrane	21/491	0.007938	21
CC	GO:00057	early endosome	21/491	0.007938	21
CC	GO:00988	lytic vacuole membrane	21/491	0.007938	21
CC	GO:00306	phagocytic vesicle membrane	8/491	0.008073	8
CC	GO:00715	integral component of lumenal side of endoplasmic reticulum membrane	5/491	0.008073	5
CC	GO:00985	lumenal side of endoplasmic reticulum membrane	5/491	0.008073	5
CC	GO:00058	eukaryotic translation initiation factor 3 complex	4/491	0.008073	4
CC	GO:00162	eukaryotic 43S preinitiation complex	4/491	0.008073	4
CC	GO:00430	costamere	4/491	0.00975	4
CC	GO:00709	translation preinitiation complex	4/491	0.00975	4
MF	GO:00452	cadherin binding	24/475	0.004848	24
