# Supplementary material for: Integrative Analysis of Bulk RNA-Seq and Single-Cell RNA-Seq Unveils the Characteristics of the Immune Microenvironment and Prognosis Signature in Prostate Cancer
Source: J Oncol. 2022 Jul 19;2022:6768139. doi: 10.1155/2022/6768139 (PMC9325591; doi:10.1155/2022/6768139)
Supplement: Supplementary Materials — Figure S1. Workflow of the analysis. Figure S2. Validation of the risk score model using the GSE54460 dataset. A. Patients with prostate cancer (PRAD) in the GSE54460 cohort are listed in ascending order of risk score. B. Progression-free interval (PFI) distribution versus the risk score of each patient in the GSE54460 cohort. C. Kaplan–Meier (KM) curves of patients with different risk levels in the GSE54460 validation set. D. Receiver Operating Characteristic (ROC) curve analysis for 1-, 3- and 5-year PFI using the clinical information of patients of the GSE54460 validation dataset. Figure S3. Validation of the risk score model using the GSE46602 dataset. A. Patients with prostate cancer (PRAD) in the GSE46602 cohort are listed in ascending order of risk score. B. Progression-free interval (PFI) distribution versus the risk score of each patient in the GSE46602 cohort. C. Kaplan–Meier (KM) curves of patients with different risk levels in the GSE46602 validation dataset. D. Receiver Operating Characteristic (ROC) curve analysis for 1-, 3- and 5-year PFI using the clinical information of patients of the GSE46602 validation dataset. Figure S4. Validation of the risk score model using the GSE70768 dataset. A. Patients with prostate cancer (PRAD) in the GSE70768 cohort are listed in ascending order of risk score. B. Progression-free interval (PFI) distribution versus the risk score of each patient in the GSE70768 cohort. C. Kaplan–Meier (KM) curves of patients with different risk levels in the GSE70768 validation dataset. D. Receiver Operating Characteristic (ROC) curve analysis for 1-, 3- and 5-year PFI using the clinical information of patients of the GSE70768 validation dataset. Figure S5. Validation of the risk score model using the GSE70769 dataset. A. Patients with prostate cancer (PRAD) in the GSE70769 validation dataset are listed in ascending order of risk score. B. Progression-free interval (PFI) distribution versus the risk score of each patient in the GSE707 [file 6768139.f1.zip › 6768139.f1/Table S11.pdf]

ONTOLOGY	ID	Description	GeneRatio	p.adjust	Count
BP	GO:0003158	endothelium development	41/1131	1.20E-14	41
BP	GO:0045446	endothelial cell differentiation	37/1131	6.75E-14	37
BP	GO:0002064	epithelial cell development	47/1131	2.29E-12	47
BP	GO:0045785	positive regulation of cell adhesion	68/1131	1.29E-10	68
BP	GO:0001885	endothelial cell development	24/1131	2.04E-10	24
BP	GO:0001667	ameboidal-type cell migration	72/1131	2.04E-10	72
BP	GO:0010810	regulation of cell-substrate adhesion	44/1131	6.53E-10	44
BP	GO:0032970	regulation of actin filament-based process	61/1131	3.02E-09	61
BP	GO:0022604	regulation of cell morphogenesis	52/1131	4.28E-09	52
BP	GO:0051271	negative regulation of cellular component movement	56/1131	1.03E-08	56
BP	GO:0010631	epithelial cell migration	56/1131	1.66E-08	56
BP	GO:0031589	cell-substrate adhesion	56/1131	1.82E-08	56
BP	GO:0030336	negative regulation of cell migration	53/1131	1.82E-08	53
BP	GO:0090132	epithelium migration	56/1131	1.82E-08	56
BP	GO:0032956	regulation of actin cytoskeleton organization	55/1131	2.13E-08	55
BP	GO:0007162	negative regulation of cell adhesion	49/1131	2.48E-08	49
BP	GO:0090130	tissue migration	56/1131	2.48E-08	56
BP	GO:2000146	negative regulation of cell motility	54/1131	2.48E-08	54
BP	GO:0051017	actin filament bundle assembly	33/1131	3.14E-08	33
BP	GO:0061028	establishment of endothelial barrier	18/1131	3.14E-08	18
BP	GO:0007015	actin filament organization	62/1131	4.61E-08	62
BP	GO:0042119	neutrophil activation	68/1131	4.61E-08	68
BP	GO:0061572	actin filament bundle organization	33/1131	5.56E-08	33
BP	GO:0040013	negative regulation of locomotion	56/1131	6.47E-08	56
BP	GO:0043312	neutrophil degranulation	66/1131	7.37E-08	66
BP	GO:0002576	platelet degranulation	29/1131	7.97E-08	29
BP	GO:0002283	neutrophil activation involved in immune response	66/1131	8.82E-08	66
BP	GO:0010811	positive regulation of cell-substrate adhesion	28/1131	8.82E-08	28
BP	GO:0034341	response to interferon-gamma	37/1131	8.96E-08	37
BP	GO:0050673	epithelial cell proliferation	60/1131	1.24E-07	60
BP	GO:0043542	endothelial cell migration	45/1131	1.83E-07	45
BP	GO:0002446	neutrophil mediated immunity	66/1131	1.91E-07	66
BP	GO:0022407	regulation of cell-cell adhesion	60/1131	2.55E-07	60
BP	GO:0060333	interferon-gamma-mediated signaling pathway	23/1131	4.16E-07	23
BP	GO:0010632	regulation of epithelial cell migration	45/1131	8.82E-07	45
BP	GO:0031532	actin cytoskeleton reorganization	24/1131	1.03E-06	24
BP	GO:0044409	entry into host	30/1131	1.08E-06	30
BP	GO:0048545	response to steroid hormone	48/1131	1.54E-06	48
BP	GO:0030856	regulation of epithelial cell differentiation	30/1131	1.64E-06	30
BP	GO:0050678	regulation of epithelial cell proliferation	52/1131	1.70E-06	52
BP	GO:0110053	regulation of actin filament organization	42/1131	2.30E-06	42
BP	GO:0072659	protein localization to plasma membrane	42/1131	2.79E-06	42
BP	GO:0006631	fatty acid metabolic process	53/1131	3.00E-06	53
BP	GO:0030198	extracellular matrix organization	53/1131	3.20E-06	53
BP	GO:0043062	extracellular structure organization	53/1131	3.41E-06	53
BP	GO:0060562	epithelial tube morphogenesis	45/1131	3.51E-06	45
BP	GO:0045229	external encapsulating structure organization	53/1131	3.83E-06	53
BP	GO:0003007	heart morphogenesis	38/1131	3.83E-06	38
BP	GO:1901654	response to ketone	33/1131	5.04E-06	33
BP	GO:0043534	blood vessel endothelial cell migration	31/1131	5.34E-06	31
BP	GO:0052126	movement in host environment	31/1131	5.34E-06	31
BP	GO:0046718	viral entry into host cell	27/1131	6.17E-06	27
BP	GO:1902903	regulation of supramolecular fiber organization	50/1131	6.17E-06	50
BP	GO:0071346	cellular response to interferon-gamma	31/1131	6.61E-06	31
BP	GO:0007044	cell-substrate junction assembly	22/1131	8.58E-06	22
BP	GO:0003382	epithelial cell morphogenesis	12/1131	9.66E-06	12
BP	GO:0001570	vasculogenesis	19/1131	1.13E-05	19
BP	GO:0061138	morphogenesis of a branching epithelium	30/1131	1.25E-05	30
BP	GO:0034329	cell junction assembly	54/1131	1.32E-05	54
BP	GO:0003151	outflow tract morphogenesis	18/1131	1.86E-05	18
BP	GO:0001763	morphogenesis of a branching structure	31/1131	1.86E-05	31
BP	GO:0035633	maintenance of blood-brain barrier	12/1131	1.96E-05	12
BP	GO:0001655	urogenital system development	44/1131	1.96E-05	44
BP	GO:0150115	cell-substrate junction organization	22/1131	2.19E-05	22
BP	GO:0007159	leukocyte cell-cell adhesion	48/1131	2.30E-05	48
BP	GO:0008360	regulation of cell shape	27/1131	2.44E-05	27
BP	GO:0071384	cellular response to corticosteroid stimulus	16/1131	2.50E-05	16
BP	GO:0045216	cell-cell junction organization	33/1131	2.51E-05	33
BP	GO:0045185	maintenance of protein location	20/1131	3.22E-05	20
BP	GO:1903034	regulation of response to wounding	28/1131	3.75E-05	28
BP	GO:0032835	glomerulus development	16/1131	3.83E-05	16
BP	GO:0052372	modulation by symbiont of entry into host	14/1131	3.83E-05	14
BP	GO:0002483	antigen processing and presentation of endogenous peptide antigen	9/1131	3.87E-05	9
BP	GO:0022409	positive regulation of cell-cell adhesion	39/1131	3.98E-05	39
BP	GO:0070371	ERK1 and ERK2 cascade	43/1131	4.26E-05	43
BP	GO:0022612	gland morphogenesis	22/1131	4.27E-05	22
BP	GO:0032507	maintenance of protein location in cell	16/1131	5.51E-05	16
BP	GO:0051893	regulation of focal adhesion assembly	16/1131	5.51E-05	16
BP	GO:0090109	regulation of cell-substrate junction assembly	16/1131	5.51E-05	16
BP	GO:1901653	cellular response to peptide	49/1131	5.51E-05	49
BP	GO:0051701	biological process involved in interaction with host	33/1131	6.28E-05	33
BP	GO:2001233	regulation of apoptotic signaling pathway	45/1131	6.35E-05	45
BP	GO:0007229	integrin-mediated signaling pathway	21/1131	7.07E-05	21
BP	GO:0010594	regulation of endothelial cell migration	34/1131	7.23E-05	34
BP	GO:0031960	response to corticosteroid	26/1131	7.56E-05	26
BP	GO:1990778	protein localization to cell periphery	43/1131	7.89E-05	43
BP	GO:0019883	antigen processing and presentation of endogenous antigen	10/1131	7.94E-05	10
BP	GO:0001952	regulation of cell-matrix adhesion	23/1131	8.13E-05	23
BP	GO:1901342	regulation of vasculature development	44/1131	8.17E-05	44
BP	GO:0071900	regulation of protein serine/threonine kinase activity	57/1131	8.41E-05	57
BP	GO:0034446	substrate adhesion-dependent cell spreading	21/1131	8.88E-05	21
BP	GO:0003018	vascular process in circulatory system	35/1131	9.35E-05	35
BP	GO:0071902	positive regulation of protein serine/threonine kinase activity	41/1131	0.000103	41
BP	GO:0034340	response to type I interferon	20/1131	0.000113	20

BP	GO:0045765	regulation of angiogenesis	43/1131	0.000116	43
BP	GO:0002478	antigen processing and presentation of exogenous peptide antigen	28/1131	0.000132	28
BP	GO:0150116	regulation of cell-substrate junction organization	16/1131	0.000132	16
BP	GO:0001954	positive regulation of cell-matrix adhesion	14/1131	0.000135	14
BP	GO:0006633	fatty acid biosynthetic process	27/1131	0.000142	27
BP	GO:0046394	carboxylic acid biosynthetic process	42/1131	0.000142	42
BP	GO:0001569	branching involved in blood vessel morphogenesis	11/1131	0.000147	11
BP	GO:0052547	regulation of peptidase activity	53/1131	0.000148	53
BP	GO:0060337	type I interferon signaling pathway	19/1131	0.000158	19
BP	GO:0071385	cellular response to glucocorticoid stimulus	14/1131	0.000161	14
BP	GO:1900026	positive regulation of substrate adhesion-dependent cell spreading	12/1131	0.000165	12
BP	GO:0071357	cellular response to type I interferon	19/1131	0.000181	19
BP	GO:1902904	negative regulation of supramolecular fiber organization	26/1131	0.000182	26
BP	GO:0009226	nucleotide-sugar biosynthetic process	9/1131	0.000186	9
BP	GO:0072001	renal system development	38/1131	0.000203	38
BP	GO:0003206	cardiac chamber morphogenesis	21/1131	0.000211	21
BP	GO:0009896	positive regulation of catabolic process	52/1131	0.000212	52
BP	GO:0072330	monocarboxylic acid biosynthetic process	32/1131	0.000213	32
BP	GO:0051098	regulation of binding	44/1131	0.000218	44
BP	GO:0018108	peptidyl-tyrosine phosphorylation	45/1131	0.000224	45
BP	GO:0070372	regulation of ERK1 and ERK2 cascade	39/1131	0.000225	39
BP	GO:0016053	organic acid biosynthetic process	42/1131	0.000226	42
BP	GO:1900024	regulation of substrate adhesion-dependent cell spreading	14/1131	0.000226	14
BP	GO:0048002	antigen processing and presentation of peptide antigen	29/1131	0.000228	29
BP	GO:0051384	response to glucocorticoid	23/1131	0.000236	23
BP	GO:0001822	kidney development	37/1131	0.000238	37
BP	GO:1901888	regulation of cell junction assembly	29/1131	0.000246	29
BP	GO:0072010	glomerular epithelium development	9/1131	0.000248	9
BP	GO:0019884	antigen processing and presentation of exogenous antigen	28/1131	0.000248	28
BP	GO:0018212	peptidyl-tyrosine modification	45/1131	0.000255	45
BP	GO:0043393	regulation of protein binding	29/1131	0.000264	29
BP	GO:0097191	extrinsic apoptotic signaling pathway	31/1131	0.000265	31
BP	GO:0003014	renal system process	21/1131	0.000278	21
BP	GO:0009225	nucleotide-sugar metabolic process	11/1131	0.000294	11
BP	GO:0050730	regulation of peptidyl-tyrosine phosphorylation	35/1131	0.00031	35
BP	GO:0006575	cellular modified amino acid metabolic process	29/1131	0.00031	29
BP	GO:0030041	actin filament polymerization	28/1131	0.000318	28
BP	GO:0010812	negative regulation of cell-substrate adhesion	15/1131	0.000318	15
BP	GO:0051494	negative regulation of cytoskeleton organization	25/1131	0.000325	25
BP	GO:0051894	positive regulation of focal adhesion assembly	9/1131	0.000336	9
BP	GO:0032231	regulation of actin filament bundle assembly	19/1131	0.00036	19
BP	GO:2000249	regulation of actin cytoskeleton reorganization	11/1131	0.000369	11
BP	GO:0048041	focal adhesion assembly	17/1131	0.00039	17
BP	GO:0071383	cellular response to steroid hormone stimulus	29/1131	0.000393	29
BP	GO:0035384	thioester biosynthetic process	13/1131	0.000418	13
BP	GO:0071616	acyl-CoA biosynthetic process	13/1131	0.000418	13
BP	GO:0019882	antigen processing and presentation	32/1131	0.000424	32
BP	GO:0048608	reproductive structure development	47/1131	0.000424	47
BP	GO:0032535	regulation of cellular component size	44/1131	0.000432	44
BP	GO:2000377	regulation of reactive oxygen species metabolic process	28/1131	0.000435	28
BP	GO:0032102	negative regulation of response to external stimulus	46/1131	0.000439	46
BP	GO:0001945	lymph vessel development	9/1131	0.000447	9
BP	GO:0071559	response to transforming growth factor beta	34/1131	0.000451	34
BP	GO:0042176	regulation of protein catabolic process	45/1131	0.000454	45
BP	GO:0031331	positive regulation of cellular catabolic process	45/1131	0.00048	45
BP	GO:0016054	organic acid catabolic process	34/1131	0.00048	34
BP	GO:0061458	reproductive system development	47/1131	0.000484	47
BP	GO:0090066	regulation of anatomical structure size	54/1131	0.000486	54
BP	GO:0048754	branching morphogenesis of an epithelial tube	23/1131	0.000488	23
BP	GO:0008154	actin polymerization or depolymerization	30/1131	0.000488	30
BP	GO:0030857	negative regulation of epithelial cell differentiation	12/1131	0.000529	12
BP	GO:0007009	plasma membrane organization	20/1131	0.000529	20
BP	GO:0033559	unsaturated fatty acid metabolic process	20/1131	0.000529	20
BP	GO:0008064	regulation of actin polymerization or depolymerization	27/1131	0.000556	27
BP	GO:0001765	membrane raft assembly	6/1131	0.00056	6
BP	GO:0010634	positive regulation of epithelial cell migration	26/1131	0.000563	26
BP	GO:0045766	positive regulation of angiogenesis	26/1131	0.000563	26
BP	GO:1904018	positive regulation of vasculature development	26/1131	0.000563	26
BP	GO:0030832	regulation of actin filament length	27/1131	0.000593	27
BP	GO:1903037	regulation of leukocyte cell-cell adhesion	40/1131	0.000593	40
BP	GO:0043405	regulation of MAP kinase activity	38/1131	0.000593	38
BP	GO:0098742	cell-cell adhesion via plasma-membrane adhesion molecules	35/1131	0.000593	35
BP	GO:0071560	cellular response to transforming growth factor beta stimulus	33/1131	0.000603	33
BP	GO:1903036	positive regulation of response to wounding	15/1131	0.000629	15
BP	GO:0043406	positive regulation of MAP kinase activity	31/1131	0.000647	31
BP	GO:0046596	regulation of viral entry into host cell	11/1131	0.000656	11
BP	GO:0072006	nephron development	22/1131	0.000771	22
BP	GO:0070661	leukocyte proliferation	38/1131	0.000819	38
BP	GO:0030833	regulation of actin filament polymerization	25/1131	0.000819	25
BP	GO:0071364	cellular response to epidermal growth factor stimulus	11/1131	0.000822	11
BP	GO:0031345	negative regulation of cell projection organization	26/1131	0.00086	26
BP	GO:0001666	response to hypoxia	41/1131	0.000861	41
BP	GO:0006470	protein dephosphorylation	39/1131	0.000882	39
BP	GO:0032092	positive regulation of protein binding	16/1131	0.000882	16
BP	GO:0019885	antigen processing and presentation of endogenous peptide antigen via MHC class I	7/1131	0.000946	7
BP	GO:0033631	cell-cell adhesion mediated by integrin	7/1131	0.000946	7
BP	GO:0031099	regeneration	27/1131	0.000955	27
BP	GO:0043434	response to peptide hormone	48/1131	0.000994	48
BP	GO:0043123	positive regulation of I-kappaB kinase/NF-kappaB signaling	26/1131	0.000994	26
BP	GO:0003205	cardiac chamber development	23/1131	0.000994	23
BP	GO:0061041	regulation of wound healing	21/1131	0.000994	21
BP	GO:0044272	sulfur compound biosynthetic process	27/1131	0.001015	27
BP	GO:0050731	positive regulation of peptidyl-tyrosine phosphorylation	27/1131	0.001015	27
BP	GO:0042110	T cell activation	51/1131	0.00109	51
BP	GO:0052548	regulation of endopeptidase activity	47/1131	0.001164	47

BP	GO:0035966	response to topologically incorrect protein	28/1131	0.001223	28
BP	GO:0150117	positive regulation of cell-substrate junction organization	9/1131	0.001256	9
BP	GO:0045862	positive regulation of proteolysis	42/1131	0.001256	42
BP	GO:0014065	phosphatidylinositol 3-kinase signaling	23/1131	0.001303	23
BP	GO:0003012	muscle system process	49/1131	0.001303	49
BP	GO:1903793	positive regulation of anion transport	51/1131	0.001309	51
BP	GO:0070293	renal absorption	7/1131	0.001348	7
BP	GO:0070663	regulation of leukocyte proliferation	31/1131	0.001384	31
BP	GO:0003094	glomerular filtration	8/1131	0.001387	8
BP	GO:0007160	cell-matrix adhesion	30/1131	0.001387	30
BP	GO:0045601	regulation of endothelial cell differentiation	11/1131	0.001436	11
BP	GO:0050671	positive regulation of lymphocyte proliferation	21/1131	0.001444	21
BP	GO:0044282	small molecule catabolic process	47/1131	0.001455	47
BP	GO:0001886	endothelial cell morphogenesis	6/1131	0.001459	6
BP	GO:0060841	venous blood vessel development	6/1131	0.001459	6
BP	GO:0001701	in utero embryonic development	39/1131	0.001497	39
BP	GO:1904019	epithelial cell apoptotic process	19/1131	0.00154	19
BP	GO:0046395	carboxylic acid catabolic process	31/1131	0.001542	31
BP	GO:0046949	fatty-acyl-CoA biosynthetic process	9/1131	0.001542	9
BP	GO:0032946	positive regulation of mononuclear cell proliferation	21/1131	0.001549	21
BP	GO:0016049	cell growth	50/1131	0.001549	50
BP	GO:0036293	response to decreased oxygen levels	41/1131	0.001564	41
BP	GO:0043254	regulation of protein-containing complex assembly	48/1131	0.00161	48
BP	GO:0070482	response to oxygen levels	43/1131	0.001634	43
BP	GO:0022408	negative regulation of cell-cell adhesion	26/1131	0.001639	26
BP	GO:0070849	response to epidermal growth factor	11/1131	0.001662	11
BP	GO:1903039	positive regulation of leukocyte cell-cell adhesion	30/1131	0.00176	30
BP	GO:0097205	renal filtration	8/1131	0.00176	8
BP	GO:1905048	regulation of metallopeptidase activity	8/1131	0.00176	8
BP	GO:0070665	positive regulation of leukocyte proliferation	22/1131	0.001796	22
BP	GO:0043001	Golgi to plasma membrane protein transport	10/1131	0.001839	10
BP	GO:0019886	antigen processing and presentation of exogenous peptide antigen via MHC class II	17/1131	0.001887	17
BP	GO:2001234	negative regulation of apoptotic signaling pathway	29/1131	0.001887	29
BP	GO:0051099	positive regulation of binding	24/1131	0.001961	24
BP	GO:0045646	regulation of erythrocyte differentiation	11/1131	0.00197	11
BP	GO:0032271	regulation of protein polymerization	29/1131	0.002018	29
BP	GO:0048015	phosphatidylinositol-mediated signaling	26/1131	0.002026	26
BP	GO:0051258	protein polymerization	35/1131	0.002039	35
BP	GO:0060411	cardiac septum morphogenesis	13/1131	0.002166	13
BP	GO:0016311	dephosphorylation	51/1131	0.002218	51
BP	GO:0010559	regulation of glycoprotein biosynthetic process	12/1131	0.002309	12
BP	GO:2000379	positive regulation of reactive oxygen species metabolic process	17/1131	0.002341	17
BP	GO:0002685	regulation of leukocyte migration	27/1131	0.002349	27
BP	GO:0072073	kidney epithelium development	20/1131	0.002429	20
BP	GO:1901655	cellular response to ketone	16/1131	0.002453	16
BP	GO:0061318	renal filtration cell differentiation	7/1131	0.002453	7
BP	GO:0072112	glomerular visceral epithelial cell differentiation	7/1131	0.002453	7
BP	GO:0002040	sprouting angiogenesis	25/1131	0.002483	25
BP	GO:0006888	endoplasmic reticulum to Golgi vesicle-mediated transport	27/1131	0.002483	27
BP	GO:0042098	T cell proliferation	26/1131	0.002483	26
BP	GO:0010595	positive regulation of endothelial cell migration	20/1131	0.002625	20
BP	GO:0048008	platelet-derived growth factor receptor signaling pathway	12/1131	0.00264	12
BP	GO:0060485	mesenchyme development	34/1131	0.00264	34
BP	GO:0006986	response to unfolded protein	25/1131	0.002647	25
BP	GO:0048017	inositol lipid-mediated signaling	26/1131	0.002648	26
BP	GO:0002042	cell migration involved in sprouting angiogenesis	16/1131	0.002684	16
BP	GO:0002495	antigen processing and presentation of peptide antigen via MHC class II	17/1131	0.002822	17
BP	GO:0060284	regulation of cell development	50/1131	0.002886	50
BP	GO:2001236	regulation of extrinsic apoptotic signaling pathway	22/1131	0.002886	22
BP	GO:0071375	cellular response to peptide hormone stimulus	37/1131	0.002997	37
BP	GO:0010769	regulation of cell morphogenesis involved in differentiation	16/1131	0.002997	16
BP	GO:2000345	regulation of hepatocyte proliferation	6/1131	0.0031	6
BP	GO:2001044	regulation of integrin-mediated signaling pathway	6/1131	0.0031	6
BP	GO:0002504	antigen processing and presentation of peptide or polysaccharide antigen via MHC class II	17/1131	0.0031	17
BP	GO:0034109	homotypic cell-cell adhesion	15/1131	0.003108	15
BP	GO:0035337	fatty-acyl-CoA metabolic process	10/1131	0.003108	10
BP	GO:0050670	regulation of lymphocyte proliferation	28/1131	0.003108	28
BP	GO:0033866	nucleoside bisphosphate biosynthetic process	13/1131	0.003108	13
BP	GO:0034030	ribonucleoside bisphosphate biosynthetic process	13/1131	0.003108	13
BP	GO:0034033	purine nucleoside bisphosphate biosynthetic process	13/1131	0.003108	13
BP	GO:0033327	Leydig cell differentiation	5/1131	0.003108	5
BP	GO:0036303	lymph vessel morphogenesis	7/1131	0.003108	7
BP	GO:0072311	glomerular epithelial cell differentiation	7/1131	0.003108	7
BP	GO:0072574	hepatocyte proliferation	7/1131	0.003108	7
BP	GO:0072575	epithelial cell proliferation involved in liver morphogenesis	7/1131	0.003108	7
BP	GO:2000047	regulation of cell-cell adhesion mediated by cadherin	7/1131	0.003108	7
BP	GO:0060840	artery development	16/1131	0.003201	16
BP	GO:0048732	gland development	44/1131	0.003203	44
BP	GO:0031667	response to nutrient levels	47/1131	0.003258	47
BP	GO:0010038	response to metal ion	39/1131	0.003294	39
BP	GO:0030218	erythrocyte differentiation	18/1131	0.003313	18
BP	GO:0006893	Golgi to plasma membrane transport	12/1131	0.003314	12
BP	GO:0090303	positive regulation of wound healing	12/1131	0.003314	12
BP	GO:0032944	regulation of mononuclear cell proliferation	28/1131	0.003426	28
BP	GO:0044331	cell-cell adhesion mediated by cadherin	8/1131	0.003426	8
BP	GO:0072593	reactive oxygen species metabolic process	33/1131	0.003429	33
BP	GO:0009267	cellular response to starvation	22/1131	0.003448	22
BP	GO:0050870	positive regulation of T cell activation	27/1131	0.003458	27
BP	GO:0003279	cardiac septum development	16/1131	0.003458	16
BP	GO:0048010	vascular endothelial growth factor receptor signaling pathway	16/1131	0.003458	16
BP	GO:0006636	unsaturated fatty acid biosynthetic process	11/1131	0.003458	11
BP	GO:0035967	cellular response to topologically incorrect protein	23/1131	0.003469	23
BP	GO:0021762	substantia nigra development	10/1131	0.003476	10
BP	GO:0072009	nephron epithelium development	17/1131	0.003518	17
BP	GO:0046651	lymphocyte proliferation	33/1131	0.003549	33

BP	GO:0034113	heterotypic cell-cell adhesion	12/1131	0.003728	12
BP	GO:0048857	neural nucleus development	12/1131	0.003728	12
BP	GO:0010770	positive regulation of cell morphogenesis involved in differentiation	14/1131	0.003766	14
BP	GO:0032272	negative regulation of protein polymerization	14/1131	0.003766	14
BP	GO:0003208	cardiac ventricle morphogenesis	13/1131	0.003806	13
BP	GO:0001894	tissue homeostasis	31/1131	0.003851	31
BP	GO:0071709	membrane assembly	9/1131	0.003885	9
BP	GO:0072576	liver morphogenesis	7/1131	0.003885	7
BP	GO:1901685	glutathione derivative metabolic process	7/1131	0.003885	7
BP	GO:1901687	glutathione derivative biosynthetic process	7/1131	0.003885	7
BP	GO:1902905	positive regulation of supramolecular fiber organization	26/1131	0.003885	26
BP	GO:0034612	response to tumor necrosis factor	36/1131	0.003885	36
BP	GO:0034976	response to endoplasmic reticulum stress	34/1131	0.003885	34
BP	GO:0071356	cellular response to tumor necrosis factor	34/1131	0.003885	34
BP	GO:0048872	homeostasis of number of cells	30/1131	0.003911	30
BP	GO:0030099	myeloid cell differentiation	44/1131	0.003923	44
BP	GO:0010232	vascular transport	15/1131	0.003923	15
BP	GO:0002693	positive regulation of cellular extravasation	6/1131	0.003923	6
BP	GO:0006047	UDP-N-acetylglucosamine metabolic process	6/1131	0.003923	6
BP	GO:0043491	protein kinase B signaling	32/1131	0.003999	32
BP	GO:0034620	cellular response to unfolded protein	21/1131	0.004001	21
BP	GO:0032943	mononuclear cell proliferation	33/1131	0.004002	33
BP	GO:0001916	positive regulation of T cell mediated cytotoxicity	8/1131	0.004002	8
BP	GO:0110020	regulation of actomyosin structure organization	16/1131	0.004011	16
BP	GO:0048145	regulation of fibroblast proliferation	14/1131	0.004035	14
BP	GO:0050680	negative regulation of epithelial cell proliferation	22/1131	0.004044	22
BP	GO:0010639	negative regulation of organelle organization	38/1131	0.004082	38
BP	GO:0045123	cellular extravasation	13/1131	0.004082	13
BP	GO:0051145	smooth muscle cell differentiation	13/1131	0.004082	13
BP	GO:0031333	negative regulation of protein-containing complex assembly	20/1131	0.004105	20
BP	GO:0060537	muscle tissue development	40/1131	0.004144	40
BP	GO:0043535	regulation of blood vessel endothelial cell migration	21/1131	0.004242	21
BP	GO:0048193	Golgi vesicle transport	40/1131	0.004354	40
BP	GO:0006900	vesicle budding from membrane	17/1131	0.004419	17
BP	GO:0045602	negative regulation of endothelial cell differentiation	5/1131	0.004438	5
BP	GO:0046598	positive regulation of viral entry into host cell	5/1131	0.004438	5
BP	GO:0075294	positive regulation by symbiont of entry into host	5/1131	0.004438	5
BP	GO:0048144	fibroblast proliferation	14/1131	0.004438	14
BP	GO:0006066	alcohol metabolic process	40/1131	0.004499	40
BP	GO:0001974	blood vessel remodeling	10/1131	0.004499	10
BP	GO:0030835	negative regulation of actin filament depolymerization	10/1131	0.004499	10
BP	GO:0032233	positive regulation of actin filament bundle assembly	12/1131	0.004506	12
BP	GO:1903018	regulation of glycoprotein metabolic process	12/1131	0.004506	12
BP	GO:0051222	positive regulation of protein transport	35/1131	0.004526	35
BP	GO:0097066	response to thyroid hormone	7/1131	0.004748	7
BP	GO:0007411	axon guidance	32/1131	0.004794	32
BP	GO:0042594	response to starvation	25/1131	0.004844	25
BP	GO:0051051	negative regulation of transport	45/1131	0.004931	45
BP	GO:2000649	regulation of sodium ion transmembrane transporter activity	11/1131	0.004954	11
BP	GO:0097485	neuron projection guidance	32/1131	0.005046	32
BP	GO:0009991	response to extracellular stimulus	48/1131	0.005046	48
BP	GO:0014706	striated muscle tissue development	38/1131	0.005051	38
BP	GO:0006695	cholesterol biosynthetic process	13/1131	0.005051	13
BP	GO:1902653	secondary alcohol biosynthetic process	13/1131	0.005051	13
BP	GO:0034101	erythrocyte homeostasis	18/1131	0.005051	18
BP	GO:0001946	lymphangiogenesis	6/1131	0.005099	6
BP	GO:0006595	polyamine metabolic process	6/1131	0.005099	6
BP	GO:0043116	negative regulation of vascular permeability	6/1131	0.005099	6
BP	GO:0007219	Notch signaling pathway	24/1131	0.005143	24
BP	GO:0048146	positive regulation of fibroblast proliferation	10/1131	0.005143	10
BP	GO:0048771	tissue remodeling	23/1131	0.005175	23
BP	GO:0071496	cellular response to external stimulus	34/1131	0.005184	34
BP	GO:0031529	ruffle organization	11/1131	0.005625	11
BP	GO:0070374	positive regulation of ERK1 and ERK2 cascade	26/1131	0.005644	26
BP	GO:0010951	negative regulation of endopeptidase activity	29/1131	0.005682	29
BP	GO:0002687	positive regulation of leukocyte migration	19/1131	0.005682	19
BP	GO:0050729	positive regulation of inflammatory response	19/1131	0.005682	19
BP	GO:0030837	negative regulation of actin filament polymerization	12/1131	0.005682	12
BP	GO:1902305	regulation of sodium ion transmembrane transport	12/1131	0.005682	12
BP	GO:0035456	response to interferon-beta	8/1131	0.005731	8
BP	GO:0010560	positive regulation of glycoprotein biosynthetic process	7/1131	0.005876	7
BP	GO:1903901	negative regulation of viral life cycle	7/1131	0.005876	7
BP	GO:0051346	negative regulation of hydrolase activity	46/1131	0.005931	46
BP	GO:0010952	positive regulation of peptidase activity	25/1131	0.006104	25
BP	GO:0008584	male gonad development	19/1131	0.006146	19
BP	GO:0007163	establishment or maintenance of cell polarity	26/1131	0.006324	26
BP	GO:0022900	electron transport chain	23/1131	0.006328	23
BP	GO:0072015	glomerular visceral epithelial cell development	5/1131	0.006406	5
BP	GO:0098911	regulation of ventricular cardiac muscle cell action potential	5/1131	0.006406	5
BP	GO:0000302	response to reactive oxygen species	27/1131	0.006435	27
BP	GO:0050920	regulation of chemotaxis	27/1131	0.006435	27
BP	GO:0003231	cardiac ventricle development	17/1131	0.006593	17
BP	GO:0045664	regulation of neuron differentiation	24/1131	0.006593	24
BP	GO:0046546	development of primary male sexual characteristics	19/1131	0.006593	19
BP	GO:0002456	T cell mediated immunity	16/1131	0.006661	16
BP	GO:0035335	peptidyl-tyrosine dephosphorylation	16/1131	0.006661	16
BP	GO:1904035	regulation of epithelial cell apoptotic process	15/1131	0.006661	15
BP	GO:0061298	retina vasculature development in camera-type eye	6/1131	0.006677	6
BP	GO:0042129	regulation of T cell proliferation	22/1131	0.006721	22
BP	GO:0045732	positive regulation of protein catabolic process	27/1131	0.006739	27
BP	GO:0001913	T cell mediated cytotoxicity	10/1131	0.00677	10
BP	GO:0035850	epithelial cell differentiation involved in kidney development	10/1131	0.00677	10
BP	GO:0098801	regulation of renal system process	8/1131	0.006786	8
BP	GO:0032355	response to estradiol	18/1131	0.006786	18
BP	GO:0031032	actomyosin structure organization	24/1131	0.00691	24

BP	GO:0034332	adherens junction organization	12/1131	0.007078	12
BP	GO:0043087	regulation of GTPase activity	48/1131	0.007078	48
BP	GO:0050679	positive regulation of epithelial cell proliferation	25/1131	0.007085	25
BP	GO:1902652	secondary alcohol metabolic process	21/1131	0.007085	21
BP	GO:0006637	acyl-CoA metabolic process	16/1131	0.007085	16
BP	GO:0035383	thioester metabolic process	16/1131	0.007085	16
BP	GO:0017145	stem cell division	7/1131	0.007085	7
BP	GO:0031579	membrane raft organization	7/1131	0.007085	7
BP	GO:0072012	glomerulus vasculature development	7/1131	0.007085	7
BP	GO:1900120	regulation of receptor binding	7/1131	0.007085	7
BP	GO:0006119	oxidative phosphorylation	20/1131	0.007621	20
BP	GO:0038066	p38MAPK cascade	10/1131	0.007746	10
BP	GO:0048661	positive regulation of smooth muscle cell proliferation	15/1131	0.007905	15
BP	GO:0014066	regulation of phosphatidylinositol 3-kinase signaling	18/1131	0.007905	18
BP	GO:0030968	endoplasmic reticulum unfolded protein response	18/1131	0.007905	18
BP	GO:0061005	cell differentiation involved in kidney development	11/1131	0.007976	11
BP	GO:0048660	regulation of smooth muscle cell proliferation	21/1131	0.008166	21
BP	GO:0044091	membrane biogenesis	9/1131	0.008166	9
BP	GO:0002028	regulation of sodium ion transport	14/1131	0.008694	14
BP	GO:0150104	transport across blood-brain barrier	14/1131	0.008694	14
BP	GO:0050863	regulation of T cell activation	35/1131	0.008827	35
BP	GO:0015980	energy derivation by oxidation of organic compounds	31/1131	0.008827	31
BP	GO:0061437	renal system vasculature development	7/1131	0.008827	7
BP	GO:0061440	kidney vasculature development	7/1131	0.008827	7
BP	GO:0070671	response to interleukin-12	10/1131	0.008827	10
BP	GO:1904036	negative regulation of epithelial cell apoptotic process	10/1131	0.008827	10
BP	GO:0006596	polyamine biosynthetic process	5/1131	0.008827	5
BP	GO:0072310	glomerular epithelial cell development	5/1131	0.008827	5
BP	GO:0002683	negative regulation of immune system process	41/1131	0.00886	41
BP	GO:0045931	positive regulation of mitotic cell cycle	17/1131	0.00886	17
BP	GO:0061951	establishment of protein localization to plasma membrane	11/1131	0.008942	11
BP	GO:0006790	sulfur compound metabolic process	39/1131	0.009012	39
BP	GO:0032388	positive regulation of intracellular transport	26/1131	0.009083	26
BP	GO:1904951	positive regulation of establishment of protein localization	35/1131	0.009083	35
BP	GO:0048762	mesenchymal cell differentiation	27/1131	0.009173	27
BP	GO:0016126	sterol biosynthetic process	13/1131	0.009173	13
BP	GO:0048659	smooth muscle cell proliferation	21/1131	0.009189	21
BP	GO:0007568	aging	33/1131	0.009389	33
BP	GO:0072522	purine-containing compound biosynthetic process	25/1131	0.009393	25
BP	GO:0003143	embryonic heart tube morphogenesis	12/1131	0.009733	12
BP	GO:0043122	regulation of I-kappaB kinase/NF-kappaB signaling	28/1131	0.009733	28
BP	GO:0035264	multicellular organism growth	18/1131	0.009805	18
BP	GO:0001558	regulation of cell growth	41/1131	0.009938	41
BP	GO:0010718	positive regulation of epithelial to mesenchymal transition	10/1131	0.009985	10
CC	GO:0005925	focal adhesion	82/1167	2.23E-19	82
CC	GO:0030055	cell-substrate junction	82/1167	3.41E-19	82
CC	GO:0005911	cell-cell junction	81/1167	5.04E-15	81
CC	GO:0005912	adherens junction	41/1167	1.97E-12	41
CC	GO:0030134	COPII-coated ER to Golgi transport vesicle	29/1167	9.76E-12	29
CC	GO:0042611	MHC protein complex	15/1167	7.53E-11	15
CC	GO:0045121	membrane raft	54/1167	4.17E-10	54
CC	GO:0098857	membrane microdomain	54/1167	4.17E-10	54
CC	GO:0012507	ER to Golgi transport vesicle membrane	21/1167	1.97E-09	21
CC	GO:0034774	secretory granule lumen	50/1167	3.00E-08	50
CC	GO:0030135	coated vesicle	47/1167	3.80E-08	47
CC	GO:0060205	cytoplasmic vesicle lumen	50/1167	3.87E-08	50
CC	GO:0031983	vesicle lumen	50/1167	4.43E-08	50
CC	GO:0071556	integral component of lumenal side of endoplasmic reticulum membrane	13/1167	1.28E-07	13
CC	GO:0098553	lumenal side of endoplasmic reticulum membrane	13/1167	1.28E-07	13
CC	GO:0030658	transport vesicle membrane	34/1167	2.44E-06	34
CC	GO:0042613	MHC class II protein complex	9/1167	2.46E-06	9
CC	GO:0098576	lumenal side of membrane	13/1167	2.46E-06	13
CC	GO:0030662	coated vesicle membrane	31/1167	3.73E-06	31
CC	GO:0030133	transport vesicle	52/1167	3.77E-06	52
CC	GO:0005774	vacuolar membrane	54/1167	5.91E-06	54
CC	GO:0031093	platelet alpha granule lumen	17/1167	7.52E-06	17
CC	GO:0030864	cortical actin cytoskeleton	18/1167	1.50E-05	18
CC	GO:0030863	cortical cytoskeleton	21/1167	1.84E-05	21
CC	GO:0005759	mitochondrial matrix	56/1167	2.33E-05	56
CC	GO:0031091	platelet alpha granule	19/1167	3.34E-05	19
CC	GO:0005765	lysosomal membrane	46/1167	7.76E-05	46
CC	GO:0098852	lytic vacuole membrane	46/1167	7.76E-05	46
CC	GO:0045177	apical part of cell	49/1167	8.02E-05	49
CC	GO:0030666	endocytic vesicle membrane	26/1167	8.56E-05	26
CC	GO:0070820	tertiary granule	26/1167	9.30E-05	26
CC	GO:0032432	actin filament bundle	16/1167	9.31E-05	16
CC	GO:0016324	apical plasma membrane	43/1167	0.000114	43
CC	GO:0031252	cell leading edge	48/1167	0.000123	48
CC	GO:0030669	clathrin-coated endocytic vesicle membrane	11/1167	0.000201	11
CC	GO:0062023	collagen-containing extracellular matrix	48/1167	0.000248	48
CC	GO:0101002	ficolin-1-rich granule	27/1167	0.000248	27
CC	GO:0016327	apicolateral plasma membrane	8/1167	0.000253	8
CC	GO:0031300	intrinsic component of organelle membrane	46/1167	0.00028	46
CC	GO:0001725	stress fiber	14/1167	0.000288	14
CC	GO:0097517	contractile actin filament bundle	14/1167	0.000288	14
CC	GO:0030176	integral component of endoplasmic reticulum membrane	24/1167	0.000294	24
CC	GO:0030667	secretory granule membrane	37/1167	0.000472	37
CC	GO:0005788	endoplasmic reticulum lumen	37/1167	0.000495	37
CC	GO:1904813	ficolin-1-rich granule lumen	20/1167	0.000601	20
CC	GO:0031227	intrinsic component of endoplasmic reticulum membrane	24/1167	0.000614	24
CC	GO:0005743	mitochondrial inner membrane	52/1167	0.000636	52
CC	GO:0031301	integral component of organelle membrane	42/1167	0.00064	42
CC	GO:0042470	melanosome	18/1167	0.00064	18
CC	GO:0048770	pigment granule	18/1167	0.00064	18
CC	GO:0005938	cell cortex	36/1167	0.000681	36

CC	GO:0045178	basal part of cell	32/1167	0.000845	32
CC	GO:0005793	endoplasmic reticulum-Golgi intermediate compartment	20/1167	0.00101	20
CC	GO:0005802	trans-Golgi network	31/1167	0.001134	31
CC	GO:0042641	actomyosin	14/1167	0.001168	14
CC	GO:0098798	mitochondrial protein-containing complex	32/1167	0.001305	32
CC	GO:0009897	external side of plasma membrane	43/1167	0.001677	43
CC	GO:0009925	basal plasma membrane	29/1167	0.002538	29
CC	GO:0005901	caveola	14/1167	0.002577	14
CC	GO:0001726	ruffle	23/1167	0.003133	23
CC	GO:1904724	tertiary granule lumen	11/1167	0.00342	11
CC	GO:0005766	primary lysosome	21/1167	0.003535	21
CC	GO:0042582	azurophil granule	21/1167	0.003535	21
CC	GO:0005884	actin filament	17/1167	0.003601	17
CC	GO:0030175	filopodium	16/1167	0.003607	16
CC	GO:0030139	endocytic vesicle	34/1167	0.003607	34
CC	GO:0009898	cytoplasmic side of plasma membrane	22/1167	0.00363	22
CC	GO:0005604	basement membrane	15/1167	0.003864	15
CC	GO:0045334	clathrin-coated endocytic vesicle	11/1167	0.004179	11
CC	GO:0030136	clathrin-coated vesicle	24/1167	0.004252	24
CC	GO:0043296	apical junction complex	19/1167	0.004359	19
CC	GO:0098858	actin-based cell projection	26/1167	0.004431	26
CC	GO:0042581	specific granule	21/1167	0.004689	21
CC	GO:0030670	phagocytic vesicle membrane	13/1167	0.004832	13
CC	GO:0001931	uropod	5/1167	0.005109	5
CC	GO:0031254	cell trailing edge	5/1167	0.005109	5
CC	GO:0044291	cell-cell contact zone	12/1167	0.005667	12
CC	GO:0005750	mitochondrial respiratory chain complex III	5/1167	0.00727	5
CC	GO:0045275	respiratory chain complex III	5/1167	0.00727	5
CC	GO:0016328	lateral plasma membrane	11/1167	0.007556	11
CC	GO:0098562	cytoplasmic side of membrane	23/1167	0.007895	23
CC	GO:0005770	late endosome	30/1167	0.007895	30
CC	GO:0070469	respirasome	15/1167	0.008341	15
CC	GO:0019897	extrinsic component of plasma membrane	21/1167	0.008341	21
MF	GO:0045296	cadherin binding	65/1136	6.21E-14	65
MF	GO:0098631	cell adhesion mediator activity	21/1136	6.02E-09	21
MF	GO:0098632	cell-cell adhesion mediator activity	18/1136	8.00E-08	18
MF	GO:0003779	actin binding	63/1136	8.00E-08	63
MF	GO:0098641	cadherin binding involved in cell-cell adhesion	11/1136	1.91E-07	11
MF	GO:0042605	peptide antigen binding	14/1136	2.83E-07	14
MF	GO:0019838	growth factor binding	29/1136	5.60E-07	29
MF	GO:0005178	integrin binding	29/1136	1.16E-06	29
MF	GO:0033218	amide binding	53/1136	6.34E-06	53
MF	GO:0042277	peptide binding	42/1136	0.000203	42
MF	GO:0019199	transmembrane receptor protein kinase activity	17/1136	0.000529	17
MF	GO:0051015	actin filament binding	30/1136	0.00101	30
MF	GO:0016614	oxidoreductase activity, acting on CH-OH group of donors	21/1136	0.003374	21
MF	GO:0046332	SMAD binding	15/1136	0.003985	15
MF	GO:0005024	transforming growth factor beta-activated receptor activity	6/1136	0.003985	6
MF	GO:0019955	cytokine binding	21/1136	0.005374	21
MF	GO:0044325	ion channel binding	20/1136	0.006227	20
MF	GO:0016616	oxidoreductase activity, acting on the CH-OH group of donors, NAD or NADP as acceptor	19/1136	0.007217	19
MF	GO:0032395	MHC class II receptor activity	5/1136	0.008406	5
