# Supplementary material for: Integrative Analysis of Bulk RNA-Seq and Single-Cell RNA-Seq Unveils the Characteristics of the Immune Microenvironment and Prognosis Signature in Prostate Cancer
Source: J Oncol. 2022 Jul 19;2022:6768139. doi: 10.1155/2022/6768139 (PMC9325591; doi:10.1155/2022/6768139)
Supplement: Supplementary Materials — Figure S1. Workflow of the analysis. Figure S2. Validation of the risk score model using the GSE54460 dataset. A. Patients with prostate cancer (PRAD) in the GSE54460 cohort are listed in ascending order of risk score. B. Progression-free interval (PFI) distribution versus the risk score of each patient in the GSE54460 cohort. C. Kaplan–Meier (KM) curves of patients with different risk levels in the GSE54460 validation set. D. Receiver Operating Characteristic (ROC) curve analysis for 1-, 3- and 5-year PFI using the clinical information of patients of the GSE54460 validation dataset. Figure S3. Validation of the risk score model using the GSE46602 dataset. A. Patients with prostate cancer (PRAD) in the GSE46602 cohort are listed in ascending order of risk score. B. Progression-free interval (PFI) distribution versus the risk score of each patient in the GSE46602 cohort. C. Kaplan–Meier (KM) curves of patients with different risk levels in the GSE46602 validation dataset. D. Receiver Operating Characteristic (ROC) curve analysis for 1-, 3- and 5-year PFI using the clinical information of patients of the GSE46602 validation dataset. Figure S4. Validation of the risk score model using the GSE70768 dataset. A. Patients with prostate cancer (PRAD) in the GSE70768 cohort are listed in ascending order of risk score. B. Progression-free interval (PFI) distribution versus the risk score of each patient in the GSE70768 cohort. C. Kaplan–Meier (KM) curves of patients with different risk levels in the GSE70768 validation dataset. D. Receiver Operating Characteristic (ROC) curve analysis for 1-, 3- and 5-year PFI using the clinical information of patients of the GSE70768 validation dataset. Figure S5. Validation of the risk score model using the GSE70769 dataset. A. Patients with prostate cancer (PRAD) in the GSE70769 validation dataset are listed in ascending order of risk score. B. Progression-free interval (PFI) distribution versus the risk score of each patient in the GSE707 [file 6768139.f1.zip › 6768139.f1/Table S12.pdf]

ONTOLOGY	ID	Description	GeneRatio	p.adjust	Count
BP	GO:0030198	extracellular matrix organization	58/972	9.69E-10	58
BP	GO:0043062	extracellular structure organization	58/972	9.69E-10	58
BP	GO:0045229	external encapsulating structure organization	58/972	9.69E-10	58
BP	GO:0032970	regulation of actin filament-based process	57/972	1.14E-09	57
BP	GO:0001667	ameboidal-type cell migration	63/972	3.63E-09	63
BP	GO:0031589	cell-substrate adhesion	53/972	3.63E-09	53
BP	GO:0010810	regulation of cell-substrate adhesion	39/972	6.18E-09	39
BP	GO:0002576	platelet degranulation	29/972	6.79E-09	29
BP	GO:0007015	actin filament organization	57/972	4.87E-08	57
BP	GO:0051271	negative regulation of cellular component movement	48/972	3.32E-07	48
BP	GO:0043312	neutrophil degranulation	59/972	3.32E-07	59
BP	GO:0030336	negative regulation of cell migration	46/972	3.32E-07	46
BP	GO:0002446	neutrophil mediated immunity	60/972	3.32E-07	60
BP	GO:0042119	neutrophil activation	60/972	3.32E-07	60
BP	GO:0002283	neutrophil activation involved in immune response	59/972	3.32E-07	59
BP	GO:2000146	negative regulation of cell motility	47/972	3.47E-07	47
BP	GO:0003012	muscle system process	56/972	3.47E-07	56
BP	GO:0006936	muscle contraction	47/972	5.79E-07	47
BP	GO:0032956	regulation of actin cytoskeleton organization	47/972	5.79E-07	47
BP	GO:0150115	cell-substrate junction organization	23/972	9.12E-07	23
BP	GO:1901654	response to ketone	32/972	1.10E-06	32
BP	GO:0051017	actin filament bundle assembly	28/972	1.19E-06	28
BP	GO:0040013	negative regulation of locomotion	48/972	1.49E-06	48
BP	GO:0090130	tissue migration	47/972	1.49E-06	47
BP	GO:0034109	homotypic cell-cell adhesion	20/972	1.75E-06	20
BP	GO:0061572	actin filament bundle organization	28/972	1.81E-06	28
BP	GO:0010631	epithelial cell migration	46/972	1.88E-06	46
BP	GO:0090132	epithelium migration	46/972	2.35E-06	46
BP	GO:0007229	integrin-mediated signaling pathway	22/972	3.27E-06	22
BP	GO:0110053	regulation of actin filament organization	38/972	4.17E-06	38
BP	GO:0002064	epithelial cell development	32/972	4.24E-06	32
BP	GO:0072659	protein localization to plasma membrane	38/972	4.67E-06	38
BP	GO:1902903	regulation of supramolecular fiber organization	46/972	4.67E-06	46
BP	GO:0030048	actin filament-based movement	26/972	7.47E-06	26
BP	GO:0150116	regulation of cell-substrate junction organization	17/972	7.86E-06	17
BP	GO:0022604	regulation of cell morphogenesis	40/972	7.98E-06	40
BP	GO:0048608	reproductive structure development	48/972	9.38E-06	48
BP	GO:0001655	urogenital system development	41/972	9.95E-06	41
BP	GO:0061458	reproductive system development	48/972	1.12E-05	48
BP	GO:0003158	endothelium development	24/972	1.68E-05	24
BP	GO:0007044	cell-substrate junction assembly	20/972	1.94E-05	20
BP	GO:1990778	protein localization to cell periphery	41/972	1.94E-05	41
BP	GO:1902905	positive regulation of supramolecular fiber organization	30/972	2.30E-05	30
BP	GO:0044272	sulfur compound biosynthetic process	29/972	2.50E-05	29
BP	GO:0010811	positive regulation of cell-substrate adhesion	22/972	2.53E-05	22
BP	GO:0044409	entry into host	25/972	3.24E-05	25
BP	GO:0043542	endothelial cell migration	36/972	3.85E-05	36
BP	GO:0034446	substrate adhesion-dependent cell spreading	20/972	5.95E-05	20
BP	GO:0031032	actomyosin structure organization	28/972	5.95E-05	28
BP	GO:0043434	response to peptide hormone	48/972	5.95E-05	48
BP	GO:0052126	movement in host environment	26/972	0.000113	26
BP	GO:0010632	regulation of epithelial cell migration	36/972	0.000125	36
BP	GO:0060537	muscle tissue development	42/972	0.000146	42
BP	GO:0048732	gland development	45/972	0.000175	45
BP	GO:0010038	response to metal ion	40/972	0.000221	40
BP	GO:1901653	cellular response to peptide	43/972	0.000221	43
BP	GO:0006790	sulfur compound metabolic process	42/972	0.000221	42
BP	GO:0045446	endothelial cell differentiation	20/972	0.000245	20
BP	GO:0007160	cell-matrix adhesion	30/972	0.000245	30
BP	GO:0046718	viral entry into host cell	22/972	0.000245	22
BP	GO:0043001	Golgi to plasma membrane protein transport	11/972	0.000245	11
BP	GO:0008360	regulation of cell shape	23/972	0.000248	23
BP	GO:0048545	response to steroid hormone	38/972	0.000257	38
BP	GO:0051495	positive regulation of cytoskeleton organization	29/972	0.000268	29
BP	GO:0061041	regulation of wound healing	21/972	0.000276	21
BP	GO:0071559	response to transforming growth factor beta	32/972	0.000277	32
BP	GO:0051893	regulation of focal adhesion assembly	14/972	0.000277	14
BP	GO:0090109	regulation of cell-substrate junction assembly	14/972	0.000277	14
BP	GO:0022612	gland morphogenesis	19/972	0.000277	19
BP	GO:1903034	regulation of response to wounding	24/972	0.000277	24
BP	GO:0034329	cell junction assembly	45/972	0.000282	45
BP	GO:0045785	positive regulation of cell adhesion	45/972	0.000282	45
BP	GO:0007163	establishment or maintenance of cell polarity	28/972	0.000333	28
BP	GO:1900024	regulation of substrate adhesion-dependent cell spreading	13/972	0.00035	13
BP	GO:0071375	cellular response to peptide hormone stimulus	37/972	0.000379	37
BP	GO:0001952	regulation of cell-matrix adhesion	20/972	0.000409	20
BP	GO:0048041	focal adhesion assembly	16/972	0.000409	16
BP	GO:0009636	response to toxic substance	30/972	0.000409	30
BP	GO:0007009	plasma membrane organization	19/972	0.000418	19
BP	GO:0006893	Golgi to plasma membrane transport	13/972	0.000486	13
BP	GO:0098754	detoxification	21/972	0.000512	21
BP	GO:1901685	glutathione derivative metabolic process	8/972	0.000512	8
BP	GO:1901687	glutathione derivative biosynthetic process	8/972	0.000512	8
BP	GO:0051701	biological process involved in interaction with host	28/972	0.000542	28
BP	GO:0007162	negative regulation of cell adhesion	34/972	0.000611	34
BP	GO:0010761	fibroblast migration	11/972	0.000622	11
BP	GO:0045216	cell-cell junction organization	27/972	0.000622	27
BP	GO:0001892	embryonic placenta development	15/972	0.000626	15

BP	GO:0016049	cell growth	47/972	0.000649	47
BP	GO:0070252	actin-mediated cell contraction	19/972	0.000692	19
BP	GO:0035384	thioester biosynthetic process	12/972	0.000704	12
BP	GO:0071616	acyl-CoA biosynthetic process	12/972	0.000704	12
BP	GO:0098911	regulation of ventricular cardiac muscle cell action potential	6/972	0.000736	6
BP	GO:0032835	glomerulus development	13/972	0.000736	13
BP	GO:0060485	mesenchyme development	33/972	0.000772	33
BP	GO:0006695	cholesterol biosynthetic process	14/972	0.000828	14
BP	GO:1902653	secondary alcohol biosynthetic process	14/972	0.000828	14
BP	GO:1903036	positive regulation of response to wounding	14/972	0.000828	14
BP	GO:0045664	regulation of neuron differentiation	25/972	0.000836	25
BP	GO:0006882	cellular zinc ion homeostasis	10/972	0.000837	10
BP	GO:0071900	regulation of protein serine/threonine kinase activity	48/972	0.000886	48
BP	GO:0003382	epithelial cell morphogenesis	9/972	0.000916	9
BP	GO:0090066	regulation of anatomical structure size	48/972	0.000916	48
BP	GO:0048015	phosphatidylinositol-mediated signaling	25/972	0.000954	25
BP	GO:0051604	protein maturation	33/972	0.001071	33
BP	GO:0052547	regulation of peptidase activity	45/972	0.001099	45
BP	GO:0051764	actin crosslink formation	6/972	0.001137	6
BP	GO:0010594	regulation of endothelial cell migration	28/972	0.001146	28
BP	GO:0052372	modulation by symbiont of entry into host	11/972	0.001209	11
BP	GO:0055069	zinc ion homeostasis	10/972	0.001242	10
BP	GO:0034976	response to endoplasmic reticulum stress	33/972	0.001246	33
BP	GO:0048017	inositol lipid-mediated signaling	25/972	0.001246	25
BP	GO:0086065	cell communication involved in cardiac conduction	12/972	0.001246	12
BP	GO:0014066	regulation of phosphatidylinositol 3-kinase signaling	19/972	0.001246	19
BP	GO:0030099	myeloid cell differentiation	42/972	0.001379	42
BP	GO:0007265	Ras protein signal transduction	36/972	0.001407	36
BP	GO:0010812	negative regulation of cell-substrate adhesion	13/972	0.001422	13
BP	GO:0030865	cortical cytoskeleton organization	12/972	0.001424	12
BP	GO:0030850	prostate gland development	10/972	0.001424	10
BP	GO:0033574	response to testosterone	10/972	0.001424	10
BP	GO:1900026	positive regulation of substrate adhesion-dependent cell spreading	10/972	0.001424	10
BP	GO:0002028	regulation of sodium ion transport	15/972	0.001499	15
BP	GO:0016485	protein processing	27/972	0.001544	27
BP	GO:0072001	renal system development	32/972	0.001573	32
BP	GO:1901342	regulation of vasculature development	36/972	0.001574	36
BP	GO:0061951	establishment of protein localization to plasma membrane	12/972	0.001608	12
BP	GO:0090303	positive regulation of wound healing	12/972	0.001608	12
BP	GO:0016126	sterol biosynthetic process	14/972	0.00163	14
BP	GO:0071364	cellular response to epidermal growth factor stimulus	10/972	0.001673	10
BP	GO:0071560	cellular response to transforming growth factor beta stimulus	29/972	0.001682	29
BP	GO:0014065	phosphatidylinositol 3-kinase signaling	21/972	0.001736	21
BP	GO:0098901	regulation of cardiac muscle cell action potential	8/972	0.001776	8
BP	GO:0070482	response to oxygen levels	39/972	0.001801	39
BP	GO:0050673	epithelial cell proliferation	42/972	0.001937	42
BP	GO:0021762	substantia nigra development	10/972	0.001988	10
BP	GO:0001822	kidney development	31/972	0.001998	31
BP	GO:0014068	positive regulation of phosphatidylinositol 3-kinase signaling	15/972	0.001998	15
BP	GO:0030856	regulation of epithelial cell differentiation	21/972	0.001998	21
BP	GO:0031345	negative regulation of cell projection organization	23/972	0.002047	23
BP	GO:0019216	regulation of lipid metabolic process	40/972	0.002049	40
BP	GO:0046686	response to cadmium ion	12/972	0.00205	12
BP	GO:0045010	actin nucleation	11/972	0.002054	11
BP	GO:0010273	detoxification of copper ion	6/972	0.002239	6
BP	GO:0010763	positive regulation of fibroblast migration	6/972	0.002239	6
BP	GO:1990169	stress response to copper ion	6/972	0.002239	6
BP	GO:0001890	placenta development	19/972	0.002284	19
BP	GO:0032231	regulation of actin filament bundle assembly	16/972	0.002284	16
BP	GO:0006631	fatty acid metabolic process	39/972	0.002398	39
BP	GO:0045926	negative regulation of growth	28/972	0.002399	28
BP	GO:0016054	organic acid catabolic process	29/972	0.002406	29
BP	GO:0032355	response to estradiol	18/972	0.002523	18
BP	GO:0019852	L-ascorbic acid metabolic process	5/972	0.002523	5
BP	GO:0032570	response to progesterone	10/972	0.002658	10
BP	GO:2000649	regulation of sodium ion transmembrane transporter activity	11/972	0.002731	11
BP	GO:0006986	response to unfolded protein	23/972	0.002797	23
BP	GO:0008064	regulation of actin polymerization or depolymerization	23/972	0.002797	23
BP	GO:1902305	regulation of sodium ion transmembrane transport	12/972	0.002963	12
BP	GO:0030968	endoplasmic reticulum unfolded protein response	18/972	0.002963	18
BP	GO:0007219	Notch signaling pathway	23/972	0.002963	23
BP	GO:0030832	regulation of actin filament length	23/972	0.002963	23
BP	GO:0032535	regulation of cellular component size	37/972	0.003021	37
BP	GO:0048146	positive regulation of fibroblast proliferation	10/972	0.003042	10
BP	GO:0070849	response to epidermal growth factor	10/972	0.003042	10
BP	GO:0055076	transition metal ion homeostasis	19/972	0.003056	19
BP	GO:0010634	positive regulation of epithelial cell migration	22/972	0.003172	22
BP	GO:0071711	basement membrane organization	8/972	0.003245	8
BP	GO:0006066	alcohol metabolic process	37/972	0.003439	37
BP	GO:0009226	nucleotide-sugar biosynthetic process	7/972	0.003499	7
BP	GO:0071248	cellular response to metal ion	23/972	0.003584	23
BP	GO:0048013	ephrin receptor signaling pathway	14/972	0.003709	14
BP	GO:0001885	endothelial cell development	12/972	0.003769	12
BP	GO:0043254	regulation of protein-containing complex assembly	42/972	0.003809	42
BP	GO:0030838	positive regulation of actin filament polymerization	15/972	0.003826	15
BP	GO:0001666	response to hypoxia	35/972	0.003826	35
BP	GO:0001765	membrane raft assembly	5/972	0.003847	5
BP	GO:0034314	Arp2/3 complex-mediated actin nucleation	9/972	0.003855	9
BP	GO:0046949	fatty-acyl-CoA biosynthetic process	8/972	0.0039	8

BP	GO:0045765	regulation of angiogenesis	34/972	0.0039	34
BP	GO:0048008	platelet-derived growth factor receptor signaling pathway	11/972	0.003932	11
BP	GO:0022898	regulation of transmembrane transporter activity	29/972	0.003935	29
BP	GO:0019885	antigen processing and presentation of endogenous peptide antigen via MHC class I	6/972	0.004036	6
BP	GO:0061687	detoxification of inorganic compound	6/972	0.004036	6
BP	GO:0033866	nucleoside bisphosphate biosynthetic process	12/972	0.004048	12
BP	GO:0034030	ribonucleoside bisphosphate biosynthetic process	12/972	0.004048	12
BP	GO:0034033	purine nucleoside bisphosphate biosynthetic process	12/972	0.004048	12
BP	GO:0006900	vesicle budding from membrane	16/972	0.004053	16
BP	GO:1903793	positive regulation of anion transport	44/972	0.004068	44
BP	GO:0031099	regeneration	23/972	0.004068	23
BP	GO:0046395	carboxylic acid catabolic process	27/972	0.004068	27
BP	GO:0010977	negative regulation of neuron projection development	18/972	0.004116	18
BP	GO:0014706	striated muscle tissue development	35/972	0.004123	35
BP	GO:0032412	regulation of ion transmembrane transporter activity	28/972	0.004258	28
BP	GO:0035966	response to topologically incorrect protein	24/972	0.004445	24
BP	GO:0030833	regulation of actin filament polymerization	21/972	0.004538	21
BP	GO:0046394	carboxylic acid biosynthetic process	33/972	0.00494	33
BP	GO:0030239	myofibril assembly	11/972	0.004953	11
BP	GO:0043491	protein kinase B signaling	29/972	0.004953	29
BP	GO:0045444	fat cell differentiation	25/972	0.005094	25
BP	GO:0046596	regulation of viral entry into host cell	9/972	0.005138	9
BP	GO:0003073	regulation of systemic arterial blood pressure	14/972	0.005174	14
BP	GO:0031579	membrane raft organization	7/972	0.005174	7
BP	GO:1905048	regulation of metallopeptidase activity	7/972	0.005174	7
BP	GO:0097501	stress response to metal ion	6/972	0.005174	6
BP	GO:0014812	muscle cell migration	15/972	0.005174	15
BP	GO:0086004	regulation of cardiac muscle cell contraction	8/972	0.005432	8
BP	GO:0048857	neural nucleus development	11/972	0.005511	11
BP	GO:0001649	osteoblast differentiation	25/972	0.005608	25
BP	GO:0007517	muscle organ development	32/972	0.005706	32
BP	GO:0001503	ossification	38/972	0.005706	38
BP	GO:0035337	fatty-acyl-CoA metabolic process	9/972	0.005888	9
BP	GO:0046688	response to copper ion	9/972	0.005888	9
BP	GO:0022617	extracellular matrix disassembly	13/972	0.005937	13
BP	GO:0034620	cellular response to unfolded protein	19/972	0.005937	19
BP	GO:0050678	regulation of epithelial cell proliferation	36/972	0.005965	36
BP	GO:0036293	response to decreased oxygen levels	35/972	0.005965	35
BP	GO:0001889	liver development	18/972	0.006347	18
BP	GO:0019883	antigen processing and presentation of endogenous antigen	7/972	0.006347	7
BP	GO:0010952	positive regulation of peptidase activity	23/972	0.006456	23
BP	GO:0009991	response to extracellular stimulus	43/972	0.006456	43
BP	GO:0019058	viral life cycle	34/972	0.006456	34
BP	GO:0009152	purine ribonucleotide biosynthetic process	21/972	0.006456	21
BP	GO:0048771	tissue remodeling	21/972	0.006456	21
BP	GO:0002483	antigen processing and presentation of endogenous peptide antigen	6/972	0.006586	6
BP	GO:0098876	vesicle-mediated transport to the plasma membrane	14/972	0.006586	14
BP	GO:0030041	actin filament polymerization	22/972	0.006596	22
BP	GO:0016053	organic acid biosynthetic process	33/972	0.006651	33
BP	GO:0086003	cardiac muscle cell contraction	12/972	0.006671	12
BP	GO:0001558	regulation of cell growth	38/972	0.006693	38
BP	GO:0032233	positive regulation of actin filament bundle assembly	11/972	0.006693	11
BP	GO:0045667	regulation of osteoblast differentiation	17/972	0.006974	17
BP	GO:0006637	acyl-CoA metabolic process	15/972	0.006974	15
BP	GO:0006892	post-Golgi vesicle-mediated transport	15/972	0.006974	15
BP	GO:0035383	thioester metabolic process	15/972	0.006974	15
BP	GO:0008154	actin polymerization or depolymerization	24/972	0.006974	24
BP	GO:0061008	hepaticobiliary system development	18/972	0.006974	18
BP	GO:0010769	regulation of cell morphogenesis involved in differentiation	14/972	0.006974	14
BP	GO:0070167	regulation of biomineral tissue development	14/972	0.006974	14
BP	GO:0006596	polyamine biosynthetic process	5/972	0.007211	5
BP	GO:0006694	steroid biosynthetic process	22/972	0.007211	22
BP	GO:0046890	regulation of lipid biosynthetic process	23/972	0.007287	23
BP	GO:0071241	cellular response to inorganic substance	24/972	0.007287	24
BP	GO:0032409	regulation of transporter activity	29/972	0.007411	29
BP	GO:0090257	regulation of muscle system process	26/972	0.007561	26
BP	GO:0071902	positive regulation of protein serine/threonine kinase activity	31/972	0.007573	31
BP	GO:1901605	alpha-amino acid metabolic process	22/972	0.007573	22
BP	GO:0052548	regulation of endopeptidase activity	39/972	0.008068	39
BP	GO:0048762	mesenchymal cell differentiation	25/972	0.008068	25
BP	GO:2000178	negative regulation of neural precursor cell proliferation	6/972	0.00812	6
BP	GO:0045637	regulation of myeloid cell differentiation	27/972	0.008167	27
BP	GO:0110149	regulation of biomineralization	14/972	0.008252	14
BP	GO:0030224	monocyte differentiation	8/972	0.008395	8
BP	GO:0071709	membrane assembly	8/972	0.008395	8
BP	GO:0035967	cellular response to topologically incorrect protein	20/972	0.008708	20
BP	GO:0071280	cellular response to copper ion	7/972	0.008918	7
BP	GO:0002027	regulation of heart rate	14/972	0.009015	14
BP	GO:0030901	midbrain development	13/972	0.00903	13
BP	GO:0032273	positive regulation of protein polymerization	17/972	0.009126	17
BP	GO:0019882	antigen processing and presentation	25/972	0.009378	25
BP	GO:0072521	purine-containing compound metabolic process	41/972	0.009529	41
BP	GO:0042493	response to drug	34/972	0.009657	34
BP	GO:0086001	cardiac muscle cell action potential	12/972	0.009657	12
BP	GO:0110020	regulation of actomyosin structure organization	14/972	0.009657	14
BP	GO:0010649	regulation of cell communication by electrical coupling	5/972	0.009657	5
BP	GO:0086069	bundle of His cell to Purkinje myocyte communication	5/972	0.009657	5
BP	GO:0002687	positive regulation of leukocyte migration	17/972	0.009657	17
BP	GO:0009225	nucleotide-sugar metabolic process	8/972	0.009657	8

BP	GO:1903115	regulation of actin filament-based movement	8/972	0.009657	8
BP	GO:1902652	secondary alcohol metabolic process	19/972	0.009952	19
BP	GO:0032516	positive regulation of phosphoprotein phosphatase activity	6/972	0.009952	6
BP	GO:0035455	response to interferon-alpha	6/972	0.009952	6
BP	GO:0150105	protein localization to cell-cell junction	6/972	0.009952	6
CC	GO:0005925	focal adhesion	93/1005	1.21E-31	93
CC	GO:0030055	cell-substrate junction	93/1005	2.49E-31	93
CC	GO:0005911	cell-cell junction	76/1005	4.57E-16	76
CC	GO:0032432	actin filament bundle	27/1005	1.28E-14	27
CC	GO:0043292	contractile fiber	45/1005	9.16E-13	45
CC	GO:0005912	adherens junction	38/1005	1.43E-12	38
CC	GO:0031252	cell leading edge	62/1005	1.73E-12	62
CC	GO:0062023	collagen-containing extracellular matrix	63/1005	1.73E-12	63
CC	GO:0001725	stress fiber	23/1005	2.03E-12	23
CC	GO:0097517	contractile actin filament bundle	23/1005	2.03E-12	23
CC	GO:0030016	myofibril	42/1005	1.29E-11	42
CC	GO:0042641	actomyosin	23/1005	5.68E-11	23
CC	GO:0005788	endoplasmic reticulum lumen	49/1005	5.68E-11	49
CC	GO:0030017	sarcomere	36/1005	2.73E-09	36
CC	GO:0030018	Z disc	27/1005	3.56E-09	27
CC	GO:0031674	I band	28/1005	6.29E-09	28
CC	GO:0001726	ruffle	32/1005	1.15E-08	32
CC	GO:0030667	secretory granule membrane	42/1005	1.89E-07	42
CC	GO:0030133	transport vesicle	50/1005	2.14E-07	50
CC	GO:0042470	melanosome	22/1005	4.86E-07	22
CC	GO:0048770	pigment granule	22/1005	4.86E-07	22
CC	GO:0005884	actin filament	22/1005	1.57E-06	22
CC	GO:0030027	lamellipodium	30/1005	2.09E-06	30
CC	GO:0005938	cell cortex	39/1005	2.61E-06	39
CC	GO:0034774	secretory granule lumen	40/1005	5.39E-06	40
CC	GO:0045177	apical part of cell	47/1005	6.92E-06	47
CC	GO:0060205	cytoplasmic vesicle lumen	40/1005	6.92E-06	40
CC	GO:0031983	vesicle lumen	40/1005	7.84E-06	40
CC	GO:0030662	coated vesicle membrane	27/1005	1.38E-05	27
CC	GO:0016324	apical plasma membrane	41/1005	1.60E-05	41
CC	GO:0031256	leading edge membrane	26/1005	1.60E-05	26
CC	GO:0005765	lysosomal membrane	43/1005	1.67E-05	43
CC	GO:0098852	lytic vacuole membrane	43/1005	1.67E-05	43
CC	GO:0030135	coated vesicle	36/1005	2.41E-05	36
CC	GO:0030670	phagocytic vesicle membrane	16/1005	2.49E-05	16
CC	GO:0045335	phagocytic vesicle	22/1005	2.70E-05	22
CC	GO:0030864	cortical actin cytoskeleton	16/1005	2.83E-05	16
CC	GO:0005774	vacuolar membrane	46/1005	3.62E-05	46
CC	GO:0030175	filopodium	18/1005	7.05E-05	18
CC	GO:0098858	actin-based cell projection	28/1005	9.39E-05	28
CC	GO:0002102	podosome	9/1005	9.72E-05	9
CC	GO:0070820	tertiary granule	23/1005	0.000161	23
CC	GO:0012507	ER to Golgi transport vesicle membrane	13/1005	0.000174	13
CC	GO:0043034	costamere	7/1005	0.000235	7
CC	GO:0030863	cortical cytoskeleton	17/1005	0.000268	17
CC	GO:0030658	transport vesicle membrane	26/1005	0.000268	26
CC	GO:0005604	basement membrane	16/1005	0.000268	16
CC	GO:0030134	COPII-coated ER to Golgi transport vesicle	16/1005	0.000268	16
CC	GO:0014704	intercalated disc	11/1005	0.00029	11
CC	GO:0140534	endoplasmic reticulum protein-containing complex	19/1005	0.000306	19
CC	GO:0101002	ficolin-1-rich granule	24/1005	0.000325	24
CC	GO:0031093	platelet alpha granule lumen	13/1005	0.000345	13
CC	GO:0055038	recycling endosome membrane	15/1005	0.000372	15
CC	GO:0005770	late endosome	31/1005	0.000387	31
CC	GO:0030139	endocytic vesicle	33/1005	0.000562	33
CC	GO:0042383	sarcolemma	19/1005	0.00057	19
CC	GO:0016327	apicolateral plasma membrane	7/1005	0.00057	7
CC	GO:0042827	platelet dense granule	7/1005	0.00057	7
CC	GO:0031253	cell projection membrane	35/1005	0.000639	35
CC	GO:0071556	integral component of lumenal side of endoplasmic reticulum membrane	8/1005	0.000733	8
CC	GO:0098553	lumenal side of endoplasmic reticulum membrane	8/1005	0.000733	8
CC	GO:0032587	ruffle membrane	15/1005	0.000898	15
CC	GO:0030666	endocytic vesicle membrane	21/1005	0.000898	21
CC	GO:0045121	membrane raft	33/1005	0.001266	33
CC	GO:0098857	membrane microdomain	33/1005	0.001266	33
CC	GO:0019897	extrinsic component of plasma membrane	21/1005	0.001437	21
CC	GO:0044291	cell-cell contact zone	12/1005	0.001614	12
CC	GO:0042581	specific granule	20/1005	0.001824	20
CC	GO:0043296	apical junction complex	18/1005	0.001963	18
CC	GO:0005798	Golgi-associated vesicle	14/1005	0.002027	14
CC	GO:0005771	multivesicular body	11/1005	0.0023	11
CC	GO:0016328	lateral plasma membrane	11/1005	0.0023	11
CC	GO:0005793	endoplasmic reticulum-Golgi intermediate compartment	17/1005	0.002893	17
CC	GO:0098576	lumenal side of membrane	8/1005	0.003093	8
CC	GO:0005581	collagen trimer	13/1005	0.003732	13
CC	GO:0098644	complex of collagen trimers	6/1005	0.003843	6
CC	GO:0031258	lamellipodium membrane	6/1005	0.004991	6
CC	GO:0035577	azurophil granule membrane	10/1005	0.005026	10
CC	GO:0005902	microvillus	13/1005	0.005425	13
CC	GO:0031091	platelet alpha granule	13/1005	0.005425	13
CC	GO:0031902	late endosome membrane	17/1005	0.006166	17
CC	GO:0009898	cytoplasmic side of plasma membrane	19/1005	0.006932	19
CC	GO:0030140	trans-Golgi network transport vesicle	7/1005	0.006932	7
CC	GO:0031941	filamentous actin	7/1005	0.006932	7

CC	GO:0019898	extrinsic component of membrane	29/1005	0.007192	29
CC	GO:0070821	tertiary granule membrane	11/1005	0.008056	11
CC	GO:0030176	integral component of endoplasmic reticulum membrane	18/1005	0.008058	18
CC	GO:0042611	MHC protein complex	6/1005	0.00908	6
MF	GO:0003779	actin binding	75/969	8.39E-17	75
MF	GO:0098631	cell adhesion mediator activity	20/969	2.74E-09	20
MF	GO:0051015	actin filament binding	38/969	3.65E-09	38
MF	GO:0098632	cell-cell adhesion mediator activity	18/969	3.65E-09	18
MF	GO:0005518	collagen binding	21/969	3.65E-09	21
MF	GO:0005178	integrin binding	28/969	1.80E-07	28
MF	GO:0008307	structural constituent of muscle	15/969	1.95E-07	15
MF	GO:0098641	cadherin binding involved in cell-cell adhesion	10/969	5.09E-07	10
MF	GO:0045296	cadherin binding	44/969	1.68E-06	44
MF	GO:0019838	growth factor binding	25/969	4.17E-06	25
MF	GO:0005201	extracellular matrix structural constituent	27/969	2.17E-05	27
MF	GO:0048407	platelet-derived growth factor binding	7/969	2.17E-05	7
MF	GO:0017022	myosin binding	16/969	4.37E-05	16
MF	GO:0005200	structural constituent of cytoskeleton	19/969	9.98E-05	19
MF	GO:0030674	protein-macromolecule adaptor activity	33/969	0.000205	33
MF	GO:0051371	muscle alpha-actinin binding	7/969	0.000263	7
MF	GO:0030020	extracellular matrix structural constituent conferring tensile strength	11/969	0.000308	11
MF	GO:0072341	modified amino acid binding	17/969	0.000308	17
MF	GO:0004857	enzyme inhibitor activity	41/969	0.000717	41
MF	GO:0005525	GTP binding	39/969	0.001841	39
MF	GO:0051020	GTPase binding	27/969	0.001841	27
MF	GO:0001882	nucleoside binding	40/969	0.001841	40
MF	GO:0032550	purine ribonucleoside binding	39/969	0.002275	39
MF	GO:0001883	purine nucleoside binding	39/969	0.00235	39
MF	GO:0032549	ribonucleoside binding	39/969	0.00235	39
MF	GO:0019001	guanyl nucleotide binding	40/969	0.00235	40
MF	GO:0032561	guanyl ribonucleotide binding	40/969	0.00235	40
MF	GO:0042805	actinin binding	9/969	0.002413	9
MF	GO:0003924	GTPase activity	34/969	0.002869	34
MF	GO:0031489	myosin V binding	6/969	0.003059	6
MF	GO:0050840	extracellular matrix binding	11/969	0.003248	11
MF	GO:0019003	GDP binding	13/969	0.003584	13
MF	GO:0017166	vinculin binding	5/969	0.003584	5
MF	GO:0086080	protein binding involved in heterotypic cell-cell adhesion	5/969	0.003584	5
MF	GO:0002020	protease binding	18/969	0.004436	18
MF	GO:0035259	glucocorticoid receptor binding	5/969	0.00555	5
MF	GO:0019207	kinase regulator activity	25/969	0.005961	25
MF	GO:0001786	phosphatidylserine binding	11/969	0.005961	11
MF	GO:0060090	molecular adaptor activity	34/969	0.006712	34
MF	GO:0005516	calmodulin binding	23/969	0.006968	23
MF	GO:0015037	peptide disulfide oxidoreductase activity	5/969	0.007575	5
MF	GO:0051393	alpha-actinin binding	7/969	0.00792	7
MF	GO:0044325	ion channel binding	17/969	0.008107	17
MF	GO:0019887	protein kinase regulator activity	22/969	0.00884	22
MF	GO:0043394	proteoglycan binding	8/969	0.008935	8
