# Supplementary material for: Integrative Analysis of Bulk RNA-Seq and Single-Cell RNA-Seq Unveils the Characteristics of the Immune Microenvironment and Prognosis Signature in Prostate Cancer
Source: J Oncol. 2022 Jul 19;2022:6768139. doi: 10.1155/2022/6768139 (PMC9325591; doi:10.1155/2022/6768139)
Supplement: Supplementary Materials — Figure S1. Workflow of the analysis. Figure S2. Validation of the risk score model using the GSE54460 dataset. A. Patients with prostate cancer (PRAD) in the GSE54460 cohort are listed in ascending order of risk score. B. Progression-free interval (PFI) distribution versus the risk score of each patient in the GSE54460 cohort. C. Kaplan–Meier (KM) curves of patients with different risk levels in the GSE54460 validation set. D. Receiver Operating Characteristic (ROC) curve analysis for 1-, 3- and 5-year PFI using the clinical information of patients of the GSE54460 validation dataset. Figure S3. Validation of the risk score model using the GSE46602 dataset. A. Patients with prostate cancer (PRAD) in the GSE46602 cohort are listed in ascending order of risk score. B. Progression-free interval (PFI) distribution versus the risk score of each patient in the GSE46602 cohort. C. Kaplan–Meier (KM) curves of patients with different risk levels in the GSE46602 validation dataset. D. Receiver Operating Characteristic (ROC) curve analysis for 1-, 3- and 5-year PFI using the clinical information of patients of the GSE46602 validation dataset. Figure S4. Validation of the risk score model using the GSE70768 dataset. A. Patients with prostate cancer (PRAD) in the GSE70768 cohort are listed in ascending order of risk score. B. Progression-free interval (PFI) distribution versus the risk score of each patient in the GSE70768 cohort. C. Kaplan–Meier (KM) curves of patients with different risk levels in the GSE70768 validation dataset. D. Receiver Operating Characteristic (ROC) curve analysis for 1-, 3- and 5-year PFI using the clinical information of patients of the GSE70768 validation dataset. Figure S5. Validation of the risk score model using the GSE70769 dataset. A. Patients with prostate cancer (PRAD) in the GSE70769 validation dataset are listed in ascending order of risk score. B. Progression-free interval (PFI) distribution versus the risk score of each patient in the GSE707 [file 6768139.f1.zip › 6768139.f1/Table S13.pdf]

ONTOLOGY	ID	Description	GeneRatio	p.adjust	Count
BP	GO:0006614	SRP-dependent cotranslational protein targeting to membrane	53/1520	1.31E-26	53
BP	GO:0006613	cotranslational protein targeting to membrane	53/1520	9.93E-26	53
BP	GO:0001667	ameboidal-type cell migration	117/1520	9.93E-26	117
BP	GO:0000184	nuclear-transcribed mRNA catabolic process, nonsense-mediated decay	55/1520	1.45E-25	55
BP	GO:0045047	protein targeting to ER	54/1520	1.14E-24	54
BP	GO:0031589	cell-substrate adhesion	97/1520	2.22E-24	97
BP	GO:0030198	extracellular matrix organization	102/1520	2.83E-24	102
BP	GO:0043062	extracellular structure organization	102/1520	3.09E-24	102
BP	GO:0003158	endothelium development	57/1520	3.20E-24	57
BP	GO:0045229	external encapsulating structure organization	102/1520	3.84E-24	102
BP	GO:0072599	establishment of protein localization to endoplasmic reticulum	54/1520	3.85E-24	54
BP	GO:0010810	regulation of cell-substrate adhesion	71/1520	6.37E-23	71
BP	GO:0070972	protein localization to endoplasmic reticulum	58/1520	1.68E-22	58
BP	GO:0045446	endothelial cell differentiation	50/1520	1.73E-21	50
BP	GO:0090130	tissue migration	92/1520	4.76E-21	92
BP	GO:0010631	epithelial cell migration	90/1520	1.36E-20	90
BP	GO:0090132	epithelium migration	90/1520	2.40E-20	90
BP	GO:0019083	viral transcription	59/1520	3.83E-19	59
BP	GO:0006413	translational initiation	61/1520	6.19E-19	61
BP	GO:0006612	protein targeting to membrane	64/1520	7.08E-19	64
BP	GO:0002064	epithelial cell development	62/1520	5.87E-18	62
BP	GO:0043542	endothelial cell migration	73/1520	1.13E-17	73
BP	GO:0090150	establishment of protein localization to membrane	84/1520	1.81E-17	84
BP	GO:0040013	negative regulation of locomotion	87/1520	2.58E-17	87
BP	GO:0019080	viral gene expression	59/1520	5.45E-17	59
BP	GO:0030336	negative regulation of cell migration	79/1520	1.18E-16	79
BP	GO:2000146	negative regulation of cell motility	81/1520	1.36E-16	81
BP	GO:0051271	negative regulation of cellular component movement	82/1520	1.36E-16	82
BP	GO:0000956	nuclear-transcribed mRNA catabolic process	60/1520	1.36E-16	60
BP	GO:0010811	positive regulation of cell-substrate adhesion	44/1520	3.60E-16	44
BP	GO:0010632	regulation of epithelial cell migration	71/1520	3.08E-15	71
BP	GO:0001885	endothelial cell development	31/1520	6.58E-15	31
BP	GO:0032970	regulation of actin filament-based process	83/1520	2.01E-14	83
BP	GO:0022604	regulation of cell morphogenesis	71/1520	2.78E-14	71
BP	GO:0001570	vasculogenesis	33/1520	2.88E-14	33
BP	GO:0045785	positive regulation of cell adhesion	87/1520	4.77E-14	87
BP	GO:0050673	epithelial cell proliferation	86/1520	2.20E-13	86
BP	GO:0006605	protein targeting	87/1520	4.56E-13	87
BP	GO:0032956	regulation of actin cytoskeleton organization	75/1520	5.63E-13	75
BP	GO:0060485	mesenchyme development	65/1520	1.85E-12	65
BP	GO:0061028	establishment of endothelial barrier	24/1520	3.19E-12	24
BP	GO:0010594	regulation of endothelial cell migration	56/1520	5.82E-12	56
BP	GO:0050678	regulation of epithelial cell proliferation	75/1520	1.43E-11	75
BP	GO:0007160	cell-matrix adhesion	55/1520	1.73E-11	55
BP	GO:0043087	regulation of GTPase activity	89/1520	1.99E-11	89
BP	GO:0007162	negative regulation of cell adhesion	64/1520	2.13E-11	64
BP	GO:1901342	regulation of vasculature development	69/1520	8.20E-11	69
BP	GO:0007015	actin filament organization	81/1520	8.55E-11	81
BP	GO:0072659	protein localization to plasma membrane	60/1520	8.88E-11	60
BP	GO:0007044	cell-substrate junction assembly	33/1520	9.45E-11	33
BP	GO:0045765	regulation of angiogenesis	68/1520	9.52E-11	68
BP	GO:0051893	regulation of focal adhesion assembly	26/1520	9.52E-11	26
BP	GO:0090109	regulation of cell-substrate junction assembly	26/1520	9.52E-11	26
BP	GO:0150115	cell-substrate junction organization	34/1520	9.83E-11	34
BP	GO:0150116	regulation of cell-substrate junction organization	27/1520	9.83E-11	27
BP	GO:0051017	actin filament bundle assembly	42/1520	1.02E-10	42
BP	GO:0001952	regulation of cell-matrix adhesion	37/1520	1.50E-10	37
BP	GO:0030856	regulation of epithelial cell differentiation	42/1520	1.57E-10	42
BP	GO:0048762	mesenchymal cell differentiation	53/1520	2.05E-10	53
BP	GO:0051056	regulation of small GTPase mediated signal transduction	65/1520	2.17E-10	65
BP	GO:0061572	actin filament bundle organization	42/1520	2.37E-10	42
BP	GO:0001655	urogenital system development	65/1520	2.43E-10	65
BP	GO:0032835	glomerulus development	25/1520	2.69E-10	25
BP	GO:0007265	Ras protein signal transduction	67/1520	3.49E-10	67
BP	GO:0002181	cytoplasmic translation	32/1520	7.52E-10	32
BP	GO:0034329	cell junction assembly	77/1520	9.53E-10	77
BP	GO:0002576	platelet degranulation	36/1520	1.33E-09	36
BP	GO:0043534	blood vessel endothelial cell migration	43/1520	1.97E-09	43
BP	GO:0006402	mRNA catabolic process	70/1520	1.97E-09	70
BP	GO:0071900	regulation of protein serine/threonine kinase activity	84/1520	2.54E-09	84
BP	GO:1902903	regulation of supramolecular fiber organization	69/1520	2.78E-09	69
BP	GO:0008360	regulation of cell shape	39/1520	3.14E-09	39
BP	GO:0048041	focal adhesion assembly	28/1520	3.59E-09	28
BP	GO:0034446	substrate adhesion-dependent cell spreading	32/1520	3.59E-09	32
BP	GO:0072001	renal system development	58/1520	4.34E-09	58
BP	GO:0110053	regulation of actin filament organization	56/1520	4.36E-09	56
BP	GO:0003018	vascular process in circulatory system	52/1520	5.63E-09	52
BP	GO:1901888	regulation of cell junction assembly	45/1520	5.79E-09	45
BP	GO:0090066	regulation of anatomical structure size	83/1520	6.12E-09	83
BP	GO:0010634	positive regulation of epithelial cell migration	42/1520	6.45E-09	42
BP	GO:0003007	heart morphogenesis	51/1520	6.63E-09	51
BP	GO:0043547	positive regulation of GTPase activity	73/1520	6.86E-09	73
BP	GO:0060562	epithelial tube morphogenesis	60/1520	8.62E-09	60
BP	GO:0001822	kidney development	56/1520	1.10E-08	56
BP	GO:0048008	platelet-derived growth factor receptor signaling pathway	22/1520	1.11E-08	22
BP	GO:1990778	protein localization to cell periphery	62/1520	1.47E-08	62
BP	GO:1903034	regulation of response to wounding	39/1520	3.69E-08	39
BP	GO:0072006	nephron development	35/1520	4.35E-08	35
BP	GO:0061138	morphogenesis of a branching epithelium	40/1520	5.32E-08	40
BP	GO:0060840	artery development	28/1520	5.43E-08	28
BP	GO:0045216	cell-cell junction organization	45/1520	5.44E-08	45
BP	GO:0006401	RNA catabolic process	71/1520	5.44E-08	71
BP	GO:0044409	entry into host	37/1520	5.75E-08	37
BP	GO:0010812	negative regulation of cell-substrate adhesion	23/1520	5.96E-08	23
BP	GO:0060284	regulation of cell development	79/1520	7.18E-08	79

BP	GO:0070371	ERK1 and ERK2 cascade	59/1520	8.61E-08	59
BP	GO:0016049	cell growth	77/1520	8.75E-08	77
BP	GO:0003012	muscle system process	75/1520	8.78E-08	75
BP	GO:0001945	lymph vessel development	14/1520	1.01E-07	14
BP	GO:0022612	gland morphogenesis	30/1520	1.24E-07	30
BP	GO:0001763	morphogenesis of a branching structure	41/1520	1.32E-07	41
BP	GO:0007411	axon guidance	53/1520	1.45E-07	53
BP	GO:0048844	artery morphogenesis	23/1520	1.45E-07	23
BP	GO:0001954	positive regulation of cell-matrix adhesion	20/1520	1.45E-07	20
BP	GO:0050920	regulation of chemotaxis	46/1520	1.55E-07	46
BP	GO:0007229	integrin-mediated signaling pathway	29/1520	1.57E-07	29
BP	GO:0097485	neuron projection guidance	53/1520	1.57E-07	53
BP	GO:0010595	positive regulation of endothelial cell migration	33/1520	1.61E-07	33
BP	GO:0001837	epithelial to mesenchymal transition	36/1520	1.82E-07	36
BP	GO:0018108	peptidyl-tyrosine phosphorylation	64/1520	1.96E-07	64
BP	GO:0071559	response to transforming growth factor beta	50/1520	2.09E-07	50
BP	GO:0052126	movement in host environment	39/1520	2.10E-07	39
BP	GO:0046578	regulation of Ras protein signal transduction	41/1520	2.29E-07	41
BP	GO:0007266	Rho protein signal transduction	33/1520	2.29E-07	33
BP	GO:0018212	peptidyl-tyrosine modification	64/1520	2.60E-07	64
BP	GO:0001935	endothelial cell proliferation	41/1520	2.63E-07	41
BP	GO:0002040	sprouting angiogenesis	40/1520	2.73E-07	40
BP	GO:0070849	response to epidermal growth factor	18/1520	2.85E-07	18
BP	GO:0003151	outflow tract morphogenesis	23/1520	3.14E-07	23
BP	GO:0070372	regulation of ERK1 and ERK2 cascade	55/1520	3.44E-07	55
BP	GO:0071364	cellular response to epidermal growth factor stimulus	17/1520	4.00E-07	17
BP	GO:0030857	negative regulation of epithelial cell differentiation	18/1520	4.13E-07	18
BP	GO:0006936	muscle contraction	61/1520	4.19E-07	61
BP	GO:0061041	regulation of wound healing	32/1520	4.45E-07	32
BP	GO:0048568	embryonic organ development	68/1520	4.57E-07	68
BP	GO:0042692	muscle cell differentiation	62/1520	4.90E-07	62
BP	GO:0048771	tissue remodeling	38/1520	6.21E-07	38
BP	GO:0010717	regulation of epithelial to mesenchymal transition	27/1520	6.22E-07	27
BP	GO:0071560	cellular response to transforming growth factor beta stimulus	48/1520	6.50E-07	48
BP	GO:0043405	regulation of MAP kinase activity	55/1520	6.50E-07	55
BP	GO:0003206	cardiac chamber morphogenesis	29/1520	9.42E-07	29
BP	GO:0061437	renal system vasculature development	13/1520	9.42E-07	13
BP	GO:0061440	kidney vasculature development	13/1520	9.42E-07	13
BP	GO:0032231	regulation of actin filament bundle assembly	27/1520	9.42E-07	27
BP	GO:0071902	positive regulation of protein serine/threonine kinase activity	55/1520	9.95E-07	55
BP	GO:0048010	vascular endothelial growth factor receptor signaling pathway	26/1520	1.01E-06	26
BP	GO:0035924	cellular response to vascular endothelial growth factor stimulus	22/1520	1.09E-06	22
BP	GO:1904019	epithelial cell apoptotic process	29/1520	1.12E-06	29
BP	GO:0048608	reproductive structure development	66/1520	1.12E-06	66
BP	GO:0007599	hemostasis	59/1520	1.17E-06	59
BP	GO:1902905	positive regulation of supramolecular fiber organization	41/1520	1.36E-06	41
BP	GO:0061458	reproductive system development	66/1520	1.47E-06	66
BP	GO:0002483	antigen processing and presentation of endogenous peptide antigen	11/1520	1.48E-06	11
BP	GO:1901653	cellular response to peptide	64/1520	1.48E-06	64
BP	GO:0010718	positive regulation of epithelial to mesenchymal transition	18/1520	1.55E-06	18
BP	GO:0048660	regulation of smooth muscle cell proliferation	35/1520	1.63E-06	35
BP	GO:0050680	negative regulation of epithelial cell proliferation	35/1520	1.63E-06	35
BP	GO:0007596	blood coagulation	58/1520	1.77E-06	58
BP	GO:1900024	regulation of substrate adhesion-dependent cell spreading	19/1520	1.85E-06	19
BP	GO:0046718	viral entry into host cell	32/1520	2.02E-06	32
BP	GO:0048659	smooth muscle cell proliferation	35/1520	2.20E-06	35
BP	GO:0001936	regulation of endothelial cell proliferation	37/1520	2.30E-06	37
BP	GO:0051348	negative regulation of transferase activity	52/1520	2.64E-06	52
BP	GO:0001890	placenta development	31/1520	2.83E-06	31
BP	GO:0050817	coagulation	58/1520	2.85E-06	58
BP	GO:0043491	protein kinase B signaling	49/1520	3.09E-06	49
BP	GO:0007009	plasma membrane organization	28/1520	3.13E-06	28
BP	GO:0035909	aorta morphogenesis	14/1520	3.14E-06	14
BP	GO:0048754	branching morphogenesis of an epithelial tube	32/1520	3.23E-06	32
BP	GO:0016358	dendrite development	44/1520	3.45E-06	44
BP	GO:0003205	cardiac chamber development	33/1520	3.67E-06	33
BP	GO:0006469	negative regulation of protein kinase activity	45/1520	3.90E-06	45
BP	GO:0032102	negative regulation of response to external stimulus	63/1520	4.03E-06	63
BP	GO:0001946	lymphangiogenesis	10/1520	4.48E-06	10
BP	GO:0072012	glomerulus vasculature development	12/1520	4.79E-06	12
BP	GO:0032535	regulation of cellular component size	60/1520	4.79E-06	60
BP	GO:0036303	lymph vessel morphogenesis	11/1520	5.07E-06	11
BP	GO:0035904	aorta development	18/1520	5.07E-06	18
BP	GO:0007409	axonogenesis	70/1520	5.65E-06	70
BP	GO:0045601	regulation of endothelial cell differentiation	16/1520	6.69E-06	16
BP	GO:2001233	regulation of apoptotic signaling pathway	57/1520	6.74E-06	57
BP	GO:0032233	positive regulation of actin filament bundle assembly	19/1520	7.28E-06	19
BP	GO:0019883	antigen processing and presentation of endogenous antigen	12/1520	7.87E-06	12
BP	GO:0010633	negative regulation of epithelial cell migration	27/1520	8.75E-06	27
BP	GO:0033673	negative regulation of kinase activity	47/1520	9.12E-06	47
BP	GO:0003382	epithelial cell morphogenesis	13/1520	9.66E-06	13
BP	GO:1900026	positive regulation of substrate adhesion-dependent cell spreading	15/1520	1.00E-05	15
BP	GO:0001558	regulation of cell growth	63/1520	1.10E-05	63
BP	GO:0060537	muscle tissue development	59/1520	1.11E-05	59
BP	GO:0034341	response to interferon-gamma	38/1520	1.15E-05	38
BP	GO:0002685	regulation of leukocyte migration	39/1520	1.17E-05	39
BP	GO:0051145	smooth muscle cell differentiation	20/1520	1.19E-05	20
BP	GO:1902305	regulation of sodium ion transmembrane transport	19/1520	1.19E-05	19
BP	GO:0007173	epidermal growth factor receptor signaling pathway	28/1520	1.20E-05	28
BP	GO:0038127	ERBB signaling pathway	31/1520	1.29E-05	31
BP	GO:0033002	muscle cell proliferation	41/1520	1.32E-05	41
BP	GO:0032486	Rap protein signal transduction	9/1520	1.37E-05	9
BP	GO:0072073	kidney epithelium development	29/1520	1.38E-05	29
BP	GO:0003179	heart valve morphogenesis	17/1520	1.38E-05	17
BP	GO:0031032	actomyosin structure organization	37/1520	1.39E-05	37
BP	GO:0050818	regulation of coagulation	20/1520	1.46E-05	20

BP	GO:0016525	negative regulation of angiogenesis	31/1520	1.46E-05	31
BP	GO:0010975	regulation of neuron projection development	65/1520	1.46E-05	65
BP	GO:0050921	positive regulation of chemotaxis	30/1520	1.52E-05	30
BP	GO:0035023	regulation of Rho protein signal transduction	22/1520	1.56E-05	22
BP	GO:0014065	phosphatidylinositol 3-kinase signaling	32/1520	1.57E-05	32
BP	GO:0052372	modulation by symbiont of entry into host	16/1520	1.60E-05	16
BP	GO:0008217	regulation of blood pressure	35/1520	1.63E-05	35
BP	GO:0030193	regulation of blood coagulation	19/1520	1.86E-05	19
BP	GO:2000181	negative regulation of blood vessel morphogenesis	31/1520	1.91E-05	31
BP	GO:0007219	Notch signaling pathway	36/1520	1.91E-05	36
BP	GO:0001701	in utero embryonic development	54/1520	1.99E-05	54
BP	GO:0001892	embryonic placenta development	21/1520	2.07E-05	21
BP	GO:1901343	negative regulation of vasculature development	31/1520	2.19E-05	31
BP	GO:0051701	biological process involved in interaction with host	40/1520	2.23E-05	40
BP	GO:0042326	negative regulation of phosphorylation	62/1520	2.25E-05	62
BP	GO:1900046	regulation of hemostasis	19/1520	2.31E-05	19
BP	GO:0003170	heart valve development	18/1520	2.34E-05	18
BP	GO:0043434	response to peptide hormone	65/1520	2.42E-05	65
BP	GO:0043393	regulation of protein binding	37/1520	2.42E-05	37
BP	GO:0048017	inositol lipid-mediated signaling	37/1520	2.42E-05	37
BP	GO:0043535	regulation of blood vessel endothelial cell migration	31/1520	2.46E-05	31
BP	GO:2001212	regulation of vasculogenesis	9/1520	2.55E-05	9
BP	GO:0045926	negative regulation of growth	43/1520	2.57E-05	43
BP	GO:0048545	response to steroid hormone	53/1520	2.65E-05	53
BP	GO:0050730	regulation of peptidyl-tyrosine phosphorylation	45/1520	2.66E-05	45
BP	GO:0043001	Golgi to plasma membrane protein transport	14/1520	2.66E-05	14
BP	GO:0022407	regulation of cell-cell adhesion	65/1520	2.73E-05	65
BP	GO:0010769	regulation of cell morphogenesis involved in differentiation	23/1520	2.84E-05	23
BP	GO:1904035	regulation of epithelial cell apoptotic process	23/1520	2.84E-05	23
BP	GO:0014812	muscle cell migration	24/1520	2.94E-05	24
BP	GO:0045664	regulation of neuron differentiation	36/1520	2.94E-05	36
BP	GO:0014910	regulation of smooth muscle cell migration	21/1520	2.95E-05	21
BP	GO:0060326	cell chemotaxis	50/1520	3.06E-05	50
BP	GO:0070482	response to oxygen levels	59/1520	3.10E-05	59
BP	GO:0045766	positive regulation of angiogenesis	34/1520	3.10E-05	34
BP	GO:1904018	positive regulation of vasculature development	34/1520	3.10E-05	34
BP	GO:0001974	blood vessel remodeling	15/1520	3.10E-05	15
BP	GO:0085029	extracellular matrix assembly	15/1520	3.10E-05	15
BP	GO:0014909	smooth muscle cell migration	22/1520	3.13E-05	22
BP	GO:0060337	type I interferon signaling pathway	23/1520	3.30E-05	23
BP	GO:0051894	positive regulation of focal adhesion assembly	11/1520	3.41E-05	11
BP	GO:0031099	regeneration	36/1520	3.61E-05	36
BP	GO:0048015	phosphatidylinositol-mediated signaling	36/1520	3.61E-05	36
BP	GO:0060841	venous blood vessel development	8/1520	3.78E-05	8
BP	GO:0071711	basement membrane organization	12/1520	3.79E-05	12
BP	GO:0150117	positive regulation of cell-substrate junction organization	12/1520	3.79E-05	12
BP	GO:0071357	cellular response to type I interferon	23/1520	3.89E-05	23
BP	GO:0060560	developmental growth involved in morphogenesis	40/1520	3.89E-05	40
BP	GO:1901654	response to ketone	36/1520	3.99E-05	36
BP	GO:0001933	negative regulation of protein phosphorylation	56/1520	3.99E-05	56
BP	GO:0032092	positive regulation of protein binding	21/1520	4.18E-05	21
BP	GO:0019885	antigen processing and presentation of endogenous peptide antigen via MHC class I	9/1520	4.37E-05	9
BP	GO:1901550	regulation of endothelial cell development	9/1520	4.37E-05	9
BP	GO:1903140	regulation of establishment of endothelial barrier	9/1520	4.37E-05	9
BP	GO:0003014	renal system process	26/1520	4.47E-05	26
BP	GO:0061448	connective tissue development	42/1520	4.47E-05	42
BP	GO:1905314	semi-lunar valve development	14/1520	4.66E-05	14
BP	GO:0097084	vascular associated smooth muscle cell development	7/1520	4.82E-05	7
BP	GO:0003176	aortic valve development	13/1520	5.20E-05	13
BP	GO:1903053	regulation of extracellular matrix organization	15/1520	5.28E-05	15
BP	GO:0001666	response to hypoxia	54/1520	5.29E-05	54
BP	GO:0051098	regulation of binding	55/1520	5.33E-05	55
BP	GO:0003180	aortic valve morphogenesis	12/1520	5.35E-05	12
BP	GO:0031345	negative regulation of cell projection organization	34/1520	5.40E-05	34
BP	GO:1903036	positive regulation of response to wounding	19/1520	6.17E-05	19
BP	GO:0048732	gland development	61/1520	6.22E-05	61
BP	GO:0003197	endocardial cushion development	14/1520	6.22E-05	14
BP	GO:0072009	nephron epithelium development	24/1520	6.29E-05	24
BP	GO:0043406	positive regulation of MAP kinase activity	40/1520	6.31E-05	40
BP	GO:0036293	response to decreased oxygen levels	55/1520	6.70E-05	55
BP	GO:0035850	epithelial cell differentiation involved in kidney development	15/1520	6.79E-05	15
BP	GO:0038084	vascular endothelial growth factor signaling pathway	15/1520	6.79E-05	15
BP	GO:0034101	erythrocyte homeostasis	26/1520	6.85E-05	26
BP	GO:2000649	regulation of sodium ion transmembrane transporter activity	16/1520	6.98E-05	16
BP	GO:0003231	cardiac ventricle development	25/1520	7.10E-05	25
BP	GO:0051895	negative regulation of focal adhesion assembly	9/1520	7.39E-05	9
BP	GO:0061298	retina vasculature development in camera-type eye	9/1520	7.39E-05	9
BP	GO:0150118	negative regulation of cell-substrate junction organization	9/1520	7.39E-05	9
BP	GO:1901889	negative regulation of cell junction assembly	12/1520	7.47E-05	12
BP	GO:0002028	regulation of sodium ion transport	21/1520	8.44E-05	21
BP	GO:0048638	regulation of developmental growth	50/1520	8.58E-05	50
BP	GO:0070167	regulation of biomineral tissue development	22/1520	8.58E-05	22
BP	GO:0034340	response to type I interferon	23/1520	8.58E-05	23
BP	GO:0052547	regulation of peptidase activity	65/1520	8.74E-05	65
BP	GO:0061045	negative regulation of wound healing	19/1520	8.92E-05	19
BP	GO:0030099	myeloid cell differentiation	61/1520	9.27E-05	61
BP	GO:0001503	ossification	59/1520	9.44E-05	59
BP	GO:0070374	positive regulation of ERK1 and ERK2 cascade	37/1520	0.000100323	37
BP	GO:0051099	positive regulation of binding	32/1520	0.000103889	32
BP	GO:0035633	maintenance of blood-brain barrier	12/1520	0.000104487	12
BP	GO:0060135	maternal process involved in female pregnancy	16/1520	0.000110942	16
BP	GO:0008347	glial cell migration	15/1520	0.000111066	15
BP	GO:0090287	regulation of cellular response to growth factor stimulus	47/1520	0.000111066	47
BP	GO:0050679	positive regulation of epithelial cell proliferation	36/1520	0.000112696	36
BP	GO:1903793	positive regulation of anion transport	67/1520	0.000115327	67
BP	GO:0031532	actin cytoskeleton reorganization	23/1520	0.000116365	23

BP	GO:0048661	positive regulation of smooth muscle cell proliferation	22/1520	0.000117211	22
BP	GO:0110149	regulation of biomineralization	22/1520	0.000117211	22
BP	GO:0051495	positive regulation of cytoskeleton organization	38/1520	0.000117236	38
BP	GO:0003208	cardiac ventricle morphogenesis	18/1520	0.00011789	18
BP	GO:0002687	positive regulation of leukocyte migration	27/1520	0.000125519	27
BP	GO:0010596	negative regulation of endothelial cell migration	22/1520	0.000137291	22
BP	GO:0014033	neural crest cell differentiation	21/1520	0.000137291	21
BP	GO:1903035	negative regulation of response to wounding	21/1520	0.000137291	21
BP	GO:0010720	positive regulation of cell development	46/1520	0.000137291	46
BP	GO:0030168	platelet activation	30/1520	0.000140011	30
BP	GO:0045123	cellular extravasation	18/1520	0.000143404	18
BP	GO:0061311	cell surface receptor signaling pathway involved in heart development	11/1520	0.000151769	11
BP	GO:0030048	actin filament-based movement	29/1520	0.000154406	29
BP	GO:0042063	gliogenesis	45/1520	0.000158489	45
BP	GO:0014031	mesenchymal cell development	20/1520	0.000159481	20
BP	GO:0048872	homeostasis of number of cells	41/1520	0.000165628	41
BP	GO:0031102	neuron projection regeneration	16/1520	0.000168775	16
BP	GO:0061005	cell differentiation involved in kidney development	16/1520	0.000168775	16
BP	GO:0048146	positive regulation of fibroblast proliferation	14/1520	0.00017464	14
BP	GO:0050819	negative regulation of coagulation	15/1520	0.00017464	15
BP	GO:0060350	endochondral bone morphogenesis	15/1520	0.00017464	15
BP	GO:0010770	positive regulation of cell morphogenesis involved in differentiation	19/1520	0.000182871	19
BP	GO:0001501	skeletal system development	67/1520	0.000185805	67
BP	GO:0034109	homotypic cell-cell adhesion	20/1520	0.000187345	20
BP	GO:0006893	Golgi to plasma membrane transport	16/1520	0.000209581	16
BP	GO:0044272	sulfur compound biosynthetic process	34/1520	0.00021323	34
BP	GO:0050731	positive regulation of peptidyl-tyrosine phosphorylation	34/1520	0.00021323	34
BP	GO:0002262	myeloid cell homeostasis	28/1520	0.000213399	28
BP	GO:0044091	membrane biogenesis	13/1520	0.000213399	13
BP	GO:0046596	regulation of viral entry into host cell	13/1520	0.000213399	13
BP	GO:0006939	smooth muscle contraction	22/1520	0.000213399	22
BP	GO:1903076	regulation of protein localization to plasma membrane	22/1520	0.000213399	22
BP	GO:0008064	regulation of actin polymerization or depolymerization	33/1520	0.000213399	33
BP	GO:2001214	positive regulation of vasculogenesis	7/1520	0.000213399	7
BP	GO:0007565	female pregnancy	32/1520	0.000215591	32
BP	GO:1902904	negative regulation of supramolecular fiber organization	30/1520	0.000215666	30
BP	GO:0002042	cell migration involved in sprouting angiogenesis	21/1520	0.000215666	21
BP	GO:0072109	glomerular mesangium development	8/1520	0.000219137	8
BP	GO:0030832	regulation of actin filament length	33/1520	0.000235713	33
BP	GO:0014706	striated muscle tissue development	52/1520	0.00023875	52
BP	GO:0071709	membrane assembly	12/1520	0.000251494	12
BP	GO:0061097	regulation of protein tyrosine kinase activity	21/1520	0.000252372	21
BP	GO:0014032	neural crest cell development	19/1520	0.000252372	19
BP	GO:0045445	myoblast differentiation	19/1520	0.000252372	19
BP	GO:0071375	cellular response to peptide hormone stimulus	49/1520	0.00025709	49
BP	GO:0007163	establishment or maintenance of cell polarity	36/1520	0.000261662	36
BP	GO:0043114	regulation of vascular permeability	13/1520	0.000271081	13
BP	GO:0046688	response to copper ion	13/1520	0.000271081	13
BP	GO:0030195	negative regulation of blood coagulation	14/1520	0.000275063	14
BP	GO:0022617	extracellular matrix disassembly	19/1520	0.000299648	19
BP	GO:2000810	regulation of bicellular tight junction assembly	10/1520	0.000302494	10
BP	GO:0051051	negative regulation of transport	61/1520	0.000306594	61
BP	GO:0032355	response to estradiol	25/1520	0.000306594	25
BP	GO:0022408	negative regulation of cell-cell adhesion	33/1520	0.000317249	33
BP	GO:0048846	axon extension involved in axon guidance	12/1520	0.000329955	12
BP	GO:1902284	neuron projection extension involved in neuron projection guidance	12/1520	0.000329955	12
BP	GO:0003279	cardiac septum development	21/1520	0.000340184	21
BP	GO:0008154	actin polymerization or depolymerization	36/1520	0.000346131	36
BP	GO:1900047	negative regulation of hemostasis	14/1520	0.000346422	14
BP	GO:0035265	organ growth	30/1520	0.000371517	30
BP	GO:0051222	positive regulation of protein transport	47/1520	0.000371517	47
BP	GO:0051494	negative regulation of cytoskeleton organization	29/1520	0.000373059	29
BP	GO:0001886	endothelial cell morphogenesis	7/1520	0.000392134	7
BP	GO:0014066	regulation of phosphatidylinositol 3-kinase signaling	25/1520	0.000392134	25
BP	GO:0035296	regulation of tube diameter	25/1520	0.000392134	25
BP	GO:0097746	blood vessel diameter maintenance	25/1520	0.000392134	25
BP	GO:0048738	cardiac muscle tissue development	35/1520	0.000392267	35
BP	GO:0048863	stem cell differentiation	40/1520	0.000396214	40
BP	GO:0003073	regulation of systemic arterial blood pressure	20/1520	0.000401645	20
BP	GO:0014068	positive regulation of phosphatidylinositol 3-kinase signaling	20/1520	0.000401645	20
BP	GO:0042119	neutrophil activation	67/1520	0.00040605	67
BP	GO:0048864	stem cell development	19/1520	0.000406544	19
BP	GO:1901798	positive regulation of signal transduction by p53 class mediator	9/1520	0.000419769	9
BP	GO:0060324	face development	13/1520	0.000439379	13
BP	GO:0035150	regulation of tube size	25/1520	0.000439379	25
BP	GO:0060333	interferon-gamma-mediated signaling pathway	20/1520	0.000468409	20
BP	GO:0007179	transforming growth factor beta receptor signaling pathway	34/1520	0.000492368	34
BP	GO:0071346	cellular response to interferon-gamma	31/1520	0.000492369	31
BP	GO:0030833	regulation of actin filament polymerization	30/1520	0.000503123	30
BP	GO:1905475	regulation of protein localization to membrane	33/1520	0.00051	33
BP	GO:0048675	axon extension	23/1520	0.000521962	23
BP	GO:0007517	muscle organ development	47/1520	0.000528242	47
BP	GO:0060411	cardiac septum morphogenesis	16/1520	0.000533988	16
BP	GO:0072577	endothelial cell apoptotic process	16/1520	0.000533988	16
BP	GO:0045185	maintenance of protein location	20/1520	0.00054037	20
BP	GO:0001893	maternal placenta development	10/1520	0.000583186	10
BP	GO:0051896	regulation of protein kinase B signaling	39/1520	0.000583962	39
BP	GO:0044706	multi-multicellular organism process	34/1520	0.000588224	34
BP	GO:0030218	erythrocyte differentiation	23/1520	0.00059059	23
BP	GO:2000351	regulation of endothelial cell apoptotic process	15/1520	0.000597638	15
BP	GO:1904951	positive regulation of establishment of protein localization	48/1520	0.000599831	48
BP	GO:0097193	intrinsic apoptotic signaling pathway	43/1520	0.000606686	43
BP	GO:1903055	positive regulation of extracellular matrix organization	9/1520	0.000606686	9
BP	GO:0009896	positive regulation of catabolic process	61/1520	0.000616859	61
BP	GO:0002683	negative regulation of immune system process	56/1520	0.000627466	56
BP	GO:0048588	developmental cell growth	36/1520	0.000627466	36

BP	GO:0030041	actin filament polymerization	32/1520	0.000627466	32
BP	GO:0060541	respiratory system development	32/1520	0.000627466	32
BP	GO:0007043	cell-cell junction assembly	27/1520	0.000628833	27
BP	GO:0032060	bleb assembly	6/1520	0.000630675	6
BP	GO:2000048	negative regulation of cell-cell adhesion mediated by cadherin	6/1520	0.000630675	6
BP	GO:0002446	neutrophil mediated immunity	66/1520	0.000643447	66
BP	GO:0048145	regulation of fibroblast proliferation	18/1520	0.000647718	18
BP	GO:0019058	viral life cycle	50/1520	0.000656895	50
BP	GO:0001569	branching involved in blood vessel morphogenesis	11/1520	0.000656895	11
BP	GO:1990138	neuron projection extension	29/1520	0.000677635	29
BP	GO:1903900	regulation of viral life cycle	27/1520	0.000696652	27
BP	GO:0061951	establishment of protein localization to plasma membrane	15/1520	0.000706636	15
BP	GO:0090303	positive regulation of wound healing	15/1520	0.000706636	15
BP	GO:0034332	adherens junction organization	16/1520	0.000749211	16
BP	GO:0034764	positive regulation of transmembrane transport	34/1520	0.00074945	34
BP	GO:0034767	positive regulation of ion transmembrane transport	34/1520	0.00074945	34
BP	GO:0048144	fibroblast proliferation	18/1520	0.000754241	18
BP	GO:0052548	regulation of endopeptidase activity	58/1520	0.000792934	58
BP	GO:0032271	regulation of protein polymerization	36/1520	0.000801028	36
BP	GO:0010721	negative regulation of cell development	30/1520	0.000803781	30
BP	GO:0060216	definitive hemopoiesis	8/1520	0.000830942	8
BP	GO:0043254	regulation of protein-containing complex assembly	60/1520	0.000833617	60
BP	GO:0007528	neuromuscular junction development	13/1520	0.000839599	13
BP	GO:0003094	glomerular filtration	9/1520	0.000839599	9
BP	GO:0072010	glomerular epithelium development	9/1520	0.000839599	9
BP	GO:0010232	vascular transport	19/1520	0.000842541	19
BP	GO:0034113	heterotypic cell-cell adhesion	15/1520	0.000842541	15
BP	GO:0010038	response to metal ion	50/1520	0.000842541	50
BP	GO:0043312	neutrophil degranulation	64/1520	0.000845309	64
BP	GO:0010952	positive regulation of peptidase activity	33/1520	0.000845515	33
BP	GO:0001953	negative regulation of cell-matrix adhesion	12/1520	0.000861165	12
BP	GO:0030323	respiratory tube development	29/1520	0.000899076	29
BP	GO:0071675	regulation of mononuclear cell migration	22/1520	0.000899076	22
BP	GO:0045927	positive regulation of growth	39/1520	0.000899076	39
BP	GO:0051492	regulation of stress fiber assembly	19/1520	0.000971938	19
BP	GO:0030038	contractile actin filament bundle assembly	21/1520	0.000976127	21
BP	GO:0043149	stress fiber assembly	21/1520	0.000976127	21
BP	GO:0002283	neutrophil activation involved in immune response	64/1520	0.000993867	64
BP	GO:0061384	heart trabecula morphogenesis	10/1520	0.001014129	10
BP	GO:0070168	negative regulation of biomineral tissue development	10/1520	0.001014129	10
BP	GO:0031960	response to corticosteroid	27/1520	0.001043193	27
BP	GO:0043244	regulation of protein-containing complex disassembly	23/1520	0.00104391	23
BP	GO:0045807	positive regulation of endocytosis	20/1520	0.00104391	20
BP	GO:0035051	cardiocyte differentiation	26/1520	0.001053346	26
BP	GO:0014912	negative regulation of smooth muscle cell migration	11/1520	0.001087162	11
BP	GO:0007159	leukocyte cell-cell adhesion	51/1520	0.00112864	51
BP	GO:0017145	stem cell division	9/1520	0.001139818	9
BP	GO:0031579	membrane raft organization	9/1520	0.001139818	9
BP	GO:0097205	renal filtration	9/1520	0.001139818	9
BP	GO:0030900	forebrain development	50/1520	0.001139818	50
BP	GO:0045444	fat cell differentiation	35/1520	0.001139818	35
BP	GO:0001765	membrane raft assembly	6/1520	0.001139818	6
BP	GO:0045602	negative regulation of endothelial cell differentiation	6/1520	0.001139818	6
BP	GO:0046598	positive regulation of viral entry into host cell	6/1520	0.001139818	6
BP	GO:0061299	retina vasculature morphogenesis in camera-type eye	6/1520	0.001139818	6
BP	GO:0061314	Notch signaling involved in heart development	6/1520	0.001139818	6
BP	GO:0075294	positive regulation by symbiont of entry into host	6/1520	0.001139818	6
BP	GO:0001764	neuron migration	26/1520	0.001145871	26
BP	GO:0071901	negative regulation of protein serine/threonine kinase activity	26/1520	0.001145871	26
BP	GO:1904375	regulation of protein localization to cell periphery	23/1520	0.001145871	23
BP	GO:0110020	regulation of actomyosin structure organization	20/1520	0.001164467	20
BP	GO:0045604	regulation of epidermal cell differentiation	15/1520	0.001165628	15
BP	GO:0036120	cellular response to platelet-derived growth factor stimulus	8/1520	0.001166019	8
BP	GO:0007492	endoderm development	17/1520	0.001167279	17
BP	GO:0045682	regulation of epidermis development	16/1520	0.001179229	16
BP	GO:0072132	mesenchyme morphogenesis	13/1520	0.001222284	13
BP	GO:1904062	regulation of cation transmembrane transport	48/1520	0.001246273	48
BP	GO:1903844	regulation of cellular response to transforming growth factor beta stimulus	24/1520	0.001290756	24
BP	GO:0060317	cardiac epithelial to mesenchymal transition	10/1520	0.001297501	10
BP	GO:0110150	negative regulation of biomineralization	10/1520	0.001297501	10
BP	GO:0061383	trabecula morphogenesis	12/1520	0.001311159	12
BP	GO:0030324	lung development	28/1520	0.001321785	28
BP	GO:0001649	osteoblast differentiation	35/1520	0.001321785	35
BP	GO:0001704	formation of primary germ layer	22/1520	0.001420036	22
BP	GO:0048813	dendrite morphogenesis	25/1520	0.001425326	25
BP	GO:2001234	negative regulation of apoptotic signaling pathway	35/1520	0.001438214	35
BP	GO:0051146	striated muscle cell differentiation	40/1520	0.001478372	40
BP	GO:0001894	tissue homeostasis	39/1520	0.001478372	39
BP	GO:0003229	ventricular cardiac muscle tissue development	13/1520	0.001478372	13
BP	GO:0007566	embryo implantation	13/1520	0.001478372	13
BP	GO:1904036	negative regulation of epithelial cell apoptotic process	13/1520	0.001478372	13
BP	GO:1990089	response to nerve growth factor	13/1520	0.001478372	13
BP	GO:0010464	regulation of mesenchymal cell proliferation	9/1520	0.001515334	9
BP	GO:0071674	mononuclear cell migration	31/1520	0.001523477	31
BP	GO:0001938	positive regulation of endothelial cell proliferation	21/1520	0.001545584	21
BP	GO:0030033	microvillus assembly	7/1520	0.001580446	7
BP	GO:0010977	negative regulation of neuron projection development	24/1520	0.001585444	24
BP	GO:0031346	positive regulation of cell projection organization	48/1520	0.001585505	48
BP	GO:0060711	labyrinthine layer development	12/1520	0.001606523	12
BP	GO:0019932	second-messenger-mediated signaling	44/1520	0.001606523	44
BP	GO:0032507	maintenance of protein location in cell	15/1520	0.001609842	15
BP	GO:0035455	response to interferon-alpha	8/1520	0.001637091	8
BP	GO:0036119	response to platelet-derived growth factor	8/1520	0.001637091	8
BP	GO:1902307	positive regulation of sodium ion transmembrane transport	8/1520	0.001637091	8
BP	GO:0035767	endothelial cell chemotaxis	10/1520	0.001653286	10
BP	GO:0070570	regulation of neuron projection regeneration	10/1520	0.001653286	10

BP	GO:0098801	regulation of renal system process	10/1520	0.001653286	10
BP	GO:0042176	regulation of protein catabolic process	52/1520	0.001684774	52
BP	GO:0030512	negative regulation of transforming growth factor beta receptor signaling pathway	18/1520	0.001691628	18
BP	GO:0031331	positive regulation of cellular catabolic process	52/1520	0.001792314	52
BP	GO:0051897	positive regulation of protein kinase B signaling	29/1520	0.001815906	29
BP	GO:0033627	cell adhesion mediated by integrin	16/1520	0.001857836	16
BP	GO:0042273	ribosomal large subunit biogenesis	16/1520	0.001857836	16
BP	GO:0032984	protein-containing complex disassembly	46/1520	0.001861736	46
BP	GO:0032388	positive regulation of intracellular transport	34/1520	0.001907482	34
BP	GO:0098911	regulation of ventricular cardiac muscle cell action potential	6/1520	0.001911123	6
BP	GO:0150104	transport across blood-brain barrier	18/1520	0.001941018	18
BP	GO:0060348	bone development	31/1520	0.001941018	31
BP	GO:0071470	cellular response to osmotic stress	11/1520	0.002106713	11
BP	GO:0003203	endocardial cushion morphogenesis	10/1520	0.002135565	10
BP	GO:0031103	axon regeneration	13/1520	0.002135565	13
BP	GO:0051496	positive regulation of stress fiber assembly	13/1520	0.002135565	13
BP	GO:0031214	biomineral tissue development	27/1520	0.002168324	27
BP	GO:0017015	regulation of transforming growth factor beta receptor signaling pathway	23/1520	0.002175608	23
BP	GO:1905477	positive regulation of protein localization to membrane	23/1520	0.002175608	23
BP	GO:0001101	response to acid chemical	22/1520	0.002179203	22
BP	GO:0002688	regulation of leukocyte chemotaxis	22/1520	0.002179203	22
BP	GO:0033157	regulation of intracellular protein transport	37/1520	0.00223891	37
BP	GO:0071496	cellular response to external stimulus	43/1520	0.00223891	43
BP	GO:1901184	regulation of ERBB signaling pathway	19/1520	0.002321947	19
BP	GO:0033631	cell-cell adhesion mediated by integrin	7/1520	0.002321947	7
BP	GO:0043116	negative regulation of vascular permeability	7/1520	0.002321947	7
BP	GO:0070252	actin-mediated cell contraction	22/1520	0.002436771	22
BP	GO:1903829	positive regulation of cellular protein localization	42/1520	0.002436771	42
BP	GO:0048639	positive regulation of developmental growth	28/1520	0.002475946	28
BP	GO:0048562	embryonic organ morphogenesis	40/1520	0.002515169	40
BP	GO:0042058	regulation of epidermal growth factor receptor signaling pathway	18/1520	0.002520661	18
BP	GO:0035384	thioester biosynthetic process	13/1520	0.002529238	13
BP	GO:0071616	acyl-CoA biosynthetic process	13/1520	0.002529238	13
BP	GO:1905517	macrophage migration	13/1520	0.002529238	13
BP	GO:0060041	retina development in camera-type eye	25/1520	0.002529238	25
BP	GO:0007369	gastrulation	29/1520	0.002545198	29
BP	GO:0110148	biomineralization	27/1520	0.002569586	27
BP	GO:0071384	cellular response to corticosteroid stimulus	14/1520	0.002573902	14
BP	GO:0043903	regulation of biological process involved in symbiotic interaction	31/1520	0.002669647	31
BP	GO:0043516	regulation of DNA damage response, signal transduction by p53 class mediator	10/1520	0.002669647	10
BP	GO:0045777	positive regulation of blood pressure	10/1520	0.002669647	10
BP	GO:2001236	regulation of extrinsic apoptotic signaling pathway	26/1520	0.002669647	26
BP	GO:0006970	response to osmotic stress	17/1520	0.002670903	17
BP	GO:0006631	fatty acid metabolic process	52/1520	0.00276733	52
BP	GO:0061326	renal tubule development	18/1520	0.002833328	18
BP	GO:0043536	positive regulation of blood vessel endothelial cell migration	16/1520	0.002833328	16
BP	GO:0050708	regulation of protein secretion	40/1520	0.002833328	40
BP	GO:0030199	collagen fibril organization	13/1520	0.003008605	13
BP	GO:0009226	nucleotide-sugar biosynthetic process	8/1520	0.0030746	8
BP	GO:0030111	regulation of Wnt signaling pathway	49/1520	0.0030746	49
BP	GO:0071692	protein localization to extracellular region	50/1520	0.003149001	50
BP	GO:0031333	negative regulation of protein-containing complex assembly	24/1520	0.003165766	24
BP	GO:0008361	regulation of cell size	28/1520	0.003185151	28
BP	GO:0097191	extrinsic apoptotic signaling pathway	33/1520	0.003193745	33
BP	GO:0043123	positive regulation of I-kappaB kinase/NF-kappaB signaling	29/1520	0.003275896	29
BP	GO:0010765	positive regulation of sodium ion transport	10/1520	0.003377117	10
BP	GO:0042110	T cell activation	60/1520	0.003378618	60
BP	GO:0060325	face morphogenesis	9/1520	0.003378618	9
BP	GO:0009306	protein secretion	49/1520	0.003422149	49
BP	GO:0045862	positive regulation of proteolysis	49/1520	0.003422149	49
BP	GO:0003143	embryonic heart tube morphogenesis	15/1520	0.003422149	15
BP	GO:0097529	myeloid leukocyte migration	33/1520	0.003422149	33
BP	GO:1990090	cellular response to nerve growth factor stimulus	12/1520	0.003422149	12
BP	GO:0050767	regulation of neurogenesis	47/1520	0.003439872	47
BP	GO:0007178	transmembrane receptor protein serine/threonine kinase signaling pathway	48/1520	0.003531771	48
BP	GO:0071385	cellular response to glucocorticoid stimulus	13/1520	0.003532139	13
BP	GO:0035592	establishment of protein localization to extracellular region	49/1520	0.003609079	49
BP	GO:0032409	regulation of transporter activity	40/1520	0.00366639	40
BP	GO:0048678	response to axon injury	16/1520	0.003730843	16
BP	GO:1904064	positive regulation of cation transmembrane transport	24/1520	0.003811038	24
BP	GO:0045616	regulation of keratinocyte differentiation	11/1520	0.003845129	11
BP	GO:0032386	regulation of intracellular transport	47/1520	0.003861698	47
BP	GO:0009991	response to extracellular stimulus	60/1520	0.003874325	60
BP	GO:0009612	response to mechanical stimulus	31/1520	0.003935012	31
BP	GO:0051258	protein polymerization	41/1520	0.004078093	41
BP	GO:1903901	negative regulation of viral life cycle	8/1520	0.004086571	8
BP	GO:0001947	heart looping	14/1520	0.004107035	14
BP	GO:0032412	regulation of ion transmembrane transporter activity	37/1520	0.004114803	37
BP	GO:0030308	negative regulation of cell growth	29/1520	0.004138222	29
BP	GO:0051216	cartilage development	29/1520	0.004138222	29
BP	GO:0044344	cellular response to fibroblast growth factor stimulus	24/1520	0.004159264	24
BP	GO:0007568	aging	42/1520	0.004199221	42
BP	GO:0035050	embryonic heart tube development	16/1520	0.004224621	16
BP	GO:0043537	negative regulation of blood vessel endothelial cell migration	16/1520	0.004224621	16
BP	GO:0070373	negative regulation of ERK1 and ERK2 cascade	16/1520	0.004224621	16
BP	GO:0060674	placenta blood vessel development	9/1520	0.004262009	9
BP	GO:0002790	peptide secretion	52/1520	0.004427135	52
BP	GO:0050792	regulation of viral process	29/1520	0.00446923	29
BP	GO:2001242	regulation of intrinsic apoptotic signaling pathway	26/1520	0.004524806	26
BP	GO:0048705	skeletal system morphogenesis	32/1520	0.004559932	32
BP	GO:0050764	regulation of phagocytosis	18/1520	0.004559932	18
BP	GO:0022898	regulation of transmembrane transporter activity	38/1520	0.004575154	38
BP	GO:0009415	response to water	6/1520	0.004594963	6
BP	GO:0034349	glial cell apoptotic process	6/1520	0.004594963	6
BP	GO:0071474	cellular hyperosmotic response	6/1520	0.004594963	6
BP	GO:0006470	protein dephosphorylation	44/1520	0.004695384	44

BP	GO:0051384	response to glucocorticoid	23/1520	0.004695384	23
BP	GO:0050922	negative regulation of chemotaxis	14/1520	0.004695384	14
BP	GO:0051960	regulation of nervous system development	54/1520	0.004704864	54
BP	GO:0022900	electron transport chain	28/1520	0.004706219	28
BP	GO:0042310	vasoconstriction	16/1520	0.004763969	16
BP	GO:0070830	bicellular tight junction assembly	16/1520	0.004763969	16
BP	GO:0045747	positive regulation of Notch signaling pathway	13/1520	0.004789134	13
BP	GO:0086065	cell communication involved in cardiac conduction	13/1520	0.004789134	13
BP	GO:0043297	apical junction assembly	17/1520	0.004965528	17
BP	GO:0009225	nucleotide-sugar metabolic process	10/1520	0.005043427	10
BP	GO:0035886	vascular associated smooth muscle cell differentiation	10/1520	0.005043427	10
BP	GO:0090316	positive regulation of intracellular protein transport	28/1520	0.005088795	28
BP	GO:0030032	lamellipodium assembly	15/1520	0.005104921	15
BP	GO:0000302	response to reactive oxygen species	33/1520	0.005170519	33
BP	GO:0030100	regulation of endocytosis	31/1520	0.005200728	31
BP	GO:0002791	regulation of peptide secretion	42/1520	0.005221266	42
BP	GO:0003148	outflow tract septum morphogenesis	8/1520	0.005221266	8
BP	GO:0033622	integrin activation	8/1520	0.005221266	8
BP	GO:1905048	regulation of metallopeptidase activity	8/1520	0.005221266	8
BP	GO:0045667	regulation of osteoblast differentiation	22/1520	0.005280888	22
BP	GO:0034405	response to fluid shear stress	9/1520	0.005280888	9
BP	GO:0046949	fatty-acyl-CoA biosynthetic process	9/1520	0.005280888	9
BP	GO:0021915	neural tube development	24/1520	0.005329136	24
BP	GO:0010508	positive regulation of autophagy	21/1520	0.005429659	21
BP	GO:0019882	antigen processing and presentation	34/1520	0.005429659	34
BP	GO:0010761	fibroblast migration	11/1520	0.005429659	11
BP	GO:0071248	cellular response to metal ion	29/1520	0.005501455	29
BP	GO:0072080	nephron tubule development	17/1520	0.005520692	17
BP	GO:0030010	establishment of cell polarity	23/1520	0.005552887	23
BP	GO:0043271	negative regulation of ion transport	43/1520	0.005643461	43
BP	GO:0003093	regulation of glomerular filtration	5/1520	0.005649321	5
BP	GO:0034350	regulation of glial cell apoptotic process	5/1520	0.005649321	5
BP	GO:0035907	dorsal aorta development	5/1520	0.005649321	5
BP	GO:1902946	protein localization to early endosome	5/1520	0.005649321	5
BP	GO:0031100	animal organ regeneration	15/1520	0.005739357	15
BP	GO:0043010	camera-type eye development	42/1520	0.005791412	42
BP	GO:0043409	negative regulation of MAPK cascade	28/1520	0.005847792	28
BP	GO:0120192	tight junction assembly	16/1520	0.006036646	16
BP	GO:0010463	mesenchymal cell proliferation	10/1520	0.006046283	10
BP	GO:0030866	cortical actin cytoskeleton organization	10/1520	0.006046283	10
BP	GO:2000249	regulation of actin cytoskeleton reorganization	10/1520	0.006046283	10
BP	GO:0046597	negative regulation of viral entry into host cell	7/1520	0.006097874	7
BP	GO:0046697	decidualization	7/1520	0.006097874	7
BP	GO:0097581	lamellipodium organization	17/1520	0.006157441	17
BP	GO:0050773	regulation of dendrite development	18/1520	0.006206525	18
BP	GO:0014911	positive regulation of smooth muscle cell migration	11/1520	0.006444451	11
BP	GO:0042551	neuron maturation	11/1520	0.006444451	11
BP	GO:0048841	regulation of axon extension involved in axon guidance	9/1520	0.00649305	9
BP	GO:1903902	positive regulation of viral life cycle	9/1520	0.00649305	9
BP	GO:0003417	growth plate cartilage development	6/1520	0.00649305	6
BP	GO:0010763	positive regulation of fibroblast migration	6/1520	0.00649305	6
BP	GO:0035635	entry of bacterium into host cell	6/1520	0.00649305	6
BP	GO:0072148	epithelial cell fate commitment	6/1520	0.00649305	6
BP	GO:2000136	regulation of cell proliferation involved in heart morphogenesis	6/1520	0.00649305	6
BP	GO:2000345	regulation of hepatocyte proliferation	6/1520	0.00649305	6
BP	GO:2001044	regulation of integrin-mediated signaling pathway	6/1520	0.00649305	6
BP	GO:0050926	regulation of positive chemotaxis	8/1520	0.006581365	8
BP	GO:0060351	cartilage development involved in endochondral bone morphogenesis	8/1520	0.006581365	8
BP	GO:0001656	metanephros development	16/1520	0.006707962	16
BP	GO:2000177	regulation of neural precursor cell proliferation	16/1520	0.006707962	16
BP	GO:0070665	positive regulation of leukocyte proliferation	24/1520	0.006763095	24
BP	GO:0071774	response to fibroblast growth factor	24/1520	0.006763095	24
BP	GO:0060349	bone morphogenesis	17/1520	0.00682224	17
BP	GO:0050918	positive chemotaxis	14/1520	0.00688467	14
BP	GO:0061371	determination of heart left/right asymmetry	14/1520	0.00688467	14
BP	GO:1904377	positive regulation of protein localization to cell periphery	14/1520	0.00688467	14
BP	GO:0031667	response to nutrient levels	56/1520	0.007022053	56
BP	GO:0022409	positive regulation of cell-cell adhesion	38/1520	0.007189471	38
BP	GO:0045773	positive regulation of axon extension	10/1520	0.007211488	10
BP	GO:1905521	regulation of macrophage migration	10/1520	0.007211488	10
BP	GO:0016239	positive regulation of macroautophagy	13/1520	0.007262063	13
BP	GO:1903078	positive regulation of protein localization to plasma membrane	13/1520	0.007262063	13
BP	GO:0001937	negative regulation of endothelial cell proliferation	15/1520	0.007263616	15
BP	GO:0072332	intrinsic apoptotic signaling pathway by p53 class mediator	15/1520	0.007263616	15
BP	GO:0030879	mammary gland development	22/1520	0.007426844	22
BP	GO:0001706	endoderm formation	12/1520	0.007482654	12
BP	GO:1901185	negative regulation of ERBB signaling pathway	12/1520	0.007482654	12
BP	GO:0009743	response to carbohydrate	32/1520	0.007576282	32
BP	GO:0002690	positive regulation of leukocyte chemotaxis	17/1520	0.007581085	17
BP	GO:0016053	organic acid biosynthetic process	44/1520	0.007812657	44
BP	GO:0060323	head morphogenesis	9/1520	0.007938864	9
BP	GO:0021537	telencephalon development	33/1520	0.007949068	33
BP	GO:0002053	positive regulation of mesenchymal cell proliferation	7/1520	0.007949068	7
BP	GO:0048745	smooth muscle tissue development	7/1520	0.007949068	7
BP	GO:0072574	hepatocyte proliferation	7/1520	0.007949068	7
BP	GO:0072575	epithelial cell proliferation involved in liver morphogenesis	7/1520	0.007949068	7
BP	GO:2000047	regulation of cell-cell adhesion mediated by cadherin	7/1520	0.007949068	7
BP	GO:0050729	positive regulation of inflammatory response	22/1520	0.00807428	22
BP	GO:0030500	regulation of bone mineralization	15/1520	0.008181634	15
BP	GO:0086001	cardiac muscle cell action potential	15/1520	0.008181634	15
BP	GO:0055001	muscle cell development	26/1520	0.008181634	26
BP	GO:0008593	regulation of Notch signaling pathway	19/1520	0.008181634	19
BP	GO:0031954	positive regulation of protein autophosphorylation	8/1520	0.008217683	8
BP	GO:0060512	prostate gland morphogenesis	8/1520	0.008217683	8
BP	GO:0098901	regulation of cardiac muscle cell action potential	8/1520	0.008217683	8
BP	GO:0045861	negative regulation of proteolysis	45/1520	0.008244083	45

BP	GO:0002832	negative regulation of response to biotic stimulus	18/1520	0.008292238	18
BP	GO:0008630	intrinsic apoptotic signaling pathway in response to DNA damage	18/1520	0.008292238	18
BP	GO:0120193	tight junction organization	16/1520	0.008302371	16
BP	GO:0030516	regulation of axon extension	17/1520	0.008346387	17
BP	GO:0046394	carboxylic acid biosynthetic process	43/1520	0.008400559	43
BP	GO:0071526	semaphorin-plexin signaling pathway	10/1520	0.008507703	10
BP	GO:0090092	regulation of transmembrane receptor protein serine/threonine kinase signaling pathway	35/1520	0.008556788	35
BP	GO:0086002	cardiac muscle cell action potential involved in contraction	12/1520	0.008570828	12
BP	GO:0003015	heart process	39/1520	0.008609185	39
BP	GO:0062197	cellular response to chemical stress	45/1520	0.008610428	45
BP	GO:0032680	regulation of tumor necrosis factor production	25/1520	0.008610428	25
BP	GO:0097048	dendritic cell apoptotic process	5/1520	0.008610428	5
BP	GO:2000668	regulation of dendritic cell apoptotic process	5/1520	0.008610428	5
BP	GO:0031952	regulation of protein autophosphorylation	11/1520	0.008637841	11
BP	GO:0045646	regulation of erythrocyte differentiation	11/1520	0.008637841	11
BP	GO:0034284	response to monosaccharide	29/1520	0.008709419	29
BP	GO:0071229	cellular response to acid chemical	14/1520	0.008709419	14
BP	GO:0032147	activation of protein kinase activity	43/1520	0.008765099	43
BP	GO:0003198	epithelial to mesenchymal transition involved in endocardial cushion formation	6/1520	0.008792296	6
BP	GO:0006047	UDP-N-acetylglucosamine metabolic process	6/1520	0.008792296	6
BP	GO:0060977	coronary vasculature morphogenesis	6/1520	0.008792296	6
BP	GO:0061323	cell proliferation involved in heart morphogenesis	6/1520	0.008792296	6
BP	GO:0090036	regulation of protein kinase C signaling	6/1520	0.008792296	6
BP	GO:1901201	regulation of extracellular matrix assembly	6/1520	0.008792296	6
BP	GO:0032612	interleukin-1 production	21/1520	0.008976543	21
BP	GO:2001235	positive regulation of apoptotic signaling pathway	21/1520	0.008976543	21
BP	GO:0043407	negative regulation of MAP kinase activity	15/1520	0.008976543	15
BP	GO:1901879	regulation of protein depolymerization	16/1520	0.009137784	16
BP	GO:0030166	proteoglycan biosynthetic process	13/1520	0.009283345	13
BP	GO:0050671	positive regulation of lymphocyte proliferation	22/1520	0.009350082	22
BP	GO:0046640	regulation of alpha-beta T cell proliferation	9/1520	0.009376515	9
BP	GO:0072507	divalent inorganic cation homeostasis	60/1520	0.009458284	60
BP	GO:0001654	eye development	46/1520	0.00949681	46
BP	GO:0048880	sensory system development	47/1520	0.009571043	47
BP	GO:0071241	cellular response to inorganic substance	31/1520	0.009686923	31
BP	GO:0001755	neural crest cell migration	12/1520	0.009768671	12
BP	GO:1901880	negative regulation of protein depolymerization	14/1520	0.009808455	14
BP	GO:0032640	tumor necrosis factor production	25/1520	0.009922943	25
BP	GO:0031668	cellular response to extracellular stimulus	33/1520	0.009922943	33
BP	GO:0030850	prostate gland development	10/1520	0.009922943	10
BP	GO:0033574	response to testosterone	10/1520	0.009922943	10
BP	GO:0090279	regulation of calcium ion import	10/1520	0.009922943	10
BP	GO:0072503	cellular divalent inorganic cation homeostasis	58/1520	0.009928884	58
BP	GO:0001913	T cell mediated cytotoxicity	11/1520	0.009958638	11
BP	GO:2001238	positive regulation of extrinsic apoptotic signaling pathway	11/1520	0.009958638	11
BP	GO:0000027	ribosomal large subunit assembly	8/1520	0.009958638	8
BP	GO:0044331	cell-cell adhesion mediated by cadherin	8/1520	0.009958638	8
BP	GO:0071280	cellular response to copper ion	8/1520	0.009958638	8
BP	GO:0060343	trabecula formation	7/1520	0.009958638	7
BP	GO:0072576	liver morphogenesis	7/1520	0.009958638	7
BP	GO:0032272	negative regulation of protein polymerization	15/1520	0.009958638	15
BP	GO:0042982	amyloid precursor protein metabolic process	17/1520	0.009968598	17
BP	GO:0030595	leukocyte chemotaxis	32/1520	0.009968598	32
BP	GO:0032946	positive regulation of mononuclear cell proliferation	22/1520	0.009968598	22
BP	GO:0045598	regulation of fat cell differentiation	22/1520	0.009968598	22
CC	GO:0005925	focal adhesion	143/1576	4.16E-51	143
CC	GO:0030055	cell-substrate junction	143/1576	2.23E-50	143
CC	GO:0022626	cytosolic ribosome	56/1576	1.00E-30	56
CC	GO:0022625	cytosolic large ribosomal subunit	37/1576	9.48E-25	37
CC	GO:0044391	ribosomal subunit	64/1576	2.21E-22	64
CC	GO:0005911	cell-cell junction	110/1576	7.42E-22	110
CC	GO:0005840	ribosome	70/1576	9.11E-20	70
CC	GO:0031252	cell leading edge	91/1576	2.68E-17	91
CC	GO:0015934	large ribosomal subunit	42/1576	7.12E-16	42
CC	GO:0062023	collagen-containing extracellular matrix	89/1576	1.65E-15	89
CC	GO:0001726	ruffle	52/1576	2.36E-15	52
CC	GO:0042788	polysomal ribosome	21/1576	2.81E-14	21
CC	GO:0005912	adherens junction	49/1576	1.04E-13	49
CC	GO:0045121	membrane raft	71/1576	1.87E-13	71
CC	GO:0098857	membrane microdomain	71/1576	1.87E-13	71
CC	GO:0030027	lamellipodium	46/1576	1.12E-09	46
CC	GO:0005844	polysome	25/1576	1.28E-09	25
CC	GO:0032432	actin filament bundle	25/1576	7.16E-09	25
CC	GO:0001725	stress fiber	23/1576	1.13E-08	23
CC	GO:0097517	contractile actin filament bundle	23/1576	1.13E-08	23
CC	GO:0042641	actomyosin	24/1576	4.99E-08	24
CC	GO:0022627	cytosolic small ribosomal subunit	18/1576	1.67E-07	18
CC	GO:0031093	platelet alpha granule lumen	22/1576	1.67E-07	22
CC	GO:0031256	leading edge membrane	38/1576	2.76E-07	38
CC	GO:0042611	MHC protein complex	13/1576	3.07E-07	13
CC	GO:0005788	endoplasmic reticulum lumen	55/1576	3.44E-07	55
CC	GO:0005604	basement membrane	26/1576	3.63E-07	26
CC	GO:0032587	ruffle membrane	26/1576	4.45E-07	26
CC	GO:0015935	small ribosomal subunit	22/1576	1.03E-06	22
CC	GO:0030134	COPII-coated ER to Golgi transport vesicle	25/1576	1.44E-06	25
CC	GO:0031091	platelet alpha granule	24/1576	3.01E-06	24
CC	GO:0034774	secretory granule lumen	54/1576	4.07E-06	54
CC	GO:0098858	actin-based cell projection	41/1576	4.07E-06	41
CC	GO:0012507	ER to Golgi transport vesicle membrane	19/1576	4.20E-06	19
CC	GO:0060205	cytoplasmic vesicle lumen	54/1576	5.61E-06	54
CC	GO:0031983	vesicle lumen	54/1576	6.67E-06	54
CC	GO:0031253	cell projection membrane	55/1576	6.77E-06	55
CC	GO:0005902	microvillus	23/1576	1.02E-05	23
CC	GO:0005901	caveola	21/1576	1.54E-05	21
CC	GO:0071556	integral component of lumenal side of endoplasmic reticulum membrane	12/1576	1.60E-05	12
CC	GO:0098553	lumenal side of endoplasmic reticulum membrane	12/1576	1.60E-05	12

CC	GO:0043292	contractile fiber	41/1576	2.01E-05	41
CC	GO:0045177	apical part of cell	62/1576	2.30E-05	62
CC	GO:0005884	actin filament	25/1576	4.15E-05	25
CC	GO:0030135	coated vesicle	47/1576	7.46E-05	47
CC	GO:0016324	apical plasma membrane	53/1576	9.49E-05	53
CC	GO:0030139	endocytic vesicle	48/1576	9.63E-05	48
CC	GO:0030658	transport vesicle membrane	36/1576	0.000108889	36
CC	GO:0005938	cell cortex	47/1576	0.000118713	47
CC	GO:0030016	myofibril	38/1576	0.000121296	38
CC	GO:0098576	lumenal side of membrane	12/1576	0.000182172	12
CC	GO:0030666	endocytic vesicle membrane	30/1576	0.00019809	30
CC	GO:0030175	filopodium	22/1576	0.000240112	22
CC	GO:0030133	transport vesicle	57/1576	0.000249978	57
CC	GO:0030662	coated vesicle membrane	32/1576	0.000260333	32
CC	GO:0030863	cortical cytoskeleton	22/1576	0.00026653	22
CC	GO:0030864	cortical actin cytoskeleton	18/1576	0.000419119	18
CC	GO:0030667	secretory granule membrane	45/1576	0.000639641	45
CC	GO:0044853	plasma membrane raft	22/1576	0.000721562	22
CC	GO:0030017	sarcomere	33/1576	0.000900902	33
CC	GO:0043296	apical junction complex	25/1576	0.000958271	25
CC	GO:0042613	MHC class II protein complex	7/1576	0.001336103	7
CC	GO:0070160	tight junction	23/1576	0.001350072	23
CC	GO:0016327	apicolateral plasma membrane	8/1576	0.001365502	8
CC	GO:0030427	site of polarized growth	30/1576	0.001618117	30
CC	GO:0030426	growth cone	29/1576	0.002033369	29
CC	GO:0045335	phagocytic vesicle	24/1576	0.002033369	24
CC	GO:0002102	podosome	9/1576	0.00221906	9
CC	GO:0009897	external side of plasma membrane	53/1576	0.002489513	53
CC	GO:0014704	intercalated disc	12/1576	0.002939965	12
CC	GO:0044291	cell-cell contact zone	15/1576	0.002939965	15
CC	GO:0030670	phagocytic vesicle membrane	16/1576	0.003205081	16
CC	GO:0005923	bicellular tight junction	21/1576	0.003559984	21
CC	GO:0014069	postsynaptic density	43/1576	0.003559984	43
CC	GO:0097386	glial cell projection	9/1576	0.003581028	9
CC	GO:0098984	neuron to neuron synapse	46/1576	0.003581028	46
CC	GO:0042383	sarcolemma	23/1576	0.0035822	23
CC	GO:0045178	basal part of cell	37/1576	0.003639005	37
CC	GO:0009925	basal plasma membrane	35/1576	0.00369567	35
CC	GO:0043025	neuronal cell body	59/1576	0.0045273	59
CC	GO:0032279	asymmetric synapse	43/1576	0.004546186	43
CC	GO:0005769	early endosome	49/1576	0.00512908	49
CC	GO:0030018	Z disc	21/1576	0.005560485	21
CC	GO:0099572	postsynaptic specialization	44/1576	0.006133519	44
CC	GO:0030669	clathrin-coated endocytic vesicle membrane	10/1576	0.006133519	10
CC	GO:0042581	specific granule	25/1576	0.007720301	25
CC	GO:0031674	I band	22/1576	0.007720301	22
CC	GO:0044295	axonal growth cone	8/1576	0.009015088	8
CC	GO:0031258	lamellipodium membrane	7/1576	0.009015088	7
MF	GO:0003735	structural constituent of ribosome	64/1534	1.02E-21	64
MF	GO:0003779	actin binding	95/1534	1.44E-15	95
MF	GO:0045296	cadherin binding	79/1534	2.28E-15	79
MF	GO:0005178	integrin binding	44/1534	1.72E-12	44
MF	GO:0019838	growth factor binding	41/1534	4.21E-11	41
MF	GO:0098631	cell adhesion mediator activity	25/1534	1.83E-10	25
MF	GO:0098632	cell-cell adhesion mediator activity	21/1534	1.16E-08	21
MF	GO:0005201	extracellular matrix structural constituent	42/1534	1.34E-08	42
MF	GO:0005518	collagen binding	24/1534	5.19E-08	24
MF	GO:0030695	GTPase regulator activity	76/1534	1.28E-06	76
MF	GO:0019199	transmembrane receptor protein kinase activity	24/1534	1.71E-06	24
MF	GO:0098641	cadherin binding involved in cell-cell adhesion	11/1534	2.05E-06	11
MF	GO:0005085	guanyl-nucleotide exchange factor activity	41/1534	2.97E-06	41
MF	GO:0050840	extracellular matrix binding	19/1534	3.21E-06	19
MF	GO:0004860	protein kinase inhibitor activity	21/1534	7.30E-06	21
MF	GO:0019210	kinase inhibitor activity	21/1534	1.99E-05	21
MF	GO:0051015	actin filament binding	40/1534	3.02E-05	40
MF	GO:0019887	protein kinase regulator activity	37/1534	5.49E-05	37
MF	GO:0004714	transmembrane receptor protein tyrosine kinase activity	18/1534	7.64E-05	18
MF	GO:1901681	sulfur compound binding	45/1534	0.000108562	45
MF	GO:0005539	glycosaminoglycan binding	41/1534	0.000108562	41
MF	GO:0042605	peptide antigen binding	12/1534	0.000234977	12
MF	GO:0019207	kinase regulator activity	39/1534	0.000247275	39
MF	GO:0017124	SH3 domain binding	27/1534	0.00024863	27
MF	GO:0048407	platelet-derived growth factor binding	7/1534	0.00027408	7
MF	GO:0004857	enzyme inhibitor activity	57/1534	0.000625377	57
MF	GO:0030020	extracellular matrix structural constituent conferring tensile strength	13/1534	0.000651141	13
MF	GO:0043394	proteoglycan binding	12/1534	0.00074655	12
MF	GO:0016247	channel regulator activity	28/1534	0.00080036	28
MF	GO:0005096	GTPase activator activity	44/1534	0.000810191	44
MF	GO:0008201	heparin binding	30/1534	0.001149865	30
MF	GO:0005543	phospholipid binding	63/1534	0.001165413	63
MF	GO:0030165	PDZ domain binding	19/1534	0.00128556	19
MF	GO:0005520	insulin-like growth factor binding	10/1534	0.002165091	10
MF	GO:0030674	protein-macromolecule adaptor activity	41/1534	0.002170689	41
MF	GO:0051020	GTPase binding	36/1534	0.002449147	36
MF	GO:0099106	ion channel regulator activity	23/1534	0.002449147	23
MF	GO:0033218	amide binding	55/1534	0.002477016	55
MF	GO:0005161	platelet-derived growth factor receptor binding	7/1534	0.002536521	7
MF	GO:0031267	small GTPase binding	32/1534	0.002622893	32
MF	GO:0086080	protein binding involved in heterotypic cell-cell adhesion	6/1534	0.002624305	6
MF	GO:0008307	structural constituent of muscle	12/1534	0.002743268	12
MF	GO:0004713	protein tyrosine kinase activity	25/1534	0.002928994	25
MF	GO:0005024	transforming growth factor beta-activated receptor activity	6/1534	0.00766769	6
MF	GO:0031994	insulin-like growth factor I binding	6/1534	0.00766769	6
MF	GO:0044325	ion channel binding	23/1534	0.007895194	23
MF	GO:0043236	laminin binding	9/1534	0.008864282	9
MF	GO:0009055	electron transfer activity	24/1534	0.00939668	24
