# Supplementary material for: Integrative Analysis of Bulk RNA-Seq and Single-Cell RNA-Seq Unveils the Characteristics of the Immune Microenvironment and Prognosis Signature in Prostate Cancer
Source: J Oncol. 2022 Jul 19;2022:6768139. doi: 10.1155/2022/6768139 (PMC9325591; doi:10.1155/2022/6768139)
Supplement: Supplementary Materials — Figure S1. Workflow of the analysis. Figure S2. Validation of the risk score model using the GSE54460 dataset. A. Patients with prostate cancer (PRAD) in the GSE54460 cohort are listed in ascending order of risk score. B. Progression-free interval (PFI) distribution versus the risk score of each patient in the GSE54460 cohort. C. Kaplan–Meier (KM) curves of patients with different risk levels in the GSE54460 validation set. D. Receiver Operating Characteristic (ROC) curve analysis for 1-, 3- and 5-year PFI using the clinical information of patients of the GSE54460 validation dataset. Figure S3. Validation of the risk score model using the GSE46602 dataset. A. Patients with prostate cancer (PRAD) in the GSE46602 cohort are listed in ascending order of risk score. B. Progression-free interval (PFI) distribution versus the risk score of each patient in the GSE46602 cohort. C. Kaplan–Meier (KM) curves of patients with different risk levels in the GSE46602 validation dataset. D. Receiver Operating Characteristic (ROC) curve analysis for 1-, 3- and 5-year PFI using the clinical information of patients of the GSE46602 validation dataset. Figure S4. Validation of the risk score model using the GSE70768 dataset. A. Patients with prostate cancer (PRAD) in the GSE70768 cohort are listed in ascending order of risk score. B. Progression-free interval (PFI) distribution versus the risk score of each patient in the GSE70768 cohort. C. Kaplan–Meier (KM) curves of patients with different risk levels in the GSE70768 validation dataset. D. Receiver Operating Characteristic (ROC) curve analysis for 1-, 3- and 5-year PFI using the clinical information of patients of the GSE70768 validation dataset. Figure S5. Validation of the risk score model using the GSE70769 dataset. A. Patients with prostate cancer (PRAD) in the GSE70769 validation dataset are listed in ascending order of risk score. B. Progression-free interval (PFI) distribution versus the risk score of each patient in the GSE707 [file 6768139.f1.zip › 6768139.f1/Table S14.pdf]

SYMBOL	ENTREZID
MFSD2A	84879
SERPINA5	5104
ACSL6	23305
AKR1B1	231
MCF2	4168
KLHL14	57565
EMX2	2018
NDRG4	65009
SLC2A9	56606
HOXB8	3218
SPINK2	6691
EMX2OS	196047
RASL10B	91608
CRTAC1	55118
CYSLTR2	57105
CLDN2	9075
KCP	375616
ANO1	55107
PAQR8	85315
KCNJ5	3762
ABCG2	9429
PATE2	399967
ST6GALNA	27090
CA2	760
PTGES	9536
PNMT	5409
STAC2	342667
GCNT4	51301
HOXB6	3216
PLA2G4A	5321
WWC3	55841
FRMD3	257019
RAB11FIP2	22841
MGAM	8972
ANXA13	312
EPHA10	284656
PRDM16	63976
SLC16A12	387700
QPRT	23475
PIP	5304
PLLP	51090
SLC45A2	51151
HPN	3249
RHOBTB2	23221
NKX2-3	159296
SNAP25	6616
FUT3	2525
GNAO1	2775
GSTM3	2947
ABHD6	57406
G0S2	50486
LPCAT2	54947
EFNB1	1947
KCNJ15	3772
GLIS3	169792
KCNQ1	3784
LAPTM4A	9741

ARHGEF38	54848
UNC5B	219699
PAX2	5076
LPL	4023
HOXC6	3223
GPRC5B	51704
SIM2	6493
GSTP1	2950
AMACR	23600
SP6	80320
PIK3C2G	5288
ONECUT2	9480
HOXB9	3219
SLC31A2	1318
PALM3	342979
ZIC2	7546
NAGS	162417
CLU	1191
MICALL1	85377
TMEM114	283953
GNAZ	2781
PRPS2	5634
GRAMD1B	57476
CDK18	5129
PJA1	64219
FAM167A	83648
ZNF185	7739
ZNF219	51222
CAMK2G	818
TRIM36	55521
ETNK2	55224
CGREF1	10669
C11orf45	219833
APOBEC3(	60489
EZH2	2146
ZIC5	85416
AOX1	316
UCN	7349
PATE4	399968
PLBD1	79887
SLCO2A1	6578
APOBEC3(	27350
RASAL1	8437
PAQR7	164091
PDE7B	27115
SNPH	9751
PYCR1	5831
YPEL1	29799
LRFN1	57622
SLC19A1	6573
ISYNA1	51477
CA14	23632
TMLHE	55217
LIPG	9388
NETO2	81831
LMX1B	4010
SCARA3	51435
MARCKSL1	65108

SLC13A2	9058
B3GNT8	374907
PTGS1	5742
AFAP1L2	84632
HOXB7	3217
SLMAP	7871
DNAH5	1767
NYNRIN	57523
SLC26A6	65010
TGFBR3	7049
AIF1L	83543
RRAS	6237
PDK4	5166
DOK4	55715
PTGS2	5743
RAB17	64284
SNHG3	8420
RBM38	55544
TSPAN18	90139
SLIT1	6585
VWA5B2	90113
ALDH1L2	160428
SMPDL3B	27293
CPLX1	10815
ME1	4199
BIRC5	332
C2orf88	84281
SOX15	6665
UGT2B4	7363
NDRG2	57447
RAB9B	51209
E2F5	1875
SLC26A3	1811
DUSP2	1844
DLGAP5	9787
SNHG4	724102
PHGR1	644844
ANXA9	8416
GATM	2628
GAS2L1	10634
ANGPT1	284
ARC	23237
PAX8	7849
PLEKHA2	59339
POU2F3	25833
STIL	6491
ILK	3611
KCNJ16	3773
NEK5	341676
MMP26	56547
NEURL1B	54492
C1QTNF1	114897
NLRP12	91662
AQP2	359
AMOT	154796
ARL4D	379
SEC23A	10484
TRPM4	54795

PCSK6	5046
KIF4A	24137
ATP8A2	51761
CST2	1470
KCNIP3	30818
PLEKHG3	26030
VWCE	220001
CAMKK2	10645
PCA3	50652
TBX2	6909
LYVE1	10894
RRM2	6241
PRR7	80758
FBLIM1	54751
LRRC56	115399
RANBP17	64901
DUOX1	53905
SLC25A45	283130
CAV1	857
SLPI	6590
IL18R1	8809
RHBDL3	162494
CCNA2	890
TCF7L1	83439
PCDH7	5099
ANO6	196527
TP53INP1	94241
C2CD4C	126567
PIP5K1B	8395
C2orf72	257407
TMEM37	140738
MELK	9833
HMMR	3161
MYBL2	4605
ALB	213
TLE2	7089
PDIA2	64714
COL2A1	1280
MATK	4145
LMO3	55885
SAMD12	401474
PAQR6	79957
APOC1	341
PAK3	5063
CRISP1	167
GOLM1	51280
ESRP2	80004
C12orf75	387882
NOX4	50507
CHEK1	1111
KCNG3	170850
MKI67	4288
IRF5	3663
TES	26136
MAMLD1	10046
SCPEP1	59342
RASL11B	65997
HOXB2	3212

CTU1	90353
ZNF485	220992
ZNF154	7710
SNORD10[illegible]	692227
GTSE1	51512
SLCO3A1	28232
CHTF18	63922
HFE	3077
SBK1	388228
CBX8	57332
STX19	415117
FRMD6	122786
CTTNBP2	83992
TMEM132[illegible]	92293
B4GALNT[illegible]	338707
RIMS3	9783
HOXC4	3221
PAEP	5047
GATA3	2625
CCDC78	124093
C16orf74	404550
ASPN	54829
NCAPG	64151
KCTD14	65987
C22orf23	84645
NCS1	23413
PDLIM5	10611
HSPA6	3310
PAQR5	54852
PLP2	5355
DEPDC1B	55789
POU5F1B	5462
FOXQ1	94234
APOF	319
DBNDD2	55861
SVIL	6840
MECOM	2122
DCHS2	54798
RHPN1	114822
B3GNT9	84752
DMPK	1760
PGF	5228
GSTM4	2948
DLX2	1746
FAM110C	642273
SNCG	6623
SYT8	90019
DNAJB5	25822
MPP2	4355
GNAL	2774
DNAH14	127602
CTF1	1489
DAAM2	23500
CCDC69	26112
TPM1	7168
ACSM1	116285
NACC2	138151
FXYD6	53826

TLCD1	116238
GDPD1	284161
F2RL2	2151
EYA4	2070
NUP210	23225
AVPI1	60370
SEMA6D	80031
PALLD	23022
PCP4L1	654790
BICD1	636
AMH	268
SLC7A11	23657
WFDC2	10406
SKA3	221150
HJURP	55355
TSPAN19	144448
JPH4	84502
CERK	64781
GPR160	26996
EPHA2	1969
L3MBTL4	91133
PAX1	5075
RASL12	51285
ARHGAP2(	57569
PRICKLE2	166336
DAB2IP	153090
MOV10L1	54456
C19orf48	84798
RAPGEFL1	51195
ACE	1636
KCTD17	79734
FEZ1	9638
KIF9	64147
CDC20	991
ASPHD1	253982
TMEM139	135932
CASKIN1	57524
CDHR1	92211
CAV2	858
STAC	6769
BUB1B	701
GPX2	2877
KITLG	4254
ZNF30	90075
EBF2	64641
CFL2	1073
EPHB1	2047
ABCC4	10257
TGFB1I1	7041
BHMT2	23743
MCC	4163
ADAM2	2515
ALDH1A2	8854
KIT	3815
TMEM132/	54972
SERPINF2	5345
CCDC40	55036
PLCD3	113026

VCL	7414
TCN2	6948
FERMT2	10979
HSPA4L	22824
HPX	3263
RBMS3	27303
CORO1C	23603
ANP32E	81611
TMTC4	84899
MR1	3140
PDZRN4	29951
GSC	145258
TARBP1	6894
ACSF2	80221
DEFB132	400830
EPCAM	4072
ADAT2	134637
PIK3R1	5295
RNF157	114804
CCDC8	83987
QRICH2	84074
GAL	51083
CYP4F8	11283
UNC5A	90249
PRKCB	5579
MAP1A	4130
KANK2	25959
GFRA2	2675
COL4A6	1288
CORO2B	10391
FAM107B	83641
A4GALT	53947
GAS6	2621
PODXL2	50512
CACNA1D	776
TWIST1	7291
TINAGL1	64129
MAMDC2	256691
SEMA3B	7869
CEND1	51286
ARHGAP2[illegible]	57636
FABP5	2171
SPINK1	6690
TMEM184[illegible]	202915
ATG9B	285973
CHRDL1	91851
RALGAPA[illegible]	57186
DBNDD1	79007
SGCB	6443
ADAMTS5	11096
KIAA0087	9808
NPR2	4882
CLIP4	79745
CRABP2	1382
HSPB8	26353
MUC15	143662
RAB38	23682
CNTFR	1271

DIXDC1	85458
ANXA6	309
ELL3	80237
ASB2	51676
EFEMP2	30008
NECAB1	64168
UBXN10	127733
ZNF853	54753
FAM107A	11170
HOXB4	3214
SMTN	6525
MSLN	10232
GPR161	23432
CDC25C	995
ACOX2	8309
NHS	4810
MCOLN2	255231
MYLK	4638
RBPMS2	348093
C9orf163	158055
CAPG	822
MTG1	92170
DLX1	1745
RIMKLA	284716
TDRD1	56165
ANXA2P2	304
CPT1B	1375
AHNAK2	113146
MSRB3	253827
PNCK	139728
LUZP2	338645
CCDC110	256309
SLC16A5	9121
CHST11	50515
PDLIM7	9260
PRKG1	5592
CDC42EP3	10602
PEG3	5178
CENPM	79019
POPDC2	64091
PVT1	5820
PPP2R2B	5521
RPL22L1	200916
SLCO1A2	6579
MXD3	83463
ASPA	443
CACHD1	57685
DUOXA1	90527
RGS10	6001
TLR2	7097
CDCA8	55143
HES4	57801
ANKRD34[illegible]	340120
PPP1R3C	5507
ANGPTL3	27329
NT5E	4907
DPYSL3	1809
TRIP6	7205

ARHGDIG	398
RGS11	8786
FGFRL1	53834
RND2	8153
NAV2	89797
LRGUK	136332
CDH23	64072
SLC10A5	347051
FGF10	2255
LIMS2	55679
PDE1C	5137
ZNF90	7643
FAM47E	1E+08
IQGAP3	128239
ITGA2	3673
CLIC4	25932
CDCA5	113130
POU3F3	5455
SH3PXD2E	285590
JAZF1	221895
GPC2	221914
PLAG1	5324
OTX1	5013
MSI1	4440
PPARGC1E	133522
SPON1	10418
NRG2	9542
TEAD1	7003
ZNF516	9658
NSUN5P1	155400
LMOD1	25802
CDK5R1	8851
ATP2B4	493
C14orf132	56967
TRPC6	7225
GCNT1	2650
GNG13	51764
SLC18A2	6571
SEMG2	6407
CLIP2	7461
SYNM	23336
BIK	638
PKIB	5570
MAK	4117
PLIN4	729359
GPR89A	653519
CLIP3	25999
FBXO17	115290
HOXD13	3239
RPGRIP1	57096
F12	2161
NKX6-1	4825
MAMSTR	284358
STOX2	56977
TROAP	10024
WNT10A	80326
FOXB2	442425
CALD1	800

OR51E2	81285
STARD8	9754
ERBB4	2066
DMD	1756
CELSR2	1952
TMEM200	399474
MXRA7	439921
YJEFN3	374887
CYP4B1	1580
MBNL2	10150
ADCY5	111
NACAD	23148
C3orf70	285382
RAB6C	84084
GMDS	2762
RERG	85004
ZP3	7784
CABP4	57010
ELAVL2	1993
DIAPH3	81624
COL21A1	81578
SLC22A10	387775
CSRNP3	80034
CENPF	1063
FOXM1	2305
ST6GALNA	10610
C10orf95	79946
KLHL35	283212
EFEMP1	2202
MYOF	26509
PGM5	5239
MYOCD	93649
PPP1R14A	94274
ACACB	32
TACC1	6867
TRIM6	117854
SHCBP1	79801
C14orf39	317761
CSRP1	1465
POLN	353497
SLC34A2	10568
TGM3	7053
LPAR1	1902
DUOX2	50506
ZNF577	84765
ACSL4	2182
RNASE1	6035
S100A6	6277
PGR	5241
QSOX1	5768
RARG	5916
CENPA	1058
SPC25	57405
GRIK5	2901
KCNMB1	3779
BEND4	389206
LYNX1	66004
DNAJB4	11080

VWA5A	4013
KSR1	8844
KRT16	3868
RBP1	5947
TRIP13	9319
COL23A1	91522
NIPAL4	348938
KIF18B	146909
SEMG1	6406
CCNB2	9133
TSLP	85480
FBXL22	283807
EHD2	30846
GPR89B	51463
SLC47A1	55244
FHOD3	80206
RAP1GAP	5909
GLDN	342035
EFHD1	80303
PARM1	25849
LGR6	59352
HAGHL	84264
ENO2	2026
SLC43A1	8501
METTL7A	25840
LHX6	26468
PIPOX	51268
PPARGC1A	10891
ITGA3	3675
SLC39A2	29986
EMILIN3	90187
FFAR2	2867
JPH2	57158
FGL1	2267
S100A16	140576
KCNMA1	3778
ADRA1A	148
VGF	7425
KLB	152831
PNMA1	9240
SLC24A3	57419
SLC41A2	84102
LIME1	54923
CYP26B1	56603
SLC7A5	8140
GLIS1	148979
PLA2G7	7941
CTHRC1	115908
SCN2B	6327
CYP2E1	1571
SH3RF1	57630
HCG11	493812
NETO1	81832
SYDE1	85360
FZD8	8325
PHYHIP	9796
SLC25A27	9481
RASSF5	83593

CAPN6	827
TMEM108	66000
PDGFD	80310
CHST3	9469
P2RY2	5029
PLK1	5347
REC8	9985
FOLR1	2348
SOX7	83595
HSPB1	3315
ERCC6L	54821
KCNH2	3757
CDO1	1036
RTN4RL2	349667
IER3	8870
KIFC1	3833
PRC1	9055
SYNPO2	171024
TUBA4A	7277
CPEB1	64506
PHYHIPL	84457
KCNQ4	9132
SPEG	10290
TPX2	22974
P2RX2	22953
ENTPD3	956
PRKCA	5578
SORBS1	10580
MANEAL	149175
P2RX5	5026
TGM1	7051
HR	55806
AK5	26289
C19orf18	147685
SNHG8	1E+08
KCNJ8	3764
KCNQ5	56479
SPDYA	245711
MEIS2	4212
TMEM100	55273
SLC8A1	6546
ID4	3400
FAM111B	374393
SLC22A17	51310
ANK2	287
C21orf62	56245
PRODH	5625
LY6D	8581
ANGPTL1	9068
REEP2	51308
ARHGEF19	128272
CA12	771
PRR16	51334
SPOCK3	50859
EFS	10278
GPM6B	2824
ZMYND10	51364
IGSF1	3547

SAMD14	201191
DZIP1	22873
RHOB	388
DNAJB1	3337
CPAMD8	27151
DDR2	4921
GALNT6	11226
SPAG5	10615
EMP3	2014
B3GNT6	192134
SLCO1B3	28234
PPP1R12B	4660
CNN1	1264
NEFM	4741
RGN	9104
CDC25A	993
TUBB6	84617
EDNRA	1909
ZP1	22917
CAMK2N2	94032
HOXB3	3213
IRAK3	11213
S100A14	57402
ROR2	4920
BUB1	699
CECR2	27443
SCUBE1	80274
SLC6A11	6538
FOLH1	2346
PRRG4	79056
TMEM158	25907
ZBTB7C	201501
LONRF3	79836
SSPN	8082
IL1RL1	9173
RNF112	7732
CCDC7	79741
NCAPH	23397
FILIP1	27145
MARVELD	83742
GLYATL1	92292
NOTCH1	4851
FAM162B	221303
RAB40A	142684
KRT20	54474
HPD	3242
DDIT4	54541
TNS1	7145
ZDHHC11	79844
PLCL1	5334
GJA1	2697
SPC24	147841
IQCA1	79781
KIF20A	10112
HLF	3131
FUT2	2524
CDCA3	83461
ITGA9	3680

ACSS3	79611
TRO	7216
ASS1	445
PHF21B	112885
ZNF804A	91752
NFE2L3	9603
DUSP15	128853
ATP2A3	489
CCNJL	79616
CCBE1	147372
SLITRK6	84189
LRRIQ1	84125
REPS2	9185
KRT13	3860
SDS	10993
GJB1	2705
KCNAB1	7881
NOL4	8715
GPLD1	2822
COLEC12	81035
KLK15	55554
ITGB3	3690
CCL18	6362
CHST2	9435
GPRASP1	9737
GPR37	2861
APOBEC3D	140564
PFKFB3	5209
BAIAP2L2	80115
RASD2	23551
DOCK3	1795
SEMA3A	10371
KCNS1	3787
PITPNM3	83394
FAT2	2196
GSTM1	2944
DUSP4	1846
LRRK2	120892
NLGN3	54413
AURKB	9212
DIO1	1733
C5orf49	134121
GLI3	2737
NEK2	4751
HS3ST3A1	9955
DYNC1I1	1780
ITIH5	80760
ZGLP1	1E+08
SERPINA1	5265
HAAO	23498
ROBO1	6091
IRX2	153572
CATSPERB	79820
CDC45	8318
CTNND2	1501
B3GAT1	27087
FZD7	8324
ROPN1B	152015

LRP4	4038
SCGB3A1	92304
NLRP8	126205
COL17A1	1308
SDK1	221935
TTLL6	284076
ITPRIPL1	150771
PITX1	5307
EME2	197342
CCNB1	891
RPSAP9	653162
RAC3	5881
SLC26A1	10861
SAMD5	389432
PTGIS	5740
GPRIN1	114787
RAB19	401409
CAND2	23066
TMSB15A	11013
CD40	958
CYBA	1535
KIF26B	55083
CCNE2	9134
PATE1	160065
ITGB8	3696
AJAP1	55966
GHRHR	2692
APBA2	321
ASPM	259266
HIF3A	64344
TRHDE	29953
CYP2J2	1573
MAML2	84441
EVX2	344191
CSPG4	1464
TFF3	7033
SCNN1D	6339
CLCA2	9635
SNHG10	283596
TUBB2A	7280
SKA1	220134
DTNA	1837
ANXA2	302
SOSTDC1	25928
CDKL1	8814
BLM	641
BNIPL	149428
PTGDS	5730
KCNG1	3755
ID3	3399
PRR22	163154
ACTC1	70
HEPH	9843
CYP4F22	126410
GJC1	10052
VAV3	10451
PLS3	5358
GSTM5	2949

MRGPRF	116535
RGS7BP	401190
PCDHGA1	56114
FAT3	120114
BRSK2	9024
ERG	2078
IQSEC3	440073
PDE2A	5138
MAL	4118
CENPN	55839
RGS22	26166
GDF15	9518
RAB34	83871
HBB	3043
TIMP3	7078
NUF2	83540
RFX3	5991
MEIS1	4211
CHKB-CPT	386593
ATOH1	474
SNAI2	6591
ITGBL1	9358
EDNRB	1910
SNHG12	85028
CDKN3	1033
GLOD5	392465
KLF5	688
SRD5A2	6716
NMUR1	10316
BEND3	57673
TTK	7272
KIF11	3832
MYRIP	25924
GNG11	2791
PRDM8	56978
NCAM1	4684
SLIT3	6586
CD200	4345
SFRP5	6425
SEC31B	25956
GJB5	2709
NOX1	27035
PBK	55872
CENPE	1062
PTTG1	9232
C1QL1	10882
COL10A1	1300
SLITRK3	22865
UGT1A3	54659
HSF4	3299
MEX3A	92312
SOX8	30812
CPZ	8532
ANO5	203859
ZNF204P	7754
ATRNL1	26033
MEIS3P1	4213
NRXN3	9369

SCARA5	286133
CFD	1675
MYL9	10398
FAM124A	220108
GABRB3	2562
KIAA1614	57710
EN2	2020
P2RX1	5023
FHIT	2272
AGMAT	79814
CAP2	10486
SYNC	81493
CLVS2	134829
LRCH2	57631
UBE2C	11065
TK1	7083
GRIN3A	116443
PENK	5179
FGFR2	2263
SSTR1	6751
AOC3	8639
CEP55	55165
NTM	50863
BNC2	54796
GABRD	2563
SMOC1	64093
ZFP36L1	677
FAM83B	222584
ATP1A2	477
KIF14	9928
SLC27A2	11001
DNAH8	1769
ENPP3	5169
CYP3A5	1577
FLNC	2318
GATA5	140628
ITGB1BP2	26548
CD248	57124
TRIB1	10221
TSPAN2	10100
TPO	7173
TBX10	347853
NRP2	8828
DNASE1L2	1775
RSPH1	89765
GJB3	2707
ALDH3A1	218
ATAD3C	219293
PPFIA2	8499
HOXD8	3234
CELSR3	1951
SLC23A3	151295
TSPAN1	10103
BCL2	596
PLXNA4	91584
NCCRP1	342897
NUDT10	170685
EXO1	9156

PDLIM1	9124
ZNF560	147741
FLNA	2316
COL9A2	1298
CLIC6	54102
SGCA	6442
HAPLN2	60484
ETV5	2119
MAP1B	4131
DGCR5	26220
NSUN5P2	260294
ITGB4	3691
PRLR	5618
TOP2A	7153
ANKRD30/	91074
MNX1	3110
C9orf43	257169
TCEAL2	140597
CES1	1066
ITPR1	3708
CBX2	84733
KIF2C	11004
WSCD2	9671
RBPMS	11030
SLITRK2	84631
PYGL	5836
LAMB3	3914
TPM2	7169
KRT7	3855
ISX	91464
PTP4A3	11156
NKAIN1	79570
FEV	54738
PCYT1B	9468
NELL2	4753
NID1	4811
TAGLN	6876
ZNF750	79755
FOXL2	668
DYSF	8291
LGALS4	3960
SLC38A5	92745
INMT	11185
ITGA5	3678
ANKRD35	148741
ARMC3	219681
ATP1B1	481
ID1	3397
PCDH10	57575
MGAT5B	146664
SNHG9	735301
VSNL1	7447
MIPEP	4285
LDB3	11155
PRIMA1	145270
HSD17B13	345275
INPP5D	3635
C4orf48	401115

MATN2	4147
DKK3	27122
CATSPER2	117155
PPEF1	5475
DCST2	127579
LGALS1	3956
IER5	51278
AFF2	2334
TMOD1	7111
IL33	90865
SPARCL1	8404
CPNE6	9362
GPX3	2878
SERPINB5	5268
APLN	8862
AURKA	6790
PCDHGB1	56104
TRPC4	7223
GAS1	2619
MAPK8IP2	23542
SERPINB1	1992
FHL1	2273
HEY2	23493
TRIM31	11074
KANK4	163782
WIF1	11197
TBX1	6899
SLC24A2	25769
ITGB6	3694
GAD1	2571
HCN2	610
ADM2	79924
NEXN	91624
ARMCX1	51309
OLFML2A	169611
AQP3	360
KCNB1	3745
POLQ	10721
MYO6	4646
HOXA7	3204
KRT19	3880
CRYAB	1410
SLC16A8	23539
CIT	11113
SS18L2	51188
SSTR2	6752
RNFT2	84900
ESPL1	9700
CAMK1G	57172
CLEC3B	7123
CKAP2L	150468
APOBEC3F	200316
PHLDA1	22822
RAD51	5888
DNAJC12	56521
ACTA2	59
RCAN2	10231
CSDC2	27254

FOXS1	2307
IL1RN	3557
PLAC9	219348
GIPC2	54810
AMPH	273
LGI3	203190
HES6	55502
SLC4A11	83959
HSD11B1	3290
MDH1B	130752
KCNH8	131096
FAM71F2	346653
SLC14A1	6563
AOC2	314
SYCE1L	1E+08
STK33	65975
HRH2	3274
SERPINA1[illegible]	256394
KCNAB3	9196
TP63	8626
GJA3	2700
PTCHD1	139411
ASF1B	55723
NAALADL[illegible]	254827
GRPR	2925
WFDC1	58189
RPL36A	6173
DLK2	65989
C3orf35	339883
TMC5	79838
PACSIN1	29993
PCDHA1	56147
C1QTNF9[illegible]	387911
SPATA18	132671
NUDT8	254552
BCHE	590
CACNB1	782
REEP1	65055
MUC6	4588
SGPP2	130367
DSC3	1825
ENTPD5	957
CPNE7	27132
AADAT	51166
EGLN3	112399
MADCAM[illegible]	8174
TMEM191[illegible]	84222
CCDC80	151887
HSPB6	126393
MCCC2	64087
COL6A2	1292
TP53AIP1	63970
SP5	389058
THBS4	7060
PKMYT1	9088
CLIC3	9022
ELF5	2001
CHIT1	1118

CCND2	894
KRT5	3852
CA4	762
ABCC3	8714
RELN	5649
IL20RB	53833
CRHBP	1393
SEMA5A	9037
EDIL3	10085
RAB3B	5865
PABPC1L	80336
CAMK1D	57118
FCGBP	8857
SLC2A5	6518
LGALS3	3958
DPP6	1804
DGKG	1608
DNASE2B	58511
LRP1B	53353
KIAA1210	57481
ADRA1D	146
FOXP2	93986
CXCL11	6373
CST6	1474
ITGAX	3687
F10	2159
ITGA7	3679
FBXO2	26232
ACTG2	72
SV2C	22987
KLK14	43847
CPA6	57094
NDP	4693
CHRNA5	1138
TMEM40	55287
GSTM2	2946
ZEB2	9839
HUNK	30811
LRFN5	145581
HLA-DMB	3109
CELF5	60680
TMEM145	284339
SFRP4	6424
OLR1	4973
SRMS	6725
B3GALT2	8707
SFTPA2	729238
CNTN1	1272
ZWINT	11130
TTR	7276
BVES	11149
SYT9	143425
RND3	390
SDC1	6382
RGS9	8787
SERPINF1	5176
FOXF1	2294
IP6K3	117283

PPM1E	22843
KLK12	43849
RNF165	494470
PILRB	29990
COMP	1311
PCDH9	5101
MASP1	5648
ASTN1	460
PCDHGA5	56110
NTRK1	4914
RAB26	25837
FGF2	2247
LILRB4	11006
RARRES2	5919
SLC17A4	10050
ADRA2A	150
TMEM52	339456
EMILIN1	11117
CDKN2A	1029
RSPH4A	345895
NRK	203447
BANK1	55024
HMGCLL1	54511
SULT4A1	25830
VSTM2L	128434
TBX5	6910
DAB1	1600
DNAJC15	29103
FAT4	79633
PCDHB16	57717
SIX2	10736
SMS	6611
GPM6A	2823
TRIM29	23650
SYCP2L	221711
C1orf53	388722
PLN	5350
CCDC3	83643
COL6A1	1291
NTN4	59277
SLC8A2	6543
FGF7	2252
FAM181B	220382
CHP2	63928
CTLA4	1493
TDRD6	221400
DPT	1805
HCG27	253018
MIAT	440823
SCNN1A	6337
CSPG5	10675
TCEAL7	56849
ANXA1	301
MYO1G	64005
FASN	2194
HOXD11	3237
RFX6	222546
SCGB1A1	7356

RSPO3	84870
TBX4	9496
S1PR5	53637
MYB	4602
PNMA2	10687
PCSK2	5126
BDNF	627
CRYBA2	1412
ACOT11	26027
DPY19L2P	442523
OGN	4969
SLC26A5	375611
KCNJ3	3760
WNT2B	7482
GGT6	124975
PTPRR	5801
TGFB3	7043
APOE	348
PRDM6	93166
UPK1A	11045
SLC6A17	388662
MYH11	4629
LSAMP	4045
THSD4	79875
ADPRHL1	113622
FADS2	9415
ISG15	9636
SORCS2	57537
PYGM	5837
ZNF556	80032
ADH1B	125
MON1B	22879
HSPB7	27129
SPON2	10417
SPZ1	84654
ADCYAP1F	117
PI16	221476
NGFR	4804
SLC2A4	6517
SV2B	9899
MLC1	23209
MET	4233
CAMK4	814
LYPD6B	130576
SCUBE3	222663
TMPRSS4	56649
PDE5A	8654
C15orf48	84419
NEAT1	283131
TUBB3	10381
DMKN	93099
GPR87	53836
ASXL3	80816
ATCAY	85300
PPP1R1A	5502
VIT	5212
CALML3	810
PCDHB11	56125

ETS2	2114
MPZ	4359
SEC14L5	9717
IL2RA	3559
SMTNL2	342527
CBR3	874
KRT4	3851
CHI3L1	1116
INHA	3623
C4A	720
NEGR1	257194
KRT23	25984
CRYGS	1427
ZNF536	9745
VAX2	25806
NOS1	4842
FXYD1	5348
CLNK	116449
TOX3	27324
SKAP1	8631
NTRK2	4915
C12orf60	144608
MCM10	55388
LEAP2	116842
RPRM	56475
PCDHB8	56128
NPY6R	4888
PCP4	5121
KLKP1	606293
ZFHX4	79776
MACC1	346389
SNORD17	692086
RAD54L	8438
LTBP4	8425
IGDCC4	57722
PRAME	23532
NTN1	9423
BMP6	654
NEURL3	93082
ECT2L	345930
ACE2	59272
SLC35F3	148641
CHD5	26038
CDC20B	166979
OIP5	11339
EME1	146956
TMEM45B	120224
NRG1	3084
TNS4	84951
HNF1A	6927
WDR93	56964
DLGAP2	9228
NCKAP5	344148
CENPI	2491
CYP2W1	54905
MYT1	4661
SPINK8	646424
NRXN1	9378

CCL22	6367
MND1	84057
FAM189A1	23359
ZYG11A	440590
CPXM1	56265
ALX4	60529
EYA1	2138
E2F2	1870
VGLL1	51442
TMEM63C	57156
ZFP92	139735
L1CAM	3897
LRRC26	389816
TACR2	6865
PRSS12	8492
HBA2	3040
DDC	1644
NCALD	83988
ANXA8	653145
PCDHB2	56133
S100B	6285
BDKRB2	624
KRT14	3861
AREG	374
C7	730
CLCA4	22802
PAX5	5079
LRRN1	57633
RPLP0P2	113157
FBLN1	2192
FOXF2	2295
NTRK3	4916
IL5RA	3568
ST18	9705
CLDN8	9073
FOSL1	8061
LRRC7	57554
SCN7A	6332
KIF15	56992
STXBP5L	9515
C16orf89	146556
WFIKKN1	117166
SLC9A3	6550
TRPM8	79054
CDK1	983
SCGB2A1	4246
LHX4	89884
HSPB2	3316
NTF4	4909
RASGRF1	5923
VTCN1	79679
WDR62	284403
AGR3	155465
IL1RAPL1	11141
WNT3A	89780
SERHL	94009
SCRG1	11341
COX7A1	1346

CDCA2	157313
CYP27A1	1593
NHSL2	340527
HS6ST3	266722
SUCNR1	56670
TNFRSF18	8784
TRPM2	7226
RPE65	6121
PCBP3	54039
YBX2	51087
DES	1674
NUSAP1	51203
ASB9	140462
ANLN	54443
CBS	875
FLRT3	23767
HS3ST4	9951
KCNK3	3777
HSPA1A	3303
DCN	1634
RET	5979
PLA2G2A	5320
FN1	2335
CST4	1472
COL14A1	7373
COL28A1	340267
MUC4	4585
ESPNL	339768
FAP	2191
PEX10	5192
CXCL10	3627
GCNT3	9245
SAMD13	148418
NPY	4852
CST1	1469
LRRC2	79442
EDN3	1908
KLHL30	377007
F5	2153
PLP1	5354
FOLH1B	219595
HAS2	3037
PROC	5624
TYRP1	7306
CD38	952
ENPP6	133121
PRR4	11272
ART4	420
KRT15	3866
SRL	6345
UHRF1	29128
PABPC1L2	645974
IFI6	2537
DSG3	1830
DPY19L2	283417
CEACAM6	4680
ACAN	176
EGF	1950

BCL11A	53335
SLC44A5	204962
PCDHGB2	56103
HS3ST2	9956
RDH12	145226
MMP9	4318
CRIP3	401262
EDAR	10913
MYH6	4624
CCK	885
GPRC5D	55507
KCNC2	3747
CD163L1	283316
SLC4A4	8671
AGTR1	185
INSM1	3642
PCDHB10	56126
PCOTH	542767
GPRC5A	9052
KCNN2	3781
MMP10	4319
LRRN3	54674
MIR17HG	407975
IL1RL2	8808
RHCG	51458
PRSS16	10279
DKK1	22943
OLFM4	10562
SOX14	8403
GOLGA8B	440270
C4orf19	55286
ERN2	10595
IL31RA	133396
RSPO2	340419
IGFN1	91156
KCNK15	60598
ZFPM2	23414
CEL	1056
ANO4	121601
SGK1	6446
GDF7	151449
DUSP5	1847
CNKSR2	22866
CLSPN	63967
SYP	6855
CXCL17	284340
PROM1	8842
ALOX15	246
PMP2	5375
TDO2	6999
GJB2	2706
EPHA6	285220
GOLGA8A	23015
ABO	28
MME	4311
TXLNB	167838
SCIN	85477
B3GNT3	10331

CGA	1081
CDH22	64405
POPDC3	64208
SULT1C2	6819
ITGA8	8516
PPP1R14C	81706
BMPER	168667
KCNMB2	10242
TMEM217	221468
VTN	7448
CD177	57126
TMEM26	219623
SLC38A11	151258
SNORA74I	677841
ANGPTL4	51129
CHRDL2	25884
CPB1	1360
SALL1	6299
CDH7	1005
NRCAM	4897
ELFN2	114794
CAPNS2	84290
MFAP5	8076
CXCL9	4283
CXCL14	9547
CASQ2	845
CARTPT	9607
BMP7	655
KRT6A	3853
C1QTNF3	114899
C2CD4A	145741
PABPC1L2	340529
PCDHA10	56139
ELOVL2	54898
ACER1	125981
HP	3240
EDDM3A	10876
SCARNA7	677767
SLC5A8	160728
SH2D1A	4068
FOXI1	2299
SCARNA2:	677763
UNC80	285175
NR4A3	8013
LIX1	167410
RIMS4	140730
MYH15	22989
EEF1A2	1917
SERPINA3	12
MUC13	56667
GC	2638
RBP4	5950
FAM3D	131177
SGCG	6445
CHST9	83539
S100A9	6280
PGC	5225
PCDHB3	56132

LIPF	8513
S100A2	6273
TMED6	146456
TGM4	7047
VEPH1	79674
PLA2G2D	26279
SOCS3	9021
DLX6	1750
CEACAM2	388550
UNC13A	23025
KRTAP13-	337959
GLP1R	2740
REG4	83998
FOXD3	27022
ETV1	2115
HPGD	3248
ABCC11	85320
OR51E1	143503
FCRL3	115352
FGFBP1	9982
EOMES	8320
CCDC141	285025
TIMP4	7079
BMP5	653
SNX31	169166
ZNF385B	151126
DAPL1	92196
CES3	23491
PCDHA4	56144
COL9A1	1297
SELE	6401
GABRP	2568
DIRAS2	54769
SEMA3D	223117
LRRC31	79782
FGB	2244
CLSTN2	64084
ECEL1	9427
NEFL	4747
DEFB1	1672
MUCL1	118430
KRT17	3872
RLN2	6019
FNDC1	84624
OXGR1	27199
CEACAM5	1048
CXCL13	10563
DPYS	1807
PCDHA11	56138
CTNNA2	1496
EML6	400954
NKX3-2	579
ARX	170302
PTPRT	11122
DEFA5	1670
POTEG	404785
MUC2	4583
XIRP1	165904

TCAP	8557
NEFH	4744
TNNC1	7134
COL11A1	1301
C12orf56	115749
SLC3A1	6519
MYH3	4621
PCDHA6	56142
SI	6476
CYP24A1	1591
H19	283120
PLA2G4D	283748
CPS1	1373
HAPLN1	1404
CKM	1158
UGT2B15	7366
GLDC	2731
SIK1	150094
CASQ1	844
APOBEC2	10930
CLEC3A	10143
SERPINB3	6317
GRM7	2917
VIP	7432
SERPINA4	5267
DEFA6	1671
GCG	2641
PNMA5	114824
SAA2	6289
CR2	1380
GFAP	2670
NPPC	4880
PON1	5444
SERPINB11	89778
HSPB3	8988
MYL3	4634
WFDC12	128488
SCARNA10	692148
TNNT3	7140
PAH	5053
CRISP3	10321
NKX2-1	7080
