# Supplementary material for: Integrative Analysis of Bulk RNA-Seq and Single-Cell RNA-Seq Unveils the Characteristics of the Immune Microenvironment and Prognosis Signature in Prostate Cancer
Source: J Oncol. 2022 Jul 19;2022:6768139. doi: 10.1155/2022/6768139 (PMC9325591; doi:10.1155/2022/6768139)
Supplement: Supplementary Materials — Figure S1. Workflow of the analysis. Figure S2. Validation of the risk score model using the GSE54460 dataset. A. Patients with prostate cancer (PRAD) in the GSE54460 cohort are listed in ascending order of risk score. B. Progression-free interval (PFI) distribution versus the risk score of each patient in the GSE54460 cohort. C. Kaplan–Meier (KM) curves of patients with different risk levels in the GSE54460 validation set. D. Receiver Operating Characteristic (ROC) curve analysis for 1-, 3- and 5-year PFI using the clinical information of patients of the GSE54460 validation dataset. Figure S3. Validation of the risk score model using the GSE46602 dataset. A. Patients with prostate cancer (PRAD) in the GSE46602 cohort are listed in ascending order of risk score. B. Progression-free interval (PFI) distribution versus the risk score of each patient in the GSE46602 cohort. C. Kaplan–Meier (KM) curves of patients with different risk levels in the GSE46602 validation dataset. D. Receiver Operating Characteristic (ROC) curve analysis for 1-, 3- and 5-year PFI using the clinical information of patients of the GSE46602 validation dataset. Figure S4. Validation of the risk score model using the GSE70768 dataset. A. Patients with prostate cancer (PRAD) in the GSE70768 cohort are listed in ascending order of risk score. B. Progression-free interval (PFI) distribution versus the risk score of each patient in the GSE70768 cohort. C. Kaplan–Meier (KM) curves of patients with different risk levels in the GSE70768 validation dataset. D. Receiver Operating Characteristic (ROC) curve analysis for 1-, 3- and 5-year PFI using the clinical information of patients of the GSE70768 validation dataset. Figure S5. Validation of the risk score model using the GSE70769 dataset. A. Patients with prostate cancer (PRAD) in the GSE70769 validation dataset are listed in ascending order of risk score. B. Progression-free interval (PFI) distribution versus the risk score of each patient in the GSE707 [file 6768139.f1.zip › 6768139.f1/Table S15.pdf]

SNHG8
SYCE1L
GJC1
EDIL3
TSPAN1
MBNL2
TRIB1
RCAN2
ABCC4
SPEG
CRISP3
MYL9
WFDC2
SPON2
CAP2
SORBS1
CDC42EP3
PDLIM5
CAMKK2
CFL2
CPLX1
FERMT2
TMSB15A
RBPMS
DNAJB4
ADAMTS5
EMILIN1
PTPRT
LDB3
PTP4A3
FAM107A
WIF1
IRAK3
CHRNA5
RNF157
C1QTNF1
FBXO17
ACSM1
MRGPRF
CLU
TMEM45B
HSPB6
CNN1
COL2A1
COL6A1
COL6A2
COL9A2
FAM3D
COX7A1
CRABP2
CRHBP
S100A16
TMEM37
CRYAB
OR51E1
TSPAN19
C12orf60
CSPG4

CSRP1
CST1
CST4
ADRA2A
ZNF385B
SLC38A11
CCDC80
CYBA
IRX2
AGR3
CYP2J2
DCN
ACE
OLFML2A
ARX
SYNPO2
DMD
ACAN
DMPK
DNAH5
DNAH8
DTNA
DUSP5
AGTR1
EDNRA
EDNRB
EFNB1
ANO6
EPHA2
SERPINB1
RPL22L1
EMP3
TMEM184A
ERG
ETS2
ETV1
ETV5
MECOM
F10
FABP5
FBLN1
PLAC9
FASN
UNC5B
FAM162B
FGF2
FGF7
FHIT
FHL1
PHLDA1
MON1B
FOXF1
PALLD
FOXS1
AKR1B1
FLNA
SYNM
FN1

FOLH1
HEY2
DAAM2
AMACR
SLC7A11
IFI6
MSRB3
NUDT8
NAALADL2
GABRD
FRMD3
C2orf72
LMOD1
METTL7A
PARM1
CLIC4
KANK2
CCDC69
TES
GAS6
HSPB8
GCNT1
MYOF
GJA1
GPR160
GJB1
PDE7B
DKK3
FILIP1
RBMS3
APOBEC3C
GMDS
GNG11
GPM6A
SLCO3A1
GPM6B
ANGPT1
GPX3
DNAJC15
GSTM3
GSTP1
EFEMP2
ANXA1
ANXA2
EHD2
ANXA6
HLA-DMB
HLF
ACACB
HOXB2
HOXB3
HOXB4
HOXB7
HOXD8
HPGD
HPN
HRH2
HSF4

HSPA6
HSPB2
DNAJB1
LUZP2
ID1
ID3
ID4
NHSL2
APOC1
NEK5
APOE
RBPMS2
AQP3
ILK
INPP5D
ITGA5
ITGA7
ITGA9
ITGB4
ITPR1
AREG
KCNC2
KCNJ8
KCNMB1
KCNN2
ARL4D
KRT7
KRT15
KRT17
C12orf75
RHOB
KRT19
BEND4
SAMD5
LRRC26
RND3
LGALS1
LGALS3
C4orf48
LPL
LSAMP
EPCAM
MAP1A
MAP1B
MATN2
MCC
MEIS2
MET
MIPEP
MME
CD200
KLK12
MXRA7
ASS1
MYH11
MYLK
MYO6
PPP1R12B

ATP1A2
ATP1B1
NID1
NOTCH1
NPY
NT5E
NTRK2
NTRK3
DDR2
ATP2B4
OGN
OTX1
PODXL2
PCA3
PLLP
ANGPTL4
SLC45A2
AADAT
SS18L2
PCP4
IER5
GOLM1
RASL12
ARMCX1
PRR16
PDE2A
ISYNA1
PDK4
GPRC5B
SERPINF1
PEX10
PGC
PGF
PGM5
PIK3R1
PLA2G2A
FXYD1
PLN
PLP2
PLS3
FXYD6
FGFRL1
A4GALT
NEURL1B
DDIT4
FBLIM1
TRPM4
ASPN
ARHGEF38
LPCAT2
BANK1
ANO1
TMEM100
TRIM36
LIMS2
PKIB
CENPN
WWC3

PRKG1
TDRD1
DNAJC12
TCEAL7
CAMK1D
CD248
RALGAPA2
PTGS2
NDRG2
SH3RF1
QSOX1
CACHD1
HES4
WFDC1
PYCR1
DNASE2B
RAB3B
RAC3
ACTA2
RAP1GAP
RARG
RARRES2
NTN4
PLEKHA2
SCPEP1
RBP1
RET
RGS10
RLN2
RNASE1
RPL36A
RRAS
S100A6
SCNN1A
BICD1
BIK
SELE
MCCC2
TINAGL1
RAB17
HIF3A
SGCA
SGCB
SGK1
PHGR1
SIM2
MARCKSL1
SMTN
BMP7
SLCO2A1
SLIT3
SLPI
SNAI2
SMS
SNCG
ZFP36L1
SVIL
TACC1

TAGLN
KLF5
TBX2
TCN2
TEAD1
TFF3
TGFB1I1
TGFBR3
THBS4
TIMP3
CLEC3B
TNS1
TPM1
TPM2
ACTG2
TRIP6
TUBB2A
SFTPA2
C7
COL14A1
VCL
CA2
CA4
CACNA1D
KCNAB1
TRPM8
TMC5
ZDHHC11
THSD4
CALD1
CSRNP3
NR4A3
EFHD1
PDGFD
ITIH5
SSPN
COLEC12
OR51E2
COL21A1
ANP32E
CAMK2G
DYSF
FZD7
TCF7L1
SOX7
FAM107B
CCDC3
RAB34
PIP5K1B
SOX14
SPARCL1
LRRIQ1
LTBP4
C15orf48
CASQ2
TUBB6
AFAP1L2
ZNF577

C19orf48
PPFIA2
RERG
SLC43A1
ITGA8
PAQR8
ABCC11
SYDE1
SCIN
CAV1
CAV2
AOC3
PDE5A
SLC4A4
CBR3
NRP2
IER3
CCND2
SERPINB11
ZNF30
TSPAN18
SOCS3
SEMA5A
GPRC5A
CLDN8
IL33
RGN
ANKRD30A
PDLIM1
NEXN
REPS2
GLYATL1
PNMA1
PDLIM7
FADS2
TP53INP1
PPP1R14A
ABCG2
CHST2
GDF15
ENTPD5
CD40
ISG15
FEZ1
LAPTM4A
STARD8
ZEB2
