# Supplementary material for: Integrative Analysis of Bulk RNA-Seq and Single-Cell RNA-Seq Unveils the Characteristics of the Immune Microenvironment and Prognosis Signature in Prostate Cancer
Source: J Oncol. 2022 Jul 19;2022:6768139. doi: 10.1155/2022/6768139 (PMC9325591; doi:10.1155/2022/6768139)
Supplement: Supplementary Materials — Figure S1. Workflow of the analysis. Figure S2. Validation of the risk score model using the GSE54460 dataset. A. Patients with prostate cancer (PRAD) in the GSE54460 cohort are listed in ascending order of risk score. B. Progression-free interval (PFI) distribution versus the risk score of each patient in the GSE54460 cohort. C. Kaplan–Meier (KM) curves of patients with different risk levels in the GSE54460 validation set. D. Receiver Operating Characteristic (ROC) curve analysis for 1-, 3- and 5-year PFI using the clinical information of patients of the GSE54460 validation dataset. Figure S3. Validation of the risk score model using the GSE46602 dataset. A. Patients with prostate cancer (PRAD) in the GSE46602 cohort are listed in ascending order of risk score. B. Progression-free interval (PFI) distribution versus the risk score of each patient in the GSE46602 cohort. C. Kaplan–Meier (KM) curves of patients with different risk levels in the GSE46602 validation dataset. D. Receiver Operating Characteristic (ROC) curve analysis for 1-, 3- and 5-year PFI using the clinical information of patients of the GSE46602 validation dataset. Figure S4. Validation of the risk score model using the GSE70768 dataset. A. Patients with prostate cancer (PRAD) in the GSE70768 cohort are listed in ascending order of risk score. B. Progression-free interval (PFI) distribution versus the risk score of each patient in the GSE70768 cohort. C. Kaplan–Meier (KM) curves of patients with different risk levels in the GSE70768 validation dataset. D. Receiver Operating Characteristic (ROC) curve analysis for 1-, 3- and 5-year PFI using the clinical information of patients of the GSE70768 validation dataset. Figure S5. Validation of the risk score model using the GSE70769 dataset. A. Patients with prostate cancer (PRAD) in the GSE70769 validation dataset are listed in ascending order of risk score. B. Progression-free interval (PFI) distribution versus the risk score of each patient in the GSE707 [file 6768139.f1.zip › 6768139.f1/Table S2.pdf]

ONTOLOGY	ID	Description	GeneRatio	pvalue	p.adjust	Count
BP	GO:0006936	muscle contraction	86/1600	9.38E-20	5.29E-16	86
BP	GO:0003012	muscle system process	99/1600	7.28E-19	2.05E-15	99
BP	GO:0060485	mesenchyme development	65/1600	1.42E-13	2.68E-10	65
BP	GO:0003018	vascular process in circulatory system	57/1600	1.40E-12	1.66E-09	57
BP	GO:0048568	embryonic organ development	80/1600	1.47E-12	1.66E-09	80
BP	GO:1903522	regulation of blood circulation	63/1600	2.41E-12	2.06E-09	63
BP	GO:0060537	muscle tissue development	74/1600	2.64E-12	2.06E-09	74
BP	GO:0001655	urogenital system development	67/1600	2.91E-12	2.06E-09	67
BP	GO:0014706	striated muscle tissue development	70/1600	1.04E-11	6.52E-09	70
BP	GO:0098742	cell-cell adhesion via plasma-membrane adhesion molecules	59/1600	1.50E-11	7.72E-09	59
BP	GO:0048762	mesenchymal cell differentiation	53/1600	1.51E-11	7.72E-09	53
BP	GO:0042692	muscle cell differentiation	71/1600	1.67E-11	7.84E-09	71
BP	GO:0007156	homophilic cell adhesion via plasma membrane adhesion molecules	43/1600	2.39E-11	9.75E-09	43
BP	GO:0051146	striated muscle cell differentiation	58/1600	2.47E-11	9.75E-09	58
BP	GO:0045229	external encapsulating structure organization	75/1600	2.59E-11	9.75E-09	75
BP	GO:0048562	embryonic organ morphogenesis	59/1600	2.81E-11	9.91E-09	59
BP	GO:0007409	axonogenesis	83/1600	3.01E-11	1.00E-08	83
BP	GO:0060047	heart contraction	59/1600	3.82E-11	1.20E-08	59
BP	GO:0072001	renal system development	60/1600	4.97E-11	1.48E-08	60
BP	GO:0003015	heart process	60/1600	5.76E-11	1.63E-08	60
BP	GO:0030198	extracellular matrix organization	73/1600	1.21E-10	3.26E-08	73
BP	GO:0007517	muscle organ development	63/1600	1.31E-10	3.36E-08	63
BP	GO:0043062	extracellular structure organization	73/1600	1.37E-10	3.36E-08	73
BP	GO:0008016	regulation of heart contraction	53/1600	1.56E-10	3.67E-08	53
BP	GO:0030900	forebrain development	68/1600	1.71E-10	3.86E-08	68
BP	GO:0031589	cell-substrate adhesion	68/1600	2.20E-10	4.77E-08	68
BP	GO:0014031	mesenchymal cell development	27/1600	6.09E-10	1.27E-07	27
BP	GO:0023061	signal release	81/1600	8.83E-10	1.78E-07	81
BP	GO:0001822	kidney development	56/1600	1.09E-09	2.12E-07	56
BP	GO:0006813	potassium ion transport	49/1600	1.80E-09	3.36E-07	49
BP	GO:0006941	striated muscle contraction	41/1600	1.85E-09	3.36E-07	41
BP	GO:0007411	axon guidance	55/1600	2.02E-09	3.54E-07	55
BP	GO:0007389	pattern specification process	74/1600	2.07E-09	3.54E-07	74
BP	GO:0097485	neuron projection guidance	55/1600	2.31E-09	3.84E-07	55
BP	GO:0048565	digestive tract development	33/1600	2.46E-09	3.94E-07	33
BP	GO:1904062	regulation of cation transmembrane transport	63/1600	2.51E-09	3.94E-07	63
BP	GO:0090596	sensory organ morphogenesis	50/1600	3.61E-09	5.51E-07	50
BP	GO:0034329	cell junction assembly	73/1600	4.39E-09	6.52E-07	73
BP	GO:0071805	potassium ion transmembrane transport	45/1600	6.96E-09	1.01E-06	45
BP	GO:0008217	regulation of blood pressure	40/1600	7.35E-09	1.04E-06	40
BP	GO:0001755	neural crest cell migration	20/1600	9.23E-09	1.27E-06	20
BP	GO:0006939	smooth muscle contraction	28/1600	9.61E-09	1.29E-06	28
BP	GO:0006816	calcium ion transport	70/1600	1.06E-08	1.39E-06	70
BP	GO:0048738	cardiac muscle tissue development	44/1600	1.14E-08	1.46E-06	44
BP	GO:0035296	regulation of tube diameter	32/1600	1.47E-08	1.80E-06	32
BP	GO:0097746	blood vessel diameter maintenance	32/1600	1.47E-08	1.80E-06	32
BP	GO:0014033	neural crest cell differentiation	26/1600	1.58E-08	1.90E-06	26
BP	GO:0055123	digestive system development	33/1600	1.62E-08	1.90E-06	33
BP	GO:0035150	regulation of tube size	32/1600	1.80E-08	2.08E-06	32
BP	GO:0042310	vasoconstriction	24/1600	1.89E-08	2.14E-06	24
BP	GO:0009914	hormone transport	57/1600	2.41E-08	2.67E-06	57
BP	GO:0014032	neural crest cell development	24/1600	2.49E-08	2.70E-06	24
BP	GO:0001501	skeletal system development	78/1600	2.85E-08	3.03E-06	78
BP	GO:0022612	gland morphogenesis	29/1600	2.90E-08	3.03E-06	29
BP	GO:0010975	regulation of neuron projection development	71/1600	3.10E-08	3.18E-06	71
BP	GO:0021700	developmental maturation	51/1600	3.35E-08	3.38E-06	51
BP	GO:0048732	gland development	69/1600	3.64E-08	3.60E-06	69
BP	GO:0043266	regulation of potassium ion transport	27/1600	4.09E-08	3.91E-06	27
BP	GO:0043583	ear development	42/1600	4.09E-08	3.91E-06	42
BP	GO:0030072	peptide hormone secretion	48/1600	4.29E-08	4.03E-06	48
BP	GO:0046879	hormone secretion	55/1600	4.73E-08	4.37E-06	55
BP	GO:0055001	muscle cell development	37/1600	5.45E-08	4.91E-06	37
BP	GO:0048864	stem cell development	24/1600	5.48E-08	4.91E-06	24
BP	GO:0030001	metal ion transport	77/1600	6.09E-08	5.37E-06	77
BP	GO:0050808	synapse organization	68/1600	7.41E-08	6.42E-06	68
BP	GO:0061337	cardiac conduction	34/1600	7.51E-08	6.42E-06	34
BP	GO:1901379	regulation of potassium ion transmembrane transport	24/1600	1.16E-07	9.64E-06	24
BP	GO:0003002	regionalization	57/1600	1.16E-07	9.64E-06	57
BP	GO:0048469	cell maturation	36/1600	1.24E-07	1.01E-05	36
BP	GO:0006937	regulation of muscle contraction	35/1600	1.73E-07	1.40E-05	35
BP	GO:0060048	cardiac muscle contraction	31/1600	2.40E-07	1.91E-05	31
BP	GO:0060284	regulation of cell development	75/1600	2.45E-07	1.91E-05	75
BP	GO:0001704	formation of primary germ layer	28/1600	2.50E-07	1.91E-05	28
BP	GO:1903429	regulation of cell maturation	11/1600	2.51E-07	1.91E-05	11
BP	GO:0050886	endocrine process	22/1600	3.15E-07	2.37E-05	22
BP	GO:0018108	peptidyl-tyrosine phosphorylation	61/1600	3.26E-07	2.42E-05	61
BP	GO:0042471	ear morphogenesis	27/1600	3.42E-07	2.51E-05	27
BP	GO:0007416	synapse assembly	36/1600	3.65E-07	2.61E-05	36
BP	GO:0019229	regulation of vasoconstriction	19/1600	3.66E-07	2.61E-05	19
BP	GO:0071695	anatomical structure maturation	42/1600	3.76E-07	2.65E-05	42
BP	GO:0035637	multicellular organismal signaling	40/1600	3.84E-07	2.68E-05	40
BP	GO:0048608	reproductive structure development	65/1600	4.05E-07	2.79E-05	65
BP	GO:0018212	peptidyl-tyrosine modification	61/1600	4.34E-07	2.95E-05	61
BP	GO:0050807	regulation of synapse organization	40/1600	4.39E-07	2.95E-05	40
BP	GO:0090257	regulation of muscle system process	45/1600	4.74E-07	3.14E-05	45
BP	GO:0061458	reproductive system development	65/1600	5.31E-07	3.48E-05	65
BP	GO:0048730	epidermis morphogenesis	13/1600	5.39E-07	3.50E-05	13
BP	GO:0048839	inner ear development	36/1600	5.66E-07	3.59E-05	36
BP	GO:0048483	autonomic nervous system development	16/1600	5.67E-07	3.59E-05	16
BP	GO:0033002	muscle cell proliferation	42/1600	6.26E-07	3.93E-05	42
BP	GO:0070252	actin-mediated cell contraction	28/1600	6.39E-07	3.96E-05	28
BP	GO:0031069	hair follicle morphogenesis	12/1600	6.93E-07	4.25E-05	12
BP	GO:0050731	positive regulation of peptidyl-tyrosine phosphorylation	38/1600	7.59E-07	4.61E-05	38
BP	GO:0001667	ameboidal-type cell migration	72/1600	7.80E-07	4.68E-05	72
BP	GO:0001763	morphogenesis of a branching structure	37/1600	8.13E-07	4.83E-05	37
BP	GO:0042391	regulation of membrane potential	67/1600	8.80E-07	5.17E-05	67
BP	GO:0090066	regulation of anatomical structure size	74/1600	9.46E-07	5.50E-05	74
BP	GO:1903779	regulation of cardiac conduction	18/1600	1.04E-06	6.00E-05	18
BP	GO:0055002	striated muscle cell development	25/1600	1.15E-06	6.55E-05	25
BP	GO:0048704	embryonic skeletal system morphogenesis	23/1600	1.17E-06	6.55E-05	23
BP	GO:0007611	learning or memory	43/1600	1.18E-06	6.55E-05	43
BP	GO:0032409	regulation of transporter activity	49/1600	1.18E-06	6.55E-05	49
BP	GO:0001764	neuron migration	31/1600	1.26E-06	6.88E-05	31
BP	GO:0048706	embryonic skeletal system development	27/1600	1.27E-06	6.88E-05	27

BP	GO:0048736	appendage development	34/1600	1.29E-06	6.88E-05	34
BP	GO:0060173	limb development	34/1600	1.29E-06	6.88E-05	34
BP	GO:0022898	regulation of transmembrane transporter activity	47/1600	1.34E-06	7.05E-05	47
BP	GO:0046883	regulation of hormone secretion	46/1600	1.35E-06	7.05E-05	46
BP	GO:0050803	regulation of synapse structure or activity	40/1600	1.58E-06	8.18E-05	40
BP	GO:0051960	regulation of nervous system development	65/1600	1.76E-06	9.05E-05	65
BP	GO:1905330	regulation of morphogenesis of an epithelium	18/1600	1.79E-06	9.11E-05	18
BP	GO:0021537	telencephalon development	42/1600	1.86E-06	9.35E-05	42
BP	GO:0010959	regulation of metal ion transport	44/1600	1.88E-06	9.40E-05	44
BP	GO:0140014	mitotic nuclear division	50/1600	1.95E-06	9.64E-05	50
BP	GO:0021543	pallium development	32/1600	2.21E-06	0.000109	32
BP	GO:0042445	hormone metabolic process	40/1600	2.56E-06	0.000124	40
BP	GO:0050673	epithelial cell proliferation	65/1600	2.88E-06	0.000137	65
BP	GO:2000811	negative regulation of anoikis	9/1600	2.88E-06	0.000137	9
BP	GO:0015711	organic anion transport	59/1600	2.88E-06	0.000137	59
BP	GO:0048863	stem cell differentiation	44/1600	2.92E-06	0.000137	44
BP	GO:0061138	morphogenesis of a branching epithelium	34/1600	2.97E-06	0.000139	34
BP	GO:0046942	carboxylic acid transport	48/1600	3.05E-06	0.000141	48
BP	GO:0035265	organ growth	33/1600	3.19E-06	0.000146	33
BP	GO:0003044	regulation of systemic arterial blood pressure mediated by a chemical signal	15/1600	3.24E-06	0.000146	15
BP	GO:0050919	negative chemotaxis	15/1600	3.24E-06	0.000146	15
BP	GO:0086001	cardiac muscle cell action potential	20/1600	3.52E-06	0.000158	20
BP	GO:0045664	regulation of neuron differentiation	36/1600	3.74E-06	0.000166	36
BP	GO:0099177	regulation of trans-synaptic signaling	62/1600	4.04E-06	0.000178	62
BP	GO:0007160	cell-matrix adhesion	41/1600	4.09E-06	0.000179	41
BP	GO:0070588	calcium ion transmembrane transport	50/1600	4.26E-06	0.000185	50
BP	GO:0042472	inner ear morphogenesis	22/1600	4.40E-06	0.000188	22
BP	GO:0090287	regulation of cellular response to growth factor stimulus	49/1600	4.40E-06	0.000188	49
BP	GO:0099504	synaptic vesicle cycle	34/1600	5.03E-06	0.000214	34
BP	GO:0060973	cell migration involved in heart development	9/1600	5.33E-06	0.000225	9
BP	GO:0002790	peptide secretion	61/1600	5.46E-06	0.000228	61
BP	GO:0019932	second-messenger-mediated signaling	50/1600	5.65E-06	0.000235	50
BP	GO:0048333	mesodermal cell differentiation	12/1600	6.02E-06	0.000248	12
BP	GO:0099003	vesicle-mediated transport in synapse	36/1600	6.12E-06	0.00025	36
BP	GO:1904064	positive regulation of cation transmembrane transport	29/1600	6.16E-06	0.00025	29
BP	GO:0010810	regulation of cell-substrate adhesion	39/1600	6.38E-06	0.000256	39
BP	GO:0035107	appendage morphogenesis	28/1600	6.45E-06	0.000256	28
BP	GO:0035108	limb morphogenesis	28/1600	6.45E-06	0.000256	28
BP	GO:0045165	cell fate commitment	43/1600	6.90E-06	0.00027	43
BP	GO:0090092	regulation of transmembrane receptor protein serine/threonine kinase signaling pathway	43/1600	6.90E-06	0.00027	43
BP	GO:0050890	cognition	46/1600	7.12E-06	0.000277	46
BP	GO:0001508	action potential	28/1600	7.48E-06	0.000288	28
BP	GO:0050804	modulation of chemical synaptic transmission	61/1600	7.52E-06	0.000288	61
BP	GO:0060571	morphogenesis of an epithelial fold	10/1600	7.66E-06	0.000292	10
BP	GO:0030048	actin filament-based movement	30/1600	7.74E-06	0.000292	30
BP	GO:0072132	mesenchyme morphogenesis	15/1600	7.82E-06	0.000292	15
BP	GO:0048638	regulation of developmental growth	51/1600	7.83E-06	0.000292	51
BP	GO:0048645	animal organ formation	17/1600	8.14E-06	0.000302	17
BP	GO:0021987	cerebral cortex development	24/1600	8.19E-06	0.000302	24
BP	GO:0048705	skeletal system morphogenesis	38/1600	8.91E-06	0.000326	38
BP	GO:0086014	atrial cardiac muscle cell action potential	9/1600	9.37E-06	0.000337	9
BP	GO:0086026	atrial cardiac muscle cell to AV node cell signaling	9/1600	9.37E-06	0.000337	9
BP	GO:0086066	atrial cardiac muscle cell to AV node cell communication	9/1600	9.37E-06	0.000337	9
BP	GO:0014074	response to purine-containing compound	29/1600	9.48E-06	0.000338	29
BP	GO:0051924	regulation of calcium ion transport	41/1600	9.74E-06	0.000346	41
BP	GO:0050678	regulation of epithelial cell proliferation	57/1600	1.04E-05	0.000366	57
BP	GO:0050879	multicellular organismal movement	16/1600	1.05E-05	0.000367	16
BP	GO:0050881	musculoskeletal movement	16/1600	1.05E-05	0.000367	16
BP	GO:0010232	vascular transport	21/1600	1.08E-05	0.000368	21
BP	GO:0060993	kidney morphogenesis	21/1600	1.08E-05	0.000368	21
BP	GO:0003007	heart morphogenesis	41/1600	1.08E-05	0.000368	41
BP	GO:0009636	response to toxic substance	41/1600	1.08E-05	0.000368	41
BP	GO:0042476	odontogenesis	26/1600	1.10E-05	0.00037	26
BP	GO:0051963	regulation of synapse assembly	22/1600	1.12E-05	0.000377	22
BP	GO:0007088	regulation of mitotic nuclear division	24/1600	1.14E-05	0.000382	24
BP	GO:2000209	regulation of anoikis	10/1600	1.21E-05	0.000402	10
BP	GO:0010720	positive regulation of cell development	47/1600	1.22E-05	0.000402	47
BP	GO:0042493	response to drug	55/1600	1.26E-05	0.000413	55
BP	GO:0032412	regulation of ion transmembrane transporter activity	43/1600	1.27E-05	0.000413	43
BP	GO:2001257	regulation of cation channel activity	33/1600	1.33E-05	0.00043	33
BP	GO:0060560	developmental growth involved in morphogenesis	39/1600	1.37E-05	0.000443	39
BP	GO:0006836	neurotransmitter transport	37/1600	1.39E-05	0.000446	37
BP	GO:0048639	positive regulation of developmental growth	32/1600	1.46E-05	0.000464	32
BP	GO:0060562	epithelial tube morphogenesis	49/1600	1.46E-05	0.000464	49
BP	GO:0015718	monocarboxylic acid transport	33/1600	1.50E-05	0.000472	33
BP	GO:0031346	positive regulation of cell projection organization	53/1600	1.54E-05	0.000482	53
BP	GO:0002027	regulation of heart rate	22/1600	1.60E-05	0.000498	22
BP	GO:0007369	gastrulation	33/1600	1.69E-05	0.000524	33
BP	GO:0042537	benzene-containing compound metabolic process	10/1600	1.87E-05	0.000576	10
BP	GO:0060045	positive regulation of cardiac muscle cell proliferation	11/1600	1.89E-05	0.000581	11
BP	GO:0050817	coagulation	53/1600	1.97E-05	0.000598	53
BP	GO:0060538	skeletal muscle organ development	30/1600	1.97E-05	0.000598	30
BP	GO:0010951	negative regulation of endopeptidase activity	41/1600	1.99E-05	0.0006	41
BP	GO:0000280	nuclear division	63/1600	2.03E-05	0.000609	63
BP	GO:0050730	regulation of peptidyl-tyrosine phosphorylation	43/1600	2.06E-05	0.000614	43
BP	GO:0030239	myofibril assembly	16/1600	2.18E-05	0.000646	16
BP	GO:0018958	phenol-containing compound metabolic process	23/1600	2.22E-05	0.000656	23
BP	GO:0051588	regulation of neurotransmitter transport	22/1600	2.25E-05	0.00066	22
BP	GO:0051962	positive regulation of nervous system development	43/1600	2.26E-05	0.000661	43
BP	GO:0006575	cellular modified amino acid metabolic process	35/1600	2.43E-05	0.000706	35
BP	GO:0006590	thyroid hormone generation	9/1600	2.55E-05	0.000735	9
BP	GO:0001654	eye development	54/1600	2.55E-05	0.000735	54
BP	GO:0034764	positive regulation of transmembrane transport	36/1600	2.69E-05	0.000766	36
BP	GO:0034767	positive regulation of ion transmembrane transport	36/1600	2.69E-05	0.000766	36
BP	GO:0048880	sensory system development	55/1600	2.80E-05	0.000785	55
BP	GO:0002026	regulation of the force of heart contraction	10/1600	2.81E-05	0.000785	10
BP	GO:0042403	thyroid hormone metabolic process	10/1600	2.81E-05	0.000785	10
BP	GO:0031016	pancreas development	18/1600	2.81E-05	0.000785	18
BP	GO:0010466	negative regulation of peptidase activity	42/1600	2.83E-05	0.000787	42
BP	GO:0060688	regulation of morphogenesis of a branching structure	14/1600	2.88E-05	0.000798	14
BP	GO:0046683	response to organophosphorus	26/1600	3.05E-05	0.000839	26
BP	GO:0016053	organic acid biosynthetic process	51/1600	3.06E-05	0.000839	51
BP	GO:0150104	transport across blood-brain barrier	20/1600	3.11E-05	0.000847	20
BP	GO:0090276	regulation of peptide hormone secretion	36/1600	3.32E-05	0.000902	36
BP	GO:0007494	midgut development	7/1600	3.38E-05	0.000909	7

BP	GO:0016264	gap junction assembly	7/1600	3.38E-05	0.000909	7
BP	GO:0016358	dendrite development	39/1600	3.44E-05	0.00092	39
BP	GO:0150063	visual system development	54/1600	3.48E-05	0.000927	54
BP	GO:0006979	response to oxidative stress	63/1600	3.56E-05	0.000942	63
BP	GO:0060043	regulation of cardiac muscle cell proliferation	14/1600	3.73E-05	0.000981	14
BP	GO:0046887	positive regulation of hormone secretion	25/1600	3.74E-05	0.000981	25
BP	GO:0007519	skeletal muscle tissue development	28/1600	3.90E-05	0.001019	28
BP	GO:0003081	regulation of systemic arterial blood pressure by renin-angiotensin	9/1600	3.99E-05	0.001037	9
BP	GO:0001505	regulation of neurotransmitter levels	36/1600	4.09E-05	0.001059	36
BP	GO:0070527	platelet aggregation	16/1600	4.25E-05	0.001094	16
BP	GO:0000070	mitotic sister chromatid segregation	30/1600	4.66E-05	0.001194	30
BP	GO:0007178	transmembrane receptor protein serine/threonine kinase signaling pathway	53/1600	4.68E-05	0.001194	53
BP	GO:0021675	nerve development	17/1600	4.71E-05	0.001197	17
BP	GO:0016079	synaptic vesicle exocytosis	22/1600	5.01E-05	0.001263	22
BP	GO:0007492	endoderm development	18/1600	5.01E-05	0.001263	18
BP	GO:0048285	organelle fission	67/1600	5.07E-05	0.001272	67
BP	GO:0060191	regulation of lipase activity	21/1600	5.15E-05	0.001282	21
BP	GO:0015849	organic acid transport	49/1600	5.16E-05	0.001282	49
BP	GO:0003073	regulation of systemic arterial blood pressure	20/1600	5.22E-05	0.001291	20
BP	GO:0086019	cell-cell signaling involved in cardiac conduction	11/1600	5.29E-05	0.001303	11
BP	GO:0007596	blood coagulation	51/1600	5.33E-05	0.001306	51
BP	GO:0086065	cell communication involved in cardiac conduction	15/1600	5.73E-05	0.001382	15
BP	GO:0010517	regulation of phospholipase activity	17/1600	5.73E-05	0.001382	17
BP	GO:0014041	regulation of neuron maturation	6/1600	5.75E-05	0.001382	6
BP	GO:0021892	cerebral cortex GABAergic interneuron differentiation	6/1600	5.75E-05	0.001382	6
BP	GO:0006874	cellular calcium ion homeostasis	62/1600	5.75E-05	0.001382	62
BP	GO:0021795	cerebral cortex cell migration	12/1600	5.96E-05	0.001413	12
BP	GO:0030049	muscle filament sliding	12/1600	5.96E-05	0.001413	12
BP	GO:0033275	actin-myosin filament sliding	12/1600	5.96E-05	0.001413	12
BP	GO:0001823	mesonephros development	20/1600	6.16E-05	0.001448	20
BP	GO:0003009	skeletal muscle contraction	13/1600	6.19E-05	0.001448	13
BP	GO:0086009	membrane repolarization	13/1600	6.19E-05	0.001448	13
BP	GO:0048754	branching morphogenesis of an epithelial tube	27/1600	6.23E-05	0.001448	27
BP	GO:1902850	microtubule cytoskeleton organization involved in mitosis	27/1600	6.23E-05	0.001448	27
BP	GO:0030073	insulin secretion	35/1600	6.29E-05	0.001455	35
BP	GO:0000302	response to reactive oxygen species	37/1600	6.57E-05	0.001513	37
BP	GO:0007229	integrin-mediated signaling pathway	22/1600	6.79E-05	0.001557	22
BP	GO:0055074	calcium ion homeostasis	63/1600	6.94E-05	0.001584	63
BP	GO:0010811	positive regulation of cell-substrate adhesion	24/1600	6.97E-05	0.001586	24
BP	GO:0060038	cardiac muscle cell proliferation	15/1600	7.13E-05	0.001615	15
BP	GO:0044060	regulation of endocrine process	11/1600	7.22E-05	0.001624	11
BP	GO:0007599	hemostasis	51/1600	7.23E-05	0.001624	51
BP	GO:0048660	regulation of smooth muscle cell proliferation	29/1600	7.36E-05	0.001647	29
BP	GO:0090130	tissue migration	53/1600	7.87E-05	0.001754	53
BP	GO:0043268	positive regulation of potassium ion transport	13/1600	7.96E-05	0.001768	13
BP	GO:0097237	cellular response to toxic substance	24/1600	7.99E-05	0.001768	24
BP	GO:0061448	connective tissue development	39/1600	8.06E-05	0.001777	39
BP	GO:0010976	positive regulation of neuron projection development	28/1600	8.16E-05	0.001792	28
BP	GO:0014855	striated muscle cell proliferation	17/1600	8.39E-05	0.001834	17
BP	GO:0051591	response to cAMP	20/1600	8.51E-05	0.001852	20
BP	GO:0003206	cardiac chamber morphogenesis	23/1600	8.57E-05	0.001852	23
BP	GO:1990748	cellular detoxification	23/1600	8.57E-05	0.001852	23
BP	GO:0001657	ureteric bud development	19/1600	8.65E-05	0.001863	19
BP	GO:0072006	nephron development	26/1600	8.78E-05	0.001883	26
BP	GO:0030318	melanocyte differentiation	9/1600	8.97E-05	0.001917	9
BP	GO:0048659	smooth muscle cell proliferation	29/1600	9.26E-05	0.001971	29
BP	GO:0001706	endoderm formation	14/1600	9.61E-05	0.00203	14
BP	GO:0021885	forebrain cell migration	14/1600	9.61E-05	0.00203	14
BP	GO:0050679	positive regulation of epithelial cell proliferation	34/1600	9.64E-05	0.00203	34
BP	GO:1901888	regulation of cell junction assembly	33/1600	9.88E-05	0.002073	33
BP	GO:0048644	muscle organ morphogenesis	17/1600	0.000101	0.002106	17
BP	GO:0072163	mesonephric epithelium development	19/1600	0.000102	0.002116	19
BP	GO:0072164	mesonephric tubule development	19/1600	0.000102	0.002116	19
BP	GO:0030850	prostate gland development	12/1600	0.000103	0.002122	12
BP	GO:0030324	lung development	29/1600	0.000104	0.002134	29
BP	GO:0061098	positive regulation of protein tyrosine kinase activity	15/1600	0.000108	0.002221	15
BP	GO:0048532	anatomical structure arrangement	7/1600	0.000109	0.002221	7
BP	GO:0030879	mammary gland development	25/1600	0.000109	0.002221	25
BP	GO:0060541	respiratory system development	32/1600	0.000112	0.00227	32
BP	GO:0033555	multicellular organismal response to stress	16/1600	0.000116	0.002346	16
BP	GO:0060415	muscle tissue morphogenesis	16/1600	0.000116	0.002346	16
BP	GO:0099624	atrial cardiac muscle cell membrane repolarization	6/1600	0.000117	0.002355	6
BP	GO:0086002	cardiac muscle cell action potential involved in contraction	14/1600	0.00012	0.002391	14
BP	GO:0001708	cell fate specification	19/1600	0.00012	0.002391	19
BP	GO:0006814	sodium ion transport	38/1600	0.000121	0.002403	38
BP	GO:0046394	carboxylic acid biosynthetic process	48/1600	0.000129	0.00254	48
BP	GO:0003014	renal system process	23/1600	0.000129	0.00254	23
BP	GO:0001990	regulation of systemic arterial blood pressure by hormone	11/1600	0.000129	0.00254	11
BP	GO:0030168	platelet activation	28/1600	0.00013	0.00254	28
BP	GO:0016266	O-glycan processing	15/1600	0.000133	0.002582	15
BP	GO:0003197	endocardial cushion development	12/1600	0.000133	0.002582	12
BP	GO:0019935	cyclic-nucleotide-mediated signaling	19/1600	0.000141	0.002724	19
BP	GO:0001656	metanephros development	18/1600	0.000144	0.002774	18
BP	GO:2000300	regulation of synaptic vesicle exocytosis	14/1600	0.000148	0.002854	14
BP	GO:0031032	actomyosin structure organization	32/1600	0.000152	0.002909	32
BP	GO:0048566	embryonic digestive tract development	10/1600	0.000158	0.003023	10
BP	GO:0030326	embryonic limb morphogenesis	22/1600	0.000159	0.003027	22
BP	GO:0035113	embryonic appendage morphogenesis	22/1600	0.000159	0.003027	22
BP	GO:0050727	regulation of inflammatory response	52/1600	0.00016	0.003027	52
BP	GO:0030323	respiratory tube development	29/1600	0.000161	0.003027	29
BP	GO:0050767	regulation of neurogenesis	50/1600	0.000161	0.003027	50
BP	GO:0003205	cardiac chamber development	27/1600	0.000162	0.00304	27
BP	GO:0015701	bicarbonate transport	12/1600	0.00017	0.003176	12
BP	GO:0002576	platelet degranulation	24/1600	0.000174	0.003244	24
BP	GO:0035051	cardiocyte differentiation	26/1600	0.000181	0.003353	26
BP	GO:0060402	calcium ion transport into cytosol	27/1600	0.000182	0.003353	27
BP	GO:0021761	limbic system development	20/1600	0.000182	0.003353	20
BP	GO:0006942	regulation of striated muscle contraction	19/1600	0.000191	0.003514	19
BP	GO:0022029	telencephalon cell migration	13/1600	0.000201	0.00368	13
BP	GO:0060420	regulation of heart growth	17/1600	0.000202	0.00368	17
BP	GO:0015850	organic hydroxy compound transport	41/1600	0.000202	0.00368	41
BP	GO:0009954	proximal/distal pattern formation	10/1600	0.000213	0.003856	10
BP	GO:0048546	digestive tract morphogenesis	12/1600	0.000216	0.00388	12
BP	GO:0060421	positive regulation of heart growth	12/1600	0.000216	0.00388	12
BP	GO:0009952	anterior/posterior pattern specification	33/1600	0.000216	0.00388	33

BP	GO:0007077	mitotic nuclear envelope disassembly	6/1600	0.000218	0.003888	6
BP	GO:0097154	GABAergic neuron differentiation	6/1600	0.000218	0.003888	6
BP	GO:0045214	sarcomere organization	11/1600	0.000221	0.003928	11
BP	GO:0045823	positive regulation of heart contraction	11/1600	0.000221	0.003928	11
BP	GO:2000242	negative regulation of reproductive process	14/1600	0.000223	0.003945	14
BP	GO:0002526	acute inflammatory response	21/1600	0.000225	0.003969	21
BP	GO:0007586	digestion	25/1600	0.000226	0.003969	25
BP	GO:0071900	regulation of protein serine/threonine kinase activity	65/1600	0.000227	0.003969	65
BP	GO:0002791	regulation of peptide secretion	45/1600	0.000229	0.003999	45
BP	GO:0034109	homotypic cell-cell adhesion	18/1600	0.000232	0.004039	18
BP	GO:0010965	regulation of mitotic sister chromatid separation	15/1600	0.000235	0.004087	15
BP	GO:0001570	vasculogenesis	17/1600	0.000238	0.004125	17
BP	GO:0072028	nephron morphogenesis	16/1600	0.00024	0.004141	16
BP	GO:0060419	heart growth	20/1600	0.000242	0.004161	20
BP	GO:0035725	sodium ion transmembrane transport	29/1600	0.000245	0.004194	29
BP	GO:0051346	negative regulation of hydrolase activity	61/1600	0.000249	0.004261	61
BP	GO:0072073	kidney epithelium development	24/1600	0.000251	0.004279	24
BP	GO:0007269	neurotransmitter secretion	27/1600	0.000253	0.004281	27
BP	GO:0099643	signal release from synapse	27/1600	0.000253	0.004281	27
BP	GO:0034446	substrate adhesion-dependent cell spreading	21/1600	0.000258	0.004352	21
BP	GO:0048588	developmental cell growth	35/1600	0.00026	0.004371	35
BP	GO:0045861	negative regulation of proteolysis	49/1600	0.000263	0.004422	49
BP	GO:0043010	camera-type eye development	45/1600	0.000265	0.00444	45
BP	GO:0006022	aminoglycan metabolic process	29/1600	0.000271	0.004473	29
BP	GO:0051965	positive regulation of synapse assembly	14/1600	0.000271	0.004473	14
BP	GO:0060675	ureteric bud morphogenesis	14/1600	0.000271	0.004473	14
BP	GO:0021545	cranial nerve development	12/1600	0.000272	0.004473	12
BP	GO:0060986	endocrine hormone secretion	12/1600	0.000272	0.004473	12
BP	GO:0085029	extracellular matrix assembly	12/1600	0.000272	0.004473	12
BP	GO:1990138	neuron projection extension	28/1600	0.000277	0.00454	28
BP	GO:0014812	muscle cell migration	20/1600	0.000278	0.004546	20
BP	GO:0003203	endocardial cushion morphogenesis	10/1600	0.000282	0.004583	10
BP	GO:1905332	positive regulation of morphogenesis of an epithelium	10/1600	0.000282	0.004583	10
BP	GO:0007158	neuron cell-cell adhesion	7/1600	0.000283	0.004583	7
BP	GO:0086003	cardiac muscle cell contraction	16/1600	0.000285	0.004583	16
BP	GO:1901381	positive regulation of potassium ion transmembrane transport	11/1600	0.000285	0.004583	11
BP	GO:0086012	membrane depolarization during cardiac muscle cell action potential	8/1600	0.000285	0.004583	8
BP	GO:0060401	cytosolic calcium ion transport	29/1600	0.000299	0.0048	29
BP	GO:0072330	monocarboxylic acid biosynthetic process	35/1600	0.000309	0.004938	35
BP	GO:0032355	response to estradiol	23/1600	0.000313	0.004996	23
BP	GO:0009306	protein secretion	51/1600	0.000316	0.005027	51
BP	GO:0007051	spindle organization	30/1600	0.000321	0.00508	30
BP	GO:0001942	hair follicle development	17/1600	0.000328	0.005146	17
BP	GO:0051047	positive regulation of secretion	43/1600	0.000328	0.005146	43
BP	GO:0030071	regulation of mitotic metaphase/anaphase transition	14/1600	0.000328	0.005146	14
BP	GO:0072171	mesonephric tubule morphogenesis	14/1600	0.000328	0.005146	14
BP	GO:0071560	cellular response to transforming growth factor beta stimulus	38/1600	0.000335	0.00522	38
BP	GO:0072503	cellular divalent inorganic cation homeostasis	63/1600	0.000336	0.00522	63
BP	GO:0021766	hippocampus development	16/1600	0.000336	0.00522	16
BP	GO:0051306	mitotic sister chromatid separation	15/1600	0.000337	0.00522	15
BP	GO:0035592	establishment of protein localization to extracellular region	51/1600	0.000338	0.00522	51
BP	GO:1900274	regulation of phospholipase C activity	12/1600	0.00034	0.005238	12
BP	GO:0010522	regulation of calcium ion transport into cytosol	19/1600	0.000342	0.005265	19
BP	GO:0007052	mitotic spindle organization	22/1600	0.000346	0.005293	22
BP	GO:0021988	olfactory lobe development	9/1600	0.000347	0.005293	9
BP	GO:0086011	membrane repolarization during action potential	9/1600	0.000347	0.005293	9
BP	GO:0090101	negative regulation of transmembrane receptor protein serine/threonine kinase signaling pathway	24/1600	0.000356	0.005419	24
BP	GO:0055023	positive regulation of cardiac muscle tissue growth	11/1600	0.000363	0.005481	11
BP	GO:1903524	positive regulation of blood circulation	11/1600	0.000363	0.005481	11
BP	GO:0014909	smooth muscle cell migration	18/1600	0.000364	0.005481	18
BP	GO:0072080	nephron tubule development	18/1600	0.000364	0.005481	18
BP	GO:0010092	specification of animal organ identity	10/1600	0.000369	0.005512	10
BP	GO:0043276	anoikis	10/1600	0.000369	0.005512	10
BP	GO:0050931	pigment cell differentiation	10/1600	0.000369	0.005512	10
BP	GO:0071599	otic vesicle development	6/1600	0.000375	0.005588	6
BP	GO:0014902	myotube differentiation	21/1600	0.000381	0.005651	21
BP	GO:0014910	regulation of smooth muscle cell migration	17/1600	0.000383	0.005653	17
BP	GO:0099601	regulation of neurotransmitter receptor activity	17/1600	0.000383	0.005653	17
BP	GO:2000241	regulation of reproductive process	27/1600	0.000385	0.005676	27
BP	GO:0045927	positive regulation of growth	38/1600	0.000392	0.005767	38
BP	GO:0048592	eye morphogenesis	25/1600	0.000397	0.005805	25
BP	GO:0042542	response to hydrogen peroxide	24/1600	0.000399	0.005805	24
BP	GO:0051384	response to glucocorticoid	24/1600	0.000399	0.005805	24
BP	GO:0051783	regulation of nuclear division	24/1600	0.000399	0.005805	24
BP	GO:0072078	nephron tubule morphogenesis	15/1600	0.000401	0.005808	15
BP	GO:2000027	regulation of animal organ morphogenesis	29/1600	0.000402	0.005808	29
BP	GO:1903532	positive regulation of secretion by cell	40/1600	0.000403	0.005808	40
BP	GO:0052547	regulation of peptidase activity	60/1600	0.000407	0.005859	60
BP	GO:0045839	negative regulation of mitotic nuclear division	12/1600	0.000421	0.006051	12
BP	GO:0051480	regulation of cytosolic calcium ion concentration	48/1600	0.000426	0.006099	48
BP	GO:0070293	renal absorption	7/1600	0.000429	0.006117	7
BP	GO:0072189	ureter development	7/1600	0.000429	0.006117	7
BP	GO:0031960	response to corticosteroid	26/1600	0.000436	0.006199	26
BP	GO:0046622	positive regulation of organ growth	13/1600	0.000457	0.006482	13
BP	GO:0044091	membrane biogenesis	11/1600	0.000459	0.006487	11
BP	GO:0007435	salivary gland morphogenesis	9/1600	0.000466	0.006553	9
BP	GO:0048240	sperm capacitation	9/1600	0.000466	0.006553	9
BP	GO:0007091	metaphase/anaphase transition of mitotic cell cycle	14/1600	0.000474	0.006644	14
BP	GO:0001707	mesoderm formation	15/1600	0.000475	0.006644	15
BP	GO:0001837	epithelial to mesenchymal transition	26/1600	0.000484	0.006758	26
BP	GO:0061326	renal tubule development	18/1600	0.000485	0.006761	18
BP	GO:0098657	import into cell	35/1600	0.000511	0.007103	35
BP	GO:0022404	molting cycle process	17/1600	0.000516	0.007127	17
BP	GO:0022405	hair cycle process	17/1600	0.000516	0.007127	17
BP	GO:0002067	glandular epithelial cell differentiation	12/1600	0.000519	0.007127	12
BP	GO:0006692	prostanoid metabolic process	12/1600	0.000519	0.007127	12
BP	GO:0006693	prostaglandin metabolic process	12/1600	0.000519	0.007127	12
BP	GO:0071692	protein localization to extracellular region	51/1600	0.000527	0.007221	51
BP	GO:0071559	response to transforming growth factor beta	38/1600	0.000536	0.007317	38
BP	GO:0010631	epithelial cell migration	49/1600	0.000545	0.00743	49
BP	GO:0006023	aminoglycan biosynthetic process	21/1600	0.000552	0.007504	21
BP	GO:0003208	cardiac ventricle morphogenesis	15/1600	0.00056	0.007514	15
BP	GO:0072088	nephron epithelium morphogenesis	15/1600	0.00056	0.007514	15
BP	GO:0007612	learning	23/1600	0.00056	0.007514	23
BP	GO:0010469	regulation of signaling receptor activity	30/1600	0.000562	0.007514	30

BP	GO:0014829	vascular associated smooth muscle contraction	8/1600	0.000562	0.007514	8
BP	GO:0045109	intermediate filament organization	8/1600	0.000562	0.007514	8
BP	GO:0072507	divalent inorganic cation homeostasis	64/1600	0.000564	0.007514	64
BP	GO:1901016	regulation of potassium ion transmembrane transporter activity	14/1600	0.000565	0.007514	14
BP	GO:1902099	regulation of metaphase/anaphase transition of cell cycle	14/1600	0.000565	0.007514	14
BP	GO:0033046	negative regulation of sister chromatid segregation	11/1600	0.000574	0.00759	11
BP	GO:0033048	negative regulation of mitotic sister chromatid segregation	11/1600	0.000574	0.00759	11
BP	GO:2000816	negative regulation of mitotic sister chromatid separation	11/1600	0.000574	0.00759	11
BP	GO:0042475	odontogenesis of dentin-containing tooth	17/1600	0.000597	0.007853	17
BP	GO:0098773	skin epidermis development	17/1600	0.000597	0.007853	17
BP	GO:0071709	membrane assembly	10/1600	0.000612	0.008014	10
BP	GO:0086010	membrane depolarization during action potential	10/1600	0.000612	0.008014	10
BP	GO:0110110	positive regulation of animal organ morphogenesis	9/1600	0.000616	0.008041	9
BP	GO:0007292	female gamete generation	23/1600	0.000626	0.008155	23
BP	GO:0032634	interleukin-5 production	7/1600	0.00063	0.008174	7
BP	GO:0032674	regulation of interleukin-5 production	7/1600	0.00063	0.008174	7
BP	GO:0055017	cardiac muscle tissue growth	18/1600	0.000639	0.008275	18
BP	GO:0048332	mesoderm morphogenesis	15/1600	0.000657	0.008465	15
BP	GO:0055021	regulation of cardiac muscle tissue growth	15/1600	0.000657	0.008465	15
BP	GO:0090132	epithelium migration	49/1600	0.000659	0.008466	49
BP	GO:0021872	forebrain generation of neurons	13/1600	0.000665	0.008526	13
BP	GO:0010927	cellular component assembly involved in morphogenesis	19/1600	0.00067	0.008554	19
BP	GO:0048662	negative regulation of smooth muscle cell proliferation	14/1600	0.00067	0.008554	14
BP	GO:0001894	tissue homeostasis	38/1600	0.000672	0.008554	38
BP	GO:0052548	regulation of endopeptidase activity	56/1600	0.000673	0.008557	56
BP	GO:0046928	regulation of neurotransmitter secretion	17/1600	0.000688	0.008728	17
BP	GO:0000819	sister chromatid segregation	31/1600	0.000697	0.00882	31
BP	GO:0010830	regulation of myotube differentiation	11/1600	0.000713	0.008985	11
BP	GO:0048599	oocyte development	11/1600	0.000713	0.008985	11
BP	GO:0048709	oligodendrocyte differentiation	18/1600	0.000731	0.009188	18
BP	GO:0032060	bleb assembly	5/1600	0.000765	0.009413	5
BP	GO:0097084	vascular associated smooth muscle cell development	5/1600	0.000765	0.009413	5
BP	GO:1900003	regulation of serine-type endopeptidase activity	5/1600	0.000765	0.009413	5
BP	GO:1902571	regulation of serine-type peptidase activity	5/1600	0.000765	0.009413	5
BP	GO:0021846	cell proliferation in forebrain	8/1600	0.000766	0.009413	8
BP	GO:0086013	membrane repolarization during cardiac muscle cell action potential	8/1600	0.000766	0.009413	8
BP	GO:0033045	regulation of sister chromatid segregation	15/1600	0.000769	0.009413	15
BP	GO:0061333	renal tubule morphogenesis	15/1600	0.000769	0.009413	15
BP	GO:1905818	regulation of chromosome separation	15/1600	0.000769	0.009413	15
BP	GO:0003229	ventricular cardiac muscle tissue development	12/1600	0.000772	0.009413	12
BP	GO:0007094	mitotic spindle assembly checkpoint	10/1600	0.000776	0.009413	10
BP	GO:0048846	axon extension involved in axon guidance	10/1600	0.000776	0.009413	10
BP	GO:0071173	spindle assembly checkpoint	10/1600	0.000776	0.009413	10
BP	GO:0071174	mitotic spindle checkpoint	10/1600	0.000776	0.009413	10
BP	GO:0099622	cardiac muscle cell membrane repolarization	10/1600	0.000776	0.009413	10
BP	GO:1902284	neuron projection extension involved in neuron projection guidance	10/1600	0.000776	0.009413	10
BP	GO:0044784	metaphase/anaphase transition of cell cycle	14/1600	0.000792	0.009567	14
BP	GO:0060411	cardiac septum morphogenesis	14/1600	0.000792	0.009567	14
BP	GO:0055008	cardiac muscle tissue morphogenesis	13/1600	0.000795	0.009589	13
BP	GO:0003180	aortic valve morphogenesis	9/1600	0.000803	0.009597	9
BP	GO:0007431	salivary gland development	9/1600	0.000803	0.009597	9
BP	GO:0010259	multicellular organism aging	9/1600	0.000803	0.009597	9
BP	GO:0045907	positive regulation of vasoconstriction	9/1600	0.000803	0.009597	9
BP	GO:0061097	regulation of protein tyrosine kinase activity	18/1600	0.000834	0.009948	18
CC	GO:0062023	collagen-containing extracellular matrix	99/1652	3.68E-21	2.03E-18	99
CC	GO:0042383	sarcolemma	47/1652	5.17E-18	1.43E-15	47
CC	GO:0043292	contractile fiber	58/1652	2.46E-14	4.52E-12	58
CC	GO:0030016	myofibril	55/1652	3.10E-13	4.28E-11	55
CC	GO:0031674	I band	39/1652	3.16E-12	3.48E-10	39
CC	GO:0030017	sarcomere	49/1652	1.18E-11	1.09E-09	49
CC	GO:0030018	Z disc	35/1652	6.33E-11	4.99E-09	35
CC	GO:0043025	neuronal cell body	77/1652	1.90E-08	1.31E-06	77
CC	GO:0045178	basal part of cell	50/1652	2.32E-08	1.42E-06	50
CC	GO:0005581	collagen trimer	25/1652	3.20E-08	1.77E-06	25
CC	GO:0008305	integrin complex	14/1652	6.11E-08	3.07E-06	14
CC	GO:0098636	protein complex involved in cell adhesion	15/1652	7.73E-08	3.28E-06	15
CC	GO:0032432	actin filament bundle	22/1652	8.31E-08	3.28E-06	22
CC	GO:0045177	apical part of cell	68/1652	8.32E-08	3.28E-06	68
CC	GO:0009925	basal plasma membrane	46/1652	1.18E-07	4.33E-06	46
CC	GO:0016323	basolateral plasma membrane	42/1652	1.40E-07	4.82E-06	42
CC	GO:1902495	transmembrane transporter complex	56/1652	1.87E-07	6.08E-06	56
CC	GO:0016324	apical plasma membrane	59/1652	2.66E-07	8.16E-06	59
CC	GO:0034702	ion channel complex	52/1652	5.69E-07	1.65E-05	52
CC	GO:0001725	stress fiber	19/1652	8.12E-07	2.13E-05	19
CC	GO:0097517	contractile actin filament bundle	19/1652	8.12E-07	2.13E-05	19
CC	GO:1990351	transporter complex	56/1652	8.56E-07	2.15E-05	56
CC	GO:0008076	voltage-gated potassium channel complex	23/1652	9.11E-07	2.19E-05	23
CC	GO:0044306	neuron projection terminus	29/1652	1.05E-06	2.42E-05	29
CC	GO:0098793	presynapse	73/1652	1.10E-06	2.42E-05	73
CC	GO:0034703	cation channel complex	41/1652	1.54E-06	3.26E-05	41
CC	GO:0030055	cell-substrate junction	65/1652	1.79E-06	3.65E-05	65
CC	GO:0034705	potassium channel complex	24/1652	1.85E-06	3.65E-05	24
CC	GO:0030315	T-tubule	16/1652	2.65E-06	5.05E-05	16
CC	GO:0097060	synaptic membrane	58/1652	2.85E-06	5.24E-05	58
CC	GO:0000779	condensed chromosome, centromeric region	26/1652	4.03E-06	7.18E-05	26
CC	GO:0005925	focal adhesion	63/1652	4.19E-06	7.23E-05	63
CC	GO:0043034	costamere	9/1652	5.23E-06	8.76E-05	9
CC	GO:0043679	axon terminus	25/1652	7.86E-06	0.000128	25
CC	GO:0150034	distal axon	47/1652	8.71E-06	0.000135	47
CC	GO:0042641	actomyosin	19/1652	8.78E-06	0.000135	19
CC	GO:0045121	membrane raft	51/1652	1.05E-05	0.000153	51
CC	GO:0098857	membrane microdomain	51/1652	1.05E-05	0.000153	51
CC	GO:0005796	Golgi lumen	23/1652	1.10E-05	0.000156	23
CC	GO:0000777	condensed chromosome kinetochore	23/1652	2.15E-05	0.000296	23
CC	GO:0043204	perikaryon	29/1652	2.34E-05	0.000315	29
CC	GO:0005788	endoplasmic reticulum lumen	47/1652	4.69E-05	0.000616	47
CC	GO:0032279	asymmetric synapse	48/1652	6.11E-05	0.000776	48
CC	GO:0000940	condensed chromosome outer kinetochore	7/1652	6.18E-05	0.000776	7
CC	GO:0000776	kinetochore	26/1652	7.47E-05	0.000916	26
CC	GO:0098984	neuron to neuron synapse	50/1652	9.90E-05	0.001188	50
CC	GO:0005911	cell-cell junction	65/1652	0.000142	0.001666	65
CC	GO:0014069	postsynaptic density	46/1652	0.000163	0.001875	46
CC	GO:1990752	microtubule end	10/1652	0.000209	0.00235	10
CC	GO:0000775	chromosome, centromeric region	32/1652	0.000237	0.002618	32
CC	GO:0000778	condensed nuclear chromosome kinetochore	7/1652	0.000279	0.00298	7

CC	GO:0035371	microtubule plus-end	8/1652	0.000281	0.00298	8
CC	GO:0005604	basement membrane	19/1652	0.000289	0.003011	19
CC	GO:0005901	caveola	17/1652	0.00032	0.003266	17
CC	GO:0000793	condensed chromosome	34/1652	0.000341	0.003423	34
CC	GO:0044853	plasma membrane raft	21/1652	0.00037	0.003643	21
CC	GO:0098936	intrinsic component of postsynaptic membrane	20/1652	0.00046	0.004458	20
CC	GO:0005884	actin filament	21/1652	0.000475	0.004495	21
CC	GO:0045211	postsynaptic membrane	39/1652	0.00048	0.004495	39
CC	GO:0016010	dystrophin-associated glycoprotein complex	7/1652	0.000622	0.005625	7
CC	GO:0090665	glycoprotein complex	7/1652	0.000622	0.005625	7
CC	GO:0016529	sarcoplasmic reticulum	15/1652	0.000642	0.005717	15
CC	GO:0099572	postsynaptic specialization	46/1652	0.00066	0.005785	46
CC	GO:0016528	sarcoplasm	16/1652	0.000828	0.00714	16
CC	GO:0031941	filamentous actin	9/1652	0.001017	0.00864	9
MF	GO:0022803	passive transmembrane transporter activity	90/1599	1.68E-12	1.68E-09	90
MF	GO:0015267	channel activity	89/1599	3.77E-12	1.89E-09	89
MF	GO:0005539	glycosaminoglycan binding	53/1599	2.48E-11	8.27E-09	53
MF	GO:0005216	ion channel activity	78/1599	2.97E-10	7.43E-08	78
MF	GO:0005201	extracellular matrix structural constituent	42/1599	3.90E-10	7.80E-08	42
MF	GO:0022836	gated channel activity	65/1599	7.72E-10	1.29E-07	65
MF	GO:0008201	heparin binding	39/1599	5.26E-09	7.52E-07	39
MF	GO:0005261	cation channel activity	62/1599	7.86E-09	9.83E-07	62
MF	GO:1901681	sulfur compound binding	52/1599	1.07E-08	1.19E-06	52
MF	GO:0046873	metal ion transmembrane transporter activity	72/1599	3.11E-08	3.11E-06	72
MF	GO:0072341	modified amino acid binding	26/1599	5.80E-08	5.27E-06	26
MF	GO:0005244	voltage-gated ion channel activity	40/1599	3.43E-07	2.64E-05	40
MF	GO:0022832	voltage-gated channel activity	40/1599	3.43E-07	2.64E-05	40
MF	GO:0003779	actin binding	69/1599	1.08E-06	7.38E-05	69
MF	GO:0005267	potassium channel activity	28/1599	1.11E-06	7.38E-05	28
MF	GO:0004857	enzyme inhibitor activity	62/1599	1.66E-06	0.000104	62
MF	GO:0015079	potassium ion transmembrane transporter activity	32/1599	2.20E-06	0.00013	32
MF	GO:0050840	extracellular matrix binding	17/1599	2.47E-06	0.000137	17
MF	GO:0005249	voltage-gated potassium channel activity	22/1599	3.77E-06	0.000198	22
MF	GO:0061134	peptidase regulator activity	41/1599	7.99E-06	0.0004	41
MF	GO:0004867	serine-type endopeptidase inhibitor activity	23/1599	8.86E-06	0.000422	23
MF	GO:0004714	transmembrane receptor protein tyrosine kinase activity	17/1599	1.17E-05	0.000515	17
MF	GO:0030414	peptidase inhibitor activity	35/1599	1.22E-05	0.000515	35
MF	GO:0019199	transmembrane receptor protein kinase activity	20/1599	1.24E-05	0.000515	20
MF	GO:0004866	endopeptidase inhibitor activity	34/1599	1.32E-05	0.000528	34
MF	GO:0030020	extracellular matrix structural constituent conferring tensile strength	13/1599	2.76E-05	0.001061	13
MF	GO:0042805	actinin binding	12/1599	3.19E-05	0.001183	12
MF	GO:0005178	integrin binding	28/1599	3.32E-05	0.001186	28
MF	GO:0048018	receptor ligand activity	69/1599	3.63E-05	0.001227	69
MF	GO:0022839	ion gated channel activity	13/1599	3.68E-05	0.001227	13
MF	GO:0004859	phospholipase inhibitor activity	7/1599	4.05E-05	0.001266	7
MF	GO:1900750	oligopeptide binding	7/1599	4.05E-05	0.001266	7
MF	GO:0061135	endopeptidase regulator activity	34/1599	5.28E-05	0.001577	34
MF	GO:0030546	signaling receptor activator activity	69/1599	5.36E-05	0.001577	69
MF	GO:0022843	voltage-gated cation channel activity	27/1599	5.98E-05	0.001709	27
MF	GO:0048306	calcium-dependent protein binding	19/1599	8.92E-05	0.002477	19
MF	GO:0033293	monocarboxylic acid binding	17/1599	9.77E-05	0.002642	17
MF	GO:0031406	carboxylic acid binding	32/1599	0.000123	0.003233	32
MF	GO:0005227	calcium activated cation channel activity	9/1599	0.00016	0.004011	9
MF	GO:0005544	calcium-dependent phospholipid binding	14/1599	0.00016	0.004011	14
MF	GO:0008307	structural constituent of muscle	12/1599	0.000172	0.004205	12
MF	GO:0005200	structural constituent of cytoskeleton	21/1599	0.000189	0.004492	21
MF	GO:0005518	collagen binding	16/1599	0.000193	0.004492	16
MF	GO:0015085	calcium ion transmembrane transporter activity	25/1599	0.000215	0.004878	25
MF	GO:0043295	glutathione binding	6/1599	0.000254	0.005637	6
MF	GO:0051015	actin filament binding	34/1599	0.000262	0.005705	34
MF	GO:0051393	alpha-actinin binding	9/1599	0.000313	0.006667	9
MF	GO:0008083	growth factor activity	28/1599	0.000352	0.007207	28
MF	GO:0008509	anion transmembrane transporter activity	62/1599	0.000355	0.007207	62
MF	GO:0098631	cell adhesion mediator activity	14/1599	0.00036	0.007207	14
MF	GO:0015276	ligand-gated ion channel activity	25/1599	0.000385	0.007399	25
MF	GO:0022834	ligand-gated channel activity	25/1599	0.000385	0.007399	25
MF	GO:0140103	catalytic activity, acting on a glycoprotein	8/1599	0.000488	0.009212	8
MF	GO:0055102	lipase inhibitor activity	7/1599	0.000508	0.009414	7
MF	GO:0001786	phosphatidylserine binding	14/1599	0.000523	0.009501	14
