# Supplementary material for: Integrative Analysis of Bulk RNA-Seq and Single-Cell RNA-Seq Unveils the Characteristics of the Immune Microenvironment and Prognosis Signature in Prostate Cancer
Source: J Oncol. 2022 Jul 19;2022:6768139. doi: 10.1155/2022/6768139 (PMC9325591; doi:10.1155/2022/6768139)
Supplement: Supplementary Materials — Figure S1. Workflow of the analysis. Figure S2. Validation of the risk score model using the GSE54460 dataset. A. Patients with prostate cancer (PRAD) in the GSE54460 cohort are listed in ascending order of risk score. B. Progression-free interval (PFI) distribution versus the risk score of each patient in the GSE54460 cohort. C. Kaplan–Meier (KM) curves of patients with different risk levels in the GSE54460 validation set. D. Receiver Operating Characteristic (ROC) curve analysis for 1-, 3- and 5-year PFI using the clinical information of patients of the GSE54460 validation dataset. Figure S3. Validation of the risk score model using the GSE46602 dataset. A. Patients with prostate cancer (PRAD) in the GSE46602 cohort are listed in ascending order of risk score. B. Progression-free interval (PFI) distribution versus the risk score of each patient in the GSE46602 cohort. C. Kaplan–Meier (KM) curves of patients with different risk levels in the GSE46602 validation dataset. D. Receiver Operating Characteristic (ROC) curve analysis for 1-, 3- and 5-year PFI using the clinical information of patients of the GSE46602 validation dataset. Figure S4. Validation of the risk score model using the GSE70768 dataset. A. Patients with prostate cancer (PRAD) in the GSE70768 cohort are listed in ascending order of risk score. B. Progression-free interval (PFI) distribution versus the risk score of each patient in the GSE70768 cohort. C. Kaplan–Meier (KM) curves of patients with different risk levels in the GSE70768 validation dataset. D. Receiver Operating Characteristic (ROC) curve analysis for 1-, 3- and 5-year PFI using the clinical information of patients of the GSE70768 validation dataset. Figure S5. Validation of the risk score model using the GSE70769 dataset. A. Patients with prostate cancer (PRAD) in the GSE70769 validation dataset are listed in ascending order of risk score. B. Progression-free interval (PFI) distribution versus the risk score of each patient in the GSE707 [file 6768139.f1.zip › 6768139.f1/Table S3.pdf]

ID	Description	GeneRatio	pvalue	p.adjust	qvalue	Count
hsa05410	Hypertrophic cardiomyopathy	28/742	3.32E-09	5.88E-07	4.85E-07	28
hsa05414	Dilated cardiomyopathy	29/742	3.73E-09	5.88E-07	4.85E-07	29
hsa04510	Focal adhesion	45/742	9.53E-09	1.00E-06	8.26E-07	45
hsa04512	ECM-receptor interaction	26/742	4.01E-08	3.12E-06	2.58E-06	26
hsa04020	Calcium signaling pathway	49/742	4.95E-08	3.12E-06	2.58E-06	49
hsa05412	Arrhythmogenic right ventricular cardiomyopathy	23/742	1.95E-07	1.03E-05	8.47E-06	23
hsa04270	Vascular smooth muscle contraction	29/742	7.32E-06	0.00033	0.000272	29
hsa04151	PI3K-Akt signaling pathway	56/742	2.89E-05	0.001139	0.00094	56
hsa04974	Protein digestion and absorption	23/742	4.29E-05	0.001502	0.001239	23
hsa00590	Arachidonic acid metabolism	16/742	8.32E-05	0.002622	0.002164	16
hsa04022	cGMP-PKG signaling pathway	31/742	0.000101	0.002879	0.002376	31
hsa04360	Axon guidance	32/742	0.000224	0.005875	0.004849	32
hsa04972	Pancreatic secretion	21/742	0.000306	0.007421	0.006126	21
hsa04261	Adrenergic signaling in cardiomyocytes	27/742	0.000463	0.010419	0.0086	27
hsa04610	Complement and coagulation cascades	18/742	0.000567	0.011916	0.009835	18
hsa04810	Regulation of actin cytoskeleton	35/742	0.000688	0.013548	0.011183	35
hsa05205	Proteoglycans in cancer	33/742	0.000925	0.017135	0.014143	33
hsa00350	Tyrosine metabolism	10/742	0.001101	0.019268	0.015904	10
hsa04024	cAMP signaling pathway	34/742	0.001483	0.024585	0.020292	34
hsa04911	Insulin secretion	17/742	0.001788	0.027433	0.022643	17
hsa04110	Cell cycle	22/742	0.001829	0.027433	0.022643	22
hsa04014	Ras signaling pathway	35/742	0.002105	0.030135	0.024874	35
hsa04918	Thyroid hormone synthesis	15/742	0.002892	0.039007	0.032196	15
hsa00360	Phenylalanine metabolism	6/742	0.002972	0.039007	0.032196	6
hsa03320	PPAR signaling pathway	15/742	0.003306	0.040054	0.03306	15
hsa04971	Gastric acid secretion	15/742	0.003306	0.040054	0.03306	15
hsa04726	Serotonergic synapse	20/742	0.003672	0.042842	0.035362	20
hsa04970	Salivary secretion	17/742	0.004227	0.046501	0.038382	17
hsa00980	Metabolism of xenobiotics by cytochrome P450	15/742	0.004281	0.046501	0.038382	15
