# Supplementary material for: Integrative Analysis of Bulk RNA-Seq and Single-Cell RNA-Seq Unveils the Characteristics of the Immune Microenvironment and Prognosis Signature in Prostate Cancer
Source: J Oncol. 2022 Jul 19;2022:6768139. doi: 10.1155/2022/6768139 (PMC9325591; doi:10.1155/2022/6768139)
Supplement: Supplementary Materials — Figure S1. Workflow of the analysis. Figure S2. Validation of the risk score model using the GSE54460 dataset. A. Patients with prostate cancer (PRAD) in the GSE54460 cohort are listed in ascending order of risk score. B. Progression-free interval (PFI) distribution versus the risk score of each patient in the GSE54460 cohort. C. Kaplan–Meier (KM) curves of patients with different risk levels in the GSE54460 validation set. D. Receiver Operating Characteristic (ROC) curve analysis for 1-, 3- and 5-year PFI using the clinical information of patients of the GSE54460 validation dataset. Figure S3. Validation of the risk score model using the GSE46602 dataset. A. Patients with prostate cancer (PRAD) in the GSE46602 cohort are listed in ascending order of risk score. B. Progression-free interval (PFI) distribution versus the risk score of each patient in the GSE46602 cohort. C. Kaplan–Meier (KM) curves of patients with different risk levels in the GSE46602 validation dataset. D. Receiver Operating Characteristic (ROC) curve analysis for 1-, 3- and 5-year PFI using the clinical information of patients of the GSE46602 validation dataset. Figure S4. Validation of the risk score model using the GSE70768 dataset. A. Patients with prostate cancer (PRAD) in the GSE70768 cohort are listed in ascending order of risk score. B. Progression-free interval (PFI) distribution versus the risk score of each patient in the GSE70768 cohort. C. Kaplan–Meier (KM) curves of patients with different risk levels in the GSE70768 validation dataset. D. Receiver Operating Characteristic (ROC) curve analysis for 1-, 3- and 5-year PFI using the clinical information of patients of the GSE70768 validation dataset. Figure S5. Validation of the risk score model using the GSE70769 dataset. A. Patients with prostate cancer (PRAD) in the GSE70769 validation dataset are listed in ascending order of risk score. B. Progression-free interval (PFI) distribution versus the risk score of each patient in the GSE707 [file 6768139.f1.zip › 6768139.f1/Table S4.pdf]

Immune_Cell	Activated	Activated	CD56dim natural killer cell	Central memory CD4 T cell	Central memory CD8 T cell	Effector memeory CD4 T cell	Effector memeory CD8 T cell	Eosinophil	Immature_B cell	Immature dendritic cell	Mast cell	Memory B cell	Monocyte	Natural killer cell	Natural killer T cell	Neutrophil	Plasmacytoid dendritic cell	T follicular helper cell	Type 1 T helper cell	Type 17 T helper cell	Type 2 T helper cell
TCGA.EJ.7331.11A.01R.2118.07	-0.1859	0.00718	0.1328229	0.36110675	0.209328295	0.051124938	0.014833505	-0.275720909	-0.085458024	0.281369531	0.046398623	-0.04736301	0.338453648	0.316793033	-0.030372855	-0.23807164	0.369015694	0.046122994	0.0046969	-0.200412105	-0.094892089
TCGA.EJ.7792.11A.01R.2118.07	-0.2123	0.02746	0.136103145	0.381386389	0.173744248	-0.033692321	0.084583201	-0.29865582	-0.106264819	0.270237176	0.041832438	-0.036344226	0.34513505	0.32324351	-0.025512699	-0.23794935	0.360021685	0.054338441	-0.005710001	-0.161004339	-0.080089917
TCGA.EJ.7797.11A.01R.2263.07	-0.2157	0.06963	0.122515491	0.339575556	0.169697035	-0.010655234	-0.00594029	-0.207324996	-0.125163836	0.288592399	0.009683582	-0.093957689	0.342611746	0.280327814	-0.053318366	-0.274678355	0.362387915	0.022288656	-0.019736603	-0.204195815	-0.085937981
TCGA.G9.6351.11A.01R.1965.07	-0.2828	-0.0037	0.144465521	0.331699326	0.123840982	-0.035066901	-0.042388108	-0.283592677	-0.18981361	0.250512395	-0.095366064	-0.103910599	0.377009479	0.264954098	-0.093726826	-0.149544456	0.323079239	-0.026086015	-0.078383268	-0.166045617	-0.123230704
TCGA.CH.5761.11A.01R.1580.07	-0.2344	0.00537	0.174608212	0.344054263	0.153979026	-0.046034977	0.030855671	-0.239054955	-0.168432911	0.233750834	-0.068363083	-0.013942368	0.376020831	0.292710065	-0.118003301	-0.199745679	0.330738386	-0.008346258	-0.073020102	-0.132897656	-0.133355663
TCGA.EJ.7115.11A.01R.2118.07	-0.2764	0.01382	0.140343285	0.32964654	0.140910538	-0.04980601	-0.03586403	-0.358211164	-0.205193334	0.241914927	-0.015007362	-0.043510234	0.370923104	0.272113377	-0.108702103	-0.178842971	0.330133455	-0.01834774	-0.077792891	-0.141773494	-0.127858312
TCGA.CH.5767.11B.01R.1789.07	-0.2702	-0.0178	0.182506184	0.3679658	0.233941758	-0.036810856	0.03214158	-0.003603054	-0.103495647	0.281806351	0.064379885	0.031121995	0.341136016	0.308509969	-0.055844256	-0.202507896	0.35849017	0.026626232	0.011602974	-0.141270231	-0.060584155
TCGA.HC.7740.11A.01R.2118.07	-0.2762	-0.0554	0.206615046	0.349510423	0.201750578	0.014660746	-0.078640705	-0.240033181	-0.091179029	0.303679838	-0.105380202	-0.061652008	0.338753211	0.291409044	-0.07234272	-0.371912369	0.372903709	0.033209692	-0.012083508	-0.08178288	-0.073137793
TCGA.EJ.7793.11A.01R.2263.07	-0.2405	-0.0073	0.15224072	0.36920471	0.195260313	-0.00023047	0.001900416	-0.263396508	-0.096865385	0.297675713	0.006640085	-0.043576699	0.34031819	0.338474943	-0.064283507	-0.232486976	0.327512189	0.039437847	-0.041236402	-0.203758748	-0.109978807
TCGA.G9.6363.11A.01R.1789.07	-0.306	-0.0026	0.149860864	0.339434747	0.167024703	0.008328393	-0.078724278	-0.43234161	-0.248732022	0.255979586	-0.091742655	-0.072907705	0.356970442	0.236095891	-0.078889276	-0.232168703	0.311737816	-0.047508606	-0.077287694	-0.248742259	-0.159062565
TCGA.EJ.7783.11A.01R.2118.07	-0.2374	-0.0073	0.085982675	0.390347717	0.2238712	0.085685343	0.06112229	-0.247048196	-0.10188069	0.264163249	0.125324518	-0.08944369	0.256133466	0.291332456	0.001772783	-0.338916731	0.369788769	0.051313212	0.027449822	-0.188429482	-0.074109403
TCGA.HC.8262.11A.01R.2263.07	-0.3159	0.00182	0.214610632	0.352275515	0.233070438	-0.010308865	0.054928783	-0.074489323	-0.178367472	0.254655504	0.068465218	0.031153936	0.338368559	0.297181692	-0.026697969	-0.310061344	0.351638821	0.038430976	0.009497586	-0.155487787	-0.079731633
TCGA.EJ.7789.11A.01R.2118.07	-0.216	0.03273	0.129510963	0.376448171	0.153323486	-0.055710159	0.027993686	-0.283383249	-0.13813883	0.275468484	0.042666522	-0.073602021	0.371376694	0.294955882	-0.092211465	-0.286128755	0.34466898	0.019373457	-0.057950071	-0.167330456	-0.088244263
TCGA.G9.6496.11A.01R.1789.07	-0.23	0.01885	0.168795393	0.378896727	0.179148063	-0.005405974	-0.033953095	-0.157356938	-0.232933196	0.241598115	0.04199867	-0.076608854	0.355619967	0.29029963	-0.036386938	-0.223761808	0.33180336	0.015839907	-0.052385111	-0.131446027	-0.115006321
TCGA.EJ.7786.11A.01R.2118.07	-0.2551	-0.0173	0.127100176	0.385463245	0.247553113	-0.023565906	0.056147348	-0.068111679	-0.119044906	0.291767523	0.159008595	-0.002410899	0.334392286	0.348028984	0.021329061	-0.237449818	0.38822173	0.106951784	0.058739802	-0.172893041	-0.024192318
TCGA.EJ.7794.11A.01R.2118.07	-0.2016	-0.0056	0.155042778	0.39955328	0.228984888	0.007432749	0.09689444	-0.135957868	-0.070566055	0.261924297	0.159718947	-0.009246407	0.34998241	0.370747278	0.023406918	-0.198289564	0.37982444	0.078359593	0.05193091	-0.134452762	-0.06211263
TCGA.EJ.7125.11A.01R.1965.07	-0.254	0.13237	0.115006908	0.339166931	0.187411688	-0.009134477	0.00630792	-0.262928461	-0.113653064	0.283070792	-0.005020869	-0.142998257	0.346954796	0.246638886	-0.049850261	-0.290973708	0.299411127	0.024815788	-0.008261791	-0.156485129	-0.087542284
TCGA.EJ.7785.11A.01R.2118.07	-0.2054	0.04224	0.09786111	0.342818199	0.185765903	0.027075584	0.016460765	-0.266209361	-0.114187558	0.291885627	0.00486594	-0.10238207	0.342298715	0.267870128	-0.059856811	-0.271808165	0.358732237	0.023837401	-0.013728716	-0.178295435	-0.076424546
TCGA.EJ.7315.11A.01R.2118.07	0.06039	0.20749	0.128630754	0.42342514	0.207591977	0.049233685	0.15809217	-0.170191946	0.049556509	0.288725013	0.055696357	0.051214587	0.354713771	0.359849836	0.02857052	-0.228738023	0.400591832	0.121626125	0.096192162	-0.168365656	-0.008086235
TCGA.EJ.7781.11A.01R.2118.07	-0.203	0.07111	0.103694616	0.382467297	0.234818986	0.016048425	0.092653702	-0.173517064	-0.04485182	0.301186008	0.126896152	-0.025028153	0.317994535	0.36607196	0.000844616	-0.286245487	0.39061914	0.111319132	0.04511877	-0.21340984	-0.050407027
TCGA.HC.8260.11A.01R.2263.07	-0.3284	-0.0431	0.144070178	0.341629643	0.12467895	-0.069836097	-0.074093539	-0.2832918	-0.263770707	0.275202342	-0.08195186	-0.116971686	0.375260946	0.250005369	-0.127621275	-0.334785063	0.328227415	-0.054067021	-0.110107952	-0.17553662	-0.115692606
TCGA.EJ.7314.11A.01R.2118.07	-0.3039	-0.0385	0.166083002	0.366117605	0.204160584	-0.012007439	0.022648393	-0.101774079	-0.147453371	0.240772806	0.099481771	-0.017430706	0.337662241	0.324342544	-0.015518487	-0.21459918	0.37488324	0.07425222	0.015475737	-0.176109498	-0.058491467
TCGA.G9.6333.11A.01R.1965.07	-0.344	0.00534	0.189126672	0.373241041	0.204084505	0.002805434	-0.034827842	-0.229679621	-0.18680752	0.288586768	-0.038343295	-0.020288026	0.357460046	0.321555715	-0.056281244	-0.361723064	0.332203936	0.038554798	-0.047277219	-0.079005149	-0.141581533
TCGA.G9.6362.11A.01R.1789.07	-0.4591	-0.0424	0.094468041	0.279149689	0.11707971	0.000369315	-0.073251901	-0.222748672	-0.229734246	0.195324158	-0.02757099	-0.181156462	0.319751041	0.217157982	-0.088398409	-0.148321661	0.308100751	-0.030214031	-0.081223432	-0.248560149	-0.098423164
TCGA.G9.6499.11A.02R.1965.07	-0.3441	0.00624	0.081205649	0.354293787	0.155373763	-0.033829068	-0.07093575	-0.302839917	-0.228580344	0.254359418	-0.107794073	-0.063431185	0.366950141	0.240931477	-0.047944757	-0.268548064	0.315049288	0.003042956	-0.057880882	-0.138146961	-0.135230989
TCGA.HC.7737.11A.02R.2118.07	-0.2573	-0.0461	0.189864417	0.358256177	0.216233494	-0.015696465	-0.03831414	-0.305937471	-0.136301734	0.286087939	-0.120641335	0.004269566	0.346694379	0.271716212	-0.060466703	-0.197661618	0.370711121	0.003227907	0.012559564	-0.104244031	-0.093743806
TCGA.EJ.A8FO.11A.11R.A36G.07	-0.3013	-0.0466	0.169050253	0.386996948	0.192648922	0.102878371	-0.020966181	-0.118874771	-0.247780009	0.272755648	0.039089264	-0.037497216	0.336046248	0.291870023	-0.061424954	-0.297117534	0.384160946	0.035650167	-0.018508641	-0.156074016	-0.056503977
TCGA.HC.7738.11A.01R.2118.07	-0.2955	-0.061	0.174885235	0.31716757	0.197574303	-0.028415305	-0.066431663	-0.192652033	-0.133879834	0.297831851	-0.080443048	-0.012708277	0.332923403	0.294398072	-0.067427427	-0.414422387	0.380195704	0.025608334	-0.002211088	-0.082369406	-0.080921467
TCGA.G9.6342.11A.02R.1965.07	-0.2743	0.04603	0.148401083	0.325730784	0.147316748	-0.014722526	-0.077687897	-0.241633664	-0.244211086	0.251875512	-0.114129209	-0.121625832	0.393985882	0.249989534	-0.100741142	-0.23921128	0.291534428	-0.036137066	-0.076091874	-0.156046671	-0.137348009
TCGA.EJ.7784.11A.01R.2118.07	-0.2585	0.02404	0.140966026	0.378810407	0.170940454	-0.000517156	0.076953523	-0.184572912	-0.141937668	0.295606889	0.011971004	-0.018627165	0.338864642	0.341227692	-0.041133657	-0.275587546	0.34673668	0.02052034	-0.041011143	-0.13339083	-0.07937273
TCGA.J4.A83J.11A.11R.A36G.07	-0.2591	0.0509	0.196531335	0.356230246	0.160794194	-0.0437099	0.083099401	-0.084522388	-0.199040334	0.254413121	0.14738913	-0.049519765	0.342644275	0.320903467	-0.022199333	-0.226124947	0.386168609	0.04445777	0.011964378	-0.104950749	-0.111085296
TCGA.HC.7211.11A.01R.2118.07	-0.3503	-0.0447	0.215595352	0.312752188	0.220635997	-0.031863842	-0.066805529	-0.187293919	-0.180286999	0.298990732	-0.176941875	-0.047003946	0.351629726	0.217416027	-0.063143756	-0.483091235	0.32704343	0.003731241	-0.037967447	-0.079515847	-0.110015482
TCGA.EJ.7317.11A.01R.2118.07	-0.2533	-0.047	0.143189094	0.328559006	0.130503595	-0.060117563	-0.055093175	-0.356161681	-0.192413079	0.29131152	-0.055467146	-0.076225257	0.372016323	0.250107087	-0.119618087	-0.244851877	0.307550082	-0.041465805	-0.102180263	-0.129742669	-0.106319736
TCGA.EJ.7782.11A.01R.2118.07	-0.0919	0.15577	0.125371519	0.374868584	0.197502944	-0.028875543	0.074338504	-0.163902396	-0.059174826	0.295494311	0.068815286	-0.094724409	0.367325665	0.303506993	-0.010461247	-0.273576143	0.326113004	0.048823715	0.042478815	-0.152703482	-0.064387114
TCGA.G9.6348.11A.01R.1789.07	-0.2077	0.01623	0.161448379	0.345265666	0.160367454	0.013260974	-0.026441749	-0.306157543	-0.20752785	0.243288163	-0.043853672	-0.142975542	0.355929048	0.285079141	-0.031923417	-0.241395613	0.354392779	0.012728292	-0.021325523	-0.161815783	-0.130655199
TCGA.EJ.7327.11A.01R.2118.07	-0.2845	-0.0102	0.118026328	0.316056077	0.151819543	-0.0219252	-0.09668454	-0.390951841	-0.21243576	0.262995224	-0.127025324	-0.176677507	0.356308244	0.190511484	-0.129080596	-0.267008691	0.291181123	-0.062578828	-0.10896427	-0.157359638	-0.120856022
TCGA.HC.8258.11A.01R.2263.07	-0.2621	0.00243	0.212877135	0.32325954	0.203778408	-0.027654386	-0.032478879	-0.210668779	-0.153563368	0.304926165	-0.165528183	-0.051925086	0.343109577	0.237732726	-0.077323582	-0.345136958	0.352622427	-0.011954834	-0.016174957	-0.091309536	-0.118548821
TCGA.HC.7747.11A.01R.2118.07	-0.2309	-0.0479	0.222568804	0.306559893	0.210246821	-0.029964626	-0.095660926	-0.312266247	-0.15324198	0.291421363	-0.138565792	-0.041687961	0.354330561	0.254638001	-0.087653919	-0.433072755	0.34453826	0.01069222	-0.027123386	-0.090568985	-0.132006367
TCGA.HC.7819.11A.01R.2118.07	-0.27	-0.0311	0.148320438	0.332314177	0.154704215	-0.02007433	-0.044776078	-0.373086991	-0.19933107	0.284786165	-0.145688096	-0.110454614	0.37640879	0.272251314	-0.106213782	-0.276761933	0.315141028	-0.034831453	-0.09755506	-0.147179541	-0.106616208
TCGA.HC.7752.11A.01R.2118.07	-0.1553	0.05514	0.161938036	0.363838393	0.191469773	-0.014818325	0.028107935	-0.361677041	-0.152764459	0.243370311	-0.056224133	-0.136906407	0.341924837	0.307965385	-0.018721655	-0.240605921	0.385171123	0.035981244	-0.006800184	-0.136096249	-0.113952945
TCGA.CH.5769.11A.01R.1580.07	-0.3933	-0.0095	0.120197674	0.337474707	0.158810244	-0.048524758	-0.031174953	-0.144194594	-0.152986447	0.21499629	-0.016488318	0.007645237	0.355034193	0.259149689	-0.073712477	-0.186353464	0.315501126	0.002297743	-0.085332802	-0.243253463	-0.145781376
TCGA.CH.5768.11A.01R.1580.07	-0.3797	-0.0304	0.141394746	0.310825484	0.15686702	0.021485894	-0.087799407	-0.284372389	-0.254657951	0.244389855	-0.195788381	-0.084950161	0.365690802	0.251155322	-0.125104797	-0.293163877	0.303912627	-0.059437048	-0.118710063	-0.130234667	-0.152322232
TCGA.HC.8259.11A.01R.2263.07	-0.2415	-0.0092	0.122441878	0.339950908	0.147463219	-0.022133708	-0.021167978	-0.277429919	-0.184465577	0.260175348	-0.022839393	-0.137657177	0.347564341	0.270745364	-0.098997126	-0.343034737	0.341604566	-0.005342031	-0.065780369	-0.173503064	-0.118083293
TCGA.HC.7745.11A.01R.2118.07	-0.293	-0.0365	0.202292299	0.333204945	0.17734154	-0.013592016	-0.027765915	-0.210744511	-0.126105315	0.283438231	-0.077796502	-0.009989555	0.321208258	0.284260778	-0.066106131	-0.42225509	0.385897414	0.046541797	-0.006827046	-0.088267499	-0.064926184
TCGA.EJ.7123.11A.01R.1965.07	-0.2277	-0.0548	0.094070672	0.346303507	0.217871873	0.077930031	0.039403587	-0.158597241	-0.095165834	0.28852106	0.131890822	-0.022916261	0.313514831	0.28784488	-0.007057481	-0.284974259	0.342191742	0.076628136	0.024609865	-0.187229473	-0.086863949
TCGA.EJ.7328.11A.01R.2118.07	-0.1964	-0.0122	0.137555804	0.377527005	0.205458461	0.022143227	0.043728925	-0.196707106	-0.098090644	0.270895789	0.051923516	-0.029559352	0.327995291	0.334672837	0.012105516	-0.287765326	0.378667908	0.10505585	0.03611328	-0.186741991	-0.060210145
TCGA.HC.7742.11A.01R.2118.07	-0.2804	0.00784	0.142915058	0.339751228	0.147555696	-0.044931038	-0.070043882	-0.307600356	-0.207791896	0.24742354	-0.018405656	-0.068101503	0.367223711	0.261842573	-0.101459351	-0.199602759	0.300685274	-0.027960363	-0.085483249	-0.17494287	-0.138355594
TCGA.EJ.7321.11A.01R.2263.07	-0.2079	0.06159	0.153690781	0.361660196	0.14203654	-0.047224337	0.013228424	-0.158490298	-0.152326473	0.268327476	0.069788634	-0.015904138	0.360198838	0.307142216	-0.102463692	-0.28596966	0.347424433	0.022523307	-0.044152018	-0.140894988	-0.087396783
TCGA.G9.6365.11A.01R.1789.07	-0.368	0.05122	0.16003092	0.347902899	0.162192635	-0.04376665	-0.046645449	-0.070505132	-0.251725688	0.249718239	-0.05763694	-0.022483004	0.358310667	0.264201799	-0.093100118	-0.316287987	0.306049285	-0.012079975	-0.057535151	-0.106255123	-0.128993155
TCGA.EJ.7330.11A.01R.2118.07	-0.2224	0.1041	0.160737274	0.401852945	0.249611359	0.029352627	0.081020375	-0.080131838	-0.054421708	0.274732978	0.188629695	0.016639784	0.345575257	0.346250481	0.059712353	-0.259178127	0.403993378	0.11801135	0.064249837	-0.169255517	-0.03754694
TCGA.G9.6356.11A.01R.1789.07	-0.3407	0.11266	0.179606915	0.343150255	0.206141473	-0.039397272	0.047715932	-0.103596393	-0.170853209	0.262627171	0.050820447	-0.047575347	0.365480664	0.265677948	-0.069080574	-0.30692215	0.300822336	-0.002323416	-0.051440566	-0.099601902	-0.125378348
TCGA.G9.6384.11A.01R.1858.07	-0.2262	-0.0282	0.157119277	0.339359369	0.141965887	-0.05438178	-0.036243403	-0.171340201	-0.229113788	0.268741961	0.12395491	-0.026994857	0.388325835	0.289760005	-0.005336081	-0.22861242	0.328028643	0.010999226	-0.003200734	-0.134619209	-0.112540148
TCGA.HC.7233.01A.11R.2118.07	-0.2597	0.11876	0.119038084	0.359183568	0.188754528	-0.027872627	0.06286761	-0.204446689	-0.049340536	0.235126753	-0.002955655	-0.080363482	0.307517904	0.257219512	-0.067093456	-0.228095109	0.304626834	0.064132453	-0.019927145	-0.153526812	-0.116071151
TCGA.EJ.7115.01A.11R.2118.07	-0.4063	0.00565	0.149792298	0.330747802	0.144192358	-0.065341341	-0.039561757	-0.309886644	-0.181646691	0.179847907	-0.038806629	-0.061718692	0.372563462	0.261979562	-0.107550557	-0.208485487	0.320714731	-0.026124693	-0.117597525	-0.151767199	-0.125455825
TCGA.EJ.A8FP.01A.21R.A36G.07	-0.2917	0.01503	0.173678289	0.356680397	0.134860603	-0.053160034	-0.000855977	-0.314440453	-0.201288578	0.264974877	-0.034862552	-0.068597812	0.353346624	0.311120317	-0.092834531	-0.243260707	0.363632061	0.022314266	-0.065673703	-0.158934963	-0.120847184
TCGA.EJ.5505.01A.01R.1580.07	-0.3776	-0.007	0.097203526	0.306461186	0.150654529	-0.042636529	-0.087475405	-0.411068469	-0.230631537	0.251758543	-0.075141826	-0.092435673	0.380097357	0.178661267	-0.138330296	-0.200584119	0.282351829	-0.076715044	-0.143345159	-0.151517271	-0.15762032
TCGA.CH.5738.01A.11R.1580.07	-0.3657	0.04769	0.118833296	0.369202516	0.258080826	-0.025189541	0.06722772	-0.00802121	-0.06946004	0.244692295	0.091444611	-0.004708076	0.313025421	0.281560728	-0.010057436	-0.163974942	0.361809001	0.117641622	0.028457446	-0.173983869	-0.079234737
TCGA.QU.A6IO.01A.11R.A31N.07	-0.1974	0.1383	0.189926244	0.261195018	0.124621238	-0.100049703	-0.01654254	-0.230690972	-0.211437117	0.158009851	-0.095816765	-0.151749007	0.374480653	0.178768554	-0.065793381	-0.228592157	0.303272682	-0.006459874	-0.081462216	-0.128075549	-0.226717819
TCGA.HC.7818.01A.11R.2118.07	-0.3019	0.00226	0.128337921	0.331996708	0.196283569	-0.055058241	-8.24E-05	-0.233151146	-0.165097815	0.225629663	-0.132908344	-0.069526675	0.36733802	0.239387095	-0.071653862	-0.175159604	0.316021676	0.002431756	-0.035367558	-0.162128257	-0.154681455
TCGA.CH.5771.01A.21R.1580.07	-0.2805	0.05977	0.16539396	0.372520908	0.193684752	-0.038606999	0.060451027	-0.158250637	-0.070704537	0.245324312	-0.006201585	-0.003635472	0.369119141	0.306947202	-0.013840678	-0.254568351	0.359317968	0.070995938	-0.00824786	-0.159533228	-0.128098956
TCGA.HC.A6AS.01A.11R.A30B.07	-0.2173	0.12177	0.164823566	0.320871032	0.161541466	-0.067813983	0.054251087	-0.118209667	-0.12735961	0.201447176	0.064244975	-0.096350465	0.367112125	0.254797252	-0.02412503	-0.247321826	0.338958611	0.052546217	-0.031015162	-0.079957295	-0.163552235
TCGA.XK.AAJ3.01A.11R.A41O.07	-0.3264	-0.008	0.092268277	0.289464696	0.162337922	-0.074256228	-0.1366379	-0.3746813	-0.179820353	0.157835833	-0.223889887	-0.106420272	0.382878746	0.198255612	-0.122606068	-0.158851183	0.278033739	-0.019683753	-0.116471356	-0.088040219	-0.199170192
TCGA.EJ.5512.01A.01R.1580.07	-0.3137	0.01273	0.090138475	0.326379621	0.127946704	-0.031103491	-0.068051626	-0.30280309	-0.223931275	0.24122436	-0.113365276	-0.077349045	0.347760447	0.24935645	-0.109760924	-0.173785135	0.306075219	-0.050044363	-0.087077754	-0.161626517	-0.152948221
TCGA.H9.A6BY.01A.11R.A30B.07	-0.3922	0.02562	0.123822727	0.250565105	0.107999852	-0.063533722	-0.075962031	-0.430002561	-0.326276245	0.2008678	-0.201082753	-0.117313845	0.367147856	0.133420023	-0.153697402	-0.20284655	0.290515233	-0.064805567	-0.139395538	-0.204615898	-0.254676021
TCGA.HC.8264.01B.11R.2403.07	-0.3986	-0.0124	0.07361354	0.329261242	0.165932316	-0.000608366	0.020274627	-0.379823447	-0.158016787	0.204330988	-0.00768349	-0.079382298	0.365421457	0.280339915	-0.069744846	-0.276787277	0.34779154	-0.00353423	-0.065237186	-0.16747736	-0.140788609
TCGA.G9.6369.01A.21R.1965.07	-0.3956	0.04713	0.103012204	0.281627949	0.116656412	-0.058314624	-0.122810177	-0.302579221	-0.275671166	0.133160688	-0.178057163	-0.193925139	0.368707345	0.152235402	-0.107169499	-0.158648918	0.283524044	-0.032311104	-0.108993489	-0.143321386	-0.166623816
TCGA.EJ.A7NM.01A.21R.A33R.07	-0.1915	0.15427	0.090195216	0.329406732	0.206620907	-0.002180872	-0.010677564	-0.360809227	-0.105668224	0.159596943	-0.163333977	-0.045903928	0.368725967	0.255843071	-0.075586834	-0.27392653	0.31737267	-0.01102034	-0.04198992	-0.176314182	-0.097000993
TCGA.KK.A59Y.01A.11R.A26U.07	-0.3513	-0.0031	0.125622208	0.273852225	0.12202297	-0.051338214	-0.090203365	-0.23151087	-0.245610221	0.237782481	-0.148501646	-0.18831535	0.363182586	0.143581293	-0.117348985	-0.161059088	0.330753854	-0.054321741	-0.107678452	-0.181461874	-0.21054339
TCGA.QU.A6IM.01A.11R.A31N.07	-0.2636	0.24151	0.193684776	0.266606735	0.138781519	-0.09869929	0.039343406	-0.175265273	-0.227685801	0.180765017	-0.075169001	-0.129987205	0.375406355	0.210984219	-0.080911277	-0.154866877	0.307694981	-0.011132807	-0.022535565	-0.095189211	-0.221802252
TCGA.ZG.A8QZ.01A.11R.A37L.07	-0.3608	0.07428	0.141402682	0.356527332	0.157452548	-0.0097729	0.021517293	-0.147008948	-0.175434687	0.248742117	0.008246387	-0.010190182	0.292976895	0.289503311	-0.049363657	-0.263522769	0.340438808	0.044655144	-0.032731256	-0.203746265	-0.111927376
TCGA.HC.8261.01A.11R.2263.07	-0.3101	0.05651	0.09225264	0.300764174	0.171084187	-0.061666263	-0.028460566	-0.423556461	-0.117227619	0.182435086	-0.209088389	-0.103287437	0.380607796	0.245031455	-0.106514664	-0.184294086	0.279587607	-0.035500071	-0.102405902	-0.167266131	-0.181840911
TCGA.EJ.8469.01A.11R.2403.07	-0.4093	0.00728	0.050034967	0.270151452	0.126558276	0.079099579	-0.016031168	-0.353860497	-0.239525592	0.213723456	-0.240706015	-0.042545016	0.304889387	0.134702696	-0.096630547	-0.214401023	0.30272871	-0.087888636	-0.138027809	-0.29234013	-0.137073921
TCGA.2A.A8W3.01A.11R.A37L.07	-0.3803	-0.0124	0.147138421	0.300068691	0.12522556	-0.034987121	-0.039028796	-0.26434507	-0.269203612	0.224586252	-0.081759442	-0.100587219	0.344759016	0.188580151	-0.081567614	-0.221110359	0.327568698	-0.033316618	-0.107093885	-0.247658031	-0.24964019
TCGA.J9.A8CL.01A.11R.A352.07	-0.3401	0.04446	0.140353837	0.349720241	0.166599609	0.009972134	0.06074332	-0.503722568	-0.062837574	0.201227505	-0.191549283	-0.040307404	0.347547277	0.226569776	-0.019098527	-0.315840882	0.310063243	0.006173773	-0.067541443	-0.172162256	-0.213589032
TCGA.KK.A7AQ.01A.11R.A33R.07	-0.3349	0.0253	0.164876058	0.292281038	0.149915943	-0.100046368	-0.065697332	-0.334844264	-0.160619644	0.153717213	-0.195976632	-0.131079337	0.392563882	0.202888368	-0.08388738	-0.233362401	0.309781407	-0.029222424	-0.098922649	-0.19715819	-0.197697729
TCGA.KK.A8IH.01A.11R.A36G.07	-0.265	0.03275	0.076463913	0.317009345	0.130958257	-0.066539847	-0.016389721	-0.176603458	-0.195370812	0.199776362	-0.10494802	-0.120229593	0.334578818	0.151010369	-0.100035482	-0.267227874	0.324462002	-0.019192547	-0.066632665	-0.170849842	-0.173510445
TCGA.VP.A87B.01A.11R.A352.07	-0.4361	-0.0144	0.134988906	0.299651598	0.151384322	-0.068837263	-0.103255735	-0.330907149	-0.140419728	0.117501777	-0.187034377	-0.081359472	0.367918829	0.173041697	-0.096529823	-0.193386638	0.295950954	-0.025053199	-0.090196541	-0.200558962	-0.237032777
TCGA.EJ.5494.01A.01R.1580.07	-0.3343	0.01625	0.093931333	0.355678082	0.196214377	-0.056374994	-0.02092399	-0.132238714	-0.248706832	0.207878604	-0.026798878	-0.084881842	0.355165652	0.223187763	-0.045266273	-0.336185155	0.264496078	0.012415688	-0.062525422	-0.242913267	-0.193218342
TCGA.EJ.5502.01A.01R.1580.07	-0.2983	0.01688	0.139368085	0.347563105	0.178520364	-0.033284654	0.033231528	-0.251233597	-0.165059113	0.206840623	-0.066258622	-0.089000218	0.368684597	0.304483407	-0.066782495	-0.21448821	0.353469901	0.026641504	-0.020040901	-0.152231882	-0.132923644
TCGA.KC.A7FD.01A.11R.A33R.07	-0.4527	0.02435	0.075572807	0.27646834	0.128372418	-0.062112083	-0.100127412	-0.3194901	-0.294725355	0.184202368	-0.11898141	-0.150314642	0.368940996	0.143602423	-0.085884203	-0.246361715	0.299380996	-0.008790189	-0.085715315	-0.162692575	-0.19287183
TCGA.VN.A943.01A.11R.A41O.07	-0.4012	-0.0075	0.088097554	0.238283528	0.091308819	-0.043367065	-0.183072077	-0.229456508	-0.326700873	0.230857702	-0.238361338	-0.168065247	0.370886883	0.087894712	-0.168786725	-0.217776457	0.246568549	-0.058104426	-0.149075864	-0.211493243	-0.183675943
TCGA.EJ.5515.01A.01R.1580.07	-0.3001	-0.0101	0.107194645	0.340661111	0.136887109	-0.026000109	-0.075689021	-0.264223893	-0.152618579	0.285125602	-0.026412998	-0.084696985	0.374995263	0.263312638	-0.119366098	-0.258588875	0.323159958	-0.066946498	-0.096519015	-0.132646456	-0.128391318
TCGA.V1.A8WW.01A.11R.A37L.07	-0.3973	0.06494	0.126218295	0.332298162	0.133433605	-0.056865886	-0.040787626	-0.362515728	-0.122962732	0.174607367	-0.217214628	-0.107836751	0.366950073	0.233818823	-0.079372134	-0.098691828	0.288524342	-0.045245773	-0.064717946	-0.207957252	-0.17952444
TCGA.EJ.7218.01B.11R.A32O.07	-0.2807	0.01907	0.123373985	0.27883878	0.120413458	-0.068605094	-0.079492283	-0.340977394	-0.197042245	0.202773068	-0.195588436	-0.15900829	0.398592697	0.185299498	-0.129303238	-0.321287001	0.31089691	-0.018318823	-0.095521637	-0.214438445	-0.206896076
TCGA.V1.A9OO.01A.11R.A41O.07	-0.2265	0.1205	0.131821771	0.308592604	0.135461231	-0.039431817	-0.041615711	-0.265857021	-0.199996184	0.208326541	-0.099059791	-0.167515246	0.386475062	0.223461562	-0.076679379	-0.212464747	0.331540091	-0.00104892	-0.051091256	-0.187813463	-0.192399353
TCGA.V1.A8MK.01A.11R.A36G.07	-0.3097	0.00888	0.138901213	0.330319361	0.131448207	-0.066704736	-0.037821845	-0.308390836	-0.245372753	0.230729096	-0.108314735	-0.125525755	0.37321689	0.256254837	-0.099686349	-0.260279555	0.331788467	-0.00381766	-0.074929068	-0.179834473	-0.157821183
TCGA.EJ.7793.01A.31R.2263.07	-0.3455	-0.0156	0.12138937	0.31210165	0.123935698	-0.055346109	-0.036221872	-0.397936634	-0.224704691	0.252476805	-0.171563758	-0.076351883	0.38399128	0.242426484	-0.103224028	-0.129696568	0.287205945	-0.062040974	-0.09614256	-0.167426039	-0.174479734
TCGA.G9.7510.01A.11R.2263.07	-0.318	0.02985	0.152520195	0.336097642	0.173884566	-0.068284333	0.004722816	-0.145557549	-0.124639925	0.213546505	-0.025961625	-0.111867036	0.359093636	0.2496111	-0.039691317	-0.212502644	0.315321331	0.03168218	-0.037941568	-0.149828554	-0.123969728
TCGA.J4.A67R.01A.21R.A30B.07	-0.1594	0.13899	0.190301148	0.319196527	0.134585468	-0.045710788	0.038344423	-0.177161486	-0.179081742	0.195232657	-0.059886125	-0.059991122	0.380047599	0.264142807	-0.071970345	-0.160368922	0.349765926	0.035138745	-0.014132042	-0.136291976	-0.174559807
TCGA.J4.A83L.01A.11R.A352.07	-0.3845	-0.0341	0.120635724	0.328346909	0.142878501	-0.064429401	-0.104872054	-0.182489774	-0.229893559	0.223020722	0.041220134	-0.158046139	0.369318947	0.233738923	-0.0759275	-0.216921044	0.290862915	-0.006318641	-0.040510047	-0.176544449	-0.190210403
TCGA.H9.A6BX.01A.31R.A311.07	-0.1811	0.0136	0.162636273	0.373934778	0.191369188	-0.03188784	0.034905124	-0.340456232	-0.159232051	0.260956287	-0.047974823	-0.068731152	0.345233743	0.317186827	-0.021839676	-0.308988945	0.382448174	0.043582368	0.006132035	-0.176956041	-0.117536422
TCGA.CH.5790.01A.11R.1580.07	-0.4437	0.00022	0.069348832	0.311237601	0.163151147	-0.055453621	-0.048389604	-0.1891002	-0.188286158	0.190418872	-0.119350676	-0.081235338	0.36877748	0.198728985	-0.105184588	-0.20591979	0.303115522	-0.028124562	-0.095046594	-0.221810134	-0.222777158
TCGA.HC.8259.01A.11R.2263.07	-0.3666	0.00649	0.132964758	0.268000838	0.115427949	-0.01647875	-0.079210915	-0.381360749	-0.213949415	0.235384359	-0.280227145	-0.104333523	0.389369463	0.173832674	-0.16008186	-0.199116142	0.28817878	-0.058218285	-0.118150791	-0.230395274	-0.214753713
TCGA.ZG.A9L6.01A.11R.A41O.07	-0.3065	0.02416	0.165094902	0.357021958	0.173041901	-0.028621135	0.011533896	-0.301945474	-0.135752424	0.236350238	-0.038100632	-0.138489929	0.353130861	0.294232345	-0.01978922	-0.255759513	0.362328704	0.046761395	-0.027436936	-0.146956288	-0.148504402
TCGA.VN.A88Q.01A.11R.A352.07	-0.4543	-0.052	0.132173739	0.286074236	0.129659206	-0.044108441	-0.062263541	-0.182962763	-0.280658926	0.178512685	-0.116232302	-0.120953877	0.376865055	0.137777335	-0.109866977	-0.136139495	0.274956718	-0.049520166	-0.096211073	-0.235345209	-0.154515855
TCGA.KC.A4BN.01A.61R.A250.07	-0.347	-0.0149	0.136941638	0.303628155	0.129313846	-0.08007046	-0.057171479	-0.18055233	-0.271024563	0.211901057	-0.111485859	-0.110037299	0.415526822	0.171375405	-0.095544099	-0.167940343	0.286813019	-0.024811324	-0.061307888	-0.1768767	-0.13842841
TCGA.J9.A52D.01A.11R.A29R.07	-0.4999	0.00749	-0.003030093	0.265770326	0.162467404	-0.041201603	-0.209878441	-0.414567217	-0.167516413	0.151817882	-0.328146278	-0.0595567	0.3265921	0.085958663	-0.16528835	-0.137928779	0.259359766	-0.077521044	-0.142341242	-0.155308762	-0.148273024
TCGA.HC.7209.01A.11R.2118.07	-0.3662	-0.0019	0.136716929	0.288673948	0.156134337	-0.058202914	-0.029334309	-0.108198099	-0.22534467	0.246950983	0.009751315	-0.058372872	0.370793884	0.240418539	-0.07940243	-0.087805156	0.293913303	0.004017832	-0.061789257	-0.17114067	-0.124450205
TCGA.CH.5750.01A.11R.1580.07	-0.1963	0.13117	0.092429075	0.324260747	0.163113887	-0.030510097	0.014174791	-0.104177379	-0.108373768	0.218080966	-0.122723686	-0.097898783	0.364120049	0.210629046	-0.078888308	-0.294153101	0.314613385	0.006544014	-0.053765246	-0.223970367	-0.177679125
TCGA.G9.6356.01A.11R.1789.07	-0.3242	0.09344	0.166224629	0.36001434	0.178768062	0.000506441	0.022739088	-0.244075437	-0.141195148	0.244370399	-0.097025829	-0.034532279	0.379751302	0.274954308	-0.051338825	-0.163976972	0.356914277	0.009255036	-0.051846124	-0.141738216	-0.145247451
TCGA.ZG.A9KY.01A.11R.A41O.07	-0.2224	0.25466	0.144879625	0.302656093	0.142624462	0.020889525	0.033390775	-0.335125398	-0.119637875	0.183679458	-0.228425215	-0.087444234	0.366048156	0.209312904	-0.084540829	-0.325534631	0.320365044	-0.000502715	-0.071311632	-0.24473652	-0.139875889
TCGA.HC.7078.01A.11R.2118.07	-0.3424	0.03844	0.158715536	0.314329032	0.174894502	-0.04464571	-0.014454268	-0.325653346	-0.151408692	0.189289136	-0.089130669	-0.12807466	0.363429682	0.226547711	-0.093814682	-0.184716758	0.305251539	-0.01375851	-0.085598941	-0.137612293	-0.204375888
TCGA.G9.6362.01A.11R.1789.07	-0.3996	0.03115	0.115050313	0.29108535	0.135057401	-0.04190933	-0.042170393	-0.369348971	-0.248089244	0.185570194	-0.168123703	-0.232981652	0.334250328	0.197519499	-0.087885804	-0.026464578	0.305562395	-0.031230375	-0.094907424	-0.223135347	-0.197410372
TCGA.EJ.7327.01A.11R.2118.07	-0.3093	-0.0248	0.094094206	0.325179754	0.185322285	-0.005021262	0.020307685	-0.283931513	-0.092906662	0.216265378	-0.17332014	0.00608191	0.371996052	0.259654256	-0.096420336	-0.206325672	0.327252494	-0.004253199	-0.089079138	-0.221778595	-0.168344673
TCGA.XK.AAIV.01A.11R.A41O.07	-0.1703	0.22819	0.141955188	0.389056179	0.221654964	-0.024467978	0.081657992	-0.316015229	-0.022678775	0.179113974	-0.048049212	0.011536128	0.343582109	0.254726448	0.003985834	-0.249138659	0.324935792	0.077177183	0.006412797	-0.174765832	-0.115684528
TCGA.M7.A720.01A.12R.A32O.07	-0.2657	0.00048	0.151332896	0.332751158	0.139304677	-0.041072322	-0.031335377	-0.288899775	-0.2157024	0.23863655	-0.08819914	-0.077452372	0.350759306	0.288710676	-0.127987414	-0.2696255	0.322180677	-0.052194759	-0.102587272	-0.154706759	-0.185428525
TCGA.V1.A9ZR.01A.11R.A41O.07	-0.2795	0.06712	0.153929258	0.366784383	0.173669004	-0.029858444	0.060964462	-0.288448463	-0.115310096	0.233473179	-0.087807432	-4.19E-05	0.342511306	0.291930457	-0.012784701	-0.337573428	0.375506388	0.042290185	-0.019388507	-0.238729758	-0.170122066
TCGA.EJ.8468.01A.21R.2403.07	-0.3834	0.06032	0.05920135	0.347379692	0.171228232	-0.018299517	0.031674516	-0.10384782	-0.055681182	0.183774865	0.074829311	-0.091630349	0.358848593	0.25425494	-0.056380265	-0.203471124	0.306504501	0.041572234	-0.055550986	-0.147364864	-0.097754195
TCGA.ZG.A9N3.01A.11R.A41O.07	-0.0822	0.12504	0.086541578	0.321314064	0.146985525	0.026364596	-0.057213108	-0.104455096	-0.120408984	0.15961892	-0.071244096	0.026115446	0.276975944	0.233727	-0.083415824	-0.232935856	0.274002496	0.029038203	-0.032843459	-0.206133995	-0.118980592
TCGA.X4.A8KS.01A.12R.A36G.07	-0.2594	0.09004	0.140084415	0.313446264	0.148249823	-0.049066382	-0.073197753	-0.25045703	-0.23067456	0.226632063	-0.041278348	-0.148629644	0.370102688	0.230274476	-0.085452263	-0.222311708	0.354742228	-0.035155546	-0.077604292	-0.093184847	-0.163962827
TCGA.HC.7819.01A.11R.2118.07	-0.3964	-0.0074	0.081464621	0.269468274	0.115911053	-0.026560428	-0.070581072	-0.187473205	-0.260022898	0.198467718	-0.216242888	-0.10786725	0.353246921	0.124349254	-0.132649843	-0.176482788	0.298689627	-0.049000232	-0.101178287	-0.184716358	-0.175324873
TCGA.ZG.A9LN.01A.11R.A41O.07	-0.0716	0.34642	0.112355316	0.411133831	0.157865069	-0.007269568	0.110207104	-0.103180328	0.004401536	0.215596671	0.038421488	-0.011161581	0.354983321	0.312967555	0.004717732	-0.260502358	0.34558347	0.078170273	0.069537777	-0.211275243	-0.074432191
TCGA.HC.8260.01A.11R.2263.07	-0.3015	0.00295	0.140186944	0.339485875	0.141501013	-0.049725085	0.003881084	-0.285305224	-0.20829512	0.246879183	-0.097453957	-0.078280403	0.370260345	0.23024548	-0.137369908	-0.296854723	0.316734848	-0.042228027	-0.094082002	-0.184868064	-0.17299148
TCGA.XJ.A83G.01A.11R.A352.07	-0.4051	0.03699	0.141022909	0.274405368	0.138369687	-0.086616826	-0.0861756	-0.31957034	-0.259131107	0.188434164	-0.130750578	-0.156471363	0.383839855	0.144404181	-0.130122041	-0.214305407	0.312945964	-0.050485831	-0.116582223	-0.176135586	-0.208818435
TCGA.KK.A8IL.01A.11R.A36G.07	-0.2877	0.13745	0.16983641	0.383473152	0.194621892	-0.053989547	0.103969275	-0.191654573	-0.122429229	0.227765662	-0.031093297	-0.089887763	0.344416807	0.241013094	0.002643135	-0.146018865	0.337239266	0.053307366	-0.011068741	-0.144075559	-0.143575345
TCGA.J9.A8CM.01A.11R.A352.07	-0.3738	0.01969	0.15399363	0.335192213	0.151022091	-0.044964899	-0.015399429	-0.292257888	-0.150011335	0.21072071	-0.097218928	-0.00273209	0.330232295	0.239230412	-0.049742074	-0.26088626	0.342397229	-0.042581764	-0.048093571	-0.257025905	-0.153290379
TCGA.EJ.7781.01A.11R.2118.07	-0.2811	0.0031	0.101700244	0.319835622	0.180519765	-0.066305432	-0.062896763	-0.231982156	-0.151992213	0.24385051	-0.073723487	-0.070119861	0.357408863	0.274320709	-0.10712007	-0.295479005	0.318281827	-0.018447959	-0.080308044	-0.181735013	-0.218660321
TCGA.V1.A9OH.01A.11R.A41O.07	-0.3624	0.025	0.164190476	0.319979989	0.151632078	-0.042329991	-0.037681036	-0.347448923	-0.21636137	0.2002895	-0.195851815	-0.069895429	0.370858694	0.217382676	-0.085909492	-0.232558717	0.315194123	-0.020034152	-0.079108735	-0.193716568	-0.178035577
TCGA.WW.A8ZI.01A.11R.A37L.07	-0.3323	0.03456	0.136957093	0.28148756	0.168171735	-0.024061481	-0.060107563	-0.407324072	-0.270205796	0.209311395	-0.158424892	-0.134272507	0.320134912	0.142187922	-0.110871319	-0.268016296	0.245383372	-0.079730726	-0.127341317	-0.279125604	-0.255178446
TCGA.XK.AAIP.01A.11R.A41O.07	-0.4489	0.00474	0.131050517	0.229380275	0.154818199	-0.018066428	0.012755272	-0.425005142	-0.153697248	0.170242108	-0.221273405	-0.058497843	0.361921726	0.197102842	-0.089964585	-0.176690383	0.308290018	-0.038671383	-0.11974389	-0.164321604	-0.126266339
TCGA.CH.5754.01A.11R.1580.07	-0.2169	0.14864	0.162208571	0.356401002	0.174913659	-0.085784558	0.042414049	-0.200416508	-0.043830174	0.176975578	0.016727881	-0.062590043	0.314681667	0.226573223	-0.054074167	-0.155975849	0.31827419	0.054565575	-0.041572165	-0.171841757	-0.125013202
TCGA.2A.A8VV.01A.11R.A37L.07	-0.4117	0.01454	0.121360464	0.296741823	0.110953033	-0.051875773	-0.104785183	-0.354256578	-0.304893804	0.215159412	-0.1677983	-0.10828192	0.376897333	0.193775082	-0.140052768	-0.165142884	0.334234105	-0.044827835	-0.087525706	-0.218576819	-0.22367695
TCGA.V1.A8MG.01A.11R.A36G.07	-0.2832	0.0818	0.10235512	0.321990078	0.151742487	-0.052992281	-0.028902455	-0.370927331	-0.221962765	0.185515223	-0.180646656	-0.110200635	0.356431747	0.235659163	-0.110043675	-0.290703414	0.31616889	-0.028316295	-0.084932357	-0.193471178	-0.166581046
TCGA.EJ.A65B.01A.12R.A30B.07	-0.2875	0.03028	0.176066445	0.331592084	0.146078824	-0.050778589	-0.034637231	-0.320663475	-0.227164255	0.230921761	-0.085779167	-0.109666365	0.371672874	0.201053559	-0.051194785	-0.201396801	0.335201957	0.027769378	-0.089338581	-0.085286173	-0.169627688
TCGA.HC.8262.01A.11R.2263.07	-0.3812	0.01274	0.11986944	0.300296426	0.142828882	-0.028936229	-0.044429876	-0.401926421	-0.143318821	0.223776925	-0.22739474	-0.037442058	0.379326572	0.218940181	-0.159925697	-0.20648914	0.31168356	-0.057907551	-0.119497473	-0.13891113	-0.117550454
TCGA.J4.A83I.01A.11R.A352.07	-0.3187	-0.0229	0.13109843	0.31805896	0.129435848	-0.017204236	-0.04780973	-0.274622011	-0.254678823	0.232563152	-0.10817914	-0.097100993	0.363774439	0.225574747	-0.1023447	-0.209465726	0.32348788	-0.056389308	-0.074125	-0.208116837	-0.222332833
TCGA.KK.A8I8.01A.11R.A36G.07	-0.3738	0.05855	0.09567946	0.278934386	0.131221928	-0.02364648	-0.042077228	-0.374800941	-0.218451428	0.213854731	-0.270019919	-0.102518296	0.325855875	0.171270244	-0.11105805	-0.235169272	0.308467265	-0.042041387	-0.126031727	-0.270732344	-0.248571977
TCGA.EJ.5495.01A.11R.1580.07	-0.2653	0.04494	0.144458811	0.331476897	0.21237737	-0.034263561	0.068458773	-0.12784367	-0.212190875	0.21879543	-0.06466855	0.208601679	0.320785674	0.18144875	-0.098940875	-0.209241008	0.34678579	0.042070751	-0.016022204	-0.17303743	-0.075838604
TCGA.V1.A8MU.01A.11R.A37L.07	-0.2406	0.1312	0.175430876	0.357381729	0.150507199	-0.037479479	0.016372168	-0.35871876	-0.17950627	0.199348197	-0.052376762	-0.114408915	0.30785634	0.2862386	-0.042761323	-0.167959015	0.369644076	0.036630658	-0.044906357	-0.179903989	-0.138417288

TCGA.YL.A8SP.01B.11R.A37L.07	-0.3581	-0.0695	0.124669347	0.238251953	0.129733605	-0.014091704	-0.070428111	-0.164772867	-0.338388497	0.175350255	-0.043847306	-0.127830381	0.319381037	0.098052369	-0.137118175	-0.21648127	0.280763895	-0.043421674	-0.137676737	-0.289119769	-0.264185146
TCGA.EJ.7312.01B.21R.A32O.07	-0.4195	0.00358	0.13661143	0.270241935	0.17308347	-0.034645305	-0.075287195	-0.426399301	-0.246350346	0.187979739	-0.202066767	-0.203217488	0.379655057	0.14470482	-0.115208543	-0.283451456	0.284663613	-0.042534658	-0.161972169	-0.156211187	-0.242330474
TCGA.G9.6378.01A.11R.1789.07	-0.3446	0.01705	0.108966294	0.323256961	0.207045344	-0.022768963	-0.097065087	-0.283748595	-0.1192062	0.2288675	-0.104068307	-0.170639176	0.341990324	0.258473619	-0.08431664	-0.28585422	0.312779581	-0.029168872	-0.063401586	-0.156280634	-0.20132664
TCGA.G9.7523.01A.11R.2263.07	-0.2542	0.13524	0.147745199	0.380718681	0.218658038	0.07128621	0.085495017	-0.189937907	-0.098361146	0.239024502	0.057077062	-0.055388121	0.331768756	0.340569081	0.062341009	-0.271883403	0.364395319	0.1021452	0.040174566	-0.130590831	-0.084632268
TCGA.G9.6364.01A.21R.1789.07	-0.299	0.00064	0.113624947	0.294402943	0.163293164	-0.032990683	-0.073236035	-0.226684015	-0.245941866	0.252668205	-0.056939116	-0.110902414	0.344736671	0.2474416	-0.065395506	-0.076699556	0.335184932	-0.019800921	-0.065860266	-0.165795049	-0.167587263
TCGA.V1.A8MF.01A.11R.A36G.07	-0.3284	0.12235	0.230542207	0.347953797	0.162835388	-0.038024586	0.030911057	-0.191037235	-0.153853032	0.23724601	0.034788532	-0.090909292	0.350089789	0.225229772	-0.047881747	-0.294326346	0.323719774	0.038188801	-0.063791481	-0.143928594	-0.211831371
TCGA.V1.A9Z9.01A.21R.A41O.07	-0.4196	0.00611	0.158623616	0.320511837	0.153993009	-0.056801891	-0.013469245	-0.405752286	-0.206555868	0.201363061	-0.189592469	-0.13941851	0.359636527	0.217992855	-0.067458019	-0.198619535	0.318524043	-0.00229351	-0.094725821	-0.163245614	-0.160627555
TCGA.KK.A8I6.01A.11R.A36G.07	-0.3896	0.01975	0.139227018	0.29314357	0.135149295	-0.049508494	-0.035452165	-0.388003357	-0.25165911	0.209545427	-0.195695682	-0.080477051	0.392340707	0.209734965	-0.134434203	-0.174655929	0.319986983	-0.038194054	-0.126991047	-0.211778559	-0.198024757
TCGA.M7.A723.01A.12R.A32O.07	-0.2225	0.18463	0.150383745	0.3481387	0.162781184	-0.052085387	0.043106863	-0.360877438	-0.097101569	0.2294078	-0.109415704	-0.09091092	0.360754833	0.261357864	-0.019745257	-0.180096612	0.337549776	0.055660419	-0.018332298	-0.174304972	-0.193493239
TCGA.EJ.5509.01A.01R.1580.07	-0.3156	0.05098	0.143569417	0.358039601	0.180881239	-0.059971056	0.016286895	-0.305618631	-0.149014112	0.180940426	-0.09515666	-0.051671053	0.395114305	0.296844067	-0.033685267	-0.233526276	0.331480791	0.031787849	-0.052730227	-0.142391519	-0.141275285
TCGA.KC.A7F3.01A.21R.A33R.07	-0.3347	0.01124	0.123617501	0.323607393	0.157563136	-0.050539108	-0.047866011	-0.20472012	-0.264909979	0.228054264	-0.077902331	-0.084051287	0.365401667	0.224647112	-0.067555551	-0.184882698	0.311189384	0.014612374	-0.040694538	-0.150399195	-0.12431209
TCGA.CH.5737.01A.11R.1580.07	-0.3527	0.00151	0.121011684	0.297934682	0.14758478	-0.051026082	-0.124984944	-0.162958919	-0.228400117	0.20359398	-0.123729507	-0.136350164	0.423720775	0.2309023	-0.124202801	-0.21433809	0.286079274	0.021733615	-0.083020646	-0.203991327	-0.133957358
TCGA.G9.6377.01A.11R.1965.07	-0.3666	0.07661	0.117448612	0.320534315	0.164456682	-0.054805065	-0.019065975	-0.147686992	-0.206537434	0.215108976	-0.071479998	-0.131070473	0.355906082	0.199917973	-0.062776899	-0.201083478	0.316907367	0.007680614	-0.04482175	-0.138261133	-0.198478235
TCGA.EJ.AB27.01A.11R.A41O.07	-0.1343	0.12197	0.128609	0.339325256	0.139936952	-0.050338538	-0.050105082	-0.205669249	-0.135411871	0.238270331	0.005647298	-0.133189484	0.364944874	0.245223973	-0.129828327	-0.248163588	0.332458865	-0.025054536	-0.05838607	-0.159969539	-0.148673844
TCGA.EJ.7782.01A.11R.2118.07	-0.3992	-0.0089	0.139958231	0.312502641	0.132428743	-0.038856518	-0.060991819	-0.315002828	-0.121014396	0.159741489	-0.080143912	-0.09281462	0.38370558	0.280357576	-0.086851661	-0.296470552	0.318856954	-0.04056815	-0.128065691	-0.21463865	-0.136545914
TCGA.EJ.5496.01A.01R.1580.07	-0.3783	0.00388	0.082973437	0.28318971	0.121920513	-0.022107969	-0.054112554	-0.321318213	-0.235998921	0.247883096	-0.205126218	-0.080919711	0.391100335	0.182333018	-0.132116081	-0.197533692	0.288651111	-0.081007694	-0.128452372	-0.217967455	-0.18162733
TCGA.EJ.A46G.01A.31R.A26U.07	-0.3218	-0.0167	0.18958273	0.302514022	0.142942617	-0.102412316	-0.060515218	-0.277581241	-0.204919134	0.138662422	-0.126507716	-0.098425773	0.376747394	0.203422862	-0.092195517	-0.221412343	0.31030314	-0.022708725	-0.073073829	-0.092258483	-0.155960849
TCGA.EJ.A8FS.01A.11R.A352.07	-0.4658	0.01504	0.141084554	0.274636072	0.115814245	-0.041781087	-0.126001584	-0.394388583	-0.110967601	0.175571865	-0.239874132	-0.076828932	0.374110693	0.142147411	-0.129297951	-0.228553421	0.290518501	-0.098135656	-0.165098567	-0.146334343	-0.216951942
TCGA.CH.5766.01A.11R.1580.07	-0.359	0.06699	0.131325953	0.328002186	0.146307094	-0.01242804	-0.027087976	-0.048053836	-0.160468026	0.244189607	-0.03564429	-0.101976642	0.332613752	0.240268724	-0.092334765	-0.277740302	0.287375083	0.015109341	-0.061311371	-0.16363562	-0.173465065
TCGA.CH.5792.01A.11R.1580.07	-0.1661	0.09449	0.158718329	0.389489537	0.201092271	-0.057272897	0.075318857	-0.254498823	-0.088503249	0.195345623	-0.017310239	0.006111399	0.39018447	0.312479924	-0.019409314	-0.191245474	0.355324452	0.06086973	-0.005485408	-0.162701112	-0.141001528
TCGA.CH.5743.01A.21R.1580.07	-0.2407	0.09822	0.171254964	0.370900492	0.221834924	0.022054739	0.153088658	-0.049808282	-0.07499035	0.276989889	0.0429587	0.050128357	0.344199309	0.345559474	-0.017386509	-0.252061794	0.355553847	0.049601629	0.024414777	-0.100601967	-0.086603761
TCGA.ZG.A9L4.01A.11R.A41O.07	-0.3793	0.02931	0.160095761	0.336712579	0.169186704	0.003441273	0.005023379	-0.08130079	-0.241103145	0.226531399	0.05541761	-0.06374538	0.338426483	0.258535371	0.00151548	-0.240881468	0.358190259	0.046962333	0.011385156	-0.153576568	-0.096033854
TCGA.H9.7775.01A.11R.2118.07	-0.4577	-0.0273	0.109846475	0.315565652	0.166832371	-0.058463328	-0.069717178	-0.360396647	-0.160720571	0.243287839	-0.170578785	-0.100265063	0.370428378	0.201029127	-0.080998405	-0.251445123	0.3298373	-0.02995481	-0.092253638	-0.196462626	-0.209687507
TCGA.J4.8200.01A.11R.A29R.07	-0.3966	-0.005	0.134168946	0.335899685	0.159914701	-0.000478403	-0.058974718	-0.19439611	-0.120734376	0.208352124	-0.041412521	-0.15433733	0.343544161	0.244661771	-0.044448746	-0.316328759	0.311293927	0.026588186	-0.040886025	-0.182410806	-0.168197832
TCGA.EJ.5497.01A.02R.1580.07	-0.3444	0.00752	0.106769574	0.32916329	0.150881262	-0.000562582	0.004922164	-0.188945376	-0.188809976	0.225923997	-0.030335937	-0.029450764	0.365184583	0.233554609	-0.100811049	-0.185474089	0.321312253	0.000820973	-0.062840086	-0.177506183	-0.140621621
TCGA.VP.A87E.01A.31R.A352.07	-0.2367	0.15683	0.158646506	0.373140964	0.183481874	0.007975255	0.06240294	-0.039799547	-0.09893102	0.215037966	0.028392612	-0.11963398	0.344748309	0.311744805	-0.011931458	-0.187450562	0.343255202	0.094235235	0.086809712	-0.114314656	-0.099155625
TCGA.HC.8258.01A.11R.2263.07	-0.2934	0.02942	0.159730816	0.31762358	0.138485464	-0.061136389	-0.020401558	-0.22917243	-0.175380383	0.22541599	-0.05119177	-0.096697809	0.378942384	0.245338818	-0.126077211	-0.30532085	0.317041274	-0.03163552	-0.068326132	-0.15605602	-0.145548497
TCGA.G9.6336.01A.11R.1789.07	-0.3004	0.00749	0.172675604	0.346324734	0.15404613	-0.038759398	-0.026726664	-0.218093305	-0.225465705	0.245425743	-0.081867172	-0.09662044	0.382177111	0.225285854	-0.085446703	-0.200687868	0.304012902	-0.025674463	-0.074855549	-0.158642328	-0.151104863
TCGA.VN.A88O.01A.11R.A352.07	-0.3275	0.03788	0.125772414	0.280017411	0.130395862	-0.019927029	-0.0495643	-0.205630413	-0.180845315	0.1985444	-0.134630169	-0.186548614	0.380543208	0.191556687	-0.089314046	-0.186692199	0.252350346	-0.018859937	-0.05872035	-0.200739394	-0.174156345
TCGA.G9.6494.01A.11R.1789.07	-0.3651	-0.003	0.118989057	0.300879915	0.134828691	-0.042829942	-0.066136866	-0.238420512	-0.18698811	0.187532117	-0.108200102	-0.156120293	0.389377231	0.243430738	-0.106785905	-0.294319284	0.297457217	-0.036787495	-0.115859573	-0.168677809	-0.200807577
TCGA.HC.A6AL.01A.11R.A30B.07	-0.3487	0.09081	0.133294896	0.30421947	0.157526415	-0.018640576	-0.038193934	-0.247100035	-0.147395139	0.163745511	-0.125229255	-0.128996241	0.378987923	0.194056557	-0.082199055	-0.24243362	0.32171612	0.010718847	-0.075441179	-0.140726336	-0.214056294
TCGA.2A.AAYU.01A.11R.A41O.07	-0.4125	-0.0063	0.091977786	0.286620362	0.116077095	-0.008789657	-0.102115032	-0.190387554	-0.252821535	0.196726557	-0.148787422	-0.094623059	0.373777857	0.21258325	-0.133973712	-0.224492699	0.314469115	-0.023398829	-0.136743495	-0.251448877	-0.204852001
TCGA.YL.A8S8.01A.11R.A37L.07	-0.4559	-0.0122	0.097444209	0.321291136	0.219990931	-0.032130876	-0.063387262	-0.139384524	-0.172564397	0.176415243	-0.150390588	-0.098758039	0.33634853	0.197332755	-0.088650015	-0.26486523	0.295304732	-0.030481161	-0.083058523	-0.128472149	-0.169843447
TCGA.KK.A6E1.01A.11R.A311.07	-0.4471	0.0008	0.128657138	0.290731442	0.121889348	-0.073058232	-0.084621424	-0.250668423	-0.274135316	0.187508346	-0.110648119	-0.081887226	0.322204262	0.194553379	-0.130124292	-0.220235517	0.295284557	-0.040721092	-0.110169071	-0.243620496	-0.143436873
TCGA.TK.A8OK.01A.22R.A36G.07	-0.3968	-0.0743	0.160452978	0.365624331	0.205058994	-0.015014239	0.077065694	-0.16992907	-0.099610913	0.249368984	0.140117551	-0.104652642	0.329727092	0.32684694	0.042993059	-0.307556256	0.379750475	0.089023483	0.020734358	-0.14210637	-0.092963617
TCGA.EJ.8472.01A.11R.2403.07	-0.4866	-0.0489	0.059620194	0.289941942	0.11585606	-0.000695142	-0.041748896	-0.224907408	-0.168012501	0.200086794	-0.07990929	-0.084546922	0.346645181	0.20867841	-0.109423912	-0.291287079	0.34643566	-0.032852955	-0.134980327	-0.168874312	-0.213257069
TCGA.HC.A6AN.01A.11R.A30B.07	-0.3151	0.08614	0.181565621	0.272958013	0.153818958	-0.073590856	-0.056118017	-0.305166456	-0.234037105	0.1881103	-0.198944613	-0.123383012	0.385709673	0.168825584	-0.092708033	-0.158273852	0.307078926	0.005994264	-0.096988496	-0.164065088	-0.25915119
TCGA.G9.6498.01A.12R.A311.07	-0.117	0.21032	0.160483546	0.372588014	0.180425084	-0.021668211	0.031450233	-0.070583917	-0.225823565	0.216465402	0.038688631	-0.140801842	0.366523219	0.257314662	0.022177151	-0.238451452	0.305099873	0.035763134	0.038085137	-0.133249406	-0.143916451
TCGA.J4.AATZ.01A.11R.A41O.07	-0.4612	-0.0408	0.124516911	0.28620205	0.118853777	-0.038279981	-0.123485269	-0.122501963	-0.230494962	0.177738639	-0.132392789	-0.124384673	0.388981605	0.222524643	-0.160863811	-0.262468293	0.307149968	-0.049187165	-0.118766916	-0.242736331	-0.105489499
TCGA.CH.5765.01A.11R.1580.07	-0.4247	-0.0325	0.099375791	0.279230294	0.130127834	-0.022866238	-0.081047801	-0.185056126	-0.223890337	0.231719481	-0.129839354	-0.060986699	0.344162275	0.193091124	-0.107452172	-0.20401683	0.331043582	-0.026081746	-0.112636139	-0.22279999	-0.169399611
TCGA.EJ.A7NG.01A.31R.A33R.07	-0.2477	-0.0115	0.139629056	0.341019406	0.146157653	-0.041909238	-0.045457253	-0.291771817	-0.216137964	0.246546656	-0.043899029	-0.102761548	0.347243716	0.279393918	-0.098821293	-0.258337838	0.346639558	-0.019967724	-0.08196981	-0.163371803	-0.153479413
TCGA.EJ.A46H.01A.31R.A26U.07	-0.191	0.02373	0.195456146	0.377652871	0.217704581	-0.016940952	0.077603364	-0.064435391	-0.161501371	0.246372101	0.07306085	0.051597984	0.344343325	0.36247418	-0.018726481	-0.297182169	0.391258957	0.075972492	0.03689872	-0.149844215	-0.07142548
TCGA.EJ.5507.01A.01R.1580.07	-0.3664	0.0319	0.135100622	0.326667811	0.149734981	-0.026198055	-0.011914055	-0.329569078	-0.177990915	0.204599006	-0.150310507	-0.059056458	0.399602273	0.239722222	-0.099757968	-0.263097153	0.329260376	-0.021248414	-0.079006058	-0.159752916	-0.105956962
TCGA.KK.A8ID.01A.11R.A36G.07	-0.3669	0.00181	0.122434142	0.27368514	0.122035921	-0.02235173	-0.082176864	-0.172839515	-0.281252496	0.190900791	-0.231240426	-0.13178884	0.348760233	0.149034165	-0.154385703	-0.35866786	0.306462084	-0.033293718	-0.123386993	-0.245822189	-0.169911471
TCGA.XK.AAJR.01A.11R.A41O.07	-0.3482	-0.0264	0.192177607	0.294044102	0.12985848	-0.030069601	-0.044302221	-0.351829387	-0.215075909	0.182826215	-0.07838235	-0.092493611	0.359628262	0.217764002	-0.126943114	-0.16404756	0.344092431	-0.001942529	-0.115319177	-0.161018414	-0.139024844
TCGA.HC.7750.01A.11R.2118.07	-0.2149	-0.0171	0.135665084	0.327604856	0.154586266	-0.059974008	-0.061700611	-0.388281238	-0.197597732	0.244654811	-0.106131901	-0.098178099	0.378927184	0.255343829	-0.104108005	-0.285463711	0.353398055	-0.014533539	-0.074607534	-0.146214969	-0.136920304
TCGA.G9.6347.01A.11R.A31N.07	-0.073	0.20374	0.153637424	0.32077586	0.182139641	0.000951615	0.09953874	-0.293348848	-0.141447951	0.214752944	-0.067831524	-0.153027188	0.369542014	0.254124873	-0.01715837	-0.238186669	0.322713393	0.054792155	0.012097226	-0.111845118	-0.201249026
TCGA.EJ.5522.01A.01R.1580.07	-0.3061	0.0446	0.096430746	0.330674607	0.159342505	0.010836846	-0.031098008	-0.324937638	-0.140269023	0.233418004	-0.102583535	-0.022689587	0.344919972	0.241586957	-0.116779599	-0.209649389	0.295566509	-0.024893031	-0.077152187	-0.187027169	-0.147266572
TCGA.HC.A631.01A.11R.A29R.07	-0.4886	-0.0208	0.126267164	0.267505896	0.138650143	-0.031110886	-0.140791853	-0.384275328	-0.199642787	0.225929921	-0.245597799	-0.04865395	0.389704764	0.173676508	-0.147292545	-0.269753097	0.317040307	-0.052167092	-0.107817131	-0.21830921	-0.226750443
TCGA.CH.5763.01A.11R.1580.07	-0.2986	-0.0056	0.122073293	0.357286159	0.185866327	-0.023991851	-0.003423382	-0.050430279	-0.154671069	0.286347523	0.065588561	-0.024843903	0.336422112	0.303224706	-0.000271526	-0.219033691	0.336743511	0.048930951	-0.005969517	-0.142778838	-0.082593782
TCGA.EJ.7794.01A.11R.2118.07	-0.2293	-0.0104	0.119720713	0.330967599	0.198447831	-0.052124372	-0.007242499	-0.235361849	-0.096672112	0.217306707	0.033234539	-0.0205924	0.388624599	0.278048432	-0.067281863	-0.163856488	0.334480261	0.033176796	-0.043304512	-0.137796706	-0.151801078
TCGA.V1.A9Z8.01A.11R.A41O.07	-0.2582	0.05217	0.141365329	0.350820759	0.175595843	-0.020365097	-0.009421363	-0.411695388	-0.213757793	0.231758569	-0.046954002	-0.108227997	0.343825111	0.272761815	-0.040168296	-0.21963297	0.344593799	0.023686763	-0.025560391	-0.201598392	-0.154545007
TCGA.EJ.7788.01A.11R.2118.07	-0.4378	0.01611	0.099154574	0.31930099	0.189361999	-0.00340993	-0.025946162	-0.263666146	-0.142842498	0.209214654	-0.099839053	-0.004515795	0.33945269	0.235925349	-0.094983843	-0.268657613	0.315497986	-0.010575775	-0.071069415	-0.268456817	-0.196244795
TCGA.VN.A88P.01A.11R.A352.07	-0.3603	0.03778	0.127611404	0.319644858	0.16248329	-0.004977562	-0.053790915	-0.26362864	-0.125711672	0.189847662	-0.049016173	-0.143712312	0.316424251	0.232675494	-0.084922741	-0.256284921	0.301394022	0.009085042	-0.075262634	-0.134730061	-0.2302578
TCGA.EJ.A65J.01A.11R.A311.07	-0.4101	0.01441	0.128919954	0.316653836	0.159981342	-0.038980163	-0.0707497	-0.254724797	-0.120459629	0.173619965	-0.177145565	-0.216566991	0.412842208	0.190567845	-0.108485602	-0.248988221	0.296902941	-0.00875587	-0.102569563	-0.201860668	-0.170344422
TCGA.EJ.8474.01A.11R.2403.07	-0.4455	0.00602	0.103807063	0.332985499	0.135913284	-0.020728551	-0.054470901	-0.291125302	-0.207801319	0.211895757	-0.133560491	-0.099017858	0.370397135	0.228677913	-0.086778686	-0.255552064	0.340085666	-0.011909556	-0.093521202	-0.170677837	-0.165369044
TCGA.KK.A8IF.01A.11R.A36G.07	-0.47	0.02203	0.046966478	0.280054881	0.147801465	-0.056230131	-0.152043503	-0.348875736	-0.166862139	0.133856843	-0.240509433	-0.160768056	0.398895544	0.137086425	-0.093476395	-0.239698457	0.304414238	-0.064104581	-0.110615071	-0.166712183	-0.221321233
TCGA.G9.6339.01A.12R.A311.07	-0.3608	0.0609	0.145278029	0.319187483	0.15721418	-0.026692311	-0.081475914	-0.248148481	-0.220257053	0.214749594	-0.143152898	-0.183902605	0.368004837	0.204374829	-0.0807183	-0.255230475	0.297697143	-0.004593878	-0.081403444	-0.137508413	-0.209052598
TCGA.HC.7081.01A.11R.1965.07	-0.3697	0.00594	0.155563829	0.3357222	0.23070151	-0.048196047	0.095716897	-0.374739173	-0.09856874	0.243073006	-0.081758117	0.011160803	0.391547308	0.262158539	0.002807848	-0.140264452	0.351473553	0.046432763	-0.03898718	-0.188126208	-0.125752618
TCGA.XJ.A9DX.01A.11R.A37L.07	-0.4242	-0.0118	0.163777128	0.24373754	0.122952323	-0.042020349	-0.067179344	-0.489481226	-0.207506537	0.151634496	-0.217513729	-0.162962712	0.371295679	0.196123438	-0.104316217	-0.245637197	0.343823133	-0.049101861	-0.108437534	-0.212550527	-0.279951616
TCGA.YL.A8SO.01B.11R.A37L.07	-0.326	0.01957	0.150871649	0.291813787	0.146960034	-0.076028227	-0.036609028	-0.30752195	-0.215632971	0.168165737	-0.122644754	-0.151523337	0.305492521	0.21249161	-0.091444948	-0.186385817	0.305015462	-0.022226345	-0.088227656	-0.211221083	-0.176514469
TCGA.V1.A8X3.01A.11R.A37L.07	-0.306	0.02318	0.125755811	0.286058675	0.150764539	-0.031208964	-0.071606971	-0.210767446	-0.211866736	0.183998919	-0.067386802	-0.12812023	0.354858736	0.21771492	-0.115765139	-0.235773088	0.316637439	-0.005470285	-0.086128503	-0.18798076	-0.169159167
TCGA.J4.A83N.01A.11R.A352.07	-0.3603	0.03343	0.100125196	0.274529616	0.124175244	-0.040787711	-0.0668326	-0.137744559	-0.285657785	0.242072028	-0.120959356	-0.108818312	0.356055659	0.194164372	-0.122097509	-0.17354695	0.29900089	-0.006912036	-0.106733135	-0.187107212	-0.198809961
TCGA.XA.A8JR.01A.11R.A36G.07	-0.2653	0.00881	0.127997652	0.312778712	0.167118571	-0.049630435	-0.032922171	-0.213740221	-0.178948506	0.226187308	0.005717599	-0.182123129	0.320247764	0.222208913	-0.097194798	-0.251182006	0.316283148	-0.007121937	-0.054534285	-0.18265153	-0.197392941
TCGA.EJ.7315.01A.31R.2118.07	-0.1373	0.17414	0.11425055	0.370282864	0.16997743	0.00971335	0.046495688	-0.303155956	-0.048066877	0.18003023	-0.165702615	-0.01295194	0.38741112	0.237411897	-0.040172743	-0.09066461	0.332531752	0.075421626	0.014292761	-0.201456658	-0.122329945
TCGA.M7.A724.01A.12R.A32O.07	-0.4678	0.01355	0.125364328	0.229455678	0.108380598	-0.039543121	-0.173542593	-0.320569559	-0.324839299	0.157710716	-0.188223816	-0.096120004	0.349907888	0.059000709	-0.142659169	-0.244768569	0.280887837	-0.017838747	-0.193550704	-0.162333183	-0.17000168
TCGA.EJ.A65M.01A.11R.A29R.07	-0.3891	0.10094	0.124112801	0.316242356	0.155818367	-0.048165429	-0.032336392	-0.322429885	-0.14797839	0.189647738	-0.110895017	-0.073851955	0.367309125	0.230296279	-0.095307749	-0.166324351	0.319764801	-0.022446016	-0.096062587	-0.183381469	-0.167134969
TCGA.G9.7519.01A.11R.2263.07	-0.3198	0.02442	0.129113844	0.313547416	0.172536463	-0.054561763	-0.09688208	-0.223024839	-0.166077941	0.220785456	-0.028988107	-0.170553324	0.36718522	0.203168283	-0.102269573	-0.219935382	0.284295094	-0.036805743	-0.084009838	-0.162838434	-0.194778227
TCGA.EJ.A7NH.01A.12R.A33R.07	-0.3331	0.00282	0.180866458	0.305309408	0.127340271	-0.081823296	-0.056215822	-0.388075441	-0.242039611	0.227831113	-0.14151914	-0.082727664	0.343343709	0.238795341	-0.119565152	-0.146924179	0.323026336	-0.042133474	-0.109443021	-0.166982268	-0.244620786
TCGA.HC.7740.01A.11R.2118.07	-0.2464	0.00468	0.16810913	0.353668339	0.190898865	-0.066327979	0.026652113	-0.121098874	-0.111229851	0.224357695	0.056670619	-0.059349332	0.363072963	0.312753974	-0.047593769	-0.223085287	0.342414111	0.033953662	-0.035903462	-0.121345434	-0.107787879
TCGA.FC.A4JI.01A.11R.A250.07	-0.4577	0.08521	0.098548049	0.27801194	0.118251849	-0.010118996	-0.136398863	-0.409476614	-0.241565501	0.238550486	-0.245698761	0.022709016	0.328384512	0.12607795	-0.114409724	-0.33640439	0.201013031	-0.109444235	-0.102703522	-0.197867792	-0.130411967
TCGA.J4.A83J.01A.11R.A36G.07	-0.4598	0.09391	0.142686905	0.299084459	0.129376013	-0.067326144	0.005275168	-0.087975165	-0.192218974	0.185142795	0.138711856	-0.120800257	0.392139442	0.238254101	-0.116757971	-0.276203955	0.295759779	0.031054341	-0.049388734	-0.196507958	-0.083605403
TCGA.HC.7737.01A.11R.2118.07	-0.2138	0.09911	0.158237641	0.363970326	0.180312529	-0.046772807	0.030894167	-0.048918239	-0.122955053	0.212306628	0.033859296	-0.061547658	0.389826964	0.298963892	-0.006657499	-0.237920207	0.363277317	0.074572983	0.001265679	-0.194876476	-0.071922516
TCGA.ZG.A9LY.01A.11R.A41O.07	-0.4118	0.00741	0.190995466	0.344475001	0.166584104	-0.029648645	-0.011569535	-0.140792419	-0.146056049	0.242916755	0.038776628	-0.068492181	0.339269226	0.295558744	-0.06454043	-0.233006014	0.357943286	0.001709677	-0.055536691	-0.203183259	-0.176527105
TCGA.EJ.A7NF.01A.11R.A33R.07	-0.3604	0.02448	0.109720135	0.254362627	0.127962082	-0.047049092	-0.081180426	-0.374693779	-0.287480258	0.218972945	-0.309628809	-0.138202729	0.356751038	0.153393282	-0.131685237	-0.183740652	0.304689707	-0.065445669	-0.145524266	-0.182319917	-0.240825673
TCGA.HC.A48F.01A.11R.A250.07	-0.4327	-0.0657	0.125131236	0.277814523	0.097451376	-0.021447664	-0.151564732	-0.354660345	-0.242141035	0.195411266	-0.067179995	-0.154894537	0.350944078	0.151785667	-0.118161102	-0.174115179	0.326572544	-0.087738777	-0.166690674	-0.202440581	-0.19840464
TCGA.YL.A9WJ.01A.11R.A37L.07	-0.3611	0.04227	0.127248415	0.357839137	0.165602695	-0.014899216	0.020507675	-0.188338832	-0.15383738	0.219437883	0.000727339	-0.059738025	0.374718384	0.263734304	-0.05250154	-0.229409413	0.357290342	0.056477376	-0.028607403	-0.145795149	-0.156855543
TCGA.G9.6379.01A.11R.A31N.07	-0.1161	0.22073	0.2099317	0.324242365	0.152121558	-0.058795519	0.09173277	-0.25477352	-0.17066221	0.190527736	-0.072720683	-0.062847623	0.383068242	0.235044713	-0.043438861	-0.182994201	0.33994151	0.060802293	-0.025312318	-0.088148676	-0.186699195
TCGA.KK.A8II.01A.11R.A36G.07	-0.4992	-0.0251	0.078735089	0.242424379	0.12485304	-0.049590778	-0.083687427	-0.179227777	-0.270243169	0.215088738	-0.15142391	-0.122316783	0.334730961	0.1224295	-0.140387166	-0.178423317	0.280002372	-0.055497621	-0.150306083	-0.19267779	-0.223207118
TCGA.G9.6353.01A.11R.1965.07	-0.2447	-0.0322	0.156551875	0.312704179	0.124088861	-0.064633495	-0.073496852	-0.343478909	-0.265419725	0.238261786	-0.082176636	-0.103774234	0.379823376	0.248065944	-0.108599115	-0.194264507	0.330615083	-0.007061333	-0.08279125	-0.163074566	-0.178372934
TCGA.HI.7170.01A.11R.2118.07	-0.2237	0.06517	0.193568352	0.380620445	0.210845394	-0.036463645	0.015419401	-0.155516056	-0.145564691	0.232146312	0.080411341	-0.057641829	0.342122859	0.325688902	0.024628603	-0.2364904	0.38221089	0.07848863	0.014599198	-0.123991635	-0.146753454
TCGA.HC.7742.01A.11R.2118.07	-0.3674	-0.0022	0.115397319	0.319805875	0.144451334	-0.013346794	-0.125705577	-0.307398132	-0.203629238	0.19467354	-0.133539571	-0.118631472	0.353259249	0.220280039	-0.078168178	-0.239970063	0.310274331	0.008795161	-0.088762481	-0.24357972	-0.164493415
TCGA.FC.A8O0.01A.41R.A37L.07	-0.3513	-0.0534	0.149867693	0.30256374	0.119954512	-0.013807765	-0.100654415	-0.363947125	-0.276863641	0.237761232	-0.172490027	-0.184229438	0.36110166	0.253389361	-0.095031087	-0.477116261	0.382291598	-0.034740946	-0.110929316	-0.203418377	-0.181596808
TCGA.EJ.5508.01A.02R.1580.07	-0.3174	0.06676	0.096748969	0.337523786	0.17474838	-0.010373322	0.047295107	-0.221918764	-0.105341252	0.226257299	-0.047483862	-0.026605629	0.339905765	0.285996985	-0.062045469	-0.167782199	0.325089661	0.008421207	-0.064797315	-0.20676227	-0.114019051
TCGA.HC.7749.01A.11R.2118.07	-0.4858	0.01663	0.10494409	0.322195611	0.162295281	-0.033864451	-0.058836742	-0.271905843	-0.164447042	0.20075761	-0.021354645	-0.096622481	0.389091535	0.197429584	-0.09871776	-0.345236644	0.333405385	-0.022285726	-0.115219478	-0.196762413	-0.187771677
TCGA.V1.A9OT.01A.11R.A41O.07	-0.321	0.07598	0.162738146	0.298623195	0.097017572	-0.05984754	-0.045345442	-0.361543898	-0.298232785	0.177629793	-0.148540363	-0.066557877	0.35999638	0.232169113	-0.122514776	-0.2051617	0.316803582	-0.039786951	-0.113651933	-0.145961712	-0.26664635
TCGA.CH.5741.01A.11R.1580.07	-0.51	-0.0102	0.123088158	0.277393228	0.139414824	-0.023922459	-0.059959241	-0.20082116	-0.30033704	0.251792658	-0.099157434	-0.104813061	0.403045523	0.152142509	-0.143848016	-0.164688878	0.281182271	-0.038280076	-0.111995665	-0.23899227	-0.139865243
TCGA.KK.A8IC.01A.11R.A36G.07	-0.3096	-0.011	0.158979292	0.358552173	0.140488319	-0.058963886	0.029775901	-0.179105593	-0.158969228	0.241939366	0.01166081	-0.042430128	0.363283895	0.302286736	-0.06406652	-0.1841995	0.341228782	0.024189184	-0.033319295	-0.198100489	-0.151552411
TCGA.YL.A9WH.01A.11R.A37L.07	-0.4977	-0.0155	0.121882218	0.327515526	0.161234722	-0.062535871	-0.136645163	-0.268299226	-0.235926818	0.182762361	-0.199350535	-0.077194192	0.35428805	0.145478041	-0.085713664	-0.223121619	0.295995807	-0.053468278	-0.110104172	-0.235170208	-0.253985093
TCGA.ZG.A9L5.01A.12R.A41O.07	-0.4604	-0.0039	0.079137821	0.246874067	0.145713197	-0.044280558	-0.105223833	-0.266005263	-0.240221581	0.201388834	-0.058513683	-0.15293128	0.307801174	0.19609709	-0.109255299	-0.342440228	0.272774009	-0.043240336	-0.162203018	-0.278669525	-0.205050284
TCGA.CH.5768.01A.11R.1580.07	-0.328	0.01054	0.062824141	0.283144851	0.165211215	-0.03703859	-0.073794362	-0.246633339	-0.219200113	0.221919278	-0.178215924	-0.016197692	0.341742864	0.170506847	-0.116883779	-0.224340538	0.297068553	-0.057190338	-0.099559489	-0.18906476	-0.150523977
TCGA.KC.A4BL.01A.31R.A250.07	-0.291	0.03382	0.185280942	0.345792294	0.171298212	-0.054919411	0.007993234	-0.153554007	-0.162348296	0.239351208	0.026075555	-0.061183909	0.343901251	0.294510117	-0.013323506	-0.291195437	0.355583555	0.035825063	-0.029618537	-0.154010461	-0.123520478
TCGA.J4.A67K.01A.21R.A30B.07	-0.2857	0.16388	0.127841468	0.271618421	0.138073895	-0.045784484	0.030553901	-0.178982064	-0.19909668	0.20446242	-0.123188596	-0.072516701	0.3539135	0.180883019	-0.095979727	-0.197097173	0.304378641	-0.023469258	-0.048839252	-0.185161649	-0.195178545
TCGA.G9.7525.01A.31R.2263.07	-0.4236	0.01614	0.110963048	0.303217562	0.116902202	-0.058034777	-0.02627181	-0.341541819	-0.202200631	0.250439798	-0.158591198	-0.093042309	0.405733002	0.209795799	-0.069102942	-0.148980907	0.278735414	0.013629401	-0.107555047	-0.162878797	-0.178796196
TCGA.G9.6371.01A.11R.1789.07	-0.3735	0.03189	0.074800246	0.28921075	0.13317687	-0.056207807	-0.11124385	-0.228241262	-0.227349829	0.20441057	-0.172633066	-0.112610209	0.388177393	0.18171064	-0.105561078	-0.171124038	0.274262944	-0.049686339	-0.116739617	-0.188417208	-0.200605604
TCGA.HC.A76W.01A.11R.A33R.07	-0.3705	0.01001	0.145503243	0.359795866	0.187844867	-0.03733279	-0.007168571	-0.203251123	-0.18805516	0.215558092	-0.019044062	-0.092303973	0.30286137	0.279639049	0.005921338	-0.24751942	0.312214656	0.020547652	-0.056553544	-0.204981975	-0.130543996
TCGA.EJ.A65G.01A.21R.A29R.07	-0.404	-0.0003	0.129106389	0.29366039	0.112725449	-0.048398756	-0.135544176	-0.278298365	-0.274077627	0.228469277	-0.177752587	-0.188744602	0.376049822	0.159741205	-0.14882515	-0.26121101	0.290367377	-0.059179648	-0.136185574	-0.171604248	-0.211751907
TCGA.ZG.A9LS.01A.12R.A41O.07	-0.1328	0.20516	0.123371149	0.377202842	0.157490443	-0.021218294	0.089734287	-0.249572538	-0.077739369	0.241643078	-0.088053555	-0.023919323	0.313613854	0.296972572	-0.005154651	-0.253913167	0.387284342	0.041770817	0.020215758	-0.0892063	-0.15507797
TCGA.XJ.A9DK.01A.11R.A37L.07	-0.3553	0.01641	0.170178948	0.33740308	0.165920917	-0.01965645	-0.029901356	-0.298365506	-0.219904231	0.232767958	-0.137931711	-0.085162674	0.359160009	0.25354438	-0.08614893	-0.276749008	0.344579298	-0.026578012	-0.098923452	-0.187780786	-0.213148416
TCGA.G9.6329.01A.13R.1965.07	-0.2923	0.09457	0.158406535	0.35056498	0.166881886	-0.005290281	-0.004271215	-0.32547763	-0.196339371	0.203031021	-0.115226896	-0.077337858	0.356517085	0.27021591	-0.079283195	-0.120850632	0.33614904	0.004323117	-0.050286956	-0.157701369	-0.151753222
TCGA.J9.A8CN.01A.11R.A352.07	-0.2845	0.01152	0.082295472	0.280963156	0.146824599	-0.047100101	-0.088274848	-0.097103631	-0.259444599	0.206130203	-0.028890593	-0.167821465	0.357618497	0.15115323	-0.069258381	-0.214951598	0.31375785	0.014225862	-0.065664553	-0.158543284	-0.115581332
TCGA.V1.A9ZK.01A.11R.A41O.07	-0.3623	0.00168	0.135568409	0.317964993	0.139674295	-0.062131063	-0.038217776	-0.429254777	-0.171403985	0.189309943	-0.11646137	-0.105578769	0.383296114	0.249953826	-0.110037619	-0.236552827	0.293519247	-0.034824346	-0.088639577	-0.19225742	-0.203963861
TCGA.EJ.5542.01A.01R.1580.07	-0.3603	0.00289	0.083790474	0.314280337	0.160166816	0.031157918	0.001739699	-0.244846251	-0.130039423	0.222734734	-0.117312422	0.001110126	0.386588862	0.235824363	-0.105423176	-0.188091469	0.30642598	-0.018790934	-0.073884666	-0.2105641	-0.139584632
TCGA.V1.A8WS.01A.11R.A37L.07	-0.3945	-0.0121	0.086229379	0.26916546	0.112998646	-0.033231543	-0.101254138	-0.284603322	-0.311679333	0.217838967	-0.252603885	-0.074682589	0.312797313	0.159059936	-0.14989174	-0.191217251	0.301391626	-0.029284295	-0.126657892	-0.250078152	-0.28124247
TCGA.ZG.A8OX.01A.11R.A37L.07	-0.2681	0.03426	0.074926393	0.311581933	0.130549929	0.0053997	-0.111607802	-0.174600945	-0.229605343	0.206571674	-0.049956891	-0.193864315	0.33074836	0.207230072	-0.097871573	-0.265306355	0.282616012	-0.010598226	-0.062852225	-0.156037835	-0.14253947
TCGA.YL.A8SC.01A.11R.A37L.07	-0.4062	0.08933	0.092288185	0.294272771	0.152213195	-0.038531125	0.002569718	-0.23726131	-0.130158708	0.218401903	-0.135140505	-0.092687802	0.344128341	0.218845249	-0.09618127	-0.19923723	0.326158195	-0.015192538	-0.103057238	-0.231102756	-0.209170004
TCGA.YL.A8HM.01A.11R.A36G.07	-0.3827	0.18189	0.013040626	0.297082452	0.210242165	-0.021945768	0.015798119	-0.434113988	-0.082934652	0.173641597	-0.209896108	-0.111768749	0.3643817	0.152590515	-0.107404557	-0.131725472	0.299896947	-0.064406206	-0.110763103	-0.196116311	-0.182866803
TCGA.M7.A721.01A.12R.A32O.07	-0.3142	0.01637	0.18532763	0.328495098	0.128137965	-0.058928042	-0.046722721	-0.36661241	-0.19997472	0.197045267	-0.096805363	-0.101885612	0.394017896	0.218262569	-0.107198155	-0.247721537	0.322850351	-0.008683556	-0.069646352	-0.20564571	-0.201653662
TCGA.V1.A8WL.01A.11R.A37L.07	-0.1731	0.14454	0.118868798	0.35797756	0.150875592	-0.040776301	-0.016424274	-0.122451099	-0.123306156	0.187452463	0.01006429	-0.146277102	0.333657212	0.235927487	-0.082522606	-0.409590223	0.325794788	-0.009810225	-0.014794105	-0.213922611	-0.176819597
TCGA.KK.A7AV.01A.11R.A32O.07	-0.0935	0.19959	0.157999906	0.378026103	0.154865866	-0.042411265	0.075396486	-0.313335199	-0.062319268	0.217441628	-0.033540734	-0.099181466	0.369143309	0.2696759	-0.04919258	-0.228713608	0.342227486	0.045880919	-0.016812766	-0.166999356	-0.184494809
TCGA.G9.6370.01A.11R.1789.07	-0.2531	0.00184	0.163733126	0.351931977	0.174544287	0.003600278	0.027671289	-0.162760758	-0.195381789	0.264567181	0.016503212	-0.02271261	0.354982784	0.31893312	-0.056840682	-0.237697171	0.310518476	0.035442362	-0.050364765	-0.143069653	-0.100317188
TCGA.EJ.5514.01A.01R.1580.07	-0.4039	0.01061	0.079085173	0.333859979	0.200543566	-0.001037108	0.009871005	-0.180237477	-0.133896275	0.196483946	-0.013672049	0.005219889	0.372368652	0.244577355	-0.028058381	-0.180601117	0.340748676	0.052202075	-0.053196843	-0.167717782	-0.071198477
TCGA.VP.A87K.01A.11R.A352.07	-0.4119	0.02184	0.111881826	0.266578018	0.1171918	-0.04219902	-0.004026681	-0.335369683	-0.243368025	0.159557853	-0.312334124	-0.096282587	0.34983758	0.142352426	-0.142211027	-0.201912014	0.288503913	-0.067574322	-0.159980733	-0.21950064	-0.253292743
TCGA.ZG.A9MC.01A.31R.A41O.07	0.01816	0.25176	0.165257799	0.412098631	0.200956645	-0.008315231	0.164633102	-0.136629913	0.036662963	0.217854253	-0.001170693	0.030462232	0.342239649	0.333983139	0.047234325	-0.259460022	0.372010434	0.073566096	0.079810946	-0.098653493	-0.083200313
TCGA.G9.7522.01A.11R.2263.07	-0.2671	0.03525	0.148758157	0.332147722	0.144635113	-0.034735052	-0.012245941	-0.180118513	-0.19535878	0.24194557	-0.048964111	-0.100799124	0.379124594	0.219311107	-0.077730122	-0.125804598	0.326389367	-0.002084589	-0.048067391	-0.159472354	-0.130197529
TCGA.CH.5752.01A.11R.1580.07	-0.4064	-0.0325	0.070154456	0.253330462	0.135795847	0.010078538	-0.054148877	-0.205105385	-0.257128746	0.196415484	-0.199356484	-0.080301785	0.341727802	0.134927383	-0.102528369	-0.164048139	0.310582958	-0.047512486	-0.13541317	-0.289877	-0.19265052
TCGA.KK.A7AP.01A.12R.A33R.07	-0.4562	0.01379	0.149406084	0.260977034	0.11202324	-0.034000048	-0.11102851	-0.425327939	-0.239882389	0.208973123	-0.249559074	-0.216068735	0.368286854	0.163062816	-0.128598643	-0.397285382	0.304574374	-0.107259433	-0.145859996	-0.241055031	-0.26431639
TCGA.KK.A6E4.01A.11R.A30B.07	-0.2942	0.09276	0.080185432	0.228540504	0.108970711	-0.036336577	-0.059454878	-0.220038676	-0.275112067	0.175337431	-0.187915822	-0.107881279	0.379378466	0.115657144	-0.112977777	-0.185325702	0.287867492	-0.015624509	-0.101420401	-0.169898075	-0.21023008
TCGA.HC.A632.01A.11R.A29R.07	-0.0541	0.18622	0.083798954	0.349211931	0.215288336	-0.001743557	0.137074402	-0.32951102	0.009132444	0.22763671	-0.121489782	-0.000358359	0.295281848	0.262343709	-0.037557633	-0.249859165	0.343518045	0.019654122	-0.006248616	-0.184883094	-0.163452138
TCGA.EJ.7321.01A.31R.2263.07	-0.2814	0.09564	0.062693573	0.293010915	0.142722035	-0.028901365	0.009577169	-0.168166207	-0.161076688	0.234355342	-0.148914742	-0.009339699	0.372909622	0.209209697	-0.156670064	-0.232822858	0.302916878	-0.029810898	-0.080426406	-0.207402878	-0.157702507
TCGA.EJ.A8FU.01A.11R.A36G.07	-0.2347	0.02345	0.162604141	0.333463288	0.148134188	-0.040298692	0.006222316	-0.209063517	-0.161923171	0.247245891	-0.001682914	-0.082873868	0.331752246	0.300733194	-0.081879931	-0.247687588	0.342485399	0.017473279	-0.052303702	-0.127027909	-0.1496941
TCGA.EJ.5518.01A.01R.1580.07	-0.3881	0.00893	0.125832724	0.330026887	0.19076091	-0.050125511	0.012038723	-0.228649925	-0.152513664	0.184998806	-0.018449915	-0.09688334	0.364532769	0.269951699	-0.034392128	-0.205703275	0.288087474	0.046132342	-0.048658232	-0.169909416	-0.169380104
TCGA.EJ.5524.01A.01R.1580.07	-0.3827	0.06412	0.083366645	0.305635768	0.161378251	0.008306476	0.03962265	-0.199122513	-0.055420416	0.251925968	-0.11320785	-0.014755936	0.334167775	0.245190089	-0.089924324	-0.229214229	0.326776156	0.001124458	-0.050605919	-0.199036255	-0.128439519
TCGA.J4.8198.01A.11R.2263.07	-0.4136	-0.0164	0.099624343	0.287364549	0.137993366	-0.015091484	-0.035564978	-0.293857786	-0.235550952	0.207987648	-0.111439077	-0.086108131	0.361311661	0.237590034	-0.109468493	-0.169946914	0.302778585	-0.042846565	-0.103222331	-0.227667134	-0.141268281
TCGA.KK.A8IM.01A.11R.A36G.07	-0.3507	0.04701	0.137933189	0.328151107	0.148480133	-0.033802869	-0.005030844	-0.289547962	-0.175197472	0.225229666	-0.160911915	-0.058788258	0.346873483	0.22525305	-0.108581715	-0.222972959	0.337894157	-0.000753655	-0.081704525	-0.224309476	-0.169329767
TCGA.CH.5748.01A.11R.1580.07	-0.3308	0.01433	0.112872987	0.294892038	0.132884681	-0.048332758	-0.095183388	-0.375483084	-0.160713344	0.223729673	-0.243569087	-0.083420718	0.373921486	0.184779732	-0.148337568	-0.267716035	0.29254917	-0.058953673	-0.108013631	-0.207548286	-0.17347023
TCGA.VP.A87J.01A.11R.A352.07	-0.5006	-0.0155	0.125527281	0.277937594	0.140206121	-0.053488842	-0.099887006	-0.071703211	-0.212969236	0.178508255	-0.044215471	-0.15671467	0.392704973	0.181038739	-0.110047938	-0.239044954	0.301518122	-0.029805796	-0.105146208	-0.134884739	-0.1527004
TCGA.HC.7231.01A.11R.2118.07	-0.3416	0.07292	0.08496449	0.337160332	0.146684341	-0.003146827	-0.003760872	-0.331495957	-0.13803942	0.230572575	-0.161613174	-0.068759221	0.390236984	0.224729067	-0.103620298	-0.146391538	0.310609136	-0.017238937	-0.065080224	-0.229309555	-0.207641825
TCGA.2A.A8W1.01A.11R.A37L.07	-0.5285	0.01502	0.022259501	0.238940151	0.113669856	-0.012809956	-0.12216636	-0.262406077	-0.31260255	0.214110618	-0.212626747	-0.113357043	0.342757944	0.063115662	-0.158942245	-0.235481871	0.262145809	-0.04835597	-0.149750685	-0.200241315	-0.149511312
TCGA.EJ.7331.01A.11R.2118.07	-0.2838	-0.0037	0.103445741	0.337453887	0.173496748	-0.035058467	-0.012100243	-0.20366372	-0.076450639	0.235111147	0.007295126	-0.100445658	0.367988385	0.278375058	-0.063206901	-0.316588319	0.34748792	0.019814528	-0.055197095	-0.227758725	-0.142344732

TCGA.EJ.7783.01A.11R.2118.07	-0.2821	-0.0143	0.152847565	0.330913186	0.142931656	-0.043808571	-0.004664634	-0.230296541	-0.148552516	0.218703884	-0.038087048	-0.052968759	0.314051602	0.298107721	-0.106878423	-0.182959299	0.375257426	-0.022506718	-0.098152065	-0.205253854	-0.117762954
TCGA.J4.A67S.01A.11R.A30B.07	-0.3109	0.12389	0.174946168	0.281175192	0.119020146	-0.063129639	0.02267315	-0.183580171	-0.253441216	0.16887027	-0.058443349	-0.131735442	0.379095916	0.164294013	-0.120125896	-0.223982078	0.306970183	-0.012426518	-0.081389522	-0.114745239	-0.204710679
TCGA.V1.A9OL.01A.11R.A41O.07	-0.377	0.07022	0.129975119	0.296432365	0.162395056	-0.029469158	-0.069629667	-0.382487061	-0.183495297	0.175371516	-0.194556698	-0.101950789	0.312683087	0.202909861	-0.092834905	-0.346434284	0.291191918	-0.029445962	-0.104840861	-0.243441885	-0.216623778
TCGA.HC.7745.01A.11R.2118.07	-0.2442	0.10188	0.155584743	0.374088327	0.180670369	-0.055906762	0.046081458	-0.180823375	-0.111814569	0.25377792	-0.025234481	0.000426766	0.352162047	0.28242401	-0.052715332	-0.233365138	0.358402106	0.017559777	-0.038331026	-0.117371894	-0.14077183
TCGA.MG.AAMC.01A.11R.A41O.07	-0.3711	0.04101	0.088532745	0.271208041	0.122794228	0.006165956	-0.097944735	-0.208332947	-0.276648734	0.198050386	-0.156350464	-0.226706787	0.379844443	0.130301853	-0.105947711	-0.216090689	0.286758865	-0.032962126	-0.113802677	-0.190340295	-0.214806485
TCGA.G9.6348.01A.11R.1789.07	-0.323	0.07262	0.17922058	0.342186834	0.169360981	0.005338213	0.007503146	-0.08900202	-0.150267439	0.223085497	0.012304674	-0.062131363	0.379146271	0.242465002	-0.017413862	-0.201965058	0.34321535	0.034776986	-0.021202364	-0.178409124	-0.145042024
TCGA.G9.6338.01A.12R.1965.07	-0.3833	-0.0143	0.163543625	0.325343386	0.14984537	0.006681906	-0.017028426	-0.24187661	-0.273394319	0.204636374	-0.120208554	-0.195691748	0.352000974	0.237792378	-0.020549485	-0.208771254	0.32519994	-0.016786112	-0.035015679	-0.093548764	-0.151787876
TCGA.EJ.7791.01A.11R.2118.07	-0.2455	0.0035	0.154555279	0.341886672	0.182701644	-0.061434926	-0.005102228	-0.212664641	-0.176685428	0.23212761	-0.001078316	-0.043907862	0.349576478	0.285052352	-0.062040021	-0.271472459	0.333029771	0.024976361	-0.041945908	-0.189976136	-0.122107238
TCGA.M7.A71Y.01A.22R.A32O.07	-0.4362	0.0209	0.154790075	0.33373587	0.152490757	-0.050924419	-0.031808486	-0.315256997	-0.201261053	0.228912839	-0.149282101	-0.085354674	0.370642816	0.247446558	-0.099872084	-0.255881248	0.321684702	-0.026489593	-0.106462194	-0.157671293	-0.234685131
TCGA.XO.A8TB.01A.11R.A36G.07	-0.4309	-0.0283	0.092430679	0.327614343	0.155706216	-0.025177148	-0.016080066	-0.10444325	-0.159902242	0.23193124	-0.115828787	-0.046222095	0.364622132	0.217796943	-0.052790668	-0.268297928	0.338135844	-0.013332073	-0.032008096	-0.226316855	-0.181479245
TCGA.M7.A725.01A.12R.A32O.07	-0.5174	-0.0187	0.099747679	0.284208506	0.141404493	-0.01991184	-0.109608962	-0.213060725	-0.201496405	0.19769665	-0.15900636	-0.157680661	0.340150395	0.168013098	-0.119665252	-0.177432806	0.275986757	-0.029826901	-0.121881267	-0.187698973	-0.155249268
TCGA.ZG.A9LU.01A.11R.A41O.07	-0.3126	0.12733	0.226753806	0.346500329	0.16368225	-0.059316014	0.016625547	-0.192143926	-0.164228967	0.212549195	-0.058205616	-0.041776029	0.351004437	0.263051214	-0.081178241	-0.293798185	0.34540293	0.022370245	-0.065521772	-0.099712998	-0.174320038
TCGA.KK.A6E7.01A.11R.A31N.07	-0.3771	0.01757	0.185403312	0.284358087	0.13160075	-0.014764345	-0.108360374	-0.326413257	-0.261873003	0.168455155	-0.166506766	-0.162817152	0.333341414	0.14581517	-0.067654781	-0.29015804	0.291846304	-0.02136668	-0.087407241	-0.127260275	-0.101421388
TCGA.KK.A8IJ.01A.11R.A352.07	-0.3955	-0.0169	0.130476276	0.307936279	0.136034655	-0.048722762	-0.110186727	-0.137831607	-0.237158403	0.192417955	-0.00372123	-0.155181179	0.372329063	0.181938901	-0.07001426	-0.201352599	0.318760596	0.0498097	-0.075258551	-0.171160812	-0.133577973
TCGA.V1.A9OF.01A.11R.A41O.07	-0.3714	0.00872	0.093908469	0.279801454	0.130893038	-0.070287739	-0.092332357	-0.397058976	-0.221990206	0.197517023	-0.146433192	-0.119583028	0.373276645	0.15644718	-0.140622129	-0.180090136	0.273351627	-0.056672559	-0.144984102	-0.187871327	-0.199522505
TCGA.KK.A6E3.01A.21R.A30B.07	-0.1686	0.1493	0.139518774	0.33358942	0.174636123	-0.042350501	0.020298634	-0.250526617	-0.12732635	0.206751055	-0.02284532	-0.172564695	0.366088216	0.24214193	-0.043681682	-0.238016102	0.320305174	0.021023652	-0.021490175	-0.09283105	-0.170844364
TCGA.KK.A7AW.01A.11R.A32O.07	-0.1409	0.20281	0.156805642	0.241631129	0.146066311	-0.057883191	-0.003029669	-0.273628684	-0.189449552	0.158281661	-0.124051554	-0.117003821	0.382848023	0.21028967	-0.096090347	-0.13309584	0.321748875	0.006820978	-0.05784646	-0.149678011	-0.252649912
TCGA.YL.A8SB.01A.31R.A37L.07	-0.3193	0.00472	0.120978848	0.345708423	0.178153219	-0.036225497	-0.035499328	-0.364985194	-0.157458228	0.225078936	-0.048566575	-0.093595582	0.361694443	0.271735147	-0.061360626	-0.34365886	0.366711462	-0.00574529	-0.069537457	-0.189891883	-0.178243974
TCGA.EJ.A7NN.01A.11R.A33R.07	-0.2365	0.02933	0.159841851	0.316644881	0.148246439	-0.027067278	-0.028930147	-0.160978078	-0.121188754	0.151450612	-0.002280525	-0.147744483	0.380411141	0.270191866	-0.101001204	-0.1547134	0.347367307	-0.009338856	-0.098561713	-0.207408229	-0.163284395
TCGA.HC.7212.01A.11R.2118.07	-0.4356	-0.0313	0.120072891	0.274591688	0.139929805	-0.031188237	-0.031002252	-0.227574449	-0.19936327	0.200040412	-0.109408743	-0.058050382	0.386526849	0.177590405	-0.12593114	-0.171982973	0.299420921	-0.011592399	-0.134183744	-0.195684347	-0.163550636
TCGA.V1.A9O5.01A.11R.A41O.07	-0.3554	0.15951	0.104448533	0.350145422	0.21112435	-0.045278554	0.02636848	-0.327146939	-0.098710433	0.199186844	-0.077817353	-0.083073632	0.366487253	0.240680245	-0.061118559	-0.329321871	0.287201969	0.030972654	-0.05165575	-0.21549484	-0.096149266
TCGA.HC.A8D0.01A.11R.A36G.07	-0.2985	0.05613	0.13847124	0.347663366	0.153468248	-0.029418541	0.026821899	-0.359088073	-0.182872465	0.2472852	-0.087564428	-0.004064303	0.35009956	0.22435189	-0.078099565	-0.228242746	0.351575584	0.010868174	-0.042348734	-0.206277008	-0.163969256
TCGA.2A.A8VT.01A.11R.A37L.07	-0.4312	-0.0058	0.127885899	0.300708498	0.103157034	-0.035883603	-0.059104224	-0.256607381	-0.179476956	0.215442212	-0.272814919	-0.105708221	0.344997058	0.199049411	-0.108158781	-0.235545542	0.303874226	-0.055212872	-0.095281694	-0.229530716	-0.273639136
TCGA.ZG.A9LB.01A.11R.A41O.07	-0.3634	0.14124	0.122709657	0.324388583	0.232327023	-0.015707627	0.027157061	-0.079693608	-0.192116114	0.208045527	0.08048682	-0.05999339	0.322629518	0.219570698	-0.008718252	-0.173244857	0.305320516	0.03781051	-0.015006568	-0.110203765	-0.126678446
TCGA.HC.7079.01A.11R.1965.07	0.31279	0.34775	0.202260144	0.469076319	0.238978778	0.04642337	0.332350104	-0.029894817	0.253268246	0.268928629	0.100926047	0.048136673	0.393452484	0.383874278	0.078561371	-0.147391006	0.445888712	0.19586774	0.187570577	-0.085679747	0.010947736
TCGA.HC.A6AP.01A.11R.A30B.07	-0.1944	0.10783	0.179917188	0.307320807	0.145452406	-0.066482123	0.005977698	-0.324411875	-0.115043971	0.196489977	-0.02429717	-0.118402868	0.36557224	0.211019068	-0.030765054	-0.188470509	0.34991761	0.042270305	-0.045225338	-0.131548953	-0.19315034
TCGA.KC.A4BR.01A.32R.A32Y.07	-0.3111	-0.0493	0.206610589	0.333642428	0.154252724	-0.046616756	0.045052582	-0.120195201	-0.280702484	0.220644512	0.09084402	-0.070832569	0.345505993	0.262830124	-0.01215058	-0.120995416	0.374578782	0.064025383	0.026697318	-0.152724948	-0.111495726
TCGA.V1.A9OA.01A.11R.A41O.07	-0.3729	0.01244	0.113874772	0.328911201	0.200283054	-0.03890016	0.029960269	-0.249422322	-0.203114493	0.248623714	-0.10493299	-0.028681292	0.313943277	0.284839948	-0.010786115	-0.305331812	0.360971969	0.003271295	-0.102185979	-0.122914032	-0.170794438
TCGA.HC.7080.01A.11R.1965.07	-0.3744	-0.0262	0.096767012	0.260174627	0.111286681	-0.046859543	-0.124548783	-0.396935783	-0.266854038	0.179794638	-0.06966895	-0.103300341	0.365352623	0.14853332	-0.150532281	-0.150006463	0.304808039	-0.058196192	-0.178501203	-0.174774077	-0.184877458
TCGA.EJ.5501.01A.01R.1580.07	-0.3449	0.01294	0.151422117	0.32695125	0.144379349	-0.051249041	-0.105560821	-0.236411003	-0.225513908	0.226286858	-0.138114933	-0.02403254	0.389697934	0.245294423	-0.106075115	-0.199721363	0.344121675	-0.038861072	-0.091663399	-0.209048306	-0.164624911
TCGA.HC.8213.01A.11R.A29R.07	-0.4078	0.00858	0.035563015	0.278090011	0.119612094	-0.011858916	-0.076351163	-0.262218401	-0.241388525	0.235236728	-0.249121203	-0.110281732	0.39606142	0.125400695	-0.13920625	-0.190145483	0.272412968	-0.050683671	-0.093254004	-0.195498252	-0.17422096
TCGA.HC.7744.01A.11R.2118.07	-0.3744	-0.0321	0.079140243	0.28680642	0.126370863	-0.039976584	-0.11479082	-0.273215448	-0.260509261	0.197075274	-0.134668448	-0.02467205	0.38067332	0.161550525	-0.116309757	-0.128758173	0.275362305	-0.03875513	-0.120174134	-0.204470893	-0.199935081
TCGA.XK.AAK1.01A.11R.A41O.07	-0.3209	0.0787	0.097564222	0.262533727	0.125072401	-0.051801279	-0.077264119	-0.281610707	-0.233271483	0.182624178	-0.12647869	-0.15109303	0.36284171	0.144699744	-0.138442365	-0.164812524	0.311007846	-0.005483292	-0.101367103	-0.173042047	-0.179280634
TCGA.HC.7747.01A.11R.2118.07	-0.222	0.10429	0.124921105	0.362723643	0.170955981	-0.038305527	0.034058545	-0.284517472	-0.159071639	0.230451318	-0.12765797	-0.051324354	0.382600007	0.264401574	-0.084547937	-0.261288733	0.294751283	0.014096538	-0.055938967	-0.114000352	-0.167888667
TCGA.ZG.A8OW.01A.11R.A37L.07	-0.4105	0.1094	0.143697542	0.346895555	0.174654197	-0.070795508	0.002125211	-0.210757198	-0.160323285	0.197727778	-0.091343385	-0.073733945	0.351229062	0.26966733	-0.048127314	-0.184768422	0.310084165	0.014408417	-0.046950204	-0.176027044	-0.130855751
TCGA.CH.5746.01A.11R.1580.07	-0.3431	-0.0189	0.136364952	0.301508778	0.129137767	-0.045973934	-0.060078933	-0.228617725	-0.261275755	0.240246365	-0.177109182	-0.050564734	0.379291953	0.22352844	-0.124623487	-0.232332877	0.314319544	-0.070805967	-0.110476315	-0.20485524	-0.158026723
TCGA.G9.6384.01A.11R.1789.07	-0.2573	0.0033	0.164991676	0.329084824	0.142745103	-0.021818802	0.012791186	-0.131260655	-0.221225352	0.225307162	0.060140534	-0.049750708	0.372319911	0.253184867	-0.07173254	-0.14378679	0.302873007	0.023415843	-0.023428646	-0.167327588	-0.138850771
TCGA.G9.6367.01A.11R.1789.07	-0.294	0.00263	0.145557342	0.332919358	0.1419821	-0.057928232	-0.047872887	-0.304561904	-0.202944554	0.261362826	0.038312507	-0.106077677	0.376552229	0.282797046	-0.099826059	-0.214096998	0.32675525	-0.029537818	-0.089271971	-0.104308628	-0.13791203
TCGA.YL.A8HO.01A.11R.A36G.07	-0.26	0.10705	0.14889581	0.379664682	0.182937904	-0.030653159	0.053462867	-0.169806807	-0.032560469	0.249371432	0.027591906	-0.046507069	0.362426497	0.283659511	0.007475997	-0.27732124	0.389174336	0.103404565	0.008125666	-0.200035693	-0.131951394
TCGA.KK.A6DY.01A.12R.A311.07	-0.4239	0.03954	0.135458134	0.320362045	0.174941748	-0.043298728	0.006702607	-0.121119042	-0.178257007	0.201738903	-0.057617743	-0.059910555	0.358899492	0.233115246	-0.058526913	-0.187817236	0.334977269	0.00814733	-0.045315839	-0.213757895	-0.124178985
TCGA.KK.A6E0.01A.11R.A311.07	-0.3427	0.02196	0.104444728	0.299387151	0.121803575	-0.012649164	-0.148308102	-0.321358039	-0.260762626	0.185973114	-0.194203627	-0.128711079	0.382053327	0.141228685	-0.137004514	-0.1915566	0.309877523	-0.017476551	-0.123179865	-0.150446703	-0.122975317
TCGA.CH.5767.01A.11R.1789.07	-0.38	-0.0099	0.067564173	0.278326483	0.136033539	-0.045177823	-0.077276159	-0.179644021	-0.244096655	0.227900606	-0.082275605	-0.12779427	0.369814427	0.177306297	-0.110801679	-0.205216374	0.272420436	-0.01272678	-0.069372978	-0.168066677	-0.088530575
TCGA.EJ.5531.01A.01R.1580.07	-0.249	0.06203	0.131607444	0.362687444	0.212461931	-0.054020228	0.022375741	-0.322723596	-0.094925976	0.217439054	-0.031550289	-0.003044251	0.375835296	0.278826151	-0.036741832	-0.223244205	0.320632799	0.024542822	-0.032407655	-0.180808199	-0.098824676
TCGA.VN.A88K.01A.11R.A352.07	-0.4701	0.02459	0.141300385	0.284503699	0.11548684	-0.047721176	-0.081906345	-0.277573888	-0.263503169	0.216988805	-0.011843983	-0.11850447	0.323165205	0.20208333	-0.096361672	-0.153953592	0.320520261	-0.007101279	-0.109741995	-0.187600478	-0.208469511
TCGA.VN.A88M.01A.11R.A352.07	-0.3531	0.0426	0.139908684	0.268347763	0.100423605	-0.013565573	-0.054205003	-0.193070993	-0.296088817	0.177616235	-0.112154609	-0.174084411	0.383635258	0.152253463	-0.133998675	-0.155581333	0.314746822	-0.052744793	-0.101866732	-0.209468771	-0.232832501
TCGA.EJ.5506.01A.01R.1580.07	-0.4206	-0.0029	0.119558966	0.304270027	0.166435012	0.014143844	-0.006122983	-0.269081686	-0.177013889	0.247938474	-0.141402336	-0.088135678	0.361955781	0.262317616	-0.143398015	-0.30681781	0.290221856	-0.06819866	-0.109480162	-0.15195997	-0.148401036
TCGA.XK.AAJU.01A.11R.A41O.07	-0.2984	0.04263	0.179112279	0.338445246	0.159501396	-0.028994127	-0.011490428	-0.351232496	-0.199981698	0.239075572	-0.104100254	-0.053576142	0.353180047	0.263635447	-0.11649284	-0.14904127	0.3369411	-0.006015192	-0.067273139	-0.14956838	-0.169453753
TCGA.XO.A8TA.01A.11R.A36G.07	-0.4376	-0.0374	0.152097816	0.222952594	0.178509207	-0.015855562	-0.118404449	-0.400891883	-0.367524232	0.149090976	-0.324973558	-0.030780573	0.273258635	0.038461329	-0.149016214	-0.500054093	0.237079208	-0.081556217	-0.168207073	-0.310901459	-0.177682814
TCGA.HC.A6AO.01A.11R.A30B.07	-0.2675	0.10408	0.146703394	0.282924775	0.157262811	-0.061719661	0.020049481	-0.307051903	-0.160805076	0.193967894	-0.033974945	-0.1685345	0.38015381	0.184905714	-0.060396751	-0.225808919	0.336468481	0.004637402	-0.059388453	-0.122952364	-0.184249582
TCGA.FC.A5OB.01A.11R.A29R.07	-0.4787	0.00062	0.072711538	0.236313531	0.111820936	0.018712642	-0.160092722	-0.281035127	-0.247454135	0.224694013	-0.304363796	-0.132955297	0.393683798	0.120981557	-0.161181677	-0.241685967	0.302026346	-0.07721517	-0.130027182	-0.185087988	-0.176743664
TCGA.HC.7817.01B.11R.A29R.07	-0.3029	0.06539	0.139393421	0.390374153	0.178500641	-0.007704321	0.044569087	-0.35881102	-0.123286253	0.223444035	-0.04184587	-0.009047306	0.376124311	0.292791906	-0.0162514	-0.237141561	0.357557524	0.041211135	-0.025138958	-0.152083055	-0.098242969
TCGA.KC.A7F6.01A.11R.A33R.07	-0.3801	0.01677	0.149946643	0.31530622	0.133906382	-0.061404091	-0.031188589	-0.250277806	-0.242000665	0.21700994	-0.12546366	-0.077146554	0.365460356	0.194269251	-0.062314492	-0.238925208	0.347910566	-0.000541015	-0.066402925	-0.200371965	-0.206837439
TCGA.KK.A7B4.01A.11R.A32O.07	-0.4252	0.08478	0.121382025	0.286988835	0.150818318	-0.051400221	0.011841494	-0.36819537	-0.214561402	0.192434508	-0.22074453	-0.031765205	0.339243352	0.190023641	-0.10280652	-0.281524199	0.301449088	-0.016755267	-0.111270168	-0.22544078	-0.196371712
TCGA.G9.6366.01A.11R.2118.07	-0.3964	0.04205	0.162685737	0.297414588	0.120395374	-0.042239404	-0.142312173	-0.241888445	-0.277171337	0.179910266	-0.17194833	-0.154204795	0.383101763	0.155150029	-0.168528649	-0.127933666	0.26558282	-0.037746622	-0.10002671	-0.125392504	-0.144929569
TCGA.KK.A59X.01A.11R.A29R.07	-0.4181	0.02079	0.112812658	0.297902519	0.140965388	0.000328293	-0.072553271	-0.314411404	-0.164759687	0.195311882	-0.139035928	-0.137446856	0.395966326	0.18342627	-0.052770981	-0.155023911	0.274575105	0.002608985	-0.108208242	-0.169708792	-0.157509064
TCGA.XK.AAIR.01A.11R.A41O.07	-0.3157	0.03448	0.134314523	0.322809634	0.162569086	0.012315223	-0.017173455	-0.392870072	-0.175278806	0.238930155	-0.117241906	-0.050523805	0.359189689	0.25061911	-0.087458974	-0.307033144	0.356340758	0.00293027	-0.08029171	-0.179725049	-0.153232517
TCGA.V1.A8ML.01A.11R.A37L.07	-0.3215	0.02129	0.106021827	0.303927964	0.131757289	-0.06169626	-0.119129723	-0.222287983	-0.158891422	0.228997848	-0.133162351	-0.162121456	0.387158451	0.21939362	-0.117679514	-0.213726075	0.2737294	-0.022549752	-0.09939207	-0.157607148	-0.194961636
TCGA.KK.A8IB.01A.11R.A36G.07	-0.2492	0.09482	0.143275625	0.342548883	0.206967935	-0.020260771	0.029794597	-0.157699218	-0.195635284	0.184578395	-0.11147092	-0.047423219	0.347783339	0.230182136	-0.007891117	-0.243351413	0.340895532	0.015690233	0.002441416	-0.178470209	-0.126006287
TCGA.J4.A67N.01A.11R.A30B.07	-0.3239	0.11647	0.180125969	0.275476257	0.146472033	-0.004514328	-0.025937765	-0.105418497	-0.191572176	0.161201235	-0.040948631	-0.098340848	0.365572966	0.175844509	-0.050779745	-0.158337148	0.301304566	-0.001939782	-0.082281929	-0.120362094	-0.231158806
TCGA.EJ.5516.01A.01R.1580.07	-0.3406	0.02733	0.128086034	0.332689542	0.186023032	-0.008926256	0.064871359	-0.213916916	-0.123070142	0.226803874	-0.015288518	-0.012293846	0.383379227	0.290607757	-0.07601564	-0.084612779	0.315771327	0.041739298	-0.040573189	-0.163582092	-0.050149952
TCGA.G9.6496.01A.11R.1789.07	-0.2905	0.03839	0.138678521	0.302165351	0.135653707	-0.034109219	-0.082024707	-0.274608984	-0.2743642	0.173047607	-0.15193458	-0.144441291	0.379355992	0.183818984	-0.073490086	-0.091859452	0.311281549	-0.014668077	-0.126004362	-0.171617959	-0.1800524
TCGA.VP.A879.01A.11R.A352.07	-0.2523	-0.0496	0.150039044	0.309289042	0.12206371	-0.020021191	-0.035939651	-0.28656346	-0.270595897	0.23931094	-0.114909276	-0.068445795	0.378748592	0.268173224	-0.143462815	-0.171419949	0.337794531	-0.032923179	-0.089373454	-0.18023463	-0.172134357
TCGA.EJ.A6RC.01A.11R.A32O.07	-0.1023	0.14237	0.13584201	0.362228023	0.185413425	-0.031470394	0.028735865	-0.262036953	-0.06258624	0.205901932	-0.00503975	-0.08534746	0.318043135	0.275040992	-0.076730322	-0.269978433	0.352917311	0.0378052	-0.008750629	-0.177235693	-0.173519407
TCGA.G9.6342.01A.11R.1965.07	-0.3176	-0.0066	0.150942976	0.303925407	0.130817419	-0.0208425	-0.069213165	-0.235331869	-0.263452873	0.221437116	-0.176717151	-0.116120111	0.375824469	0.230642271	-0.092089962	-0.124434999	0.346388511	-0.029866106	-0.096721299	-0.210071376	-0.152841348
TCGA.G9.6354.01A.11R.A311.07	-0.4077	0.03798	0.119280023	0.274571695	0.123814045	-0.011804057	-0.06735059	-0.213215321	-0.264796481	0.212076879	-0.164626669	-0.19817601	0.384622021	0.156748022	-0.100088025	-0.159439455	0.306407814	-0.042463704	-0.107878814	-0.153017157	-0.223069329
TCGA.EJ.A7NK.01A.12R.A352.07	-0.2964	-0.0225	0.172642667	0.341956858	0.129875407	-0.05132107	-0.043538688	-0.296394096	-0.245432592	0.242291245	-0.068481371	-0.063668096	0.365673176	0.266580623	-0.131027324	-0.309526815	0.351343133	-0.034626603	-0.117515107	-0.144963339	-0.16363726
TCGA.KK.A8I5.01A.11R.A36G.07	-0.3793	-0.0044	0.107407757	0.309643131	0.15463475	-0.051748234	-0.022244308	-0.074933015	-0.220678994	0.225825584	-0.064114886	-0.129935866	0.339234415	0.241275165	-0.077784642	-0.214278819	0.333631101	0.022359033	-0.043653887	-0.169574318	-0.173902321
TCGA.VP.A876.01A.11R.A352.07	-0.4982	0.02564	0.026524518	0.261416004	0.119521125	0.005364843	-0.12213188	-0.235786084	-0.28403545	0.179639874	-0.161164724	-0.049984298	0.389451238	0.118973316	-0.141780207	-0.161522893	0.265877735	-0.072465304	-0.121775948	-0.227853591	-0.183248806
TCGA.YJ.A8SW.01A.11R.A37L.07	-0.4781	-0.0309	0.052847979	0.265381383	0.121655578	-0.090084007	-0.090484121	-0.346888567	-0.178136824	0.171439212	-0.230874291	-0.100849421	0.353967748	0.180138961	-0.15033697	-0.28289012	0.263601948	-0.073482622	-0.138345972	-0.213233279	-0.235898075
TCGA.G9.6365.01A.11R.1789.07	-0.3721	0.04374	0.099096757	0.34473964	0.174975799	-0.031808766	-0.005136887	-0.312441086	-0.229793897	0.260226263	-0.11169728	-0.087814489	0.348853925	0.253560578	-0.059909529	-0.189860749	0.354347039	0.018824704	-0.010684258	-0.1958845	-0.103835316
TCGA.2A.A8VO.01A.11R.A37L.07	-0.2756	0.01061	0.141258586	0.327380906	0.143813354	-0.019524924	-0.038425159	-0.258385796	-0.244597833	0.192463067	-0.086898612	-0.157863128	0.353710359	0.225565397	-0.069000245	-0.243666889	0.298961707	-0.020757866	-0.0706733	-0.17775269	-0.234768055
TCGA.EJ.7789.01A.11R.2118.07	-0.4257	-0.0307	0.06392496	0.274515387	0.162396326	-0.00449991	-0.119291632	-0.304864599	-0.18251765	0.211626372	-0.186466468	-0.076202967	0.371496401	0.150483969	-0.112821645	-0.392472924	0.326630191	-0.051267467	-0.144107153	-0.245271849	-0.110405431
TCGA.V1.A8WN.01A.11R.A37L.07	-0.3241	######	0.128239143	0.332198404	0.130500206	-0.053501493	-0.023565335	-0.078832174	-0.226333434	0.235578362	-0.031310791	-0.077291646	0.378067987	0.282637013	-0.095108234	-0.207664175	0.312186673	0.00030706	-0.054854823	-0.20769324	-0.142413415
TCGA.G9.6343.01A.21R.1965.07	-0.2762	0.01781	0.174164205	0.272381594	0.153094362	-0.060284585	-0.090211636	-0.356989888	-0.223097587	0.194773453	-0.150507765	-0.193407886	0.352412882	0.181702257	-0.077747765	-0.178364542	0.294614396	-0.000791878	-0.106251486	-0.137034585	-0.224645348
TCGA.EJ.A8FO.01A.21R.A36G.07	-0.3111	-0.0252	0.153076974	0.316858319	0.125326457	-0.055158733	-0.063853165	-0.363115723	-0.251449927	0.251823167	-0.125586086	-0.113929317	0.366960865	0.221825917	-0.104614334	-0.197362797	0.314465131	-0.040166629	-0.100163175	-0.162063244	-0.137158896
TCGA.HI.7168.01A.11R.2118.07	-0.381	-0.0287	0.170041293	0.336335628	0.135041249	0.029567069	-0.055872129	-0.121988471	-0.238417408	0.200425621	-0.07625308	-0.06404642	0.330470517	0.263269572	-0.004274066	-0.259181779	0.350717636	0.030297226	-0.062035378	-0.189034516	-0.115629206
TCGA.EJ.7785.01A.11R.2118.07	-0.2664	-0.0009	0.122266929	0.332568779	0.175276667	-0.029468703	0.056958225	-0.272652081	-0.102997338	0.223929359	-0.032661422	-0.017083819	0.361821552	0.290581816	-0.065169487	-0.137198347	0.37002894	0.018543779	-0.055314183	-0.182887016	-0.11396467
TCGA.J4.A67M.01A.11R.A30B.07	-0.2261	0.12848	0.186962181	0.33680567	0.154732578	-0.0203582	0.036476297	-0.216953973	-0.161364371	0.191710906	-0.042498093	-0.057567489	0.370493735	0.278496078	-0.045047512	-0.233067148	0.345433411	0.04788805	-0.042037985	-0.139994542	-0.171784228
TCGA.CH.5753.01A.11R.1580.07	-0.458	0.02853	0.071358577	0.29139641	0.167803797	-0.011719586	-0.003794406	-0.07916573	-0.003324722	0.162732575	-0.0769665	-0.113380556	0.288883163	0.233848881	-0.08845866	-0.246108209	0.266177934	-0.031025107	-0.081877772	-0.221922015	-0.133124634
TCGA.KC.A7F5.01A.11R.A33R.07	-0.4635	0.01211	0.154661535	0.25981535	0.084649621	-0.077607009	-0.19682593	-0.352437861	-0.312986344	0.134751427	-0.15441724	-0.169149419	0.392548697	0.124445811	-0.17432181	-0.311163461	0.313811689	-0.08119705	-0.18190168	-0.099732543	-0.143822226
TCGA.HC.A9TH.01A.11R.A41O.07	-0.4693	0.02307	0.130233599	0.341794526	0.173511869	-0.032619801	-0.026397315	-0.368891658	-0.070683822	0.195857762	-0.125969647	-0.092651576	0.354124126	0.235025063	-0.056667496	-0.269066864	0.313172017	-0.004978094	-0.058391826	-0.259244551	-0.1506454
TCGA.2A.AAYF.01A.11R.A41O.07	-0.3331	0.01388	0.123331387	0.297539769	0.126867479	-0.044181099	-0.11576257	-0.27449206	-0.253151096	0.215162452	-0.165530118	-0.122865811	0.366957685	0.147285357	-0.13915396	-0.165682197	0.304021047	-0.059506318	-0.108855513	-0.161791824	-0.227855296
TCGA.KK.A8I7.01A.21R.A36G.07	-0.2569	0.03743	0.164416819	0.34861158	0.159337449	-0.034500422	0.01662984	-0.304556038	-0.164531971	0.213390636	-0.024711382	-0.043485052	0.356740076	0.253038609	-0.085953096	-0.197753549	0.385137353	0.086966337	-0.06759859	-0.133404504	-0.160644846
TCGA.EJ.7797.01A.11R.2263.07	-0.3041	0.03462	0.125046512	0.330994301	0.14878639	-0.031029917	-0.040751931	-0.190371066	-0.242939936	0.19311298	-0.072915984	-0.072079651	0.383690451	0.233790906	-0.117927783	-0.199681636	0.344338687	-0.013991651	-0.081444072	-0.226510045	-0.184614698
TCGA.HC.7230.01A.11R.2118.07	-0.4606	-0.0082	0.110509014	0.271139261	0.11713566	-0.052350043	-0.107280598	-0.189016339	-0.282615762	0.193512402	-0.154678729	-0.081076536	0.392803075	0.15724055	-0.144282899	-0.201869707	0.27615466	-0.0158342	-0.1144481	-0.236804821	-0.20460267
TCGA.XJ.A9DO.01A.11R.A37L.07	-0.2859	0.0033	0.154480036	0.31076295	0.110090284	-0.064150234	-0.023978663	-0.380304194	-0.293776218	0.211493324	-0.139224616	-0.115360128	0.354299758	0.220205008	-0.122512188	-0.181757321	0.344093808	-0.041620833	-0.111495489	-0.159367059	-0.194719863
TCGA.J9.A52B.01A.11R.A26U.07	-0.425	0.02553	0.135594987	0.298048291	0.125453433	-0.029366093	-0.066686717	-0.269934112	-0.251703804	0.178783817	-0.168974579	-0.127619496	0.365482971	0.227732528	-0.106252725	-0.197680795	0.300551662	0.008597516	-0.102592205	-0.262595884	-0.129634979
TCGA.EJ.5511.01A.01R.1580.07	-0.3183	-0.0325	0.108895642	0.288843504	0.162093169	-0.065778192	-0.091891082	-0.350518932	-0.179370346	0.186266224	-0.145511432	-0.064038403	0.323343788	0.234281302	-0.118447691	-0.297108021	0.331676423	-0.049782871	-0.096068648	-0.198326274	-0.220063719
TCGA.EJ.5527.01A.01R.1580.07	-0.3203	0.02874	0.117855054	0.330831749	0.182027319	0.008532321	0.017023779	-0.176311351	-0.08224271	0.240700598	-0.052256824	0.023113709	0.33630844	0.273566329	-0.075900134	-0.263806402	0.318118737	0.016862837	-0.054716159	-0.198941547	-0.059167447
TCGA.YL.A8S9.01A.11R.A37L.07	-0.3855	-0.0282	0.080157937	0.237974978	0.115311108	-0.034759341	-0.143321453	-0.350642505	-0.277794019	0.2225087	-0.245511123	-0.125082137	0.384392137	0.081211003	-0.16231359	-0.345468687	0.331531252	-0.06455783	-0.165495792	-0.199921473	-0.141667955
TCGA.J4.A67O.01A.11R.A30B.07	-0.2162	0.06516	0.199241341	0.285113087	0.141747247	-0.051810693	0.065887904	-0.208018433	-0.194747886	0.192597992	0.014967467	-0.135721496	0.381373856	0.230237381	-0.033127411	-0.235086049	0.326322898	0.014395886	-0.014706385	-0.083362603	-0.169702944
TCGA.V1.A9O9.01A.11R.A41O.07	-0.0817	0.23278	0.094179606	0.368271093	0.153317255	-0.026696196	0.102274004	-0.262688441	-0.007397986	0.210441622	-0.140400499	-0.07942845	0.361884766	0.270909108	-0.072155834	-0.157408272	0.315294853	0.032778844	-0.010608812	-0.190594231	-0.168902835
TCGA.CH.5791.01A.11R.1580.07	-0.3916	-0.0419	0.105823676	0.313089117	0.164739029	-0.00119029	-0.031176288	-0.344288406	-0.194228454	0.1817363	-0.102892595	-0.078457827	0.295329459	0.204836246	-0.092014181	-0.233718143	0.305274205	-0.009784279	-0.09049145	-0.198319651	-0.13519726
TCGA.V1.A8MM.01A.11R.A37L.07	-0.2729	0.10089	0.101617482	0.318800461	0.150156765	-0.046595497	0.021402059	-0.383984196	-0.136649118	0.216800822	-0.140754498	-0.175293581	0.380926983	0.228680764	-0.067871886	-0.380765421	0.347499801	0.044383157	-0.056607535	-0.199507308	-0.22170712
TCGA.FC.7708.01A.11R.2118.07	-0.307	0.03887	0.147373033	0.348993177	0.152767053	-0.054010251	0.043579268	-0.140543413	-0.157619944	0.239631093	-0.016636227	-0.041343808	0.352711161	0.295232338	-0.055628267	-0.247831024	0.342066376	0.045559756	-0.039987392	-0.198693666	-0.135680414
TCGA.ZG.A8QY.01A.11R.A37L.07	-0.3665	-0.0189	0.136450447	0.297560811	0.116943506	0.015186183	-0.044706432	-0.289331224	-0.22959497	0.197245919	-0.058188892	-0.126125084	0.333594888	0.222877312	-0.114789712	-0.305059653	0.326224866	-0.030720085	-0.129812046	-0.217670059	-0.082549664
TCGA.EJ.7328.01A.31R.2118.07	-0.2087	0.1466	0.152906869	0.381920298	0.163708252	-0.024400267	0.084895843	-0.354057868	-0.106267552	0.248871051	-0.114566608	-0.036158031	0.363899495	0.27752065	-0.01878318	-0.175757629	0.360800993	0.033560886	-0.026427476	-0.181579823	-0.147818877
TCGA.EJ.5510.01A.01R.1580.07	-0.3346	0.04929	0.159705485	0.340469381	0.140002518	-0.03481122	0.00173889	-0.236574219	-0.107763907	0.208862857	-0.018089453	-0.043860593	0.358568167	0.251343152	-0.12676562	-0.27802693	0.318214953	-0.010381224	-0.081067096	-0.176956614	-0.159347711
TCGA.EJ.5498.01A.01R.1580.07	-0.2602	0.02338	0.108042215	0.358839928	0.193181862	0.020351519	0.063206652	-0.185877074	-0.154827371	0.246308605	0.02043989	-0.031508781	0.356910577	0.321302792	-0.003157491	-0.204712701	0.323150387	0.030786867	-0.025831663	-0.161042251	-0.084117509
TCGA.KK.A6E6.01A.11R.A311.07	-0.3965	0.07606	0.113311973	0.29658684	0.155143233	-0.0414628	0.007531061	-0.169174894	-0.200827239	0.209028441	0.003126809	-0.074926887	0.337191312	0.256858068	-0.080972592	-0.146499816	0.326905152	0.011234986	-0.046971577	-0.198085094	-0.164186796
TCGA.YL.A9WY.01A.11R.A41O.07	-0.279	0.20765	0.145353344	0.367525647	0.160609164	-0.011980302	0.086322603	-0.30937172	-0.065194242	0.210250892	-0.037452285	-0.021234639	0.319974398	0.285836069	-0.001153701	-0.282267233	0.366228157	0.054403699	-0.009139991	-0.235260118	-0.194925871
TCGA.VN.A88N.01A.11R.A36G.07	-0.4228	0.02537	0.139245641	0.288977103	0.103496384	0.019087087	-0.099789263	-0.236024677	-0.298928374	0.203823522	-0.11321146	-0.173461919	0.391544632	0.164568539	-0.139400037	-0.174223756	0.325411193	-0.013479144	-0.129533273	-0.194428014	-0.183800102
TCGA.YL.A9WK.01A.11R.A37L.07	-0.3212	0.07937	0.15576089	0.306194033	0.167109696	0.013749396	0.015029712	-0.23937655	-0.199312313	0.195423134	0.064089391	-0.181467744	0.308609252	0.2244814	-0.060655667	-0.270392036	0.327340914	0.008292438	-0.047442126	-0.198461559	-0.156299048
TCGA.2A.A8VL.01A.21R.A37L.07	-0.3371	0.0037	0.128665431	0.312204125	0.095215832	-0.063233656	-0.096011059	-0.199423767	-0.155040596	0.217941942	-0.064217628	-0.15777254	0.395229497	0.216601741	-0.126921356	-0.243734835	0.316942353	-0.061628236	-0.075809875	-0.105448139	-0.177173065
TCGA.V1.A9Z7.01A.11R.A41O.07	-0.3524	0.05999	0.108231949	0.327219104	0.184982569	-0.005199382	0.005248948	-0.291995427	-0.146315903	0.215765405	-0.064465635	-0.097027286	0.359372711	0.25540107	-0.046897894	-0.228868211	0.329901882	0.033194442	-0.029876283	-0.216119707	-0.131566718
TCGA.VP.AA1N.01A.31R.A41O.07	-0.4301	0.10721	0.148471323	0.329100148	0.185771285	-0.057307155	0.035193476	-0.161792047	-0.170292355	0.212845926	-0.081647354	-0.139281922	0.360014968	0.212152693	-0.008737835	-0.228658128	0.301788163	0.034041052	0.00938526	-0.129423131	-0.155249747
TCGA.4L.AA1F.01A.11R.A41O.07	-0.1974	0.16286	0.206955061	0.417319057	0.272335676	0.018198597	0.187747378	-0.175419581	0.013580805	0.248543708	-0.00180172	0.027228891	0.344876543	0.342337425	0.114680335	-0.167557527	0.389200616	0.076870072	0.099797116	-0.116411135	-0.080179407
TCGA.EJ.5526.01A.01R.1580.07	-0.3632	0.00899	0.096808991	0.324566295	0.161292587	-0.01983056	0.047037254	-0.244433495	-0.054822627	0.245747208	-0.040803296	-0.011600969	0.373083349	0.279138393	-0.069556144	-0.230272205	0.326373632	0.012665319	-0.086905428	-0.190663235	-0.14203653
TCGA.G9.6333.01A.12R.1965.07	-0.3293	0.03559	0.131296047	0.303044014	0.142325379	-0.072602678	-0.022176516	-0.153066094	-0.164496598	0.196416783	-0.036006847	-0.129582118	0.381122393	0.227018894	-0.063731354	-0.25758634	0.305479575	0.037633158	-0.047348698	-0.103979166	-0.098405127
TCGA.KC.A7FA.01A.21R.A33R.07	-0.3514	0.05031	0.150181005	0.289110147	0.146340518	-0.054171384	-0.051994781	-0.246926849	-0.241621582	0.191736205	-0.131634276	-0.177809416	0.322902611	0.187861166	-0.089172975	-0.141199098	0.309723481	0.016250458	-0.071949835	-0.192677962	-0.19519582
TCGA.HC.7736.01A.11R.2118.07	-0.3683	0.00667	0.151597303	0.338416174	0.195899758	-0.052431206	-0.025536438	-0.103604192	-0.148868326	0.161002792	0.097464719	-0.071167732	0.353488474	0.260655428	-0.066258067	-0.263304244	0.295732744	0.034817545	-0.05546169	-0.16106288	-0.112780104
TCGA.ZG.A9ND.01A.11R.A41O.07	-0.482	0.05143	0.106693805	0.271498194	0.128063115	-0.074749546	-0.100656632	-0.172868399	-0.254989859	0.170224948	-0.083497834	-0.183622438	0.375623975	0.149187836	-0.135978221	-0.191489695	0.278262301	-0.014906834	-0.109648466	-0.151425094	-0.172181969
TCGA.G9.6373.01A.11R.1789.07	-0.3151	0.05869	0.13273387	0.318911376	0.142704504	-0.027837148	-0.029379898	-0.237215067	-0.226093167	0.188149052	-0.166998545	-0.093328945	0.372235218	0.244707377	-0.046618537	-0.117894407	0.323917015	-0.014451873	-0.070655843	-0.164007182	-0.194422087
TCGA.VP.A878.01A.31R.A352.07	-0.2759	0.01166	0.173133639	0.3580147	0.142367766	-0.051491023	0.011979429	-0.325201081	-0.190679224	0.203479239	0.00948949	-0.060231308	0.381737579	0.262620029	-0.009149056	-0.192594237	0.341301347	0.056149113	-0.046026243	-0.162806947	-0.106103346
TCGA.J9.A52C.01A.11R.A26U.07	-0.2835	0.10886	0.170699706	0.37891483	0.164556635	-0.022905184	0.093078037	-0.094491755	-0.125388141	0.245189327	0.119461157	-0.034708427	0.339844267	0.318418162	0.04782479	-0.270866369	0.37288044	0.092024097	-0.001519558	-0.216266978	-0.087960818
TCGA.YL.A8HL.01A.11R.A36G.07	-0.38	-0.0278	0.111489569	0.257320012	0.116128547	-0.033283182	-0.092458976	-0.216340941	-0.239661096	0.19391195	-0.218389574	-0.135667717	0.386723846	0.153885387	-0.126245273	-0.178082667	0.302118061	-0.041366894	-0.137481692	-0.252564173	-0.156590486
TCGA.ZG.A9LZ.01A.11R.A41O.07	-0.3295	0.07602	0.125847236	0.299223047	0.159744361	-0.032815423	0.004223045	-0.210910053	-0.156796149	0.265547381	-0.177494692	-0.10627059	0.318957289	0.206093119	-0.044913773	-0.182035078	0.314921485	-0.011785649	-0.066884475	-0.19546117	-0.196563377
TCGA.J9.A8CP.01A.11R.A352.07	-0.2698	-0.0085	0.119911252	0.303878837	0.157576684	-0.067683503	-0.052670286	-0.237315275	-0.146498112	0.2474447	0.099339536	-0.087269888	0.364798563	0.238757558	-0.085898199	-0.174509364	0.349903788	0.019395541	-0.065306378	-0.165868662	-0.107063879
TCGA.FC.A6HD.01A.11R.A31N.07	-0.3534	0.06437	0.142040337	0.237606721	0.099438931	-0.04064922	-0.12584694	-0.303549545	-0.32126657	0.183243543	-0.095438872	-0.06500146	0.379810801	0.102514476	-0.177159135	-0.338875054	0.287982124	-0.087344902	-0.161236977	-0.208938393	-0.204271964
TCGA.G9.6351.01A.21R.1965.07	-0.3196	-0.0004	0.161435765	0.300417026	0.112501499	-0.047751694	-0.042853247	-0.252120064	-0.276575292	0.204250635	-0.09175266	-0.116753828	0.384063942	0.217062482	-0.118201092	-0.120221858	0.305054792	-0.053189269	-0.081071711	-0.17579636	-0.158202605
TCGA.EJ.5532.01A.01R.1580.07	-0.4325	0.00127	0.096911615	0.307175809	0.141929231	-0.019491319	-0.081001827	-0.280631564	-0.191621699	0.239467983	-0.112852946	-0.117750243	0.34540566	0.199576366	-0.114092341	-0.297924544	0.304680656	-0.022254759	-0.093974031	-0.181418364	-0.198861852
TCGA.EJ.5525.01A.01R.1580.07	-0.4322	-0.0416	0.080644677	0.310157312	0.228088894	-0.014910312	0.006891965	-0.171810726	-0.12656993	0.21272685	-0.166659085	0.021686243	0.29603584	0.161999473	0.004598734	-0.173716719	0.315101505	0.020268416	-0.049751266	-0.232473513	-0.103540221
TCGA.ZG.A9L9.01A.11R.A41O.07	-0.3285	0.12906	0.062014518	0.345800551	0.181455474	0.049595593	0.048720788	-0.158174353	-0.148480704	0.203086585	-0.017411094	-0.033659709	0.322348481	0.267653554	0.001770426	-0.284419521	0.353482391	0.075546773	-0.008367828	-0.21805192	-0.177491317
TCGA.EJ.A65F.01A.21R.A311.07	-0.4027	0.04982	0.146262019	0.298343439	0.14422389	0.023204783	-0.013112556	-0.089607888	-0.200454084	0.230054916	-0.040618494	-0.087242983	0.330712296	0.204289144	-0.098484627	-0.200041683	0.330018809	-0.025964694	-0.077033494	-0.222510619	-0.133795504
TCGA.2A.A8VX.01A.11R.A37L.07	-0.4869	-0.0182	0.007706815	0.295467725	0.109739453	0.008155655	-0.228175706	-0.319425661	-0.161992631	0.142871633	-0.081531739	-0.125232954	0.374718676	0.149287683	-0.152278432	-0.420005262	0.304235296	-0.07352914	-0.152579854	-0.209353652	-0.185901738
TCGA.KK.A8I4.01A.11R.A36G.07	-0.1953	0.18709	0.167371674	0.39859917	0.174667201	-0.043390654	0.121108933	-0.211933181	-0.057559511	0.213237155	-0.03038971	-0.04036049	0.312377043	0.259383923	0.020340179	-0.07934196	0.364662483	0.098608362	0.032556127	-0.162316531	-0.141638334
TCGA.KK.A8IA.01A.11R.A36G.07	-0.4389	-0.019	0.111056742	0.284426571	0.12715119	-0.034515688	-0.067246378	-0.361808962	-0.244262987	0.198120844	-0.260739696	-0.146118495	0.361628364	0.177088341	-0.115772927	-0.233319305	0.29751029	-0.035945828	-0.129494194	-0.230326797	-0.174695707
TCGA.Y6.A9XI.01A.11R.A41O.07	-0.4411	0.00191	0.184721602	0.287740437	0.115413589	-0.034452534	-0.09062176	-0.360630541	-0.259392177	0.163960317	-0.092373763	-0.156475331	0.370031816	0.18099254	-0.13027758	-0.292154767	0.286743368	-0.030814797	-0.128256246	-0.204168346	-0.190586442
TCGA.VN.A88I.01A.11R.A352.07	-0.2457	0.06212	0.188455101	0.374465755	0.172322077	0.005133644	0.049215178	-0.276586604	-0.160264481	0.24099139	0.001779124	-0.076800837	0.315557644	0.315363377	-0.006298122	-0.298794501	0.400074002	0.06796736	0.035275503	-0.170999296	-0.117093035
TCGA.M7.A71Z.01A.12R.A32O.07	-0.5142	-0.0333	0.114473073	0.213051963	0.117169058	-0.079178745	-0.150355921	-0.29842054	-0.320640254	0.194557097	-0.298496395	-0.104080279	0.349993283	0.037409865	-0.184748194	-0.130195079	0.276529073	-0.052091309	-0.13533042	-0.259855049	-0.298283673

TCGA.EJ.A46E.01A.31R.A250.07	-0.3418	-0.0155	0.18118949	0.340724394	0.159322829	-0.063314459	0.003599694	-0.061755167	-0.200683411	0.256505077	0.044126633	0.021221547	0.347952922	0.292119853	-0.069047131	-0.262941031	0.357529531	0.011258672	-0.033720173	-0.119911677	-0.081632057
TCGA.CH.5788.01A.11R.1580.07	-0.371	-0.0336	0.033295215	0.273002234	0.20356181	-0.054230386	-0.159789214	-0.342297703	-0.181114633	0.176610929	-0.277092561	0.017865599	0.292911679	0.111561278	-0.105892447	-0.130022233	0.275673833	-0.097188397	-0.116449412	-0.181633281	-0.113522451
TCGA.KK.A7AU.01A.11R.A32O.07	-0.4988	-0.0053	0.110130016	0.221625816	0.112680211	-0.030998464	-0.114900211	-0.397890034	-0.301800573	0.19592958	-0.334623278	-0.143798027	0.363691828	0.103754908	-0.152321182	-0.197394721	0.304821985	-0.087008988	-0.171315102	-0.23711719	-0.235475419
TCGA.CH.5740.01A.11R.1580.07	-0.2819	0.04733	0.064201942	0.284927436	0.165632656	0.068842458	-0.017307697	-0.236119641	-0.152674273	0.201014854	-0.176222162	-0.075536143	0.365737046	0.200124078	-0.145842976	-0.215325308	0.315272797	-0.045944769	-0.087495013	-0.195543584	-0.137639887
TCGA.KK.A7B2.01A.12R.A32O.07	-0.2029	0.1908	0.215216398	0.369139663	0.17282022	-0.021347455	0.122214517	-0.181146483	-0.136254104	0.220476929	-0.010499128	-0.033851706	0.360626357	0.260563654	-0.011892372	-0.175694139	0.344780146	0.090968509	0.001262875	-0.133089643	-0.167504582
TCGA.HC.8266.01A.11R.2263.07	-0.0999	0.3041	0.131672627	0.406737032	0.185800161	-0.011204155	0.185257422	-0.272634118	0.036925925	0.209785613	-0.042499068	-0.021698096	0.37930875	0.33248963	-0.020060083	-0.305483254	0.362836668	0.08737814	0.028949087	-0.17626706	-0.112845802
TCGA.KC.A4BV.01A.31R.A26U.07	-0.4033	0.06863	0.098612713	0.339360443	0.142839503	-0.022741818	-0.000444312	-0.254599638	-0.159771321	0.205713993	-0.104193503	-0.106480863	0.305963547	0.262751085	-0.076597124	-0.310676378	0.342043726	-0.015525467	-0.070197076	-0.207558396	-0.167677942
TCGA.XJ.A9DI.01A.11R.A37L.07	-0.2893	0.16114	0.184829379	0.392939755	0.173380662	-0.023237093	0.105606632	-0.255369518	-0.113990919	0.219112534	-0.072556301	-0.011959233	0.341550131	0.323650684	-0.013769397	-0.251470759	0.371489449	0.02039812	-0.019646831	-0.157371609	-0.142389789
TCGA.HC.A4ZV.01A.11R.A26U.07	-0.2515	0.14304	0.174725425	0.314982164	0.151352754	0.025169603	0.039308846	-0.197380046	-0.046301311	0.179037549	-0.070740688	-0.046694135	0.32683189	0.219384249	-0.048273012	-0.296072976	0.347263326	-0.002543163	-0.064528384	-0.213564502	-0.118249127
TCGA.HI.7169.01A.11R.2118.07	-0.2216	0.00792	0.15655012	0.3171281	0.148345685	0.049362991	-0.075124245	-0.321996104	-0.274258855	0.199982037	-0.074873872	-0.195425449	0.361506012	0.258019977	-0.043617302	-0.152270602	0.327275455	0.006515125	-0.043195375	-0.133296247	-0.201333557
TCGA.G9.7509.01A.11R.A41O.07	-0.3554	-0.0017	0.126190425	0.292523545	0.119691963	-0.057141668	-0.045283902	-0.287193155	-0.261094576	0.211891539	-0.147097996	-0.116020674	0.383184661	0.224761525	-0.13163192	-0.198589828	0.300391457	-0.048139928	-0.111136452	-0.19641177	-0.200995555
TCGA.KC.A7FE.01A.12R.A33R.07	-0.2119	-0.0161	0.157355827	0.342779334	0.165123627	-0.034241107	-0.009914546	-0.292245438	-0.225149806	0.245006023	-0.025277483	-0.07902033	0.361733015	0.282124062	-0.054142629	-0.276744994	0.357342264	0.014609804	-0.051825812	-0.164454017	-0.164569642
TCGA.XK.AAJA.01A.11R.A41O.07	-0.3152	-0.0138	0.123029707	0.290177879	0.111225447	-0.104981954	-0.090687439	-0.276124925	-0.107273123	0.158575221	-0.069233286	-0.093597193	0.375264083	0.173810082	-0.133628868	-0.236037382	0.302204897	-0.036881254	-0.11950913	-0.17581257	-0.202404372
TCGA.G9.7521.01A.11R.2263.07	-0.3405	0.16425	0.08334665	0.363264157	0.195150435	-0.008254657	0.072513777	-0.183459225	-0.114990281	0.189823329	0.037184682	-0.147068396	0.337395764	0.242128621	-0.069308908	-0.30415539	0.287000596	0.040890648	-0.023886222	-0.227208908	-0.112371838
TCGA.EJ.A8FN.01A.11R.A352.07	-0.3058	0.07142	0.096646502	0.348614547	0.160393753	0.004545378	-0.039128442	-0.278133201	-0.192773307	0.205820763	-0.114897836	-0.179939589	0.355401429	0.23580021	-0.066606633	-0.28426035	0.301767768	0.000432908	-0.036861145	-0.153665781	-0.211572038
TCGA.EJ.5499.01A.01R.1580.07	-0.2897	0.07352	0.120978427	0.351349046	0.150750128	0.000185133	0.006198141	-0.34409208	-0.166789382	0.27214764	-0.092138579	0.068793166	0.366974773	0.253720492	-0.084062816	-0.196982615	0.326606411	0.002239438	-0.075331877	-0.176066656	-0.105562281
TCGA.EJ.A46F.01A.31R.A250.07	-0.4536	0.02519	0.092810966	0.211521685	0.109663151	0.012471352	-0.105768951	-0.404779818	-0.321536555	0.17328924	-0.247508779	-0.120531689	0.387538161	0.106144531	-0.110733889	-0.16674517	0.307198386	-0.081870335	-0.16003076	-0.252430992	-0.243358295
TCGA.EJ.8470.01A.11R.2403.07	-0.1766	0.24028	0.111968184	0.367687269	0.177736492	0.035169898	0.125040971	-0.219418495	-0.024472927	0.187944281	-0.102816853	-0.108287331	0.330781047	0.269881785	-0.046140302	-0.26960846	0.311900823	0.048113478	-0.015667564	-0.193141543	-0.12832952
TCGA.FC.7961.01A.11R.A29R.07	-0.4215	-0.0019	0.104670943	0.319534551	0.20987437	-0.014040617	0.000509084	-0.362788658	-0.154401682	0.206440399	-0.137388963	-0.084368524	0.315856405	0.297483182	-0.060576724	-0.208073814	0.284032272	-0.043016932	-0.07175322	-0.204269957	-0.14888317
TCGA.YL.A8HK.01A.11R.A36G.07	-0.3565	0.01776	0.190825811	0.346630185	0.14777365	-0.053143031	-0.002439124	-0.218894313	-0.223527641	0.198780118	-0.041299229	-0.053246584	0.344742716	0.239351492	-0.066409037	-0.167387453	0.350578959	-0.018569078	-0.05764952	-0.197539215	-0.17607752
TCGA.HC.7748.01A.11R.2118.07	-0.3315	0.02465	0.132453516	0.319317366	0.164091306	-0.041629744	0.036627068	-0.139371208	-0.098138097	0.208407973	0.007581915	-0.037054611	0.384531618	0.270104784	-0.081171261	-0.158462253	0.308052946	0.013818244	-0.054239901	-0.19690364	-0.125737317
TCGA.EJ.7317.01A.31R.2118.07	-0.3285	-0.0147	0.133084831	0.284509892	0.12206156	-0.076213591	-0.091318681	-0.337696252	-0.241717382	0.203915158	-0.164909709	-0.143791922	0.394819483	0.189573083	-0.133907537	-0.275467135	0.270179742	-0.027820683	-0.135262578	-0.192723442	-0.166816113
TCGA.SU.A7E7.01A.22R.A33R.07	-0.2962	0.05721	0.167732136	0.325520347	0.150094639	-0.04924742	-0.00242399	-0.096252516	-0.188001858	0.200132941	0.043864558	-0.106081303	0.339808415	0.26450316	-0.088562952	-0.251071669	0.350575733	0.014031381	-0.046001669	-0.175833661	-0.15994537
TCGA.XJ.A83F.01A.11R.A352.07	-0.341	-0.0058	0.136126645	0.263077611	0.130379175	-0.039842614	-0.046069571	-0.359464856	-0.2523498	0.211596109	-0.110595001	-0.11723735	0.350242463	0.170731119	-0.10126108	-0.19867603	0.329146289	-0.041312997	-0.080272802	-0.205851427	-0.226080642
TCGA.2A.AAYO.01A.11R.A41O.07	-0.0557	0.16837	0.110175549	0.365051349	0.194063404	-0.028985916	0.020687466	-0.219287826	0.021675208	0.207760638	0.009458827	-0.158107704	0.342554332	0.22344647	-0.055899786	-0.276547748	0.340178164	0.038021473	-0.023166475	-0.185222796	-0.203078659
TCGA.CH.5762.01A.11R.1580.07	-0.2619	0.08305	0.116724765	0.352954374	0.183701724	-0.017559957	0.013772751	-0.027455127	-0.083095822	0.232333958	0.037271395	-0.037148957	0.301281625	0.288081499	-0.070870308	-0.288959663	0.327061778	0.035580256	-0.006550454	-0.192966864	-0.129934042
TCGA.G9.A9S7.01A.11R.A41O.07	-0.3606	0.04542	0.174458737	0.270088889	0.131720283	-0.012886338	-0.056813381	-0.308248041	-0.246733055	0.198326863	-0.200377632	-0.14773252	0.358744253	0.1629779	-0.121770327	-0.169422577	0.334089763	-0.019437393	-0.118019727	-0.205200879	-0.19705892
TCGA.J4.AAU2.01A.11R.A41O.07	-0.3427	0.04405	0.143846738	0.298422788	0.118931177	-0.073784118	-0.084502984	-0.18954729	-0.229048884	0.194636386	-0.094807317	-0.15712219	0.360475202	0.186996469	-0.114096457	-0.232288098	0.284377485	0.003430779	-0.062354352	-0.152505966	-0.170599886
TCGA.EJ.AB20.01A.12R.A41O.07	-0.2168	0.16959	0.159457607	0.343018922	0.191077832	-0.006486136	0.033973035	-0.184131548	-0.136890718	0.253401402	0.017055096	-0.089577753	0.323083772	0.291202473	0.001623421	-0.397382029	0.405809394	0.073855019	0.017793808	-0.163206576	-0.092400615
TCGA.KK.A6E8.01A.11R.A31N.07	-0.4637	0.03264	0.08537455	0.279345852	0.126303191	-0.046925966	-0.130658514	-0.363899071	-0.249822335	0.198485049	-0.155843485	-0.236097434	0.384744061	0.0907271	-0.126166234	-0.328955503	0.321875286	-0.029867311	-0.123387814	-0.188080425	-0.183940048
TCGA.EJ.A65E.01A.11R.A29R.07	-0.4301	0.03374	0.121714811	0.298301537	0.107427745	-0.037808655	-0.116828976	-0.310429312	-0.254174838	0.180964022	-0.087156679	-0.187785437	0.365884316	0.168370095	-0.143523151	-0.227032122	0.279082508	-0.064377766	-0.148333098	-0.179255941	-0.137303774
TCGA.ZG.A9L0.01A.11R.A41O.07	-0.3742	0.10776	0.152261828	0.381640245	0.203291676	0.007104634	0.069828063	-0.240368221	-0.111235864	0.21139547	-0.002569054	-0.036742683	0.345033325	0.274753593	0.0379831	-0.278124837	0.36542777	0.065045081	0.008600693	-0.201714064	-0.113243177
TCGA.YL.A9WL.01A.11R.A41O.07	-0.4627	-0.0381	0.13313423	0.257415598	0.116733046	-0.077243222	-0.112384391	-0.352536299	-0.333442242	0.228694816	-0.261582958	-0.103511105	0.394394329	0.135076558	-0.150071316	-0.203756061	0.315869948	-0.07598419	-0.144768889	-0.234810886	-0.228956409
TCGA.CH.5772.01A.11R.1580.07	-0.3081	0.04183	0.072028124	0.296835294	0.189912434	-0.009166469	-0.044932414	-0.22666856	-0.080890592	0.215292421	-0.014695581	-0.141314023	0.341754167	0.2153272	-0.054917674	-0.231911399	0.26669543	-0.016079229	-0.087625934	-0.178158231	-0.134336626
TCGA.ZG.A9L2.01A.31R.A41O.07	-0.0931	0.16678	0.122716316	0.359174243	0.187772224	0.051158251	0.08147115	-0.176740312	-0.094502829	0.220066144	-0.04959867	-0.030445093	0.328071776	0.300988081	-0.028168204	-0.217526167	0.34343193	0.079419779	0.022879826	-0.231903358	-0.119877744
TCGA.HC.8216.01A.11R.A29R.07	-0.3052	0.07757	0.145332675	0.33600166	0.166356116	-0.007993762	-0.000818868	-0.362680352	-0.095960888	0.20862837	-0.104905621	-0.084648607	0.339631298	0.229223934	-0.056990979	-0.337688847	0.299579576	0.004681423	-0.081403327	-0.156857799	-0.181318756
TCGA.M7.A722.01A.12R.A36G.07	-0.4236	0.03596	0.054341295	0.20148012	0.184832544	-0.053338875	-0.093919039	-0.323935696	-0.106847682	0.20197833	-0.20344324	-0.093749567	0.330744103	0.123570001	-0.130363114	-0.493694663	0.287852317	-0.049595344	-0.1299553	-0.198335486	-0.177556903
TCGA.YL.A8SJ.01B.11R.A37L.07	-0.3213	-0.032	0.135095888	0.317833887	0.136466594	-0.033286527	-0.084222578	-0.2608852	-0.263535794	0.217099356	-0.166854284	-0.070989487	0.317011899	0.274900295	-0.059042665	-0.23026118	0.340071385	-0.014399041	-0.085063695	-0.25877095	-0.134718896
TCGA.J9.A8CK.01A.11R.A352.07	-0.3757	-0.0177	0.174153595	0.332856772	0.152307192	-0.03407597	0.055525503	-0.174000389	-0.22346081	0.22489687	0.01198852	-0.039195799	0.360307143	0.236640717	-0.031518348	-0.192368277	0.345522286	0.016220002	-0.080606465	-0.19721894	-0.188640098
TCGA.EJ.7792.01A.11R.2118.07	-0.2152	0.07011	0.130393403	0.360484881	0.195573554	0.021569974	0.033868385	-0.230421918	-0.048223841	0.226260074	-0.02442412	-0.07243614	0.311628741	0.281447688	-0.044320057	-0.195243047	0.33890066	0.051684084	0.029060747	-0.168883895	-0.120758772
TCGA.G9.6332.01A.11R.1789.07	-0.443	0.02356	0.096303527	0.263825044	0.139111411	-0.026267673	-0.109477757	-0.274512254	-0.271340397	0.206701665	-0.226331951	-0.138913923	0.365877722	0.124645042	-0.140846487	-0.176201137	0.323271777	-0.057005186	-0.118951662	-0.259485293	-0.249998136
TCGA.EJ.7784.01A.11R.2118.07	-0.3858	-0.0075	0.036301423	0.277722616	0.139885928	-0.042527171	-0.056838978	-0.292942105	-0.192451079	0.18157577	-0.246265612	-0.030467939	0.345618218	0.189027146	-0.148622663	-0.19442351	0.291371194	-0.090425888	-0.107960888	-0.19550072	-0.186806421
TCGA.YL.A8SR.01B.11R.A37L.07	-0.3696	-0.0027	0.148311801	0.287544338	0.115029919	0.013573222	-0.061719175	-0.255138711	-0.297938485	0.171233819	-0.048329641	-0.194659241	0.354437212	0.190559721	-0.105295325	-0.117006816	0.337322751	0.015289059	-0.110562323	-0.148846522	-0.17340383
TCGA.J4.A83M.01A.11R.A352.07	-0.3906	0.06092	0.091054951	0.301578541	0.130323094	-0.075314646	-0.066264646	-0.230300918	-0.180321093	0.170178991	-0.074444039	-0.096375758	0.381939558	0.234180572	-0.109419267	-0.194558747	0.312293684	-0.007739208	-0.080885832	-0.184969023	-0.218037542
TCGA.J9.A52E.01A.11R.A26U.07	-0.3688	0.09435	0.138197674	0.325066869	0.171489253	0.027053804	0.035222757	-0.358839816	-0.088488042	0.17341367	-0.216593994	0.049146826	0.299772847	0.239311492	-0.001381508	-0.343633185	0.307950702	-0.022502937	-0.025538775	-0.180023264	-0.074458222
TCGA.KK.A8IK.01A.11R.A36G.07	-0.4459	-0.0015	0.11257774	0.300650579	0.129900505	-0.02212247	-0.161287449	-0.426809481	-0.153532033	0.147985948	-0.160928846	-0.089275341	0.358897141	0.132901163	-0.080557504	-0.349182854	0.299924487	-0.063733967	-0.147309862	-0.114430579	-0.205026661
TCGA.KK.A7B0.01A.11R.A32O.07	-0.1297	0.2113	0.185794997	0.346430595	0.184209973	-0.055944734	0.062632893	-0.33173871	-0.100039618	0.17640662	-0.072098116	-0.04913282	0.373346277	0.250756388	-0.045762594	-0.226967897	0.316266251	0.028403101	-0.008983688	-0.145934561	-0.222837134
TCGA.G9.A9S0.01A.11R.A41O.07	-0.3296	0.12033	0.061632458	0.336662113	0.133047939	-0.036964581	-0.08096764	-0.405559903	-0.221366571	0.181863442	-0.231821818	-0.045448179	0.382337183	0.158878719	-0.095460597	-0.22858212	0.278748299	-0.0204275	-0.062653596	-0.126390514	-0.211912998
TCGA.CH.5769.01A.11R.1580.07	-0.4004	-0.0014	0.103696701	0.341632775	0.171231657	-0.036507415	-0.028362713	-0.143098468	-0.143323071	0.216241033	-0.009905684	0.009505291	0.348883356	0.262443037	-0.067509526	-0.199124054	0.30998111	-0.005735424	-0.084756392	-0.251191075	-0.136334746
TCGA.YL.A8SK.01B.21R.A37L.07	-0.3927	0.03785	0.163475242	0.349203541	0.156279647	-0.014750679	0.034344127	-0.144279815	-0.172172542	0.217150655	0.003150096	-0.082228968	0.334340351	0.335418499	-0.0251827	-0.283673236	0.371925399	0.013465634	-0.013012464	-0.145751822	-0.107858276
TCGA.EJ.7786.01A.11R.2118.07	-0.3355	-0.0102	0.12764274	0.317488103	0.162861062	-0.060377938	-0.098209208	-0.268590657	-0.232126645	0.206840655	-0.123524924	-0.127006293	0.372184934	0.223831233	-0.119482163	-0.20754835	0.300560348	-0.029509487	-0.078670156	-0.157319388	-0.163240487
TCGA.VP.A875.01A.31R.A352.07	-0.496	-0.0031	0.07243392	0.235429437	0.121508501	-0.039182416	-0.086093439	-0.333024617	-0.281974001	0.197215801	-0.327528143	-0.098055041	0.378257351	0.101534218	-0.16790431	-0.217273948	0.27697754	-0.057946183	-0.160944533	-0.208819647	-0.21880188
TCGA.EJ.A46D.01A.21R.A32Y.07	-0.3367	0.01178	0.164459716	0.320935506	0.124523334	-0.029365604	-0.016726236	-0.355952874	-0.204238835	0.228311393	-0.114318073	-0.060009416	0.355734348	0.246541629	-0.131578054	-0.220204719	0.330472488	-0.025868004	-0.092124978	-0.166299825	-0.215400785
TCGA.J4.A67L.01A.11R.A30B.07	-0.2858	0.17756	0.178774457	0.309485113	0.130198594	-0.04663322	0.009776549	-0.22509458	-0.19593363	0.205095928	-0.132132993	-0.070955702	0.380409576	0.233846919	-0.085296022	-0.239561805	0.310589978	0.021468485	-0.031890218	-0.151470549	-0.184468646
TCGA.G9.6363.01A.21R.1789.07	-0.3554	0.07045	0.099624867	0.317681437	0.155886068	-0.006681838	-0.054506492	-0.349747711	-0.207418677	0.255547089	-0.001590529	-0.113926117	0.355587727	0.203654276	-0.070734315	-0.193650222	0.273560261	-0.048090909	-0.046627991	-0.253902827	-0.155072667
TCGA.HC.A9TE.01A.11R.A41O.07	-0.2573	0.12342	0.200188078	0.380563681	0.175165462	-0.037843787	0.025179466	-0.254643299	-0.101743479	0.200986718	-0.047385693	-0.079958566	0.386717884	0.248801507	0.002446505	-0.31355685	0.381221412	0.0571282	-0.012463439	-0.133861485	-0.149442691
TCGA.J4.A6G3.01A.11R.A311.07	-0.2309	0.12656	0.159289952	0.389450775	0.190242522	-0.048631227	0.084138907	-0.084059273	-0.121072831	0.196383151	0.021482909	-0.02289628	0.328514884	0.295659017	-0.006950117	-0.174227868	0.339971149	0.070478156	0.004966015	-0.137250332	-0.063778902
TCGA.KK.A7B3.01A.11R.A33R.07	-0.3255	0.11758	0.165643464	0.349746533	0.160250055	-0.022028399	-0.009065756	-0.229931229	-0.140862688	0.173006916	-0.072701043	-0.122399005	0.355825857	0.26358477	-0.049057391	-0.189071466	0.350602891	0.027128154	-0.061423324	-0.184411152	-0.273892731
TCGA.YL.A8HJ.01A.11R.A36G.07	-0.2881	0.07971	0.118558794	0.352195624	0.1872286	0.005765438	0.097991011	-0.284424531	-0.147977318	0.211774251	0.002817239	-0.055243331	0.361152381	0.277715772	-0.003160279	-0.231730648	0.378779096	0.052651473	-0.002836845	-0.203138425	-0.150194596
TCGA.ZG.A9NI.01A.11R.A41O.07	-0.4165	0.00523	0.133280535	0.338264154	0.192172887	-0.020858805	-0.028093692	-0.218146352	-0.212558385	0.209987359	-0.040219536	-0.132600931	0.336481723	0.254680556	-0.012320142	-0.347127461	0.321970515	0.047701437	-0.0574993	-0.160024129	-0.175240987
TCGA.VN.A88R.01A.11R.A36G.07	-0.4076	0.00539	0.136755618	0.279443459	0.116880774	-0.019016829	-0.059425582	-0.341886192	-0.192570114	0.153199433	-0.098143556	-0.197973294	0.371859204	0.187052376	-0.113274926	-0.13782739	0.31863823	0.004611692	-0.138302248	-0.071711117	-0.185380724
TCGA.EJ.5530.01A.01R.1580.07	-0.4005	-0.0136	0.092229895	0.289502651	0.147820014	-0.046225779	-0.056544659	-0.23989655	-0.243890612	0.21278186	-0.099551884	-0.052038703	0.368377441	0.205835103	-0.11111534	-0.193531057	0.306212827	-0.021111146	-0.092129399	-0.225385844	-0.21629303
TCGA.HC.7752.01A.11R.2118.07	-0.2571	0.00291	0.221983005	0.273105778	0.123963139	-0.076756431	-0.055455472	-0.365915775	-0.259824476	0.160052795	-0.163919377	-0.189393275	0.371762883	0.133837313	-0.098783914	-0.189617724	0.337562455	-0.038051364	-0.148570319	-0.198187891	-0.300482541
TCGA.EJ.5521.01A.01R.1580.07	-0.3354	0.02662	0.140839855	0.316226334	0.168474115	-0.055998348	-0.05119823	-0.351872761	-0.220419203	0.221604677	-0.105740618	-0.086899884	0.396251934	0.20544783	-0.078316415	-0.115122995	0.319904645	0.004106134	-0.07979687	-0.148663093	-0.128830517
TCGA.YL.A9WX.01A.21R.A41O.07	-0.2481	0.04514	0.158147581	0.353528757	0.156896797	-0.033074078	0.061837821	-0.092561404	-0.173766476	0.230463212	0.063566676	-0.042106613	0.347871093	0.281446743	-0.081216263	-0.245363232	0.346793381	0.016997364	-0.030156779	-0.152403942	-0.165565054
TCGA.KK.A6E5.01A.11R.A311.07	-0.2396	0.05729	0.13512313	0.31173096	0.145443417	-0.054063966	-0.059049719	-0.39354208	-0.210578831	0.185959769	-0.14246406	-0.113291565	0.356006488	0.213864952	-0.104709998	-0.197771078	0.322327452	-0.004158143	-0.092422779	-0.154715748	-0.198914391
TCGA.TP.A8TT.01A.12R.A41O.07	-0.2508	0.16128	0.139091075	0.338943769	0.17252746	-0.000644742	0.107839102	-0.235979733	-0.065335944	0.243430854	0.009651258	-0.063685239	0.345698832	0.303492493	-0.048621378	-0.15993655	0.330630749	0.035718747	-0.015022409	-0.21009636	-0.161369256
TCGA.KK.A7AY.01A.11R.A33R.07	-0.3644	0.07514	0.124566389	0.24387603	0.151766714	0.000725509	-0.094760618	-0.215961643	-0.264512136	0.206644976	-0.133208643	-0.13262566	0.373120885	0.193520125	-0.121596817	-0.246866753	0.343756699	-0.058411217	-0.091804716	-0.223064616	-0.210285111
TCGA.HC.7213.01A.11R.2118.07	-0.3407	-0.0148	0.105031216	0.303914988	0.107054509	-0.031570978	-0.029900364	-0.227056633	-0.126337833	0.219850557	-0.026639205	-0.044851154	0.320470722	0.193219854	-0.065259343	-0.197557267	0.313639112	-0.014777066	-0.08550328	-0.216891636	-0.152091462
TCGA.EJ.5517.01A.01R.1580.07	-0.2679	0.1083	0.08609666	0.341525646	0.195187204	-0.00308694	-0.039773758	-0.15532383	-0.067419754	0.217912252	-0.015338009	-0.097786215	0.323770766	0.211042594	-0.066327467	-0.25513936	0.315361604	0.018599254	-0.045540847	-0.174048112	-0.161235827
TCGA.CH.5751.01A.11R.1580.07	0.10641	0.28737	0.097303399	0.406897649	0.223656678	0.024447929	0.138205951	-0.234375554	0.098194452	0.200709562	-0.170477934	0.001725914	0.306456425	0.212128227	-0.024812213	-0.384186582	0.338487221	0.094059971	0.016768541	-0.238551637	-0.127370802
TCGA.HC.7820.01A.11R.2118.07	-0.348	0.02158	0.143661486	0.26916871	0.111967713	-0.056154328	-0.054229203	-0.394634471	-0.265624057	0.229630044	-0.262188049	-0.131795891	0.36605262	0.185910708	-0.14774484	-0.160477909	0.307459163	-0.066958432	-0.101495439	-0.158623028	-0.237243094
TCGA.J4.A6G1.01A.11R.A311.07	0.234	0.29624	0.175653505	0.41966939	0.173530665	0.013846888	0.154725005	-0.182368252	0.163538023	0.233094845	-0.016410219	0.010203686	0.364969022	0.30864673	-0.045214949	-0.246846646	0.372019069	0.10249067	0.084451424	-0.181803884	-0.134520989
TCGA.EJ.5503.01A.01R.1580.07	-0.2337	0.01122	0.171059607	0.34686724	0.148899477	-0.053773324	-0.017128945	-0.243807539	-0.205497948	0.232195711	0.000103722	-0.079039554	0.381281764	0.304068478	-0.101492941	-0.19161465	0.322737877	-0.002068187	-0.063628504	-0.145368957	-0.162551854
TCGA.YL.A8SA.01A.21R.A37L.07	-0.4394	-0.0251	0.091556682	0.25512735	0.114711766	-0.031438708	-0.116097428	-0.133277245	-0.206106286	0.18082962	-0.225836142	-0.142225102	0.405436845	0.179298913	-0.14968248	-0.115560516	0.331852959	-0.070459234	-0.100981536	-0.229079859	-0.20416159
TCGA.TP.A8TV.01A.11R.A41O.07	-0.4987	-0.0154	0.094435362	0.278102645	0.110528577	-0.050531668	-0.117174197	-0.259882875	-0.285361517	0.160058495	-0.168544283	-0.14776852	0.379254376	0.146199964	-0.161640295	-0.217162533	0.285814865	-0.073404489	-0.117356624	-0.189590658	-0.15888621
TCGA.ZG.A9LM.01A.11R.A41O.07	-0.4506	0.0506	0.10134862	0.345986545	0.184995254	-0.020549904	-0.003943108	-0.259493078	-0.136077778	0.212402196	-0.161357962	-0.116987634	0.336507572	0.214801542	-0.034724263	-0.255233821	0.307157579	0.011036848	-0.067433228	-0.130411414	-0.18031427
TCGA.J4.A6M7.01A.11R.A31N.07	-0.4371	-0.0325	0.105921987	0.279524074	0.113221375	-0.036808777	-0.093721611	-0.327015457	-0.288256919	0.202832967	-0.114134406	-0.108438243	0.387896949	0.170868021	-0.102167822	-0.184033559	0.296006038	-0.039819028	-0.097177606	-0.257237349	-0.209988847
TCGA.ZG.A9M4.01A.11R.A41O.07	-0.2203	0.21053	0.116869569	0.372200103	0.16469125	-0.061751793	0.026572745	-0.235456919	-0.151288468	0.176708564	-0.146557634	-0.106449103	0.344691202	0.242660928	-0.04682219	-0.340561537	0.373522601	0.053620696	-0.020369618	-0.227649019	-0.159993224
TCGA.CH.5745.01A.11R.1580.07	-0.3621	0.08749	0.081836377	0.333631525	0.175511335	-0.058765814	0.013242958	-0.132615497	-0.144838248	0.20373147	0.033667419	-0.037491055	0.351725079	0.266255858	-0.092235821	-0.192878799	0.329395398	0.036414936	-0.027497901	-0.121231697	-0.168292705
TCGA.KK.A8IG.01A.11R.A36G.07	-0.2892	0.03215	0.110320239	0.338134791	0.185680883	-0.031168513	-0.0272889	-0.106123408	-0.113276277	0.215727246	-0.041283147	-0.164198051	0.350755597	0.255897089	0.011085857	-0.28006894	0.325655425	0.070455809	-0.024233834	-0.170590446	-0.159671229
TCGA.HC.7821.01A.12R.2118.07	-0.3643	0.05127	0.116580961	0.294887291	0.172941656	-0.000866474	0.079693114	-0.241663826	-0.096950706	0.214675368	-0.143843342	-0.087028527	0.364111491	0.239274866	-0.023398165	-0.17783192	0.303292376	0.016585791	-0.052501173	-0.227029377	-0.220425244
TCGA.Y6.A8TL.01A.21R.A37L.07	-0.3068	0.02288	0.115540635	0.319261963	0.123180733	-0.060024439	-0.085587675	-0.296797291	-0.242955148	0.205744878	-0.12713246	-0.144891332	0.352496109	0.223019237	-0.119164645	-0.189749116	0.281990529	-0.056202369	-0.075162411	-0.156158058	-0.151139917
TCGA.KK.A7AZ.01A.12R.A32O.07	-0.4432	0.03993	0.139808194	0.283079731	0.113796192	-0.055307845	-0.110306583	-0.269344382	-0.169726941	0.164520696	-0.199954905	-0.106953319	0.368915064	0.141311277	-0.135848702	-0.263240449	0.289043603	-0.067920568	-0.151183659	-0.16809604	-0.220414576
TCGA.HC.8256.01A.11R.2263.07	-0.3875	0.05254	0.103387655	0.279611108	0.112235365	-0.022970402	-0.098113754	-0.383745305	-0.265267797	0.220459032	-0.201122199	-0.161436414	0.353624868	0.148423553	-0.160459463	-0.214629896	0.286527771	-0.05239929	-0.133568839	-0.213792143	-0.137900735
TCGA.HC.7738.01A.11R.2118.07	-0.3253	-0.002	0.107108259	0.296627697	0.138877357	-0.060573574	-0.126109174	-0.36242654	-0.086083542	0.227913875	-0.248928171	-0.127234427	0.358419595	0.191298921	-0.10710988	-0.279772306	0.296475547	-0.030806371	-0.117795861	-0.182265458	-0.16306349
TCGA.HC.8265.01A.11R.2263.07	-0.3612	0.04427	0.135716551	0.320469111	0.144349301	-0.047472156	-0.016991564	-0.324332298	-0.186026361	0.197322537	-0.133968469	-0.117051256	0.35869733	0.255585053	-0.072549415	-0.379695135	0.324755264	-0.017748007	-0.065064725	-0.197636954	-0.246821242
TCGA.HI.7171.01A.12R.2118.07	-0.4028	0.03172	0.052178534	0.29597813	0.126347328	-0.041243377	-0.041150443	-0.317694058	-0.262995716	0.215767623	-0.166791025	-0.085048953	0.365412937	0.191242241	-0.104372011	-0.226041253	0.278150346	-0.091859919	-0.129396575	-0.176128434	-0.140944352
TCGA.CH.5794.01A.11R.1580.07	-0.4348	-0.0078	0.113706468	0.294387279	0.130747634	-0.070987412	-0.074097766	-0.109848828	-0.141954647	0.197326989	-0.200416367	-0.07156985	0.402386872	0.211380407	-0.12614106	-0.147260509	0.304227203	-0.027870369	-0.113471927	-0.216180732	-0.141037679
TCGA.KK.A7B1.01A.11R.A32O.07	-0.3954	-0.0128	0.168898054	0.32378711	0.122655102	-0.042304487	0.009667612	-0.226909551	-0.239097915	0.206694493	-0.073884538	-0.078528392	0.372983986	0.244921919	-0.055830501	-0.120432445	0.337103506	0.006313133	-0.072858014	-0.150753511	-0.119611452
TCGA.HC.A6HY.01A.11R.A31N.07	-0.3301	0.06826	0.146619028	0.254495221	0.111626518	-0.045044191	-0.081769399	-0.40019737	-0.296630652	0.159101142	-0.182690975	-0.11204875	0.370683065	0.174446532	-0.150854329	-0.18511784	0.303885484	-0.043883205	-0.115463593	-0.176970693	-0.239419992
TCGA.V1.A9O7.01A.21R.A41O.07	-0.2962	0.041	0.19547806	0.35694437	0.189368702	0.004202402	0.008428363	-0.066288781	-0.155039535	0.218155737	0.081737317	-0.089624445	0.314571632	0.302708621	0.002199117	-0.289075826	0.357241453	0.06849743	0.007414959	-0.190895656	-0.120950244
TCGA.XJ.A83H.01A.11R.A352.07	-0.3775	0.01047	0.160099206	0.301486241	0.166776911	-0.05905933	-0.064199521	-0.088789841	-0.226906336	0.198767471	-0.037507957	-0.130514683	0.372141783	0.216344657	-0.075100742	-0.183790627	0.303746632	0.009541716	-0.060089844	-0.146865974	-0.154077641
TCGA.HC.8265.01B.04R.2302.07	-0.258	0.08079	0.031112777	0.298371242	0.18678091	0.09566797	0.145094842	-0.080052701	0.053677623	0.161017091	-0.062046752	0.026629391	0.309789282	0.312662841	-0.09721718	-0.263219248	0.265674895	0.078388688	0.028188926	-0.173181712	-0.077426686
TCGA.HC.A8D1.01A.11R.A36G.07	-0.3306	0.06143	0.146185637	0.342524953	0.140809616	-0.045045236	0.047930925	-0.151517544	-0.177189342	0.254319661	-0.048187358	-0.050240247	0.358174579	0.265423609	-0.101522447	-0.252780829	0.331702896	0.004738566	-0.030710451	-0.131282973	-0.144382213
TCGA.YL.A8SO.01B.31R.A37L.07	-0.3862	-0.0058	0.163573223	0.32504614	0.247520505	-0.059232833	-0.043185117	-0.297571158	-0.107667359	0.166023143	-0.194788336	-0.074958756	0.346115086	0.196549733	-0.067087518	-0.19737824	0.326287454	-0.01738484	-0.09104395	-0.171333752	-0.19178611
TCGA.CH.5761.01A.11R.1580.07	-0.4301	-0.037	0.161632954	0.315914961	0.160320805	-0.041084998	-0.111214604	-0.249145764	-0.209095007	0.175890695	-0.135602134	-0.018243701	0.33232348	0.237309662	-0.083792122	-0.405847057	0.326159791	0.019064228	-0.129145661	-0.143477353	-0.126634819
TCGA.KK.A59V.01A.11R.A29R.07	0.01395	0.41854	0.133459353	0.426285375	0.174876571	0.046138125	0.193158044	-0.105579863	0.003588716	0.204278818	-0.089050307	-0.007873766	0.338326986	0.234582909	-0.02843893	-0.326599099	0.334217096	0.071856391	0.068201908	-0.158796901	-0.060228604
TCGA.V1.A9OY.01A.11R.A41O.07	-0.2976	0.08713	0.151826069	0.329546546	0.13852448	-0.05069817	-0.008344066	-0.392950093	-0.213949839	0.204228481	-0.14753797	-0.083669962	0.371602104	0.236831929	-0.08229766	-0.135284434	0.328738096	-0.009457437	-0.053083795	-0.200361586	-0.211017719
TCGA.OU.A6IP.01A.11R.A31N.07	-0.2785	0.01927	0.152827977	0.310050798	0.115009969	-0.076445423	-0.037923748	-0.277582179	-0.256951137	0.20112203	-0.123034012	-0.141836824	0.352225794	0.218800802	-0.119902421	-0.203011292	0.334852758	-0.037009757	-0.086620495	-0.132474964	-0.211114962
TCGA.HC.7077.01A.11R.1965.07	-0.3882	-0.0088	0.167682628	0.24221801	0.119285595	-0.061659015	-0.133163841	-0.300239447	-0.295153848	0.204403509	-0.233230902	-0.096591661	0.392270262	0.156569525	-0.176777618	-0.196681117	0.300652132	-0.060740515	-0.145450057	-0.18190998	-0.231829281
TCGA.EJ.7123.01A.11R.1965.07	-0.337	-0.0102	0.113790403	0.282897204	0.14188467	-0.055475168	-0.054572155	-0.392245457	-0.214391918	0.209392335	-0.176163382	-0.127176081	0.38617272	0.185506022	-0.127170547	-0.180552771	0.295469412	-0.016034907	-0.103219027	-0.155281801	-0.151895806
TCGA.HC.A8CY.01A.11R.A36G.07	-0.3354	0.12559	0.057342417	0.279803708	0.118653581	-0.012637222	0.004493602	-0.216497866	-0.076658964	0.167360276	-0.137166285	-0.166173935	0.367609477	0.183181068	-0.106279405	-0.293609815	0.305684104	-0.009346048	-0.094138546	-0.19288335	-0.219912758
TCGA.G9.A9S4.01A.11R.A41O.07	-0.4151	0.0024	0.123591811	0.267419241	0.113736821	-0.023496017	-0.098916032	-0.242296374	-0.328426696	0.190524623	-0.238561789	-0.127442759	0.332277679	0.153586076	-0.137058613	-0.207959293	0.287632434	-0.045088167	-0.146353301	-0.219736444	-0.222782497
TCGA.VP.A87H.01A.11R.A352.07	-0.4142	-0.0086	0.127199044	0.30024886	0.182819636	-0.058682268	-0.070596243	-0.192966718	-0.140492452	0.159568985	-0.116803786	-0.080209674	0.354148354	0.197194376	-0.135557339	-0.287992466	0.273570465	-0.036812669	-0.109039295	-0.162925579	-0.13038282
TCGA.YL.A9WI.01A.11R.A37L.07	-0.4765	-0.0063	0.016548645	0.290548931	0.190524141	-0.000453199	-0.147661802	-0.443729362	-0.103270485	0.144222444	-0.228294477	-0.03963217	0.322439479	0.1832526	-0.112220379	-0.364499325	0.260246956	-0.086478698	-0.148399612	-0.203543944	-0.181933233
TCGA.VP.A87D.01A.11R.A352.07	-0.4731	-0.0346	0.12521584	0.251693217	0.11497565	-0.004856805	-0.074397979	-0.412142171	-0.26275854	0.192706357	-0.296561166	-0.101332935	0.333317474	0.16726332	-0.175336579	-0.283090674	0.311438386	-0.08917355	-0.15702803	-0.229703318	-0.212666459
TCGA.KK.A6E2.01A.11R.A311.07	-0.4095	0.01917	0.083281783	0.255558289	0.124669262	-0.013115607	-0.079291635	-0.244291965	-0.265005943	0.198505998	-0.195021403	-0.06050499	0.388270702	0.191858641	-0.134243263	-0.210663459	0.294321126	-0.0454757	-0.120550707	-0.275670599	-0.232726387
TCGA.YL.A8SH.01B.11R.A37L.07	-0.316	0.01917	0.150041542	0.318290968	0.117210082	-0.025420185	-0.053912353	-0.288021782	-0.25041965	0.173725518	-0.191235068	-0.111795549	0.367203782	0.224463207	-0.103783904	-0.155753985	0.318068753	-0.04427233	-0.106268024	-0.144586344	-0.177538664
TCGA.EJ.7318.01B.11R.A32O.07	-0.3044	0.0534	0.192541401	0.307559859	0.160982162	-0.064993939	-0.037215131	-0.397533144	-0.19340762	0.176535925	-0.155030377	-0.117126126	0.350846985	0.245590702	-0.103211186	-0.167014949	0.29028456	0.004111725	-0.07953286	-0.156495477	-0.211815302
TCGA.HC.7740.01B.04R.2302.07	-0.1874	-0.0851	0.091315115	0.335253211	0.225999627	0.048128659	0.177513351	0.021552401	0.101343729	0.208372532	0.118319088	0.075631568	0.230628932	0.37184755	-0.062973137	-0.128017886	0.325263571	0.101607739	0.037790244	-0.180378615	-0.025054196
TCGA.HC.7075.01A.11R.1965.07	-0.2542	0.05695	0.11294305	0.29073324	0.120708525	-0.035195961	-0.107224337	-0.222992335	-0.136134291	0.209771802	-0.156676321	-0.129629814	0.385463216	0.17166697	-0.111146036	-0.246091787	0.255998677	-0.046492225	-0.095605875	-0.155374078	-0.220748033
TCGA.HC.A6HX.01A.11R.A31N.07	-0.3102	0.06971	0.196288998	0.280986227	0.140639317	-0.078039631	0.006435471	-0.20660887	-0.254801581	0.193774633	-0.096706589	-0.119528548	0.374638598	0.236113649	-0.07051858	-0.287652048	0.330242631	0.012915008	-0.087335478	-0.070587309	-0.19737139
TCGA.V1.A9ZI.01A.11R.A41O.07	-0.3759	0.05334	0.133891036	0.323370535	0.171706258	-0.040176866	0.030312048	-0.417163123	-0.174587322	0.177997491	-0.156218646	-0.132461744	0.335248391	0.203570452	-0.018448083	-0.213794574	0.313558292	-0.011876844	-0.076044502	-0.187984291	-0.186787588
TCGA.HC.7211.01A.11R.2118.07	-0.2673	-0.0216	0.1083695	0.305248095	0.189936687	-0.032542083	0.014543788	0.038935028	-0.192182373	0.230378087	0.070314012	-0.075745634	0.319519145	0.221797566	-0.063695778	-0.256794054	0.322567806	0.015833525	-0.060923284	-0.159534072	-0.152691523
TCGA.HC.7232.01A.11R.2118.07	-0.4571	-0.0179	0.121260718	0.313357707	0.143169697	0.009045162	-0.070997541	-0.314349635	-0.200667355	0.200194229	-0.216169105	-0.109797277	0.374422301	0.23213347	-0.107875595	-0.170294894	0.318670257	-0.009698027	-0.080635101	-0.205899584	-0.162334222
TCGA.EJ.A7NJ.01A.22R.A352.07	-0.2687	0.06377	0.124394404	0.33683945	0.117628567	-0.054658139	-0.048225985	-0.32852649	-0.16382337	0.271208768	-0.148139759	-0.163904382	0.375130633	0.238515589	-0.147288366	-0.338612557	0.319839713	-0.041757669	-0.119597989	-0.135101821	-0.160994963
TCGA.X4.A8KO.01A.12R.A36G.07	-0.3118	0.01415	0.137244988	0.339037496	0.161763247	-0.027546617	0.020944991	-0.150009306	-0.215737096	0.204037761	-0.106081454	-0.104994981	0.376113326	0.22240357	-0.001119844	-0.182787656	0.344376192	0.063273561	-0.0195164	-0.127154985	-0.198400033
TCGA.HC.7210.01A.11R.2118.07	-0.219	0.02669	0.146911989	0.32062776	0.177892743	-0.035040265	0.030558658	-0.003259733	-0.112132143	0.202601801	-0.007304584	-0.05334625	0.390406766	0.273994341	-0.057440077	-0.200493135	0.336892934	0.06320575	-0.023597327	-0.192655324	-0.093357357
TCGA.HC.A6AO.01A.11R.A30B.07	-0.2468	0.09615	0.162266547	0.284386715	0.119275126	-0.052546515	-0.079137889	-0.316205282	-0.200461813	0.205734957	-0.153048776	-0.118705419	0.381122863	0.168025948	-0.114511673	-0.168991071	0.314812858	-0.041654691	-0.102561374	-0.133951273	-0.241901595
TCGA.J4.A67T.01A.11R.A311.07	-0.3388	0.00526	0.121148995	0.345490668	0.148691619	-0.083715643	-0.015814601	-0.218608937	-0.251320185	0.20748355	-0.04628415	-0.096180422	0.368290263	0.216750654	-0.034139536	-0.155596955	0.341819535	-0.000981117	-0.019771321	-0.156082137	-0.127934676
TCGA.CH.5739.01A.11R.1580.07	-0.3314	0.04495	0.066650343	0.301322687	0.172433471	-0.020957314	0.042843794	-0.134300703	-0.146282394	0.233907393	-0.067546562	-0.04512618	0.355785881	0.198648927	-0.07516526	-0.191241948	0.318514605	0.043980431	-0.040030437	-0.189230664	-0.144072628
TCGA.ZG.A9L1.01A.11R.A41O.07	-0.3696	-0.0515	0.044718033	0.281021434	0.097330986	0.017605747	-0.156452285	-0.147840943	-0.272016803	0.132725632	-0.11242294	-0.097493089	0.328357264	0.208821308	-0.081342817	-0.119920457	0.272484214	-0.092379536	-0.069926153	-0.233445337	-0.13083674
TCGA.EJ.7325.01B.11R.A32O.07	-0.3536	0.04638	0.126206034	0.260769411	0.127516739	-0.015836281	-0.083567166	-0.218495428	-0.197132763	0.166054768	-0.172404194	-0.083981209	0.364589952	0.146115073	-0.094873096	-0.217845123	0.304406208	-0.019146852	-0.105732615	-0.140010334	-0.258407601
TCGA.KK.A5A1.01A.11R.A29R.07	-0.3931	0.05249	0.060105108	0.29678313	0.204708437	-0.002887124	-0.03355937	-0.313599486	-0.117031476	0.166091589	-0.142500024	-0.041388377	0.331428624	0.22640051	-0.083183865	-0.346978451	0.260763134	0.013300587	-0.085335267	-0.205276978	-0.206161628
TCGA.EJ.5504.01A.01R.1580.07	-0.3297	0.00445	0.100312466	0.335417104	0.176422118	-0.0490174	-0.058236246	-0.25099042	-0.123381114	0.209040177	-0.095928105	-0.066916327	0.393677427	0.242331611	-0.07139706	-0.285607796	0.314719442	0.018722268	-0.07115732	-0.206876265	-0.150959136
TCGA.EJ.7314.01A.31R.2118.07	-0.3581	-0.0114	0.135784614	0.305082363	0.162249972	-0.009774113	-0.02092876	-0.148577834	-0.261882877	0.204174091	-0.045011561	-0.052885071	0.374169087	0.184123117	-0.094770792	-0.154498481	0.271194908	-0.028227893	-0.074049075	-0.215639385	-0.161768499
TCGA.J4.A83I.01A.11R.A36G.07	-0.3309	0.01692	0.085859728	0.289340893	0.125885293	-0.009391415	0.019148329	-0.162177848	-0.231781627	0.254282775	-0.113749797	-0.083274073	0.34183458	0.216411021	-0.131881411	-0.228120786	0.328262353	-0.082764916	-0.094038208	-0.229839362	-0.202847253
TCGA.EJ.A6RA.01A.11R.A33R.07	-0.3052	0.06559	0.164900333	0.314701874	0.147997549	-0.063055399	-0.044303789	-0.398106448	-0.239777389	0.215286365	-0.138202337	-0.120008224	0.361953611	0.215028342	-0.117124342	-0.193169337	0.334271183	-0.014702508	-0.096193564	-0.152182358	-0.198038665
TCGA.HC.8257.01A.11R.2263.07	-0.3485	0.04694	0.083426176	0.310824627	0.138045697	0.024101379	-0.07031742	-0.31840145	-0.211388936	0.226040228	-0.188152855	-0.121947937	0.388475187	0.186308271	-0.139298254	-0.197977319	0.295411162	-0.0426001	-0.102634094	-0.257251923	-0.222105191

TCGA.VP.A87C.01A.11R.A352.07	0.09858	0.34246	0.100188284	0.411567447	0.187632431	-0.000972823	0.196364735	-0.091297571	0.089984188	0.220929402	0.002206217	0.012350095	0.384671594	0.352684364	0.006313824	-0.267131949	0.367486206	0.120911973	0.082576315	-0.154325787	-0.135385131
TCGA.CH.5744.01A.11R.1580.07	-0.3442	0.00239	0.142254911	0.300243939	0.146676578	-0.072714145	-0.039297182	-0.009787685	-0.186871885	0.211496905	0.003723776	-0.113480062	0.397559997	0.20295267	-0.097119107	-0.104454672	0.327667574	0.034617812	-0.069408481	-0.146930789	-0.140173546
TCGA.V1.A8WV.01A.11R.A37L.07	-0.3177	0.10073	0.120546701	0.346113749	0.20358849	0.012086775	0.007348407	-0.409067783	-0.140590986	0.160211467	-0.161546478	-0.042280661	0.351815256	0.234198122	-0.054605534	-0.370240391	0.342694923	-0.000822594	-0.057592377	-0.075318059	-0.091537963
TCGA.VP.A872.01A.11R.A352.07	-0.214	0.04804	0.115605908	0.360232015	0.153617985	-0.059677271	-0.0068089	-0.190921652	-0.208571996	0.211350302	-0.141719685	-0.060072384	0.377554367	0.246425911	-0.039675024	-0.212699492	0.308316406	0.037623797	-0.025179722	-0.154873892	-0.207538368
TCGA.J4.AATV.01A.11R.A41O.07	-0.3056	0.00507	0.158993636	0.350206955	0.152680055	-0.068879824	0.036025357	-0.121179014	-0.177889815	0.246775867	0.012016779	-0.020003103	0.352905381	0.301711963	-0.028654431	-0.134992894	0.373655352	0.070454658	0.007714169	-0.105674834	-0.116085908
TCGA.EJ.A65D.01A.11R.A30B.07	-0.4513	0.01308	0.124292915	0.234713213	0.091290357	-0.041360917	-0.151893951	-0.386868763	-0.352507148	0.191884348	-0.294821806	-0.040817409	0.317735557	0.082188504	-0.1637198	-0.185141303	0.283946586	-0.103497588	-0.175994619	-0.179468349	-0.247074936
TCGA.EJ.5519.01A.01R.1580.07	-0.3912	-0.0399	0.160961782	0.333773491	0.119030149	-0.054474877	-0.061310024	-0.318899984	-0.238737428	0.194966086	-0.177198663	-0.060271614	0.401089404	0.219555821	-0.114874448	-0.129533573	0.335726291	-0.010619499	-0.100163672	-0.26575638	-0.208080948
TCGA.EJ.A46B.01A.31R.A250.07	-0.2866	-0.041	0.148435352	0.318303269	0.106099468	-0.078238652	-0.09356101	-0.39413301	-0.227164351	0.268840829	-0.116499628	-0.132591697	0.382676939	0.221503542	-0.121570878	-0.243828511	0.293778941	-0.045675688	-0.103234633	-0.155146911	-0.202183125
TCGA.QU.A6IN.01A.11R.A31N.07	-0.3605	0.0344	0.111717197	0.234915399	0.10375474	-0.062499534	-0.109582595	-0.176883169	-0.269661248	0.170663902	-0.179612922	-0.147685236	0.359041742	0.098379569	-0.149253274	-0.283117536	0.320446777	-0.089549715	-0.127408256	-0.054679712	-0.307533765
TCGA.KK.A59Z.01A.12R.A26U.07	-0.4589	-0.0069	0.090190236	0.339592044	0.213329279	-0.065177013	-0.006563162	-0.010886656	-0.15389232	0.189122283	-0.013811083	-0.084870292	0.373459591	0.245859642	-0.011509711	-0.206271264	0.325217239	0.049493323	-0.03535428	-0.189120932	-0.168293428
TCGA.XK.AAJT.01A.11R.A41O.07	-0.2813	0.01824	0.173166855	0.333547794	0.179159735	-0.061935302	0.016830021	-0.297856703	-0.198787727	0.225218391	-0.059736998	-0.088658197	0.352428385	0.268918935	-0.031264381	-0.278036725	0.342163842	0.018298194	-0.031133242	-0.193812674	-0.160260096
TCGA.EJ.7125.01A.11R.1965.07	-0.2879	0.02901	0.094920982	0.314683592	0.145707621	-0.016018811	-0.079773175	-0.136345407	-0.154044642	0.22640537	0.013308507	-0.145118689	0.349397737	0.208678847	-0.088649749	-0.31477983	0.278074346	0.044779502	-0.049257608	-0.218320224	-0.104976364
TCGA.J4.A67Q.01A.21R.A30B.07	-0.1871	0.10617	0.169398731	0.331355692	0.164163993	-0.052640415	0.047904486	-0.237690898	-0.177849729	0.193840048	0.01957836	-0.110051272	0.349660082	0.238020054	-0.036708982	-0.232699115	0.346474986	0.027737709	-0.030272531	-0.110830296	-0.174982371
TCGA.G9.6361.01A.21R.1965.07	-0.3986	0.00921	0.085256722	0.314180098	0.141996248	-0.048405834	-0.030668405	-0.344931045	-0.195709882	0.215297941	-0.234906773	-0.075346669	0.328832236	0.245893054	-0.078571253	-0.174031939	0.349327743	-0.052077852	-0.06662908	-0.150251643	-0.171833143
TCGA.VN.A88L.01A.11R.A352.07	-0.3865	-0.0261	0.1510395	0.302864414	0.117100847	-0.032585956	-0.009290806	-0.164278609	-0.195230333	0.194469354	0.053460074	-0.101276166	0.357432732	0.231830011	-0.063722781	-0.196311729	0.332247093	-0.004515584	-0.032638624	-0.221418214	-0.156667093
TCGA.KK.A8I9.01A.11R.A36G.07	-0.2872	0.04195	0.17409024	0.362100098	0.189247003	-0.050271127	0.073202638	-0.261309043	-0.208044719	0.178685496	0.014616352	-0.041516767	0.370992453	0.251960544	0.008923897	-0.183376351	0.375800172	0.072971479	-0.001442895	-0.158459725	-0.100952179
TCGA.V1.A9ZG.01A.11R.A41O.07	-0.4425	-0.0287	0.163677377	0.323842808	0.164492923	-0.00068659	-0.008075071	-0.157106508	-0.240841246	0.233273153	-0.066644737	-0.12136	0.347696216	0.222951275	-0.053204037	-0.245832006	0.329852712	0.02049528	-0.036851254	-0.263434224	-0.188884841
TCGA.V1.A9OX.01A.11R.A41O.07	-0.324	0.07997	0.177214832	0.331588685	0.138658431	-0.037885134	-0.053522635	-0.287267992	-0.187420197	0.214413731	-0.085876959	-0.148827488	0.379968919	0.242094094	-0.086420129	-0.243112882	0.307831786	-0.027979126	-0.072487017	-0.147016877	-0.185027565
TCGA.YL.A8SI.01A.11R.A41O.07	-0.2911	0.00683	0.135302511	0.325471114	0.170333343	-0.031651442	-0.00995563	-0.200657936	-0.199046962	0.230021892	-0.031210531	-0.124922422	0.34313044	0.252543097	-0.04993835	-0.207219966	0.338248695	0.035360797	-0.035281894	-0.161566602	-0.128215551
TCGA.G9.6499.01A.12R.1965.07	-0.3248	0.04334	0.121952788	0.301812211	0.144554318	-0.068377805	-0.05773058	-0.206020621	-0.240361181	0.207564714	-0.079795466	-0.112965952	0.391289271	0.187265692	-0.061713285	-0.18741826	0.275318278	-0.002002082	-0.05677159	-0.145290004	-0.120369325
TCGA.QU.A6IL.01A.11R.A31N.07	-0.2582	0.10148	0.167133122	0.240016938	0.119652393	-0.07387644	-0.072277973	-0.162913311	-0.23346573	0.14928747	-0.112346203	-0.174990024	0.385545278	0.162333496	-0.056229573	-0.283284553	0.327858445	-0.027429005	-0.066000551	-0.080003105	-0.201220533
TCGA.CH.5764.01A.21R.1580.07	-0.3444	0.06634	0.093234892	0.304757432	0.146557967	0.000819543	-0.01583546	-0.125040087	-0.141972493	0.191351098	-0.072206681	-0.040820005	0.367089939	0.246649283	-0.103844718	-0.206448551	0.321920845	-0.013668269	-0.073394824	-0.203769888	-0.148023077
TCGA.CH.5789.01A.11R.1580.07	-0.3021	-0.0305	0.143608521	0.354840695	0.181094264	-0.044669229	-0.016327488	-0.047062285	-0.141940439	0.265874965	0.082274966	-0.034596967	0.364639188	0.257045071	-0.073853794	-0.175155281	0.346028506	0.058147673	-0.015707882	-0.133560531	-0.10457137
TCGA.FC.A66V.01A.21R.A30B.07	-0.2829	0.04471	0.176492724	0.32630425	0.15855038	-0.05452172	-0.024721707	-0.295556965	-0.21758283	0.230850709	-0.117949621	-0.170461584	0.36050446	0.228414504	-0.084170662	-0.31347722	0.285916441	0.005331707	-0.112935653	-0.095473298	-0.235517928
TCGA.EJ.7330.01A.11R.2118.07	-0.3424	-0.0112	0.114243372	0.333183719	0.172040321	-0.042159871	-0.035073468	-0.177093003	-0.103911042	0.215008652	0.020478838	-0.073908986	0.368439847	0.2890529	-0.069247089	-0.23754217	0.349391001	0.022838759	-0.061948209	-0.193824021	-0.093123522
TCGA.HC.8258.01B.05R.2302.07	-0.0797	0.0912	0.043925632	0.357761115	0.219412897	0.090898727	0.291990403	0.082533625	0.191115345	0.233713307	0.206910043	0.104207894	0.266137496	0.385723528	0.025344617	-0.105321068	0.30160729	0.137361869	0.099949574	-0.104303132	0.055664637
TCGA.YL.A8SL.01B.21R.A37L.07	-0.4457	-0.0172	0.122179795	0.221080647	0.105647425	-0.042214749	-0.136714613	-0.308674434	-0.32545608	0.179105815	-0.301132148	-0.092820242	0.305832218	0.141329119	-0.132715091	-0.177543656	0.304676652	-0.098467693	-0.168928116	-0.184238834	-0.18239652
TCGA.XK.AAIW.01A.11R.A41O.07	-0.3832	0.18423	0.090369383	0.308222676	0.142624823	0.038221441	-0.090190746	-0.394851094	-0.220840715	0.19902111	-0.247387335	-0.090252497	0.339917481	0.125257981	-0.106338462	-0.273748918	0.311223961	-0.064326015	-0.11692277	-0.300040775	-0.093117756
TCGA.G9.6385.01A.11R.1789.07	-0.3106	-0.0165	0.139538415	0.331591635	0.156293976	-0.058334833	-0.072209251	-0.260461123	-0.210508901	0.206020495	-0.060410488	-0.137261932	0.38455225	0.249093068	-0.096348519	-0.272320623	0.316645823	-0.026902137	-0.088692163	-0.134578916	-0.163195689
TCGA.HC.A76X.01A.11R.A33R.07	-0.4896	-0.0055	0.089093163	0.261575978	0.110226338	-0.024339705	-0.125096327	-0.285002437	-0.307199491	0.212590814	-0.153513416	-0.104110808	0.379208189	0.134544097	-0.138268831	-0.175528785	0.27321052	-0.080977126	-0.130503463	-0.247934858	-0.181577575
TCGA.EJ.A46I.01A.12R.A26U.07	-0.2657	-0.0242	0.153411721	0.332477629	0.135194379	-0.068842711	-0.077914291	-0.304684509	-0.237701924	0.24250558	-0.153373423	-0.079582269	0.377249593	0.271455317	-0.133298164	-0.246840858	0.343534564	-0.04520664	-0.083833099	-0.150342348	-0.192118277
pvalue	1.38E-09	0.00017	0.000119314	7.15E-12	1.22E-08	1.63E-05	0.018544874	0.028829056	0.003795153	1.02E-26	3.44E-09	6.58E-06	0.002297795	4.30E-15	7.99E-05	2.87E-06	1.52E-07	0.000176607	1.11E-08	9.63E-06	4.23E-20
