# Supplementary material for: Integrative Analysis of Bulk RNA-Seq and Single-Cell RNA-Seq Unveils the Characteristics of the Immune Microenvironment and Prognosis Signature in Prostate Cancer
Source: J Oncol. 2022 Jul 19;2022:6768139. doi: 10.1155/2022/6768139 (PMC9325591; doi:10.1155/2022/6768139)
Supplement: Supplementary Materials — Figure S1. Workflow of the analysis. Figure S2. Validation of the risk score model using the GSE54460 dataset. A. Patients with prostate cancer (PRAD) in the GSE54460 cohort are listed in ascending order of risk score. B. Progression-free interval (PFI) distribution versus the risk score of each patient in the GSE54460 cohort. C. Kaplan–Meier (KM) curves of patients with different risk levels in the GSE54460 validation set. D. Receiver Operating Characteristic (ROC) curve analysis for 1-, 3- and 5-year PFI using the clinical information of patients of the GSE54460 validation dataset. Figure S3. Validation of the risk score model using the GSE46602 dataset. A. Patients with prostate cancer (PRAD) in the GSE46602 cohort are listed in ascending order of risk score. B. Progression-free interval (PFI) distribution versus the risk score of each patient in the GSE46602 cohort. C. Kaplan–Meier (KM) curves of patients with different risk levels in the GSE46602 validation dataset. D. Receiver Operating Characteristic (ROC) curve analysis for 1-, 3- and 5-year PFI using the clinical information of patients of the GSE46602 validation dataset. Figure S4. Validation of the risk score model using the GSE70768 dataset. A. Patients with prostate cancer (PRAD) in the GSE70768 cohort are listed in ascending order of risk score. B. Progression-free interval (PFI) distribution versus the risk score of each patient in the GSE70768 cohort. C. Kaplan–Meier (KM) curves of patients with different risk levels in the GSE70768 validation dataset. D. Receiver Operating Characteristic (ROC) curve analysis for 1-, 3- and 5-year PFI using the clinical information of patients of the GSE70768 validation dataset. Figure S5. Validation of the risk score model using the GSE70769 dataset. A. Patients with prostate cancer (PRAD) in the GSE70769 validation dataset are listed in ascending order of risk score. B. Progression-free interval (PFI) distribution versus the risk score of each patient in the GSE707 [file 6768139.f1.zip › 6768139.f1/Table S5.pdf]

Immune_Cell	indivs	mean	sd	n	se
Activated B cell	Normal	-0.25899	0.078035	52	0.010821
Activated B cell	Tumor	-0.3297	0.102065	498	0.004574
Activated CD8 T cell	Normal	0.011024	0.05641	52	0.007823
Activated CD8 T cell	Tumor	0.043479	0.070571	498	0.003162
CD56dim natural killer cell	Normal	0.150853	0.034648	52	0.004805
CD56dim natural killer cell	Tumor	0.128496	0.037161	498	0.001665
Central memory CD4 T cell	Normal	0.35201	0.027532	52	0.003818
Central memory CD4 T cell	Tumor	0.314824	0.038739	498	0.001736
Central memory CD8 T cell	Normal	0.182236	0.034302	52	0.004757
Central memory CD8 T cell	Tumor	0.152389	0.030116	498	0.00135
Effector memeory CD4 T cell	Normal	-0.01067	0.037059	52	0.005139
Effector memeory CD4 T cell	Tumor	-0.03248	0.029899	498	0.00134
Effector memeory CD8 T cell	Normal	-0.00434	0.060819	52	0.008434
Effector memeory CD8 T cell	Tumor	-0.02648	0.071647	498	0.003211
Eosinophil	Normal	-0.22184	0.094698	52	0.013132
Eosinophil	Tumor	-0.25184	0.09839	498	0.004409
Immature B cell	Normal	-0.1564	0.064988	52	0.009012
Immature B cell	Tumor	-0.18286	0.080239	498	0.003596
Immature dendritic cell	Normal	0.26968	0.023739	52	0.003292
Immature dendritic cell	Tumor	0.208226	0.028128	498	0.00126
Mast cell	Normal	-0.00601	0.096733	52	0.013414
Mast cell	Tumor	-0.09768	0.091256	498	0.004089
Memory B cell	Normal	-0.0554	0.052957	52	0.007344
Memory B cell	Tumor	-0.09047	0.054307	498	0.002434
Monocyte	Normal	0.349587	0.021988	52	0.003049
Monocyte	Tumor	0.358609	0.025546	498	0.001145
Natural killer cell	Normal	0.286993	0.039602	52	0.005492
Natural killer cell	Tumor	0.222296	0.056563	498	0.002535
Natural killer T cell	Normal	-0.05565	0.045114	52	0.006256
Natural killer T cell	Tumor	-0.08289	0.047353	498	0.002122
Neutrophil	Normal	-0.27285	0.070283	52	0.009747
Neutrophil	Tumor	-0.22549	0.064768	498	0.002902
Plasmacytoid dendritic cell	Normal	0.344958	0.031167	52	0.004322
Plasmacytoid dendritic cell	Tumor	0.319	0.029615	498	0.001327
T follicular helper cell	Normal	0.020664	0.045663	52	0.006332
T follicular helper cell	Tumor	-0.00516	0.045672	498	0.002047
Type 1 T helper cell	Normal	-0.02646	0.050569	52	0.007013
Type 1 T helper cell	Tumor	-0.07152	0.051214	498	0.002295
Type 17 T helper cell	Normal	-0.15162	0.04237	52	0.005876
Type 17 T helper cell	Tumor	-0.17937	0.041925	498	0.001879
Type 2 T helper cell	Normal	-0.09777	0.033662	52	0.004668
Type 2 T helper cell	Tumor	-0.1659	0.047332	498	0.002121
