# Supplementary material for: Integrative Analysis of Bulk RNA-Seq and Single-Cell RNA-Seq Unveils the Characteristics of the Immune Microenvironment and Prognosis Signature in Prostate Cancer
Source: J Oncol. 2022 Jul 19;2022:6768139. doi: 10.1155/2022/6768139 (PMC9325591; doi:10.1155/2022/6768139)
Supplement: Supplementary Materials — Figure S1. Workflow of the analysis. Figure S2. Validation of the risk score model using the GSE54460 dataset. A. Patients with prostate cancer (PRAD) in the GSE54460 cohort are listed in ascending order of risk score. B. Progression-free interval (PFI) distribution versus the risk score of each patient in the GSE54460 cohort. C. Kaplan–Meier (KM) curves of patients with different risk levels in the GSE54460 validation set. D. Receiver Operating Characteristic (ROC) curve analysis for 1-, 3- and 5-year PFI using the clinical information of patients of the GSE54460 validation dataset. Figure S3. Validation of the risk score model using the GSE46602 dataset. A. Patients with prostate cancer (PRAD) in the GSE46602 cohort are listed in ascending order of risk score. B. Progression-free interval (PFI) distribution versus the risk score of each patient in the GSE46602 cohort. C. Kaplan–Meier (KM) curves of patients with different risk levels in the GSE46602 validation dataset. D. Receiver Operating Characteristic (ROC) curve analysis for 1-, 3- and 5-year PFI using the clinical information of patients of the GSE46602 validation dataset. Figure S4. Validation of the risk score model using the GSE70768 dataset. A. Patients with prostate cancer (PRAD) in the GSE70768 cohort are listed in ascending order of risk score. B. Progression-free interval (PFI) distribution versus the risk score of each patient in the GSE70768 cohort. C. Kaplan–Meier (KM) curves of patients with different risk levels in the GSE70768 validation dataset. D. Receiver Operating Characteristic (ROC) curve analysis for 1-, 3- and 5-year PFI using the clinical information of patients of the GSE70768 validation dataset. Figure S5. Validation of the risk score model using the GSE70769 dataset. A. Patients with prostate cancer (PRAD) in the GSE70769 validation dataset are listed in ascending order of risk score. B. Progression-free interval (PFI) distribution versus the risk score of each patient in the GSE707 [file 6768139.f1.zip › 6768139.f1/Table S6.pdf]

ONTOLOGY	ID	Description	GeneRatio	p.adjust	Count
BP	GO:0051701	biological process involved in interaction with host	19/408	0.001369	19
BP	GO:0019883	antigen processing and presentation of endogenous antigen	7/408	0.001393	7
BP	GO:0019885	antigen processing and presentation of endogenous peptide antigen via MHC class I	6/408	0.001393	6
BP	GO:0052126	movement in host environment	16/408	0.001393	16
BP	GO:0042119	neutrophil activation	29/408	0.001393	29
BP	GO:0002483	antigen processing and presentation of endogenous peptide antigen	6/408	0.001521	6
BP	GO:0044409	entry into host	14/408	0.003799	14
BP	GO:0043312	neutrophil degranulation	27/408	0.004195	27
BP	GO:0002283	neutrophil activation involved in immune response	27/408	0.004195	27
BP	GO:0002474	antigen processing and presentation of peptide antigen via MHC class I	11/408	0.004195	11
BP	GO:0002446	neutrophil mediated immunity	27/408	0.005158	27
BP	GO:0019882	antigen processing and presentation	17/408	0.005198	17
BP	GO:0045785	positive regulation of cell adhesion	24/408	0.006647	24
BP	GO:0072521	purine-containing compound metabolic process	25/408	0.007649	25
BP	GO:0034341	response to interferon-gamma	15/408	0.007649	15
BP	GO:0009150	purine ribonucleotide metabolic process	23/408	0.008252	23
CC	GO:0012507	ER to Golgi transport vesicle membrane	10/421	0.000198	10
CC	GO:0030134	COPII-coated ER to Golgi transport vesicle	12/421	0.000198	12
CC	GO:0030176	integral component of endoplasmic reticulum membrane	14/421	0.001123	14
CC	GO:0042611	MHC protein complex	6/421	0.001123	6
CC	GO:0045177	apical part of cell	24/421	0.001123	24
CC	GO:0031227	intrinsic component of endoplasmic reticulum membrane	14/421	0.001123	14
CC	GO:0005911	cell-cell junction	26/421	0.001302	26
CC	GO:0030135	coated vesicle	19/421	0.001302	19
CC	GO:0016324	apical plasma membrane	21/421	0.001302	21
CC	GO:0071556	integral component of lumenal side of endoplasmic reticulum membrane	6/421	0.001302	6
CC	GO:0098553	lumenal side of endoplasmic reticulum membrane	6/421	0.001302	6
CC	GO:0030662	coated vesicle membrane	14/421	0.001681	14
CC	GO:0062023	collagen-containing extracellular matrix	23/421	0.001869	23
CC	GO:0031301	integral component of organelle membrane	21/421	0.002073	21
CC	GO:0031300	intrinsic component of organelle membrane	22/421	0.002139	22
CC	GO:0055038	recycling endosome membrane	9/421	0.00293	9
CC	GO:0098576	lumenal side of membrane	6/421	0.00293	6
CC	GO:0005925	focal adhesion	22/421	0.00293	22
CC	GO:0030055	cell-substrate junction	22/421	0.003524	22
CC	GO:0030670	phagocytic vesicle membrane	8/421	0.005941	8
