# Supplementary material for: Integrative Analysis of Bulk RNA-Seq and Single-Cell RNA-Seq Unveils the Characteristics of the Immune Microenvironment and Prognosis Signature in Prostate Cancer
Source: J Oncol. 2022 Jul 19;2022:6768139. doi: 10.1155/2022/6768139 (PMC9325591; doi:10.1155/2022/6768139)
Supplement: Supplementary Materials — Figure S1. Workflow of the analysis. Figure S2. Validation of the risk score model using the GSE54460 dataset. A. Patients with prostate cancer (PRAD) in the GSE54460 cohort are listed in ascending order of risk score. B. Progression-free interval (PFI) distribution versus the risk score of each patient in the GSE54460 cohort. C. Kaplan–Meier (KM) curves of patients with different risk levels in the GSE54460 validation set. D. Receiver Operating Characteristic (ROC) curve analysis for 1-, 3- and 5-year PFI using the clinical information of patients of the GSE54460 validation dataset. Figure S3. Validation of the risk score model using the GSE46602 dataset. A. Patients with prostate cancer (PRAD) in the GSE46602 cohort are listed in ascending order of risk score. B. Progression-free interval (PFI) distribution versus the risk score of each patient in the GSE46602 cohort. C. Kaplan–Meier (KM) curves of patients with different risk levels in the GSE46602 validation dataset. D. Receiver Operating Characteristic (ROC) curve analysis for 1-, 3- and 5-year PFI using the clinical information of patients of the GSE46602 validation dataset. Figure S4. Validation of the risk score model using the GSE70768 dataset. A. Patients with prostate cancer (PRAD) in the GSE70768 cohort are listed in ascending order of risk score. B. Progression-free interval (PFI) distribution versus the risk score of each patient in the GSE70768 cohort. C. Kaplan–Meier (KM) curves of patients with different risk levels in the GSE70768 validation dataset. D. Receiver Operating Characteristic (ROC) curve analysis for 1-, 3- and 5-year PFI using the clinical information of patients of the GSE70768 validation dataset. Figure S5. Validation of the risk score model using the GSE70769 dataset. A. Patients with prostate cancer (PRAD) in the GSE70769 validation dataset are listed in ascending order of risk score. B. Progression-free interval (PFI) distribution versus the risk score of each patient in the GSE707 [file 6768139.f1.zip › 6768139.f1/Table S7.pdf]

ONTOLOGY	ID	Description	GeneRatio	p.adjust	Count
BP	GO:0007565	female pregnancy	8/108	0.009861	8
BP	GO:0036499	PERK-mediated unfolded protein response	4/108	0.009861	4
CC	GO:0016324	apical plasma membrane	11/111	0.001035	11
CC	GO:0045177	apical part of cell	11/111	0.001964	11
CC	GO:0062023	collagen-containing extracellular matrix	11/111	0.001964	11
