# Supplementary material for: Integrative Analysis of Bulk RNA-Seq and Single-Cell RNA-Seq Unveils the Characteristics of the Immune Microenvironment and Prognosis Signature in Prostate Cancer
Source: J Oncol. 2022 Jul 19;2022:6768139. doi: 10.1155/2022/6768139 (PMC9325591; doi:10.1155/2022/6768139)
Supplement: Supplementary Materials — Figure S1. Workflow of the analysis. Figure S2. Validation of the risk score model using the GSE54460 dataset. A. Patients with prostate cancer (PRAD) in the GSE54460 cohort are listed in ascending order of risk score. B. Progression-free interval (PFI) distribution versus the risk score of each patient in the GSE54460 cohort. C. Kaplan–Meier (KM) curves of patients with different risk levels in the GSE54460 validation set. D. Receiver Operating Characteristic (ROC) curve analysis for 1-, 3- and 5-year PFI using the clinical information of patients of the GSE54460 validation dataset. Figure S3. Validation of the risk score model using the GSE46602 dataset. A. Patients with prostate cancer (PRAD) in the GSE46602 cohort are listed in ascending order of risk score. B. Progression-free interval (PFI) distribution versus the risk score of each patient in the GSE46602 cohort. C. Kaplan–Meier (KM) curves of patients with different risk levels in the GSE46602 validation dataset. D. Receiver Operating Characteristic (ROC) curve analysis for 1-, 3- and 5-year PFI using the clinical information of patients of the GSE46602 validation dataset. Figure S4. Validation of the risk score model using the GSE70768 dataset. A. Patients with prostate cancer (PRAD) in the GSE70768 cohort are listed in ascending order of risk score. B. Progression-free interval (PFI) distribution versus the risk score of each patient in the GSE70768 cohort. C. Kaplan–Meier (KM) curves of patients with different risk levels in the GSE70768 validation dataset. D. Receiver Operating Characteristic (ROC) curve analysis for 1-, 3- and 5-year PFI using the clinical information of patients of the GSE70768 validation dataset. Figure S5. Validation of the risk score model using the GSE70769 dataset. A. Patients with prostate cancer (PRAD) in the GSE70769 validation dataset are listed in ascending order of risk score. B. Progression-free interval (PFI) distribution versus the risk score of each patient in the GSE707 [file 6768139.f1.zip › 6768139.f1/Table S8.pdf]

ONTOLOGY	ID	Description	GeneRatio	p.adjust	Count
BP	GO:0006613	cotranslational protein targeting to membrane	80/443	5.37E-104	80
BP	GO:0006614	SRP-dependent cotranslational protein targeting to membrane	78/443	7.90E-103	78
BP	GO:0000184	nuclear-transcribed mRNA catabolic process, nonsense-mediated decay	79/443	1.49E-97	79
BP	GO:0045047	protein targeting to ER	79/443	1.49E-97	79
BP	GO:0072599	establishment of protein localization to endoplasmic reticulum	79/443	6.96E-96	79
BP	GO:0070972	protein localization to endoplasmic reticulum	83/443	7.08E-93	83
BP	GO:0019083	viral transcription	84/443	2.19E-86	84
BP	GO:0006413	translational initiation	85/443	8.79E-85	85
BP	GO:0019080	viral gene expression	84/443	4.64E-82	84
BP	GO:0000956	nuclear-transcribed mRNA catabolic process	83/443	2.42E-78	83
BP	GO:0006612	protein targeting to membrane	82/443	3.06E-76	82
BP	GO:0090150	establishment of protein localization to membrane	86/443	2.45E-60	86
BP	GO:0006402	mRNA catabolic process	87/443	2.79E-59	87
BP	GO:0006605	protein targeting	90/443	2.63E-56	90
BP	GO:0006401	RNA catabolic process	87/443	1.89E-55	87
BP	GO:0002181	cytoplasmic translation	36/443	1.64E-30	36
BP	GO:0042254	ribosome biogenesis	41/443	3.73E-17	41
BP	GO:0042255	ribosome assembly	21/443	1.45E-16	21
BP	GO:0022613	ribonucleoprotein complex biogenesis	45/443	3.02E-13	45
BP	GO:0042273	ribosomal large subunit biogenesis	18/443	9.09E-12	18
BP	GO:0042274	ribosomal small subunit biogenesis	18/443	1.87E-11	18
BP	GO:0006364	rRNA processing	27/443	9.93E-10	27
BP	GO:0016072	rRNA metabolic process	27/443	2.62E-09	27
BP	GO:0000028	ribosomal small subunit assembly	9/443	8.02E-08	9
BP	GO:0035966	response to topologically incorrect protein	22/443	6.02E-07	22
BP	GO:0000027	ribosomal large subunit assembly	9/443	1.50E-06	9
BP	GO:0006986	response to unfolded protein	20/443	2.34E-06	20
BP	GO:0034248	regulation of cellular amide metabolic process	34/443	3.57E-06	34
BP	GO:0097193	intrinsic apoptotic signaling pathway	24/443	9.42E-06	24
BP	GO:0022618	ribonucleoprotein complex assembly	21/443	1.12E-05	21
BP	GO:0034470	ncRNA processing	29/443	1.19E-05	29
BP	GO:0006417	regulation of translation	30/443	1.23E-05	30
BP	GO:0071826	ribonucleoprotein complex subunit organization	21/443	1.72E-05	21
BP	GO:0034976	response to endoplasmic reticulum stress	24/443	1.86E-05	24
BP	GO:0001836	release of cytochrome c from mitochondria	10/443	6.28E-05	10
BP	GO:0006457	protein folding	20/443	7.88E-05	20
BP	GO:0034660	ncRNA metabolic process	31/443	7.88E-05	31
BP	GO:2001242	regulation of intrinsic apoptotic signaling pathway	16/443	0.0001329	16
BP	GO:0045444	fat cell differentiation	19/443	0.000135	19
BP	GO:0043618	regulation of transcription from RNA polymerase II promoter in response to stress	13/443	0.0001718	13
BP	GO:0019883	antigen processing and presentation of endogenous antigen	7/443	0.0001718	7
BP	GO:0030968	endoplasmic reticulum unfolded protein response	14/443	0.0001746	14
BP	GO:0038066	p38MAPK cascade	9/443	0.0001746	9
BP	GO:0035967	cellular response to topologically incorrect protein	16/443	0.0002192	16
BP	GO:0034620	cellular response to unfolded protein	15/443	0.0002219	15
BP	GO:0034975	protein folding in endoplasmic reticulum	5/443	0.0002618	5
BP	GO:0045727	positive regulation of translation	14/443	0.0002694	14
BP	GO:0043620	regulation of DNA-templated transcription in response to stress	13/443	0.0002694	13
BP	GO:0002483	antigen processing and presentation of endogenous peptide antigen	6/443	0.0002906	6
BP	GO:0051384	response to glucocorticoid	14/443	0.0003046	14
BP	GO:0034101	erythrocyte homeostasis	13/443	0.0004484	13
BP	GO:0034250	positive regulation of cellular amide metabolic process	15/443	0.0005843	15
BP	GO:0042110	T cell activation	28/443	0.000651	28
BP	GO:1901798	positive regulation of signal transduction by p53 class mediator	6/443	0.0006721	6
BP	GO:0019058	viral life cycle	23/443	0.0006721	23
BP	GO:2001233	regulation of apoptotic signaling pathway	23/443	0.0006721	23
BP	GO:0036499	PERK-mediated unfolded protein response	6/443	0.0008581	6
BP	GO:0060337	type I interferon signaling pathway	11/443	0.0010157	11
BP	GO:0031960	response to corticosteroid	14/443	0.0010185	14
BP	GO:0036003	positive regulation of transcription from RNA polymerase II promoter in response to stress	6/443	0.0010185	6
BP	GO:0022407	regulation of cell-cell adhesion	26/443	0.0010185	26
BP	GO:0001701	in utero embryonic development	22/443	0.0010185	22
BP	GO:0071357	cellular response to type I interferon	11/443	0.0010185	11
BP	GO:0044409	entry into host	14/443	0.0010185	14
BP	GO:0050671	positive regulation of lymphocyte proliferation	13/443	0.0011657	13
BP	GO:0032946	positive regulation of mononuclear cell proliferation	13/443	0.0012428	13
BP	GO:0030099	myeloid cell differentiation	25/443	0.0013301	25
BP	GO:0034340	response to type I interferon	11/443	0.0015546	11
BP	GO:0046718	viral entry into host cell	13/443	0.0016126	13
BP	GO:0045785	positive regulation of cell adhesion	25/443	0.0016126	25
BP	GO:0006984	ER-nucleus signaling pathway	8/443	0.001801	8
BP	GO:0140467	integrated stress response signaling	6/443	0.0018395	6
BP	GO:0019885	antigen processing and presentation of endogenous peptide antigen via MHC class I	5/443	0.0019663	5
BP	GO:0050792	regulation of viral process	15/443	0.0019809	15
BP	GO:0002262	myeloid cell homeostasis	13/443	0.0021593	13
BP	GO:0071243	cellular response to arsenic-containing substance	5/443	0.0025649	5
BP	GO:0070665	positive regulation of leukocyte proliferation	13/443	0.0025817	13
BP	GO:1903320	regulation of protein modification by small protein conjugation or removal	17/443	0.0025817	17
BP	GO:1901653	cellular response to peptide	23/443	0.0029857	23
BP	GO:0007159	leukocyte cell-cell adhesion	22/443	0.0030899	22
BP	GO:0046685	response to arsenic-containing substance	6/443	0.0030899	6
BP	GO:0002689	negative regulation of leukocyte chemotaxis	5/443	0.0031642	5
BP	GO:0034341	response to interferon-gamma	15/443	0.0033791	15
BP	GO:0043903	regulation of biological process involved in symbiotic interaction	15/443	0.0033791	15
BP	GO:0052126	movement in host environment	14/443	0.0034115	14
BP	GO:0045861	negative regulation of proteolysis	21/443	0.0036761	21
BP	GO:2001244	positive regulation of intrinsic apoptotic signaling pathway	8/443	0.0036761	8
BP	GO:0030218	erythrocyte differentiation	11/443	0.0040096	11
BP	GO:1990440	positive regulation of transcription from RNA polymerase II promoter in response to endoplasmic reticulum stress	4/443	0.0040939	4
BP	GO:0008637	apoptotic mitochondrial changes	11/443	0.0048986	11
BP	GO:2000117	negative regulation of cysteine-type endopeptidase activity	9/443	0.0048986	9
BP	GO:1903037	regulation of leukocyte cell-cell adhesion	20/443	0.0051885	20
BP	GO:0051098	regulation of binding	21/443	0.0052976	21
BP	GO:0008630	intrinsic apoptotic signaling pathway in response to DNA damage	10/443	0.0054093	10
BP	GO:0050870	positive regulation of T cell activation	15/443	0.0067923	15
BP	GO:0051403	stress-activated MAPK cascade	17/443	0.0068398	17
BP	GO:0070841	inclusion body assembly	5/443	0.00709	5
BP	GO:1904666	regulation of ubiquitin protein ligase activity	5/443	0.00709	5
BP	GO:0036498	IRE1-mediated unfolded protein response	8/443	0.0071289	8
BP	GO:0009991	response to extracellular stimulus	25/443	0.0071289	25
BP	GO:2001235	positive regulation of apoptotic signaling pathway	11/443	0.007924	11
BP	GO:1901654	response to ketone	14/443	0.0080433	14
BP	GO:0052548	regulation of endopeptidase activity	23/443	0.0081773	23
BP	GO:1902229	regulation of intrinsic apoptotic signaling pathway in response to DNA damage	6/443	0.0081985	6
BP	GO:1903901	negative regulation of viral life cycle	5/443	0.0081985	5
BP	GO:0062197	cellular response to chemical stress	20/443	0.0087917	20
BP	GO:0001666	response to hypoxia	20/443	0.0090444	20
BP	GO:0010951	negative regulation of endopeptidase activity	16/443	0.0091453	16
BP	GO:0050670	regulation of lymphocyte proliferation	15/443	0.009317	15
CC	GO:0022626	cytosolic ribosome	80/450	2.91E-107	80

CC	GO:0044391	ribosomal subunit	84/450	1.25E-85	84
CC	GO:0005840	ribosome	89/450	1.97E-81	89
CC	GO:0022625	cytosolic large ribosomal subunit	46/450	7.74E-64	46
CC	GO:0015934	large ribosomal subunit	49/450	8.56E-48	49
CC	GO:0022627	cytosolic small ribosomal subunit	34/450	1.38E-44	34
CC	GO:0015935	small ribosomal subunit	35/450	5.80E-36	35
CC	GO:0005925	focal adhesion	67/450	2.15E-35	67
CC	GO:0030055	cell-substrate junction	67/450	5.60E-35	67
CC	GO:0042788	polysomal ribosome	23/450	1.30E-29	23
CC	GO:0005844	polysome	28/450	1.07E-26	28
CC	GO:0005791	rough endoplasmic reticulum	15/450	5.22E-09	15
CC	GO:0014069	postsynaptic density	27/450	1.58E-07	27
CC	GO:0032279	asymmetric synapse	27/450	2.06E-07	27
CC	GO:0099572	postsynaptic specialization	27/450	5.12E-07	27
CC	GO:0098984	neuron to neuron synapse	27/450	8.38E-07	27
CC	GO:0042470	melanosome	13/450	2.45E-05	13
CC	GO:0048770	pigment granule	13/450	2.45E-05	13
CC	GO:0098576	lumenal side of membrane	8/450	3.01E-05	8
CC	GO:0071556	integral component of lumenal side of endoplasmic reticulum membrane	7/450	7.04E-05	7
CC	GO:0098553	lumenal side of endoplasmic reticulum membrane	7/450	7.04E-05	7
CC	GO:0030176	integral component of endoplasmic reticulum membrane	14/450	0.0003428	14
CC	GO:0042611	MHC protein complex	6/450	0.0003428	6
CC	GO:0031227	intrinsic component of endoplasmic reticulum membrane	14/450	0.0005515	14
CC	GO:0030134	COPII-coated ER to Golgi transport vesicle	10/450	0.0010883	10
CC	GO:0034774	secretory granule lumen	20/450	0.0010883	20
CC	GO:0060205	cytoplasmic vesicle lumen	20/450	0.0012423	20
CC	GO:0031983	vesicle lumen	20/450	0.0013027	20
CC	GO:0012507	ER to Golgi transport vesicle membrane	8/450	0.0013067	8
CC	GO:0005788	endoplasmic reticulum lumen	19/450	0.0014369	19
CC	GO:0030867	rough endoplasmic reticulum membrane	5/450	0.0040295	5
CC	GO:0030139	endocytic vesicle	18/450	0.0041283	18
CC	GO:0098554	cytoplasmic side of endoplasmic reticulum membrane	4/450	0.004192	4
CC	GO:0030864	cortical actin cytoskeleton	8/450	0.0056092	8
MF	GO:0003735	structural constituent of ribosome	84/442	1.94E-85	84
MF	GO:0019843	rRNA binding	20/442	6.75E-15	20
MF	GO:0045296	cadherin binding	31/442	2.76E-08	31
MF	GO:0048027	mRNA 5'-UTR binding	8/442	1.82E-05	8
MF	GO:0051087	chaperone binding	13/442	0.000118	13
MF	GO:0098631	cell adhesion mediator activity	9/442	0.0010063	9
MF	GO:0044389	ubiquitin-like protein ligase binding	21/442	0.0022256	21
MF	GO:0031625	ubiquitin protein ligase binding	20/442	0.0023608	20
MF	GO:0051082	unfolded protein binding	12/442	0.0023608	12
MF	GO:0055106	ubiquitin-protein transferase regulator activity	5/442	0.0033546	5
MF	GO:0001228	DNA-binding transcription activator activity, RNA polymerase II-specific	25/442	0.0046295	25
MF	GO:0001216	DNA-binding transcription activator activity	25/442	0.0048251	25
MF	GO:0043021	ribonucleoprotein complex binding	12/442	0.0048251	12
MF	GO:0035259	glucocorticoid receptor binding	4/442	0.0064423	4
